# Intra- and intermolecular Fe-catalyzed dicarbofunctionalization of vinyl cyclopropanes†

**DOI:** 10.1039/d0sc00467g

**Published:** 2020-02-27

**Authors:** Lei Liu, Wes Lee, Mingbin Yuan, Chris Acha, Michael B. Geherty, Brandon Williams, Osvaldo Gutierrez

**Affiliations:** Department of Chemistry and Biochemistry, University of Maryland College Park Maryland 20742 USA ogs@umd.edu

## Abstract

Design and implementation of the first (asymmetric) Fe-catalyzed intra- and intermolecular difunctionalization of vinyl cyclopropanes (VCPs) with alkyl halides and aryl Grignard reagents has been realized *via* a mechanistically driven approach. Mechanistic studies support the diffusion of alkyl radical intermediates out of the solvent cage to participate in an intra- or intermolecular radical cascade with a range of VCPs followed by re-entering the Fe radical cross-coupling cycle to undergo (stereo)selective C(sp^2^)–C(sp^3^) bond formation. This work provides a proof-of-concept of the use of vinyl cyclopropanes as synthetically useful 1,5-synthons in Fe-catalyzed conjunctive cross-couplings with alkyl halides and aryl/vinyl Grignard reagents. Overall, we provide new design principles for Fe-mediated radical processes and underscore the potential of using combined computations and experiments to accelerate the development of challenging transformations.

## Abstract 2

Design and implementation of the first (asymmetric) Fe-catalyzed intra- and intermolecular difunctionalization of vinyl cyclopropanes (VCPs) with alkyl halides and aryl Grignard reagents has been realized *via* a mechanistically driven approach.

## Graphical abstract

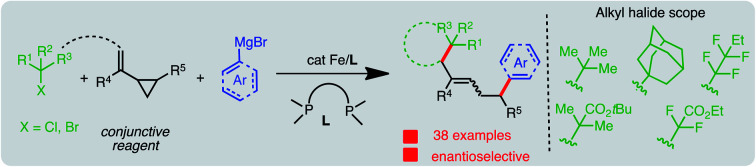

## Introduction

**Scheme 1:**
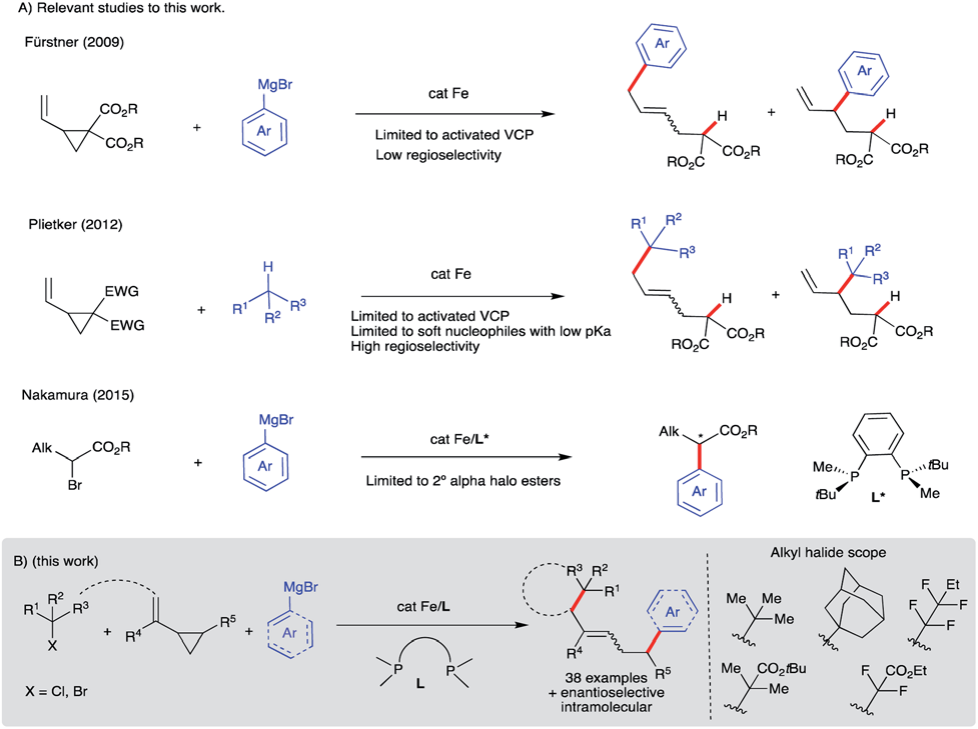
Design and development of Fe-catalyzed intra- and inter-molecular difunctionalization of vinyl cyclopropanes *via* a new radical cascade reaction.

Iron-catalyzed C–C cross-coupling reactions have attracted much attention due to the higher abundance, cost-effectiveness, and lower toxicity of iron in comparison to precious transition metals.^1^ Methods for ligand-supported (*e.g.*, N-heterocycles, bisphosphines, and diamines) and ligand-free systems for iron-catalyzed C–C cross-coupling reactions using C(sp), C(sp^2^), and C(sp^3^) partners have been developed.^2^ In particular, bisphosphine-iron systems have emerged as highly versatile and promising candidates for the formation of new C–C bonds with a range of organometallic nucleophiles including Mg- (Kumada),^3^ Zn- (Negishi),^4^ B- (Suzuki–Miyaura),^5^ and Al^6^ reagents with alkyl halides and redox active esters.^4*a*^ Electron-poor vinyl cyclopropanes have also been used as π-coupling partners in Fe-catalyzed C(sp^2^)–C(sp^3^) bond formation (Scheme 1A). In particular, Fürstner used low valent iron ferrates to promote tandem ring-opening/*mono*arylation of *electron-poor* vinyl cyclopropanes (VCPs) using aryl Grignard reagents.^7^ In a related study, Plietker used a low valent, electron rich ferrate complex (Bu_4_N[Fe(CO)_3_(NO)]) to promote ring-opening/*mono*arylation of *electron-deficient* VCPs with acidic pronucleophiles.^8^ Despite these advancements, to the best of our knowledge, there are only two reports of asymmetric iron-catalyzed cross-couplings: between aryl Grignard reagents or lithium aryl borates as nucleophiles and α-halo esters as electrophiles (Scheme 1A).^9,10^ Further, despite the use of vinylcyclopropanes as useful reagents in organic synthesis, the application of VCPs in Fe-catalyzed conjunctive cross-couplings is not known. Thus, development of new (asymmetric) iron-catalyzed radical cascade/C(sp^2^)–C(sp^3^) cross-coupling reactions will expand the synthetic toolbox and lead to an increase in diversification of carbocycles.^11^ Herein, we used a mechanistically guided approach to design and develop a new and selective Fe-catalyzed *intra*- and *inter*-molecular dicarbofunctionalization of synthetically versatile vinyl cyclopropanes (Scheme 1B).^12,13^ Conceptually, this work establishes the use of readily accessible vinyl cyclopropanes in Fe-catalyzed conjunctive cross-coupling reactions.

**Scheme 2:**
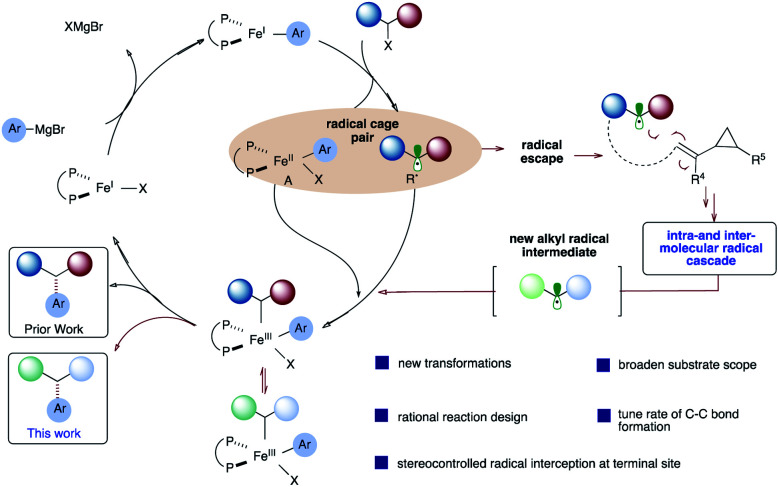
A proposed mechanism of iron-catalyzed cross-coupling reactions.

Contributing to the scarcity of iron-catalyzed asymmetric reactions is likely the fact that mechanistic details of iron-catalyzed cross-coupling are not well understood, in comparison to palladium systems.^14^ Pioneering mechanistic studies by Kochi^15^ in the 1970s and more recent reports by Bedford,^16^ Nakamura,^17^ Norrby,^18^ Fürstner,^19^ Tonzetich,^20^ Koszinowski,^21^ and Neidig^22^ have led to a greater understanding of these transformations. In 2017, parallel quantum mechanical studies in our lab^23^ and by Morokuma^24^ were reported on the mechanism of chiral bisphosphine cross-coupling reactions between α-chloro esters and aryl Grignard reagents. These studies revealed a mechanism involving halogen abstraction by an aryl Fe(i) complex, leading to an alkyl radical and halo aryl Fe(ii) species (Scheme 2; circled). In turn, these two species could combine, leading to an Fe(iii) intermediate which will then undergo reductive elimination, leading to the desired cross-coupled product. More experimental studies (*i.e.*, spectroscopic and kinetic) are needed to assess the validity of the computational models, and the mechanism likely depends on subtle changes to the alkyl halide, Grignard, and ligand structures. Nonetheless, based on these mechanistic studies, we envisage diverting the reactivity from the Fe radical cross-coupling catalytic cycle (black) to a programmed *intra*- and *inter*molecular radical cascade (red) with vinyl cyclopropanes, leading to a new alkyl radical that could then re-enter the catalytic cycle and undergo stereoselective C(sp^2^)–C(sp^3^) bond formation. Given that most of the transition-metal catalyzed cascade reactions terminate with C–H bond formation^25^ and fail to control the stereoselectivity at the termination step, if successful, this approach could lead to a rapid increase in molecular diversity.

## Results and discussion

### Stereoselective *intra*-molecular dicarbofunctionalization of VCPs

Li's group demonstrated the use of cyclopropyl olefins to promote a radical alkylation, ring-opening, intramolecular arylation cascade reaction under photoredox conditions.^26^ Fu and co-workers reported that chiral nickel catalysts could achieve enantioselective cross-coupling of a wide range of racemic alkyl halides (as radical precursors) with organometallic nucleophiles^27^ including those involving stereoconvergent radical cyclization/arylation.^28^ We hypothesize that upon radical formation and in the presence of a pendant vinyl cyclopropane (Scheme 2; right), we could divert reactivity from the cross-coupling cycle to promote an *intra*-molecular radical cascade reaction. Specifically, we envisage a 5-*exo*-trig cyclization outcompeting radical rebound to an aryl Fe species and divert reactivity towards *Fe-catalyzed 1,5-dicarbofunctionalization of vinyl cyclopropanes*.

**Scheme 3:**
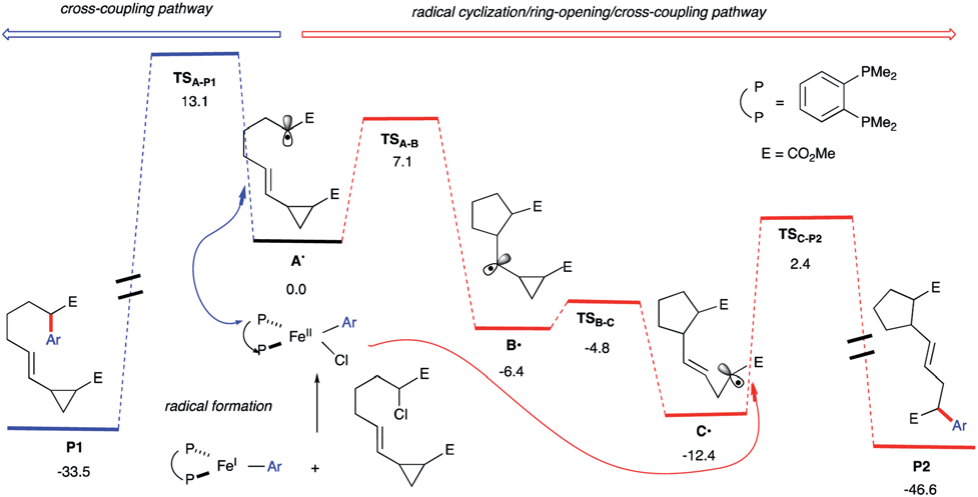
Energetics for the in-cage (blue) and out-of-cage (red) arylation computed at the UPBEPBE/6-311+G(d,p)-SDD(Fe)-THF(SMD)//UB3LYP/6-31G(d) levels of theory.

Indeed, as shown in Scheme 3, quantum mechanical calculations support our hypothesis. Specifically, the barrier for radical 5-*exo*-cyclization (*via***TSA-B**) to form **B˙** is 6.0 kcal mol^−1^ lower in energy than the barrier for the radical rebound transition state **TSA-P1** that will lead to cross-coupling product **P1** (7.1 kcal mol^−1^*vs.* 13.1 kcal mol^−1^, respectively). In turn, the kinetically favored cyclic alkyl radical **B˙** will then undergo radical ring-opening (the barrier is only 1.6 kcal mol^−1^) to form the thermodynamically favored alkyl radical **C˙**. Finally, **C˙** could then re-enter the iron cross-coupling cycle and undergo C(sp^2^)–C(sp^3^) bond formation (*via***TSC-P2**; the barrier is 14.8 kcal mol^−1^ from **C˙**) leading, after reductive elimination (not shown), to the radical cascade product **P2**. Overall, these calculations suggest that the radical cyclization/ring-opening/cross-coupling pathway (leading to **P2**) is kinetically favored over cross-coupling (leading to **P1**). Moreover, we anticipate that the chiral aryl iron species could control the final radical coupling step (with **C˙**) and permit high-levels of stereocontrol.^29^

**Scheme 4:**
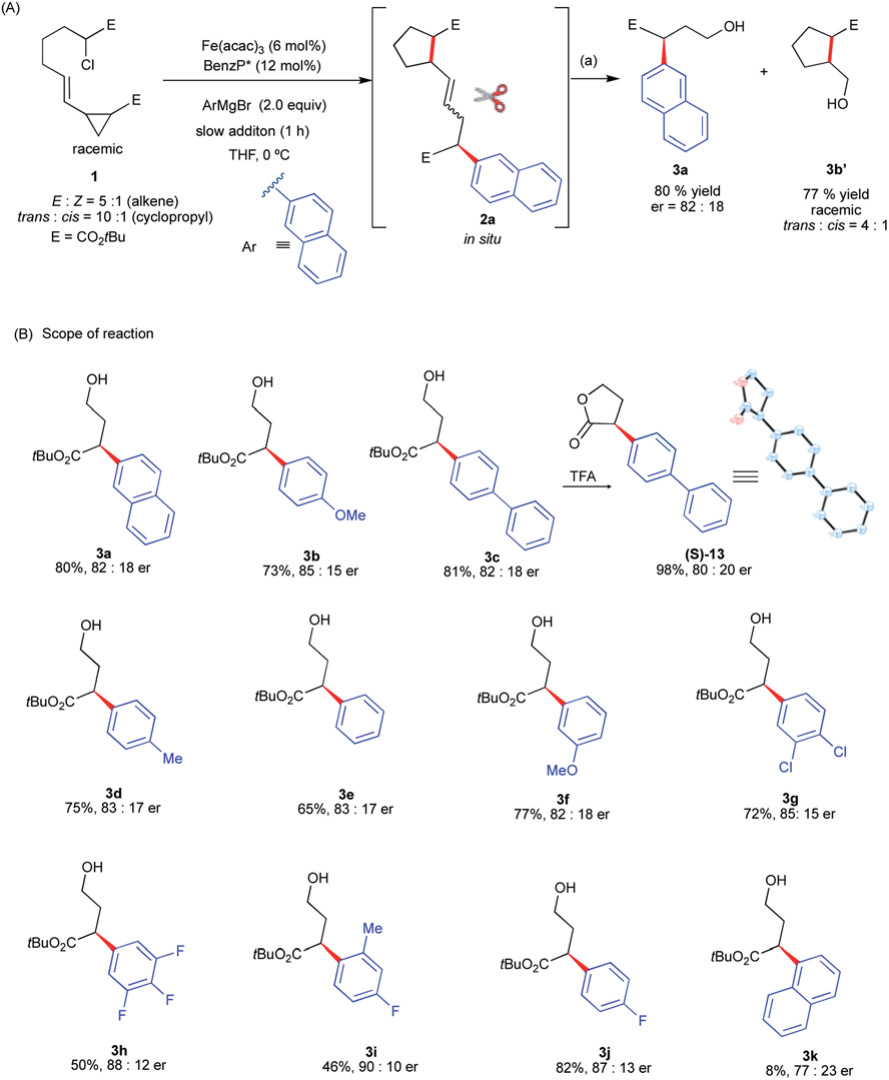
Design and application of asymmetric Fe-catalyzed intra-molecular dicarbofunctionalization of vinyl cyclopropanes. (a) O_3_, CH_2_Cl_2_/MeOH −78 °C, 5 min and then NaBH_4_ (10 equiv.) −78 °C to rt, 1 h.

Gratifyingly, we found that we can divert reactivity towards the *asymmetric intra*-molecular 1,5-difunctionalization of vinyl cyclopropanes using **1** as the substrate (Scheme 4). Specifically, using the standard conditions for Fe-catalyzed α-arylation,^30^ the reaction of **1** with 2-napthylmagnesium bromide gave a mixture of diastereomeric radical cascade products **2a** (see ESI†). Using column chromatography, we separated and identified the *trans*, *E* stereoisomer as the major diastereomer (see ESI†). At this moment, noting that most of the transition-metal catalyzed cascade reactions terminate with C–H bond formation^25^ and fail to control stereoselectivity at the termination step, we were primarily concerned with determining if the chiral iron species controlled the stereochemistry of the terminating C(sp^2^)–C(sp^3^) arylation. As a proof-of-concept, to determine stereochemistry at the terminating C–C bond forming step, we subjected **2a** to a one-pot procedure of ozonolysis followed by reduction with NaBH_4_ that led to the corresponding product **3a** in good yields and enantioselectivities. Notably, as expected from the radical cyclization event in the absence of chiral iron species (Scheme 3), the corresponding *racemic* cyclopentane fragment **3b′** was isolated in 77% yield in 4 : 1 dr. Having established, as a proof-of-concept, that we could control the enantioselectivity at the terminating step in the cascade reaction, we examine the aryl Grignard reagent scope in this transformation and its effect on enantioselectivity. As shown in Scheme 4B, the aryl Grignard reagent scope is broad. Both electron-poor and electron-rich aryl Grignard reagents participate in the diverted Fe-catalyzed intra-molecular difunctionalization of vinyl cyclopropanes to give the desired products in good yields (up to 82% over three steps) and enantioselectivities (up to 90 : 10 er). We established the absolute stereochemistry as (*S*) from the cyclized lactone (*S*)-**13** from **3c**. Notably, Grignard reagents that failed in the asymmetric Fe-catalyzed cross-coupling reactions were compatible reagents in these radical cascade/cross-coupling transformations. Specifically, highly electron-deficient (*i.e.*, 3,4,5-trifluorophenyl **3h**) and even sterically congested (*e.g.*, *ortho*-methyl aryl **3i** and 1-naphthyl **3k**) aryl Grignard reagents formed the desired products with good enantioselectivities (77 : 23 to 90 : 10 er) albeit in lower yields (8–50%). However, at this moment, this method is limited to 5-*exo*-trig radical cyclization. Lengthening the tether with an extra methylene group leads to the formation of the Fe radical cross-coupled product and no products from diverted 6-*exo*-trig radical cyclization are observed (see ESI†). Presumably, the much higher energy barrier to undergo radical 6-*exo*-trig cyclization in comparison to 5-*exo*-trig radical cyclization prevents the formation of radical cascade/arylation (see Fig. S9 in the ESI† for energetics).

### Design and application of *inter*-molecular dicarbo-functionalization of vinyl cyclopropanes

**Scheme 5:**
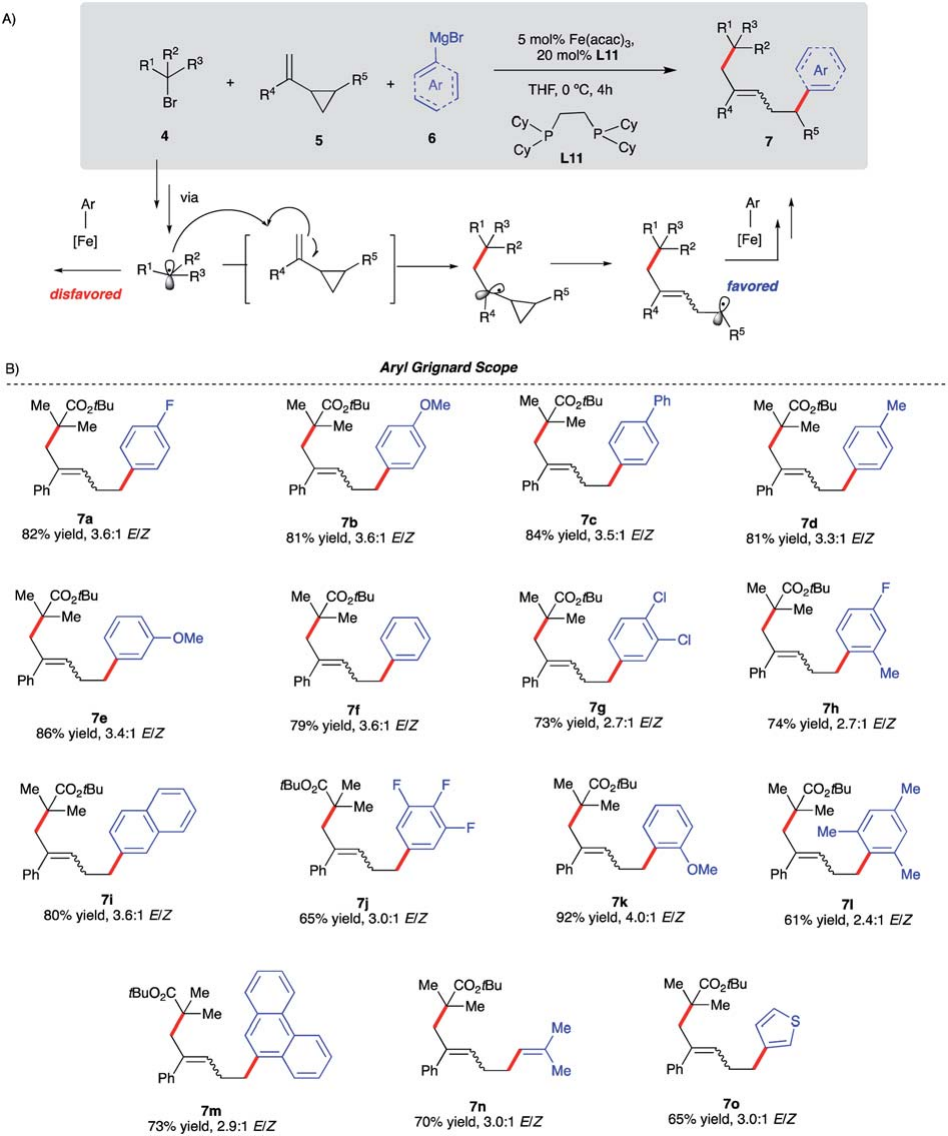
Reaction scope of the aryl Grignard reagent in Fe-catalyzed intermolecular dicarbofunctionalization of vinyl cyclopropanes. (a) Reactions were performed on a 0.20 mmol scale. (b) ArMgBr was added dropwise *via* a syringe pump over 4 h. (c) Isolated yield.

Given the scarcity of stereoconvergent transition metal-catalyzed radical cyclization-arylation cascades and underutilization of vinyl cyclopropanes in transition metal-catalyzed conjunctive cross-couplings, our proof-of-principle results with *intra*molecular functionalization of VCPs (Scheme 4) represent an attractive strategy towards this unmet need.^31,32^ Specifically, we envisage that by tuning the properties of alkyl halides, we could divert reactivity towards regioselective *inter*molecular ring-opening/dicarbofunctionalization of VCPs. Specifically, we hypothesize that sterically hindered *tertiary* alkyl halides will lead to *higher* barriers for *Fe radical coupling* and, instead, favor *inter*molecular radical addition to the vinyl cyclopropane (Scheme 5A). In turn, the incipient radical could then undergo cyclopropyl ring-opening and re-enter the Fe radical cross-coupling cycle to undergo (stereoselective) C(sp^2^)–C(sp^3^) arylation at the least sterically hindered site. Previous work by Fürstner and Plietker using iron as the catalyst with vinyl cyclopropanes (VCPs) resulted in ring-opening/monocarbofunctionalization, required electron-deficient VCPs, and terminated in protonation (Scheme 1A).^7,8^ Thus, if successful, this approach will expand the range of Fe-catalyzed transformations using vinyl cyclopropanes as valuable synthons in chemical synthesis. Also, if realized, this will represent the first example of (asymmetric) Fe-catalyzed 3-component cross-coupling.

**Scheme 6:**
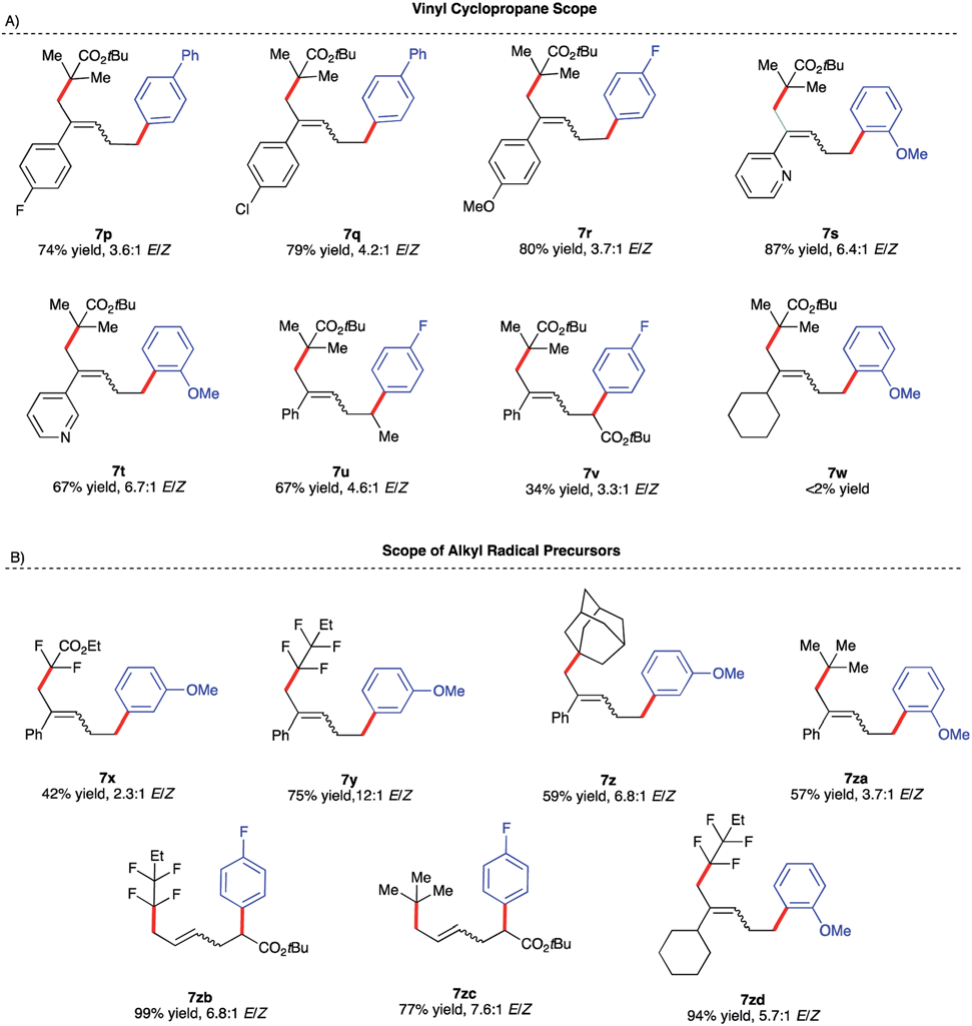
Vinyl cyclopropane and alkyl halide reaction scope. (a) Reactions were performed on a 0.20 mmol scale. (b) ArMgBr was added dropwise *via* a syringe pump over 4 h. (c) Isolated yield.

We initiated our optimization studies using sterically hindered *tert*-butyl 2-bromo-2-methylpropanoate **4**, (1-cyclopropylvinyl)-benzene **5** and aryl 4-fluorophenylmagnesium bromide **6** as model substrates (Scheme 5). Gratifyingly, after extensive ligand screening using bisphosphine, monophosphine, and diamine ligands (see Table S2 in the ESI†) and optimization studies, we identified acyclic 1,2-bis(dicyclohexylphosphino)ethane ligand **L11** to be suitable to promote the desired and highly selective ring-opening/1,5-alkylarylation of vinyl cyclopropanes, forming compound **7a** in 82% isolated yield and 3.6 : 1 *E*/*Z* ratio (Scheme 5B). With optimized conditions in hand, we next investigated the reaction scope of this transformation. As shown in Scheme 5B, the scope of this Fe-catalyzed 3-component dicarbofunctionalization is broad with respect to the Grignard nucleophile. Specifically, the reaction tolerated both withdrawing and electron-donating aryl nucleophiles, forming the desired 1,5-alkylaryl products in 61–92% yield. Notably, the reaction tolerated sterically hindered *ortho*-substituted aryl Grignard reagents (**7h**, **7k**, **7l**, and **7m**) that proved to be problematic in previous direct Fe-catalyzed cross-coupling reactions.^33^ To highlight the versatility of this method, we also used both *vinyl* and *hetero*aryl Grignard reagents and, gratifyingly, obtained the desired products **7n** and **7o**, in good yields, 70% and 65% yield, respectively. Next, we explored the scope of vinyl cyclopropane and alkyl radical precursors (Scheme 6). Installing electron-rich or electron-poor aryl groups in the vinyl cyclopropane moiety (R^4^) allows the formation of the desired products in good to excellent yields (Scheme 6A). Notably, the reaction tolerates medicinally relevant 2- and 3-pyridyl moieties (**7s**, **7t**). Moreover, the substituted cyclopropanes (R^5^) were suitable for this catalytic system, affording **7u** and **7v** in 67% and 34% yield, respectively. Unfortunately, sterically hindered *tert*-butyl 2-bromo-2-methylpropanoate **4** with alkyl VCPs such as **7w** are beyond the reach of the present Fe-based system. In contrast to previously reported Fe-catalyzed cross-couplings, we found a wide range of alkyl bromides to be suitable radical precursors in this Fe-catalyzed conjunctive cross-coupling (Scheme 6B). Specifically, we found that alkyl fluorinated radical precursors are effective partners in the Fe-catalyzed intermolecular dicarbofunctionalization of VCPs, yielding the desired products **7x** and **7y** in 42% and 75% yield, respectively. Further, we observed a much higher *E*/*Z* ratio for **7y** (12 : 1 *E*/*Z*), although the origin of this high selectivity is currently unknown. Finally, this method tolerated unactivated 3˙ alkyl radical precursors which led to the desired 3-component products **7z** and **7za**. We also performed quantum mechanical calculations on the intermolecular dicarbofunctionalization, and the calculated results are consistent with our hypothesis and the experimental result (see Fig. S10 in the ESI† for energetics). Specifically, *tertiary* alkyl radicals preferably undergo *inter*-molecular radical addition (with a barrier of 14.0 kcal mol^−1^) to the vinyl cyclopropane over radical coupling with Fe (with a barrier of 18.2 kcal mol^−1^).

**Scheme 7:**
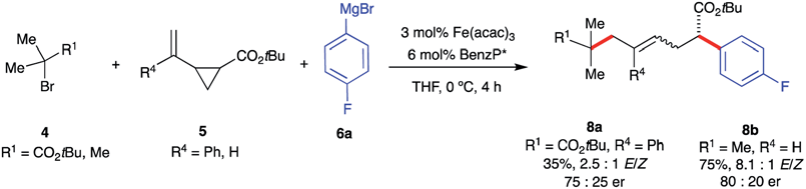
Preliminary results of asymmetric Fe-catalyzed intermolecular dicarbofunctionalization of vinyl cyclopropanes. (a) The enantiomeric ratio (er) values of (*E*)-**8a** and **8b** were determined using chiral HPLC analysis.

Interestingly, fluorinated and *tert*-butyl radical precursors in combination with unactivated vinyl cyclopropanes led to the desired products **7zb**, **7zc**, and **7zd** in excellent yields (77–99%). As observed for *intra*molecular Fe-catalyzed difunctionalization of VCPs (*vide supra*), preliminary results (Scheme 7) demonstrate that the chiral iron species controls the enantioselectivity at the terminal site of C–C bond formation in the presence of an ester moiety to form **8a** and **8b** in 35% and 75% yield, respectively. These results represent the first examples of an asymmetric Fe-catalyzed conjunctive cross-coupling. Ongoing work is geared towards expanding this transformation.

**Scheme 8:**
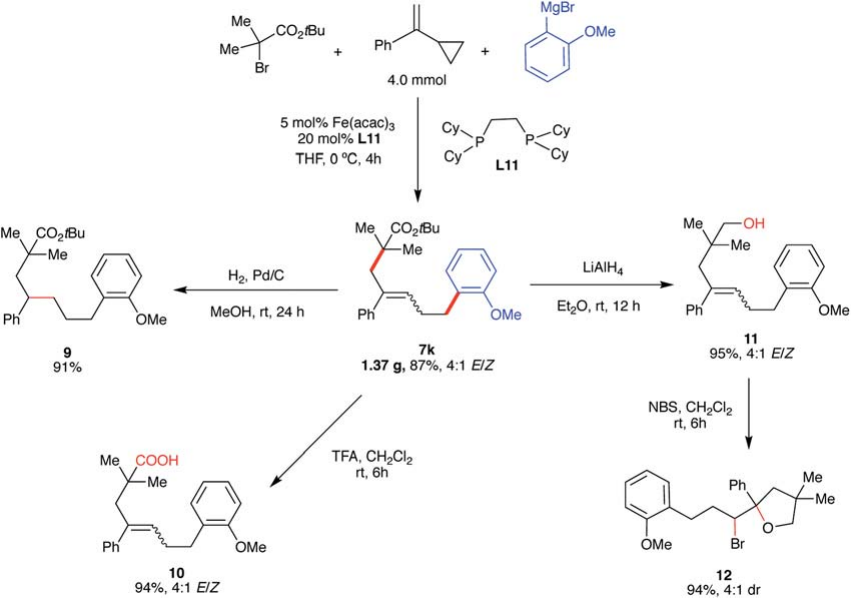
Synthetic applications of five-carbon unsaturated chain ester. (a) Isolated yield. (b) Yield of all isomers.

To demonstrate the synthetic versatility of the five-carbon unsaturated chain – potentially useful transformation, we performed the gram-scale synthesis of **7k** (1.37 g, 87% yield) and performed several diversifications (Scheme 8). First, reduction of the alkene (Pd/C) provided the alkyl ester (**9**). Next, we performed hydrolysis of the ester to carboxylic acid (**10**) which could be a useful substrate in, *inter alia*, subsequent decarboxylative radical cross-couplings.^34^ Notably, derivatization of **7k** to the carboxylic acid analog **10** permitted facile separation and purification of the major isomer (*E*). Finally, reduction of the ester formed the corresponding primary alcohol (**11**) which could undergo bromocyclization with *N*-bromosuccinimide (NBS) leading to the substituted tetrahydropyran with two stereogenic centers (**12**).

## Conclusions

In summary, Fe-catalyzed *intra*- and *inter*-molecular dicarbofunctionalizations of vinyl cyclopropanes have now been realized. In particular, we have used a mechanistically driven (computational and experimental) approach to divert the reactivity of the alkyl radical from the Fe radical cross-coupling cycle to undergo *intra*- and *inter*-molecular radical cascade reactions with activated and unactivated vinyl cyclopropanes. In turn, the incipient alkyl radical then re-enters the Fe cross-coupling cycle and undergoes (stereoselective) C(sp^2^)–C(sp^3^) bond formation. Finally, for the first time, as a proof-of-concept we show that VCPs can be used as effective conjunctive partners in asymmetric Fe-catalyzed 3-component cross-coupling reactions. We anticipate that, in particular, the intermolecular 3-component Fe-catalyzed dicarbofunctionalization reaction will impact the synthesis of medicinally relevant molecules. However, at present, some drawbacks of this method are the use of Grignard reagents that limits functional group incorporation into the nucleophile/radical precursors along with *E*/*Z* mixtures. Ongoing work is focused on expanding this strategy to a wide range of alkyl radical precursors, π-coupling partners, and nucleophilic partners participating in diverted asymmetric Fe-catalyzed radical cascade/cross-coupling reactions.

## Conflicts of interest

There are no conflicts to declare.

## Supplementary Material

SC-011-D0SC00467G-s001

SC-011-D0SC00467G-s002
