# Supplementary material for: Intra- and intermolecular Fe-catalyzed dicarbofunctionalization of vinyl cyclopropanes
Source: Chem Sci. 2020 Feb 27;11(12):3146–51. doi: 10.1039/d0sc00467g (PMC8157325; doi:10.1039/d0sc00467g)
Supplement: SC-011-D0SC00467G-s001 [file SC-011-D0SC00467G-s001.pdf]

Electronic Supplementary Material (ESI) for Chemical Science.
This journal is © The Royal Society of Chemistry 2020

Intra- and Intermolecular Fe-Catalyzed Dicarbofunctionalization of Vinyl Cyclopropanes

Lei Liu, Wes Lee, Mingbin Yuan, Chris Acha, Michael B. Geherty, Brandon Williams, and Osvaldo Gutierrez*[‡]

[‡]*Department of Chemistry and Biochemistry, University of Maryland, College Park, Maryland 20742, United States*

ogs@umd.edu

Supporting Information

Table of Contents

1. General Considerations S3
2. Preparation and Characterization of Materials S4
3. General Procedure of Iron-catalyzed Difunctionalization S17
4. Screening of Reaction Condition for Iron-Catalyzed Difunctionalization S21
5. Study Diastereoselectivity and β-Hydride Elimination of Radical Cascade S24
6. Comparison of Radical Cascade Arylation and Direct Arylation S28
7. Structural Confirmation of Product **2a’**, **2a** and **7** S29
8. Product Characterization Data S33
9. Determination of Absolute Stereochemistry S70
10. Iron-catalyzed Enantioselective Intermolecular Difunctionalization S71
11. Derivatizations of **7k** S74
12. Spectral Data S79
13. HPLC Chromatography of the Products S163
14. Computational Methods, Energies, and Coordinates S176
15. Crystallographic Data S276
16. References S282

1. General Considerations

Unless otherwise stated, all non-aqueous reactions were carried out under an atmosphere of dry nitrogen in oven- (150 °C) or flame-dried glassware. When necessary, solvents and reagents were dried prior to use. Dichloromethane (CH_2Cl_2) was distilled from calcium hydride. Tetrahydrofuran (THF) was dried by passage through activated alumina in Inert's PureSolv PS-MD-3 solvent purification system. Triethylamine (Et_3N) and diisopropylamine (*i*-Pr_2NH) were distilled over calcium hydride prior to use. All work-up and purification procedures used reagent grade solvents purchased from VRW, Sigma-Aldrich, or Fisher. Organometallic reagents were purchased from Sigma-Aldrich. Analytical thin layer chromatography (TLC) was performed on Silicycle 250 μm silica-gel F-254 plates. Isolera™ Flash Systems silica gel chromatography was performed on prepacked silica-gel cartridges (SNAP Ultra; Biotage). Purification via flash column chromatography was performed on silica gel 60 (230-400 mesh ASTM). ^1H NMR and ^{13}C NMR spectra were recorded on Bruker AV (400 MHz) and Bruker AV-III (600 MHz) NMR spectrometer. Chemical shifts (δ) are reported in parts per million (ppm) relative to the internal residual solvent resonance peak δ 7.26 ($CDCl_3$) and δ 0.00 (TMS) for ^1H and δ 77.16 ($CDCl_3$) and δ 0.00 (TMS) for ^{13}C. Data are reported as follows: chemical shift, multiplicity (s = singlet, d = doublet, t = triplet, q = quartet, p = quintet, b = broad singlet, m = multiplet, dd= doublet of doublets, dt = doublet of triplets, dq = doublet of quartets, td = triplet of doublets, qd = quartet of doublets, pd = quintet of doublets, ddd = doublet of doublet of doublets, ddt = doublet of doublet of triplets, dtd = doublet of triplet of doublets, dtt = doublet of triplet of triplets, tdd = triplet of doublet of doublets, qdd = quartet of doublet of doublets, dddd = doublet of doublet of doublet of doublets, dddt = doublet of doublet of doublet of triplets), coupling constants (*J)* are reported in Hertz (Hz), and number of protons. High Resolution Mass (HRMS) spectra using Electrospray Ionization (ESI) and DART modes were obtained using a JEOL AccuTOF-CS. Enantiomeric ratio values were determined by HPLC with Daicel Chiralcel OJ-H, AS-H and AD-H columns with hexane and *i*-PrOH as solvents. IR spectra were recorded on a Thermo Nicolet NEXUS 670 FTIR and are reported in wavenumbers (cm^{-1}). Optical rotations were measured on a JASCO P-2000 polarimeter. Melting points were obtained and are uncorrected.

2. Preparation and Characterization of Materials

Synthesis of α-chloro esters 1a

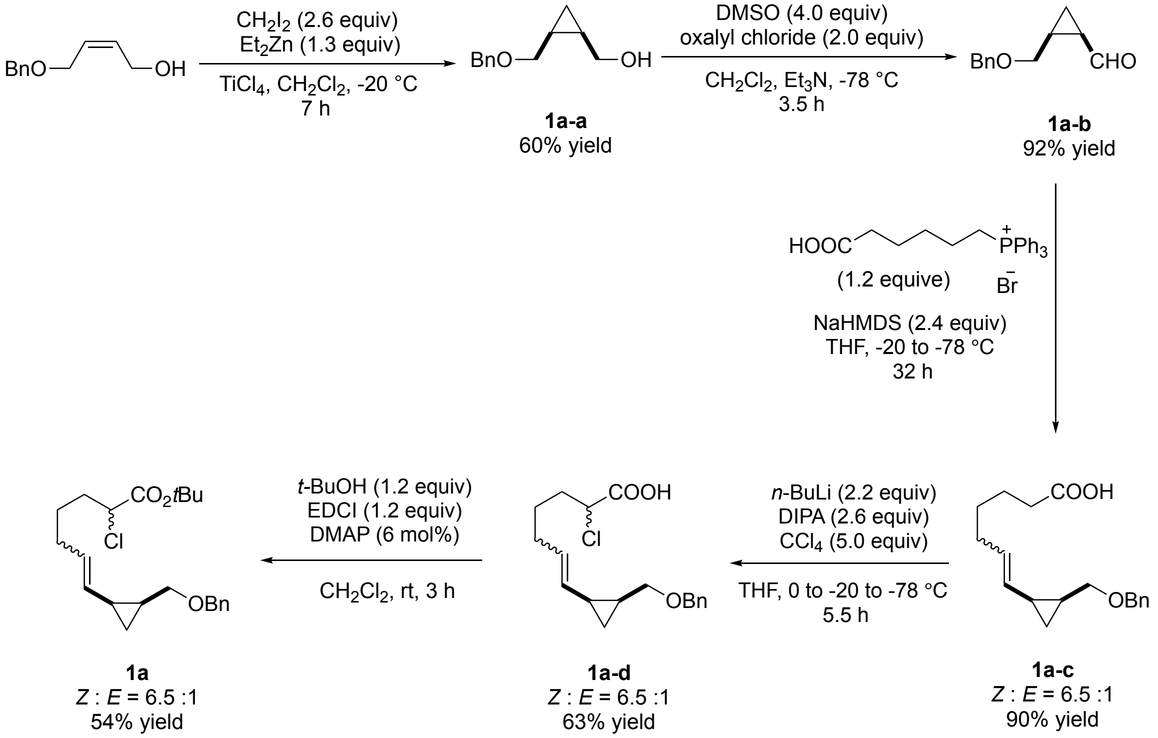

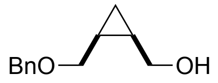

***cis*-2-(Benzyloxymethyl)cyclopropylmethanol (1a-a)** : Following a modified procedure by Charette et al.,[1] to a flame-dried 250 mL of round bottom flask equipped with a stir bar, diethylzinc (8.12 mL, 8.11 mmol, 1.0 M in Hexane) was added dropwise via syringe into a stirred solution of CH_2I_2 (1.3 mL, 16.22 mmol) in anhydrous CH_2Cl_2 (64 mL) at 0 °C under nitrogen. The resulting solution was stirred at that temperature for 15 min and a white precipitate was formed. The solution was cooled to -78 °C and a solution of (*Z*)-4-benzyloxy-2-butenol (1.05 mL, 6.24 mmol) in anhydrous CH_2Cl_2 (40 mL) was added dropwise via syringe. Then, the resulting heterogeneous solution was stirred at -20 °C for 15 min and titanium chloride solution (1.25 mL, 1.25 mmol, 1.0 M in CH_2Cl_2) was then added via syringe dropwise. After 3 h of stirring at -20 °C, the resulting solution was cooled at -40 °C and poured into an aqueous solution of saturated NH_4Cl (60 mL). The layers were separated and the aqueous layer was extracted with

ethyl acetate (3 × 60 mL). The combined organic layers were washed with saturated aqueous NH_4Cl and brine, dried over $MgSO_4$, filtered, and concentrated under reduced pressure. The crude residue was osmylated to destroy any residual alkene and to facilitate the purification. Osmylation condition: OsO_4 (4 w% in H_2O, 272 μL), 4-Methylmorpholine *N*-oxide (NMO) (568 mg, 2 equiv) and acetone/water (4:1, 16 mL) were added and stirred at room temperature for 3 h (monitored by TLC). After completion of the reaction, the catalyst was removed by filtration then purified by flash chromatography on silica gel with hexane/EtOAc (3:1). Product **1a-a** (720 mg) was obtained as a colorless liquid in 60% yield.

^{1}H NMR (400 MHz, $CDCl_3$) δ = 7.38–7.27 (m, 5H), 4.60–4.50 (m, 2H), 3.93 (ddd, *J* = 10.4, 8.6, 5.1 Hz, 2H), 3.21–3.13 (m, 2H), 2.73 (bs, 1H), 1.42–1.27 (m, 2H), 0.81 (td, *J* = 8.2, 5.0 Hz, 1H), 0.21 (q, *J* = 5.3 Hz, 1H). Spectral data matched those reported previously.[1]

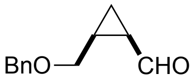

***cis*-2-(Benzyloxymethyl)cyclopropane-1-carbaldehyde (1a-b)** : Following a modified procedure by Shuto et al.,[2] to a flame-dried 100 mL of round bottom flask equipped with a stir bar, a solution of dimethyl sulfoxide (1.24 mL, 17.44 mmol) and anhydrous CH_2Cl_2 (16 mL) was added slowly into a stirred solution of oxalyl chloride (9.74 mL, 8.72 mmol) in CH_2Cl_2 (8 mL) at -78 °C over 30 min via syringe pump under nitrogen. To the resulting mixture, a solution of *cis*-2-(benzyloxymethyl)cyclopropylmethanol **1a-a** (828 mg, 4.36 mmol) in CH_2Cl_2 (8 mL) was added dropwise via syringe. The resulting mixture was stirred at the same temperature for 2 h, and then triethylamine (4.86 mL, 34.88 mmol) was added dropwise via syringe. After the resulting mixture was stirred at the same temperature for a further 30 min, aqueous saturated NH_4Cl (20 mL) and then CH_2Cl_2 (30 mL) were added to the mixture, and the aqueous and organic layers were separated. The organic layer was washed with brine and dried over Na_2SO_4. After concentration, the material was purified by flash chromatography on silica gel with hexane/EtOAc (4:1). Product **1a-b** (764 mg) was obtained as a colorless liquid in 92% yield.

^{1}H NMR (600 MHz, CDCl$_3$) δ = 9.47 (d, *J* = 4.6 Hz, 1H), 7.36–7.27 (m, 5H), 4.50–4.44 (m, 2H), 3.82 (dd, *J* = 10.5, 5.7 Hz, 1H), 3.43 (dd, *J* = 10.5, 8.6 Hz, 1H), 2.04 (dddd, *J* = 8.7, 7.8, 5.5, 4.6 Hz, 1H), 1.85 (qdd, *J* = 8.5, 6.9, 5.7 Hz, 1H), 1.33 (dt, *J* = 6.9, 5.2 Hz, 1H), 1.25 (td, *J* = 8.0, 4.9 Hz, 1H);
^{13}C NMR (150 MHz, CDCl$_3$) δ = 200.61, 138.05, 128.58 (2C), 127.96 (2C), 127.91, 73.13, 68.01, 26.99, 23.83, 12.56;
IR (film) 3030, 2925, 2858, 1703, 1454, 1378, 1089, 741, 699 cm^{-1};
HRMS (ESI) calcd for $C_{12}H_{14}O_2Na$ $[M+Na]^+$ *m/z* = 213.0892; found 213.0889.

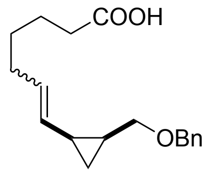

7-(*cis*-2-(Benzyloxymethyl)cyclopropyl))hept-6-enoic acid (1a-c): To a flame-dried 150 mL of round bottom flask equipped with a stir bar, (5-carboxypentyl)triphenylphosphonium bromide[3] (2.2 g, 4.8 mmol) was added and suspended into a stirred THF (25 mL) solution at -20 °C. Sodium bis(trimethylsilyl)amide solution (NaHMDS) (4.8 mL, 9.6 mmol, 2.0 M solution in THF) was added dropwise via syringe into the suspension and further stirred for 20 min under nitrogen. The reaction mixture was then cooled to -78 °C and *cis*-2-(benzyloxymethyl)cyclopropane-1-carbaldehyde **1a-b** (761 mg, 4.0 mmol) was added dropwise via syringe. After 32 h, the solvent was removed in *vacuo*. Water (60 ml) was added to the residue and extracted with diethyl ether (3 × 20 mL). The diethyl ether layers were discarded while the water layer was acidified to pH= 2 using hydrochloric acid (1 M). The acidified aqueous layer was further extracted with ethyl acetate (3 × 20 mL). The organic layers were combined and dried over Na_2SO_4. After concentration, the material was purified via flash chromatography on silica gel with hexane/EtOAc (2:1). Product **1a-c** (*Z*:*E* = 6.5:1, 764 mg) was obtained as a colorless liquid in 90% yield. Notably, product **1e-c** was obtained different ratios (*Z*:*E* = 6.5:1 to 11:1) when purified using Isolera™ Flash Systems silica gel chromatography with prepacked silica-gel cartridges (SNAP Ultra; Biotage) and a gradient elution hexane/EtOAc (80:20) to hexane/EtOAc (40:60).

(*Z*)-1a-c (major): **^{1}H NMR (600 MHz, CDCl$_3$)** δ = 7.35–7.32 (m, 4H), 7.30–7.26 (m, 1H), 5.41 (dtd, *J* = 10.8, 7.3, 1.2 Hz, 1H), 5.04 (ddt, *J* = 10.9, 9.3, 1.6 Hz, 1H), 4.55–4.50 (m, 2H), 3.51 (dd, *J* = 10.4, 6.8 Hz, 1H), 3.42 (dd, *J* = 10.4, 7.6 Hz, 1H), 2.36 (t, *J* = 7.5 Hz, 2H), 2.19 (qd, *J* = 7.4, 1.6 Hz, 2H), 1.74–1.64 (m, 3H), 1.48–1.43 (m, 2H), 1.35 (tddd, *J* = 8.5, 7.6, 6.8, 5.8 Hz, 1H), 1.02 (td, *J* = 8.3, 4.7 Hz, 1H), 0.32 (q, *J* = 5.4 Hz, 1H);

(*Z*)-1a-c (major): **^{13}C NMR (150 MHz, CDCl$_3$)** δ = 178.24, 138.66, 130.68, 128.88, 128.47 (2C), 127.88 (2C), 127.67, 72.79, 70.58, 33.81, 29.13, 27.27, 24.45, 18.00, 14.28, 12.34;

1a-c: **IR (film)** 3065, 3029, 2930, 2858, 1727, 1454, 1074, 737, 698 cm^{-1};

1a-c: **HRMS (ESI)** calcd for $C_{18}H_{24}O_3Na$ $[M+Na]^+$ *m/z* = 311.1623; found 311.1619.

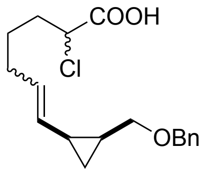

7-(*cis*-2-(Benzyloxymethyl)cyclopropyl))-2-chlorohept-6-enoic acid (1a-d): To a flame-dried 100 mL of round bottom flask equipped with a stir bar, *n*-BuLi solution (2.2 mL, 5.5 mmol, 2.5 M in hexane) was added dropwise via syringe into a stirred THF solution (5 mL) of diisopropylamine (0.91 mL, 6.5 mmol) at 0 °C under nitrogen. The mixture was stirred at that temperature for 40 min, then cooled to –20 °C. After addition of *N*,*N*'-dimethylpropyleneurea (DMPU) (1.25 mL) and 7-(*cis*-2-(benzyloxymethyl)cyclopropyl))hept-6-enoic acid **1a-c** (*Z*:*E* = 6.5:1, 721 mg, 2.5 mmol) in THF (5 mL), the resulting yellow solution was stirred at –20 °C for 2 h. The reaction mixture was then cooled to –78 °C and a THF solution (5 mL) of carbon tetrachloride (1.2 mL, 12.5 mmol) was added in a single aliquot resulting in a black mixture. After stirring at –78 °C for 2 h and then at 0 °C for 1 h, sodium chloride (1.5 g) and 1 M aqueous solution of hydrochloric acid (10 mL) were added. The mixture was extracted with methyl *tert*-butyl ether (MTBE) (3 × 10 mL) and the solvent was evaporated in *vacuo*. The residue was purified by flash chromatography on silica gel with hexane/EtOAc (1:2). Product **1a-d** (*Z*:*E* = 6.5:1, 508 mg) was obtained as a yellow liquid in 63% yield. Notably, product **1a-d** was obtained different ratios (*Z*:*E* = 6.5:1 to 11:1)

when purified by Isolera™ Flash Systems silica gel chromatography with performed on prepacked silica-gel cartridges (SNAP Ultra; Biotage) and a gradient elution hexane/EtOAc (80:20) to hexane/EtOAc (25:75).

(*Z*)-1a-d (major, two 1:1 inseparable diastereomers): **^{1}H NMR (600 MHz, $CDCl_3$)** δ = 7.34–7.33 (m, 8H), 7.30–7.26 (m, 2H), 5.40 (dt, *J* = 10.7, 7.0 Hz, 2H), 5.07–5.03 (m, 2H), 4.57–4.51 (m, 4H), 4.34 (ddd, *J* = 16.4, 7.6, 5.8 Hz, 2H), 3.56 (ddd, *J* = 13.6, 10.4, 6.4 Hz, 2H), 3.38 (ddd, *J* = 10.4, 8.1, 3.7 Hz, 2H), 2.29–2.17 (m, 4H), 2.13–1.97 (m, 4H), 1.73–1.54 (m, 6H), 1.41–1.34 (m, 2H), 1.02 (tdd, *J* = 8.4, 4.8, 1.4 Hz, 2H), 0.33–0.30 (m, 2H);

(*Z*)-1a-d (major, two 1:1 inseparable diastereomers): **^{13}C NMR (150 MHz, $CDCl_3$)** δ = 172.80, 172.72, 138.34, 138.26, 129.97, 129.92, 129.57, 129.54, 128.53 (2C), 128.52 (2C), 128.01 (2C), 127.99 (2C), 127.83, 127.80, 72.86, 72.83, 70.51, 70.47, 57.33, 57.24, 34.37, 34.15, 26.59, 26.56, 25.94, 25.70, 18.05 (2C), 14.47, 14.40, 12.27, 12.20;

1a-d: **IR (film)** 3066, 3012, 2927, 2859, 1723, 1454, 1251, 1175, 1071, 739, 698 cm^{-1};

1a-d: **HRMS (ESI)** calcd for $C_{18}H_{23}O_3ClNa$ $[M+Na]^+$ *m/z* = 345.1233; found 345.1235.

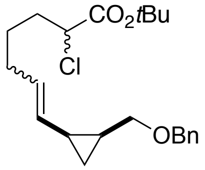

***tert*-Butyl 7-(*cis*-2-(benzyloxymethyl)cyclopropyl))-2-chlorohept-6-enoate (1a)** : To an oven-dried 100 mL of round bottom flask equipped with a stir bar, 7-(*cis*-2-(benzyloxymethyl)cyclopropyl))-2-chlorohept-6-enoic acid **1a-d** (484 mg, 1.5 mmol) was added into a stirred solution of *N*-(3-dimethylaminopropyl)-*N'*-ethylcarbodiimide hydrochloride (EDC) (345 mg, 1.8 mmol) in CH_2Cl_2 (5 mL) at room temperature. Then a mixed solution of the *tert*-butanol (0.15 mL, 1.5 mmol) and 4-dimethylaminopyridine (DMAP) (11 mg, 0.09 mmol) in CH_2Cl_2 (2 mL) was added dropwise via syringe. After the addition was complete, the reaction mixture was maintained for 3 h at the same temperature then diluted with CH_2Cl_2 (10 mL). The resulting mixture solution was transferred to a separatory funnel and washed with water and brine, then dried over Na_2SO_4. After concentration, the material was purified by flash chromatography on silica gel with hexane/EtOAc (20:1). Product **1a** (*Z*:*E* = 6.5:1, 307 mg) was obtained as a

colorless liquid in 54% yield. Notably, product **1a** (*Z*:*E* = 11:1, 102 mg) was obtained as a colorless liquid when purified by Isolera™ Flash Systems silica gel chromatography with prepacked silica-gel cartridges (SNAP Ultra; Biotage) and a gradient elution 100% hexane to hexane/EtOAc (95:5).

(*cis*, *Z*)-1a (major, two 1:1 inseparable diastereomers): **^{1}H NMR (600 MHz, $CDCl_3$)** δ = 7.36–7.32 (m, 8H), 7.30–7.26 (m, 2H), 5.40 (dtd, *J* = 10.8, 7.3, 1.2 Hz, 2H), 5.07 (ddt, *J* = 10.8, 9.3, 1.6 Hz, 2H), 4.55–4.50 (m, 4H), 4.16 (ddd, *J* = 7.9, 6.1, 0.8 Hz, 2H), 3.50 (ddd, *J* = 10.4, 6.9, 1.8 Hz, 2H), 3.42 (dd, *J* = 10.4, 7.5 Hz, 2H), 2.26–2.15 (m, 4H), 2.04–1.98 (m, 2H), 1.94–1.88 (m, 2H), 1.72–1.67 (m, 2H), 1.59–1.50 (m, 4H), 1.48 (s, 18H), 1.39–1.33 (m, 2H), 1.02 (tdd, *J* = 8.4, 4.8, 1.0 Hz, 2H), 0.33 (q, *J* = 5.4 Hz, 2H);

(*cis*, *Z*)-1a (major, two 1:1 inseparable diastereomers): **^{13}C NMR (150 MHz, $CDCl_3$)** δ = 168.94 (2C), 138.70 (2C), 130.16 (2C), 129.32 (2C), 128.48 (4C), 127.86 (4C), 127.67 (2C), 82.59 (2C), 72.81 (2C), 70.53 (2C), 58.59 (2C), 34.64, 34.62, 28.01 (6C), 26.96, 26.93, 26.11, 26.10, 18.03, 18.03, 14.24 (2C), 12.39 (2C);

1a: **IR (film)** 3065, 2978, 2929, 2857, 1740, 1454, 1369, 1149, 1092, 845, 736, 698 cm^{-1};

1a: **HRMS (ESI)** calcd for $C_{22}H_{32}O_3Cl$ $[M+H]^+$ *m/z* = 379.2040; found 379.2037.

Synthesis of α-chloro esters 1b

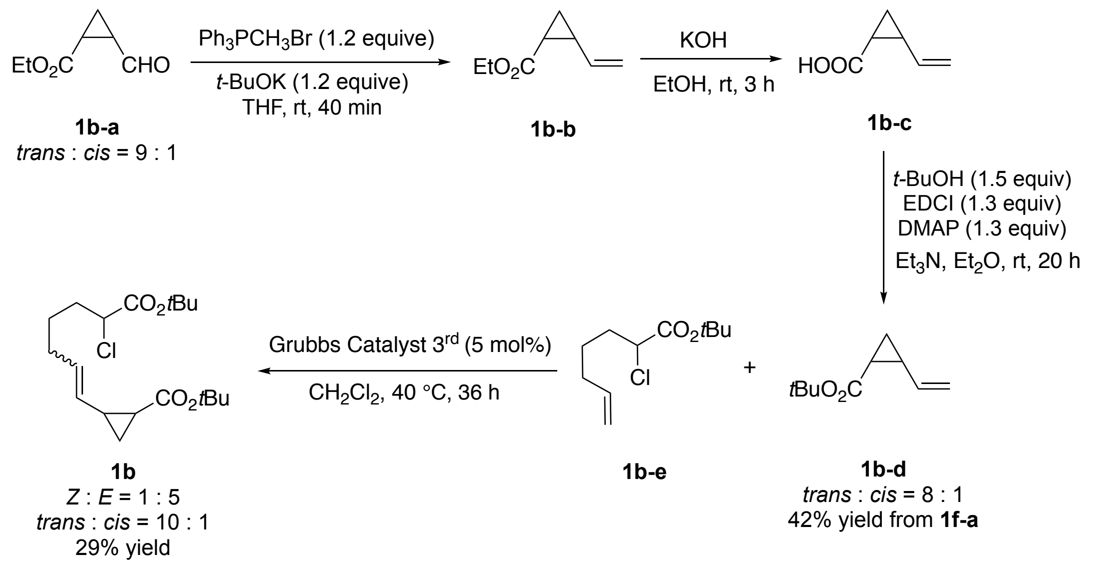

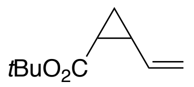

***tert*-Butyl 2-vinylcyclopropane-1-carboxylate (1b-d):** Following a modified procedure by DeLuca and Shibata et al,[4] to an oven-dried 500 mL of round bottom flask equipped with a stir bar, potassium *tert*-butoxide (2.3 g, 20 mmol) was added portion wise into a THF (100 mL) solution of methyltriphenylphosphonium bromide (7.14 g, 20 mmol), and the mixture was stirred at room temperature for 20 min. Then, a solution of ethyl 2-formylcyclopropane-1-carboxylate **1b-a** (2.6 g, 20 mmol, commercially available of Sigma-Aldrich) in THF (100 mL) was added dropwise via syringe. The resulting mixture was stirred at room temperature for 30 min. The mixture was quenched by 10 mL of water and was extracted with diethyl ether (3 × 100 mL). The combined organic layer was dried over $MgSO_4$. After concentration, the ethyl 2-vinylcyclopropane-1-carboxylate **1f-b** was obtained in quantitative yield and was used without further purification.

To the crude ethyl 2-vinylcyclopropane-1-carboxylate **1b-b**, a solution of potassium hydroxide (4.0 g) in ethyl alcohol (20 mL) was added dropwise. The resulting mixture was stirred at room temperature for 3 h (monitored by TLC) then the solvent was evaporated in *vacuo*. Water (100 mL) was added to the residue and extracted with CH_2Cl_2 (3 × 30 mL). The CH_2Cl_2 layers were discarded while the water layer was acidified to pH= 2 using hydrochloric acid (2 M). The acidified aqueous layer was further extracted with diethyl ether (3 × 100 mL). The organic layers were combined and dried over Na_2SO_4. After concentration, the crude 2-vinylcyclopropane-1-carboxylic acid **1b-c** was used without further purification.

A solution of *tert*-butanol (2.9 mL, 30 mmol) in diethyl ether (100 mL) was added to the crude 2-vinylcyclopropane-1-carboxylic acid **1b-c**. The reaction mixture was stirred at 0 °C and triethylamine (8.4 mL, 60 mmol) was added dropwise via syringe. Then *N*-(3-dimethylaminopropyl)-*N*′-ethylcarbodiimide hydrochloride (EDC) (5.0 g, 26 mmol) and 4-dimethylaminopyridine (DMAP) (3.2 g, 26 mmol) were added portion wise. After the addition was complete, the reaction mixture was stirred for 20 h at room temperature, then diluted with diethyl ether (100 mL). The resulting mixture solution was transferred to a separatory funnel washed with water and brine, then dried over Na_2SO_4. After concentration, the material was purified by flash chromatography on silica gel with pentane/Et_2O (30:1). Product **1b-d** (*trans*:*cis* = 8:1, 1.4 g) was obtained as a colorless liquid in 42% yield from **1b-a**.

***trans*-1b-d** (major): **^{1}H NMR (600 MHz, $CDCl_3$)** δ = 5.38 (ddd, *J* = 17.0, 10.3, 8.5 Hz, 1H), 5.15 (ddd, *J* = 17.0, 1.5, 0.7 Hz, 1H), 4.97 (dd, *J* = 10.3, 1.5 Hz, 1H), 1.95 (tdd, *J* = 8.6, 6.1, 3.9 Hz, 1H), 1.57–1.54 (m, 1H), 1.45 (s, 9H), 1.29 (ddd, *J* = 8.9, 5.2, 4.3 Hz, 1H), 0.90 (ddd, *J* = 8.4, 6.2, 4.3 Hz, 1H);

***trans*-1b-d** (major): **^{13}C NMR (150 MHz, $CDCl_3$)** δ = 172.72, 138.64, 114.54, 80.56, 28.30 (3C), 25.24, 23.07, 15.48;

1b-d: **IR (film)** 2979, 1718, 1390, 1367, 1287, 1211, 1147, 903, 844 cm^{-1};

1b-d: **HRMS (ESI)** calcd for $C_{10}H_{17}O_2$ $[M+H]^+$ *m/z* = 169.1228; found 169.1223.

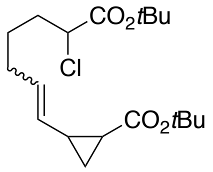

***tert*-Butyl 2-(7-(*tert*-butoxy)-6-chloro-7-oxohept-1-en-1-yl)cyclopropane-1-carboxylate (1b):** Following a modified procedure by Grubbs et al,[5] to a flame-dried 50 mL of round bottom flask equipped with a stir bar, *tert*-butyl 2-vinylcyclopropane-1-carboxylate **1b-d** (505 mg, 3 mmol) was added followed by the catalyst Grubbs catalyst 3rd generation (133 mg, 0.15 mmol) into a CH_2Cl_2 (7.5 mL) solution of *tert*-butyl 2-chlorohept-6-enoate **1b-e** (1.31 g, 6 mmol). After the addition was complete, the reaction mixture was stirred with a condenser at 40 °C under argon for 36 h. The resulting mixture was filtered through a short pad of silica gel. The solvent was removed in *vacuo*, and the residue was purified by Isolera™ Flash Systems silica gel chromatography with prepacked silica-gel cartridges (SNAP Ultra; Biotage) and a gradient elution 100% pentane to pentane/Et_2O (95:5). Product **1b** (*trans*:*cis* = 10:1, *Z*:*E* = 1:5, 312 mg) was obtained as a colorless liquid in 29% yield and recovered **1b-e (**570 mg). Notably, product **1b** was obtained different ratios (*trans*:*cis* = 8:1 to 10:1, *Z*:*E* = 1:4 to 1:5) when purified by Isolera™ Flash Systems silica gel chromatography with prepacked silica-gel cartridges (SNAP Ultra; Biotage) and a gradient elution 100% hexane to hexane/EtOAc (90:10).

(*trans*, *E*)-1b (major): **^{1}H NMR (600 MHz, $CDCl_3$)** δ = 5.55 (dt, *J* = 15.4, 6.8 Hz, 1H), 5.03 (ddt, *J* = 15.3, 8.4, 1.5 Hz, 1H), 4.14 (dd, *J* = 7.9, 6.0 Hz, 1H), 2.07–1.99 (m, 2H), 1.99–1.94 (m, 1H), 1.91–1.84 (m, 2H), 1.56–1.47 (m, 3H), 1.49 (s, 9H), 1.44 (s, 9H),

1.25 (dt, J = 9.1, 4.7 Hz, 1H), 0.84 (ddd, J = 8.4, 6.2, 4.2 Hz, 1H);

(***trans*, *E*)-1b** (major): ^{13}C NMR (150 MHz, $CDCl_3$) δ = 172.90, 168.87, 131.08, 129.89, 82.60, 80.44, 58.51, 34.45, 31.72, 28.29 (3C), 27.99 (3C), 25.81, 24.35, 22.91, 15.44;

1b: **IR (film)** 2978, 2933, 1717, 1456, 1367, 1211, 1146, 963, 843 cm^{-1};

1b: **HRMS (ESI)** calcd for $C_{19}H_{31}O_4ClNa$ $[M+Na]^+$ m/z = 381.1809; found 381.1813.

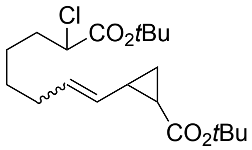

***tert*-Butyl 2-(7-(*tert*-butoxy)-6-chloro-7-oxohept-1-en-1-yl)cyclopropane-1-carboxylate (1c):** Following the compound **1b** procedure, to a flame-dried 50 mL of round bottom flask equipped with a stir bar, *tert*-butyl 2-vinylcyclopropane-1-carboxylate **1b-d** (337 mg, 2 mmol) was added followed by the catalyst Grubbs catalyst 3rd generation (88 mg, 0.1 mmol) into a CH_2Cl_2 (2 mL) solution of *tert*-butyl 2-chlorooct-7-enoate **1c-e** (930 mg, 4 mmol). After the addition was complete, the reaction mixture was stirred with a condenser at 40 °C under argon for 36 h. The resulting mixture was filtered through a short pad of silica gel. The solvent was removed in *vacuo*, and the residue was purified by Isolera™ Flash Systems silica gel chromatography with prepacked silica-gel cartridges (SNAP Ultra; Biotage) and a gradient elution 100% pentane to pentane/Et_2O (95:5). Product **1c** (*trans*:*cis* = 8:1, *Z*:*E* = 1:5, 209 mg) was obtained as a colorless liquid in 28% yield and recovered *tert*-butyl 2-chlorooct-7-enoate **1c-e (**394 mg).

(***trans*, *E*)-1c** (major): **^{1}H NMR (600 MHz, $CDCl_3$)** δ = 5.55 (dtd, J = 15.3, 6.8, 0.7 Hz, 1H), 5.00 (ddt, J = 15.3, 8.4, 1.5 Hz, 1H), 4.14 (dd, J = 7.9, 6.1 Hz, 1H), 2.03–1.92 (m, 3H), 1.92–1.82 (m, 2H), 1.52–1.34 (m, 5H), 1.48 (s, 9H), 1.44 (s, 9H), 1.24 (ddd, J = 9.1, 5.1, 4.2 Hz, 1H), 0.83 (ddd, J = 8.3, 6.2, 4.2 Hz, 1H).;

(***trans*, *E*)-1c** (major): **^{13}C NMR (150 MHz, $CDCl_3$)** δ = 172.96, 168.95, 130.63, 130.49, 82.58, 80.42, 58.59, 34.96, 32.20, 28.79, 28.32 (3C), 28.01 (3C), 25.58, 24.42, 22.95, 15.47;

1c: **IR (film)** 2978, 2931, 1720, 1454, 1368, 1209, 1148, 851 cm^{-1};

1c: **HRMS (ESI)** calcd for $C_{20}H_{33}O_4ClNa$ $[M+Na]^+$ m/z = 395.1965; found 395.1972.

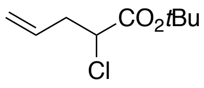

***tert*-Butyl 2-chloropent-4-enoate (1d):** To an oven-dried 100 mL of round bottom flask equipped with a stir bar, 2-chloropent-4-enoic acid[6] (672 mg, 5 mmol) was added into a stirred CH_2Cl_2 (15 mL) solution of *N*-(3-dimethylaminopropyl)-*N*′-ethylcarbodiimide hydrochloride (EDC) (1.15 g, 6 mmol) at room temperature. Then a mixed solution of the *tert*-butanol (0.58 mL, 6 mmol) and 4-dimethylaminopyridine (DMAP) (37 mg, 0.3 mmol) in CH_2Cl_2 (5 mL) was added dropwise via a syringe. After the addition was complete, the reaction mixture was maintained for 3 h at the room temperature, then diluted with CH_2Cl_2 (20 mL). The resulting mixture solution was transferred to a separatory funnel washed with water and brine, then dried over Na_2SO_4. After concentration, the material was purified by flash chromatography on silica gel with pentane/Et_2O (40:1). Product **1d** (553 mg) was obtained as a colorless liquid in 58% yield.

^{1}H NMR (600 MHz, $CDCl_3$) δ = 5.79 (ddt, *J* = 17.1, 10.2, 6.9 Hz, 1H), 5.20–5.15 (m, 2H), 4.19 (t, *J* = 6.9 Hz, 1H), 2.74 (dtt, *J* = 14.8, 6.8, 1.3 Hz, 1H), 2.64 (dtt, *J* = 14.4, 7.0, 1.3 Hz, 1H), 1.48 (s, 9H);

^{13}C NMR (150 MHz, $CDCl_3$) δ = 168.28, 132.49, 119.17, 82.84, 57.44, 39.38, 28.01 (3C);

IR (film) 2981, 2934, 1734, 1643, 1369, 1245, 1147, 924, 844 cm^{-1};

HRMS (ESI) calcd for $C_9H_{15}O_2ClNa$ $[M+Na]^+$ *m/z* = 213.0658; found 213.0656.

Synthesis of vinyl cyclopropane 5a to 5i

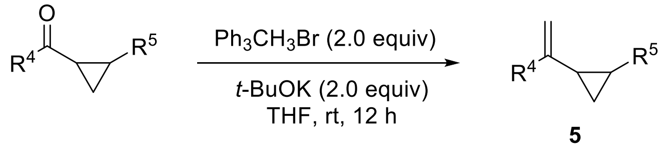

Following a modified procedure by Li and Lu et al,[7] to an oven-dried 100 mL of round bottom flask equipped with a stir bar, potassium *tert*-butoxide (1.12 g, 10 mmol, 2.0 equiv) was added portion wise into a THF (20 mL) solution of methyltriphenylphosphonium bromide (3.57 g, 10 mmol, 2.0 equiv), and the mixture was

stirred at room temperature for 30 min. Then, a solution of cyclopropyl ketone (5 mmol, 1.0 equiv) in THF (5 mL) was added dropwise via syringe. The resulting mixture was stirred at room temperature for 12 h (monitored by TLC). The mixture was quenched by 10 mL of water and was extracted with diethyl ether (3 × 20 mL). The combined organic layer was dried over $MgSO_4$. After concentration, the residue was purified by flash chromatography on silica gel with pentane/Et_2O to afford the desired product.

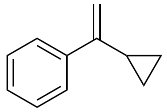

(1-cyclopropylvinyl)benzene (5a): Compound **5a** was synthesized following the above general procedure, using cyclopropyl(phenyl)methanone (731 mg, 5 mmol), potassium *tert*-butoxide (1.12 g, 10 mmol) and methyltriphenylphosphonium bromide (3.57 g, 10 mmol). The product **5a** was obtained as a colorless liquid (541 mg, 75% yield) after purified by flash chromatography on silica gel with Pentane/Et_2O (50:1).

^{1}H NMR (400 MHz, $CDCl_3$) δ = 7.60–7.58 (m, 2H), 7.36–7.25 (m, 3H), 5.27 (s, 1H), 4.93 (s, 1H), 1.69–1.62 (m, 1H), 0.86–0.81 (m, 2H), 0.61–0.57 (m, 2H). Spectral data matched those reported previously.[7]

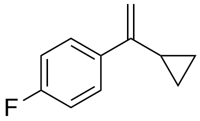

1-(1-cyclopropylvinyl)-4-fluorobenzene (5b): Compound **5b** was synthesized following the above general procedure, using cyclopropyl(4-fluorophenyl)methanone (821 mg, 5 mmol), potassium *tert*-butoxide (1.12 g, 10 mmol) and methyltriphenylphosphonium bromide (3.57 g, 10 mmol). The product **5b** was obtained as a colorless liquid (641 mg, 79% yield) after purified by flash chromatography on silica gel with Pentane/Et_2O (50:1).

^{1}H NMR (400 MHz, $CDCl_3$) δ = 7.57–7.53 (m, 2H), 7.03–6.99 (m, 2H), 5.21 (s, 1H), 4.91 (s, 1H), 1.64–1.57 (m, 1H), 0.86–0.81 (m, 2H), 0.60–0.56 (m, 2H). Spectral data matched those reported previously.[7]

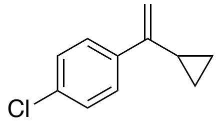

1-chloro-4-(1-cyclopropylvinyl)benzene (5c): Compound **5c** was synthesized following the above general procedure, using (4-chlorophenyl)(cyclopropyl)methanone (903 mg, 5 mmol), potassium *tert*-butoxide (1.12 g, 10 mmol) and methyltriphenylphosphonium bromide (3.57 g, 10 mmol). The product **5c** was obtained as a colorless liquid (643 mg, 72% yield) after purified by flash chromatography on silica gel with Pentane/Et_2O (50:1).
^{1}H NMR (400 MHz, $CDCl_3$) δ = 7.51 (d, *J* = 8.0 Hz, 2H), 7.29 (d, *J* = 8.0 Hz, 2H), 5.26 (s, 1H), 4.95 (s, 1H), 1.63–1.56 (m, 1H), 0.86–0.81 (m, 2H), 0.59–0.56 (m, 2H). Spectral data matched those reported previously.[7]

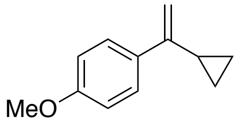

1-(1-cyclopropylvinyl)-4-methoxybenzene (5d): Compound **5d** was synthesized following the above general procedure, using cyclopropyl(4-methoxyphenyl)methanone (881 mg, 5 mmol), potassium *tert*-butoxide (1.12 g, 10 mmol) and methyltriphenylphosphonium bromide (3.57 g, 10 mmol). The product **5d** was obtained as a colorless liquid (665 mg, 76% yield) after purified by flash chromatography on silica gel with Pentane/Et_2O (20:1).
^{1}H NMR (400 MHz, $CDCl_3$) δ =7.54 (d, *J* = 8.0 Hz, 2H), 6.87 (d, *J* = 8.0 Hz, 2H), 5.19 (s, 1H), 4.85 (s, 1H), 3.82 (s, 3H), 1.65-1.59 (m, 1H), 0.84-0.79 (m, 2H), 0.59-0.55 (m, 2H). Spectral data matched those reported previously.[7]

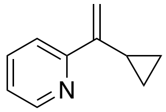

2-(1-cyclopropylvinyl)pyridine (5e): Compound **5e** was synthesized following the above general procedure, using cyclopropyl(pyridin-2-yl)methanone (736 mg, 5 mmol), potassium *tert*-butoxide (1.12 g, 10 mmol) and methyltriphenylphosphonium bromide (3.57 g, 10 mmol). The product **5e** was obtained as a colorless liquid (443 mg, 61% yield)

after purified by flash chromatography on silica gel with Pentane/Et_2O (2:1).

^{1}H NMR (400 MHz, $CDCl_3$) δ = 8.60 (d, *J* = 8.0 Hz, 1H), 7.70–7.63 (m, 2H), 7.19–7.16 (m, 1H), 5.89 (s, 1H), 5.12 (s, 1H), 1.90–1.82 (m, 1H), 0.90–0.86 (m, 2H), 0.62–0.58 (m, 2H). Spectral data matched those reported previously.[7]

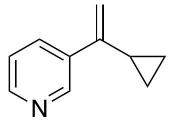

3-(1-cyclopropylvinyl)pyridine (5f): Compound **5f** was synthesized following the above general procedure, using cyclopropyl(pyridin-3-yl)methanone (736 mg, 5 mmol), potassium *tert*-butoxide (1.12 g, 10 mmol) and methyltriphenylphosphonium bromide (3.57 g, 10 mmol). The product **5f** was obtained as a colorless liquid (377 mg, 52% yield) after purified by flash chromatography on silica gel with Pentane/Et_2O (2:1).

^{1}H NMR (600 MHz, $CDCl_3$) δ = 8.83 (s, 1H), 8.51 (d, *J* = 8.0 Hz, 1H), 7.85 (d, *J* = 8.0 Hz, 1H), 7.27–7.24 (m, 1 H), 5.32 (s, 1H), 5.04 (s, 1H), 1.66–1.60 (m, 1H), 0.89–0.84 (m, 2H), 0.62–0.58 (m, 2H). Spectral data matched those reported previously.[7]

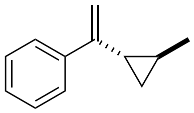

(1-(*trans*-2-methylcyclopropyl)vinyl)benzene (5g): Compound **5g** was synthesized following the above general procedure, using (*trans*-2-methylcyclopropyl)(phenyl)methanone[7] (801 mg, 5 mmol), potassium *tert*-butoxide (1.12 g, 10 mmol) and methyltriphenylphosphonium bromide (3.57 g, 10 mmol). The product **5g** was obtained as a colorless liquid (498 mg, 63% yield) after purified by flash chromatography on silica gel with Pentane/Et_2O (50:1).

^{1}H NMR (600 MHz, $CDCl_3$) δ = 7.60–7.51 (m, 2H), 7.35–7.24 (m, 3H), 5.22 (s, 1H), 4.87 (s, 1H), 1.38–1.30 (m, 1H), 1.21 (d, *J* = 5.8 Hz, 1H), 0.91–0.82 (m, 2H), 0.59–0.55 (m, 2H). Spectral data matched those reported previously.[7]

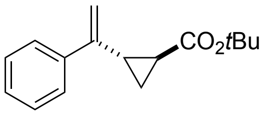

***tert*-butyl (*trans*)-2-(1-phenylvinyl)cyclopropane-1-carboxylate (5h):** Compound **5h** was synthesized following the above general procedure, using *tert*-butyl (*trans*)-2-benzoylcyclopropane-1-carboxylate[7] (1.23 g, 5 mmol), potassium *tert*-butoxide (1.12 g, 10 mmol) and methyltriphenylphosphonium bromide (3.57 g, 10 mmol). The product **5h** was obtained as a colorless liquid (916 mg, 75% yield) after purified by flash chromatography on silica gel with Pentane/Et_2O (20:1).

^{1}H NMR (600 MHz, $CDCl_3$) δ = 7.53–7.50 (m, 2H), 7.36–7.26 (m, 3H), 5.36 (s, 1H), 5.00 (s, 1H), 2.25–2.20 (m, 1H), 1.71–1.66 (m, 1H), 1.48 (s, 9H), 1.43–1.38 (m, 1H), 1.16–1.11 (m, 1H). Spectral data matched those reported previously.[7]

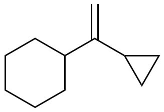

(1-cyclopropylvinyl)cyclohexane (5i): Compound **5i** was synthesized following the above general procedure, using cyclohexyl(cyclopropyl)methanone (152 mg, 1 mmol), potassium *tert*-butoxide (224 mg, 2 mmol) and methyltriphenylphosphonium bromide (714 mg, 2 mmol). The product **5i** was obtained as a colorless liquid (120 mg, 80% yield) after purified by flash chromatography on silica gel with Pentane/Et_2O (40:1).

^{1}H NMR (400 MHz, $CDCl_3$) δ = 4.59 (s, 1H), 4.50 (s, 1H), 1.95 (t, *J* = 8.0 Hz, 1H), 1.83–1.76 (m, 4H), 1.71–1.67 (m, 1H), 1.31–1.16 (m, 6H), 0.65–0.60 (m, 2H), 0.42–0.38 (m, 2H). Spectral data matched those reported previously.[7]

3. General Procedure of Iron-catalyzed Difunctionalization [8]

Procedure 1: Iron-catalyzed Enantioselective Intramolecular Difunctionalization

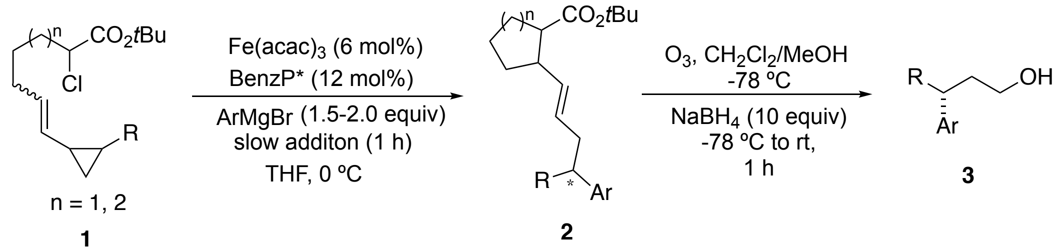

Part A: A flame-dried 5 mL microwave vial with a stir bar was brought into a argon-filled glovebox and the vial was charged with $Fe(acac)_3$ (4.2 mg, 6 mol%), (*R*,*R*)-BenzP* (6.8 mg, 12 mol%), and *tert*-butyl 2-chloroalkanoate (0.2 mmol). The vial was sealed with a Teflon cap and was brought out of the glovebox and 0.4 mL of THF were added. The red solution was stirred at room temperature for 5 min. The reaction mixture was then cooled to 0 °C and a ArMgBr solution (0.25–1.0 M solution in THF, 1.5–2.0 equiv) was added slowly over 1 h using a syringe pump, over which time the heterogeneous solution turned from red to colorless to yellow, brown or dark red color (depending on ArMgBr and substrate). After the addition was complete, the reaction mixture was maintained at 0 °C for an additional 10 min. Then the resulting mixture was quenched with a 1.0 M aqueous solution (0.4 mL) of hydrochloric acid and extracted with ethyl acetate (3 × 2 mL). The organic layer was filtered through a plug of silica and concentrated in *vacuo*. The resulting residue was filtered through a short pad of silica gel with hexane/CH_2Cl_2. Unless noted, the crude product **2** was used without further purification.

Part B: To a flame-dried 5 mL microwave vial with a stir bar, was added a solution of crude **2** in 4 mL of CH_2Cl_2/MeOH (v:v = 1:1). The solution was cooled to - 78 °C and stirred for 10 min. Ozone was bubbled through the alkene solution until a blue color persisted for 5 min. Nitrogen was bubbled through the solution to remove the excess ozone for 10 min. To the reaction sodium borohydride (10 equiv) was added at - 78 °C before warming up to room temperature. The reaction mixture was stirred at room temperature for another 1 h. Next, 5 mL of deionized water was added and the resulting mixture was extracted with CH_2Cl_2 (3 × 5 mL). The combined organic layers were dried

over $NaSO_4$, filtered and concentrated. The residue was purified by Isolera™ Flash Systems silica gel chromatography with prepacked silica-gel cartridges (SNAP Ultra; Biotage) and a gradient elution of pentane/Et_2O.

Procedure 2: Iron-catalyzed Intermolecular Difunctionalization

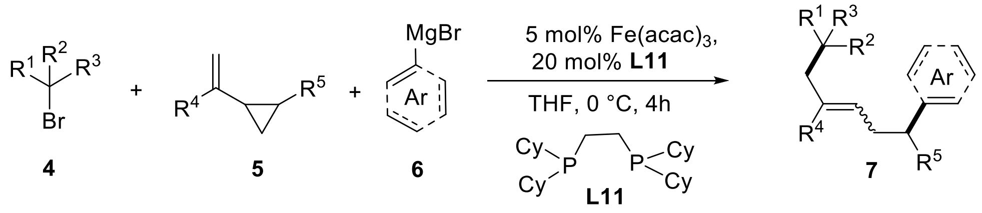

A flame-dried 5 mL microwave vial with a stir bar was brought into a argon-filled glovebox and the vial was charged with $Fe(acac)_3$ (3.5 mg, 5 mol%), 1,2-bis(dicyclohexylphosphanyl)ethane **L11** (16.9 mg, 20 mol%), vinyl cyclopropane **5** (0.2 mmol), and alkyl bromide **4** (1.1 mmol, 5.5 equiv). The vial was sealed with a Teflon cap and was brought out of the glovebox and 0.2 mL of THF was added. The red solution was stirred at room temperature for 5 min. The reaction mixture was then cooled to 0 °C and a ArMgBr solution (0.3–1.0 M solution in THF, 8.0 equiv) was added slowly over 4 h using a syringe pump, over which time the heterogeneous solution turned from red to colorless to yellow, brown or grass green color (depending on ArMgBr and substrate). After the addition was complete, the reaction mixture was maintained at 0 °C for an additional 10 min. Then the resulting mixture was quenched with a 1.0 M aqueous solution (0.4 mL) of hydrochloric acid or saturated aqueous NH_4Cl (depending on product properties), then extracted with ethyl acetate (3 × 2 mL). The organic layer was filtered through a plug of silica and concentrated in *vacuo*. The resulting residue was purified by flash chromatography on silica gel with hexane/CH_2Cl_2.

Procedure 3: Synthesis of racemic α-aryl alcohols 3a to 3k

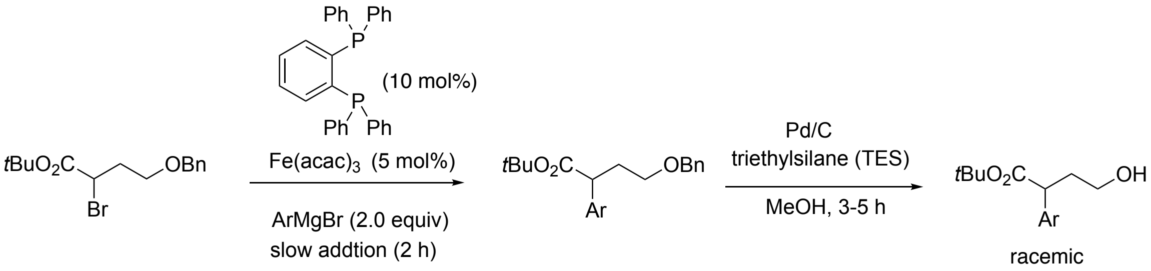

A flame-dried 5 mL microwave vial with a stir bar was brought into a argon-filled glovebox and the vial was charged with $Fe(acac)_3$ (3.6 mg, 5 mol%), 1,2-bis(diphenylphosphino)benzene (9.0 mg, 10 mol%), and *tert*-butyl 4-(benzyloxy)-2-bromobutanoate[8] (66 mg, 0.2 mmol). The vial was sealed with a Teflon cap and, outside the glovebox, 0.4 mL of THF were added. The red solution was stirred at room temperature for 5 min. The reaction mixture was then cooled to 0 °C and a ArMgBr solution (0.25–1.0 M solution in THF, 2.0 equiv) was added slowly over 2 h using a syringe pump. After the addition was complete, the reaction mixture was maintained at 0 °C for 10 min. The resulting mixture was quenched with a 1.0 M aqueous solution (0.4 mL) of hydrochloric acid and extracted with ethyl acetate (3 × 2 mL). The organic layer was filtered through a plug of silica and concentrated in *vacuo*, then the residue was purified by flash chromatography on silica gel with hexane/CH_2Cl_2.

Following a modified procedure by McMurray et al,[9] to a stirred solution of *tert*-butyl 2-aryl-4-(benzyloxy)butanoate and 10% Pd/C (20% by weight) in MeOH (0.4 mL) was added neat triethylsilane (TES) (1.0 equiv) dropwise under nitrogen. When the reaction was completed (monitored by TLC), the mixture was filtered through Celite and rinsed by ethyl acetate. The crude product was purified by flash chromatography on silica gel with hexane/EtOAc.

4. Screening of Reaction Condition for Iron-Catalyzed Difunctionalization

Iron-catalyzed Enantioselective Intramolecular Difunctionalization

Table S1. Screening of reaction conditions for iron-catalyzed enantioselective intramolecular difunctionalization

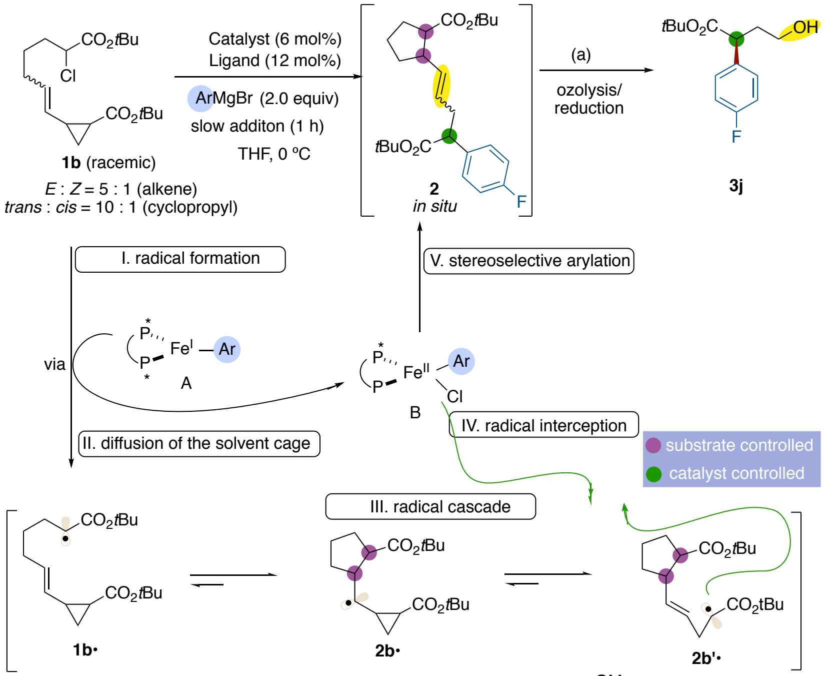

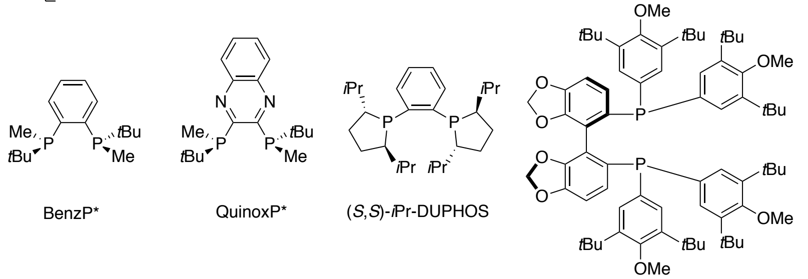

BenzP* QuinoxP* (*S*,*S*)-*i*Pr-DUPHOS (*R*)-DTMB-SEGPHOS

Entry	Metal	Ligand	Yield of **3j** (%)[c] [er][d]
1	$Pd(acac)_2$	BenzP*	<5
2	$Ni(acac)_2$	BenzP*	<5
3	$Cu(acac)_2$	BenzP*	<5
4	$Co(acac)_3$	BenzP*	15 [49:51]
5	$Fe(acac)_3$	BenzP*	84 [87:13]
6	$Fe(acac)_3$	QuinoxP*	<5
7	$Fe(acac)_3$	(*R*)-DTMB-SEGPHOS	<5
8	$Fe(acac)_3$	(*S*,*S*)-*i*Pr-DUPHOS	<5
9	$FeCl_2$	BenzP*	77 [87:13]

[a] O_3, CH_2Cl_2/MeOH -78 °C, 5 min then $NaBH_4$ (10 equiv) -78 °C to rt, 1 h. [b] Reactions were carry out on a 0.20 mmol scale. ArMgBr was added slowly, via syringe pump, over 1 h. [c] ^1H NMR yields were determined using CH_2Br_2 as internal standard. [d] The enantiomeric ratios (er) values were determined using chiral HPLC analysis.

Iron-catalyzed Intermolecular Difunctionalization

Table S2. Screening of ligands for iron-catalyzed intermolecular difunctionalization of vinyl cyclopropane

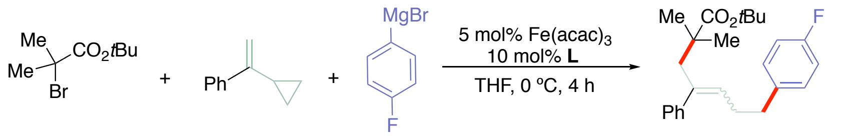

4a **5a** **6a** **7a**

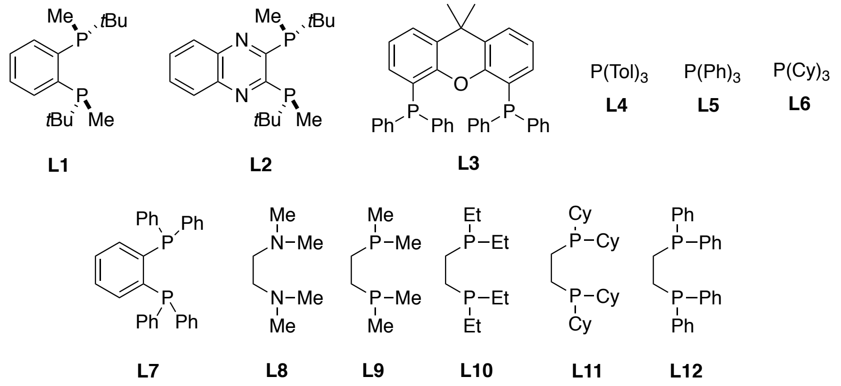

Entry	Ligand	Yield (%)[c]	*E*:*Z*[c]
1	**L1**	92	3.3:1
2	**L2**	70	3.3:1
3	**L3**	0	NA
4	**L4**	0	NA
5	**L5**	0	NA
6	**L6**	0	NA
7	**L7**	40	2.5:1
8[d]	**L7**	34	1.4:1
9[e]	**L7**	40	2.5:1
10	**L8**	55	3.8:1
11	**L9**	0	NA
12	**L10**	0	NA
13	**L11**	73	3.7:1
14	**L12**	0	NA
15[f]	none	0	NA
16[g]	none	0	NA

[a] Reactions were carry out on a 0.10 mmol scale, *tert*-butyl 2-bromo-2-methylpropanoate **4a** (5.5 equiv) and ArMgBr **6a** (8.0 equiv). [b] ArMgBr **6a** was added slowly, via syringe pump, over 4 h. [c] ^{1}H NMR yields and *E*/*Z* ratio were determined using CH_2Br_2 as internal standard. [d] At room temperature. [e] ArMgBr was added slowly for 6 h. [f] Without $Fe(acac)_3$. [g] Only $Fe(acac)_3$.

Table S3. Screening of catalyst loading and solvent for iron-catalyzed intermolecular difunctionalization of vinyl cyclopropane

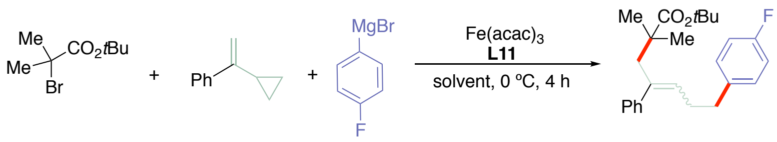

4a **5a** **6a** **7a**

Entry	$Fe(acac)_3$ (mol%)	**L11** (mol%)	Solvent	Yield (%)[c]	*E*:*Z*[c]
1	5	10	THF	73	3.7:1
2	5	15	THF	83	3.6:1
3	5	20	THF	89	3.8:1
4	5	100	THF	89	3.8:1
5	3	12	THF	82	3.5:1
6	5	10	toluene	47	3.1:1
7	5	10	CH_2Cl_2	34	3.5:1
8	5	10	Et_2O	63	3.7:1

[a] Reactions were carry out on a 0.10 mmol scale, *tert*-butyl 2-bromo-2-methylpropanoate **4a** (5.5 equiv) and ArMgBr **6a** (8.0 equiv). [b] ArMgBr **6a** was added slowly, via syringe pump, over 4 h. [c] ^1H NMR yields and *E*/*Z* ratio were determined using CH_2Br_2 as internal standard.

Table S4. Screening of substrates equiv and Grignard addtion for iron-catalyzed intermolecular difunctionalization of vinyl cyclopropane

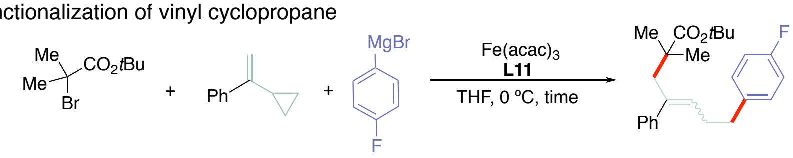

4a **5a** **6a** **7a**

Entry	**4a** (equiv)	**6a** (equiv)	$Fe(acac)_3$/**L11** (mol%)	Yield (%)[c]	*E*:*Z*[c]
1[d]	6.0	9.0	5/10	83	3.5:1
2[d]	6.0	9.0	5/20	79	3.4:1
3[d]	6.0	9.0	3/12	73	3.9:1
4	5.5	8.0	5/20	89	3.8:1
5[d]	5.0	7.3	5/20	80	3.7:1
6[d]	4.0	5.8	5/20	79	3.8:1
7[d]	3.0	4.4	5/20	63	3.7:1
8[d]	2.0	2.9	5/20	54	3.9:1
9[e]	1.0	1.5	5/20	30	3.9:1
10[e,f]	1.0	1.5	5/20	63	3.8:1
11[g]	5.5	8.0	5/20	49	3.4:1
12	5.5	7.0	5/20	71	3.6:1

[a] Reactions were carry out on a 0.10 mmol scale, *tert*-butyl 2-bromo-2-methylpropanoate **4a** (5.5 equiv) and ArMgBr **6a** (8.0 equiv). [b] ArMgBr **6a** was added slowly, via syringe pump, over 4 h. [c] ^1H NMR yields and *E*/*Z* ratio were determined using CH_2Br_2 as internal standard. [d] ArMgBr **6a** addition rate was same as entry 4. [e] ArMgBr **6a** addtion for 1h then stirred more 3 h. [f] vinyl cyclopropane **5a** (5.0 equiv). [g] ArMgBr **6a** addtion for 0.5 h then stirred more 3.5 h.

5. Study Diastereoselectivity and β-Hydride Elimination of Radical Cascade

Iron-catalyzed enantioselective radical-cascade/cross-coupling of compound **1a** (*Z*:*E* = 6.5:1) and **1a'** (*Z*:*E* = 11:1) under the general procedure 1 part A, led to the formation of **2a'** with identical diastereoselectivity (1.0 : 1.0 : 1.7 : 1.9 : 7.0 : 7.8 : 28.4 : 28.7 dr; **Figure S4** crude ^{19}F NMR, see below). It was determined by both crude NMR using dibromomethane as the internal standard (**Figure S2**, **S3** and **S4**). In Figure S1 was shown the comparison of product **2a'** *tert*-butyl group ratio of both reactions (**1a** and **1a'** as the substrate, respectively) crude ^{1}H NMR, the two images line up over each other exactly that supported the formation of **2a'** with identical diastereoselectivity. This result suggests that there is rapid equilibration of diastereomeric alkyl radical intermediates under Curtin-Hammett conditions. Thus we proposed the overall diastereoselectivity was substrate-dependent, but not related to the *Z*:*E* ratio of the substrate. Furthermore, the overall enatioselectivity of the terminating step is catalyst-controlled and does not depend on the *Z*:*E* ratio of the substrate, because both reactions gave the same er of product **3a'** (60:40 er) and **3b'** (racemic).

Scheme S1. Comparison of product enantioselectivity and diastereoselectivity on the *Z*:*E* ratio of the substrate.

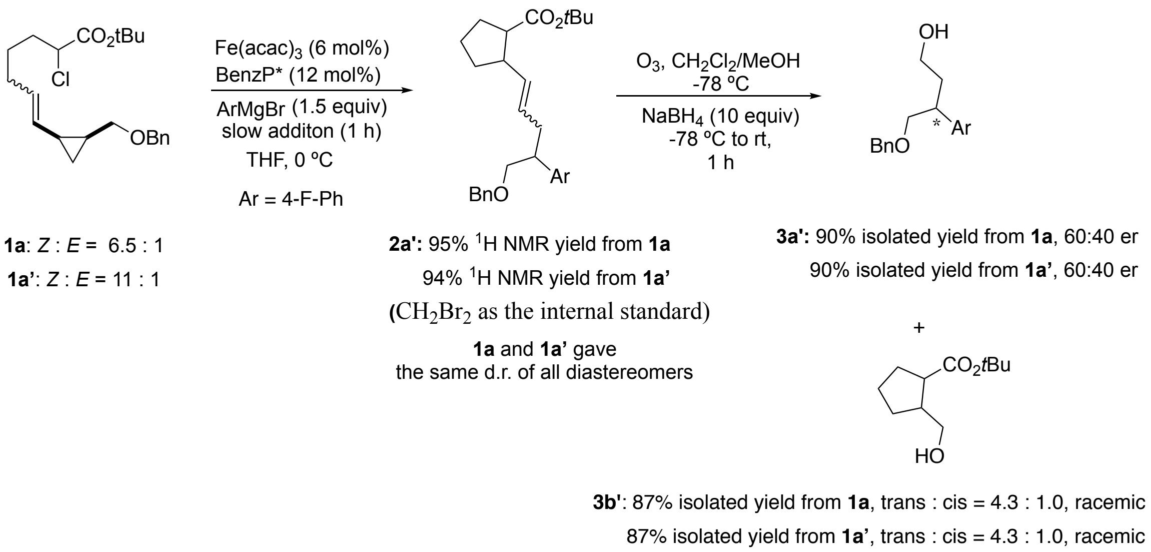

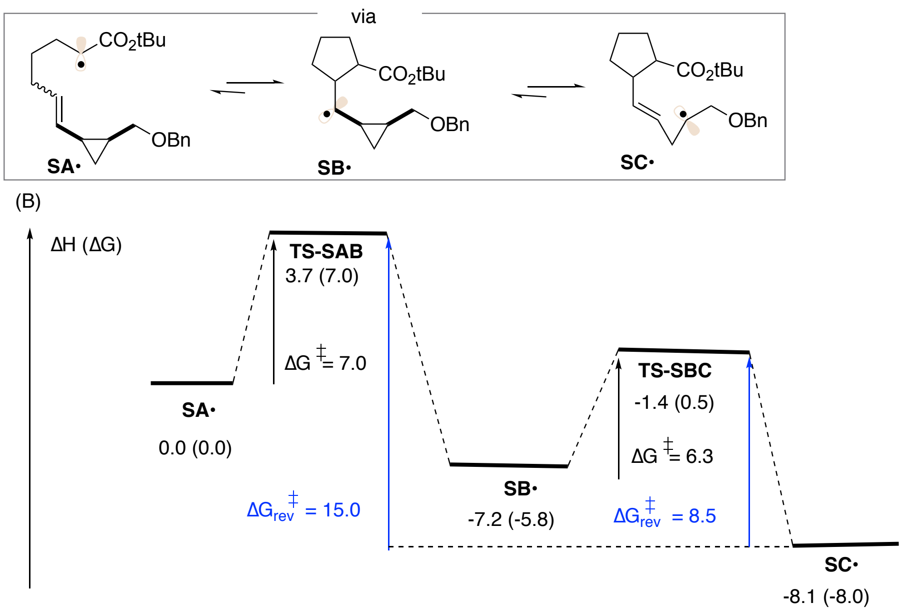

Figure S1. Trapping alkyl radicals from Fe-catalyzed radical cascade reactions. Energies were determined at the UPBEPBE/6-311+G(d,p)-SDD(Fe)-THF(SMD)//UB3LYP/6-31G(d) levels of theory.

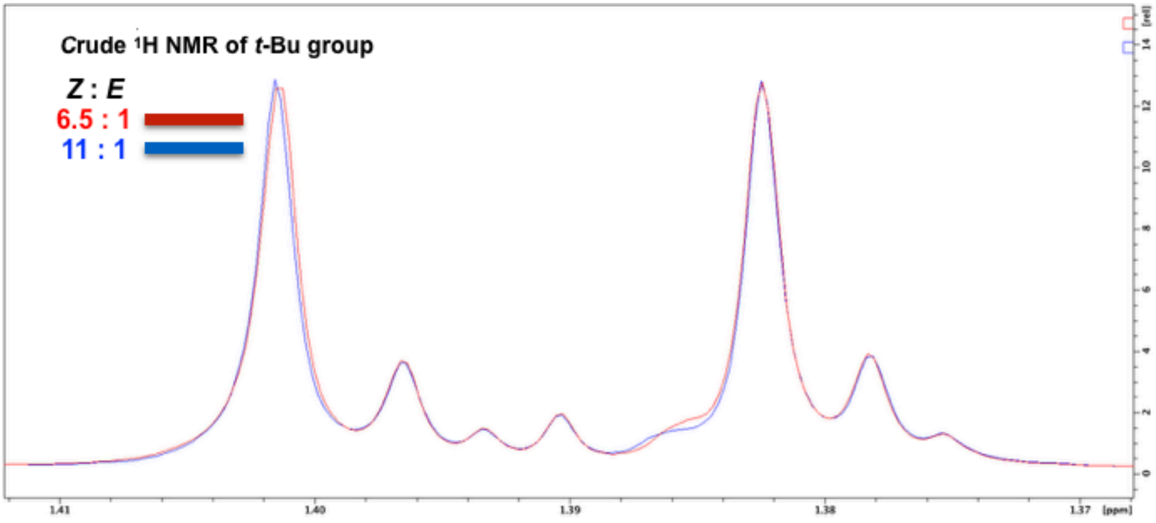

Figure S2. Comparison of *tert*-butyl group ratio of **1a** and **1a’** reactions crude ^{1}H NMR.

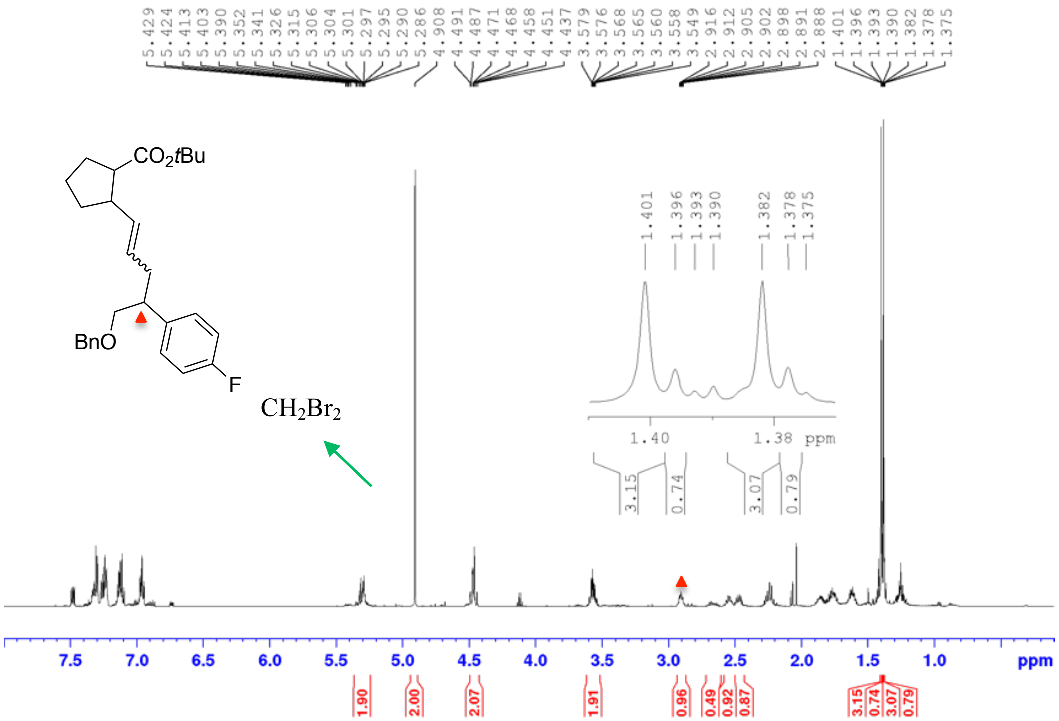

Figure S3. Compound **2a’** crude ^{1}H NMR ($CDCl_3$, 600 MHz).

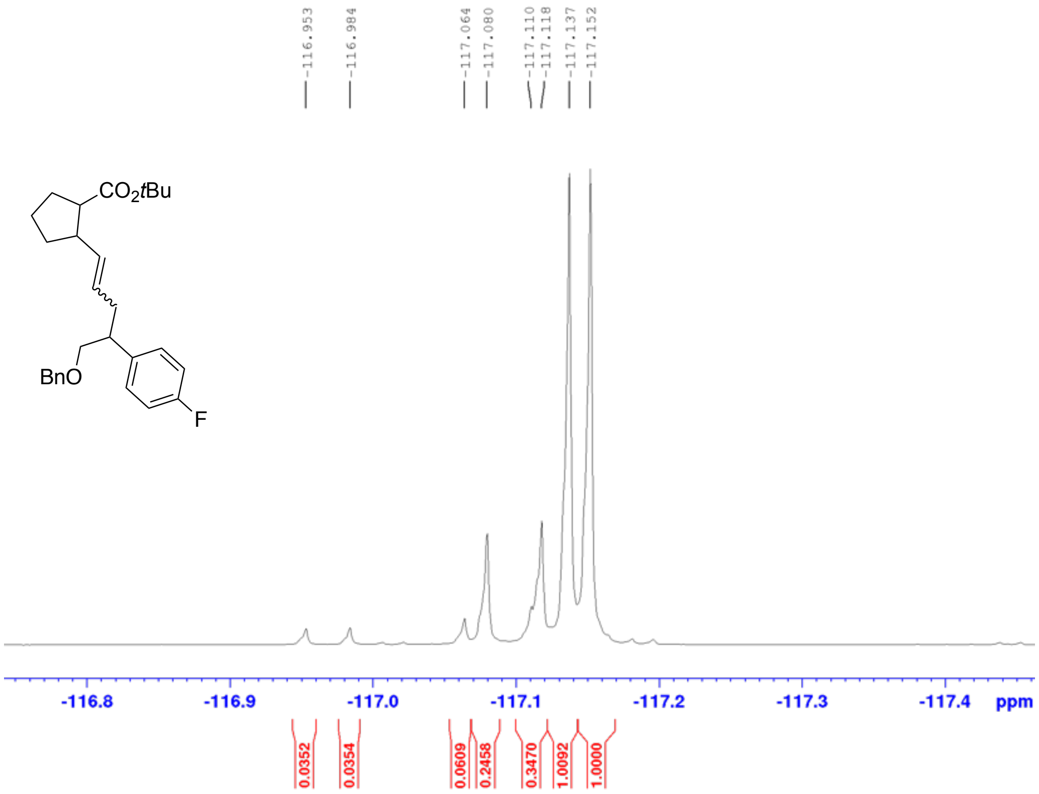

Figure S4. Compound **2a’** crude ^{19}F NMR ($CDCl_3$, 565 MHz).

Scheme S2. Lack of β-hydride elimination products in Fe-catalyzed direct arylation of α-chloro esters with pendant alkene.

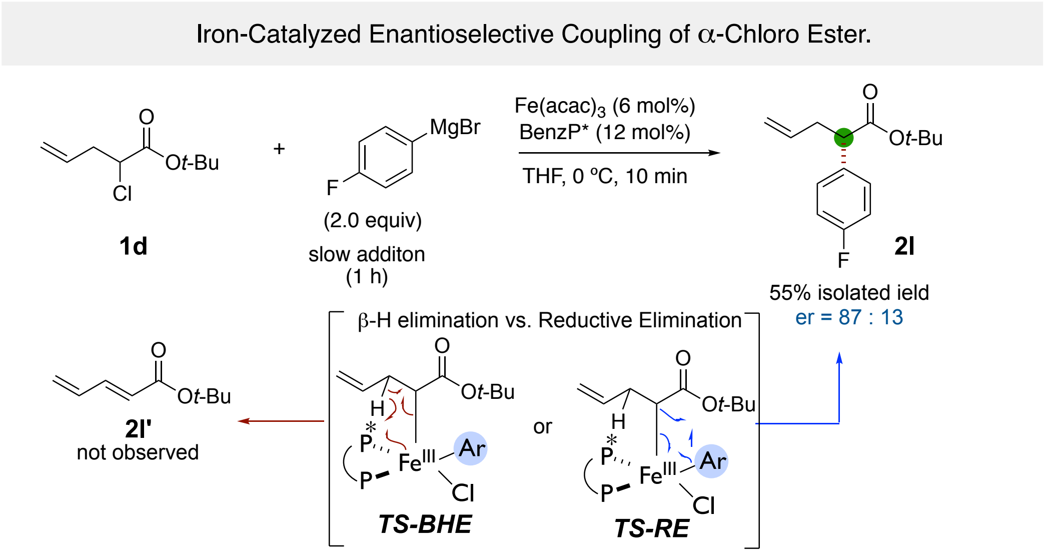

The results shown in Table S1 suggest that after trapping of the **2b'•** alkyl radical by the chiral Fe(II) species, the presumed Fe(III) intermediate will undergo reductive elimination rather than β-hydride elimination (BHE). This result is surprising since β-hydride elimination is known to be a fast process,[10] although the spin-state of the iron species is likely to play a role in slowing down BHE.[11] To validate this hypothesis we synthesized substrate **1d** and subjected to standard conditions. As shown in Scheme S2, the reaction forms **2l** as the major product in 55% yield and 87:13 er, presumably from ***TS-RE***. Further, analysis of the reaction mixture does not show the formation of conjugated diene **2l'** which would have resulted from β-hydride elimination (via ***TS-BHE***).

6. Comparison of Radical Cascade Arylation and Direct Arylation

Scheme S3. Iron-catalyzed intramolecular radical cascade arylation and direct arylation.

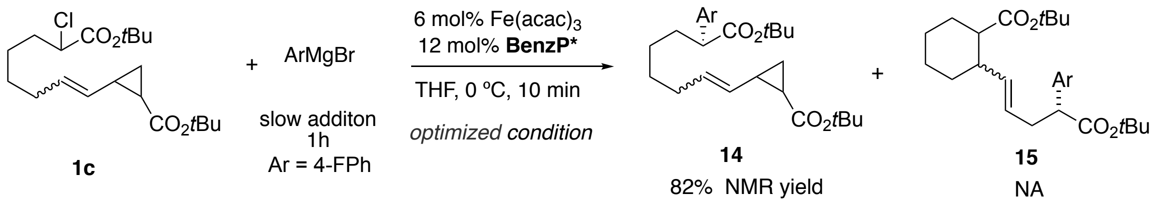

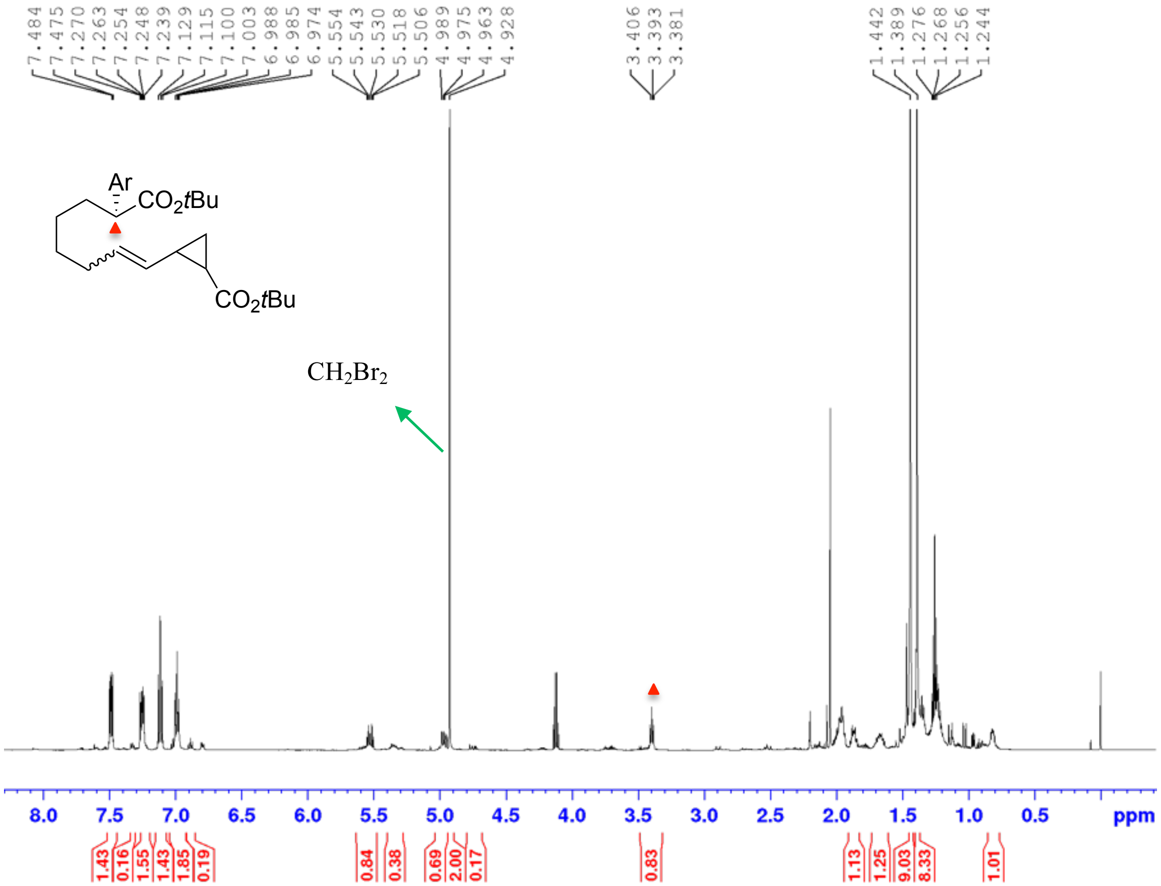

Figure S5. Compound **10** crude ^{1}H NMR ($CDCl_3$, 600 MHz).

Scheme S4. Iron-catalyzed intermolecular radical cascade arylation and direct arylation.

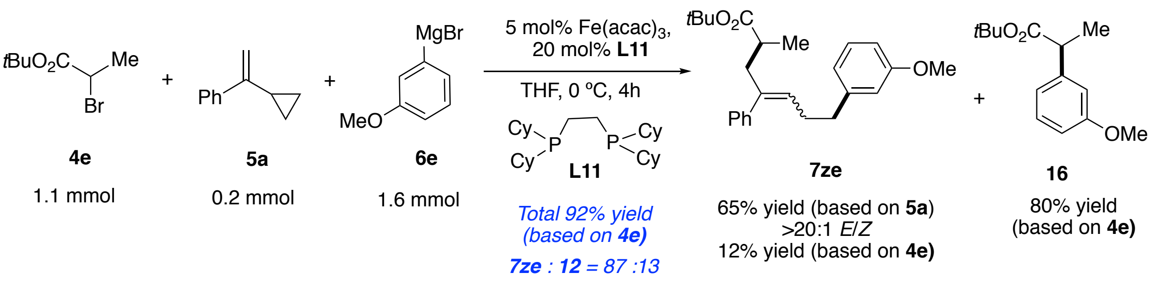

7. Structural Confirmation of Product 2a', 2a and 7

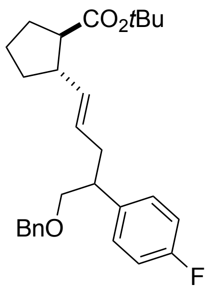

***tert*-butyl *trans*-2-((*E*)-4-argio-5-(benzyloxy)pent-1-en-1-yl)cyclopentane-1-carboxylate (2a')**: Compound **2a'** was synthesized following the general procedure 1 as described in Part A, using *tert*-butyl 7-(*cis*-2-(benzyloxymethyl)cyclopropyl))-2-chlorohept-6-enoate **1a** (75.8 mg, 0.2 mmol) and 4-fluorophenylmagnesium bromide (0.3 mL, 1.0 M solution in THF, 0.3 mmol). The crude product was purified by preparative HPLC (Waters XBridge, Prep Shield, 10 × 250 mm, 5 μm, flow-rate of 4 mL/min) with CH_3CN/H_2O (70:30) to give a 1:1 diastereomer mixture of the purity major product **2a'** (10.4 mg, *trans*-*E*) as a colorless liquid (not baseline separation of all diastereomers). ^1H NMR yield was 70% using dibromomethane as the internal standard. However, despite numerous attempts we were not able to determine the enantioselectivity of this diastereomeric mixture using chiral HPLC analysis.

^{1}H NMR (600 MHz, $CDCl_3$) δ = 7.33–7.30 (m, 4H), 7.29–7.26 (m, 2H), 7.25–7.23 (m, 4H), 7.15–7.11 (m, 4H), 6.98–6.94 (m, 4H), 5.35–5.27(m, 4H), 4.50–4.44 (m, 4H), 3.60–3.54 (m, 4H), 2.93–2.88 (m, 2H), 2.57–2.51 (m, 2H), 2.50–2.44 (m, 2H), 2.28–2.21 (m, 4H), 1.89–1.83 (m, 2H), 1.82–1.73 (m, 4H), 1.65–1.58 (m, 4H), 1.40 (s, 9H), 1.38 (s, 9H), 1.31–1.20 (m, 2H);

^{13}C NMR (150 MHz, $CDCl_3$) δ = 175.34 (2C), 161.64 (d, J = 243.6 Hz, 2C), 138.68–138.59 (m, 4C), 135.20, 135.15, 129.57–129.47 (m, 4C), 128.45 (4C), 127.63(6C), 127.12 (2C), 115.08 (d, J = 20.9 Hz, 2C), 115.03 (d, J = 21.0 Hz, 2C), 79.90 (2C), 74.06, 74.01, 73.18 (2C), 51.70, 51.62, 47.75, 47.62, 45.63 (2C), 36.05 , 36.02 , 33.37 , 33.31 , 30.03 (2C), 28.31 (3C), 28.28 (3C), 24.53 , 24.51;

^{19}F NMR (565 MHz, $CDCl_3$) δ = -117.22, -117.23;

^{1}H NMR (600 MHz, C_6D_6) δ = 7.23–7.16 (m, 8H), 7.10–7.08 (m, 2H), 6.93–6.90 (m, 4H), 6.86–6.82 (m, 4H), 5.44–5.34 (m, 3H), 5.32 (dd, J = 15.2, 7.5 Hz, 1H), 4.32–4.27 (m, 4H), 3.44–3.38 (m, 4H), 2.86–2.81 (m, 2H), 2.77–2.70 (m, 2H), 2.57–2.50 (m, 2H), 2.32 (q, J = 8.8, 1H), 2.31 (q, J = 8.8, 1H), 2.29–2.21 (m, 2H), 1.93–1.86 (m, 2H), 1.80–1.65 (m, 4H), 1.55–1.47 (m, 2H), 1.44–1.36 (m, 2H), 1.39 (s, 9H), 1.37 (s, 9H), 1.20–1.09 (m, 2H);

IR (film) 3032, 2929, 2857, 1723, 1605, 1510, 1454, 1366, 1223, 1148, 1115, 969, 834, 735, 698 cm^{-1}.

HRMS (ESI) calcd for $C_{28}H_{35}O_3FNa$ $[M+Na]^+$ m/z = 461.2468; found 461.2462;

$[\alpha]_D^{23}$ +2.7 (c 0.30, $CHCl_3$).

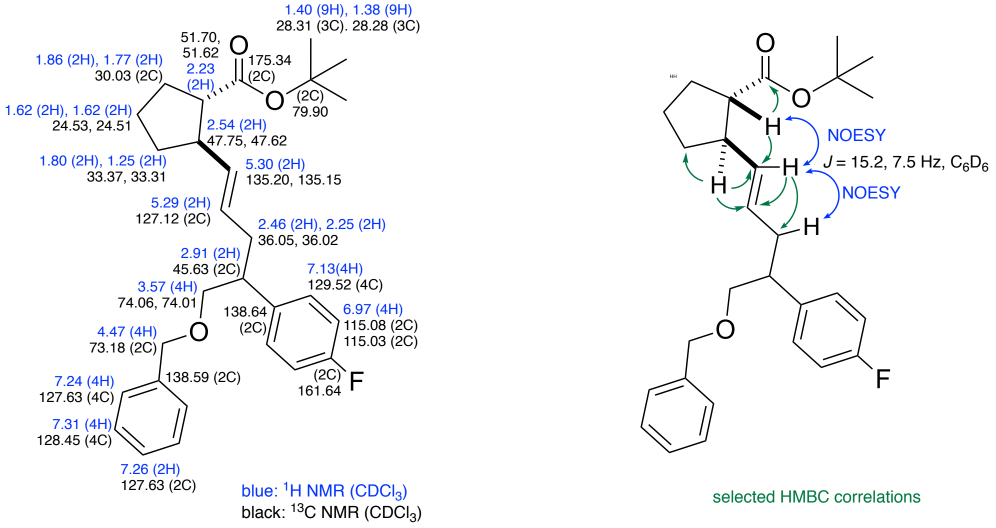

Figure S6. Confirmed the structure of ($trans$, E)- **2a'** (1:1, two diastereomers) based on ^{1}H NMR (C_6D_6, J coupling of alkene), NOSEY and selected HMBC correlations. NMR signal assignments based on HSQC, COSY and HMBC.

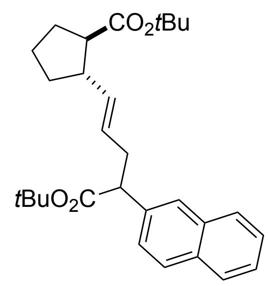

***tert*-butyl *trans*-2-((*E*)-5-(tert-butoxy)-4-(naphthalen-2-yl)-5-oxopent-1-en-1-yl)cyclopentane-1-carboxylate (2a)**: Compound **2a** was synthesized following the general procedure 1 as described in Part A, using *tert*-butyl 2-(7-(*tert*-butoxy)-6-chloro-7-oxohept-1-en-1-yl)cyclopropane-1-carboxylate **1b** (71.8 mg, 0.2 mmol) and 2-naphthylmagnesium bromide (0.8 mL, 0.5 M solution in THF, 0.4 mmol). The crude product was purified by Isolera™ Flash Systems silica gel chromatography with prepacked silica-gel cartridges (SNAP Ultra; Biotage) and a gradient elution pentane/Et_2O (98:2) to pentane/Et_2O (88:12) to separate the most of minor diastereomers, then a gradient elution toluene/CH_2Cl_2 (79:21) to toluene /CH_2Cl_2 (10:90) to give a 1:1 diastereomer mixture of the purity major product **2a** (4.3 mg, *trans-E*) as a colorless liquid (not baseline separation of all diastereomers). ^{1}H NMR yield was 67% using dibromomethane as the internal standard. However, despite numerous attempts we were not able to determine the enantioselectivity of this diastereomeric mixture using chiral HPLC analysis.

^{1}H NMR (600 MHz, $CDCl_3$) δ = 7.81–7.78 (m, 6H), 7.73 (d, *J* = 1.8 Hz, 1H), 7.71 (d, *J* = 1.8 Hz, 1H), 7.47–7.43 (m, 6H), 5.50–5.37 (m, 4H), 3.65–3.62 (m, 2H), 2.84–2.77 (m, 2H), 2.61–2.55 (m, 2H), 2.50–2.44 (m, 2H), 2.31–2.25 (m, 2H), 1.90–1.74 (m, 6H), 1.68–1.59 (m, 4H), 1.41 (s, 9H), 1.39 (s, 9H), 1.39 (s, 9H), 1.39 (s, 9H), 1.35–1.26 (m, 2H);

^{13}C NMR (150 MHz, $CDCl_3$) δ = 175.33 (2C), 172.83, 172.78, 137.06, 136.99, 135.45, 135.37, 133.61, 133.60, 132.73 (2C), 128.20, 128.16, 127.98, 127.97, 127.72 (2C), 126.83, 126.71 (2C), 126.56, 126.25, 126.21, 126.12, 126.10, 125.79, 125.78, 80.89 (2C), 79.97 (2C), 53.19, 53.13, 51.60, 51.56, 47.86, 47.61, 36.85, 36.60, 33.34, 33.24, 29.97, 29.92, 28.31 (3C), 28.30 (3C), 28.18 (3C), 28.17 (3C), 24.55, 24.52;

^{1}H NMR (600 MHz, C_6D_6) δ = 7.77 (dd, *J* = 6.8, 1.7 Hz, 2H), 7.63–7.58 (m, 6H), 7.55–7.52 (m, 2H), 7.25–7.18 (m, 4H), 5.63–5.56 (m, 2H), 5.53 (dd, *J* = 15.2, 7.6 Hz, 1H), 5.49 (dd, *J* = 15.4, 7.6 Hz, 1H), 3.78–3.75 (m, 2H), 3.05–2.99 (m, 2H), 2.79–2.73 (m,

2H), 2.59–2.51 (m, 2H), 2.39–2.33 (m, 2H), 1.96–1.87 (m, 2H), 1.80–1.65 (m, 4H), 1.55–1.45 (m, 2H), 1.44–1.29 (m, 2H), 1.41 (s, 9H), 1.40 (s, 9H), 1.34 (s, 9H), 1.33 (s, 9H), 1.24–1.11 (m, 2H);

IR (film) 3058, 2975, 2931, 2872, 1724, 1456, 1367, 1253, 1147, 967, 848, 746 cm^{-1};

HRMS (ESI) calcd for $C_{29}H_{39}O_4$ $[M+H]^+$ *m/z* = 451.2848; found 451.2853;

$[\alpha]_D^{23}$ +9.3 (c 0.19, $CHCl_3$).

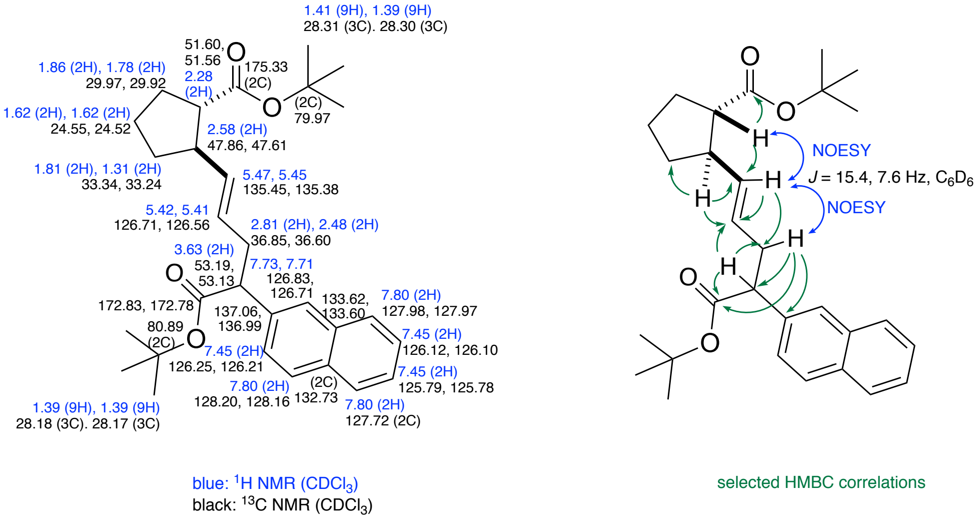

Figure S7. Confirmed the structure of (*trans*, *E*)- **2a** (1:1, two diastereomers) based on ^{1}H NMR (C_6D_6, *J* coupling of alkene), NOSEY and selected HMBC correlations. NMR signal assignments based on HSQC, COSY and HMBC.

Assignment of stereochemistry of product 7

The assignment of alkene configuration was corroborated by γ-substituent effects evident for ^{13}C NMR signals[12] of the allylic carbon-atom bound to the α-styryl atom. In all cases, the sterically compressed allylic carbon atom in the (*E*)-isomer exhibits δ_C lower than the corresponding atom in the (*Z*)-isomer. Diagnostic δ_C values are indicated in blue below.

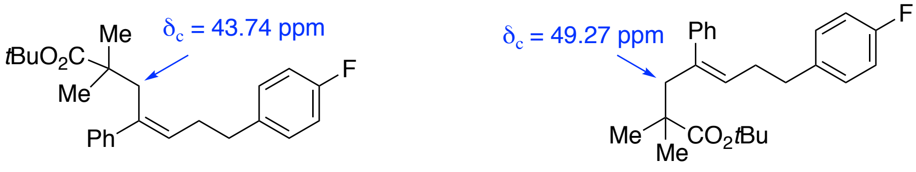

(*E*)-**7a** (*Z*)-**7a**

8. Product Characterization Data

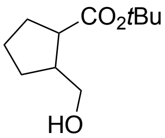

***tert*-butyl 2-(hydroxymethyl)cyclopentane-1-carboxylate (3b')**:

From **1a**: Compound **3b'** was synthesized following the general procedure 1 Part A and B, using *tert*-butyl 7-(*cis*-2-(benzyloxymethyl)cyclopropyl))-2-chlorohept-6-enoate **1a** (75.8 mg, 0.2 mmol) and 4-fluorophenylmagnesium bromide (0.3 mL, 1.0 M solution in THF, 0.3 mmol). The product **3b'** was obtained as a colorless liquid (34.8 mg, 87% yield, racemic, *trans*:*cis* = 4.3:1 from **1a**) after purified by Isolera™ Flash Systems silica gel chromatography with prepacked silica-gel cartridges (SNAP Ultra; Biotage) and a gradient elution pentane/Et_2O (90:10) to pentane/Et_2O (50:50).

From **1b**: Compound **3b'** was also obtained following the general procedure 1 Part A and B, using *tert*-butyl 2-(7-(*tert*-butoxy)-6-chloro-7-oxohept-1-en-1-yl)cyclopropane-1-carboxylate **1b** (71.8 mg, 0.2 mmol) and 2-naphthylmagnesium bromide (0.8 mL, 0.5 M solution in THF, 0.4 mmol). The product **3b'** was obtained as a colorless liquid (30.8 mg, 77% yield, racemic, *trans*:*cis* = 4:1 from **1b**) after purified by Isolera™ Flash Systems silica gel chromatography with prepacked silica-gel cartridges (SNAP Ultra; Biotage) and a gradient elution pentane/Et_2O (90:10) to pentane/Et_2O (50:50).

***trans*-3b'** (major): **^{1}H NMR (600 MHz, $CDCl_3$)** δ = 3.67 (dt, *J* = 10.6, 6.1 Hz, 1H), 3.55 (ddd, *J* = 10.6, 7.7, 4.8 Hz, 1H), 2.44 (q, *J* = 8.4 Hz, 1H), 2.30 (pd, *J* = 8.2, 5.5 Hz, 1H), 2.14 (dd, *J* = 6.3, 4.9 Hz, 1H), 1.94–1.82 (m, 3H), 1.72–1.58 (m, 2H), 1.46 (s, 9H), 1.31 (dq, *J* = 12.8, 8.1 Hz, 1H);

***trans*-3b'** (major): **^{13}C NMR (150 MHz, $CDCl_3$)** δ = 175.90, 80.75, 66.90, 49.31, 46.22, 30.31, 29.45, 28.23 (3C), 24.94;

***trans*-3b'** (major): **IR (film)** 3434, 2957, 2872, 1725, 1455, 1367, 1249, 1150, 1061, 846 cm^{-1};

***trans*-3b'** (major): **HRMS (ESI)** calcd for $C_{11}H_{20}O_3Na$ $[M+Na]^+$ *m/z* = 223.1310; found 223.1313;

***cis*-3b'** (minor): spectral data of matched those reported previously.[13]

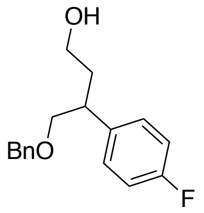

4-(benzyloxy)-3-(4-fluorophenyl)butan-1-ol (3a'): Compound **3a'** was synthesized following the general procedure 1 Part A and B, using *tert*-butyl 7-(*cis*-2-(benzyloxymethyl)cyclopropyl))-2-chlorohept-6-enoate **1a** (75.8 mg, 0.2 mmol) and 4-fluorophenylmagnesium bromide (0.3 mL, 1.0 M solution in THF, 0.3 mmol). The product **3a'** was obtained as a colorless liquid (49.3 mg, 90% yield, 60:40 er) after purified by Isolera™ Flash Systems silica gel chromatography with prepacked silica-gel cartridges (SNAP Ultra; Biotage) and a gradient elution pentane/Et_2O (90:10) to pentane/Et_2O (50:50). The er was determined by HPLC analysis on Daicel Chiralcel OJ-H column (Hexane/*i*-PrOH 90:10, 1.0 mL/min, 214 nm; t_r (minor) = 12.97 min, t_r (major)= 16.90 min).

^{1}H NMR (600 MHz, $CDCl_3$) δ = 7.35–7.32 (m, 2H), 7.30–7.26 (m, 3H), 7.19–7.15 (m, 2H), 7.01–6.97 (m, 2H), 4.53–4.49 (m, 2H), 3.65–3.62 (m, 1H), 3.59 (d, *J* = 6.5 Hz, 1H), 3.56–3.52 (m, 1H), 3.07 (dq, *J* = 8.4, 6.4 Hz, 1H), 2.07 (ddt, *J* = 14.0, 7.8, 6.1 Hz, 1H), 1.90–1.82 (m, 2H);

^{13}C NMR (150 MHz, $CDCl_3$) δ = 161.77 (d, *J* = 244.5 Hz, 1C), 138.55 (d, *J* = 3.0 Hz, 1C), 138.12, 129.32 (d, *J* = 7.8 Hz, 2C), 128.56 (2C), 127.85, 127.78 (2C), 115.44 (d, *J* = 20.9 Hz, 2C), 75.12, 73.38, 61.19, 42.70, 36.77;

^{19}F NMR (565 MHz, $CDCl_3$) δ = -116.54;

IR (film) 3388, 3033, 2924, 2855, 1604, 1509, 1221, 1096, 1052, 833, 697 cm^{-1};

HRMS (ESI) calcd for $C_{17}H_{20}O_2F$ $[M+H]^+$ *m/z* = 275.1447; found 275.1451;

$[\alpha]_D^{23}$ +3.4 (c 0.41, $CHCl_3$).

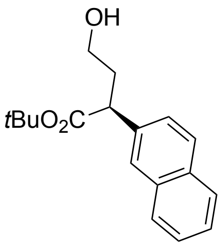

***tert*-butyl (*S*)-4-hydroxy-2-(naphthalen-2-yl)butanoate (3a):** Compound **3a** was synthesized following the general procedure 1 Part A and B, using *tert*-butyl 2-(7-(*tert*-butoxy)-6-chloro-7-oxohept-1-en-1-yl)cyclopropane-1-carboxylate **1b** (71.8 mg, 0.2 mmol) and 2-naphthylmagnesium bromide (0.8 mL, 0.5 M solution in THF, 0.4 mmol). The product **3a** was obtained as a white solid (45.7 mg, 80% yield, 82:18 er) after purified by Isolera™ Flash Systems silica gel chromatography with prepacked silica-gel cartridges (SNAP Ultra; Biotage) and a gradient elution pentane/Et_2O (90:10) to pentane/Et_2O (50:50). The er was determined by HPLC analysis on Daicel Chiralcel OJ-H column (Hexane/*i*-PrOH 90:10, 1.0 mL/min, 214 nm; t_r (minor) = 6.90 min, t_r (major)= 8.28 min).

mp: 60-61 °C;

^{1}H NMR (600 MHz, $CDCl_3$) δ = 7.83–7.80 (m, 3H), 7.74 (d, *J* = 1.3 Hz, 1H), 7.49–7.44 (m, 3H), 3.86 (t, *J* = 7.5 Hz, 1H), 3.71–3.67 (m, 1H), 3.63–3.58 (m, 1H), 2.43–2.37 (m, 1H), 2.06 (dddd, *J* = 14.1, 7.2, 6.1, 5.4 Hz, 1H), 1.55–1.53 (m, 1H), 1.39 (s, 9H);

^{13}C NMR (150 MHz, $CDCl_3$) δ = 173.45, 136.86, 133.61, 132.73, 128.41, 127.95, 127.75, 126.89, 126.23, 126.10, 125.91, 81.16, 60.88, 49.53, 36.22, 28.10 (3C);

IR (film) 3413, 2973, 2933, 2873, 1727, 1699, 1363, 1149, 1056, 1035, 1015, 818, 746 cm^{-1};

HRMS (ESI) calcd for $C_{18}H_{22}O_3Na$ $[M+Na]^+$ *m/z* =309.1467; found 301.1471;

$[\alpha]_D^{22}$ +28.6 (c 0.30, $CHCl_3$).

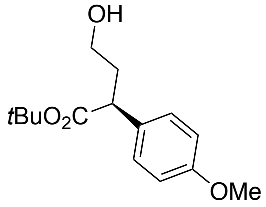

***tert*-butyl (*S*)-4-hydroxy-2-(4-methoxyphenyl)butanoate (3b):** Compound **3b** was synthesized following the general procedure 1 Part A and B, using *tert*-butyl 2-(7-(*tert*-butoxy)-6-chloro-7-oxohept-1-en-1-yl)cyclopropane-1-carboxylate **1b** (71.8 mg, 0.2 mmol) and 4-methoxyphenylmagnesium bromide (0.8 mL, 0.5 M solution in THF, 0.4 mmol). The product **3b** was obtained as a colorless liquid (38.8 mg, 73% yield, 85:15 er) after purified by Isolera™ Flash Systems silica gel chromatography with prepacked

silica-gel cartridges (SNAP Ultra; Biotage) and a gradient elution pentane/Et_2O (88:12) to pentane/Et_2O (25:75). The er was determined by HPLC analysis on Daicel Chiralcel OJ-H column (Hexane/*i*-PrOH 90:10, 1.0 mL/min, 214 nm; t_r (minor) = 6.52 min, t_r (major)= 7.22 min).

^{1}H NMR (600 MHz, $CDCl_3$) δ = 7.23–7.20 (m, 2H), 6.86–6.84 (m, 2H), 3.79 (s, 3H), 3.67–3.62 (m, 1H), 3.62 (t, *J* = 7.8 Hz, 1H), 3.60–3.55 (m, 1H), 2.27 (dddd, *J* = 13.9, 8.0, 7.3, 5.6 Hz, 1H), 1.94 (dddd, *J* = 12.7, 7.1, 6.2, 5.5 Hz, 1H), 1.49–1.48 (m, 1H), 1.39 (s, 9H);

^{13}C NMR (150 MHz, $CDCl_3$) δ = 173.75, 158.78, 131.42, 129.02 (2C), 114.09 (2C), 80.91, 60.92, 55.37, 48.58, 36.31, 28.09 (3C);

IR (film) 3422, 2977, 2934, 2839, 1708, 1512, 1366, 1247, 1145, 1033, 834 cm^{-1};

HRMS (ESI) calcd for $C_{15}H_{23}O_4$ $[M+H]^+$ *m/z* = 367.1596; found 267.1598;

$[\alpha]_D^{22}$ +19.4 (c 0.44, $CHCl_3$).

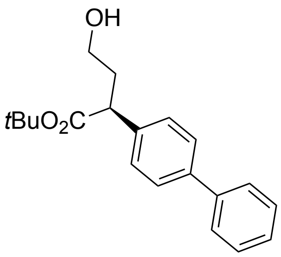

***tert*-butyl (*S*)-2-([1,1'-biphenyl]-4-yl)-4-hydroxybutanoate (3c):** Compound **3c** was synthesized following the general procedure 1 Part A and B, using *tert*-butyl 2-(7-(*tert*-butoxy)-6-chloro-7-oxohept-1-en-1-yl)cyclopropane-1-carboxylate **1b** (71.8 mg, 0.2 mmol) and 4-biphenylmagnesium bromide (0.8 mL, 0.5 M solution in THF, 0.4 mmol). The product **3c** was obtained as a white solid (50.5 mg, 81% yield, 82:18 er) after purified by Isolera™ Flash Systems silica gel chromatography which was performed on prepacked silica-gel cartridges (SNAP Ultra; Biotage) with gradient elution pentane/Et_2O (88:12) to pentane/Et_2O (50:50). The er was determined by HPLC analysis on Daicel Chiralcel OJ-H column (Hexane/*i*-PrOH 95:5, 0.7 mL/min, 214 nm; t_r (major) = 21.20 min, t_r (minor)= 23.63 min).

mp: 56-57 °C;

^{1}H NMR (600 MHz, $CDCl_3$) δ = 7.60–7.58 (m, 2H), 7.57–7.54 (m, 2H), 7.45–7.42 (m,

2H), 7.39–7.36 (m, 2H), 7.36–7.33 (m, 1H), 3.74 (t, *J* = 7.6 Hz, 1H), 3.69 (dt, *J* = 10.8, 5.9 Hz, 1H), 3.62 (ddd, *J* = 10.8, 7.2, 5.4 Hz, 1H), 2.35 (dddd, *J* = 13.8, 8.1, 7.2, 5.6 Hz, 1H), 2.01 (dddd, *J* = 14.0, 7.0, 6.2, 5.5 Hz, 1H), 1.63 (bs, 1H), 1.42 (s, 9H);

^{13}C NMR (150 MHz, $CDCl_3$) δ = 173.45, 140.86, 140.07, 138.41, 128.88 (2C), 128.43 (2C), 127.40 (2C), 127.38, 127.14 (2C), 81.13, 60.87, 49.07, 36.30, 28.10 (3C);

IR (film) 3425, 3030, 2977, 2931, 1724, 1486, 1368, 1146, 1048, 843, 759, 698 cm^{-1};

HRMS (ESI) calcd for $C_{20}H_{25}O_3$ $[M+H]^+$ *m/z* = 313.1804; found 313.1807;

$[\alpha]_D^{22}$ +16.6 (c 0.52, $CHCl_3$).

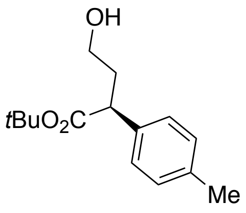

***tert*-butyl (*S*)-4-hydroxy-2-(*p*-tolyl)butanoate (3d):** Compound **3d** was synthesized following the general procedure 1 Part A and B, using *tert*-butyl 2-(7-(*tert*-butoxy)-6-chloro-7-oxohept-1-en-1-yl)cyclopropane-1-carboxylate **1b** (71.8 mg, 0.2 mmol) and *p*-tolylmagnesium bromide (0.4 mL, 1.0 M solution in THF, 0.4 mmol). The product **3d** was obtained as a colorless liquid (37.5 mg, 75% yield, 83:17 er) after purified by Isolera™ Flash Systems silica gel chromatography with prepacked silica-gel cartridges (SNAP Ultra; Biotage) and a gradient elution pentane/Et_2O (90:10) to pentane/Et_2O (55:45). The er was determined by HPLC analysis on Daicel Chiralcel OJ-H column (Hexane/*i*-PrOH 95:5, 0.7 mL/min, 214 nm; t_r (minor) = 8.45 min, t_r (major)= 10.47 min).

^{1}H NMR (600 MHz, $CDCl_3$) δ = 7.19–7.17 (m, 2H), 7.12 (d, *J* = 7.8 Hz, 2H), 3.67–3.62 (m, 1H), 3.64 (t, *J* = 7.5 Hz, 1H), 3.58 (ddt, *J* = 10.9, 7.1, 5.5 Hz, 1H), 2.33 (s, 3H), 2.28 (dddd, *J* = 13.8, 8.1, 7.2, 5.6 Hz, 1H), 1.97–1.92 (m, 1H), 1.50 (t, *J* = 5.5 Hz, 1H), 1.39 (s, 9H);

^{13}C NMR (150 MHz, $CDCl_3$) δ = 173.65, 136.79, 136.33, 129.41 (2C), 127.86 (2C), 80.91, 60.96, 49.05, 36.33, 28.09 (3C), 21.20;

IR (film) 3412, 2977, 2929, 2877, 1724, 1514, 1367, 1144, 1046, 844, 779 cm^{-1};

HRMS (ESI) calcd for $C_{15}H_{23}O_3$ $[M+H]^+$ *m/z* =251.1647; found 251.1646;

$[\alpha]_D^{22}$ +20.9 (c 0.63, $CHCl_3$).

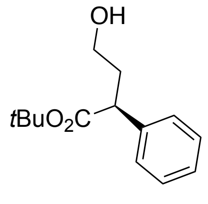

***tert*-butyl (*S*)-4-hydroxy-2-phenylbutanoate (3e):** Compound **3e** was synthesized following the general procedure 1 Part A and B, using *tert*-butyl 2-(7-(*tert*-butoxy)-6-chloro-7-oxohept-1-en-1-yl)cyclopropane-1-carboxylate **1b** (71.8 mg, 0.2 mmol) and phenylmagnesium bromide (0.4 mL, 1.0 M solution in THF, 0.4 mmol). The product **3e** was obtained as a colorless liquid (30.7 mg, 65% yield, 83:17 er) after purified by Isolera™ Flash Systems silica gel chromatography with prepacked silica-gel cartridges (SNAP Ultra; Biotage) and a gradient elution pentane/Et_2O (90:10) to pentane/Et_2O (55:45). The er was determined by HPLC analysis on Daicel Chiralcel OJ-H column (Hexane/*i*-PrOH 95:5, 0.7 mL/min, 214 nm; t_r (minor) = 11.13 min, t_r (major)= 11.82 min).

^{1}H NMR (600 MHz, $CDCl_3$) δ = 7.33–7.28 (m, 4H), 7.26–7.24 (m, 1H), 3.70–3.64 (m, 1H), 3.68 (t, *J* = 7.6 Hz, 1H), 3.58 (ddt, *J* = 10.8, 7.0, 5.4 Hz, 1H), 2.31 (dddd, *J* = 13.9, 8.2, 7.3, 5.6 Hz, 1H), 1.99–1.94 (m, 1H), 1.53 (t, *J* = 5.3 Hz, 1H), 1.39 (s, 9H);

^{13}C NMR (150 MHz, $CDCl_3$) δ = 173.48, 139.40, 128.71 (2C), 128.01 (2C), 127.21, 81.02, 60.90, 49.45, 36.27, 28.07 (3C);

IR (film) 3404, 2978, 2932, 2872, 1725, 1455, 1368, 1149, 1052, 699 cm^{-1};

HRMS (ESI) calcd for $C_{14}H_{20}O_3Na$ $[M+Na]^+$ *m/z* = 259.1310; found 259.1307;

$[\alpha]_D^{22}$ +20.6 (c 0.70, $CHCl_3$).

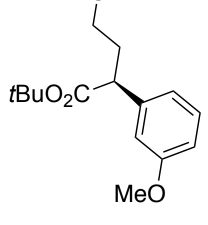

***tert*-butyl (*S*)-4-hydroxy-2-(3-methoxyphenyl)butanoate (3f):** Compound **3f** was synthesized following the general procedure 1 Part A and B, using *tert*-butyl 2-(7-(*tert*-butoxy)-6-chloro-7-oxohept-1-en-1-yl)cyclopropane-1-carboxylate **1b** (71.8 mg, 0.2

mmol) and 3-methoxyphenylmagnesium bromide (0.4 mL, 1.0 M solution in THF, 0.4 mmol). The product **3f** was obtained as a colorless liquid (41 mg, 77% yield, 82:18 er) after purified by Isolera™ Flash Systems silica gel chromatography with prepacked silica-gel cartridges (SNAP Ultra; Biotage) and a gradient elution pentane/Et_2O (88:12) to pentane/Et_2O (25:75). The er was determined by HPLC analysis on Daicel Chiralcel OJ-H column (Hexane/*i*-PrOH 99.5:0.5, 0.7 mL/min, 214 nm; t_r (major) = 31.49 min, t_r (minor)= 33.50 min).

^{1}H NMR (600 MHz, $CDCl_3$) δ = 7.23 (t, *J* = 7.9 Hz, 1H), 6.88 (dt, *J* = 7.7, 1.3 Hz, 1H), 6.85 (t, *J* = 2.1 Hz, 1H), 6.79 (ddd, *J* = 8.3, 2.6, 0.9 Hz, 1H), 3.80 (s, 3H), 3.68–3.64 (m, 1H), 3.65 (t, *J* = 7.6 Hz, 1H), 3.58 (ddd, *J* = 10.8, 7.2, 5.4 Hz, 1H), 2.29 (dddd, *J* = 13.8, 8.2, 7.3, 5.6 Hz, 1H), 1.98–1.93 (m, 1H), 1.50 (bs, 1H), 1.40 (s, 9H);

^{13}C NMR (150 MHz, $CDCl_3$) δ = 173.31, 159.85, 140.93, 129.65, 120.42, 113.68, 112.64, 81.04, 60.91, 55.34, 49.46, 36.26, 28.08 (3C);

IR (film) 3473, 2934, 2864, 1724, 1600, 1455, 1368, 1259, 1145, 1047, 779, 696 cm^{-1};

HRMS (ESI) calcd for $C_{15}H_{23}O_4$ $[M+H]^+$ *m/z* = 267.1596; found 267.1602;

$[\alpha]_D^{22}$ +20.5 (c 0.79, $CHCl_3$).

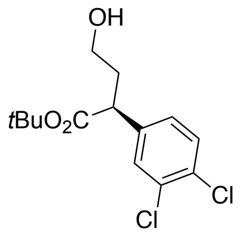

***tert*-butyl (*S*)-2-(3,4-dichlorophenyl)-4-hydroxybutanoate (3g):** Compound **3g** was synthesized following the general procedure 1 Part A and B, using *tert*-butyl 2-(7-(*tert*-butoxy)-6-chloro-7-oxohept-1-en-1-yl)cyclopropane-1-carboxylate **1b** (71.8 mg, 0.2 mmol) and 3,4-dichlorophenylmagnesium bromide (0.8 mL, 0.5 M solution in THF, 0.4 mmol). The product **3g** was obtained as a colorless liquid (43.9 mg, 72% yield, 85:15 er) after purified by Isolera™ Flash Systems silica gel chromatography with prepacked silica-gel cartridges (SNAP Ultra; Biotage) and a gradient elution pentane/Et_2O (90:10) to pentane/Et_2O (50:50). The er was determined by HPLC analysis on Daicel Chiralcel OJ-H column (Hexane/*i*-PrOH 99:1, 1.0 mL/min, 214 nm; t_r (major) = 11.71 min, t_r

(minor)= 12.77 min).

^{1}H NMR (600 MHz, CDCl$_3$) δ = 7.41–7.38 (m, 2H), 7.15 (dd, *J* = 8.3, 2.1 Hz, 1H), 3.68–3.65 (m, 2H), 3.58–3.54 (m, 1H), 2.31–2.25 (m, 1H), 1.91 (dddd, *J* = 14.1, 7.1, 6.2, 5.3 Hz, 1H), 1.45–1.43 (m, 1H), 1.40 (s, 9H);

^{13}C NMR (150 MHz, CDCl$_3$) δ = 172.52, 139.59, 132.70, 131.37, 130.63, 130.16, 127.49, 81.63, 60.46, 48.46, 36.06, 28.06 (3C);

IR (film) 3421, 2978, 2931, 2882, 1726, 1707, 1473, 1369, 1257, 1149, 1032, 844, 699 cm^{-1};

HRMS (ESI) calcd for $C_{14}H_{18}O_3Cl_2Na$ $[M+Na]^+$ *m/z* = 327.0531; found 327.0530;

$[\alpha]_D^{22}$ +19.2 (c 0.40, CHCl$_3$).

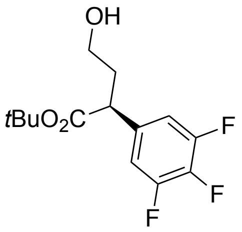

***tert*-butyl (*S*)-4-hydroxy-2-(3,4,5-trifluorophenyl)butanoate (3h):** Compound **3h** was synthesized following the general procedure 1 Part A and B, using *tert*-butyl 2-(7-(*tert*-butoxy)-6-chloro-7-oxohept-1-en-1-yl)cyclopropane-1-carboxylate **1b** (71.8 mg, 0.2 mmol) and 3,4,5-trifluorophenylmagnesium bromide (1.3 mL, 0.3 M solution in THF, 0.4 mmol). The product **3h** was obtained as a colorless liquid (29 mg, 50% yield, 88:12 er) after purified by Isolera™ Flash Systems silica gel chromatography with prepacked silica-gel cartridges (SNAP Ultra; Biotage) and a gradient elution pentane/Et$_2$O (90:10) to pentane/Et$_2$O (55:45). The er was determined by HPLC analysis on Daicel Chiralcel OJ-H column (Hexane/*i*-PrOH 99.5:0.5, 0.7 mL/min, 214 nm; t_r (major) = 11.52 min, t_r (minor)= 12.18 min).

^{1}H NMR (600 MHz, CDCl$_3$) δ = 6.98–6.93 (m, 2H), 3.69–3.63 (m, 2H), 3.55 (ddt, *J* = 10.6, 7.4, 5.2 Hz, 1H), 2.26 (dddd, *J* = 14.0, 8.1, 7.5, 5.3 Hz, 1H), 1.88 (dddd, *J* = 14.0, 7.0, 6.1, 5.1 Hz, 1H), 1.45 (t, *J* = 5.1 Hz, 1H), 1.40 (s, 9H);

^{13}C NMR (150 MHz, CDCl$_3$) δ = 172.21, 151.26 (ddd, *J* = 250.0, 9.8, 3.9 Hz, 2C), 139.05 (dt, *J* = 250.8, 15.2 Hz, 1C), 135.66–135.53 (m, 1C), 112.25 (dd, *J* = 17.0, 4.5 Hz, 2C), 81.83, 60.28, 48.48, 36.02, 28.04 (3C);

^{19}F NMR (565 MHz, $CDCl_3$) δ = -134.20 (d, *J* = 20.5 Hz, 2F), -162.39 (t, *J* = 20.5 Hz, 1F);

IR (film) 3372, 2978, 2961, 2930, 1728, 1705, 1530, 1450, 1152, 1041, 846 cm^{-1};

HRMS (ESI) calcd for $C_{14}H_{17}O_3F_3Na$ $[M+Na]^+$ *m/z* = 313.1028; found 313.1025;

$[\alpha]_D^{22}$ +17.2 (c 0.60, $CHCl_3$).

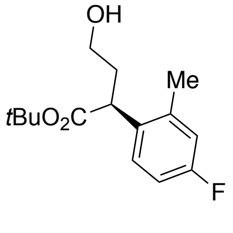

***tert*-butyl (*S*)-2-(4-fluoro-2-methylphenyl)-4-hydroxybutanoate (3i):** Compound **3i** was synthesized following the general procedure 1 Part A and B, using *tert*-butyl 2-(7-(*tert*-butoxy)-6-chloro-7-oxohept-1-en-1-yl)cyclopropane-1-carboxylate **1b** (71.8 mg, 0.2 mmol) and 4-fluoro-2-methylphenylmagnesium bromide (0.8 mL, 0.5 M solution in THF, 0.4 mmol). The product **3i** was obtained as a colorless liquid (24.6 mg, 46% yield, 90:10 er) after purified by Isolera™ Flash Systems silica gel chromatography which was performed on prepacked silica-gel cartridges (SNAP Ultra; Biotage) with gradient elution pentane/Et_2O (90:10) to pentane/Et_2O (55:45). The er was determined by HPLC analysis on Daicel Chiralcel OJ-H column (Hexane/*i*-PrOH 95:5, 0.7 mL/min, 214 nm; t_r (major) = 7.52 min, t_r (minor)= 8.25 min).

^{1}H NMR (600 MHz, $CDCl_3$) δ = 7.22 (dd, *J* = 8.3, 5.8 Hz, 1H), 6.88–6.84 (m, 2H), 3.93 (dd, *J* = 8.1, 6.7 Hz, 1H), 3.70–3.65 (m, 1H), 3.57 (ddt, *J* = 10.4, 7.4, 5.2 Hz, 1H), 2.38 (s, 3H), 2.31 (dddd, *J* = 14.1, 8.2, 7.4, 5.3 Hz, 1H), 1.89 (dtd, *J* = 14.1, 6.4, 5.2 Hz, 1H), 1.49–1.47 (m, 1H), 1.37 (s, 9H);

^{13}C NMR (150 MHz, $CDCl_3$) δ = 173.61, 161.57 (d, *J* = 244.8 Hz, 1C), 138.75 (d, *J* = 7.5 Hz, 1C), 133.71 (d, *J* = 2.8 Hz, 1C), 128.32 (d, *J* = 8.4 Hz, 1C), 117.14 (d, *J* = 21.0 Hz, 1C), 113.10 (d, *J* = 21.0 Hz, 1C), 81.07, 60.92, 44.10, 35.66, 28.07 (3C), 20.01;

^{19}F NMR (565 MHz, $CDCl_3$) δ = -116.71;

IR (film) 3436, 2977, 2932, 2879, 1725, 1590, 1498, 1368, 1251, 1143, 1046, 954, 862, 844 cm^{-1};

HRMS (ESI) calcd for $C_{15}H_{21}O_3FNa$ $[M+Na]^+$ *m/z* = 291.1372; found 291.1374;

[α]$_D^{22}$ +47.4 (c 0.23, $CHCl_3$).

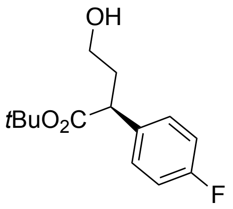

***tert*-butyl (*S*)-2-(4-fluorophenyl)-4-hydroxybutanoate (3j):** Compound **3j** was synthesized following the general procedure 1 Part A and B, using *tert*-butyl 2-(7-(*tert*-butoxy)-6-chloro-7-oxohept-1-en-1-yl)cyclopropane-1-carboxylate **1b** (71.8 mg, 0.2 mmol) and 4-fluorophenylmagnesium bromide (0.4 mL, 1.0 M solution in THF, 0.4 mmol). The product **3j** was obtained as a colorless liquid (41.7 mg, 82% yield, 87:13 er) after purified by Isolera™ Flash Systems silica gel chromatography with prepacked silica-gel cartridges (SNAP Ultra; Biotage) and a gradient elution pentane/Et_2O (90:10) to pentane/Et_2O (55:45). The er was determined by HPLC analysis on Daicel Chiralcel OJ-H column (Hexane/*i*-PrOH 99:1, 0.7 mL/min, 214 nm; t_r (minor) = 22.57 min, t_r (major)= 23.29 min).

^{1}H NMR (600 MHz, $CDCl_3$) δ = 7.28–7.25 (m, 2H), 7.02–6.98 (m, 2H), 3.68–3.63 (m, 2H), 3.58–3.54 (m, 1H), 2.29 (dtd, *J* = 13.6, 7.7, 5.6 Hz, 1H), 1.95–1.90 (m, 1H), 1.51 (bs, 1H), 1.38 (s, 9H);

^{13}C NMR (150 MHz, $CDCl_3$) δ = 173.32, 162.10 (d, *J* = 245.4 Hz, 1C), 135.09 (d, *J* = 3.1 Hz, 1C), 129.55 (d, *J* = 7.9 Hz, 2C), 115.54 (d, *J* = 21.4 Hz, 2C), 81.18, 60.71, 48.59, 36.27, 28.06 (3C);

^{19}F NMR (565 MHz, $CDCl_3$) δ = -115.79;

IR (film) 3416, 2978, 2931, 2880, 1724, 1604, 1509, 1368, 1223, 1144, 1047, 839 cm^{-1};

HRMS (ESI) calcd for $C_{14}H_{19}O_3FNa$ $[M+Na]^+$ *m/z* = 277.1216; found 277.1214;

[α]$_D^{22}$ +20.6 (c 0.71, $CHCl_3$).

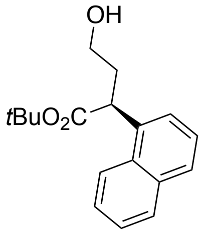

***tert*-butyl (*S*)-4-hydroxy-2-(naphthalen-1-yl)butanoate (3k):** Compound **3k** was synthesized following the general procedure 1 Part A and B, using *tert*-butyl 2-(7-(*tert*-butoxy)-6-chloro-7-oxohept-1-en-1-yl)cyclopropane-1-carboxylate **1b** (71.8 mg, 0.2 mmol) and 1-naphthylmagnesium bromide (1.6 mL, 0.25 M solution in THF, 0.4 mmol). The product **3k** was obtained as a colorless liquid (4.6 mg, 8% yield, 77:23 er) after purified by Isolera™ Flash Systems silica gel chromatography which was performed on prepacked silica-gel cartridges (SNAP Ultra; Biotage) with gradient elution pentane/Et_2O (90:10) to pentane/Et_2O (60:40). The er was determined by HPLC analysis on Daicel Chiralcel OJ-H column (Hexane/*i*-PrOH 95:5, 0.7 mL/min, 214 nm; t_r (minor) = 11.12 min, t_r (major)= 13.67 min).

^{1}H NMR (600 MHz, $CDCl_3$) δ = 8.16 (d, *J* = 8.4 Hz, 1H), 7.87 (dd, *J* = 8.1, 1.4 Hz, 1H), 7.77 (dt, *J* = 7.7, 1.1 Hz, 1H), 7.55–7.52 (m, 1H), 7.50–7.43 (m, 3H), 4.54 (dd, *J* = 8.5, 5.9 Hz, 1H), 3.75–3.71 (m, 1H), 3.68–3.63 (m, 1H), 2.49 (dddd, *J* = 13.9, 8.5, 7.0, 5.1 Hz, 1H), 2.09 (dtd, *J* = 14.2, 6.4, 5.4 Hz, 1H), 1.63 (t, *J* = 5.3 Hz, 1H), 1.36 (s, 9H);

^{13}C NMR (151 MHz, $CDCl_3$) δ = 173.91, 135.94, 134.20, 131.70, 129.05, 127.77, 126.31, 125.74, 125.65, 124.91, 123.53, 81.22, 61.20, 44.84, 35.96, 28.05 (3C);

IR (film) 3406, 3049, 2977, 2932, 2881, 1724, 1368, 1256, 1149, 1052, 844, 777 cm^{-1};

HRMS (ESI) calcd for $C_{18}H_{23}O_3$ $[M+H]^+$ *m/z* = 287.1647; found 287.1644;

$[\alpha]_D^{22}$ +32.3 (c 0.10, $CHCl_3$).

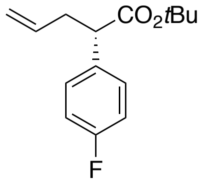

***tert*-butyl (*S*)-2-(4-fluorophenyl)pent-4-enoate (2l):** Compound **2l** was synthesized following the general procedure 1 Part A, using *tert*-butyl 2-chloropent-4-enoate **1d** (38.1 mg, 0.2 mmol) and 4-fluorophenylmagnesium bromide (0.4 mL, 1.0 M solution in THF, 0.4 mmol). The product **2l** was obtained as a colorless liquid (27.5 mg, 55% yield, 87:13 er) after flash chromatography on silica gel with Hexane/CH_2Cl_2 (5:1) to Hexane/CH_2Cl_2 (1:1). The er was determined by HPLC analysis on Daicel Chiralcel OJ-H column (Hexane/*i*-PrOH 99.9:0.1, 0.7 mL/min, 214 nm; t_r (minor) = 6.85 min, t_r (major)= 7.33

min).

^{1}H NMR (600 MHz, CDCl$_3$) δ = 7.28–7.25 (m, 2H), 7.01–6.98 (m, 2H), 5.71 (ddt, *J* = 17.0, 10.2, 6.8 Hz, 1H), 5.08–4.99 (m, 2H), 3.51 (dd, *J* = 8.5, 7.0 Hz, 1H), 2.75 (dddt, *J* = 14.3, 8.4, 7.0, 1.3 Hz, 1H), 2.43 (dtt, *J* = 14.6, 6.8, 1.4 Hz, 1H), 1.39 (s, 9H);

^{13}C NMR (150 MHz, CDCl$_3$) δ = 172.63, 162.08 (d, *J* = 245.3 Hz, 1C), 135.42, 135.01 (d, *J* = 3.3 Hz, 1C), 129.51 (d, *J* = 8.1 Hz, 2C), 117.03, 115.44 (d, *J* = 21.4 Hz, 2C), 81.05, 51.75, 37.86, 28.10 (3C);

^{19}F NMR (565 MHz, CDCl$_3$) δ = -115.91;

IR (film) 2980, 2932, 1728, 1510, 1369, 1226, 1146, 839 cm^{-1};

HRMS (ESI) calcd for $C_{15}H_{20}O_2F$ $[M+H]^+$ *m/z* = 251.1447; found 251.1451;

$[\alpha]_D^{22}$ +19.7 (c 0.76, CHCl$_3$).

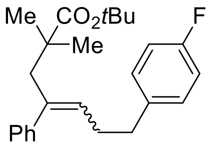

***tert*-butyl 7-(4-fluorophenyl)-2,2-dimethyl-4-phenylhept-4-enoate (7a):** Compound **7a** was synthesized following the general procedure 2, using (1-cyclopropylvinyl)benzene (28.9 mg, 0.2 mmol), *tert*-butyl 2-bromo-2-methylpropanoate (245.4 mg, 1.1 mmol) and 4-fluorophenylmagnesium bromide (1.6 mL, 1.0 M solution in THF, 1.6 mmol). The product **7a** was obtained as a colorless liquid (62.7 mg, 82% yield, 3.6:1 *E*/*Z*) after purified by flash chromatography on silica gel with Hexane/CH_2Cl_2 (2:1).

(*E*)-7a (major): **^{1}H NMR (600 MHz, CDCl$_3$)** δ = 7.28–7.22 (m, 4H), 7.20–7.17 (m, 1H), 7.16–7.11 (m, 2H), 6.98–6.93 (m, 2H), 5.59 (t, *J* = 7.2 Hz, 1H), 2.72 (s, 2H), 2.68 (t, *J* = 7.7 Hz, 2H), 2.47 (q, *J* = 7.5 Hz, 2H), 1.25 (s, 9H), 0.94 (s, 6H);

(*Z*)-7a (minor): **^{1}H NMR (600 MHz, CDCl$_3$)** δ = 7.28–7.22 (m, 2H), 7.21–7.16 (m, 1H), 7.06–7.01 (m, 2H), 7.01–6.98 (m, 2H), 6.92–6.87 (m, 2H), 5.46 (t, *J* = 7.3 Hz, 1H), 2.60 (s, 2H), 2.57 (t, *J* = 7.6 Hz, 2H), 2.21 (q, *J* = 7.5 Hz, 2H), 1.25 (s, 9H), 0.97 (s, 6H);

(*E*)-7a (major): **^{13}C NMR (150 MHz, CDCl$_3$)** δ = 177.07, 161.47 (d, *J* = 243.3 Hz, 1C), 145.10, 138.67, 137.57 (d, *J* = 3.2 Hz, 1C), 132.44, 129.96 (d, *J* = 7.8 Hz, 2C), 128.20 (2C), 127.18 (2C), 126.72, 115.17 (d, *J* = 21.4 Hz, 2C), 79.89, 43.74, 38.78, 35.25, 31.64, 27.89 (3C), 25.67 (2C);

(*Z*)-7a (minor): **^{13}C NMR (150 MHz, CDCl$_3$)** δ = 176.79, 161.38 (d, *J* = 243.2 Hz, 1C), 141.40, 138.82, 137.53 (d, *J* = 3.3 Hz, 2C), 130.26, 129.89 (d, *J* = 7.8 Hz, 2C), 128.83 (2C), 128.02 (2C), 126.63, 115.02 (d, *J* = 22.0 Hz, 2C), 79.69, 49.27, 43.38, 35.47, 30.92, 27.96 (3C), 25.93 (2C);

(*E*)-7a (major): **^{19}F NMR (565 MHz, CDCl$_3$)** δ = -117.70;

(*Z*)-7a (minor): **^{19}F NMR (565 MHz, CDCl$_3$)** δ = -117.95;

IR (film) 2975, 2929, 1718, 1509, 1221, 1131, 731, 699 cm^{-1};

HRMS (DART) calcd for $C_{25}H_{32}O_2F$ $[M+H]^+$ *m/z* = 383.2386; found 383.2387.

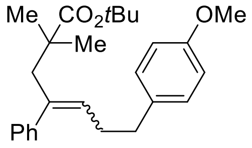

***tert*-butyl 7-(4-methoxyphenyl)-2,2-dimethyl-4-phenylhept-4-enoate (7b):** Compound **7b** was synthesized following the general procedure 2, using (1-cyclopropylvinyl)benzene (28.9 mg, 0.2 mmol), *tert*-butyl 2-bromo-2-methylpropanoate (245.4 mg, 1.1 mmol) and 4-methoxyphenylmagnesium bromide (3.2 mL, 0.5 M solution in THF, 1.6 mmol). The product **7b** was obtained as a colorless liquid (63.9 mg, 81% yield, 3.6:1 *E*/*Z*) after purified by flash chromatography on silica gel with Hexane/CH_2Cl_2 (1:1).

(*E*)-7b (major): **^{1}H NMR (600 MHz, CDCl$_3$)** δ = 7.29–7.22 (m, 4H), 7.20–7.16 (m, 1H), 7.14–7.09 (m, 2H), 6.86–6.80 (m, 2H), 5.63 (t, *J* = 7.2 Hz, 1H), 3.78 (s, 3H), 2.75 (s, 2H), 2.66 (dd, *J* = 8.9, 6.7 Hz, 2H), 2.49–2.45 (m, 2H), 1.25 (s, 9H), 0.96 (s, 6H);

(*Z*)-7b (minor): **^{1}H NMR (600 MHz, CDCl$_3$))** δ = 7.30–7.22 (m, 2H), 7.21–7.15 (m, 1H), 7.08–7.05 (m, 2H), 7.01–6.96 (m, 2H), 6.79–6.75 (m, 2H), 5.49 (t, *J* = 7.3 Hz, 1H), 3.76 (s, 3H), 2.61 (s, 2H), 2.55 (dd, *J* = 8.6, 6.8 Hz, 2H), 2.21 (q, *J* = 7.5 Hz, 2H), 1.26 (s, 9H), 0.98 (s, 6H);

(*E*)-7b (major): **^{13}C NMR (150 MHz, CDCl$_3$)** δ = 177.11, 157.99, 145.18, 138.31, 134.12, 132.91, 129.49 (2C), 128.16 (2C), 127.20 (2C), 126.63, 113.91 (2C), 79.83, 55.41, 43.73, 38.75, 35.18, 31.84, 27.89 (3C), 25.64 (2C);

(*Z*)-7b (minor): **^{13}C NMR (150 MHz, CDCl$_3$)** δ = 176.83, 157.88, 141.48, 138.41, 132.91, 130.73, 129.43 (2C), 128.88 (2C), 127.98 (2C), 126.55, 113.79 (2C), 79.66, 55.39, 49.25, 43.39, 35.44, 31.12, 27.96 (3C), 25.92 (2C);

IR (film) 2974, 2931, 1718, 1512, 1245, 1130, 699 cm^{-1};

HRMS (DART) calcd for $C_{26}H_{35}O_3$ $[M+H]^+$ *m/z* = 395.2586; found 395.2895.

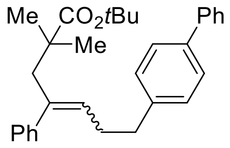

***tert*-butyl 7-([1,1'-biphenyl]-4-yl)-2,2-dimethyl-4-phenylhept-4-enoate (7c):** Compound **7c** was synthesized following the general procedure 2, using (1-cyclopropylvinyl)benzene (28.9 mg, 0.2 mmol), *tert*-butyl 2-bromo-2-methylpropanoate (245.4 mg, 1.1 mmol) and 4-biphenylmagnesium bromide (3.2 mL, 0.5 M solution in THF, 1.6 mmol). The product **7c** was obtained as a colorless liquid (74 mg, 84% yield, 3.5:1 *E*/*Z*) after purified by flash chromatography on silica gel with Hexane/CH_2Cl_2 (2:1).

(*E*)-7c (major): **^{1}H NMR (600 MHz, CDCl$_3$)** δ = 7.64–7.60 (m, 2H), 7.57–7.53 (m, 2H), 7.47–7.43 (m, 2H), 7.37–7.33 (m, 1H), 7.33–7.27 (m, 6H), 7.24–7.19 (m, 1H), 5.69 (t, *J* = 7.2 Hz, 1H), 2.81 (s, 2H), 2.81–2.78 (m, 2H) 2.57 (dt, *J* = 9.4, 7.2 Hz, 2H), 1.28 (s, 9H), 1.00 (s, 6H);

(*Z*)-7c (minor): **^{1}H NMR (600 MHz, CDCl$_3$))** δ = 7.60–7.57 (m, 2H), 7.52–7.47 (d, *J* = 8.1 Hz, 2H), 7.47–7.41 (m, 2H), 7.37–7.33 (m, 1H), 7.32–7.27 (m, 2H), 7.24–7.19 (m, 1H), 7.19–7.15 (m, 2H), 7.12–7.08 (m, 2H), 5.55 (t, *J* = 7.3 Hz, 1H), 2.66 (dd, *J* = 8.6, 6.8 Hz, 2H), 2.65 (s, 2H), 2.31 (q, *J* = 7.5 Hz, 2H), 1.29 (s, 9H), 1.02 (s, 6H);

(*E*)-7c (major): **^{13}C NMR (150 MHz, CDCl$_3$)** δ = 177.10, 145.14, 141.27, 141.14, 139.03, 138.51, 132.75 (2C), 129.05 (2C), 128.85 (2C), 128.20 (2C), 127.22 (4C), 127.15(2C), 126.69, 79.87, 43.75, 38.81, 35.74, 31.56, 27.90 (3C), 25.68 (2C);

(*Z*)-7c (minor): **^{13}C NMR (150 MHz, CDCl$_3$)** δ = 176.82, 141.46, 141.31, 141.11, 138.87, 138.65, 130.53 (2C), 129.01 (2C), 128.88 (2C), 128.83 (2C), 128.02 (2C), 127.12 (2C), 127.10 (2C), 126.60, 79.68, 49.28, 43.42, 35.97, 30.85, 27.97 (3C), 25.95 (2C);

IR (film) 2974, 2929, 1718, 1130, 697 cm^{-1};

HRMS (DART) calcd for $C_{31}H_{37}O_2$ $[M+H]^+$ *m/z* = 441.2794; found 441.2786.

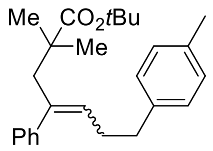

***tert*-butyl 2,2-dimethyl-4-phenyl-7-(*p*-tolyl)hept-4-enoate (7d):** Compound **7d** was synthesized following the general procedure 2, using (1-cyclopropylvinyl)benzene (28.9 mg, 0.2 mmol), *tert*-butyl 2-bromo-2-methylpropanoate (245.4 mg, 1.1 mmol) and *p*-tolylmagnesium bromide (1.6 mL, 1.0 M solution in THF, 1.6 mmol). The product **7d** was obtained as a colorless liquid (61.3 mg, 81% yield, 3.3:1 *E*/*Z*) after purified by flash chromatography on silica gel with Hexane/CH_2Cl_2 (2:1).

(*E*)-7d (major): **^{1}H NMR (600 MHz, $CDCl_3$)** δ = 7.29–7.22 (m, 4H), 7.20–7.15 (m, 1H), 7.09 (s, 4H), 5.64 (t, *J* = 7.2 Hz, 1H), 2.77 (s, 2H), 2.68 (dd, *J* = 9.1, 6.7 Hz, 2H), 2.48 (dt, *J* = 9.4, 7.2 Hz, 2H), 2.31 (s, 3H), 1.25 (s, 9H), 0.96 (s, 6H);

(*Z*)-7d (minor): **^{1}H NMR (600 MHz, $CDCl_3$))** δ = 7.27–7.22 (m, 2H), 7.20–7.15 (m, 1H), 7.12–7.05 (m, 2H), 7.05–7.02 (d, *J* = 7.8 Hz, 2H), 6.98–6.94 (d, *J* = 8.0 Hz, 2H), 5.50 (t, *J* = 7.3 Hz, 1H), 2.61 (s, 2H), 2.57 (dd, *J* = 8.8, 6.8 Hz, 2H), 2.28 (s, 3H), 2.22 (q, *J* = 7.5 Hz, 2H), 1.26 (s, 9H), 0.98 (s, 6H);

(*E*)-7d (major): **^{13}C NMR (150 MHz, $CDCl_3$)** δ = 177.10, 145.17, 138.94, 138.28, 135.41, 132.95, 129.15 (2C), 128.47 (2C), 128.16 (2C), 127.21 (2C), 126.63, 79.82, 43.72, 38.75, 35.66, 31.75, 27.88 (3C), 25.64 (2C), 21.14;

(*Z*)-7d (minor): **^{13}C NMR (150 MHz, $CDCl_3$)** δ = 176.82, 141.48, 138.91, 138.37, 135.24, 130.78, 129.02 (2C), 128.88 (2C), 128.42 (2C), 127.98 (2C), 126.55, 79.65, 49.25, 43.39, 35.93, 31.03, 27.95 (3C), 25.92 (2C), 21.10;

IR (film) 2974, 2927, 1719, 1130, 698 cm^{-1};

HRMS (DART) calcd for $C_{26}H_{35}O_2$ $[M+H]^+$ *m/z* = 379.2637; found 379.2631.

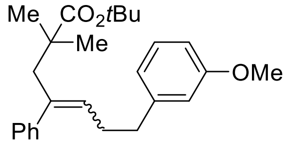

***tert*-butyl 7-(3-methoxyphenyl)-2,2-dimethyl-4-phenylhept-4-enoate (7e):** Compound **7e** was synthesized following the general procedure 2, using (1-cyclopropylvinyl)benzene

(28.9 mg, 0.2 mmol), *tert*-butyl 2-bromo-2-methylpropanoate (245.4 mg, 1.1 mmol) and 3-methoxyphenylmagnesium bromide (1.6 mL, 1.0 M solution in THF, 1.6 mmol). The product **7e** was obtained as a colorless liquid (67.8 mg, 86% yield, 3.4:1 *E*/*Z*) after purified by flash chromatography on silica gel with Hexane/CH_2Cl_2 (1:1).

(*E*)-7e (major): **^{1}H NMR (600 MHz, $CDCl_3$)** δ = 7.29–7.24 (m, 4H), 7.21 (t, *J* = 8.0 Hz, 1H), 7.21–7.17 (m, 1H), 6.85–6.79 (m, 1H), 6.78–6.74 (m, 2H), 5.64 (t, *J* = 7.2 Hz, 1H), 3.80 (s, 3H), 2.78 (s, 2H), 2.71 (dd, *J* = 9.0, 6.7 Hz, 2H), 2.52 (dt, *J* = 9.6, 7.2 Hz, 2H), 1.26 (s, 9H), 0.97 (s, 6H);

(*Z*)-7e (minor): **^{1}H NMR (600 MHz, $CDCl_3$))** δ = 7.29–7.24 (m, 2H), 7.21–7.17 (m, 1H), 7.17–7.13 (m, 1H), 7.09–7.05 (m, 2H), 6.71 (ddd, *J* = 8.3, 2.6, 0.9 Hz, 1H), 6.68 (dt, *J* = 7.4, 1.2 Hz, 1H), 6.62 (t, *J* = 2.0 Hz, 1H), 5.51 (t, *J* = 7.3 Hz, 1H), 3.76 (s, 3H), 2.62 (m, 2H), 2.60 (dd, *J* = 8.7, 6.8 Hz, 2H), 2.25 (q, *J* = 7.5 Hz, 2H), 1.27 (s, 9H), 0.99 (s, 6H);

(*E*)-7e (major): **^{13}C NMR (150 MHz, $CDCl_3$)** δ = 177.11, 159.80, 145.14, 143.66, 138.44, 132.80, 129.44, 128.18 (2C), 127.22 (2C), 126.67, 121.06, 114.40, 111.36, 79.87, 55.30, 43.74, 38.77, 36.15, 31.48, 27.89 (3C), 25.67 (2C);

(*Z*)-7e (minor): **^{13}C NMR (150 MHz, $CDCl_3$)** δ = 176.84, 159.71, 143.63, 141.44, 138.56, 130.59, 129.29, 128.89 (2C), 128.01 (2C), 126.59, 121.05, 114.26, 111.29, 79.69, 55.25, 49.26, 43.38, 36.42, 30.80, 27.97 (3C), 25.93 (2C);

IR (film) 2973, 2929, 1719, 1258, 1130, 697 cm^{-1};

HRMS (DART) calcd for $C_{26}H_{35}O_3$ $[M+H]^+$ *m/z* = 395.2586; found 395.2582.

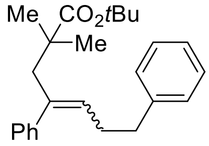

***tert*-butyl 2,2-dimethyl-4,7-diphenylhept-4-enoate (7f):** Compound **7f** was synthesized following the general procedure 2, using (1-cyclopropylvinyl)benzene (28.9 mg, 0.2 mmol), *tert*-butyl 2-bromo-2-methylpropanoate (245.4 mg, 1.1 mmol) and phenylmagnesium bromide (3.2 mL, 0.5 M solution in THF, 1.6 mmol). The product **7f** was obtained as a colorless liquid (57.6 mg, 79% yield, 3.6:1 *E*/*Z*) after purified by flash chromatography on silica gel with Hexane/CH_2Cl_2 (2:1).

(*E*)-7f (major): **^{1}H NMR (600 MHz, $CDCl_3$)** δ = 7.29–7.16 (m, 9H), 7.06 (td, *J* = 8.3, 1.3 Hz, 1H), 5.63 (t, *J* = 7.2 Hz, 1H), 2.75 (s, 2H), 2.71 (dd, *J* = 9.0, 6.7 Hz, 2H), 2.50 (dt, *J* = 9.4, 7.2 Hz, 2H), 1.24 (s, 9H), 0.95 (s, 6H);

(*Z*)-7f (minor): **^{1}H NMR (600 MHz, $CDCl_3$)** δ = 7.31–7.15 (m, 9H), 7.15–7.12 (m, 1H), 5.49 (t, *J* = 7.3 Hz, 1H), 2.62–2.59 (m, 4H), 2.29–2.20 (q, *J* = 7.5 Hz, 2H), 1.25 (s, 9H), 0.97 (s, 6H);

(*E*)-7f (major): **^{13}C NMR (150 MHz, $CDCl_3$)** δ = 177.10, 145.14, 142.00, 138.41, 132.80, 128.62 (2C), 128.47 (2C), 128.17 (2C), 127.20 (2C), 126.65, 126.01, 79.84, 43.72, 38.74, 36.11, 31.60, 27.88 (3C), 25.65 (2C);

(*Z*)-7f (minor): **^{13}C NMR (150 MHz, $CDCl_3$)** δ = 176.82, 141.96, 141.44, 138.52, 130.62, 128.86 (2C), 128.58 (2C), 128.34 (2C), 128.00 (2C), 126.57, 125.86, 79.66, 49.26, 43.38, 36.36, 30.90, 27.96 (3C), 25.92 (2C);

IR (film) 2974, 2929, 1719, 1130, 697 cm^{-1};

HRMS (DART) calcd for $C_{25}H_{33}O_2$ $[M+H]^+$ *m/z* = 365.2481; found 365.2488.

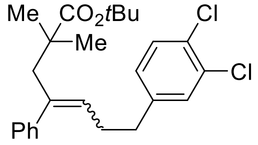

***tert*-butyl 7-(3,4-dichlorophenyl)-2,2-dimethyl-4-phenylhept-4-enoate (7g):** Compound **7g** was synthesized following the general procedure 2, using (1-cyclopropylvinyl)benzene (28.9 mg, 0.2 mmol), *tert*-butyl 2-bromo-2-methylpropanoate (245.4 mg, 1.1 mmol) and 3,4-dichlorophenylmagnesium bromide (3.2 mL, 0.5 M solution in THF, 1.6 mmol). The product **7g** was obtained as a colorless liquid (63.2 mg, 73% yield, 2.7:1 *E*/*Z*) after purified by flash chromatography on silica gel with Hexane/CH_2Cl_2 (2:1).

(*E*)-7g (major): **^{1}H NMR (600 MHz, $CDCl_3$)** δ = 7.34 (d, *J* = 8.2 Hz, 1H), 7.29 (d, *J* = 2.1 Hz, 1H), 7.28–7.23 (m, 4H), 7.22–7.17 (m, 1H), 7.06–7.00 (m, 1H), 5.57 (t, *J* = 7.2 Hz, 1H), 2.72 (s, 2H), 2.67 (dd, *J* = 8.6, 6.8 Hz, 2H), 2.48 (q, *J* = 7.5 Hz, 2H), 1.26 (s, 9H), 0.95 (s, 6H);

(*Z*)-7g (minor): **^{1}H NMR (600 MHz, $CDCl_3$)** δ = 7.28–7.23 (m, 3H), 7.22–7.17 (m, 1H), 7.12 (d, *J* = 2.1 Hz, 1H), 7.06–6.97 (m, 2H), 6.87 (dd, *J* = 8.2, 2.1 Hz, 1H), 5.44–5.42 (m,

1H), 2.60 (s, 2H), 2.55 (t, J = 7.5 Hz, 2H), 2.23 (q, J = 7.5 Hz, 2H), 1.26 (s, 9H), 0.98 (s, 6H);

(*E*)-7g (major): **^{13}C NMR (150 MHz, $CDCl_3$)** δ = 176.98, 144.93, 142.17, 139.19, 132.30, 131.75, 130.59, 130.34, 129.55, 128.23 (2C), 128.12, 127.18 (2C), 126.82, 79.94, 43.73, 38.86, 35.15, 31.07, 27.90 (3C), 25.69 (2C);

(*Z*)-7g (minor): **^{13}C NMR (150 MHz, $CDCl_3$)** δ = 176.71, 142.10, 141.19, 139.43, 132.16, 130.51, 130.17, 129.97, 129.77, 128.76 (2C), 128.10, 128.08 (2C), 126.74, 79.71, 49.27, 43.33, 35.30, 30.27, 27.94 (3C), 25.93 (2C);

IR (film) 2974, 2930, 1717, 1471, 1129, 698 cm^{-1};

HRMS (DART) calcd for $C_{25}H_{31}O_2Cl_2$ $[M+H]^+$ m/z = 433.1701; found 433.1703.

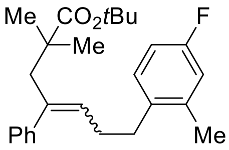

***tert*-butyl 7-(4-fluoro-2-methylphenyl)-2,2-dimethyl-4-phenylhept-4-enoate (7h):** Compound **7h** was synthesized following the general procedure 2, using (1-cyclopropylvinyl)benzene (28.9 mg, 0.2 mmol), *tert*-butyl 2-bromo-2-methylpropanoate (245.4 mg, 1.1 mmol) and 4-fluoro-2-methylphenylmagnesium bromide (3.2 mL, 0.5 M solution in THF, 1.6 mmol). The product **7h** was obtained as a colorless liquid (58.7 mg, 74% yield, 2.7:1 *E*/*Z*) after purified by flash chromatography on silica gel with Hexane/CH_2Cl_2 (2:1).

(*E*)-7h (major): **^{1}H NMR (600 MHz, $CDCl_3$)** δ = 7.27–7.21 (m, 4H), 7.21–7.11 (m, 1H), 7.06 (dd, J = 8.4, 6.0 Hz, 1H), 6.83 (dd, J = 9.8, 2.8 Hz, 1H), 6.80 (td, J = 8.4, 2.8 Hz, 1H), 5.62 (t, J = 7.3 Hz, 1H), 2.72 (s, 2H), 2.65 (dd, J = 9.1, 6.6 Hz, 2H), 2.42 (q, J = 7.5 Hz, 2H), 2.29 (s, 3H), 1.24 (s, 9H), 0.94 (s, 6H);

(*Z*)-7h (minor): **^{1}H NMR (600 MHz, $CDCl_3$)** δ = 7.27–7.21 (m, 1H), 7.21–7.11 (m, 2H), 7.03–6.97 (m, 2H), 6.91 (dd, J = 8.4, 6.0 Hz, 1H), 6.76–6.69 (m, 2H), 5.49 (t, J = 7.4 Hz, 1H), 2.60 (m, 2H), 2.51 (dd, J = 8.9, 6.7 Hz, 2H), 2.14 (q, J = 7.5 Hz, 2H), 2.09 (s, 3H), 1.25 (s, 9H), 0.97 (s, 6H);

(*E*)-7h (major): **^{13}C NMR (150 MHz, $CDCl_3$)** δ = 177.04, 161.30 (d, J = 243.2 Hz, 1C), 145.08, 138.63, 138.10 (d, J = 7.6 Hz, 1C), 135.70 (d, J = 3.2 Hz, 1C), 132.56, 130.30 (d,

J = 8.2 Hz, 1C), 128.21 (2C), 127.17 (2C), 126.72, 116.81 (d, J = 20.7 Hz, 1C), 112.55 (d, J = 20.8 Hz, 1C), 79.88, 43.69, 38.73, 32.63, 30.31, 27.89 (3C), 25.65 (2C), 19.61;

(*Z*)-7h (minor): **^{13}C NMR (150 MHz, $CDCl_3$)** δ = 176.78, 161.21 (d, J = 243.1 Hz, 1C), 141.37, 138.94, 138.14 (d, J = 7.5 Hz, 1C), 135.65 (d, J = 3.0 Hz, 1C), 130.36, 130.28 (d, J = 8.1 Hz, 1C), 128.84 (2C), 128.02 (2C), 126.62, 116.66 (d, J = 20.2 Hz, 1C), 112.35 (d, J = 20.4 Hz, 1C), 79.69, 49.35, 43.38, 32.93, 29.64, 27.96 (3C), 25.96 (2C), 19.33;

(*E*)-7h (major): **^{19}F NMR (565 MHz, $CDCl_3$)** δ = -118.11;

(*Z*)-7h (minor): **^{19}F NMR (565 MHz, $CDCl_3$)** δ = -118.38;

IR (film) 2974, 1717, 1495, 1366, 1249, 1130, 851, 698 cm^{-1};

HRMS (DART) calcd for $C_{26}H_{34}O_2F$ $[M+H]^+$ *m/z* = 397.2543; found 397.2537.

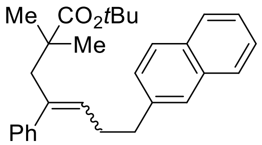

***tert*-butyl 2,2-dimethyl-7-(naphthalen-2-yl)-4-phenylhept-4-enoate (7i):** Compound **7i** was synthesized following the general procedure 2, using (1-cyclopropylvinyl)benzene (28.9 mg, 0.2 mmol), *tert*-butyl 2-bromo-2-methylpropanoate (245.4 mg, 1.1 mmol) and 2-naphthylmagnesium bromide (3.2 mL, 0.5 M solution in THF, 1.6 mmol). The product **7i** was obtained as a colorless liquid (66.3 mg, 80% yield, 3.6:1 *E*/*Z*) after purified by flash chromatography on silica gel with Hexane/CH_2Cl_2 (2:1).

(*E*)-7i (major): **^{1}H NMR (600 MHz, $CDCl_3$)** δ = 7.82–7.79 (m, 1H), 7.78 (d, J = 8.3 Hz, 2H), 7.64 (d, J = 1.7 Hz, 1H), 7.47–7.40 (m, 2H), 7.36 (dd, J = 8.4, 1.7 Hz, 1H), 7.29–7.23 (m, 4H), 7.21–7.16 (m, 1H), 5.69 (t, J = 7.2 Hz, 1H), 2.89 (dd, J = 8.8, 6.8 Hz, 2H), 2.77 (s, 2H), 2.61 (q, J = 7.3 Hz, 2H), 1.25 (s, 9H), 0.97 (s, 6H);

(*Z*)-7i (minor): **^{1}H NMR (600 MHz, $CDCl_3$)** δ = 7.80–7.76 (m, J = 8.3 Hz, 1H), 7.72 (t, J = 8.5 Hz, 2H), 7.51 (s, 1H), 7.44–7.38 (m, 2H), 7.29–7.23 (m, 2H), 7.23–7.16 (m, 2H), 7.09–7.05 (m, 2H), 5.54 (t, J = 7.3 Hz, 1H), 2.80–2.77 (m, 2H), 2.62 (s, 2H), 2.36 (q, J = 7.5 Hz, 2H), 1.25 (s, 9H), 0.98 (s, 6H);

(*E*)-7i (major): **^{13}C NMR (150 MHz, $CDCl_3$)** δ = 177.09, 145.13, 139.50, 138.53, 133.77, 132.73, 132.18, 128.19 (2C), 128.01, 127.74, 127.58, 127.49, 127.21 (2C),

126.68, 126.62, 126.02, 125.27, 79.85, 43.75, 38.79, 36.24, 31.49, 27.88 (3C), 25.67 (2C);

(*Z*)-7i (minor): **^{13}C NMR (150 MHz, $CDCl_3$)** δ = 176.81, 141.43, 139.43, 138.64, 133.71, 132.12, 130.55, 128.88 (2C), 128.01, 127.85 (2C), 127.68, 127.54, 127.51, 126.60, 126.54, 125.92, 125.17, 79.66, 49.26, 43.39, 36.45, 30.66, 27.94 (3C), 25.92 (2C);

IR (film) 2974, 2929, 1718, 1366, 1130, 851, 698 cm^{-1};

HRMS (DART) calcd for $C_{29}H_{35}O_2$ $[M+H]^+$ *m/z* = 415.2637; found 415.2631.

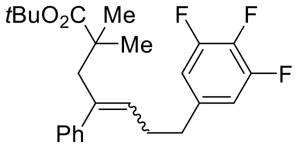

***tert*-butyl 2,2-dimethyl-4-phenyl-7-(3,4,5-trifluorophenyl)hept-4-enoate (7j):** Compound **7j** was synthesized following the general procedure 2, using (1-cyclopropylvinyl)benzene (28.9 mg, 0.2 mmol), *tert*-butyl 2-bromo-2-methylpropanoate (245.4 mg, 1.1 mmol) and 3,4,5-trifluorophenylmagnesium bromide (5.3 mL, 0.3 M solution in THF, 1.6 mmol). The product **7j** was obtained as a colorless liquid (54.4 mg, 65% yield, 3.0:1 *E*/*Z*) after purified by flash chromatography on silica gel with Hexane/CH_2Cl_2 (2:1).

(*E*)-7j (major): **^{1}H NMR (600 MHz, $CDCl_3$)** δ = 7.29–7.20 (m, 4H), 7.20–7.14 (m, 1H), 6.82–6.72 (m, 2H), 5.52 (t, *J* = 7.2 Hz, 1H), 2.70 (s, 2H), 2.63 (t, *J* = 7.7 Hz, 2H), 2.45 (q, *J* = 7.4 Hz, 2H), 1.24 (s, 9H), 0.93 (s, 6H);

(*Z*)-7j (minor): **^{1}H NMR (600 MHz, $CDCl_3$)** δ = 7.26–7.21 (m, 2H), 7.19–7.16 (m, 1H), 7.05–6.99 (m, 2H), 6.64–6.56 (m, 2H), 5.39 (t, *J* = 7.3 Hz, 1H), 2.58 (s, 2H), 2.50 (t, *J* = 7.5 Hz, 2H), 2.19 (q, *J* = 7.5 Hz, 2H), 1.24 (s, 9H), 0.96 (s, 6H);

(*E*)-7j (major): **^{13}C NMR (150 MHz, $CDCl_3$)** δ = 176.95, 151.17 (ddd, *J* = 249.2, 9.9, 4.0 Hz, 2C), 144.84, 139.43, 138.27(dt, *J* = 248.6, 15.3 Hz, 1C), 138.28–138.02 (m, 1C), 131.39, 128.27 (2C), 127.16 (2C), 126.90, 112.41 (dd, *J* = 16.3, 4.1 Hz, 2C), 79.99, 43.76, 38.91, 35.35, 30.88, 27.89 (3C), 25.71 (2C);

(*Z*)-7j (minor): **^{13}C NMR (150 MHz, $CDCl_3$)** δ = 176.69, 151.04 (ddd, *J* = 248.7, 9.4, 4.4 Hz, 2C), 141.11, 139.71, 139.27–137.24 (m, 1C), 138.10–137.97 (m, 1C), 129.23, 128.74

(2C), 128.12 (2C), 126.83, 112.31 (dd, *J* = 13.9, 4.2 Hz, 2C), 79.75, 49.28, 43.33, 35.50, 30.08, 27.94 (3C), 25.93 (2C);

(*E*)-7j (major): **^{19}F NMR (565 MHz, $CDCl_3$)** δ = -135.23 (d, *J* = 20.5 Hz, 2F), -164.59 (t, *J* = 20.5 Hz, 1F);

(*Z*)-7j (minor): **^{19}F NMR (565 MHz, $CDCl_3$)** δ = -135.57 (d, *J* = 20.5 Hz, 2F), -164.92 (t, *J* = 20.5 Hz, 1F);

IR (film) 2976, 1717, 1530, 1131, 1043, 849, 698 cm^{-1};

HRMS (DART) calcd for $C_{25}H_{30}O_2F_3$ $[M+H]^+$ *m/z* = 419.2198; found 419.2193.

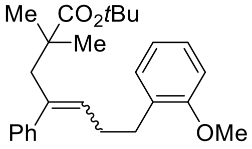

***tert*-butyl 7-(2-methoxyphenyl)-2,2-dimethyl-4-phenylhept-4-enoate (7k):** Compound **7k** was synthesized following the general procedure 2, using (1-cyclopropylvinyl)benzene (28.9 mg, 0.2 mmol), *tert*-butyl 2-bromo-2-methylpropanoate (245.4 mg, 1.1 mmol) and 2-methoxyphenylmagnesium bromide (1.6 mL, 1.0 M solution in THF, 1.6 mmol). The product **7k** was obtained as a colorless liquid (72.6 mg, 92% yield, 4.0:1 *E*/*Z*) after purified by flash chromatography on silica gel with Hexane/CH_2Cl_2 (1:1).

(*E*)-7k (major): **^{1}H NMR (600 MHz, $CDCl_3$)** δ = 7.29–7.21 (m, 4H), 7.19–7.14 (m, 2H), 7.13 (dd, *J* = 7.3, 1.7 Hz, 1H), 6.87 (td, *J* = 7.4, 1.1 Hz, 1H), 6.83 (dd, *J* = 8.2, 1.2 Hz, 1H), 5.66 (t, *J* = 7.3 Hz, 1H), 3.81 (s, 3H), 2.77 (s, 2H), 2.71 (dd, *J* = 9.1, 6.6 Hz, 2H), 2.46 (q, *J* = 7.6 Hz, 2H), 1.24 (s, 9H), 0.96 (s, 6H);

(*Z*)-7k (minor): **^{1}H NMR (600 MHz, $CDCl_3$)** δ = 7.28–7.21 (m, 2H), 7.20–7.15 (m, 1H), 7.13–7.10 (m, 1H), 7.07–7.04 (m, 2H), 7.00 (dd, *J* = 7.4, 1.8 Hz, 1H), 6.85–6.78 (m, 1H), 6.77 (dd, *J* = 8.2, 1.0 Hz, 1H), 5.52 (t, *J* = 7.4 Hz, 1H), 3.71 (s, 3H), 2.65–2.58 (m, 4H), 2.21 (q, *J* = 7.5 Hz, 2H), 1.26 (s, 9H), 0.97 (s, 6H);

(*E*)-7k (major): **^{13}C NMR (150 MHz, $CDCl_3$)** δ = 177.19, 157.65, 145.28, 137.96, 133.53, 130.41, 130.09, 128.12 (2C), 127.27, 127.23 (2C), 126.53, 120.49, 110.35, 79.75, 55.30, 43.64, 38.58, 30.67, 29.90, 27.88 (3C), 25.60 (2C);

(*Z*)-7k (minor): **^{13}C NMR (150 MHz, CDCl$_3$)** δ = 176.90, 157.57, 141.58, 138.02, 131.35, 130.38, 130.01, 128.97 (2C), 127.87 (2C), 127.09, 126.40, 120.34, 110.26, 79.63, 55.22, 49.26, 43.40, 30.79, 29.22, 27.96 (3C), 25.90 (2C);

IR (film) 2974, 1718, 1492, 1241, 1130, 750, 698 cm^{-1};

HRMS (DART) calcd for $C_{26}H_{35}O_3$ $[M+H]^+$ *m/z* = 395.2586; found 395.2589.

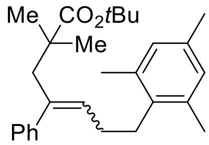

***tert*-butyl 7-mesityl-2,2-dimethyl-4-phenylhept-4-enoate (7l):** Compound **7l** was synthesized following the general procedure 2, using (1-cyclopropylvinyl)benzene (28.9 mg, 0.2 mmol), *tert*-butyl 2-bromo-2-methylpropanoate (245.4 mg, 1.1 mmol) and 2-mesitylmagnesium bromide (1.6 mL, 1.0 M solution in THF, 1.6 mmol). The product **7l** was obtained as a colorless liquid (49.6 mg, 61% yield, 2.4:1 *E*/*Z*) after purified by flash chromatography on silica gel with Hexane/CH_2Cl_2 (2:1).

(*E*)-7l (major): **^{1}H NMR (600 MHz, CDCl$_3$)** δ = 7.32–7.23 (m, 4H), 7.21–7.18 (m, 1H), 6.88–6.80 (s, 2H), 5.70 (t, *J* = 7.3 Hz, 1H), 2.80 (s, 2H), 2.72–2.66 (m, 2H), 2.40–2.28 (m, 2H), 2.32 (s, 6H), 2.26 (s, 3H), 1.25 (s, 9H), 0.97 (s, 6H);

(*Z*)-7l (minor): **^{1}H NMR (600 MHz, CDCl$_3$)** δ = 7.30–7.23 (m, 2H), 7.18–7.15 (m, 1H), 7.13–7.07 (m, 2H), 6.75 (s, 2H), 5.59 (t, *J* = 7.5 Hz, 1H), 2.65 (s, 2H), 2.56–2.49 (m, 2H), 2.20 (s, 3H), 2.10 (s, 6H), 2.08–2.01 (m, 2H), 1.29 (s, 9H), 1.03 (s, 6H);

(*E*)-7l (major): **^{13}C NMR (150 MHz, CDCl$_3$)** δ = 177.09, 145.10, 138.22, 136.11 (2C), 135.70, 135.29, 133.19, 129.09 (2C), 128.21 (2C), 127.20 (2C), 126.70, 79.87, 43.63, 38.64, 29.58, 29.30, 27.89 (3C), 25.59 (2C), 20.95, 19.97 (2C);

(*Z*)-7l (minor): **^{13}C NMR (150 MHz, CDCl$_3$)** δ = 176.85, 141.41, 138.64, 136.05 (2C), 135.72, 135.04, 130.88, 128.95 (2C), 128.90 (2C), 128.03 (2C), 126.57, 79.72, 49.46, 43.43, 29.78, 28.69, 27.99 (3C), 26.02 (2C), 20.89, 19.65 (2C);

IR (film) 2973, 1720, 1130, 850, 699 cm^{-1};

HRMS (DART) calcd for $C_{28}H_{39}O_2$ $[M+H]^+$ *m/z* = 407.2950; found 407.2959.

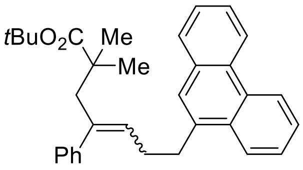

***tert*-butyl 2,2-dimethyl-7-(phenanthren-9-yl)-4-phenylhept-4-enoate (7m):** Compound **7m** was synthesized following the general procedure 2, using (1-cyclopropylvinyl)benzene (28.9 mg, 0.2 mmol), *tert*-butyl 2-bromo-2-methylpropanoate (245.4 mg, 1.1 mmol) and 9-phenanthrylmagnesium bromide (3.2 mL, 0.5 M solution in THF, 1.6 mmol). The product **7m** was obtained as a colorless liquid (67.8 mg, 73% yield, 2.9:1 *E*/*Z*) after purified by flash chromatography on silica gel with Hexane/CH_2Cl_2 (2:1).

(*E*)-7m (major): **^{1}H NMR (600 MHz, $CDCl_3$)** δ = 8.81–8.74 (m, 1H), 8.70–8.66 (m, 1H), 8.20–8.11 (m, 1H), 7.85 (dd, *J* = 7.6, 1.8 Hz, 1H), 7.71–7.57 (m, 5H), 7.33–7.18 (m, 5H), 5.81 (t, *J* = 7.2 Hz, 1H), 3.29–3.19 (m, 2H), 2.79 (s, 2H), 2.77–2.71 (m, 2H), 1.24 (s, 9H), 0.97 (s, 6H);

(*Z*)-7m (minor): **^{1}H NMR (600 MHz, $CDCl_3$)** δ = 8.71 (dd, *J* = 8.3, 1.2 Hz, 1H), 8.64 (d, *J* = 8.0 Hz, 1H), 7.88–7.82 (m, 1H), 7.79 (dd, *J* = 7.7, 1.6 Hz, 1H), 7.72–7.54 (m, 3H), 7.53 (ddd, *J* = 8.2, 6.9, 1.3 Hz, 1H), 7.49 (s, 1H), 7.34–7.17 (m, 3H), 7.12–7.06 (m, 2H), 5.67 (t, *J* = 7.4 Hz, 1H), 3.11 (dd, *J* = 8.8, 6.7 Hz, 2H), 2.66 (s, 2H), 2.47 (q, *J* = 7.6 Hz, 2H), 1.27 (s, 9H), 1.01 (s, 6H);

(*E*)-7m (major): **^{13}C NMR (150 MHz, $CDCl_3$)** δ = 177.05, 145.09, 138.66, 136.06, 132.91, 132.03, 131.34, 130.89, 129.85, 128.23, 128.21 (2C), 127.24 (2C), 126.76, 126.72 (2C), 126.38, 126.31, 126.15, 124.43, 123.42, 122.59, 79.85, 43.68, 38.86, 33.52, 30.23, 27.86 (3C), 25.66 (2C);

(*Z*)-7m (minor): **^{13}C NMR (150 MHz, $CDCl_3$)** δ = 176.82, 141.40, 138.93, 135.98, 131.99, 131.31, 130.77, 130.70, 129.80, 128.89 (2C), 128.18, 128.07 (2C), 126.66, 126.62, 126.57, 126.28, 126.15, 126.03, 124.46, 123.24, 122.52, 79.69, 49.37, 43.37, 33.83, 29.52, 27.95 (3C), 25.95 (2C);

IR (film) 2973, 2930, 1716, 1130, 725, 699 cm^{-1};

HRMS (DART) calcd for $C_{33}H_{40}NO_2$ $[M+NH_4]^+$ *m/z* = 482.3059; found 482.3052.

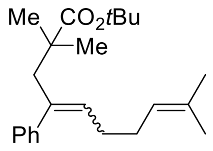

***tert*-butyl 2,2,9-trimethyl-4-phenyldeca-4,8-dienoate (7n):** Compound **7n** was synthesized following the general procedure 2, using (1-cyclopropylvinyl)benzene (28.9 mg, 0.2 mmol), *tert*-butyl 2-bromo-2-methylpropanoate (245.4 mg, 1.1 mmol) and 2-methyl-1-propenylmagnesium bromide (3.2 mL, 0.5 M solution in THF, 1.6 mmol). The product **7n** was obtained as a colorless liquid (48 mg, 70% yield, 3.0:1 *E*/*Z*) after purified by flash chromatography on silica gel with Hexane/CH_2Cl_2 (3:1).

(*E*)-7n (major): **^{1}H NMR (600 MHz, $CDCl_3$)** δ = 7.28–7.21 (m, 4H), 7.19–7.14 (m, 1H), 5.58(t, *J* = 7.2 Hz, 1H), 5.14 (ddq, *J* = 8.6, 5.7, 1.4 Hz, 1H), 2.80(s, 2H), 2.20 (q, *J* = 7.4 Hz, 2H), 2.08 (q, *J* = 7.4 Hz, 2H), 1.68 (s, 3H), 1.59 (s, 3H), 1.25 (s, 9H), 0.97 (s, 6H);

(*Z*)-7n (minor): **^{1}H NMR (600 MHz, $CDCl_3$)** δ = 7.27–7.21 (m, 2H), 7.18–7.14 (m, 1H), 7.13–7.10 (m, 2H), 5.46 (t, *J* = 6.6 Hz, 1H), 5.02 (tdd, *J* = 7.3, 3.1, 1.6 Hz, 1H), 2.61 (s, 2H), 2.00–1.90 (m, 4H), 1.63 (s, 3H), 1.53 (s, 3H), 1.27 (s, 9H), 0.97 (s, 6H);

(*E*)-7n (major): **^{13}C NMR (150 MHz, $CDCl_3$)** δ = 177.21, 145.32, 137.82, 133.68, 132.09, 128.15 (2C), 127.21 (2C), 126.54, 124.15, 79.82, 43.77, 38.75, 29.89, 28.33, 27.90 (3C), 25.86, 25.66 (2C), 17.89;

(*Z*)-7n (minor): **^{13}C NMR (150 MHz, $CDCl_3$)** δ = 176.88, 141.69, 137.84, 131.82, 131.45, 128.98 (2C), 127.96 (2C), 126.47, 124.26, 79.65, 49.32, 43.45, 29.30, 28.59, 27.98 (3C), 25.92 (2C), 25.81, 17.79;

IR (film) 2973, 2929, 1721, 1130, 698 cm^{-1};

HRMS (DART) calcd for $C_{23}H_{35}O_2$ $[M+H]^+$ *m/z* = 343.2637; found 343.2640.

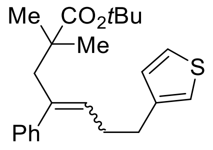

***tert*-butyl 2,2-dimethyl-4-phenyl-7-(thiophen-3-yl)hept-4-enoate (7o):** Compound **7o** was synthesized following the general procedure 2, using (1-cyclopropylvinyl)benzene (28.9 mg, 0.2 mmol), *tert*-butyl 2-bromo-2-methylpropanoate (245.4 mg, 1.1 mmol) and thiophen-3-ylmagnesium bromide (3.6 mL, 0.45 M solution in THF, 1.6 mmol). The

product **7o** was obtained as a colorless liquid (48.2 mg, 65% yield, 3.0:1 *E*/*Z*) after purified by flash chromatography on silica gel with Hexane/CH_2Cl_2 (3:2).

(*E*)-7o (major): **^{1}H NMR (600 MHz, $CDCl_3$)** δ = 7.31–7.24 (m, 5H), 7.23–7.18 (m, 1H), 6.98 (dt, *J* = 3.2, 0.8 Hz, 2H), 5.65 (t, *J* = 7.2 Hz, 1H), 2.79 (s, 2H), 2.77 (dd, *J* = 8.7, 6.7 Hz, 2H), 2.56–2.51 (m, 2H), 1.28 (s, 9H), 0.99 (s, 6H);

(*Z*)-7o (minor): **^{1}H NMR (600 MHz, $CDCl_3$)** δ = 7.33–7.24 (m, 2H), 7.23–7.18 (m, 2H), 7.12–7.07 (m, 2H), 6.88–6.82 (m, 2H), 5.51 (t, *J* = 7.3 Hz, 1H), 2.68–2.62 (m, 4H), 2.28 (q, *J* = 7.4 Hz, 2H), 1.29 (s, 9H), 1.00 (s, 6H);

(*E*)-7o (major): **^{13}C NMR (150 MHz, $CDCl_3$)** δ = 177.11, 145.14, 142.35, 138.53, 132.79, 128.42, 128.20 (2C), 127.20 (2C), 126.69, 125.41, 120.43, 79.89, 43.76, 38.79, 30.66, 30.40, 27.91 (3C), 25.68 (2C);

(*Z*)-7o (minor): **^{13}C NMR (150 MHz, $CDCl_3$)** δ = 176.83, 142.34, 141.47, 138.60, 130.64, 128.88 (2C), 128.42, 128.04 (2C), 126.62, 125.20, 120.26, 79.71, 49.26, 43.42, 30.68, 30.00, 27.98 (3C), 25.93 (2C);

IR (film) 2974, 2928, 1718, 1130, 698 cm^{-1};

HRMS (DART) calcd for $C_{23}H_{31}O_2S$ $[M+H]^+$ *m/z* = 371.2045; found 371.2053.

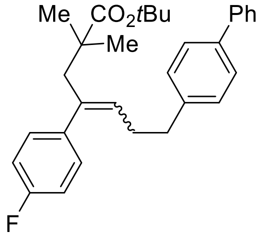

***tert*-butyl 7-([1,1'-biphenyl]-4-yl)-4-(4-fluorophenyl)-2,2-dimethylhept-4-enoate (7p):** Compound **7p** was synthesized following the general procedure 2, using 1-(1-cyclopropylvinyl)-4-fluorobenzene (32.5 mg, 0.2 mmol), *tert*-butyl 2-bromo-2-methylpropanoate (245.4 mg, 1.1 mmol) and 4-biphenylmagnesium bromide (3.2 mL, 0.5 M solution in THF, 1.6 mmol). The product **7p** was obtained as a colorless liquid (67.9 mg, 74% yield, 3.6:1 *E*/*Z*) after purified by flash chromatography on silica gel with Hexane/CH_2Cl_2 (2:1).

(*E*)-7p (major): **^{1}H NMR (600 MHz, $CDCl_3$)** δ = 7.63–7.59 (m, 2H), 7.56–7.52 (m, 2H), 7.45 (t, *J* = 7.7 Hz, 2H), 7.37–7.33 (m, 1H), 7.29 (d, *J* = 8.1 Hz, 2H), 7.26–7.23 (m, 2H),

6.97 (t, J = 8.7 Hz, 2H), 5.63 (t, J = 7.2 Hz, 1H), 2.78 (dd, J = 9.0, 6.9 Hz, 2H), 2.77 (s, 2H), 2.58–2.53 (m, 2H), 1.28 (s, 9H), 1.00 (s, 6H);

(*Z*)-7p (minor): **^{1}H NMR (600 MHz, $CDCl_3$)** δ = 7.59–7.57 (m, 2H), 7.51–7.47 (m, 2H), 7.45–7.40 (m, 2H), 7.35–7.32 (m, 1H), 7.18–7.12 (m, 2H), 7.05–7.01 (m, 2H), 6.99–6.93 (m, 2H), 5.55 (t, J = 7.3 Hz, 1H), 2.67 (t, J = 7.6 Hz, 2H), 2.62 (s, 2H), 2.28 (q, J = 7.5 Hz, 2H), 1.29 (s, 9H), 1.01 (s, 6H);

(*E*)-7p (major): **^{13}C NMR (150 MHz, $CDCl_3$)** δ = 176.97, 162.03 (d, J = 245.2 Hz, 1C), 141.22, 141.03 (d, J = 3.2 Hz, 1C), 141.02, 139.07, 137.56, 132.79, 129.03 (2C), 128.86 (2C), 128.69 (d, J = 7.8 Hz, 2C), 127.24 (2C), 127.19, 127.14 (2C), 114.94 (d, J = 21.1 Hz, 2C), 79.96, 43.66, 39.04, 35.69, 31.49, 27.89 (3C), 25.73 (2C);

(*Z*)-7p (minor): **^{13}C NMR (150 MHz, $CDCl_3$)** δ = 176.70, 161.74 (d, J = 245.1 Hz, 1C), 141.25, 140.94, 138.95, 137.72, 137.19 (d, J = 3.5 Hz, 1C), 130.94, 130.40 (d, J = 7.9 Hz, 2C), 129.01 (2C), 128.85 (2C), 127.14, 127.12 (2C), 127.11 (2C), 114.89 (d, J = 21.0 Hz, 2C), 79.77, 49.41, 43.35, 35.86, 30.85, 27.96 (3C), 25.99 (2C);

(*E*)-7p (major): **^{19}F NMR (565 MHz, $CDCl_3$)** δ = -116.72;

(*Z*)-7p (minor): **^{19}F NMR (565 MHz, $CDCl_3$)** δ = -116.33;

IR (film) 2975, 1716, 1507, 1220, 1131, 731, 697 cm^{-1};

HRMS (DART) calcd for $C_{31}H_{36}O_2F$ $[M+H]^+$ m/z = 459.2699; found 459.2702.

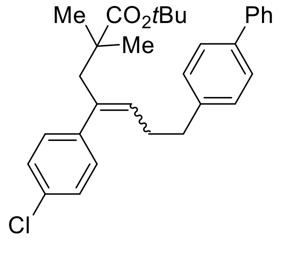

***tert*-butyl 7-([1,1'-biphenyl]-4-yl)-4-(4-chlorophenyl)-2,2-dimethylhept-4-enoate (7q):** Compound **7q** was synthesized following the general procedure 2, using 1-chloro-4-(1-cyclopropylvinyl)benzene (35.7 mg, 0.2 mmol), *tert*-butyl 2-bromo-2-methylpropanoate (245.4 mg, 1.1 mmol) and 4-biphenylmagnesium bromide (3.2 mL, 0.5 M solution in THF, 1.6 mmol). The product **7q** was obtained as a colorless liquid (75.1 mg, 79% yield, 4.2:1 *E*/*Z*) after purified by flash chromatography on silica gel with Hexane/CH_2Cl_2 (2:1).

(*E*)-7q (major): **^{1}H NMR (600 MHz, CDCl$_3$)** δ = 7.64–7.60 (m, 2H), 7.55 (d, *J* = 8.1 Hz, 2H), 7.47–7.43 (m, 2H), 7.35 (td, *J* = 7.2, 2.0 Hz, 1H), 7.29 (d, *J* = 8.0 Hz, 2H), 7.27–7.20 (m, 4H), 5.67 (t, *J* = 7.2 Hz, 1H), 2.79 (t, *J* = 7.8 Hz, 2H), 2.76 (s, 2H), 2.56 (q, *J* = 7.5 Hz, 2H), 1.29 (s, 9H), 0.99 (s, 6H);

(*Z*)-7q (minor): **^{1}H NMR (600 MHz, CDCl$_3$)** δ = 7.59–7.57 (m, 2H), 7.52–7.48 (m, 2H), 7.45–7.42 (m, 2H), 7.35–7.32 (m, 1H), 7.26–7.20 (m, 2H), 7.16 (d, *J* = 8.1 Hz, 2H), 7.00 (d, *J* = 8.4 Hz, 2H), 5.56 (t, *J* = 7.3 Hz, 1H), 2.67 (t, *J* = 7.6 Hz, 2H), 2.61 (s, 2H), 2.28 (q, *J* = 7.5 Hz, 2H), 1.29 (s, 9H), 1.01 (s, 6H);

(*E*)-7q (major): **^{13}C NMR (150 MHz, CDCl$_3$)** δ = 176.92, 143.51, 141.20, 140.93, 139.08, 137.50, 133.26, 132.48, 129.02 (2C), 128.86 (2C), 128.54 (2C), 128.31 (2C), 127.24 (2C), 127.19, 127.13 (2C), 80.02, 43.71, 38.81, 35.62, 31.50, 27.88 (3C), 25.74 (2C);

(*Z*)-7q (minor): **^{13}C NMR (150 MHz, CDCl$_3$)** δ = 176.64, 141.23, 140.84, 139.77, 138.97, 137.58, 132.42, 131.20, 130.27 (2C), 129.02 (2C), 128.85 (2C), 128.21 (2C), 127.14, 127.13 (2C), 127.10 (2C), 79.83, 49.21, 43.38, 35.82, 30.84, 27.93 (3C), 25.99 (2C);

IR (film) 2977, 1715, 1478, 1264, 732, 698 cm^{-1};

HRMS (DART) calcd for $C_{31}H_{39}NO_2Cl$ $[M+H]^+$ *m/z* = 492.2669; found 492.2672.

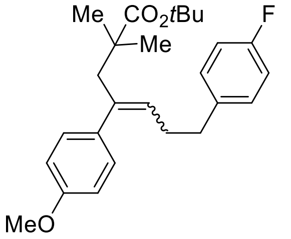

***tert*-butyl 7-(4-fluorophenyl)-4-(4-methoxyphenyl)-2,2-dimethylhept-4-enoate (7r):** Compound **7r** was synthesized following the general procedure 2, using 1-(1-cyclopropylvinyl)-4-methoxybenzene (34.8 mg, 0.2 mmol), *tert*-butyl 2-bromo-2-methylpropanoate (245.4 mg, 1.1 mmol) and 4-fluorophenylmagnesium bromide (1.6 mL, 1.0 M solution in THF, 1.6 mmol). The product **7r** was obtained as a colorless liquid (66 mg, 80% yield, 3.7:1 *E*/*Z*) after purified by flash chromatography on silica gel with Hexane/CH_2Cl_2 (1:1).

(**E)-7r** (major): **^{1}H NMR (600 MHz, $CDCl_3$)** δ = 7.20–7.16 (m, 2H), 7.16–7.12 (m, 2H), 6.97 (t, J = 8.6 Hz, 2H), 6.85–6.74 (m, 2H), 5.55 (t, J = 7.2 Hz, 1H), 3.79 (s, 3H), 2.75–2.65 (m, 4H), 2.47 (q, J = 7.5 Hz, 2H), 1.28 (s, 9H), 0.96 (s, 6H);

(**Z)-7r** (minor): **^{1}H NMR (600 MHz, $CDCl_3$)** δ = 7.04–7.00 (m, 2H), 6.99–6.94 (m, 2H), 6.94–6.89 (m, 2H), 6.85–6.77 (m, 2H), 5.43 (t, J = 7.2 Hz, 1H), 3.79 (s, 3H), 2.62–2.55 (m, 4H), 2.24 (q, J = 7.5 Hz, 2H), 1.28 (s, 9H), 0.98 (s, 6H);

(**E)-7r** (major): **^{13}C NMR (150 MHz, $CDCl_3$)** δ = 177.16, 161.47 (d, J = 243.3 Hz, 1C), 158.72, 138.05, 137.66 (d, J = 3.0 Hz, 1C), 137.62, 131.25, 129.96 (d, J = 8.0 Hz, 2C), 128.17 (2C), 115.16 (d, J = 21.0 Hz, 2C), 113.65 (2C), 79.86, 55.43, 43.71, 38.83, 35.34, 31.61, 27.94 (3C), 25.68 (2C);

(**Z)-7r** (minor): **^{13}C NMR (150 MHz, $CDCl_3$)** δ = 176.86, 161.47 (d, J = 243.3 Hz, 1C), 158.42, 138.33, 137.65, 137.62 (d, J = 3.0 Hz, 1C), 131.25, 129.90 (d, J = 7.8 Hz, 2C), 129.89 (2C), 115.02 (d, J = 20.8 Hz, 2C), 113.49 (2C), 79.66, 55.35, 49.37, 43.40, 35.53, 30.97, 28.00 (3C), 25.93 (2C);

(**E)-7r** (major): **^{19}F NMR (565 MHz, $CDCl_3$)** δ = -117.79;

(**Z)-7r** (minor): **^{19}F NMR (565 MHz, $CDCl_3$)** δ = -118.00;

IR (film) 2974, 2931, 1717, 1508, 1244, 1130, 829 cm^{-1};

HRMS (DART) calcd for $C_{26}H_{34}O_3F$ $[M+H]^+$ m/z = 413.2492; found 413.2489.

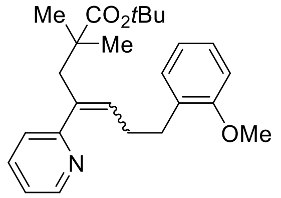

***tert*-butyl 7-(2-methoxyphenyl)-2,2-dimethyl-4-(pyridin-2-yl)hept-4-enoate (7s):** Compound **7s** was synthesized following the general procedure 2, using 2-(1-cyclopropylvinyl)pyridine (29 mg, 0.2 mmol), *tert*-butyl 2-bromo-2-methylpropanoate (245.4 mg, 1.1 mmol) and 2-methoxyphenylmagnesium bromide (1.6 mL, 1.0 M solution in THF, 1.6 mmol). The product **7s** was obtained as a colorless liquid (68.8 mg, 87% yield, 6.4:1 E/Z) after purified by flash chromatography on silica gel with CH_2Cl_2/EtOAc (15:1).

(*E*)-7s (major): **^{1}H NMR (600 MHz, $CDCl_3$)** δ = 8.52 (ddd, *J* = 4.8, 1.9, 0.9 Hz, 1H), 7.59 (td, *J* = 7.7, 1.9 Hz, 1H), 7.33 (dt, *J* = 8.0, 1.1 Hz, 1H), 7.19 (ddd, *J* = 8.1, 7.4, 1.7 Hz, 1H), 7.15 (dd, *J* = 7.4, 1.7 Hz, 1H), 7.09 (ddd, *J* = 7.5, 4.8, 1.1 Hz, 1H), 6.88 (td, *J* = 7.4, 1.1 Hz, 1H), 6.85(dd, *J* = 8.1, 1.1 Hz, 1H), 6.07 (t, *J* = 7.3 Hz, 1H), 3.82 (s, 3H), 3.01 (s, 2H), 2.78–2.70 (m, 2H), 2.54 (q, *J* = 7.6 Hz, 2H), 1.32 (s, 9H), 0.96 (s, 6H);

(*E*)-7s (major): **^{13}C NMR (150 MHz, $CDCl_3$)** δ = 177.31, 162.48, 157.65, 148.55, 137.98, 136.46, 136.34, 130.31, 130.00, 127.34, 121.51, 121.41, 120.52, 110.37, 79.80, 55.30, 43.93, 36.92, 30.51, 30.11, 28.00 (3C), 25.40 (2C);

IR (film) 2974, 1716, 1242, 1131, 749 cm^{-1};

HRMS (ESI) calcd for $C_{25}H_{34}NO_3$ $[M+H]^+$ *m/z* = 396.2539; found 396.2542.

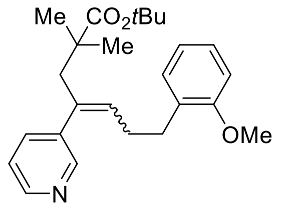

***tert*-butyl 7-(2-methoxyphenyl)-2,2-dimethyl-4-(pyridin-3-yl)hept-4-enoate (7t):** Compound **7t** was synthesized following the general procedure 2, using 3-(1-cyclopropylvinyl)pyridine (29 mg, 0.2 mmol), *tert*-butyl 2-bromo-2-methylpropanoate (245.4 mg, 1.1 mmol) and 2-methoxyphenylmagnesium bromide (1.6 mL, 1.0 M solution in THF, 1.6 mmol). The product **7t** was obtained as a colorless liquid (53 mg, 67% yield, 6.7:1 *E*/*Z*) after purified by flash chromatography on silica gel with CH_2Cl_2/EtOAc (15:1).

(*E*)-7t (major): **^{1}H NMR (600 MHz, $CDCl_3$)** δ = 8.52 (d, *J* = 2.3 Hz, 1H), 8.43 (dd, *J* = 4.9, 1.6 Hz, 1H), 7.58 (ddd, *J* = 7.9, 2.3, 1.6 Hz, 1H), 7.22–7.18 (m, 2H), 7.12 (dd, *J* = 7.4, 1.7 Hz, 1H), 6.89 (td, *J* = 7.4, 1.1 Hz, 1H), 6.85 (dd, *J* = 8.2, 1.1 Hz, 1H), 5.70 (t, *J* = 7.3 Hz, 1H), 3.82 (s, 3H), 2.76 (s, 2H), 2.73 (m, 2H), 2.49 (q, *J* = 7.5 Hz, 2H), 1.22 (s, 9H), 0.99 (s, 6H);

(*Z*)-7t (minor): **^{1}H NMR (600 MHz, $CDCl_3$)** δ = 8.43 (dd, *J* = 4.9, 1.6 Hz, 1H), 8.30–8.27 (m, 1H), 7.33 (dt, *J* = 7.8, 1.9 Hz, 1H), 7.19–7.14 (m, 2H), 7.00 (dd, *J* = 7.4, 1.8 Hz, 1H), 6.85–6.80 (m, 1H), 6.78 (dd, *J* = 8.2, 1.1 Hz, 1H), 5.64 (t, *J* = 7.6 Hz, 1H), 3.71 (s, 3H), 2.65–2.59 (m, 4H), 2.19 (q, *J* = 7.6 Hz, 2H), 1.24 (s, 9H), 0.99 (s, 6H);

(*E*)-**7t** (major): **^{13}C NMR (150 MHz, $CDCl_3$)** δ = 176.80, 157.63, 148.08, 147.34, 140.64, 135.73, 134.91, 134.78, 130.12, 130.01, 127.45, 123.10, 120.54, 110.40, 80.09, 55.32, 43.55, 38.52, 30.49, 29.89, 27.84 (3C), 25.77 (2C);

(*Z*)-**7t** (minor): **^{13}C NMR (150 MHz, $CDCl_3$)** δ = 176.55, 157.54, 149.63, 147.34, 137.15, 136.76, 134.72, 134.67, 133.52, 129.83, 127.34, 123.01, 120.37, 110.30, 79.94, 55.21, 49.02, 43.28, 30.74, 29.17, 27.93 (3C), 26.01 (2C);

IR (film) 2974, 1717, 1242, 1131, 751 cm^{-1};

HRMS (ESI) calcd for $C_{25}H_{34}NO_3$ $[M+H]^+$ *m/z* = 396.2539; found 396.2538.

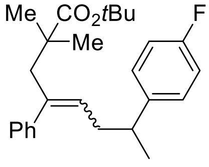

***tert*-butyl 7-(4-fluorophenyl)-2,2-dimethyl-4-phenyloct-4-enoate (7u):** Compound **7u** was synthesized following the general procedure 2, using (1-(*trans*-2-methylcyclopropyl)vinyl)benzene (31.6 mg, 0.2 mmol), *tert*-butyl 2-bromo-2-methylpropanoate (245.4 mg, 1.1 mmol) and 4-fluorophenylmagnesium bromide (1.6 mL, 1.0 M solution in THF, 1.6 mmol). The product **7u** was obtained as a colorless liquid (53.1 mg, 67% yield, 4.6:1 *E*/*Z*) after purified by flash chromatography on silica gel with Hexane/CH_2Cl_2 (2:1).

(*E*)-**7u** (major): **^{1}H NMR (600 MHz, $CDCl_3$)** δ = 7.28–7.18 (m, 5H), 7.18 –7.13 (m, 2H), 6.99–6.95 (m, 2H), 5.52 (t, *J* = 7.2 Hz, 1H), 2.87–2.77 (m, 1H), 2.72 (s, 2H), 2.48–2.37 (m, 2H), 1.28 (d, *J* = 6.9 Hz, 3H), 1.27 (s, 9H), 0.95 (d, *J* = 3.2 Hz, 6H);

(*Z*)-**7u** (minor): **^{1}H NMR (600 MHz, $CDCl_3$)** δ = 7.29–7.18 (m, 2H), 7.17–7.14 (m, 1H), 7.05–7.00 (m, 4H), 6.95–6.90 (m, 2H), 5.36 (t, *J* = 7.3 Hz, 1H), 2.76–2.66 (m, 1H), 2.57 (d, *J* = 2.0 Hz, 2H), 2.24–2.13 (m, 2H), 1.24 (s, 9H), 1.14 (d, *J* = 7.0 Hz, 3H), 0.96 (d, *J* = 3.6 Hz, 6H);

(*E*)-**7u** (major): **^{13}C NMR (150 MHz, $CDCl_3$)** δ = 177.11, 161.42 (d, *J* = 243.4 Hz, 1C), 145.20, 142.69 (d, *J* = 3.2 Hz, 1C), 139.02, 131.71, 128.48 (d, *J* = 7.7 Hz, 2C), 128.17 (2C), 127.20 (2C), 126.67, 115.14 (d, *J* = 20.9 Hz, 2C), 79.88, 43.78, 39.71, 38.79, 38.39, 27.91 (3C), 25.71, 25.66, 21.81;

(*Z*)-7u (minor): **^{13}C NMR (150 MHz, $CDCl_3$)** δ = 176.80, 161.35 (d, *J* = 243.0 Hz, 1C), 142.67 (d, *J* = 3.8 Hz, 1C), 141.52, 139.04, 129.63, 128.95 (2C), 128.48 (d, *J* = 7.7 Hz, 2C), 128.00 (2C), 126.56, 115.00 (d, *J* = 19.6 Hz, 2C), 79.66, 49.39, 43.33, 39.86, 37.67, 27.93 (3C), 25.99, 25.80, 21.96;

(*E*)-7u (major): **^{19}F NMR (565 MHz, $CDCl_3$)** δ = -117.61;

(*Z*)-7u (minor): **^{19}F NMR (565 MHz, $CDCl_3$)** δ = -117.88;

IR (film) 2973, 1717, 1509, 1265, 1133, 832, 733, 699 cm^{-1};

HRMS (DART) calcd for $C_{26}H_{34}O_2F$ $[M+H]^+$ *m/z* = 397.2543; found 397.2550.

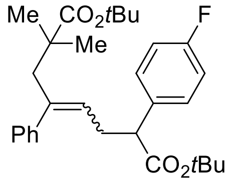

di-*tert*-butyl 7-(4-fluorophenyl)-2,2-dimethyl-4-phenyloct-4-enedioate (7v): Compound **7v** was synthesized following the general procedure 2, using *tert*-butyl (*trans*)-2-(1-phenylvinyl)cyclopropane-1-carboxylate (48.9 mg, 0.2 mmol), *tert*-butyl 2-bromo-2-methylpropanoate (245.4 mg, 1.1 mmol) and 4-fluorophenylmagnesium bromide (1.6 mL, 1.0 M solution in THF, 1.6 mmol). The product **7v** was obtained as a colorless liquid (32.8 mg, 34% yield, 3.3:1 *E*/*Z*) after purified by flash chromatography on silica gel with Hexane/CH_2Cl_2 (1:1).

(*E*)-7v (major): **^{1}H NMR (600 MHz, $CDCl_3$)** δ = 7.31–7.26 (m, 2H), 7.26–7.22 (m, 2H), 7.21–7.16 (m, 3H), 7.02–6.97 (m, 2H), 5.48 (t, *J* = 7.2 Hz, 1H), 3.50 (dd, *J* = 8.3, 7.1 Hz, 1H), 2.91 (ddd, *J* = 14.9, 8.3, 7.5 Hz, 1H), 2.83–2.69 (m, 2H), 2.60–2.54 (m, 1H), 1.38 (s, 9H), 1.25 (s, 9H), 0.97 (s, 3H), 0.96 (s, 3H);

(*Z*)-7v (minor): **^{1}H NMR (600 MHz, $CDCl_3$)** δ = 7.31–7.26 (m, 1H), 7.25–7.22 (m, 1H), 7.21–7.15 (m, 1H), 7.14–7.09 (m, 2H), 7.06–7.03 (m, 2H), 6.96–6.90 (m, 2H), 5.35 (t, *J* = 7.2 Hz, 1H), 3.38 (t, *J* = 7.7 Hz, 1H), 2.63– 2.55 (m, 3H), 2.37 (dt, *J* = 15.0, 7.7 Hz, 1H), 1.35 (s, 9H), 1.23 (s, 9H), 0.95 (s, 3H), 0.95 (s, 3H);

(*E*)-7v (major): **^{13}C NMR (150 MHz, $CDCl_3$)** δ = 176.95, 172.74, 162.14 (d, *J* = 245.1 Hz, 1C), 144.88, 140.04, 135.10 (d, *J* = 3.2 Hz, 1C), 129.84, 129.55 (d, *J* = 7.9 Hz, 2C),

128.19 (2C), 127.19 (2C), 126.83, 115.47 (d, J = 21.4 Hz, 2C), 81.07, 79.93, 52.16, 43.76, 38.92, 33.82, 28.10 (3C), 27.89 (3C), 25.82, 25.67;

(*Z*)-7v (minor): **^{13}C NMR (150 MHz, $CDCl_3$)** δ = 176.71, 172.74, 162.05 (d, J = 245.0 Hz, 1C), 141.00, 140.16, 134.93 (d, J = 3.2 Hz, 1C), 129.57 (d, J = 8.1 Hz, 2C), 128.84 (2C), 128.10, 127.88 (2C), 126.80, 115.29 (d, J = 21.4 Hz, 2C), 80.90, 79.72, 52.35, 49.27, 43.76, 32.95, 28.07 (3C), 27.93 (3C), 25.90, 25.82;

(*E*)-7v (major): **^{19}F NMR (565 MHz, $CDCl_3$)** δ = -115.82;

(*Z*)-7v (minor): **^{19}F NMR (565 MHz, $CDCl_3$)** δ = -116.12;

IR (film) 2977, 2931, 1724, 1509, 1367, 1223, 1141, 849, 699 cm^{-1};

HRMS (DART) calcd for $C_{30}H_{40}O_4F$ $[M+H]^+$ m/z = 483.2911; found 483.2917.

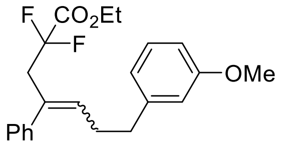

ethyl 2,2-difluoro-7-(3-methoxyphenyl)-4-phenylhept-4-enoate (7x): Compound **7x** was synthesized following the general procedure 2, using (1-cyclopropylvinyl)benzene (28.9 mg, 0.2 mmol), ethyl 2-bromo-2,2-difluoroacetate (223.3 mg, 1.1 mmol) and 3-methoxyphenylmagnesium bromide (1.6 mL, 1.0 M solution in THF, 1.6 mmol). The product **7x** was obtained as a colorless liquid (31.4 mg, 42% yield, 2.3:1 *E*/*Z*) after purified by flash chromatography on silica gel with Hexane/CH_2Cl_2 (1:1).

(*E*)-7x (major): **^{1}H NMR (600 MHz, $CDCl_3$)** δ = 7.33–7.20 (m, 6H), 6.85–6.79 (m, 1H), 6.79–6.74 (m, 2H), 5.92 (t, J = 7.3 Hz, 1H), 3.86 (q, J = 7.2 Hz, 2H), 3.80 (s, 3H), 3.27 (t, J = 15.7 Hz, 2H), 2.75 (t, J = 7.7 Hz, 2H), 2.57 (q, J = 7.6 Hz, 2H), 1.13 (t, J = 7.2 Hz, 3H);

(*Z*)-7x (minor): **^{1}H NMR (600 MHz, $CDCl_3$)** δ = 7.36–7.20 (m, 3H), 7.16 (t, J = 7.9 Hz, 1H), 7.08–7.01 (m, 2H), 6.72 (ddd, J = 8.3, 2.6, 0.9 Hz, 1H), 6.68 (ddd, J = 7.5, 1.6, 0.9 Hz, 1H), 6.62 (t, J = 2.0 Hz, 1H), 5.72 (t, J = 7.3 Hz, 1H), 3.97 (q, J = 7.2 Hz, 2H), 3.76 (s, 3H), 3.16–3.07 (m, 2H), 2.61 (t, J = 7.7 Hz, 2H), 2.29 (q, J = 7.5 Hz, 2H), 1.17 (t, J = 7.2 Hz, 3H);

(*E*)-7x (major): **^{13}C NMR (150 MHz, $CDCl_3$)** δ = 164.00 (t, J = 32.5 Hz, 1C), 159.82, 143.22, 142.26, 135.55, 130.75 (t, J = 4.2 Hz, 1C), 129.52, 128.33 (2C), 127.31, 126.93

(2C), 121.05, 115.30 (t, J = 252.4 Hz, 1C), 114.37, 111.51, 62.79, 55.31, 35.71, 35.56 (t, J = 24.6 Hz, 1C), 31.17, 13.78;

(*Z*)-7x (minor): **^{13}C NMR (150 MHz, $CDCl_3$)** δ = 163.94 (t, J = 33.1 Hz, 1C), 159.73, 143.13, 139.30, 134.20, 131.62 (t, J = 4.8 Hz, 1C), 129.38, 128.72 (2C), 128.19 (2C), 127.24, 120.99, 115.08 (t, J = 251.4 Hz, 1C), 114.23, 111.42, 62.69, 55.25, 44.10 (t, J = 23.7 Hz, 1C), 35.97, 30.89, 13.91;

(*E*)-7x (major): **^{19}F NMR (565 MHz, $CDCl_3$)** δ = -103.18;

(*Z*)-7x (minor): **^{19}F NMR (565 MHz, $CDCl_3$)** δ = -103.55;

IR (film) 2938, 1767, 1600, 1489, 1260, 1092, 697 cm^{-1};

HRMS (DART) calcd for $C_{22}H_{25}O_3F_2$ $[M+H]^+$ m/z = 375.1772; found 375.1778.

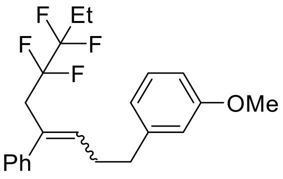

1-methoxy-3-(6,6,7,7-tetrafluoro-4-phenylnon-3-en-1-yl)benzene (7y): Compound **7y** was synthesized following the general procedure 2, using (1-cyclopropylvinyl)benzene (28.9 mg, 0.2 mmol), 1-bromo-1,1,2,2-tetrafluorobutane (229.9 mg, 1.1 mmol) and 3-methoxyphenylmagnesium bromide (1.6 mL, 1.0 M solution in THF, 1.6 mmol). The product **7y** was obtained as a colorless liquid (57.1 mg, 75% yield, 12:1 *E*/*Z*) after purified by flash chromatography on silica gel with Hexane/CH_2Cl_2 (4:1).

(*E*)-7y (major): **^{1}H NMR (600 MHz, $CDCl_3$)** δ = 7.38–7.27 (m, 4H), 7.26–7.17 (m, 2H), 6.85–6.80 (m, 1H), 6.78–6.72 (m, 2H), 6.00 (t, J = 7.4 Hz, 1H), 3.80 (s, 1H), 3.19 (t, J = 18.9 Hz, 2H), 2.75 (dd, J = 8.8, 6.8 Hz, 2H), 2.54 (q, J = 7.6 Hz, 2H), 2.11–1.82 (m, 2H), 1.06 (t, J = 7.5 Hz, 3H);

(*E*)-7y (major): **^{13}C NMR (150 MHz, $CDCl_3$)** δ = 159.85, 143.37, 143.20, 134.73, 130.77, 129.51, 128.37 (2C), 127.06, 126.52 (2C), 121.05, 120.34–117.02 (m, 2C), 114.36, 111.51, 55.29, 35.80, 31.25, 30.36 (t, J = 22.9 Hz, 1C), 23.42 (t, J = 23.6 Hz, 1C), 5.02;

(*E*)-7y (major): **^{19}F NMR (565 MHz, $CDCl_3$)** δ = -111.98 (dt, J = 12.9, 7.1 Hz, 2F), -117.12 (dt, J = 12.8, 7.1 Hz, 2F);

IR (film) 2950, 1600, 1488, 1260, 1167, 1151, 1090, 1001, 695 cm^{-1};

HRMS (DART) calcd for $C_{22}H_{25}OF_4$ $[M+H]^+$ *m/z* = 381.1842; found 381.1847.

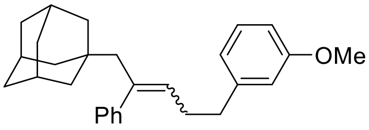

(3*r*,5*r*,7*r*)-1-(5-(3-methoxyphenyl)-2-phenylpent-2-en-1-yl)adamantane (7z): Compound **7z** was synthesized following the general procedure 2, using (1-cyclopropylvinyl)benzene (28.9 mg, 0.2 mmol), 1-bromoadamantane (236.6 mg, 1.1 mmol) and 3-methoxyphenylmagnesium bromide (1.6 mL, 1.0 M solution in THF, 1.6 mmol). The product **7z** was obtained as a colorless liquid (45.5 mg, 59% yield, 6.8:1 *E*/*Z*) after purified by flash chromatography on silica gel with Hexane/CH_2Cl_2 (5:1).

(*E*)-7z (major): **^{1}H NMR (600 MHz, $CDCl_3$)** δ = 7.34–7.25 (m, 4H), 7.24–7.18 (m, 2H), 6.83 (dt, *J* = 7.6, 1.2 Hz, 1H), 6.78 (t, *J* = 2.1 Hz, 1H), 6.75 (ddd, *J* = 8.2, 2.7, 0.9 Hz, 1H), 5.69(t, *J* = 7.1 Hz, 1H), 3.81 (s, 3H), 2.71 (dd, *J* = 9.2, 6.7 Hz, 2H), 2.62–2.41 (m, 2H), 2.33 (s, 2H), 1.81 (s, 2H), 1.62–1.56 (m, 3H), 1.53–1.47 (m, 3H), 1.34 (d, *J* = 2.9 Hz, 6H);

(*E*)-7z (major): **^{13}C NMR (150 MHz, $CDCl_3$)** δ = 159.80, 146.55, 143.96, 138.48, 131.70, 129.43, 128.13 (2C), 126.77 (2C), 126.31, 121.09, 114.45, 111.26, 55.31, 43.96, 43.49 (3C), 37.11 (3C), 36.33, 35.32, 31.81, 28.94 (3C);

IR (film) 2898, 2845, 1600, 1488, 1451, 1260, 1151, 1048, 696 cm^{-1};

HRMS (DART) calcd for $C_{28}H_{35}O$ $[M+H]^+$ *m/z* = 387.2688; found 387.2696.

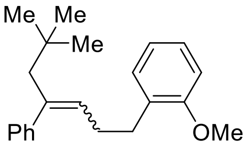

1-(6,6-dimethyl-4-phenylhept-3-en-1-yl)-2-methoxybenzene (7za): Compound **7za** was synthesized following the general procedure 2, using (1-cyclopropylvinyl)benzene (28.9 mg, 0.2 mmol), 2-bromo-2-methylpropane (150.7 mg, 1.1 mmol) and 2-methoxyphenylmagnesium bromide (1.6 mL, 1.0 M solution in THF, 1.6 mmol). The product **7za** was obtained as a colorless liquid (35.2 mg, 57% yield, 3.7:1 *E*/*Z*) after purified by flash chromatography on silica gel with Hexane/CH_2Cl_2 (9:1).

(*E*)-7za (major): **^{1}H NMR (600 MHz, $CDCl_3$)** δ = 7.34–7.24 (m, 4H), 7.22–7.14 (m, 3H), 6.90 (td, *J* = 7.4, 1.1 Hz, 1H), 6.86 (dd, *J* = 8.1, 1.1 Hz, 1H), 5.69 (t, *J* = 7.2 Hz, 1H), 3.84 (s, 3H), 2.90–2.63 (m, 2H), 2.56–2.21 (m, 4H), 0.76 (s, 9H);

(*E*)-7za (major): **^{13}C NMR (150 MHz, $CDCl_3$)** δ = 157.67, 146.42, 139.38, 132.26, 130.65, 130.06, 128.10 (2C), 127.23, 126.94 (2C), 126.24, 120.50, 110.37, 55.34, 42.65, 33.03, 30.75, 30.51 (3C), 30.03;

IR (film) 2949, 2861, 1492, 1241, 1032, 750, 698 cm^{-1};

HRMS (DART) calcd for $C_{22}H_{29}O$ $[M+H]^+$ *m/z* = 309.2218; found 309.2219.

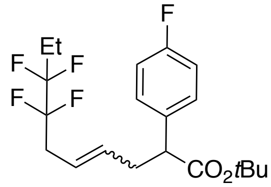

***tert*-butyl 7,7,8,8-tetrafluoro-2-(4-fluorophenyl)dec-4-enoate (7zb):** Compound **7zb** was synthesized following the general procedure 2, using *tert*-Butyl 2-vinylcyclopropane-1-carboxylate **1b-d** (33.6 mg, 0.2 mmol), 1-bromo-1,1,2,2-tetrafluorobutane (229.9 mg, 1.1 mmol) and 4-fluorophenylmagnesium bromide (1.6 mL, 1.0 M solution in THF, 1.6 mmol). The product **7zb** was obtained as a colorless liquid (77.7 mg, 99% yield, 6.8:1 *E*/*Z*) after purified by flash chromatography on silica gel with Hexane/CH_2Cl_2 (5:1) to Hexane/CH_2Cl_2 (1:1);

(*E*)-7zb (major): **^{1}H NMR (600 MHz, $CDCl_3$)** δ = 7.29–7.20 (m, 2H), 7.06–6.94 (m, 2H), 5.59–5.45 (m, 2H), 3.49 (dd, *J* = 8.4, 7.0 Hz, 1H), 2.81–2.72 (m, 1H), 2.71–2.61 (m, 2H), 2.43 (dddd, *J* = 14.5, 7.3, 6.1, 1.2 Hz, 1H), 2.06–1.92 (m, 2H), 1.39 (s, 9H), 1.07 (t, *J* = 7.6 Hz, 3H);

(*E*)-7zb (major): **^{13}C NMR (150 MHz, $CDCl_3$)** δ = 172.49, 162.12 (d, *J* = 245.2 Hz, 1C), 134.89 (d, *J* = 3.3 Hz, 1C), 133.74, 129.90 (d, *J* = 7.8 Hz, 2C), 121.25 (t, *J* = 4.4 Hz, 1C), 121.05–116.53(m, 2C), 115.45 (d, *J* = 21.4 Hz, 2C), 81.09, 51.88, 36.67, 33.97 (t, *J* = 23.4 Hz, 1C), 28.06 (3C), 23.62 (t, *J* = 23.6 Hz, 1C), 4.97 (t, *J* = 5.1 Hz, 1C);

(*E*)-7zb (major): **^{19}F NMR (565 MHz, $CDCl_3$)** δ = -114.51 – -114.60 (m, 2F), -115.88, -117.15 – -117.25 (m, 2F);

IR (film) 2981, 1726, 1509, 1386, 1226, 1146, 1004, 840 cm^{-1};

HRMS (DART) calcd for $C_{20}H_{26}F_5O_2$ $[M+H]^+$ *m/z* = 393.1853; found 393.1844.

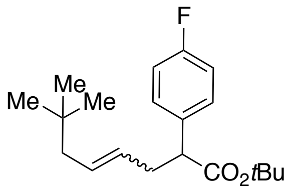

***tert*-butyl 2-(4-fluorophenyl)-7,7-dimethyloct-4-enoate (7zc):** Compound **7zc** was synthesized following the general procedure 2, using *tert*-Butyl 2-vinylcyclopropane-1-carboxylate **1b-d** (33.6 mg, 0.2 mmol), 2-bromo-2-methylpropane (150.7 mg, 1.1 mmol) and 4-fluorophenylmagnesium bromide (1.6 mL, 1.0 M solution in THF, 1.6 mmol). The product **7zc** was obtained as a colorless liquid (49.3 mg, 77% yield, 7.6:1 *E*/*Z*) after purified by flash chromatography on silica gel with Hexane/CH_2Cl_2 (5:1) to Hexane/CH_2Cl_2 (1:1);

(*E*)-7zc (major): **^{1}H NMR (600 MHz, $CDCl_3$)** δ = 7.30–7.18 (m, 2H), 7.05–6.91 (m, 2H), 5.54–5.41 (m, 1H), 5.32–5.22 (m, 1H), 3.47 (t, *J* = 7.7 Hz, 1H), 2.76–2.65 (m, 1H), 2.43–2.36 (m, 1H), 1.84–1.77 (m, 2H), 1.38 (s, 9H), 0.79 (s, 9H);

(*E*)-7zc (major): **^{13}C NMR (150 MHz, $CDCl_3$)** δ = 172.84, 162.05 (d, *J* = 244.9 Hz, 1C), 135.22 (d, *J* = 3.3 Hz, 1C), 130.40, 129.57 (d, *J* = 7.9 Hz, 2C), 128.86, 115.35 (d, *J* = 21.2 Hz, 2C), 80.85, 52.45, 47.20, 36.86, 30.92, 29.31 (3C), 28.12 (3C);

(*E*)-7zc (major): **^{19}F NMR (565 MHz, $CDCl_3$)** δ = -116.18;

IR (film) 2954, 1728, 1509, 1366, 1226, 1141, 972, 838 cm^{-1};

HRMS (DART) calcd for $C_{20}H_{30}FO_2$ $[M+H]^+$ *m/z* = 321.2230; found 321.2237.

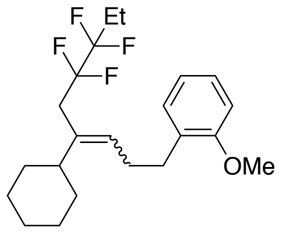

1-(4-cyclohexyl-6,6,7,7-tetrafluoronon-3-en-1-yl)-2-methoxybenzene (7zd): Compound **7zd** was synthesized following the general procedure 2, using (1-cyclopropylvinyl)cyclohexane (30.1 mg, 0.2 mmol), 1-bromo-1,1,2,2-tetrafluorobutane (229.9 mg, 1.1 mmol) and 2-methoxyphenylmagnesium bromide (1.6 mL, 1.0 M solution

in THF, 1.6 mmol). The product **7zd** was obtained as a colorless liquid (72.6 mg, 94% yield, 5.7:1 *E*/*Z*) after purified by flash chromatography on silica gel with Hexane/CH_2Cl_2 (8:1).

(*E*)-7zd (major): **^{1}H NMR (600 MHz, $CDCl_3$)** δ = 7.18 (td, *J* = 7.8, 1.8 Hz, 1H), 7.10 (dd, *J* = 7.3, 1.8 Hz, 1H), 6.87 (td, *J* = 7.4, 1.1 Hz, 1H), 6.84 (dd, *J* = 8.1, 1.1 Hz, 1H), 5.53 (t, *J* = 7.4 Hz, 1H), 3.82 (s, 3H), 2.69 (t, *J* = 19.9 Hz, 2H), 2.63 (dd, *J* = 8.9, 6.7 Hz, 2H), 2.30 (q, *J* = 7.6 Hz, 2H), 2.09–1.94 (m, 2H), 1.90–1.82 (m, 1H), 1.79–1.73 (m, 4H), 1.70–1.64 (m, 1H), 1.25 (qt, *J* = 12.5, 3.5 Hz, 2H), 1.16 (tt, J = 12.9, 3.3 Hz, 1H), 1.11–1.01 (m, 5H);

(*E*)-7zd (major): **^{13}C NMR (150 MHz, $CDCl_3$)** δ = 157.65, 135.31, 130.48, 130.19, 129.10, 127.16, 120.67–117.92 (m, 2C), 120.38, 110.25, 55.27, 45.08, 33.00 (2C), 30.68, 29.90 (t, *J* = 22.8 Hz, 1C), 28.70, 27.14 (2C), 26.60, 23.54 (t, *J* = 23.7 Hz, 1C), 5.09 (t, *J* = 4.8 Hz, 1C);

(*E*)-7zd (major): **^{19}F NMR (565 MHz, $CDCl_3$)** δ = -111.82 – -113.82 (m, 2F), -117.43 (dt, *J* = 12.3, 6.7 Hz, 2F);

IR (film) 2925, 2852, 1493, 1464, 1242, 1169, 1116, 1002, 751 cm^{-1};

HRMS (DART) calcd for $C_{22}H_{31}F_4O$ $[M+H]^+$ *m/z* = 387.2311; found 387.2316.

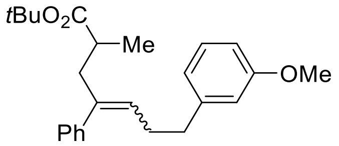

***tert*-butyl 7-(3-methoxyphenyl)-2-methyl-4-phenylhept-4-enoate (7ze):** Compound **7ze** was synthesized following the general procedure 2, using (1-cyclopropylvinyl)benzene (28.9 mg, 0.2 mmol), *tert*-butyl 2-bromopropanoate (230 mg, 1.1 mmol) and 3-methoxyphenylmagnesium bromide (1.6 mL, 1.0 M solution in THF, 1.6 mmol). The product **7ze** was obtained as a colorless liquid (49.5 mg, 65% yield, >20:1 *E*/*Z*) after purified by flash chromatography on silica gel with Hexane/CH_2Cl_2 (2:1).

(*E*)-7ze (major): **^{1}H NMR (600 MHz, $CDCl_3$)** δ = 7.33–7.28 (m, 4H), 7.25–7.19 (m, 2H), 6.85–6.81 (m, 1H), 6.77 (t, *J* = 2.1 Hz, 1H), 6.75 (ddd, *J* = 8.1, 2.6, 0.9 Hz, 1H),

5.72 (t, J = 7.2 Hz, 1H), 3.80 (s, 3H), 2.84 (dd, J = 14.1, 6.3 Hz, 1H), 2.77–2.66 (m, 2H), 2.59–2.45 (m, 3H), 2.32–2.22 (m, 1H), 1.38 (s, 9H), 0.98 (d, J = 7.0 Hz, 3H);

(*E*)-7ze (major): **^{13}C NMR (150 MHz, $CDCl_3$)** δ = 175.92, 159.79, 143.63, 142.77, 138.61, 130.30, 129.44, 128.38 (2C), 126.91, 126.76 (2C), 121.10, 114.39, 111.39, 80.03, 55.31, 39.02, 36.21, 33.38, 30.72, 28.18 (3C), 16.59;

IR (film) 2974, 2934, 1725, 1600, 1489, 1366, 1259, 1151, 697 cm^{-1};

HRMS (DART) calcd for $C_{25}H_{33}O_3$ $[M+H]^+$ m/z = 381.2430; found 381.2429.

9. Determination of Absolute Stereochemistry

Compound **13** had positive rotation, compared to literature values by MacMillan and co-workers was designated *S*.[14] Thus the absolute configuration of compound **3c** was determined as *S*. All other absolute configurations were assigned by analogy based on a uniform reaction mechanism.

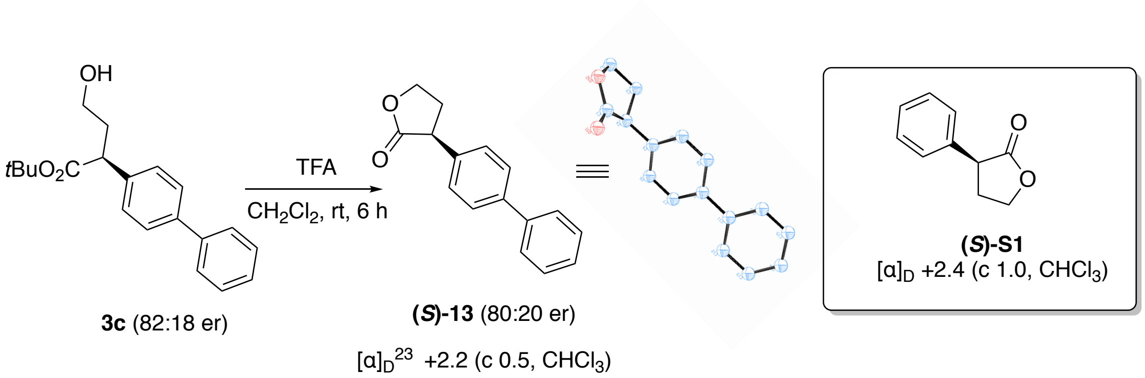

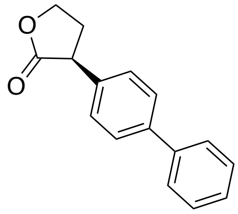

***(S)*-3-([1,1'-biphenyl]-4-yl)dihydrofuran-2(3H)-one (13):** Following a modified procedure by Ghassan et al,[15] a mixture of compound **3c** (43 mg, 0.14 mmol), and trifluoroacetic acid (TFA) (12 μL) was stirred at room temperature in CH_2Cl_2 (4.0 mL) for 6 h. The reaction mixture was diluted with Et_2O (20 mL) and washed with saturated sodium bicarbonate (2 × 5 mL). The organic layer was dried over $MgSO_4$ and concentrated to give compound **13** as a white solid (32 mg, 98 % yield, 80:20 er). The er was determined by HPLC analysis on Daicel Chiralcel AS-H column (Hexane/*i*-PrOH 70:30, 1.0 mL/min, 214 nm; t_r (minor) = 12.93 min, t_r (major)= 13.73 min);

mp: 138-139 °C;

^{1}H NMR (600 MHz, $CDCl_3$) δ = 7.61–7.57 (m, 4H), 7.46–7.43 (m, 2H), 7.39–7.34 (m, 3H), 4.52 (ddd, *J* = 9.1, 8.2, 3.2 Hz, 1H), 4.39 (td, *J* = 9.2, 6.6 Hz, 1H), 3.87 (dd, *J* = 10.4, 9.0 Hz, 1H), 2.79–2.74 (m, 1H), 2.50 (dddd, *J* = 12.9, 10.4, 9.4, 8.2 Hz, 1H);

^{13}C NMR (150 MHz, CDCl$_3$) δ = 177.44, 140.86, 140.73, 135.71, 128.95 (2C), 128.47 (2C), 127.85 (2C), 127.58, 127.25(2C), 66.67, 45.37, 31.73;

IR (film) 2922, 1753, 1488, 1371, 1149, 1116, 947, 846, 762, 692 cm^{-1};

HRMS (ESI) calcd for $C_{16}H_{14}O_2Na$ $[M+Na]^+$ *m/z* = 261.0892; found 261.0898;

[α]$_D^{23}$ +2.2 (c 0.5, CHCl$_3$).

10. Iron-catalyzed Enantioselective Intermolecular Difunctionalization

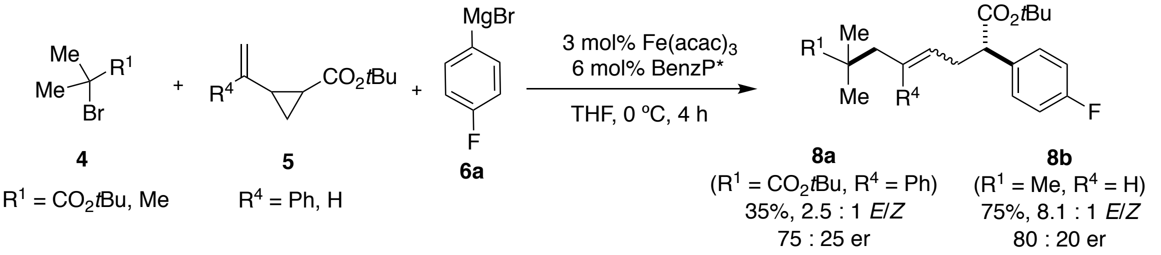

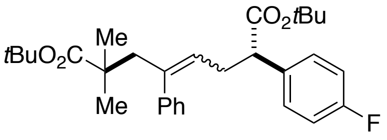

di-*tert*-butyl-7-(4-fluorophenyl)-2,2-dimethyl-4-phenyloct-4-enedioate (8a): Compound **8a** was synthesized following the general procedure 2, using Fe(acac)$_3$ (2.1 mg, 3 mol%), (*R*,*R*)-BenzP* (3.4 mg, 6 mol%), *tert*-butyl (*trans*)-2-(1-phenylvinyl)cyclopropane-1-carboxylate (48.9 mg, 0.2 mmol), *tert*-butyl 2-bromo-2-methylpropanoate **4a** (245.4 mg, 1.1 mmol) and 4-fluorophenylmagnesium bromide **6a** (1.6 mL, 1.0 M solution in THF, 1.6 mmol). The product **8a** was obtained as a colorless liquid (33.8 mg, 34% yield, 2.5:1 *E*/*Z*) after purified by flash chromatography on silica gel with Hexane/CH$_2$Cl$_2$ (1:1). The er of (*E*)-**8a** was determined by HPLC analysis on Daicel Chiralcel AD-H column (75:25 er, Hexane/*i*-PrOH 99.5:0.5, 0.7 mL/min, 254 nm; t_r (minor) = 6.47 min, t_r (major)= 6.74 min);

(*E*)-8a (major): **^{1}H NMR (600 MHz, CDCl$_3$)** δ = 7.30–7.26 (m, 2H), 7.26–7.22 (m, 2H), 7.21–7.16 (m, 3H), 7.04–6.96 (m, 2H), 5.48 (t, *J* = 7.2 Hz, 1H), 3.50 (dd, *J* = 8.4, 7.0 Hz, 1H), 2.90 (ddd, *J* = 14.9, 8.3, 7.5 Hz, 1H), 2.83–2.68 (m, 2H), 2.56 (dt, *J* = 14.6, 7.0 Hz, 1H), 1.38 (s, 9H), 1.25 (s, 9H), 0.97 (s, 3H), 0.96 (s, 3H);

(*E*)-8a (major): **^{13}C NMR (150 MHz, $CDCl_3$)** δ = 176.96, 172.74, 162.14 (d, *J* = 245.3 Hz, 1C), 144.88, 140.04, 135.10 (d, *J* = 2.8 Hz, 1C), 129.84, 129.55 (d, *J* = 7.9 Hz, 2C), 128.20 (2C), 127.19 (2C), 126.83, 115.47 (d, *J* = 21.4 Hz, 2C), 81.08, 79.93, 52.16, 43.76, 38.92, 33.81, 28.11 (3C), 27.89 (3C), 25.82, 25.67;

(*E*)-8a (major): **^{19}F NMR (565 MHz, $CDCl_3$)** δ = -115.83;

(*E*)-8a (major): **IR (film)** 2976, 2931, 1722, 1509, 1367, 1223, 1141, 848, 699 cm^{-1};

(*E*)-8a (major): **HRMS (DART)** calcd for $C_{30}H_{40}O_4F$ $[M+H]^+$ *m/z* = 483.2911; found 483.2913.

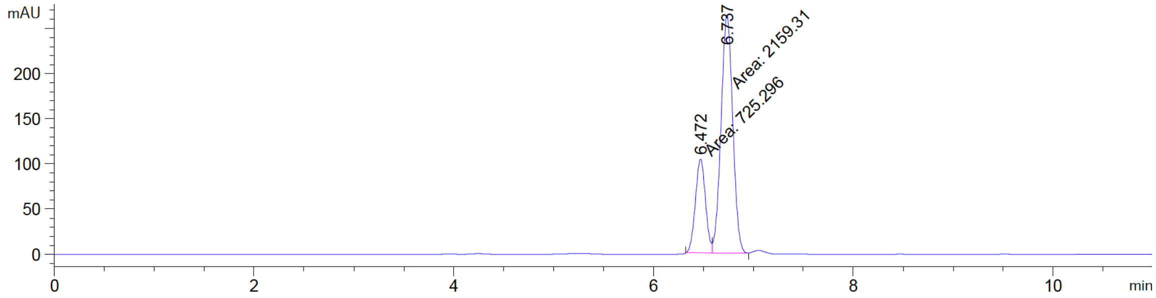

Signal 1: VWD1 A, Wavelength=254 nm

Peak #	RetTime [min]	Type	Width [min]	Area [mAU*s]	Height [mAU]	Area %
1	6.472	MF	0.1163	725.29602	103.93562	25.1437
2	6.737	FM	0.1371	2159.30933	262.53864	74.8563
Totals :				2884.60535	366.47425	

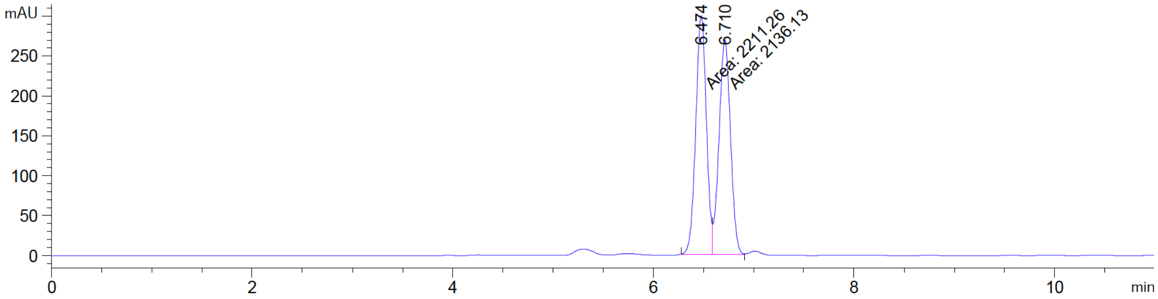

Signal 1: VWD1 A, Wavelength=254 nm

Peak #	RetTime [min]	Type	Width [min]	Area [mAU*s]	Height [mAU]	Area %
1	6.474	MF	0.1230	2211.25757	299.56464	50.8641
2	6.710	FM	0.1324	2136.12549	268.99951	49.1359
Totals :				4347.38306	568.56415	

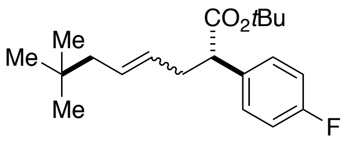

***tert*-butyl 2-(4-fluorophenyl)-7,7-dimethyloct-4-enoate (8b):** Compound **8b** was synthesized following the general procedure 2, using Fe(acac)$_3$ (2.1 mg, 3 mol%), (*R*,*R*)-BenzP* (3.4 mg, 6 mol%), *tert*-Butyl 2-vinylcyclopropane-1-carboxylate **1b-d** (33.6 mg, 0.2 mmol), 2-bromo-2-methylpropane (150.7 mg, 1.1 mmol) and 4-fluorophenylmagnesium bromide (1.6 mL, 1.0 M solution in THF, 1.6 mmol). The product **8b** was obtained as a colorless liquid (40.1 mg, 75% yield, 8.1:1 *E*/*Z*) after purified by flash chromatography on silica gel with Hexane/CH_2Cl_2 (5:1) to Hexane/CH_2Cl_2 (1:1). The er of (*E*)-**8b** was determined by HPLC analysis on Daicel Chiralcel AD-H column (80:20 er, Hexane/*i*-PrOH 99.8:0.2, 0.7 mL/min, 214 nm; t_r (minor) = 7.84 min, t_r (major)= 8.27 min);

(*E*)-8b (major): **^{1}H NMR (600 MHz, $CDCl_3$)** δ = 7.28–7.22 (m, 2H), 7.02–6.95 (m, 2H), 5.53–5.42 (m, 1H), 5.29–5.23 (m, 1H), 3.47 (t, *J* = 7.7 Hz, 1H), 2.75–2.65 (m, 1H), 2.43–2.36 (m, 1H), 1.87–1.69 (m, 2H), 1.38 (s, 9H), 0.79 (s, 9H);

(*E*)-8b (major): **^{13}C NMR (150 MHz, $CDCl_3$)** δ = 172.85, 162.05 (d, *J* = 244.9 Hz, 1C), 135.22 (d, *J* = 3.3 Hz, 1C), 130.41, 129.57 (d, *J* = 7.8 Hz, 2C), 128.86, 115.35 (d, *J* = 21.3 Hz, 2C), 80.86, 52.45, 47.20, 36.86, 30.92, 29.31 (3C), 28.13 (3C). Spectral data matched the racemic compound **7zc** (*vide supra*).

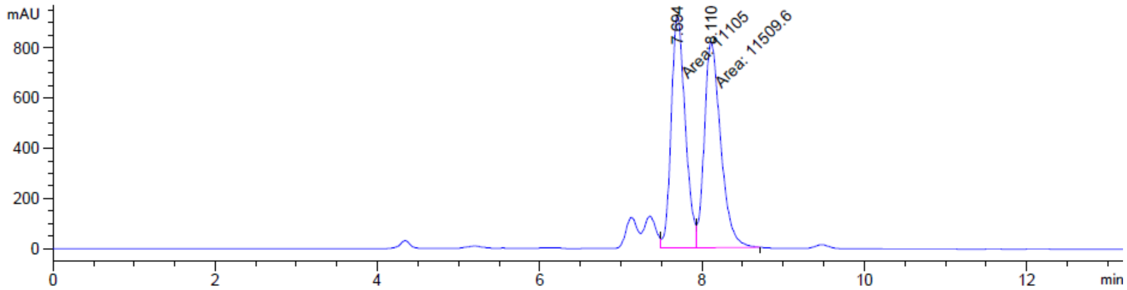

Signal 2: VWD1 B, Wavelength=214 nm

Peak #	RetTime [min]	Type	Width [min]	Area [mAU*s]	Height [mAU]	Area %
1	7.694	MF	0.2011	1.11050e4	920.52136	49.1054
2	8.110	FM	0.2346	1.15096e4	817.83044	50.8946

Totals : 2.26146e4 1738.35181

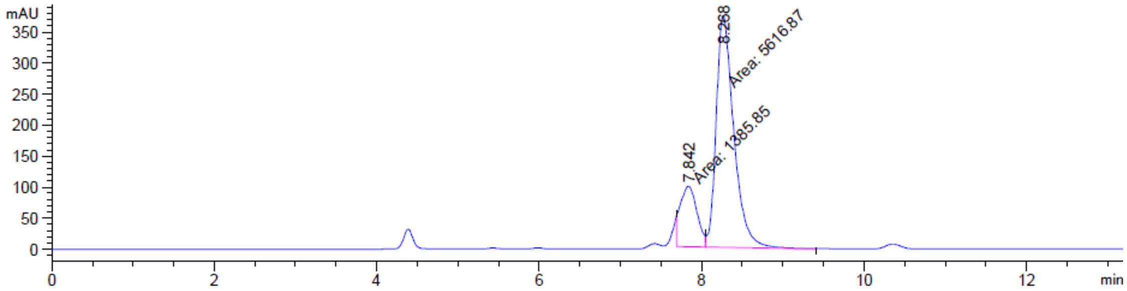

Signal 2: VWD1 B, Wavelength=214 nm

Peak #	RetTime [min]	Type	Width [min]	Area [mAU*s]	Height [mAU]	Area %
1	7.842	FM	0.2379	1385.84888	97.07858	19.7902
2	8.268	FM	0.2513	5616.86719	372.53925	80.2098

Totals : 7002.71606 469.61782

11. Derivatizations of 7k

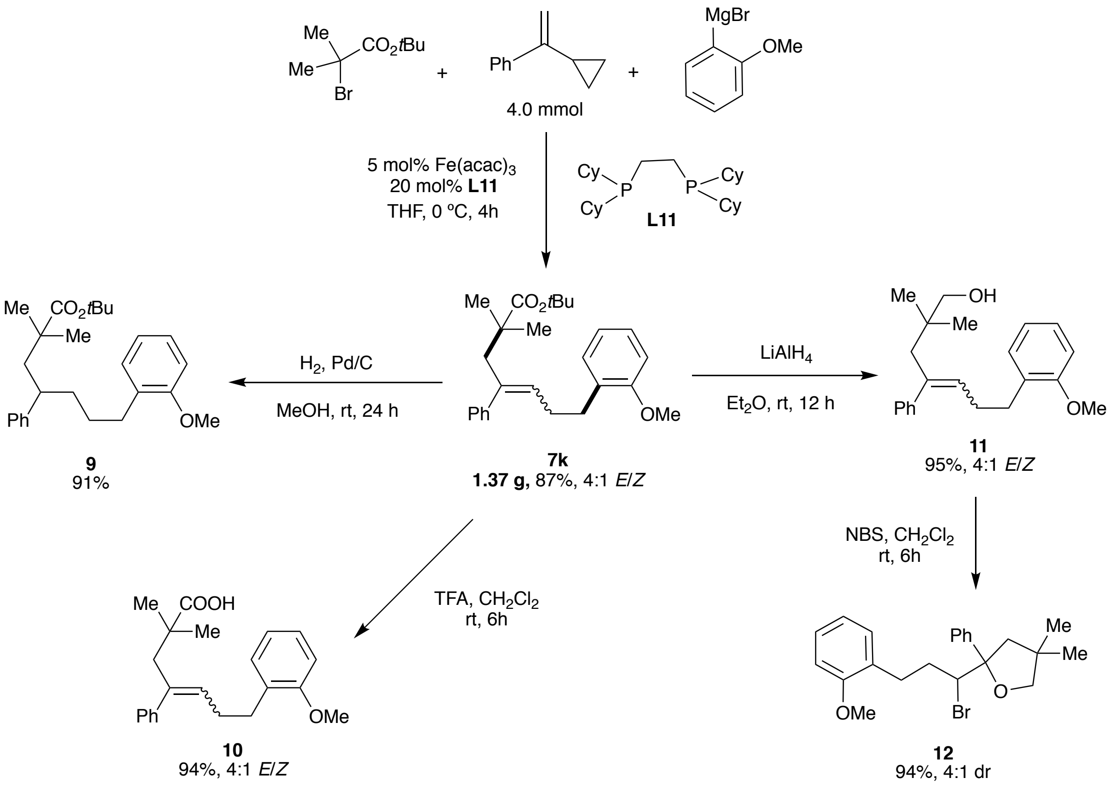

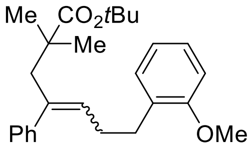

***tert*-butyl 7-(2-methoxyphenyl)-2,2-dimethyl-4-phenylhept-4-enoate (7k)-gram-scale :** Compound **7k** was synthesized following the general procedure 2, using Fe(acac)$_3$ (70.7 mg, 5 mol%), 1,2-bis(dicyclohexylphosphanyl)ethane **L11** (338 mg, 20 mol%), (1-cyclopropylvinyl)benzene (577 mg, 4.0 mmol), *tert*-butyl 2-bromo-2-methylpropanoate (4.91 g, 22 mmol), 2-methoxyphenylmagnesium bromide (32 mL, 1.0 M solution in THF, 32 mmol) and THF (4 mL). The product **7k** was obtained as a colorless liquid (1.37 g, 87% yield, 4:1 *E*/*Z*) after purified by flash chromatography on silica gel with Hexane/CH_2Cl_2 (2:1).

(*E*)-7k (major): **^{1}H NMR (600 MHz, CDCl$_3$)** δ = 7.30–7.21 (m, 4H), 7.20–7.14 (m, 2H), 7.13 (dd, *J* = 7.4, 1.7 Hz, 1H), 6.87 (td, *J* = 7.3, 1.1 Hz, 1H), 6.83 (dd, *J* = 8.2, 1.2 Hz, 1H), 5.65 (t, *J* = 7.3 Hz, 1H), 3.81 (s, 3H), 2.77 (s, 2H), 2.71 (dd, *J* = 9.1, 6.5 Hz, 2H),

2.46 (q, J = 7.6 Hz, 2H), 1.24 (s, 9H), 0.96 (s, 6H). Spectral data matched the small scale **7k** (*vide supra*).

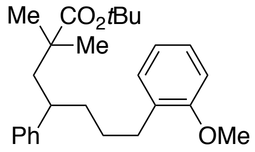

***tert*-butyl 7-(2-methoxyphenyl)-2,2-dimethyl-4-phenylheptanoate (9):** To an oven-dried 5 mL microwave vial equipped with a stir bar, 10% Pd/C (9.9 mg, 10 wt % of **7k**) was added into a MeOH (0.5 mL) solution of **7k** (98.6 mg, 0.25 mmol). After two vacuum/H_2 cycles to replace air inside the reaction tube with hydrogen, the reaction mixture was vigorously stirred at room temperature under ordinary hydrogen pressure (balloon) for 24 h. The reaction mixture was filtered using Celite. The product **9** was obtained as a colorless liquid (90.2 mg, 91% yield) after purified by flash chromatography on silica gel with Hexane/CH_2Cl_2 (1:1);

^{1}H NMR (600 MHz, $CDCl_3$) δ = 7.31–7.21 (m, 2H), 7.18–7.11 (m, 4H), 7.02 (dd, J = 7.4, 1.7 Hz, 1H), 6.84 (td, J = 7.4, 1.1 Hz, 1H), 6.80 (dd, J = 8.2, 1.1 Hz, 1H), 3.76 (s, 3H), 2.67–2.54 (m, 2H), 2.53–2.44 (m, 1H), 2.00–1.82 (m, 2H), 1.73–1.62 (m, 1H), 1.61–1.53 (m, 1H), 1.45–1.39 (m, 1H), 1.37 (s, 9H), 1.36 – 1.29 (m, 1H), 1.07 (s, 3H), 0.95 (s, 3H);

^{13}C NMR (150 MHz, $CDCl_3$) δ = 177.36, 157.50, 146.79, 131.05, 129.78, 128.29 (2C), 128.11 (2C), 126.88, 125.90, 120.37, 110.31, 79.75, 55.28, 47.42, 43.16, 43.11, 38.87, 30.16, 28.04 (3C), 27.78, 27.11, 25.33;

IR (film) 2929, 1719, 1492, 1455, 1366, 1241, 1131, 1032, 851, 751, 701 cm^{-1};

HRMS (DART) calcd for $C_{26}H_{37}O_3$ $[M+H]^+$ m/z = 397.2743; found 397.2751.

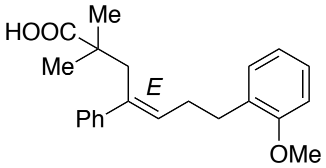

(*E*)-7-(2-methoxyphenyl)-2,2-dimethyl-4-phenylhept-4-enoic acid (*E*)-10: To an oven-dried 5 mL microwave vial equipped with a stir bar, trifluoroacetic acid (191 μL, 2.5 mmol) was added into a CH_2Cl_2 (1.0 mL) solution of **7k** (98.6 mg, 0.25 mmol) at room

temperature, and the mixture was stirred for 6 h. Then the reaction mixture was diluted with CH_2Cl_2 (5.0 mL) and washed with 10% $NaHCO_3$ (2 × 3 mL). The aqueous layer was extracted with CH_2Cl_2 (4 × 5 mL). The organic layers were combined and dried over $MgSO_4$. The purity major product **(*E*)-10** was obtained as a colorless liquid 55 mg in 65% yield (all isomers in 94% yield, 4:1 *E*/*Z*) after purified by Isolera™ Flash Systems silica gel chromatography with prepacked silica-gel cartridges (SNAP Ultra; Biotage) and a gradient elution Hexene/EtOAc (90:10) to Hexene/EtOAc (55:45);

(*E*)-10 (major): **^{1}H NMR (600 MHz, $CDCl_3$)** δ = 11.21 (bs, 1H), 7.30–7.22 (m, 4H), 7.20 (td, *J* = 7.8, 1.8 Hz, 1H), 7.18–7.13 (m, 2H), 6.90 (td, *J* = 7.4, 1.1 Hz, 1H), 6.87 (dd, *J* = 8.2, 1.1 Hz, 1H), 5.73 (t, *J* = 7.3 Hz, 1H), 3.84 (s, 3H), 2.82 (s, 2H), 2.73 (dd, *J* = 9.1, 6.6 Hz, 2H), 2.48 (q, *J* = 7.5 Hz, 2H), 1.02 (s, 6H);

(*E*)-10 (major): **^{13}C NMR (150 MHz, $CDCl_3$)** δ = 183.84, 157.61, 144.64, 137.39, 133.57, 130.35, 130.12, 128.03 (2C), 127.33, 127.25 (2C), 126.66, 120.58, 110.47, 55.39, 42.93, 39.02, 30.68, 29.77, 25.07 (2C).

(*E*)-10 (major): **IR (film)** 2928, 1697, 1493, 1466, 1242, 1032, 752, 700 cm^{-1};

(*E*)-10 (major): **HRMS (DART)** calcd for $C_{22}H_{27}O_3$ $[M+H]^+$ *m/z* = 339.1960; found 339.1951.

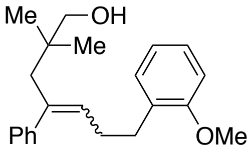

7-(2-methoxyphenyl)-2,2-dimethyl-4-phenylhept-4-en-1-ol (11): To an oven-dried 5 mL microwave vial equipped with a stir bar, $LiAlH_4$ (12 mg, 0.3 mmol) and anhydrous Et_2O (0.5 mL) was added followed by a solution of **7k** (98.6 mg, 0.25 mmol) in anhydrous Et_2O (1.0 mL) at 0 °C . The reaction mixture was stirred for 12 h at room temperature. Then the reaction mixture was quenched by sat. NH_4Cl (3 mL). The aqueous layer was extracted with Et_2O (3 × 5 mL). The organic layers were combined and dried over $MgSO_4$. The product **11** was obtained as a colorless liquid (76.9 mg, 95% yield, 4:1 *E*/*Z*) after purified by flash chromatography on silica gel with Hexane/EtOAc (3:1);

(*E*)-11 (major): **^{1}H NMR (600 MHz, $CDCl_3$)** δ = 7.36–7.27 (m, 4H), 7.25–7.19 (m, 2H), 7.17 (dd, *J* = 7.4, 1.8 Hz, 1H), 6.91 (td, *J* = 7.4, 1.1 Hz, 1H), 6.88 (dd, *J* = 8.2, 1.1 Hz,

1H), 5.72 (t, J = 7.2 Hz, 1H), 3.85 (s, 3H), 3.10 (s, 2H), 2.82–2.72 (m, 2H), 2.52 (s, 2H), 2.52–2.45 (m, 2H), 1.22 (bs, 1H), 0.78 (s, 6H);

(*Z*)-11 (minor): **^{1}H NMR (600 MHz, $CDCl_3$)** δ = 7.36–7.27 (m, 2H), 7.25–7.19 (m, 1H), 7.18 (dd, J = 5.9, 1.7 Hz, 1H), 7.13–7.09 (m, 2H), 7.06 (dd, J = 7.4, 1.7 Hz, 1H), 6.89–6.83 (m, 1H), 6.82 (dd, J = 8.2, 1.0 Hz, 1H), 5.55 (t, J = 7.5 Hz, 1H), 3.76 (s, 3H), 3.10 (s, 2H), 2.69 (dd, J = 8.4, 6.7 Hz, 2H), 2.39 (s, 2H), 2.30 (q, J = 7.6 Hz, 2H), 1.64 (bs, 1H), 0.74 (s, 6H);

(*E*)-11 (major): **^{13}C NMR (150 MHz, $CDCl_3$)** δ = 157.60, 146.07, 138.53, 132.99, 130.42, 130.11, 128.40 (2C), 127.30, 126.74 (2C), 126.63, 120.54, 110.40, 71.63, 55.36, 37.64, 37.62, 30.71, 29.94, 24.97 (2C);

(*Z*)-11 (minor): **^{13}C NMR (150 MHz, $CDCl_3$)** δ = 157.57, 142.11, 138.46, 131.12, 130.32, 130.10, 128.68 (2C), 128.10 (2C), 127.16, 126.54, 120.37, 110.30, 71.38, 55.28, 47.85, 36.81, 30.82, 29.26, 24.92 (2C).

IR (film) 3402, 2954, 2867, 1600, 1492, 1464, 1242, 1178, 1119, 1032, 751, 700 cm^{-1};

HRMS (DART) calcd for $C_{22}H_{29}O_2$ $[M+H]^+$ m/z = 325.2168; found 325.2178.

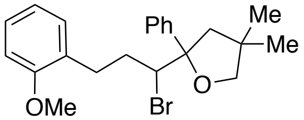

2-(1-bromo-3-(2-methoxyphenyl)propyl)-4,4-dimethyl-2-phenyltetrahydrofuran (12): A oven-dried 50 mL flask with argon inlet and Teflon cap was charged with a suspension of NBS (49.8 mg, 0.28 mmol) in CH_2Cl_2 (4 mL). The suspension was cooled to 0 °C, and a CH_2Cl_2 (2 mL) solution of **11** (64.8 mg, 0.2 mmol) was added dropwise. The reaction mixture was stirred for 0.5 h at 0 °C and for 6 h at room temperature. After the reaction completed, diluted with Et_2O (5 mL) and washed with water (10 mL). The aqueous layer was extracted with Et_2O (3 × 5 mL). The organic layers were combined and dried over Na_2SO_4. The product **12** was obtained as a colorless liquid (75.7 mg, 94% yield, 4:1 dr) after purified by flash chromatography on silica gel with Hexane/EtOAc (10:1). The major product of **12** was obtained as a colorless liquid (58 mg, 72% yield) after purified by flash chromatography on silica gel with Hexane/CH_2Cl_2 (2:1);

12 (major): **^{1}H NMR (600 MHz, $CDCl_3$)** δ = 7.38–7.31 (m, 2H), 7.31–7.25 (m, 2H), 7.25–7.19 (m, 1H), 7.16 (td, *J* = 7.8, 1.8 Hz, 1H), 7.06 (dd, *J* = 7.3, 1.7 Hz, 1H), 6.84 (td, *J* = 7.4, 1.1 Hz, 1H), 6.75 (dd, *J* = 8.2, 1.0 Hz, 1H), 4.09 (dd, *J* = 11.1, 1.9 Hz, 1H), 3.84 (d, *J* = 8.2 Hz, 1H), 3.63 (s, 3H), 3.62 (d, *J* = 8.1 Hz, 1H), 2.89 (ddd, *J* = 13.1, 8.2, 4.5 Hz, 1H), 2.59 (dt, *J* = 13.6, 8.0 Hz, 1H), 2.42 (d, *J* = 12.7 Hz, 1H), 2.20 (d, *J* = 12.7 Hz, 1H), 1.99 (dtd, *J* = 14.7, 8.1, 1.9 Hz, 1H), 1.88 (dddd, *J* = 14.8, 11.1, 8.2, 4.5 Hz, 1H), 1.12 (s, 3H), 0.79 (s, 3H);

12 (major): **^{13}C NMR (150 MHz, $CDCl_3$)** δ = 157.60, 144.08, 130.41, 129.21, 127.85 (2C), 127.39, 126.91, 126.64 (2C), 120.36, 110.27, 89.41, 81.05, 66.81, 55.13, 52.51, 40.26, 33.50, 29.34, 27.55, 27.25;

12 (major): **IR (film)** 2955, 2869, 1493, 1463, 1242, 1060, 1032, 753, 706 cm^{-1};

12 (major): **HRMS (DART)** calcd for $C_{22}H_{28}BrO_2$ $[M+H]^+$ *m/z* = 403.1273; found 403.1276.

12. Spectral Data

Compound 1a-b. Top: ^{1}H NMR ($CDCl_3$, 600 MHz). Bottom: ^{13}C NMR ($CDCl_3$, 150 MHz)

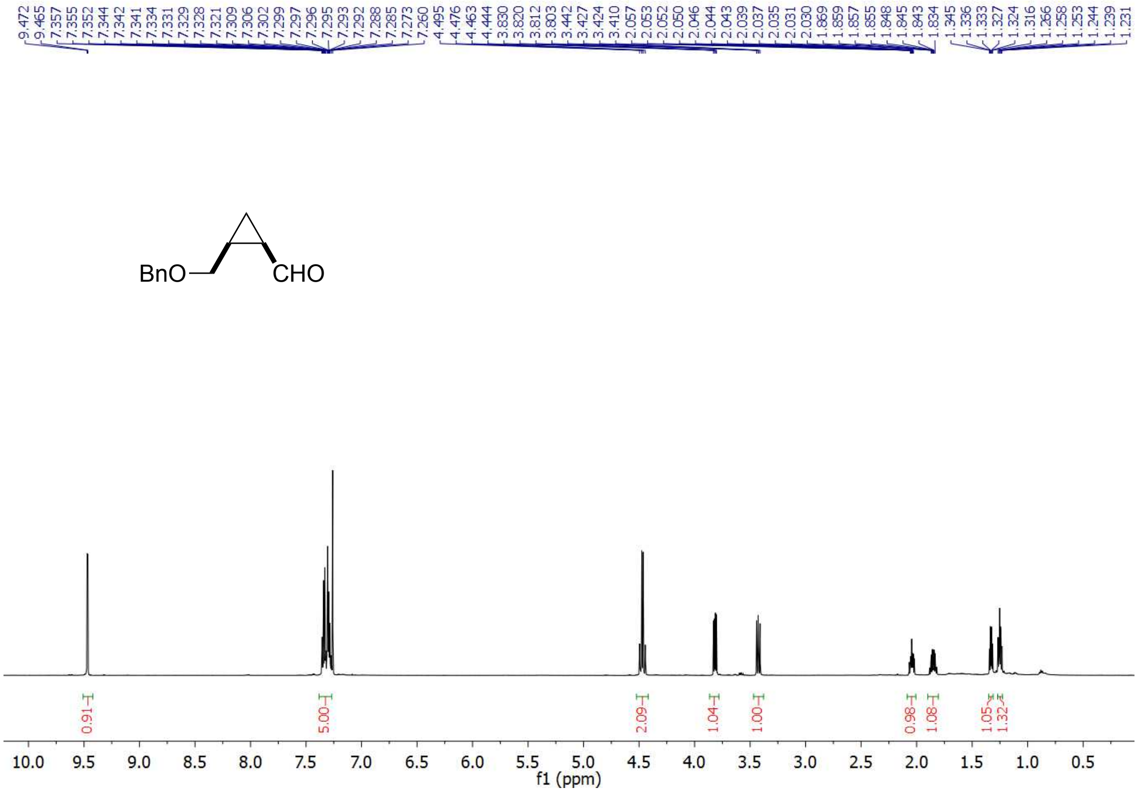

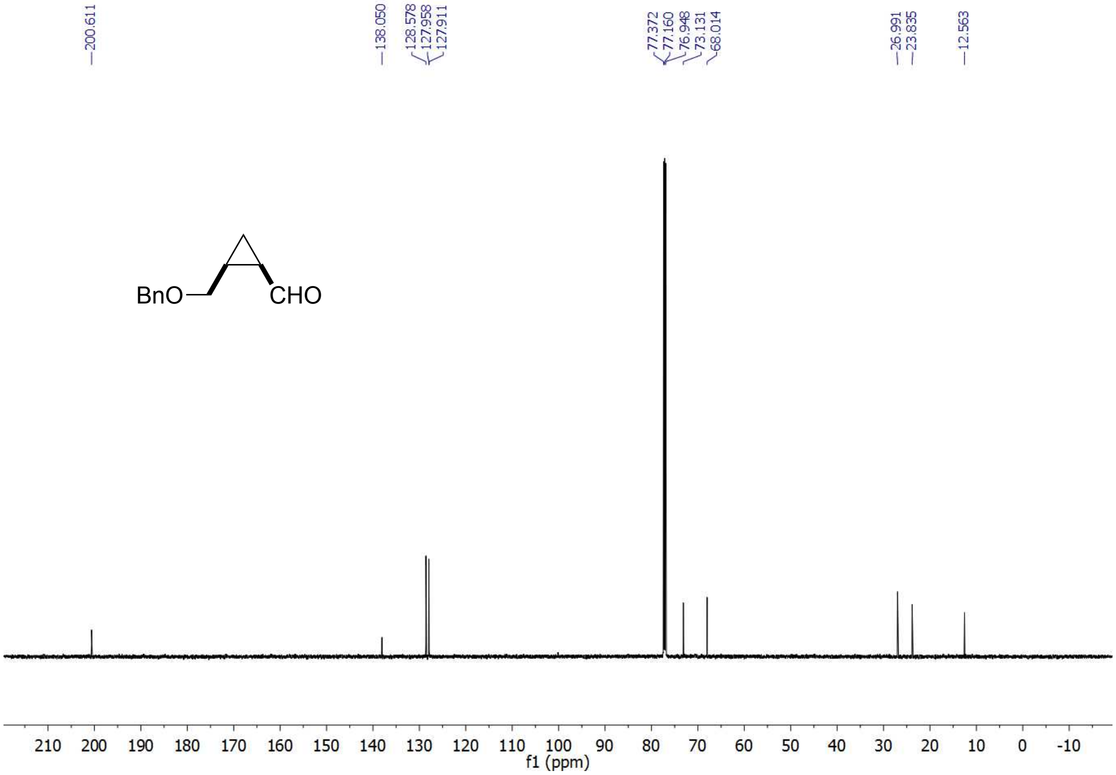

Compound 1a-c. Top: ^{1}H NMR ($CDCl_3$, 600 MHz). Bottom: ^{13}C NMR ($CDCl_3$, 150 MHz)

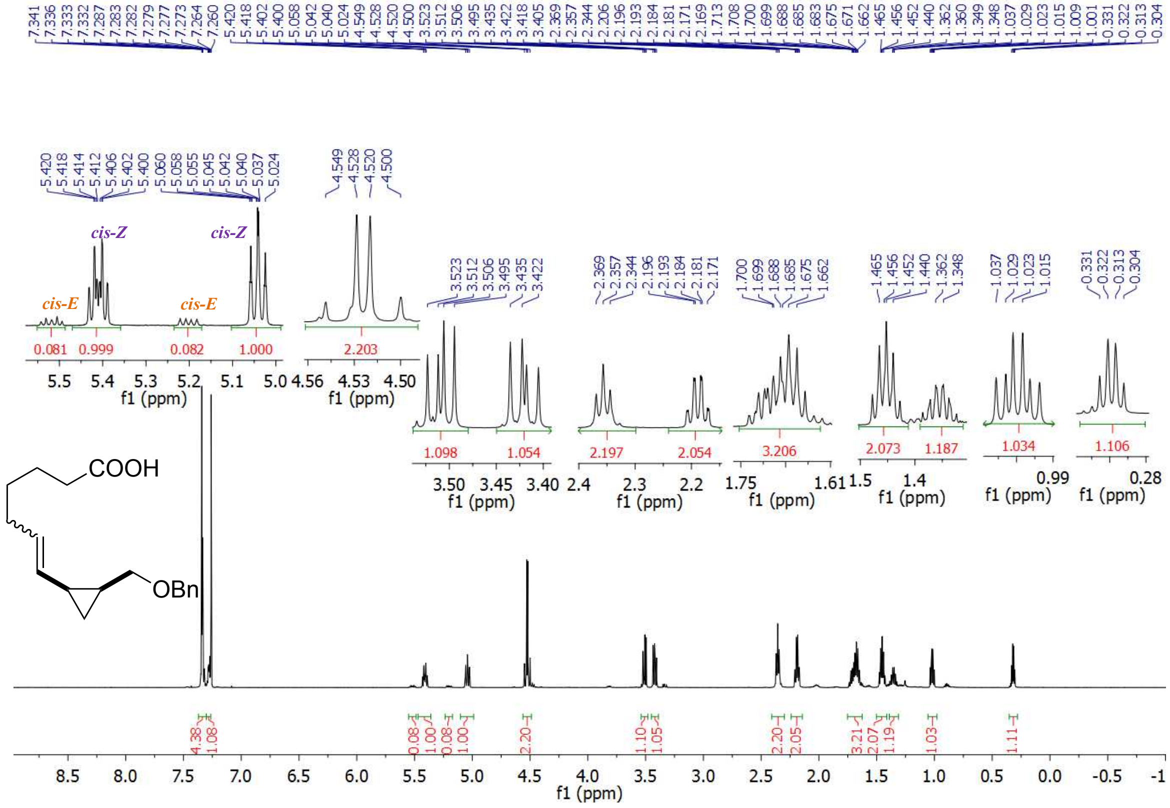

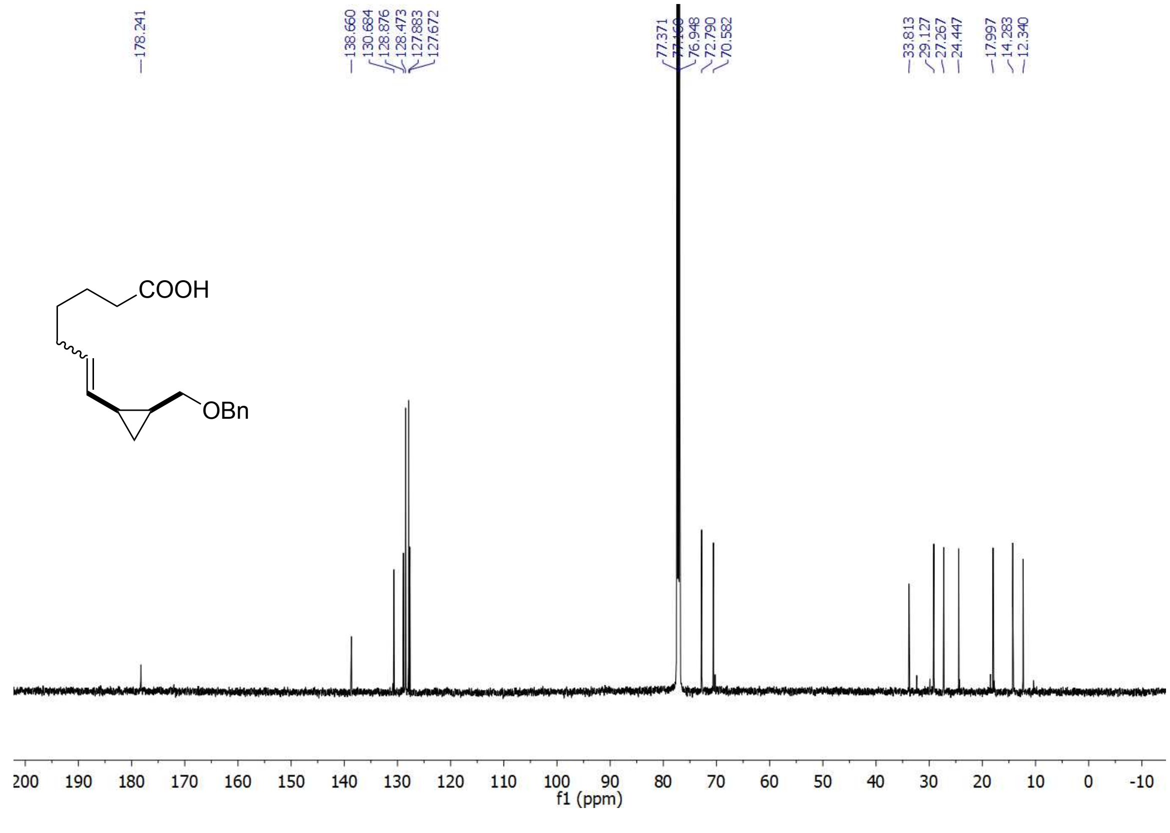

Compound 1a-d. Top: ^{1}H NMR ($CDCl_3$, 600 MHz). Bottom: ^{13}C NMR ($CDCl_3$, 150 MHz)

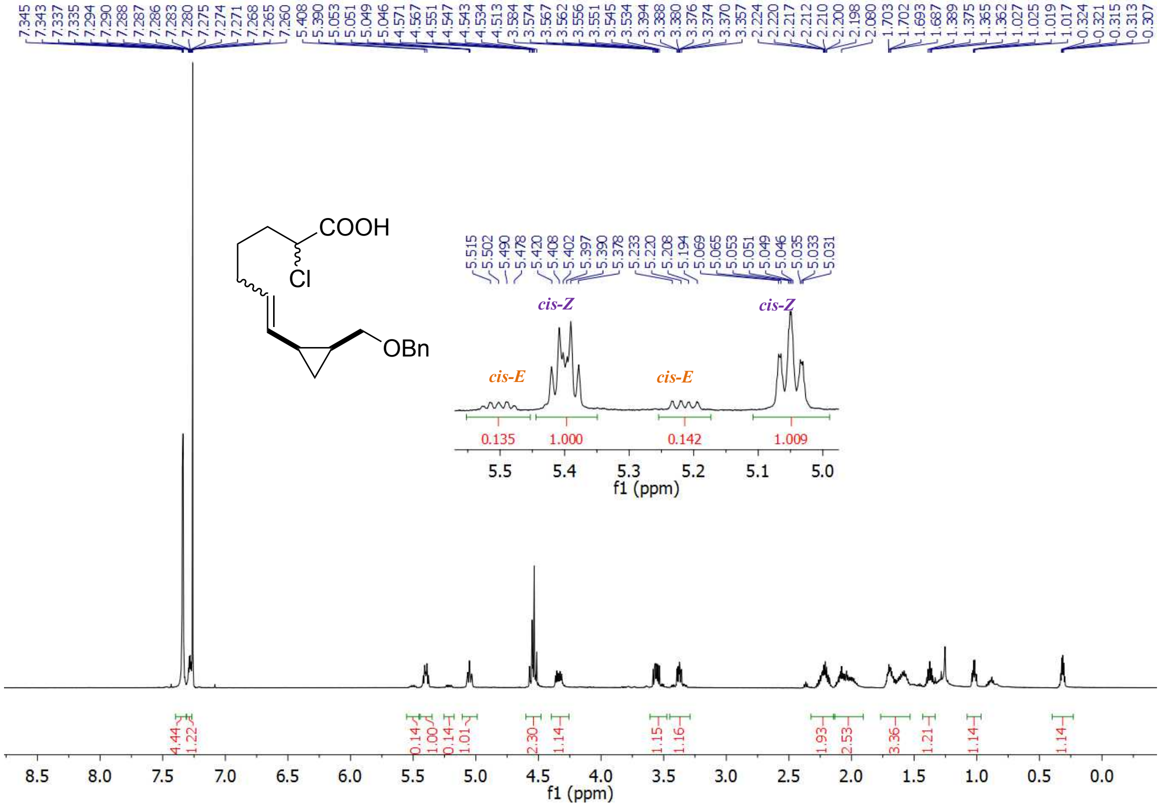

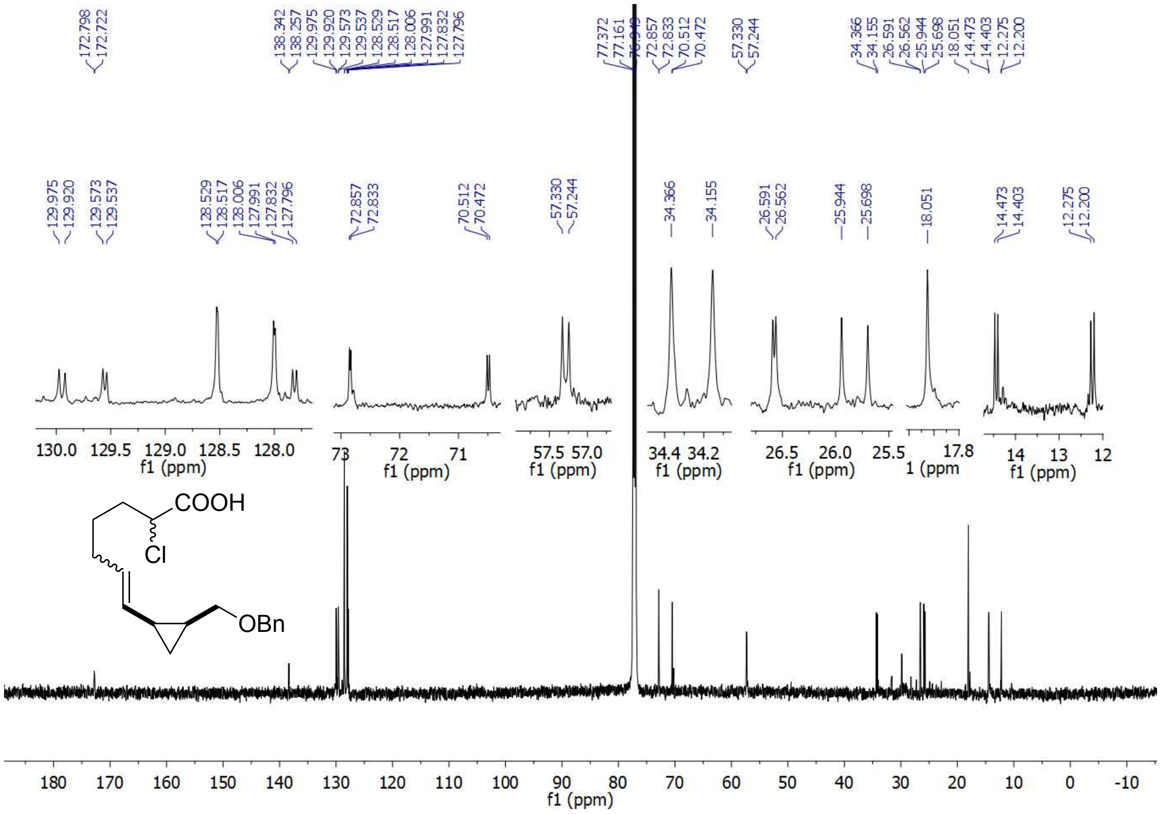

Compound 1a. Top: ^{1}H NMR ($CDCl_3$, 600 MHz). Bottom: ^{13}C NMR ($CDCl_3$, 150 MHz)

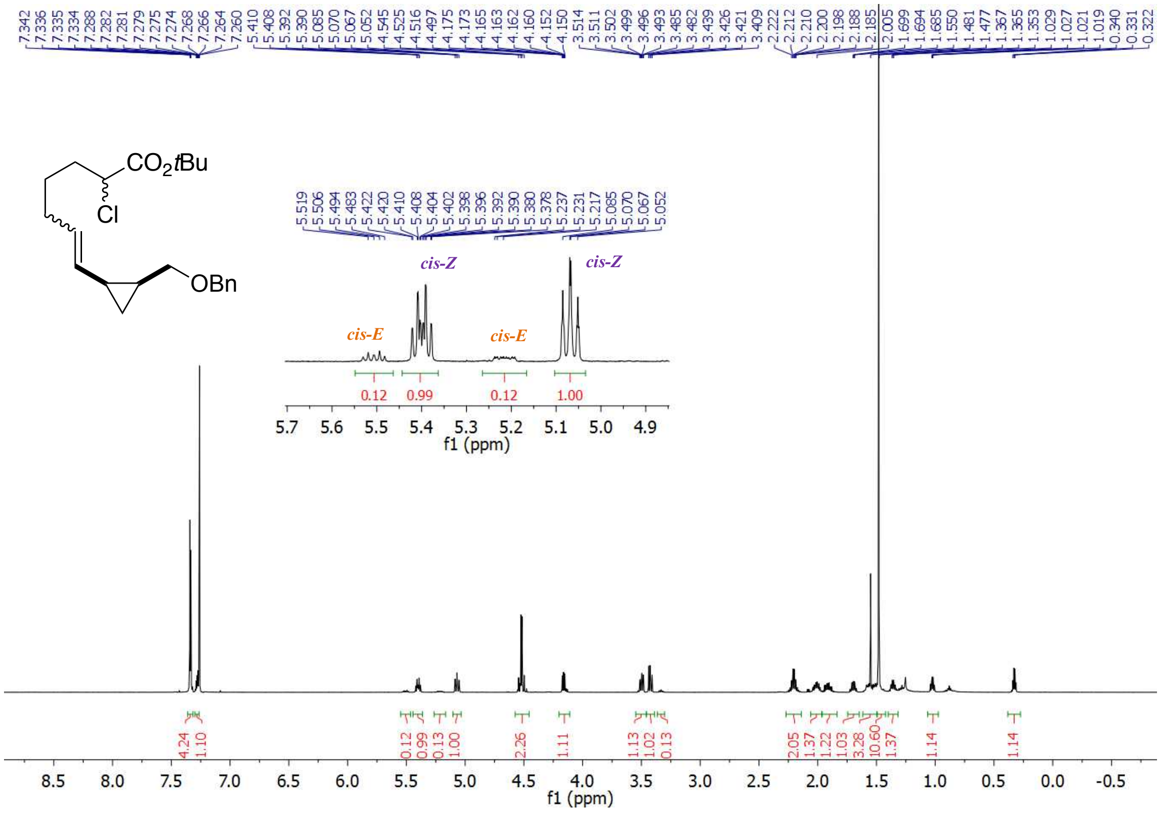

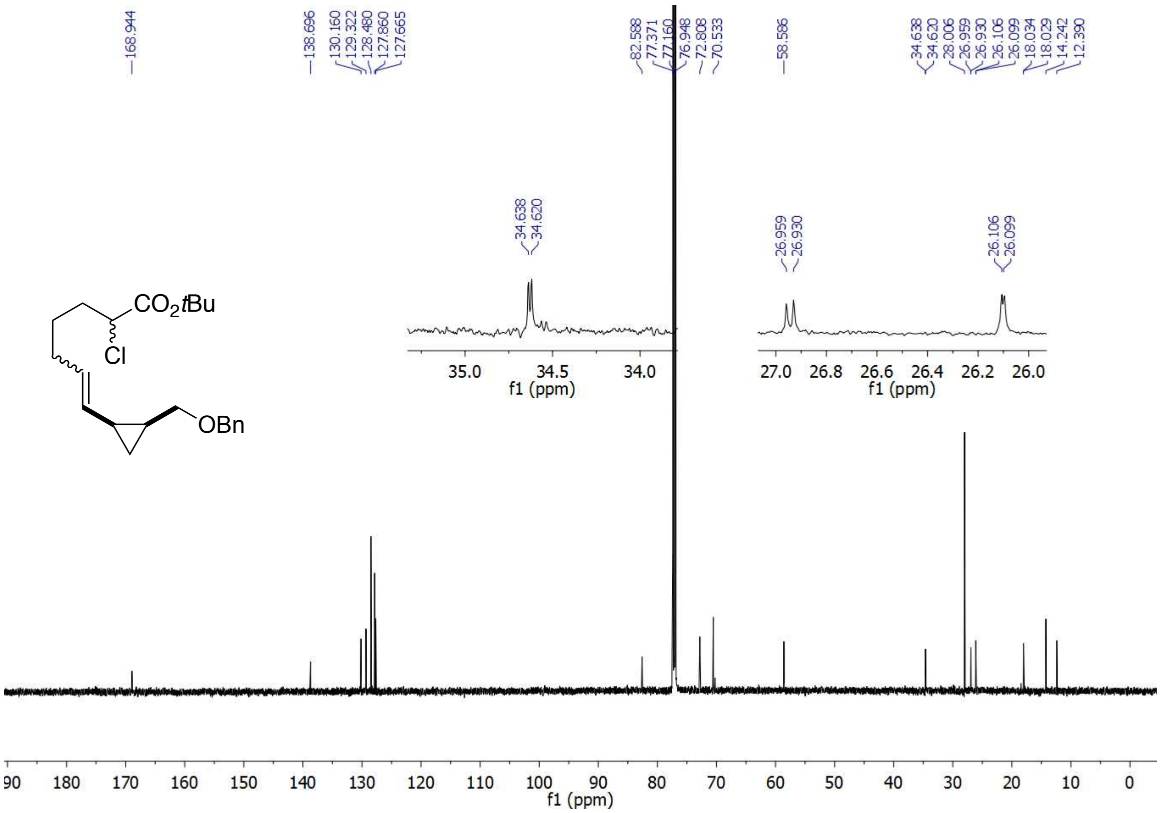

Compound 1b-d. Top: ^{1}H NMR ($CDCl_3$, 600 MHz). Bottom: ^{13}C NMR ($CDCl_3$, 150 MHz)

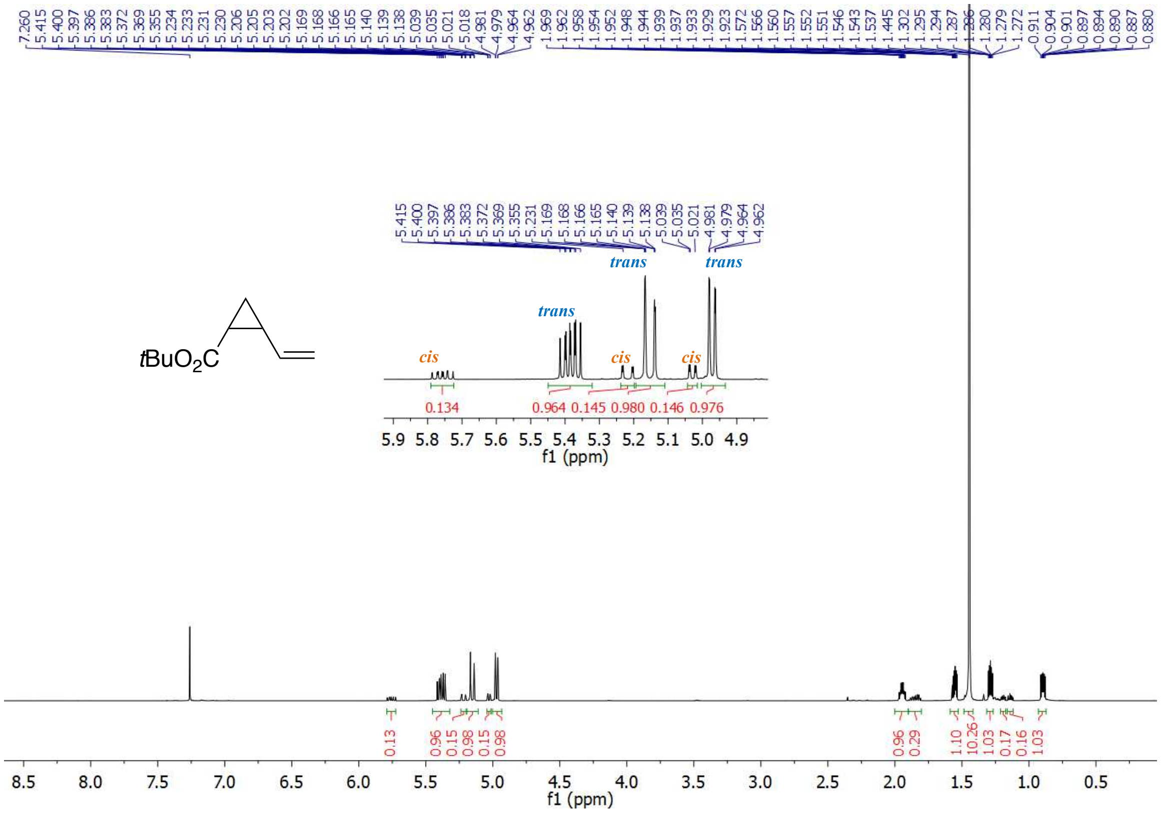

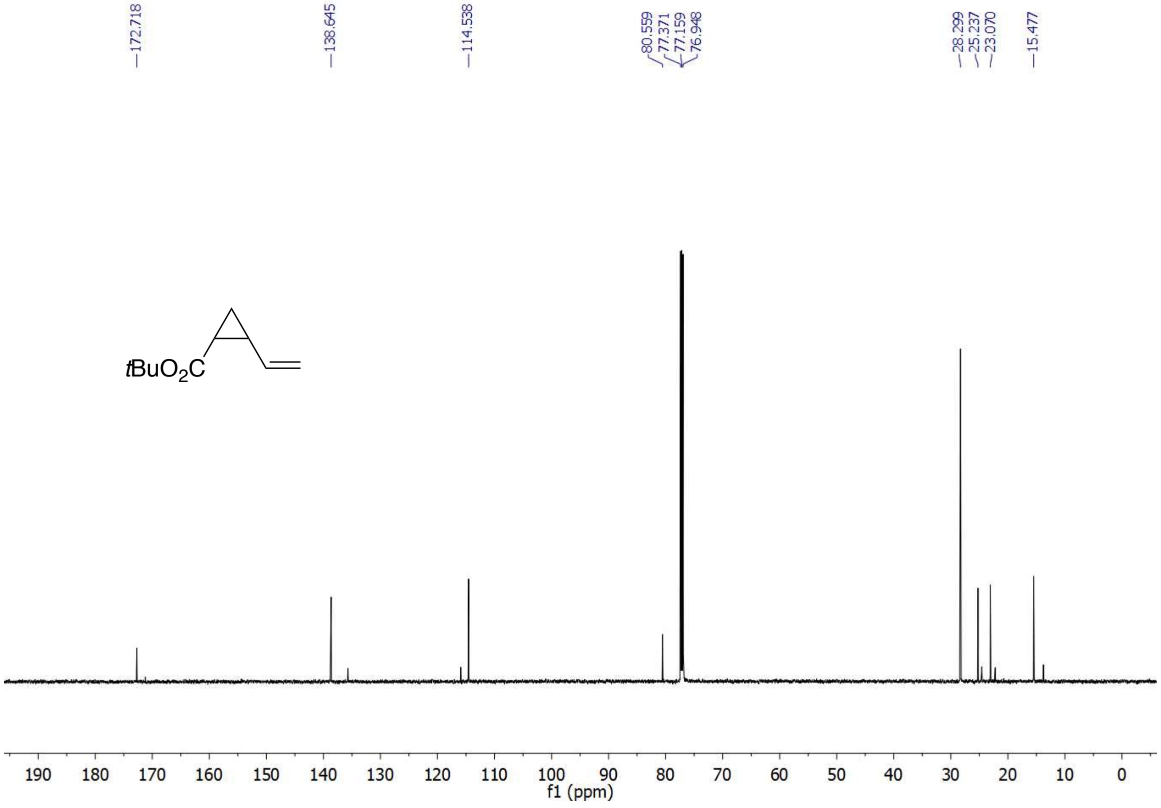

Compound 1b. Top: ^{1}H NMR ($CDCl_3$, 600 MHz). Bottom: ^{13}C NMR ($CDCl_3$, 150 MHz)

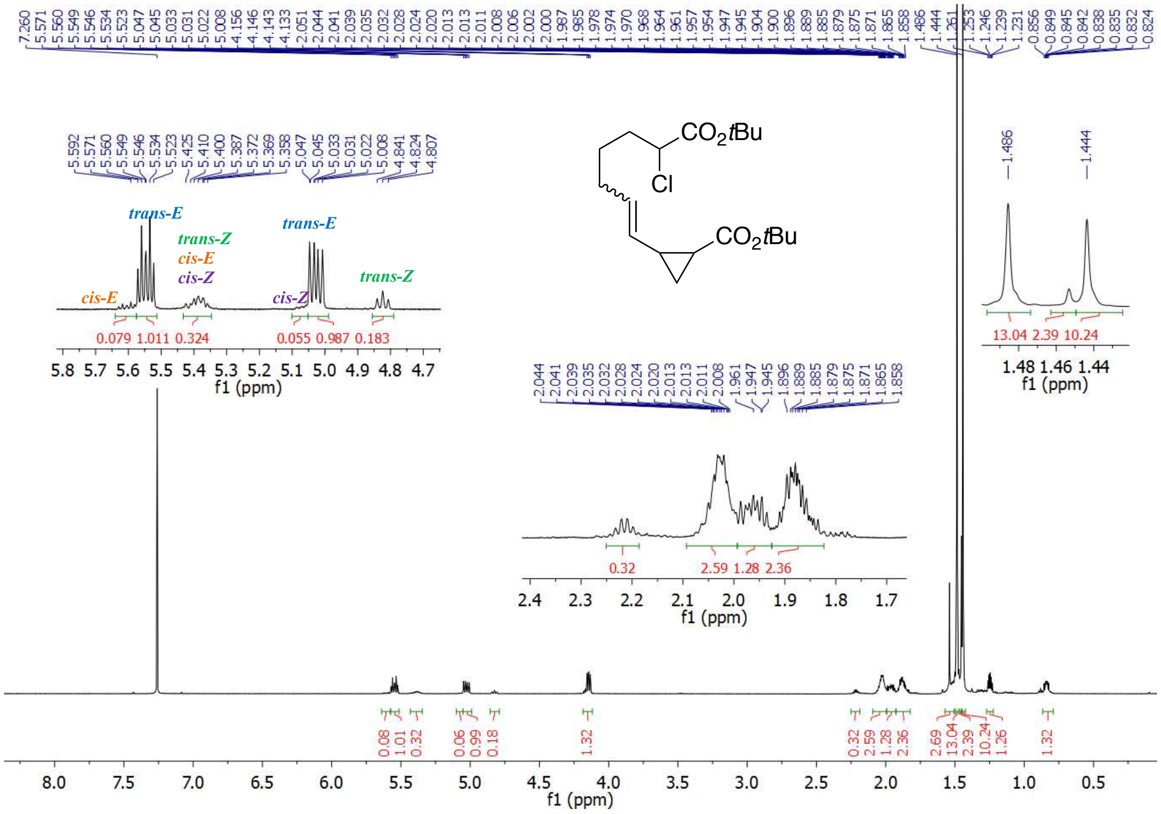

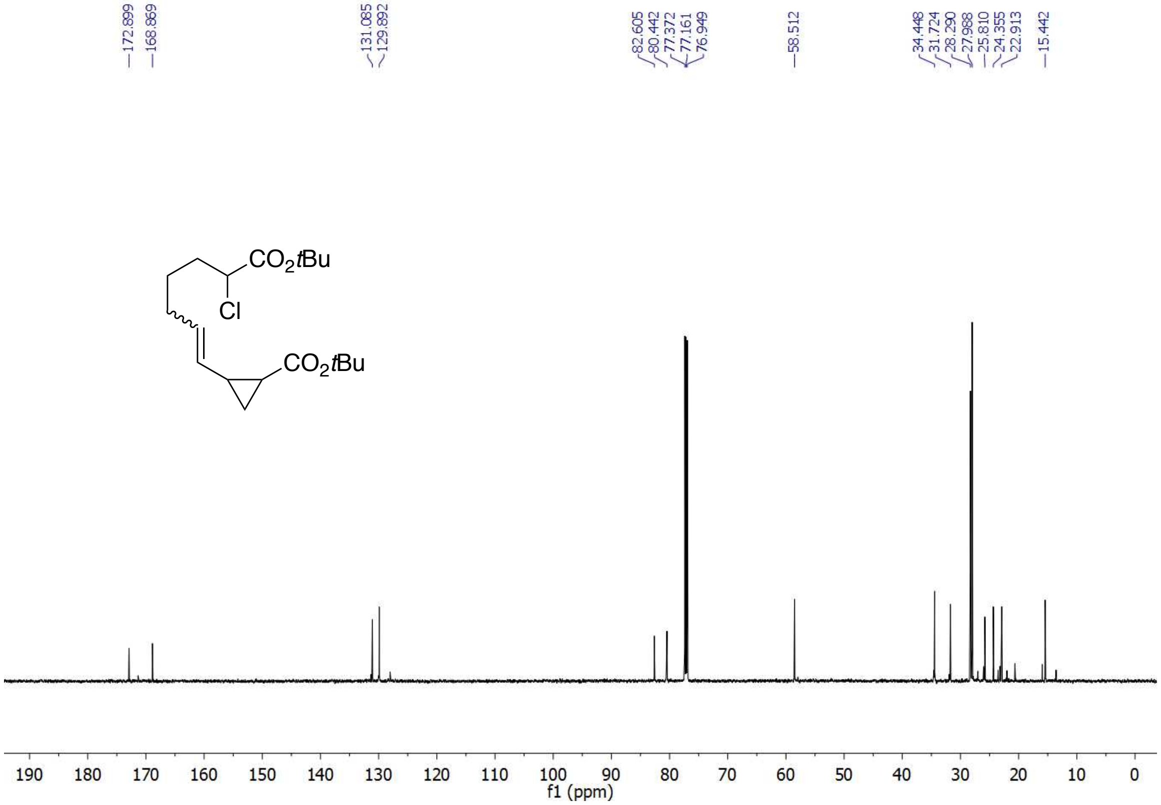

Compound 1c. Top: ^{1}H NMR ($CDCl_3$, 600 MHz). Bottom: ^{13}C NMR ($CDCl_3$, 150 MHz)

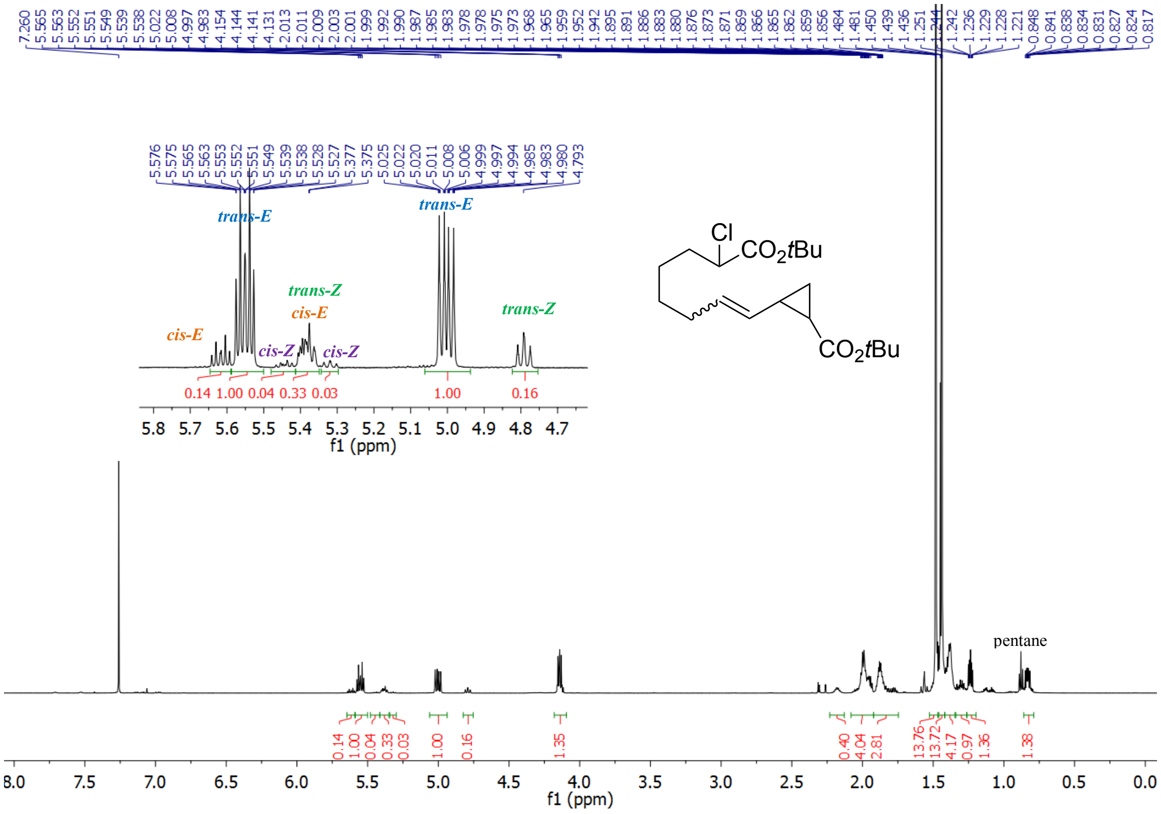

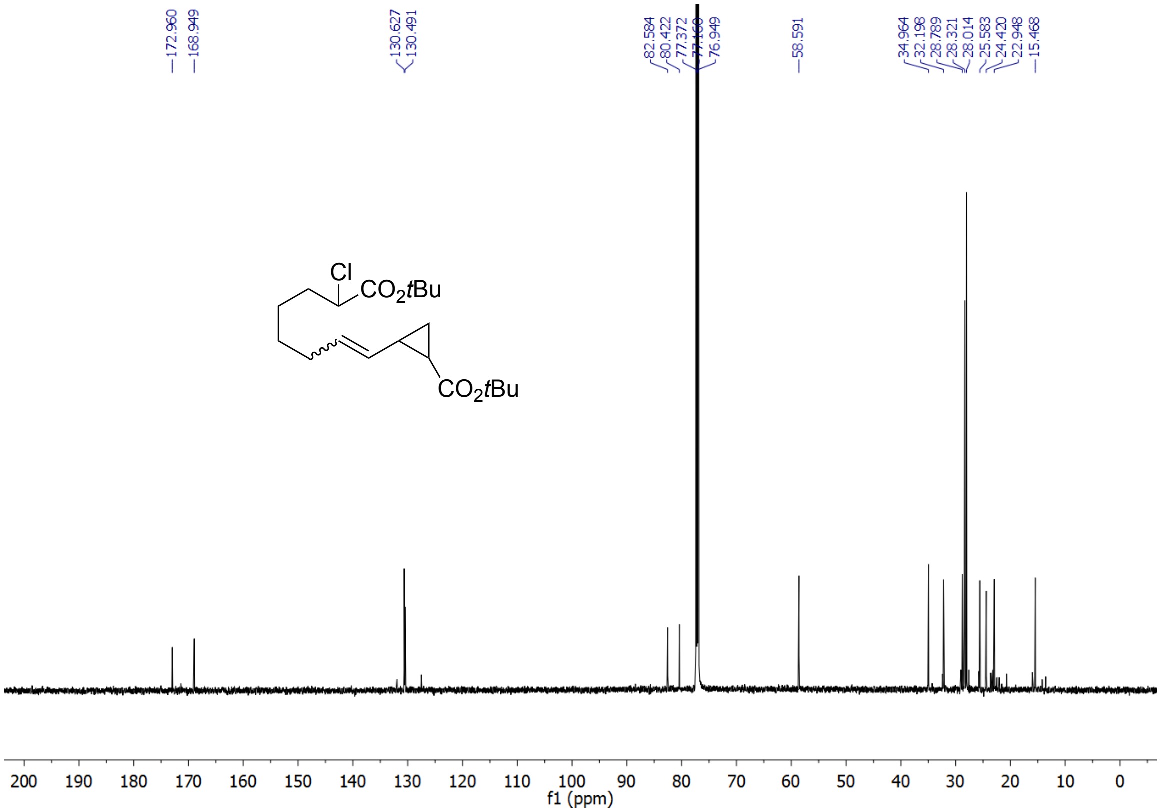

Compound 1d. Top: ^{1}H NMR ($CDCl_3$, 600 MHz). Bottom: ^{13}C NMR ($CDCl_3$, 150 MHz)

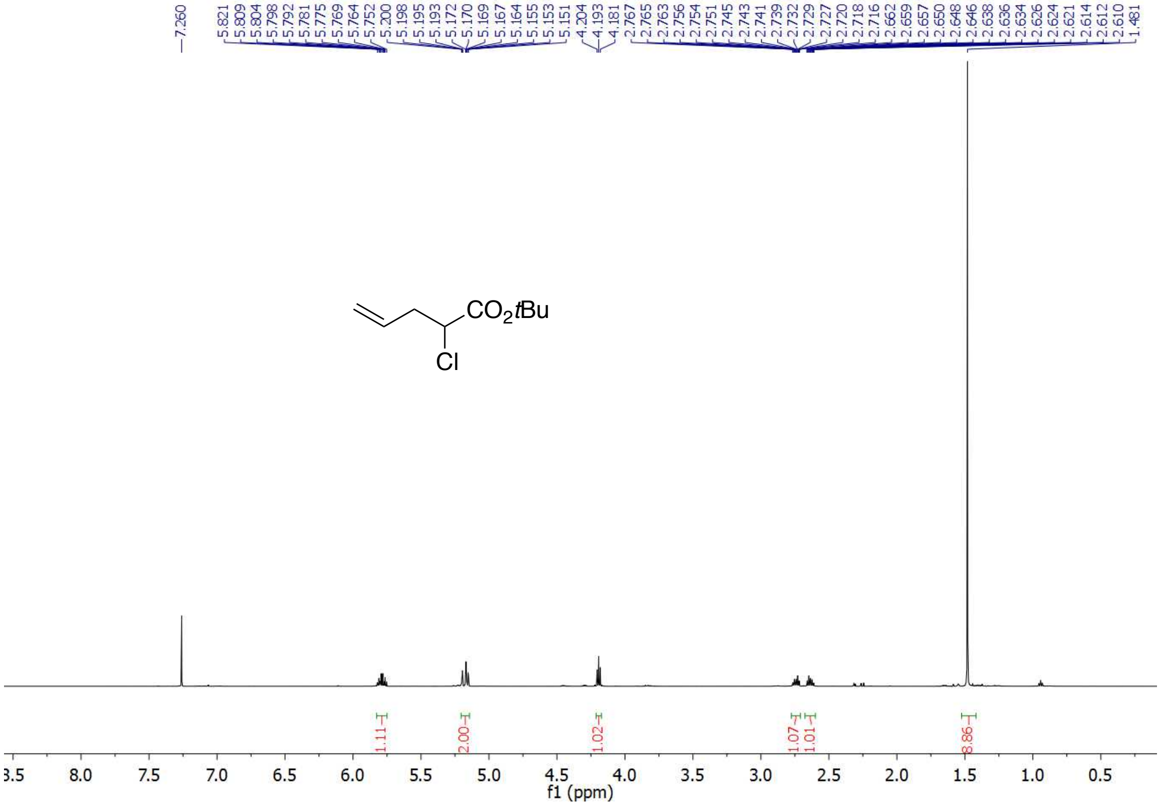

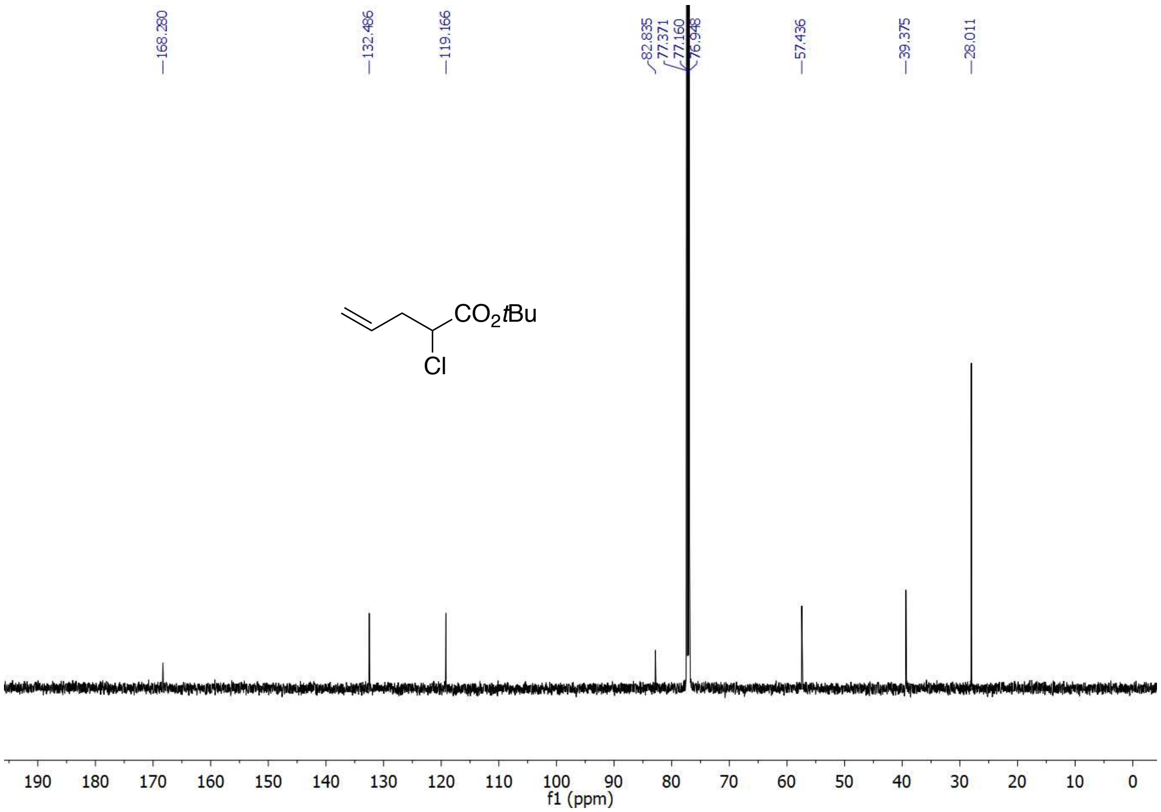

Compound (*trans*, *E*)-2a'. Top: ^1H NMR ($CDCl_3$, 600 MHz). Bottom: ^{13}C NMR ($CDCl_3$, 150 MHz)

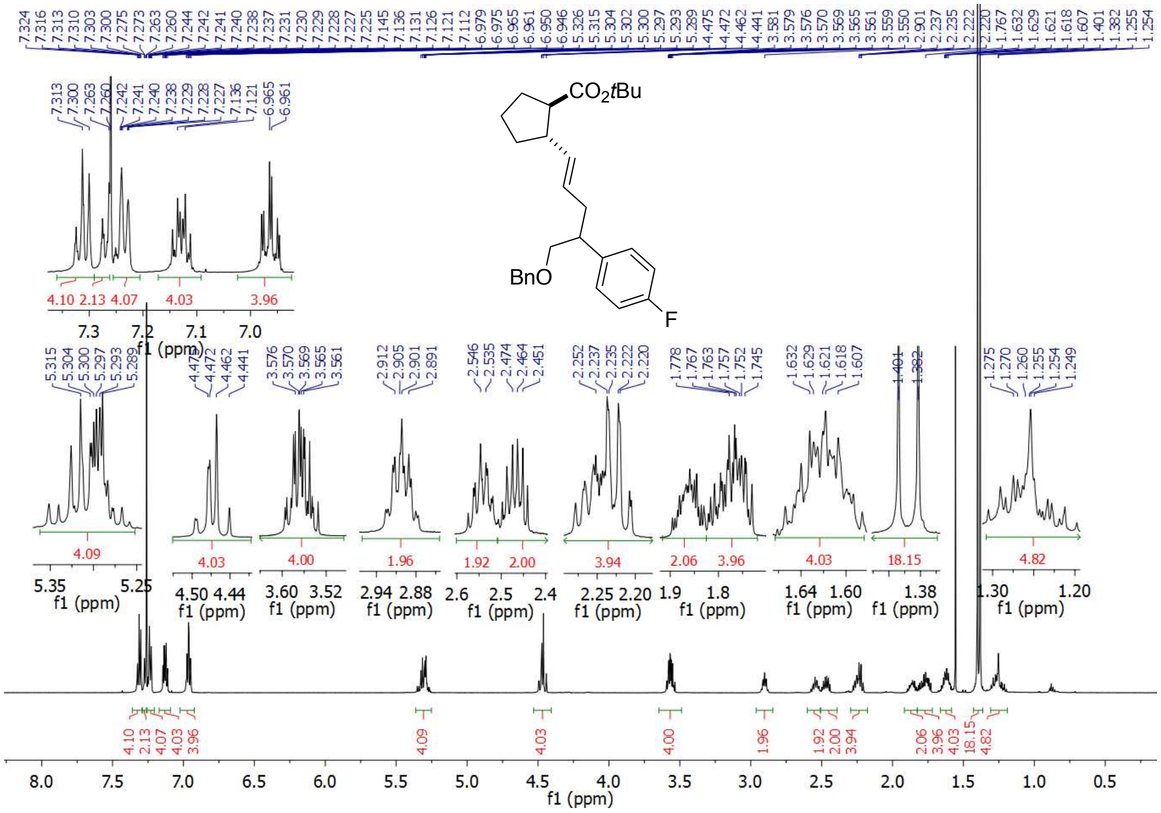

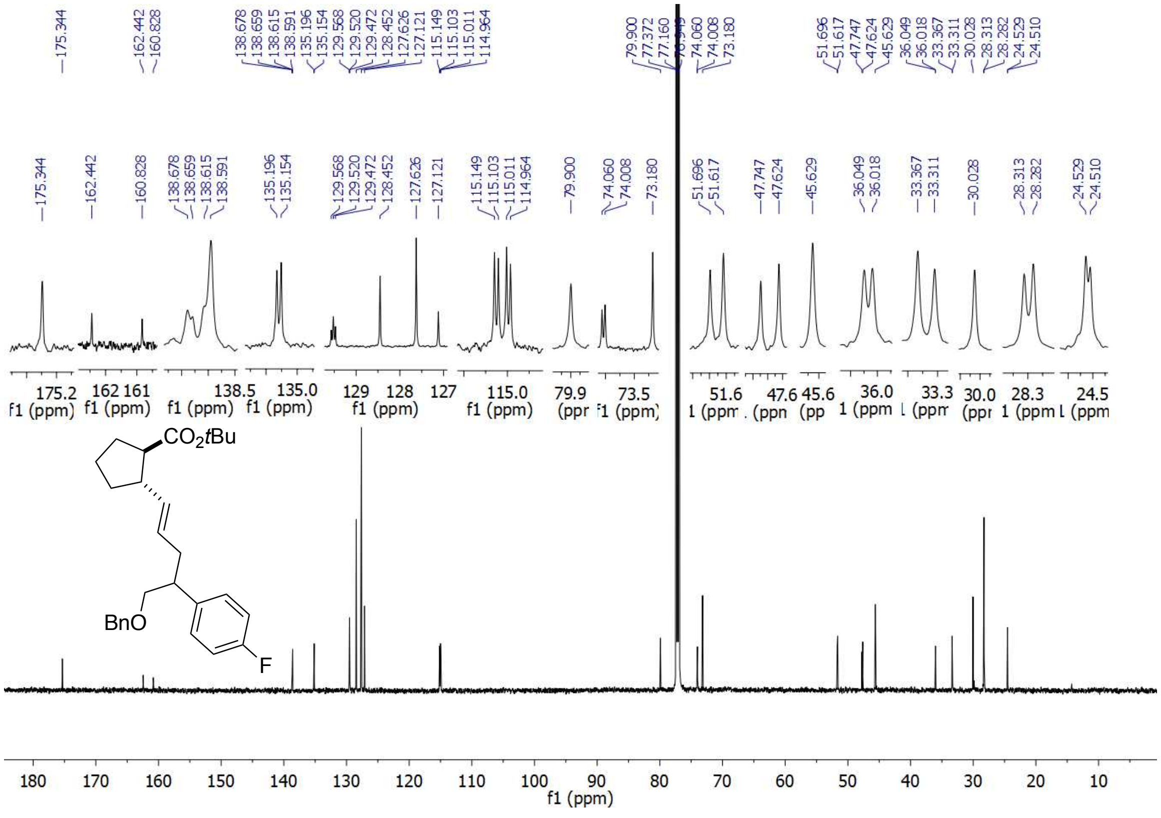

Compound (*trans*, *E*)-2a'. Top: ^{19}F NMR ($CDCl_3$, 565 MHz). Bottom: ^{1}H NMR (C_6D_6, 600 MHz)

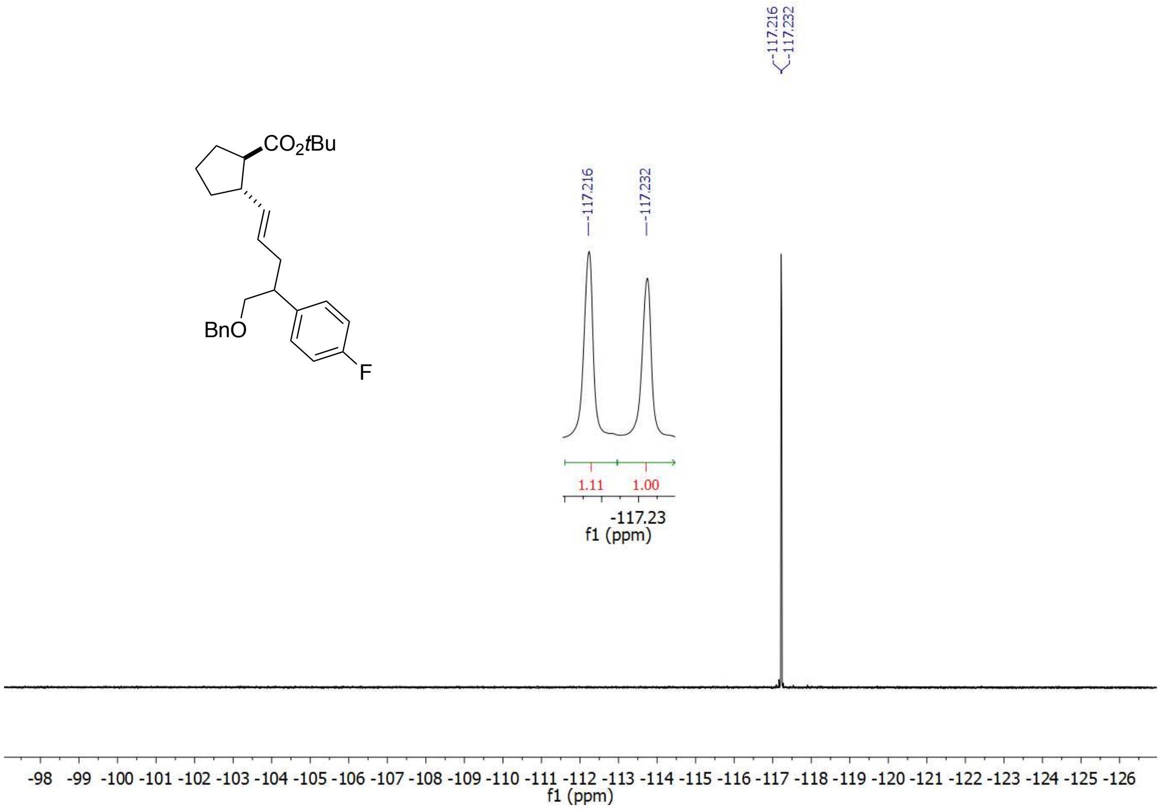

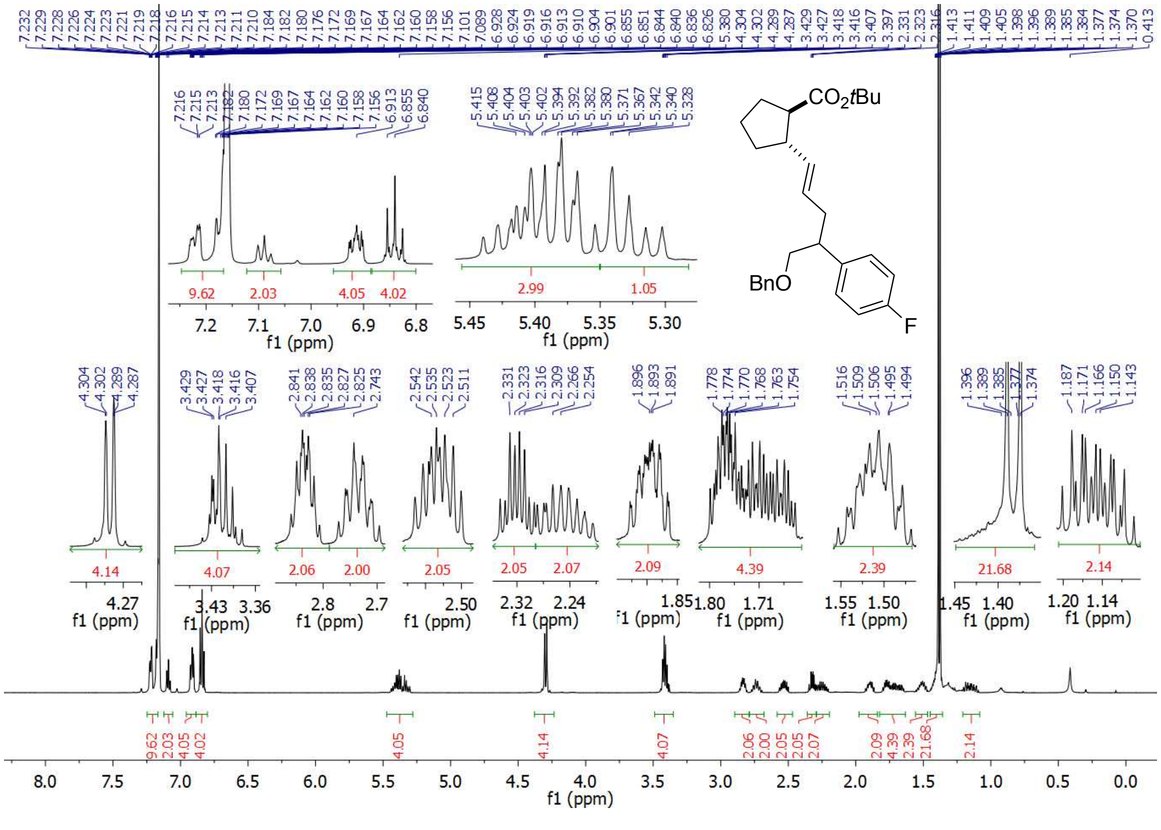

Compound (*trans*, *E*)-2a'. Top: COSY ($CDCl_3$, 600 MHz). Bottom: HSQC ($CDCl_3$, 600 MHz)

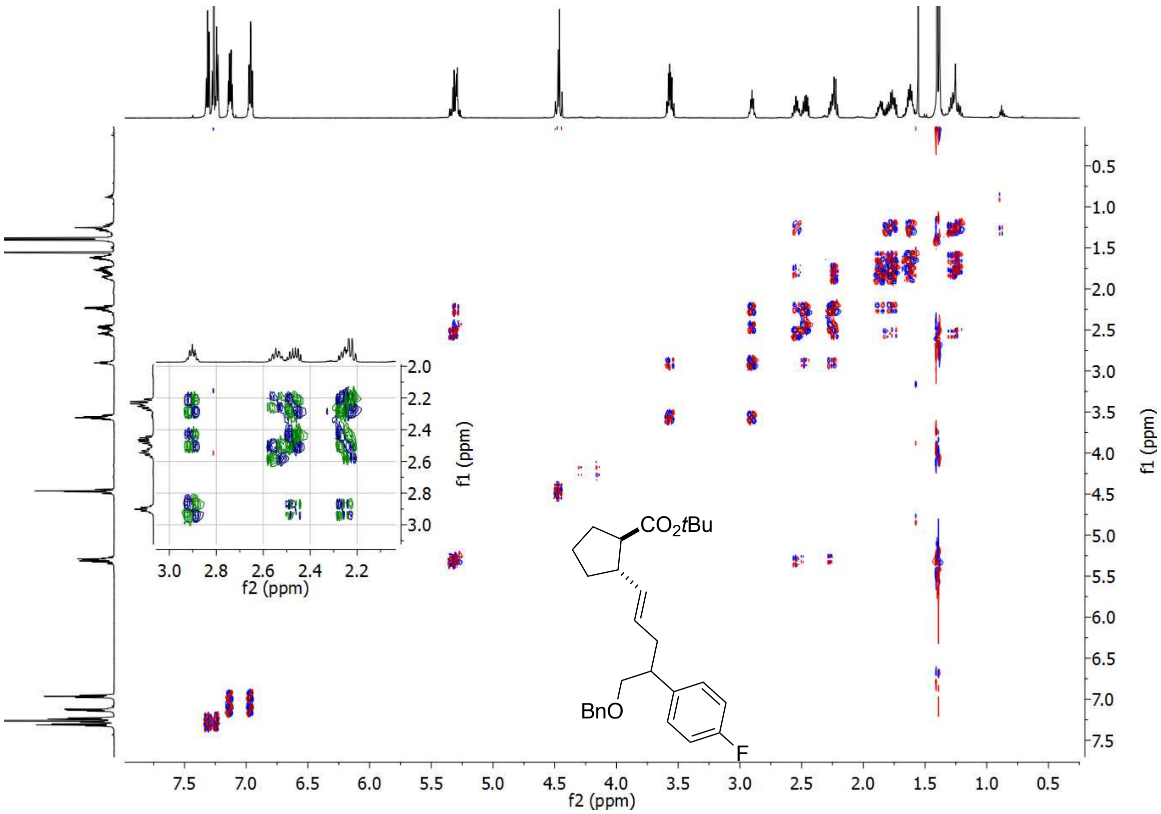

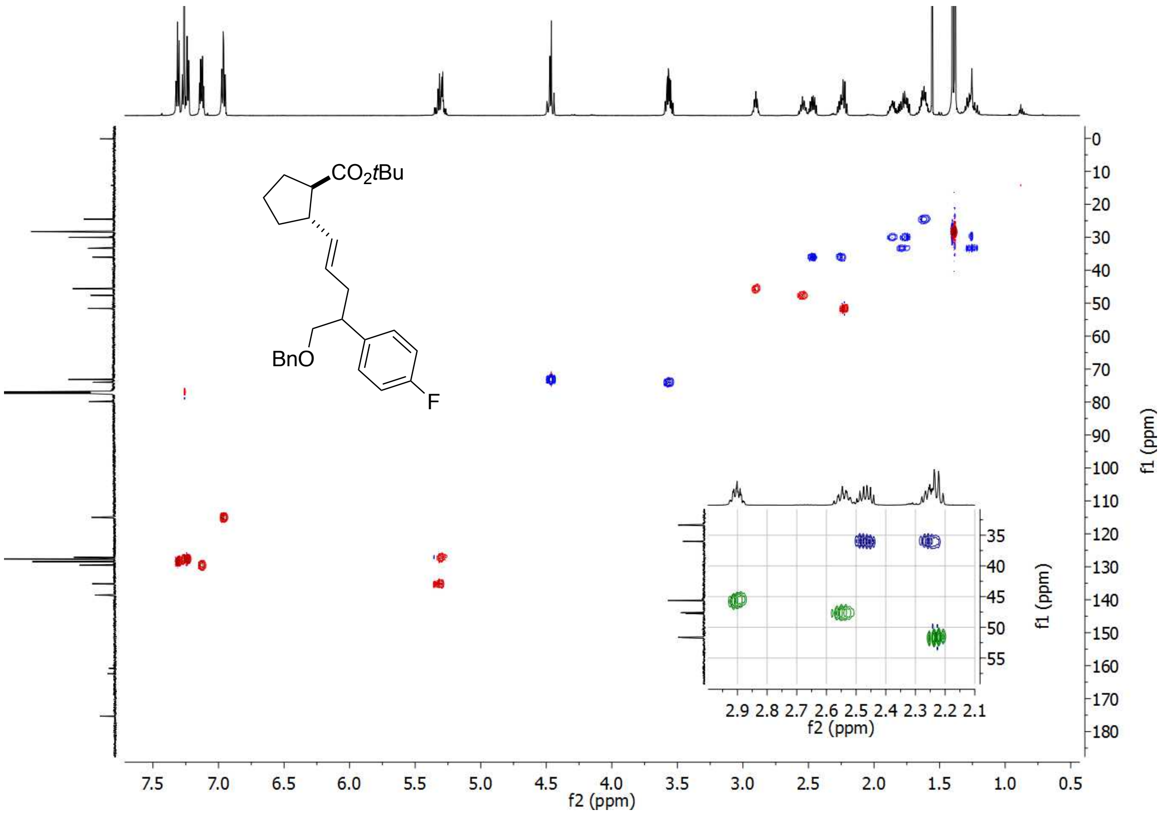

Compound (*trans*, *E*)-2a'. Top: NOESY ($CDCl_3$, 600 MHz). Bottom: HMBC ($CDCl_3$, 600 MHz)

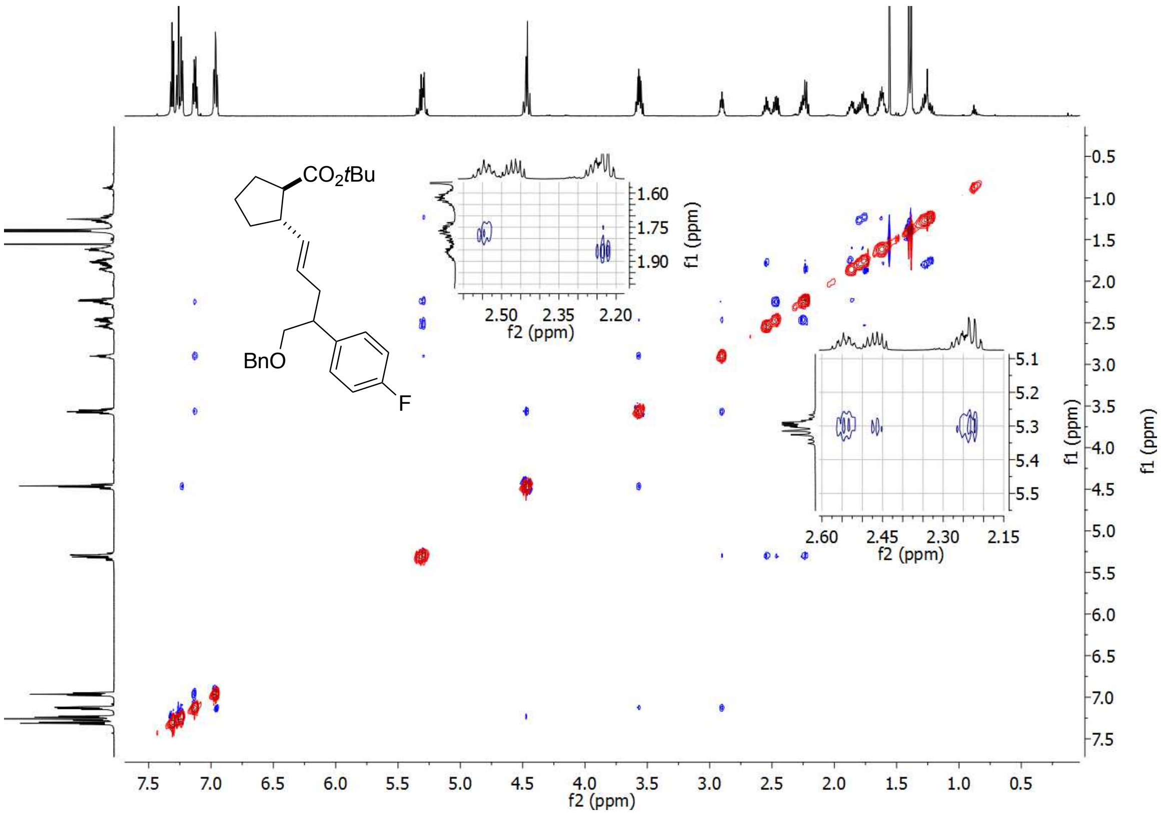

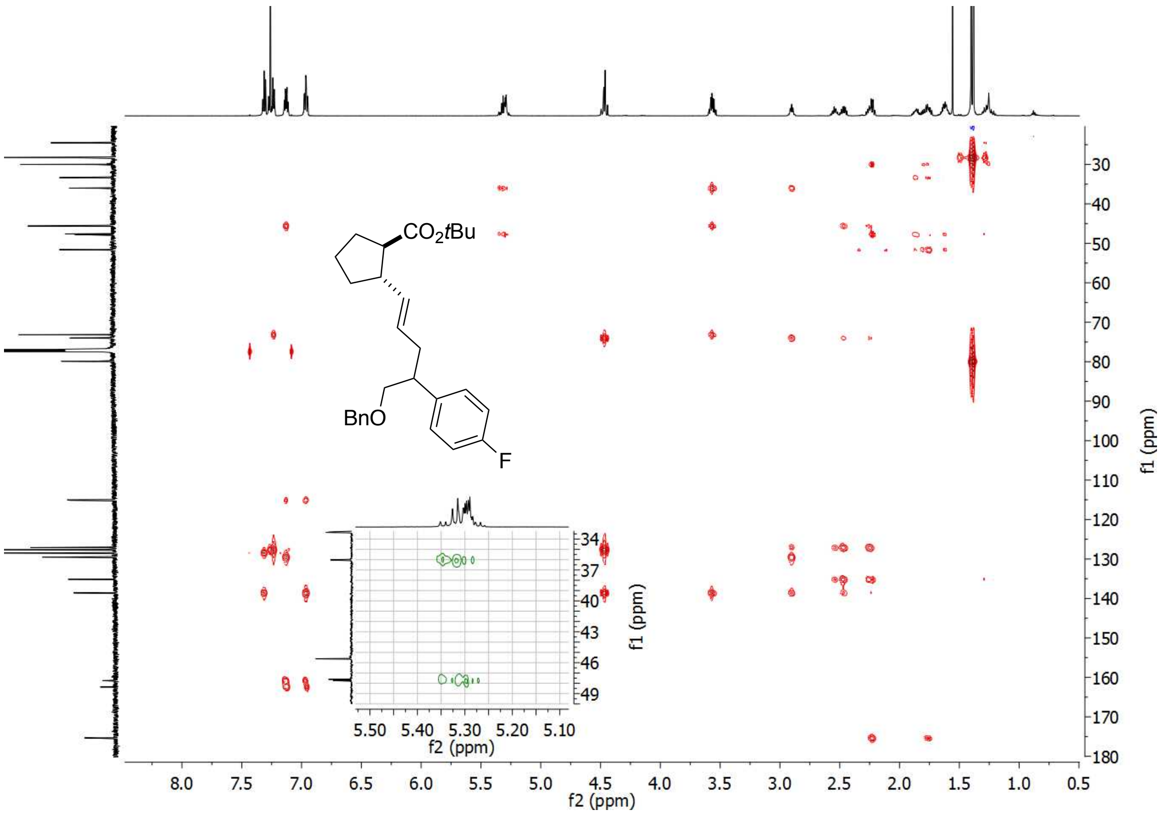

Compound (*trans*, *E*)-2a. Top: ^{1}H NMR ($CDCl_3$, 600 MHz). Bottom: ^{13}C NMR ($CDCl_3$, 150 MHz)

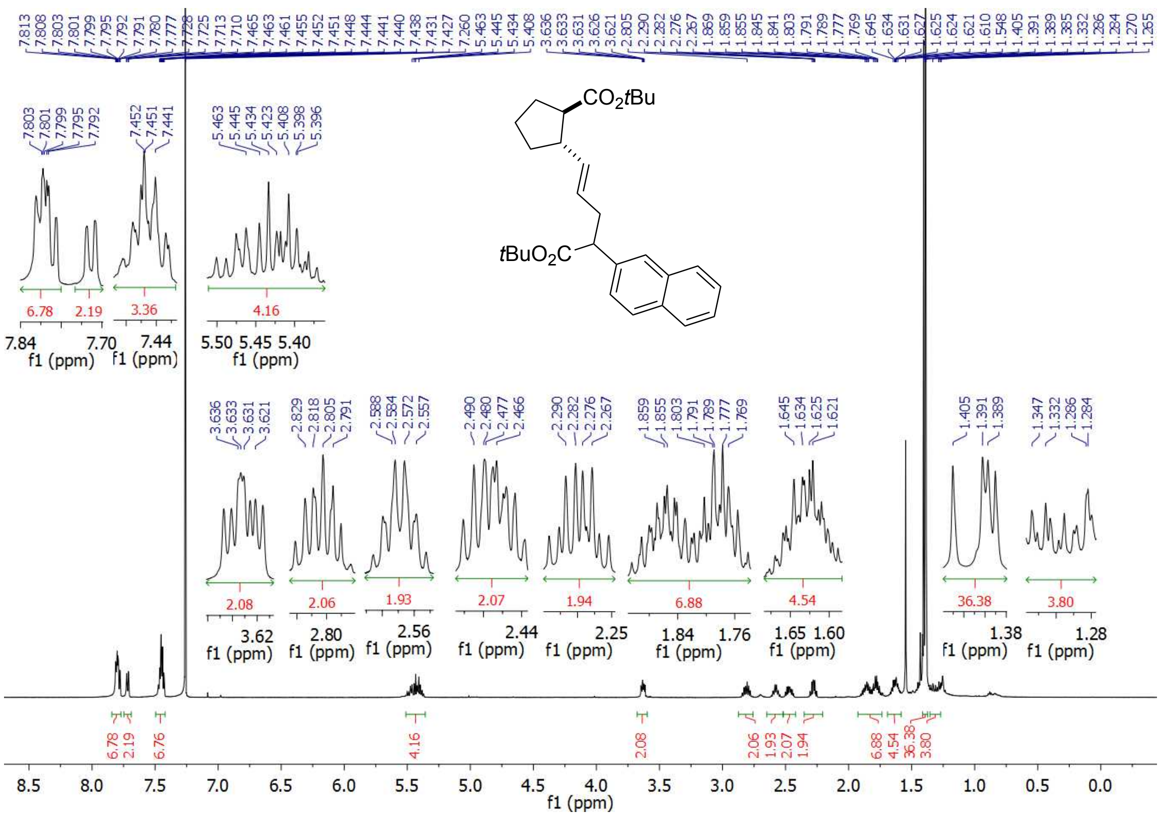

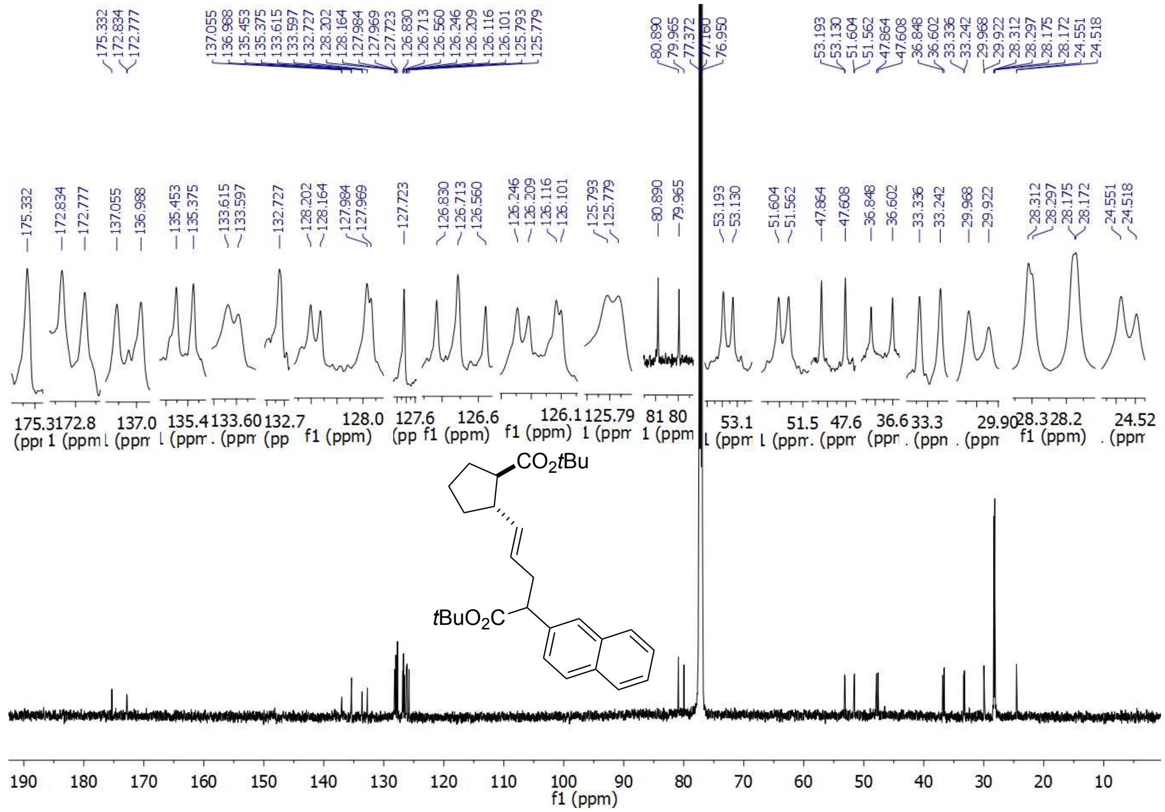

Compound (*trans*, *E*)-2a. Top: ^{1}H NMR (C_6D_6, 600 MHz). Bottom: COSY ($CDCl_3$, 600 MHz)

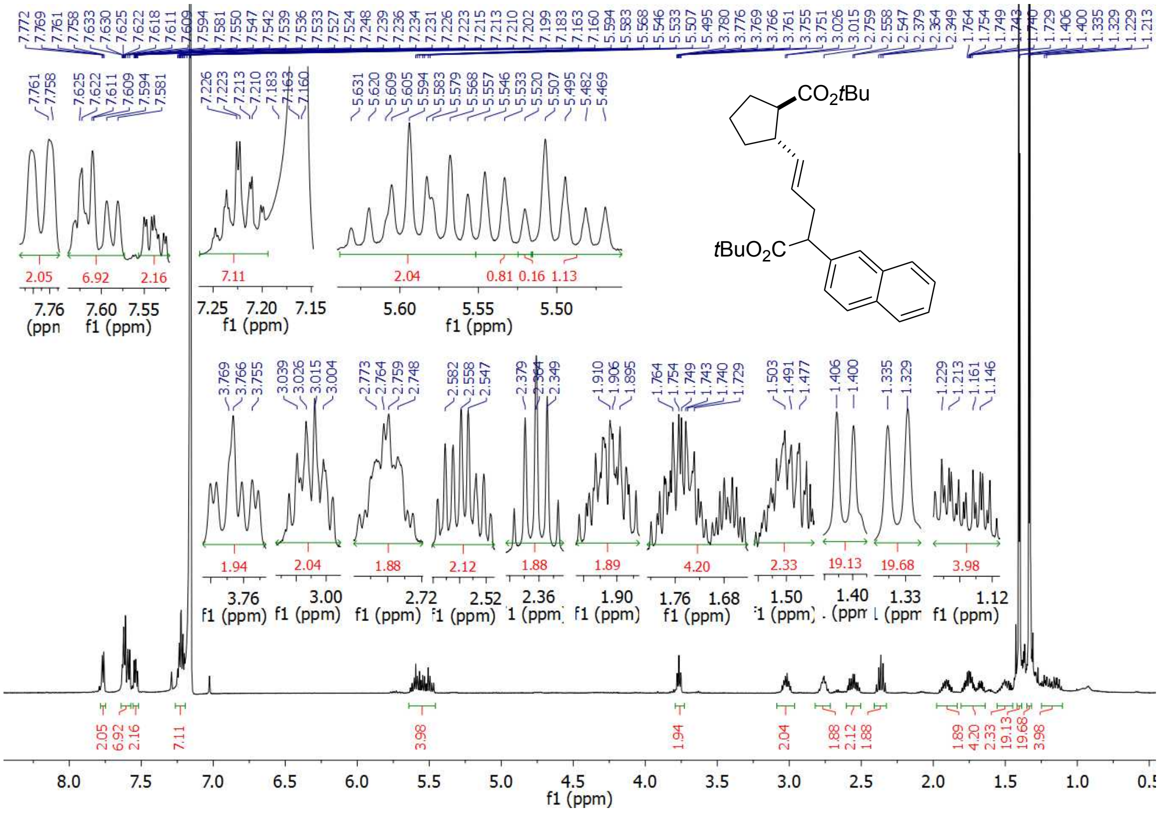

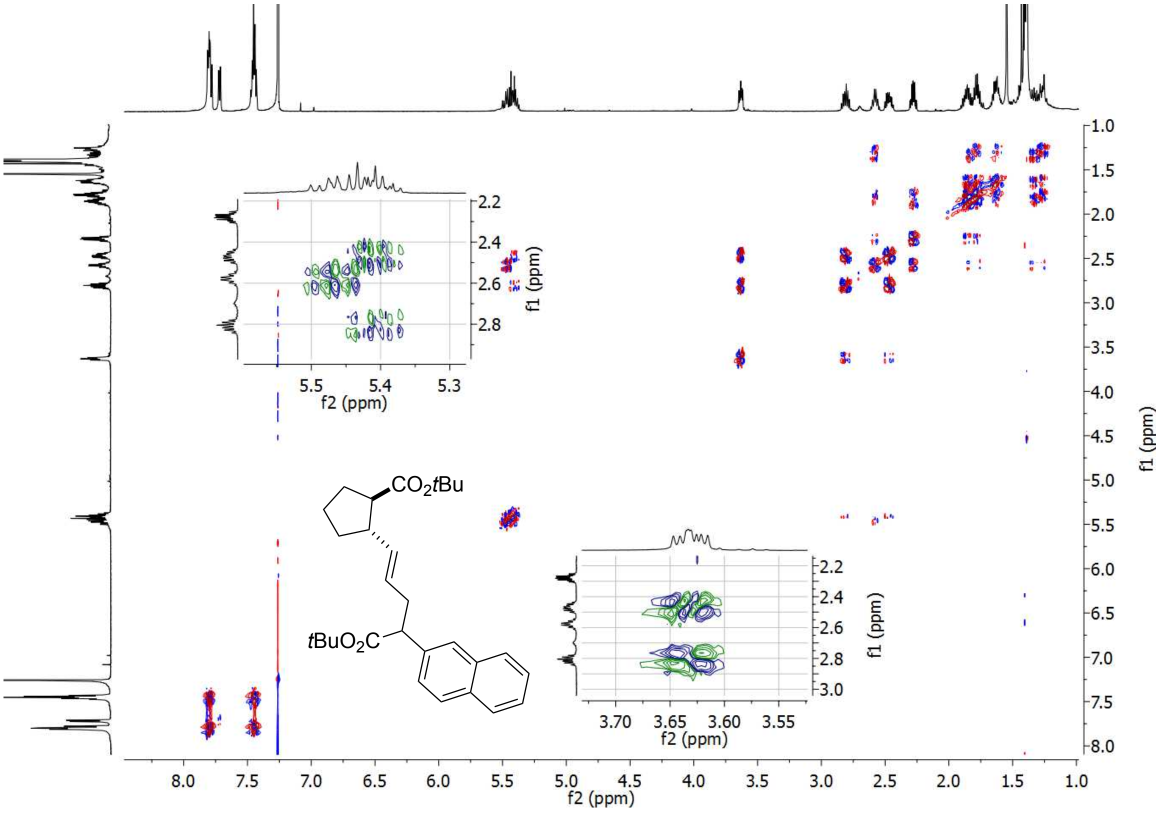

Compound (*trans*, *E*)-2a. Top: HSQC ($CDCl_3$, 600 MHz). Bottom: NOESY ($CDCl_3$, 600 MHz)

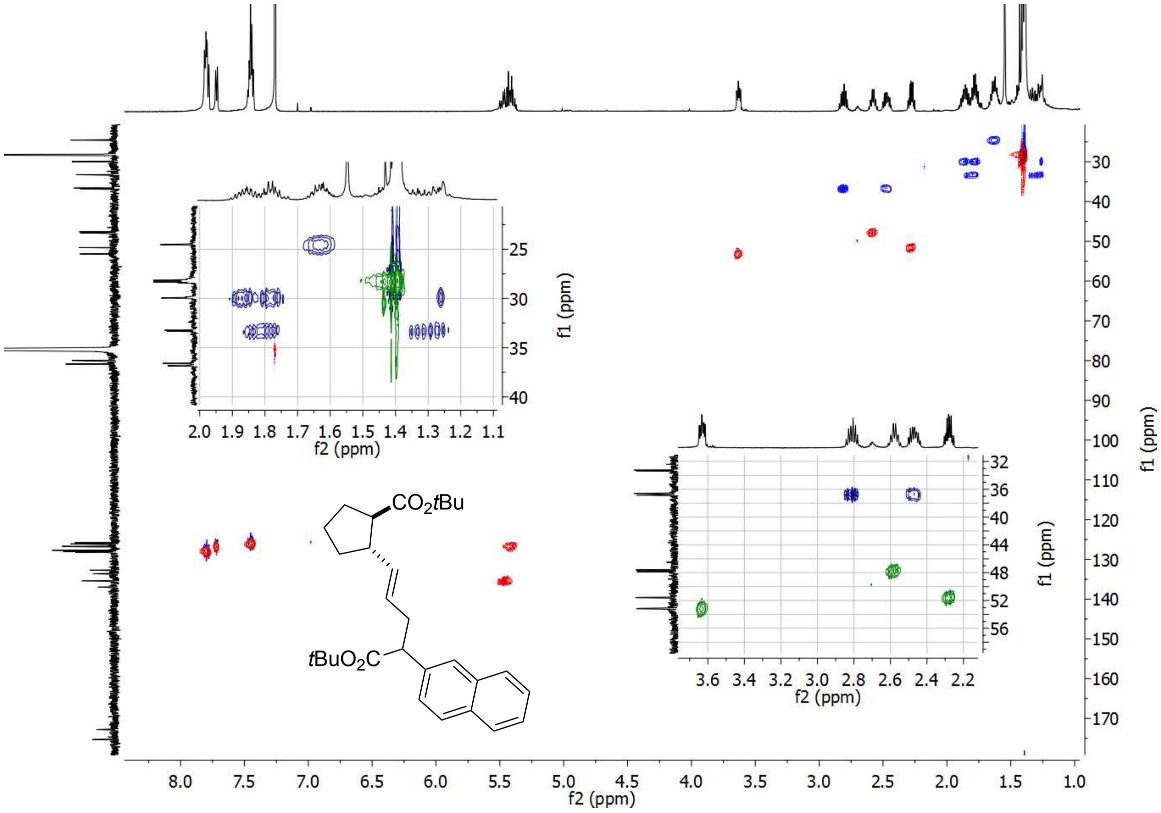

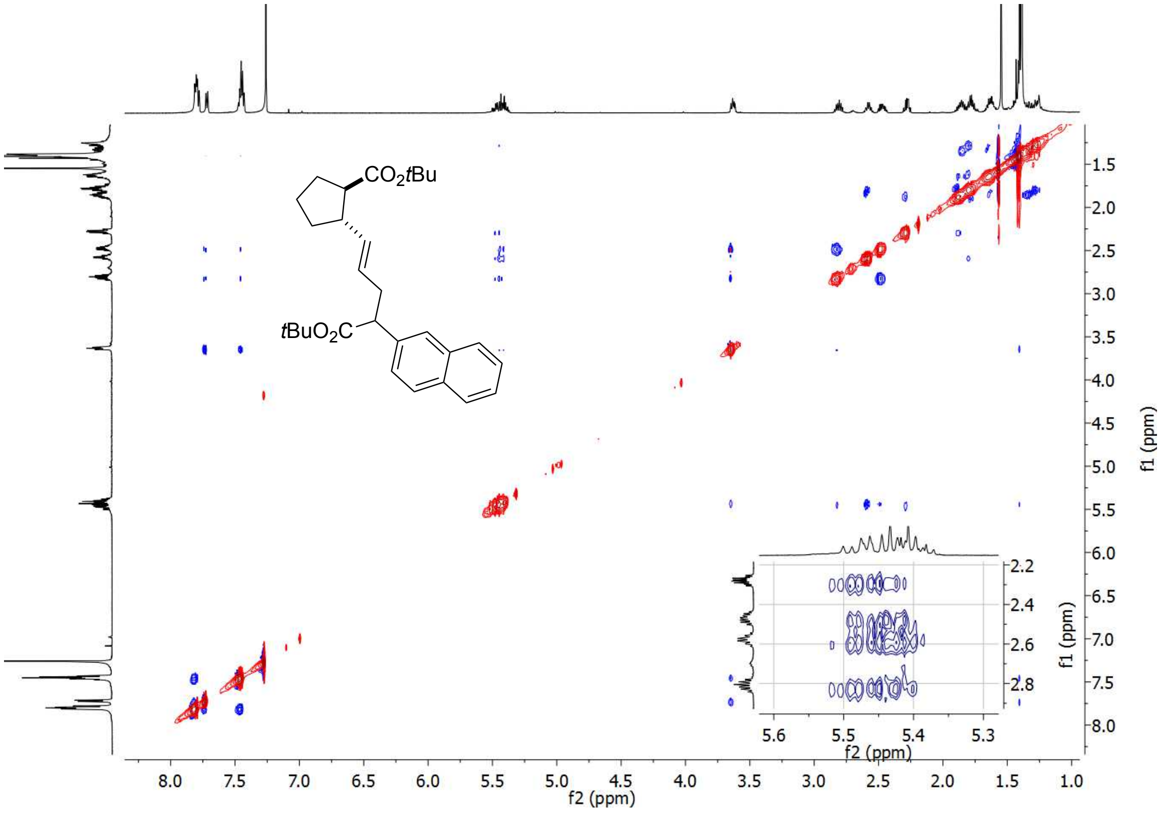

Compound (*trans*, *E*)-2a. HMBC ($CDCl_3$, 600 MHz)

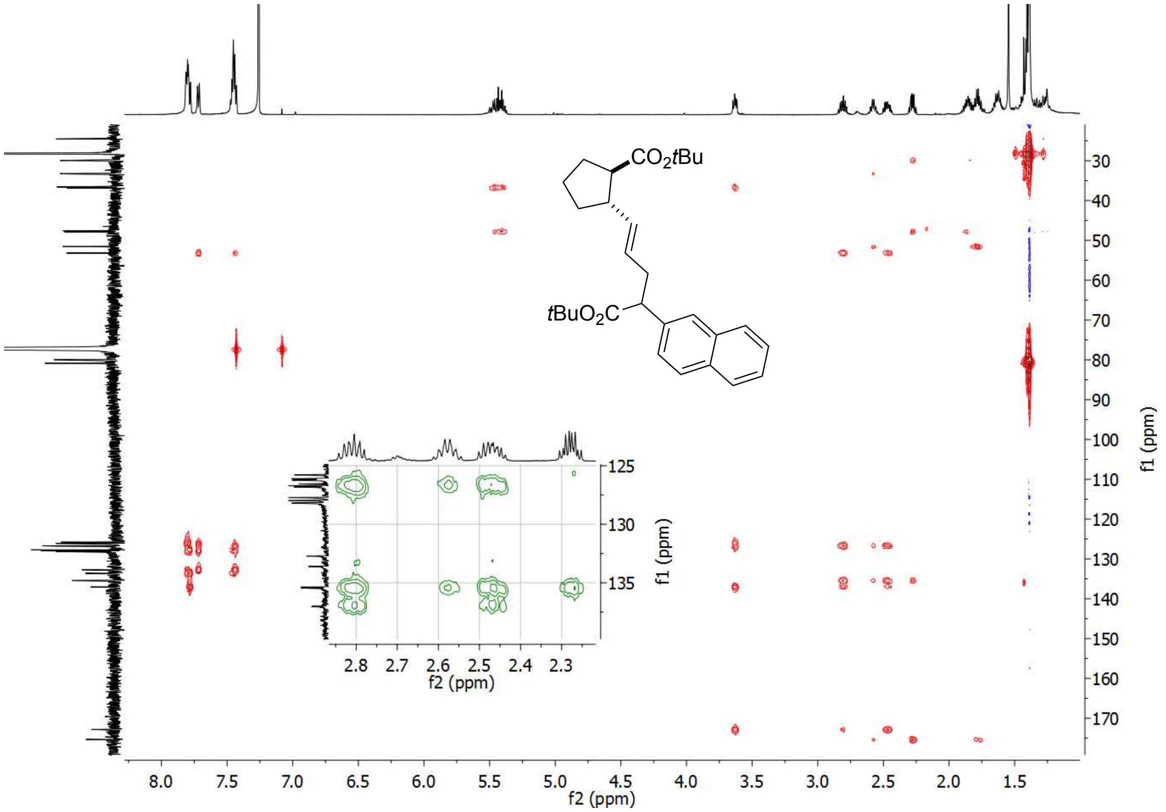

Compound *trans*-3b'. Top: ^{1}H NMR ($CDCl_3$, 600 MHz). Bottom: ^{13}C NMR ($CDCl_3$, 150 MHz)

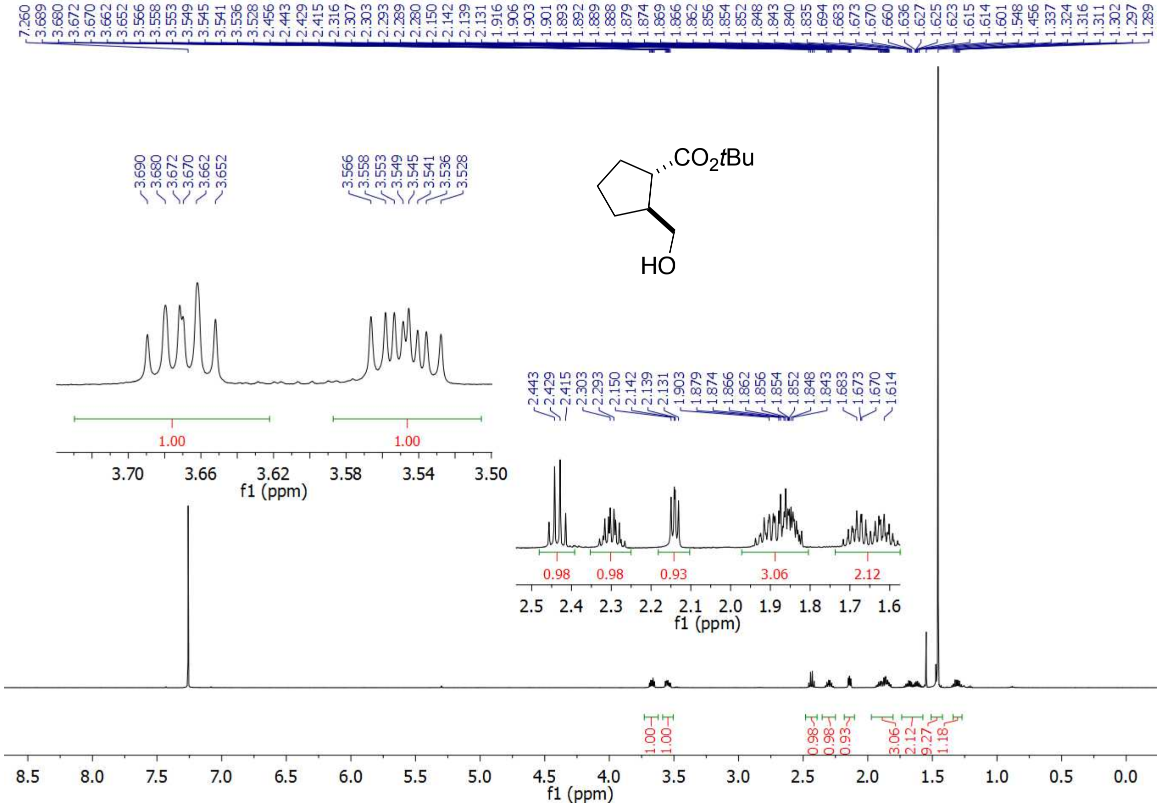

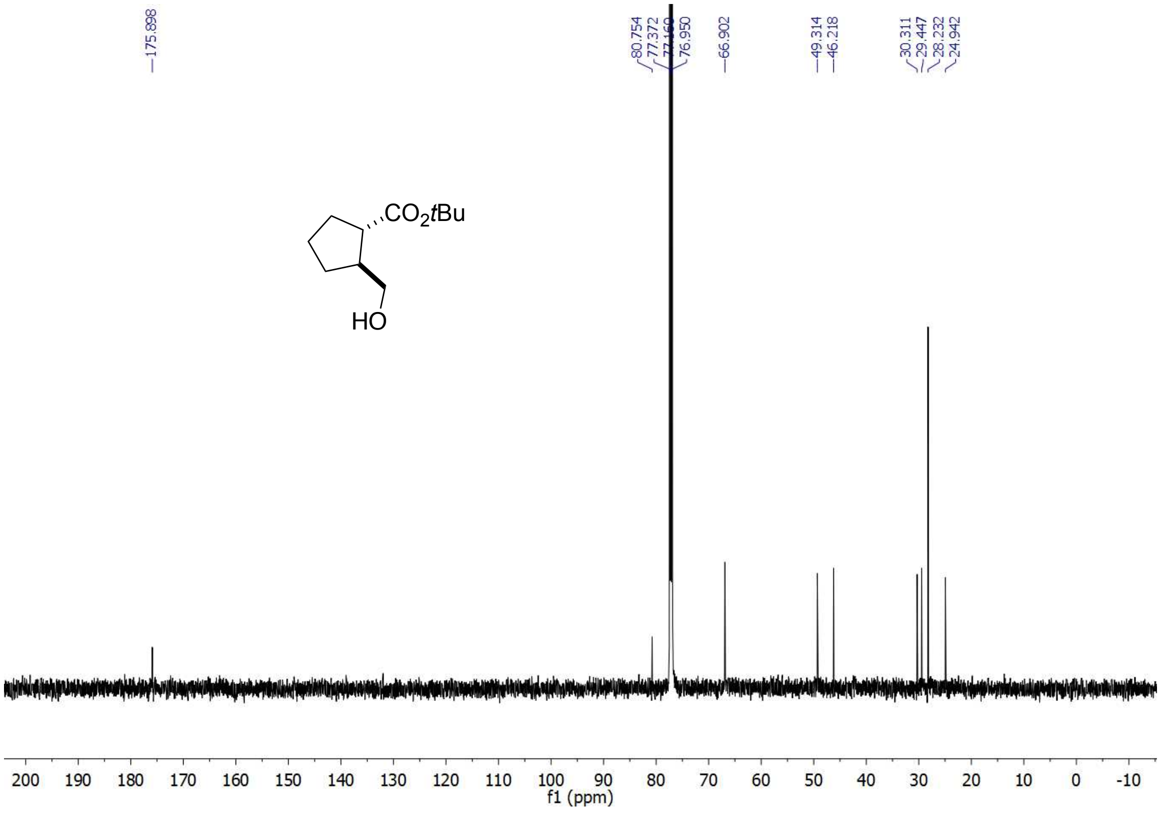

Compound 3a'. Top: ^{1}H NMR ($CDCl_3$, 600 MHz). Bottom: ^{13}C NMR ($CDCl_3$, 150 MHz)

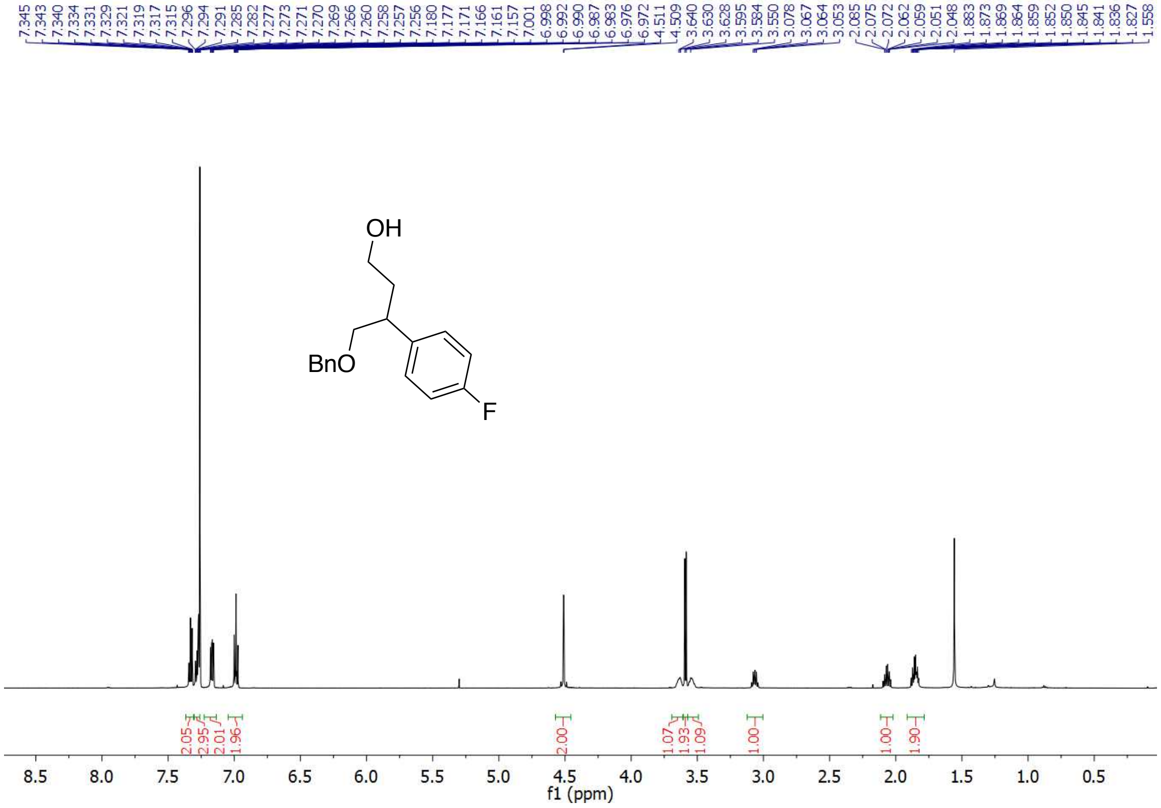

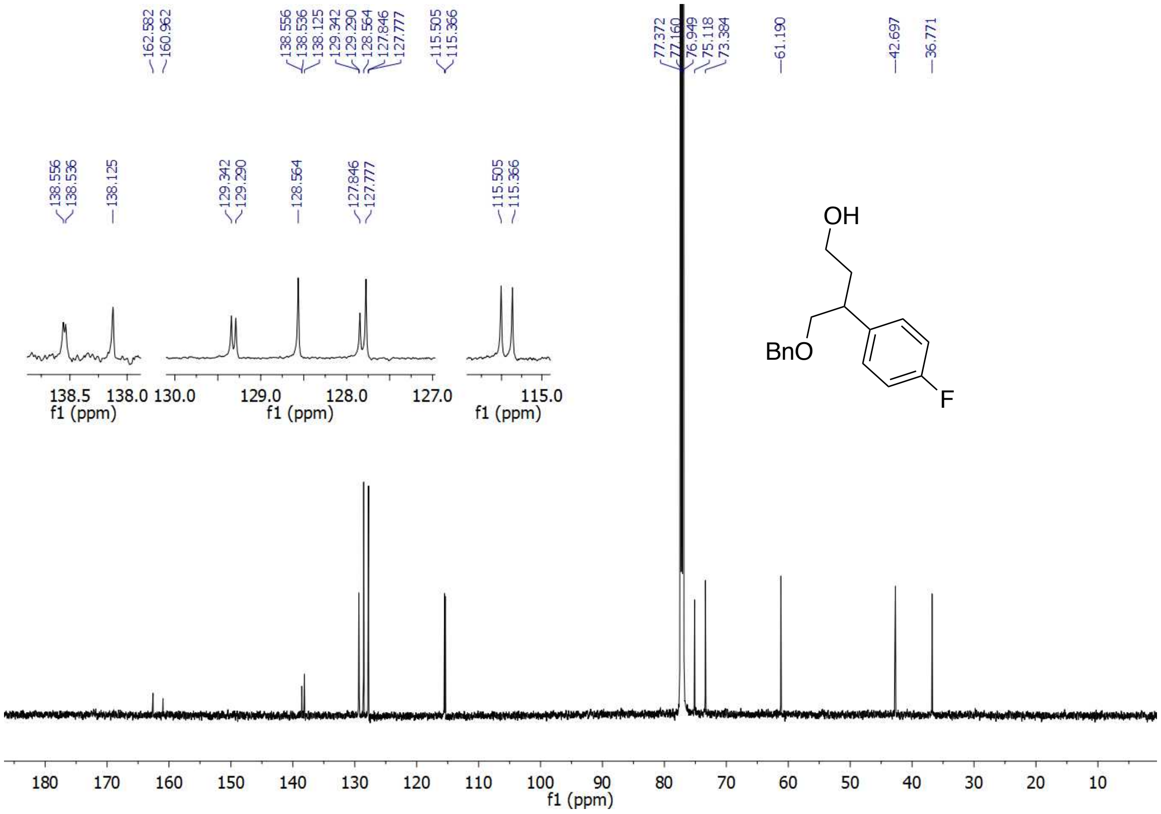

Compound 3a’. ^{19}F NMR (CDCl$_3$, 565 MHz).

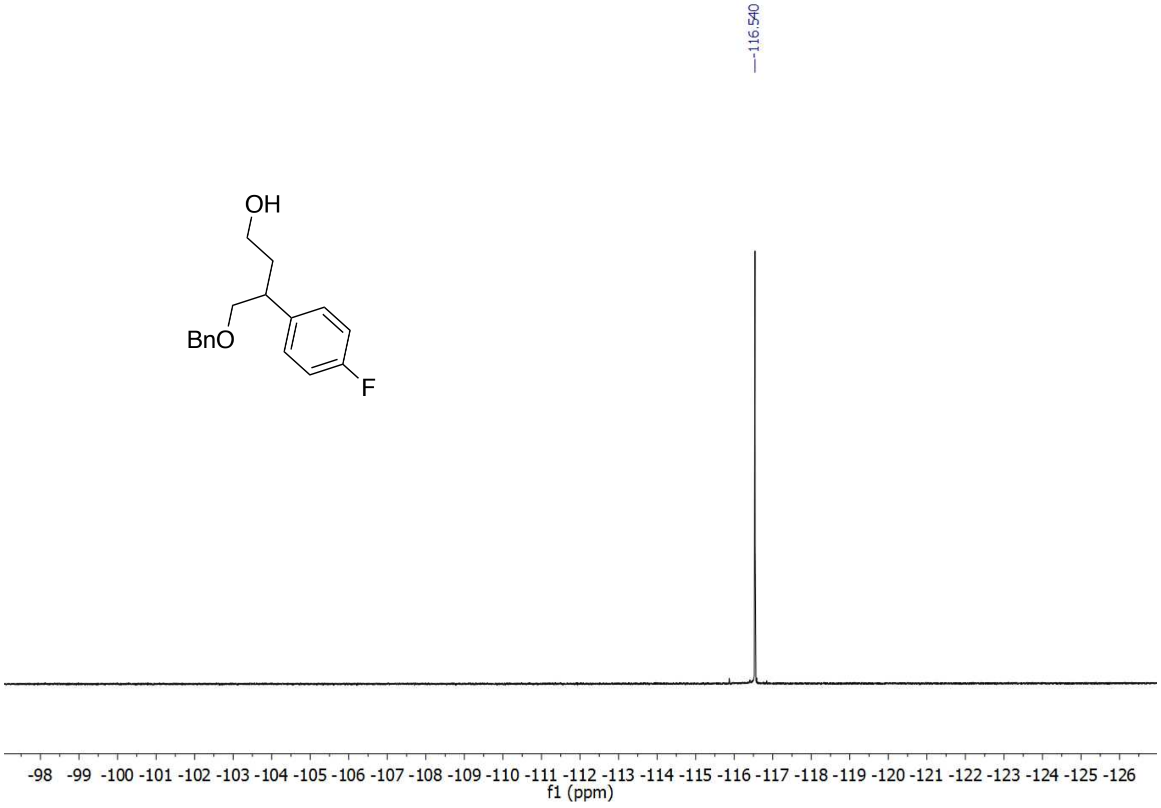

Compound 3a. Top: ^{1}H NMR ($CDCl_3$, 600 MHz). Bottom: ^{13}C NMR ($CDCl_3$, 150 MHz)

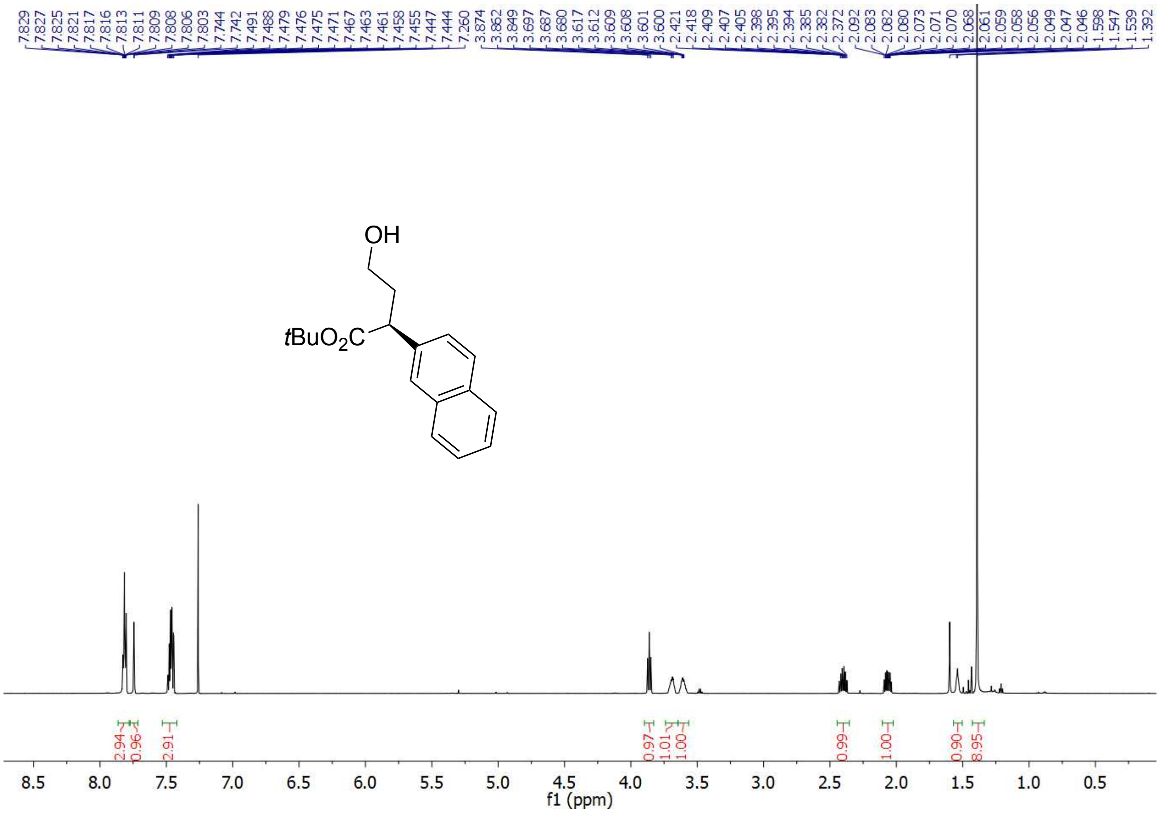

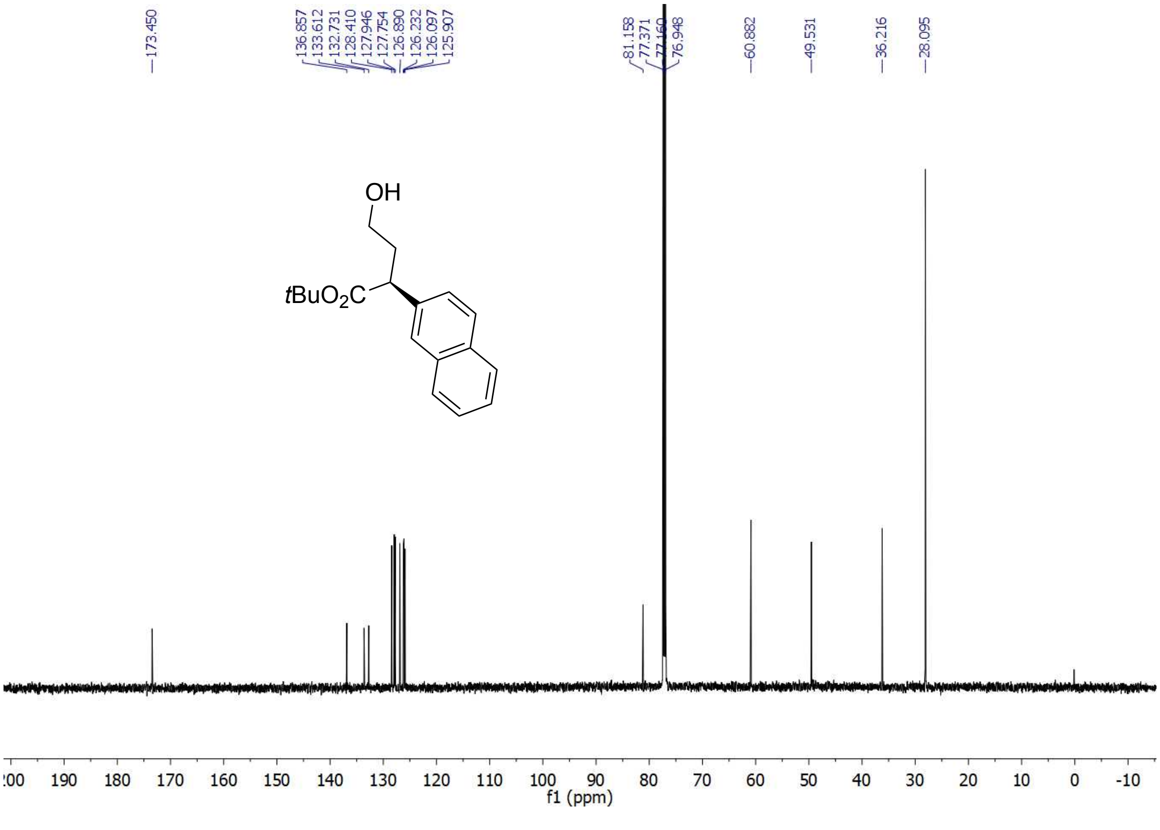

Compound 3b. Top: ^{1}H NMR ($CDCl_3$, 600 MHz). Bottom: ^{13}C NMR ($CDCl_3$, 150 MHz)

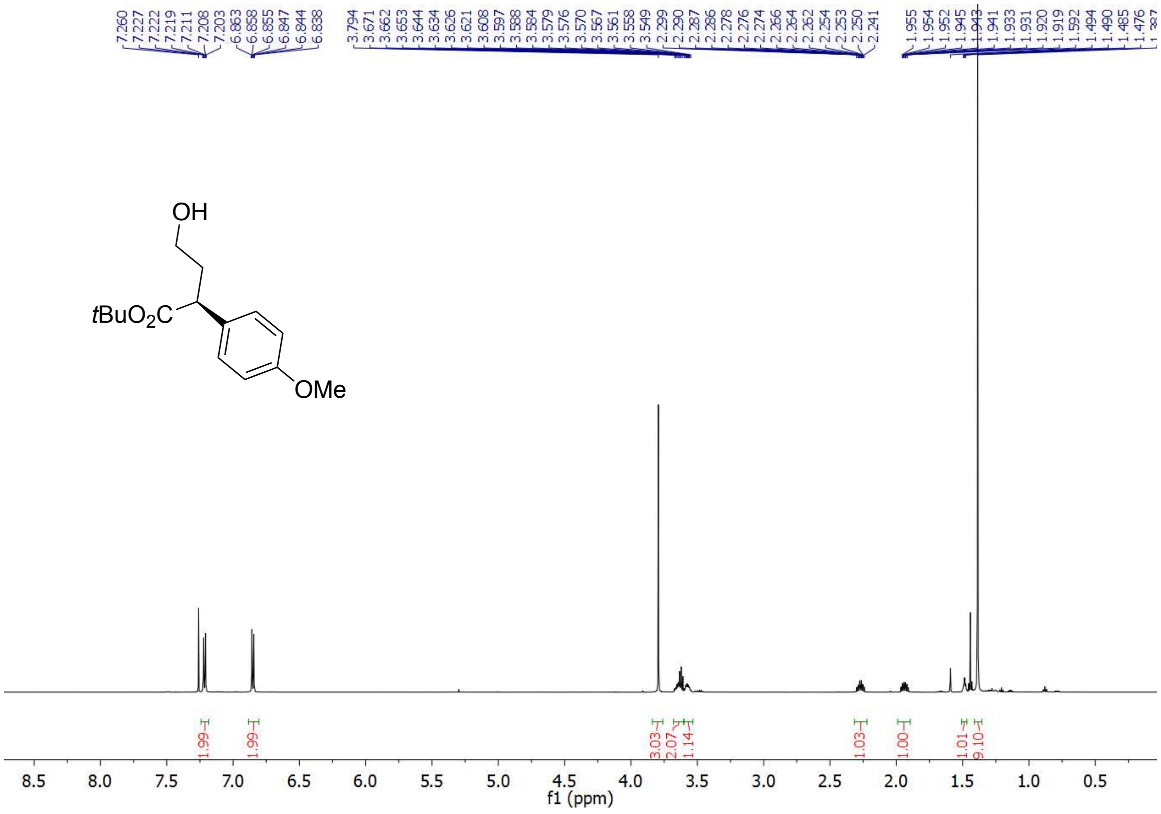

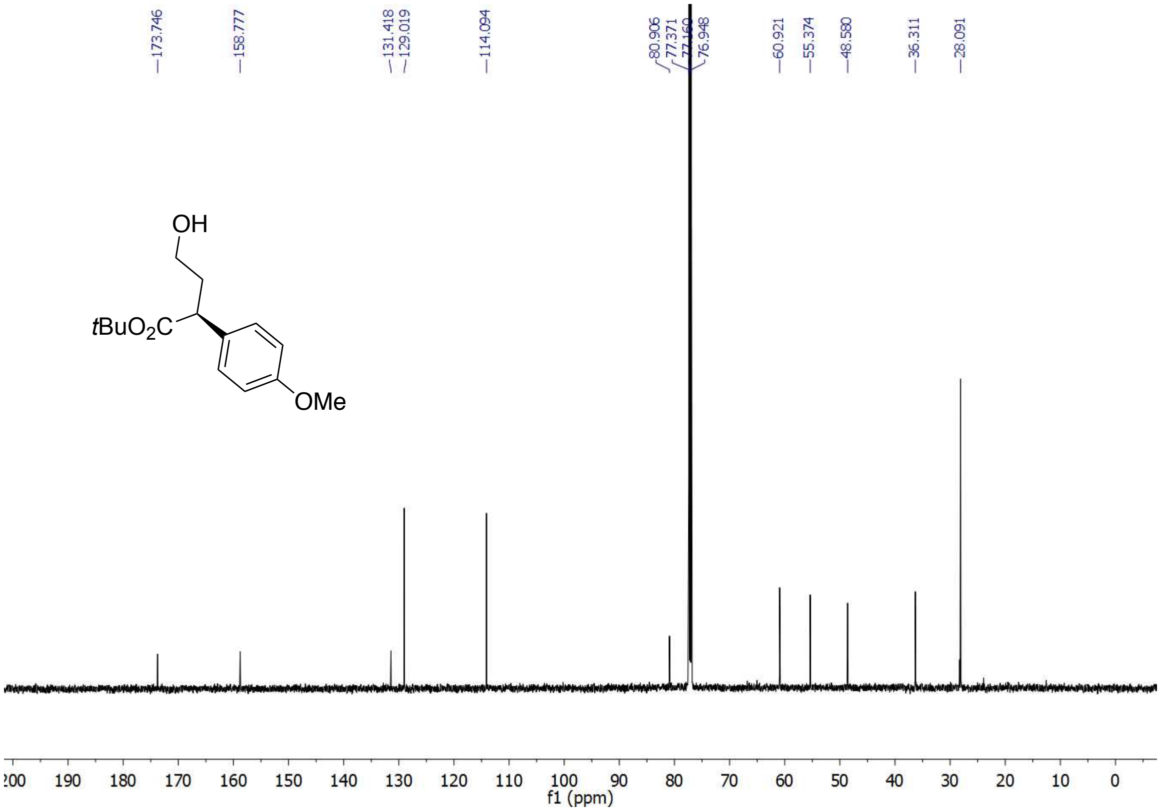

Compound 3c. Top: ^{1}H NMR ($CDCl_3$, 600 MHz). Bottom: ^{13}C NMR ($CDCl_3$, 150 MHz)

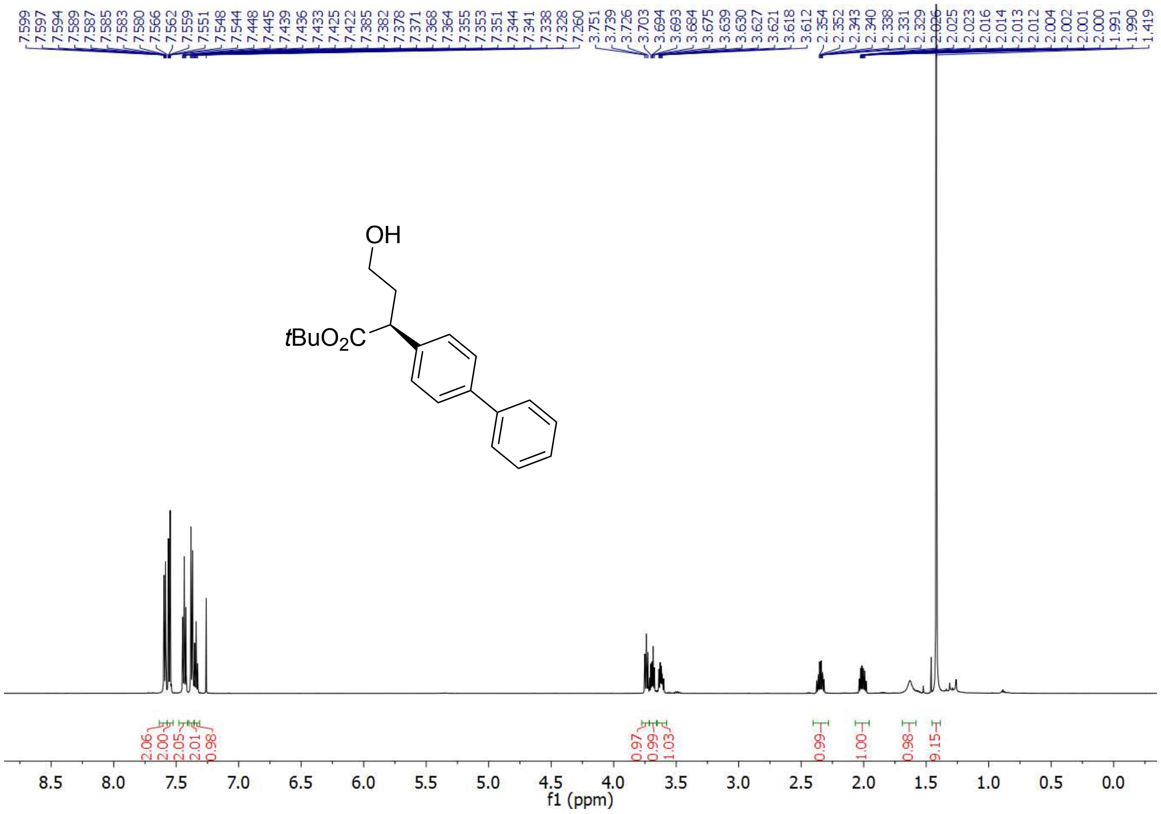

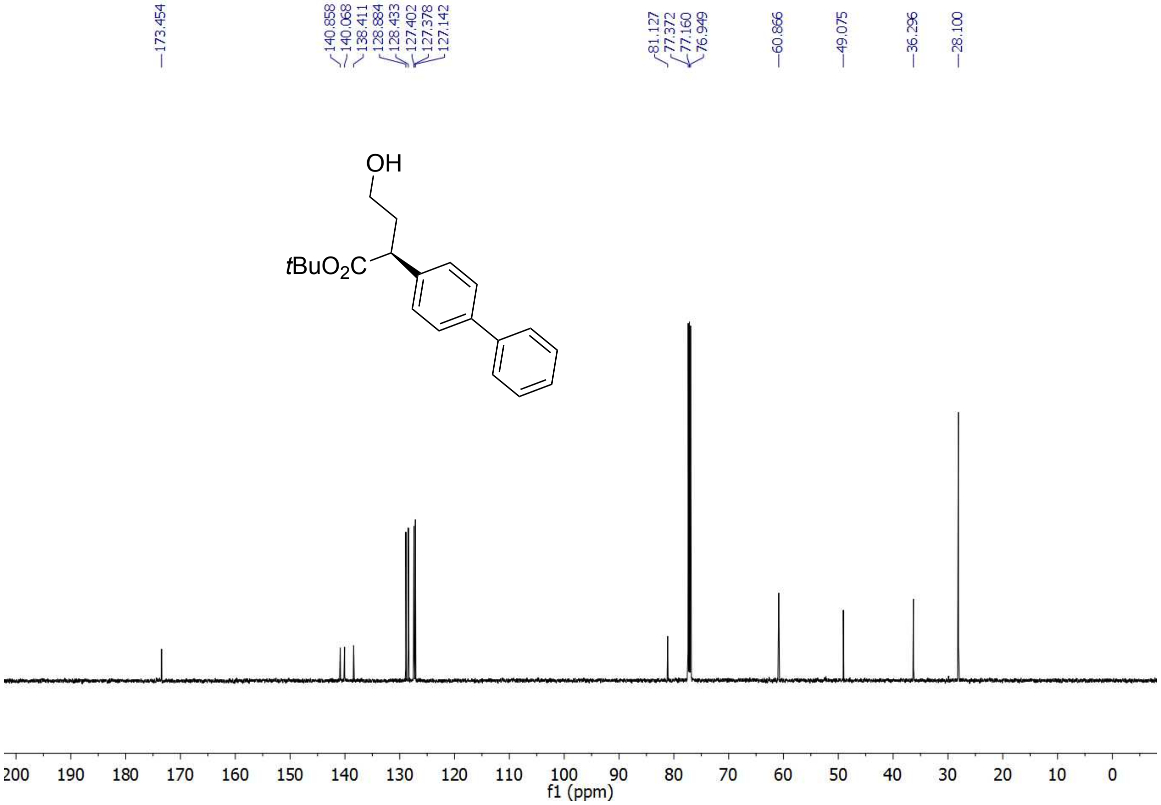

Compound 3d. Top: ^{1}H NMR ($CDCl_3$, 600 MHz). Bottom: ^{13}C NMR ($CDCl_3$, 150 MHz)

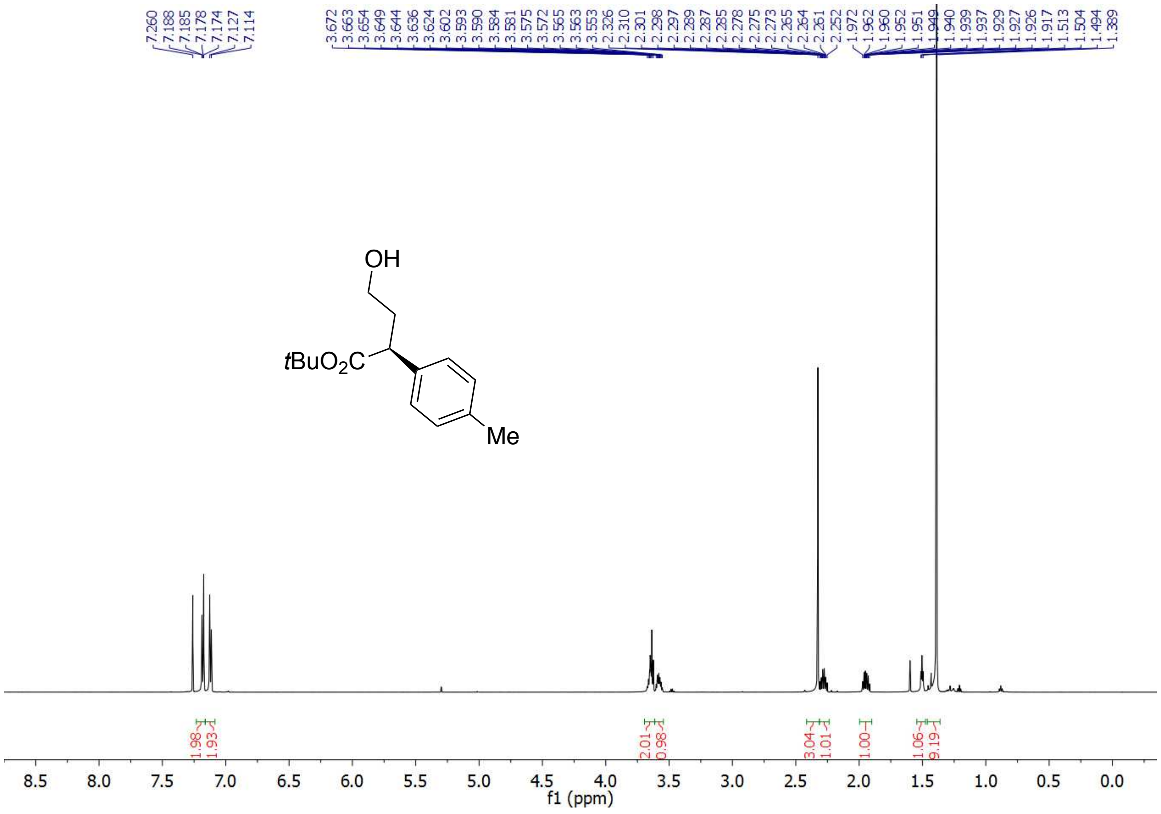

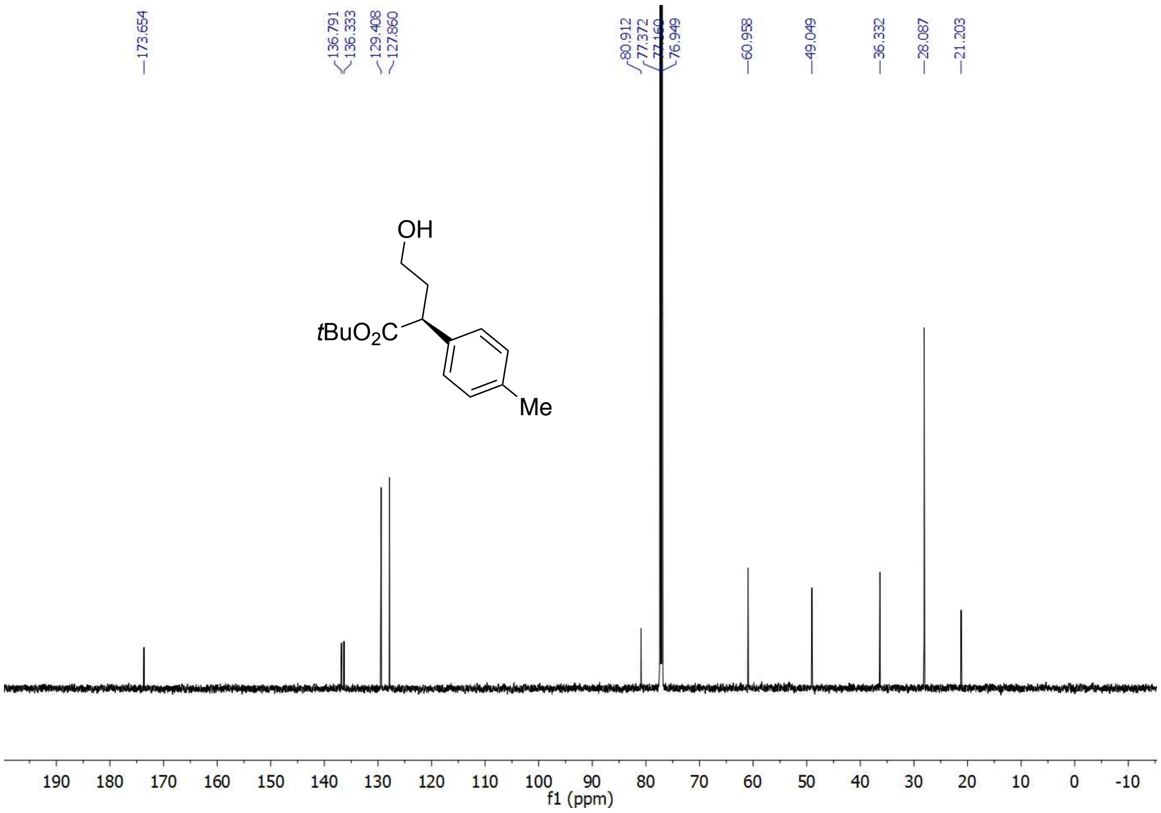

Compound 3e. Top: ^{1}H NMR ($CDCl_3$, 600 MHz). Bottom: ^{13}C NMR ($CDCl_3$, 150 MHz)

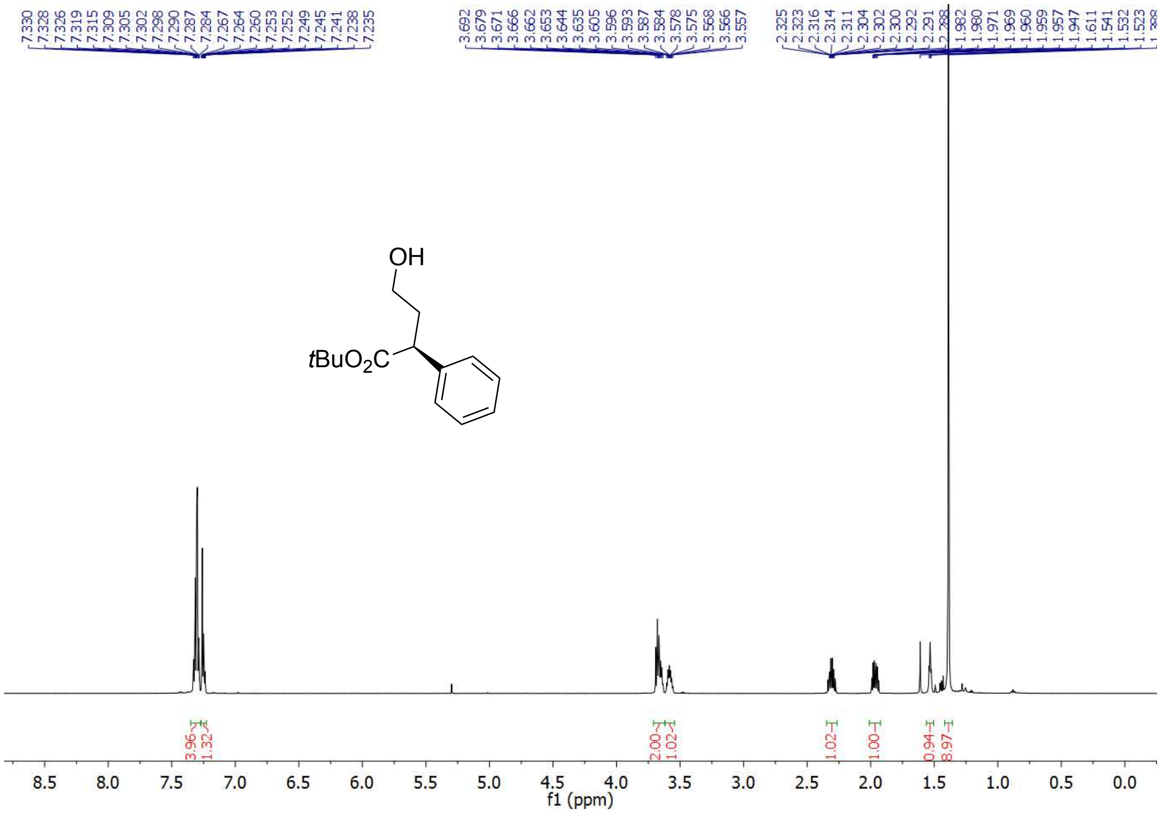

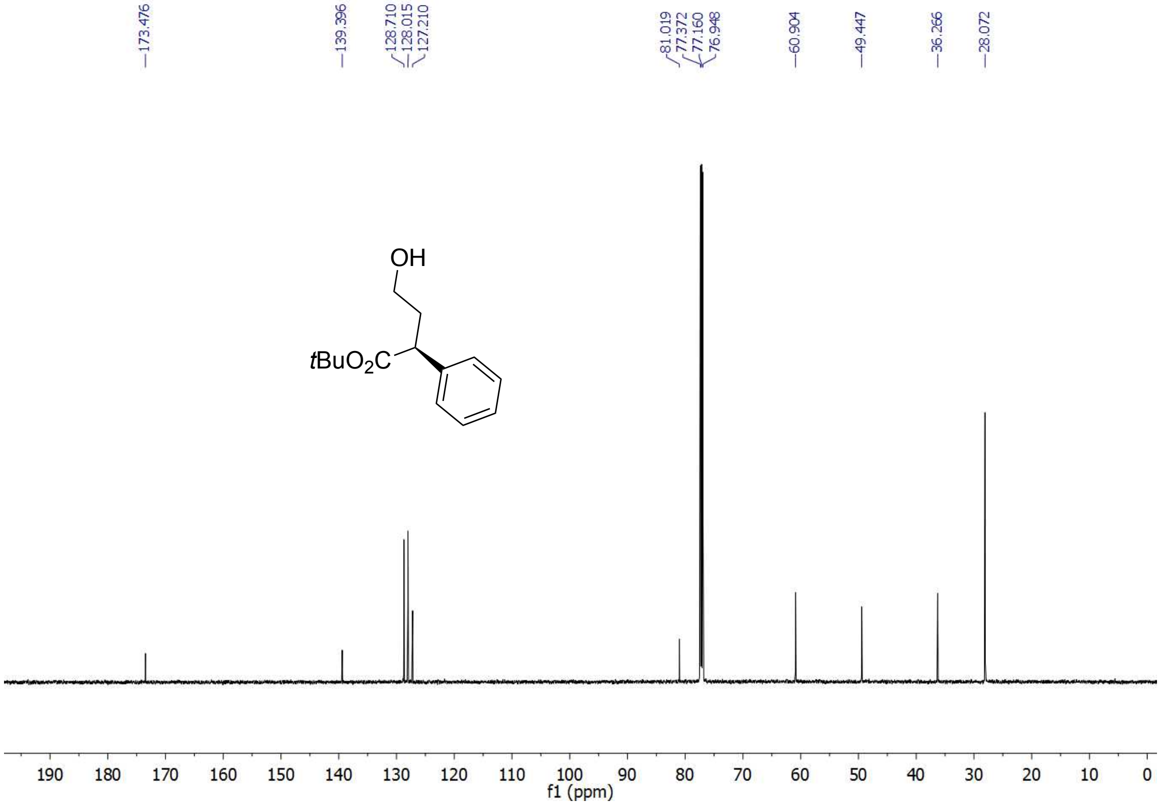

Compound 3f. Top: ^{1}H NMR ($CDCl_3$, 600 MHz). Bottom: ^{13}C NMR ($CDCl_3$, 150 MHz)

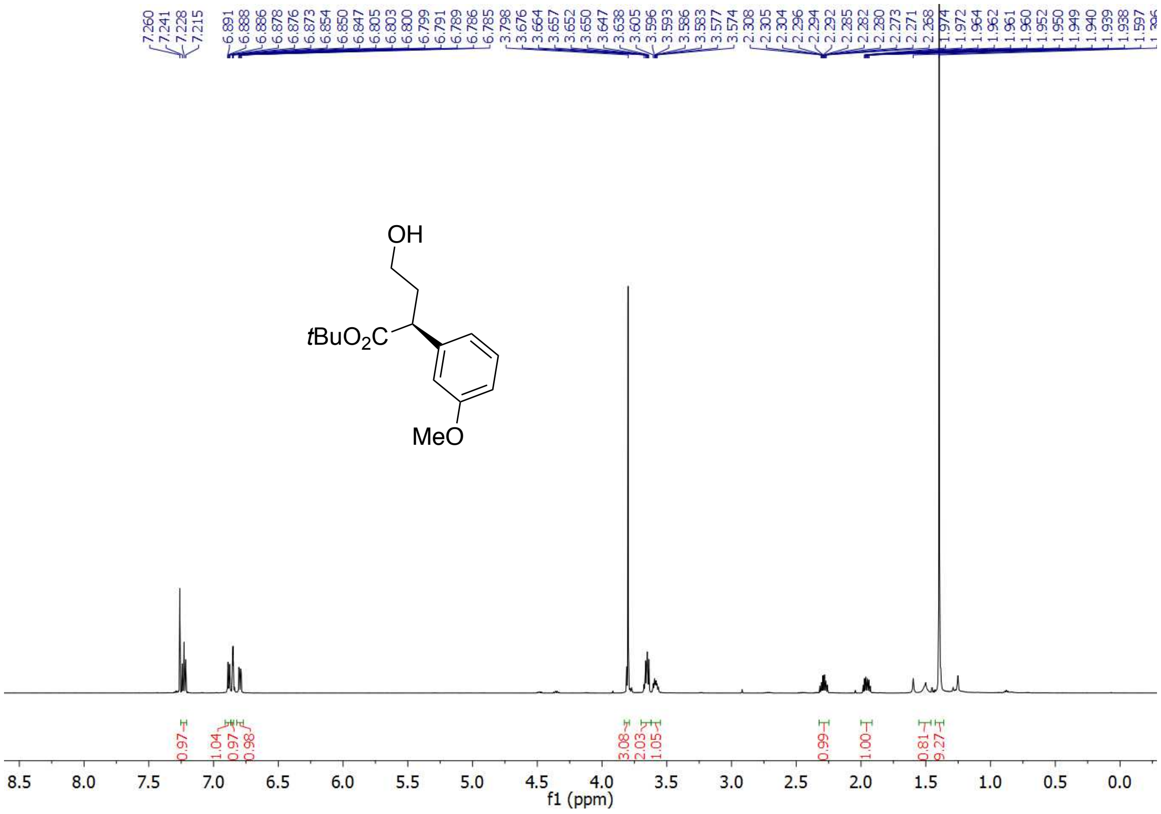

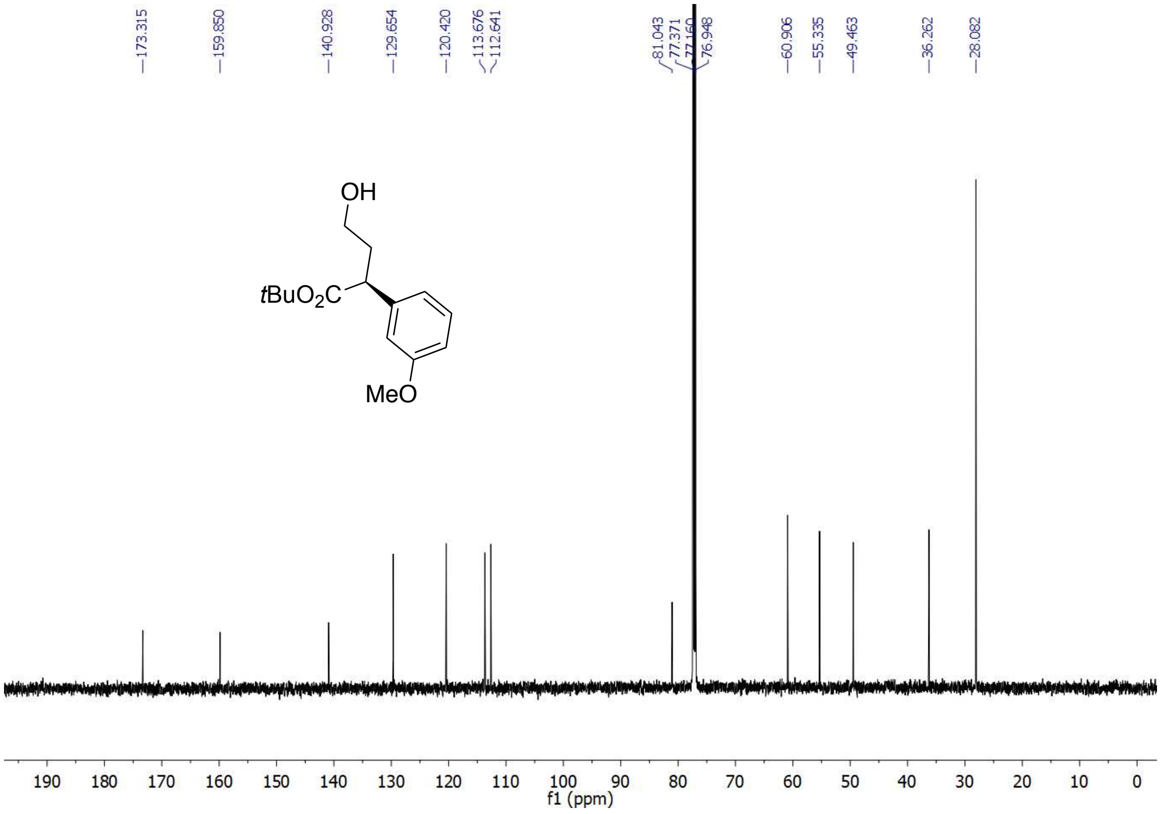

Compound 3g. Top: ^{1}H NMR ($CDCl_3$, 600 MHz). Bottom: ^{13}C NMR ($CDCl_3$, 150 MHz)

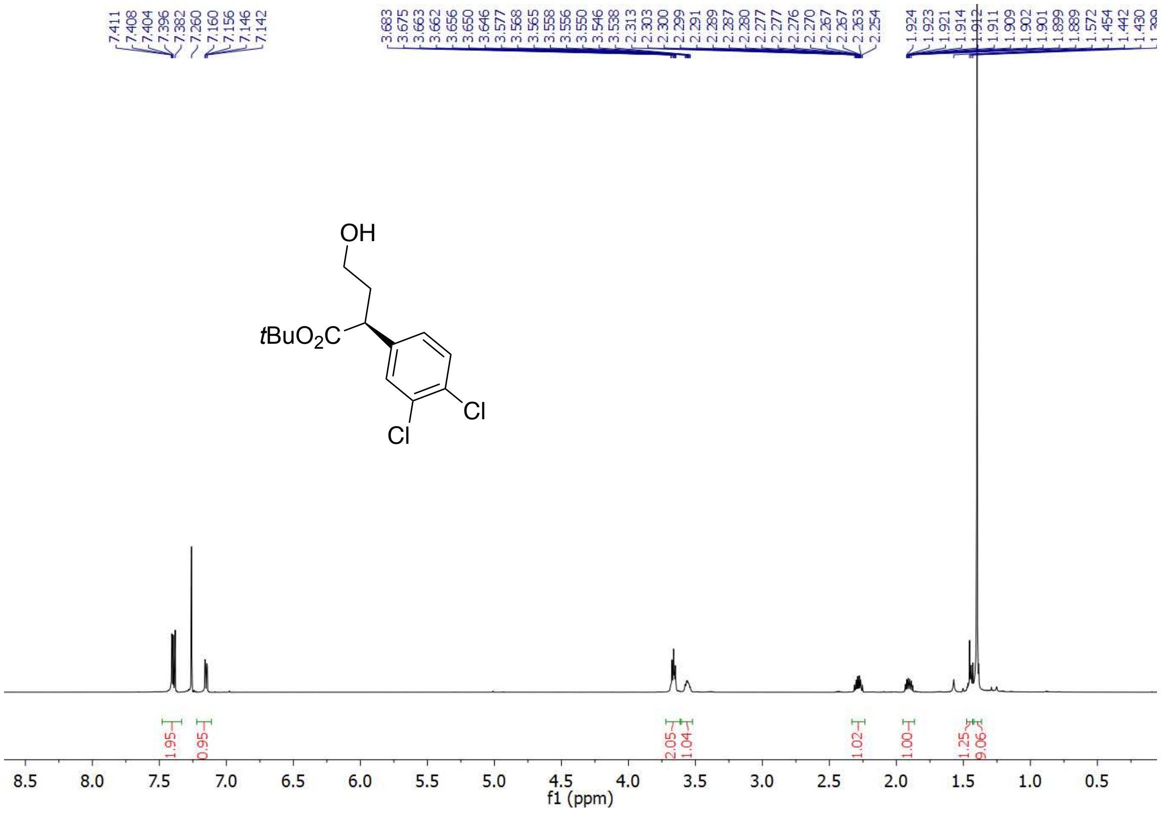

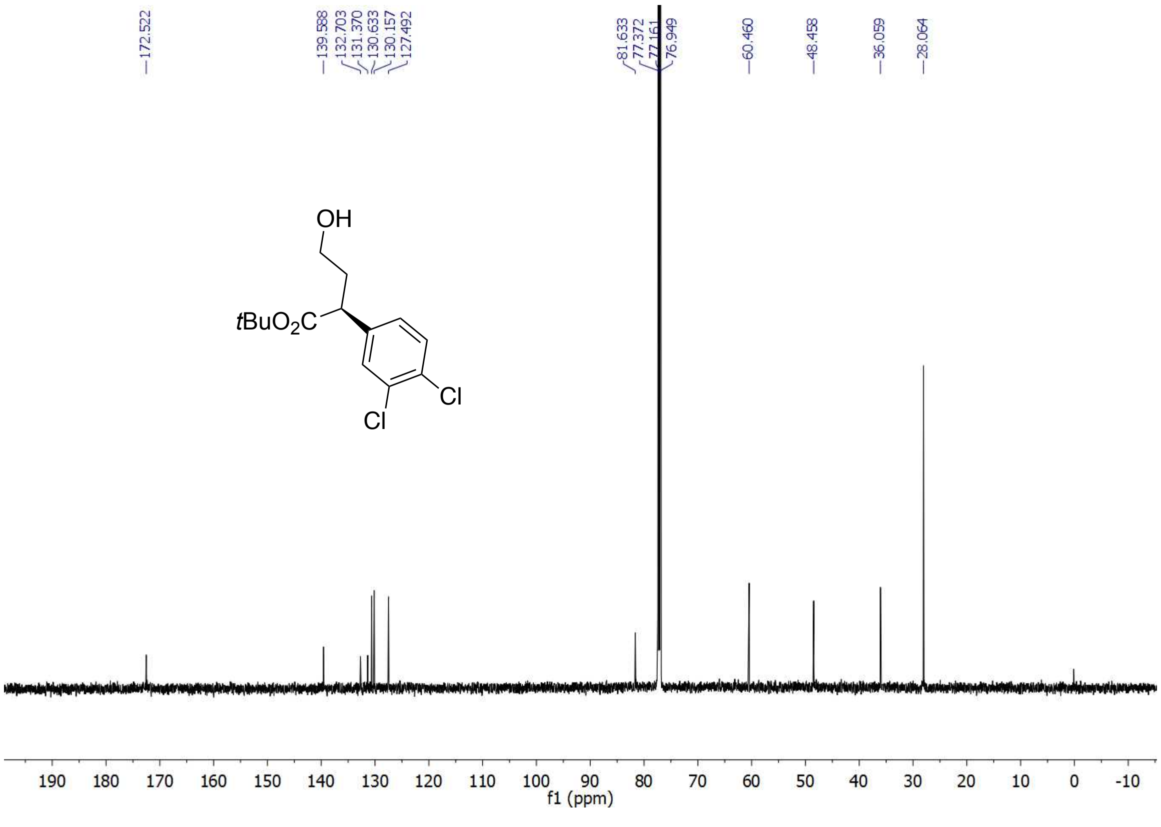

Compound 3h. Top: ^{1}H NMR ($CDCl_3$, 600 MHz). Bottom: ^{13}C NMR ($CDCl_3$, 150 MHz)

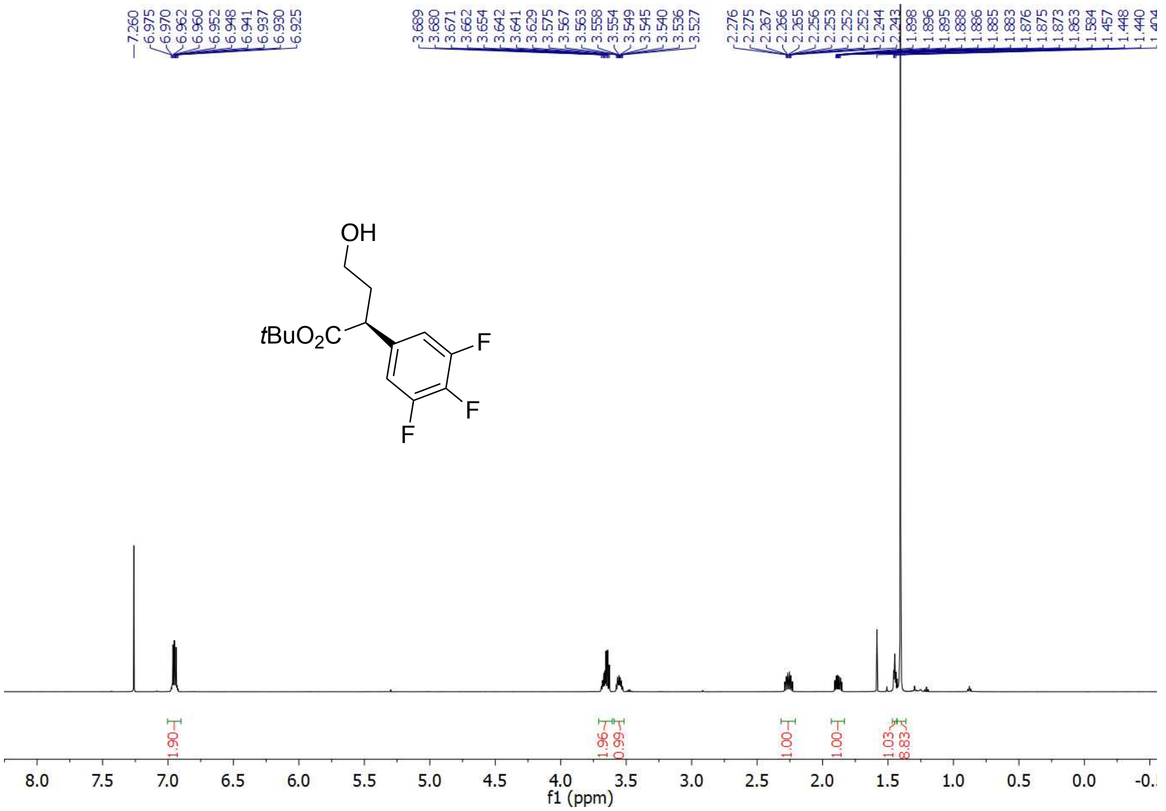

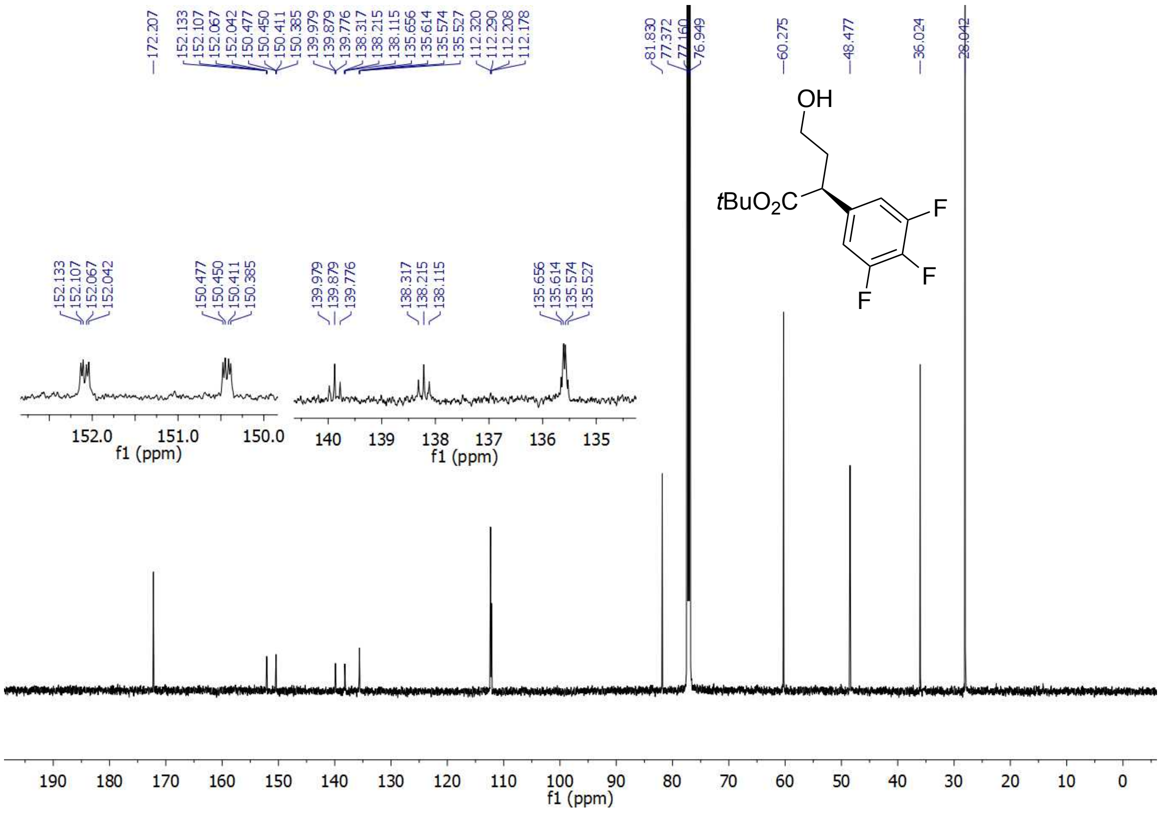

Compound 3h. ^{19}F NMR ($CDCl_3$, 565 MHz).

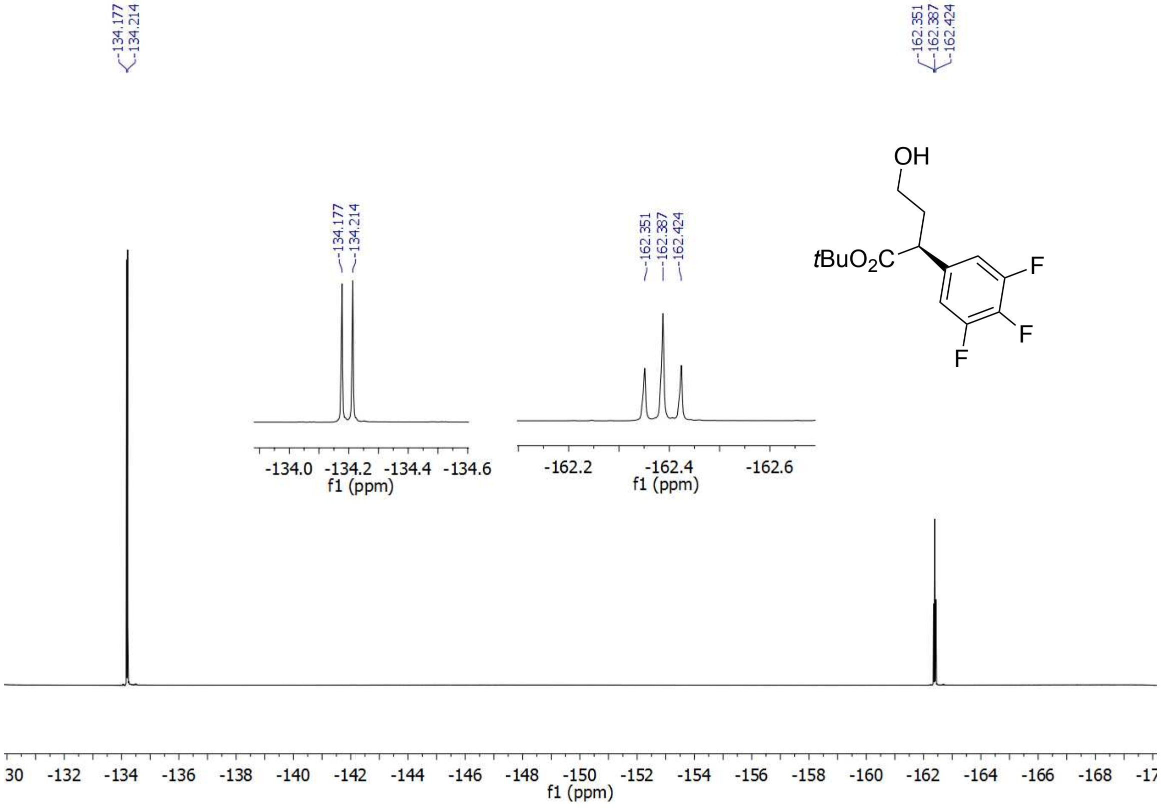

Compound 3i. Top: ^{1}H NMR ($CDCl_3$, 600 MHz). Bottom: ^{13}C NMR ($CDCl_3$, 150 MHz)

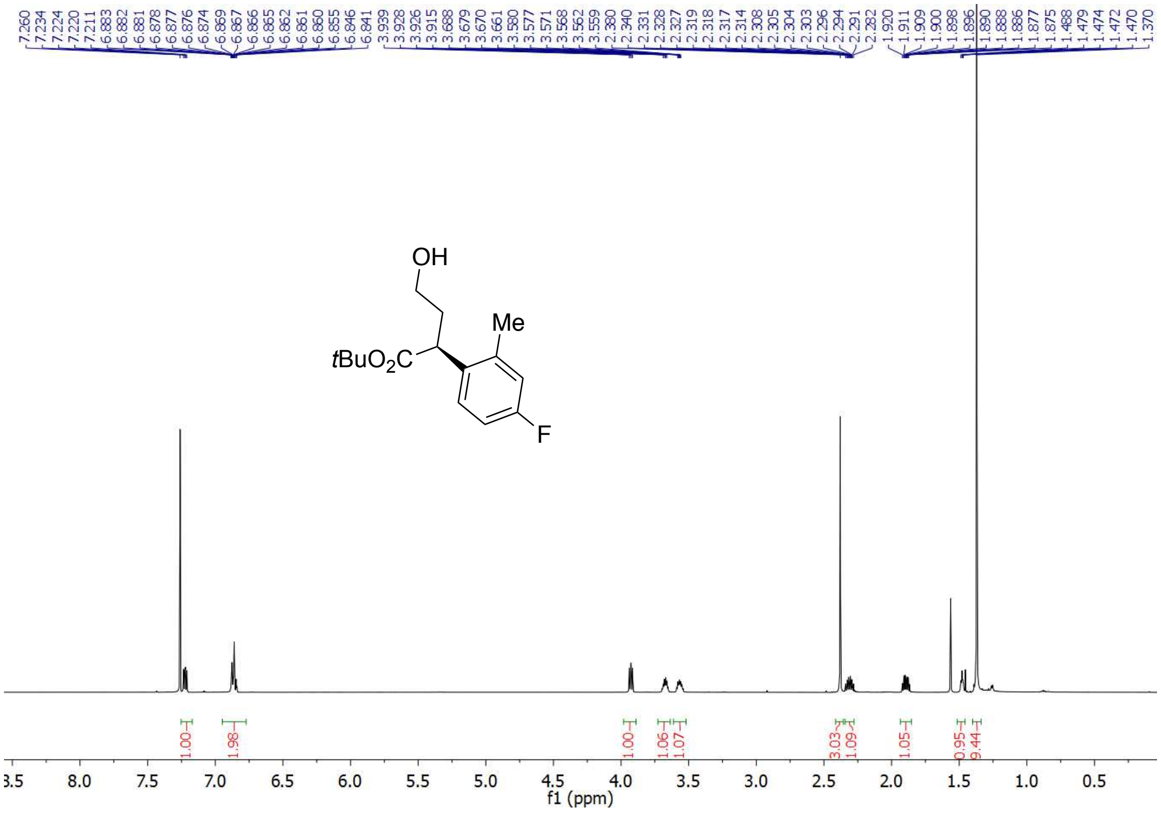

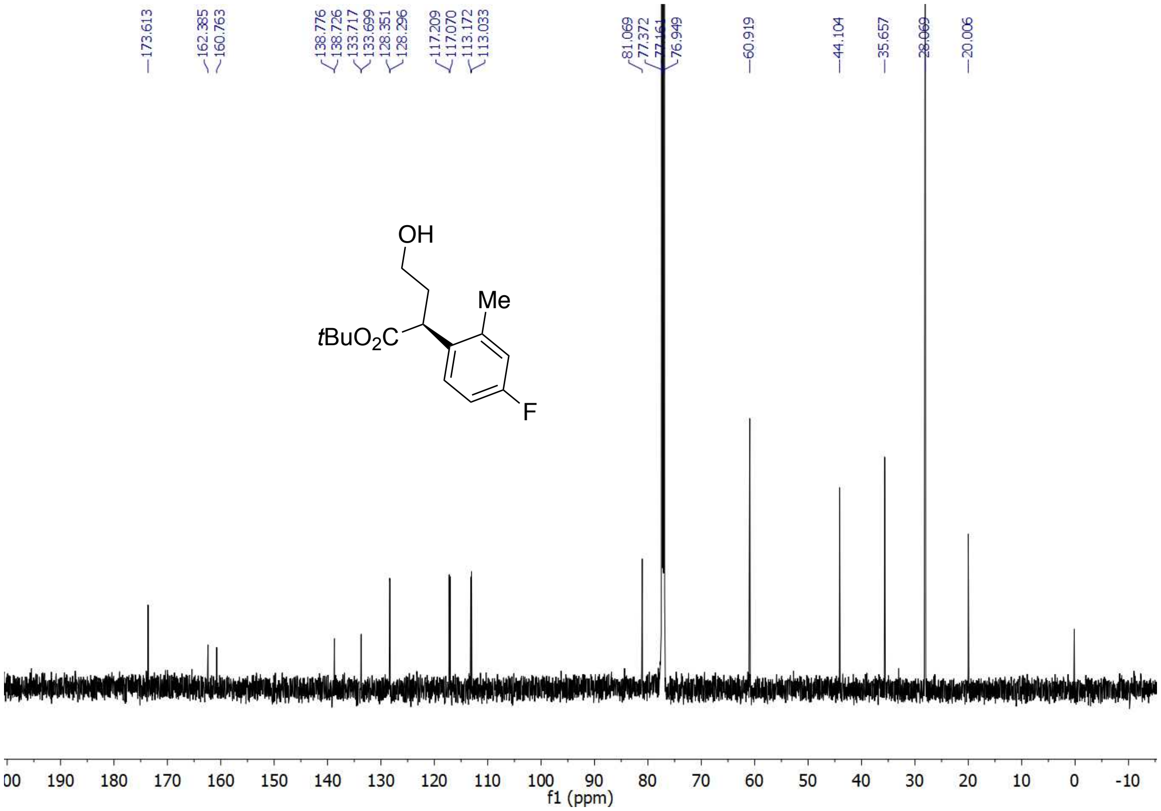

Compound 3i. ^{19}F NMR ($CDCl_3$, 565 MHz).

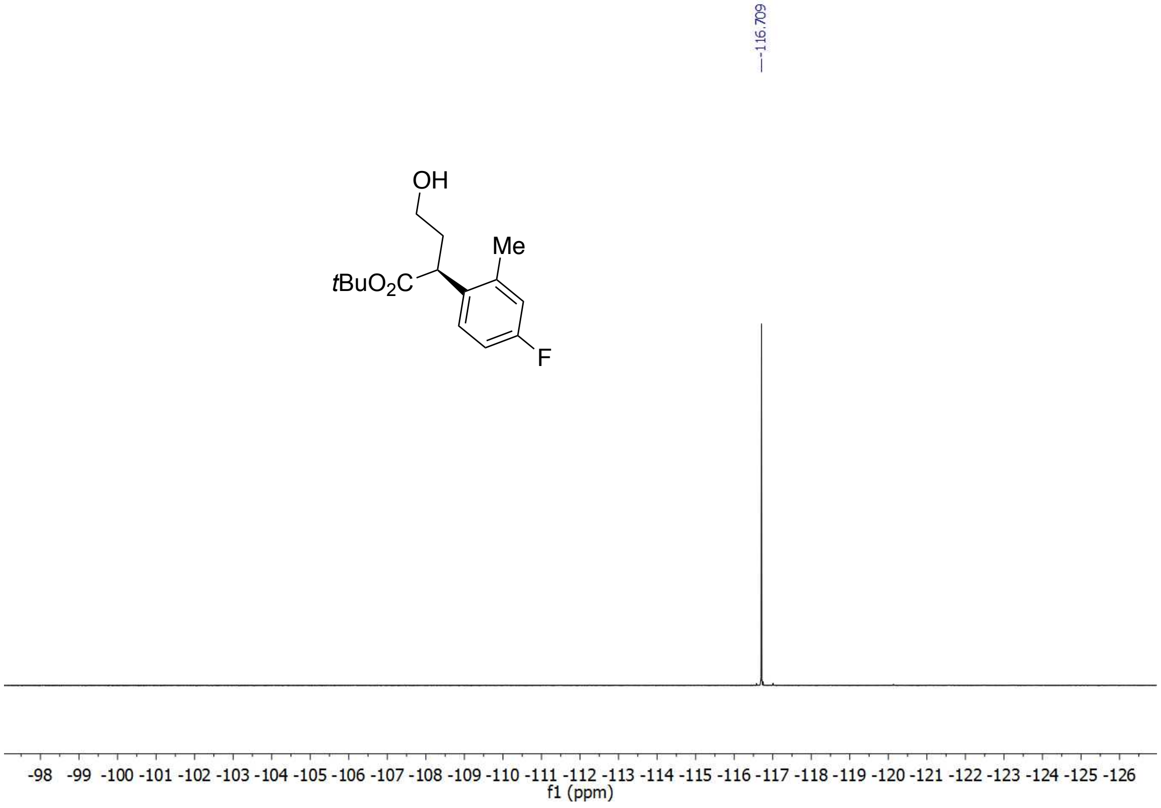

Compound 3j. Top: ^{1}H NMR ($CDCl_3$, 600 MHz). Bottom: ^{13}C NMR ($CDCl_3$, 150 MHz)

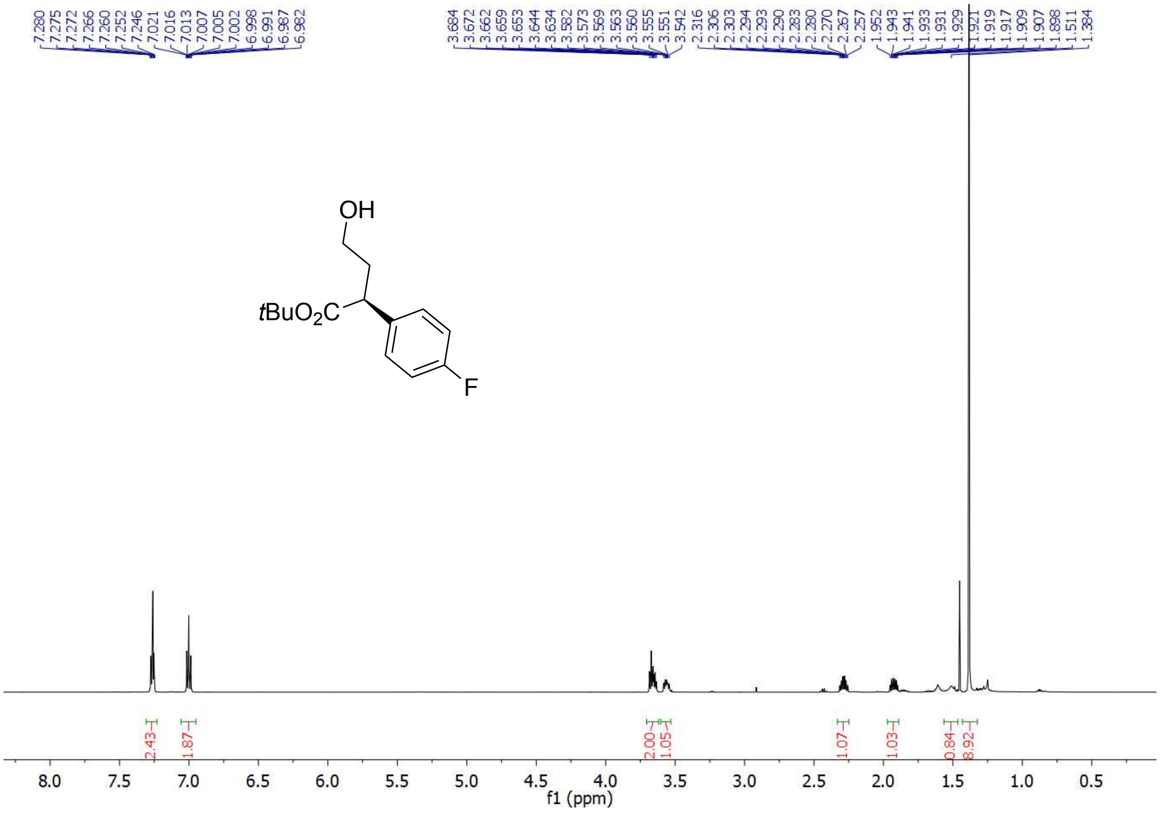

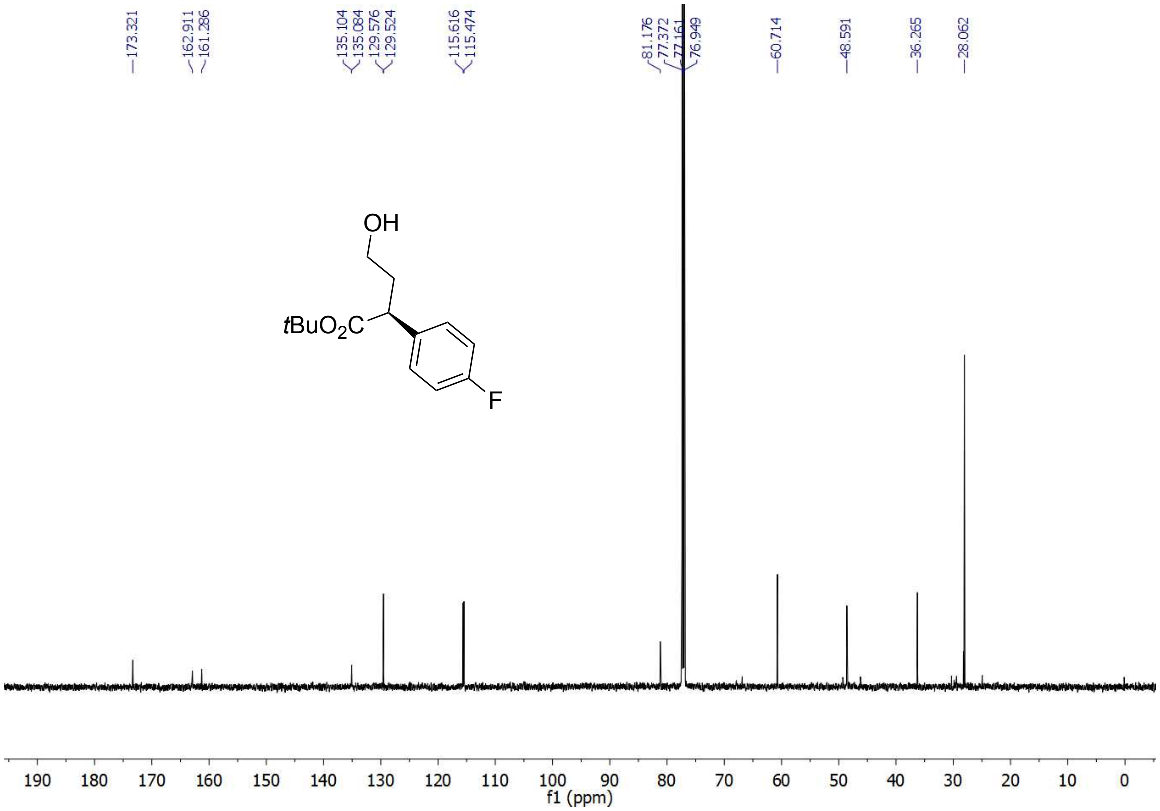

Compound 3j. ^{19}F NMR ($CDCl_3$, 565 MHz).

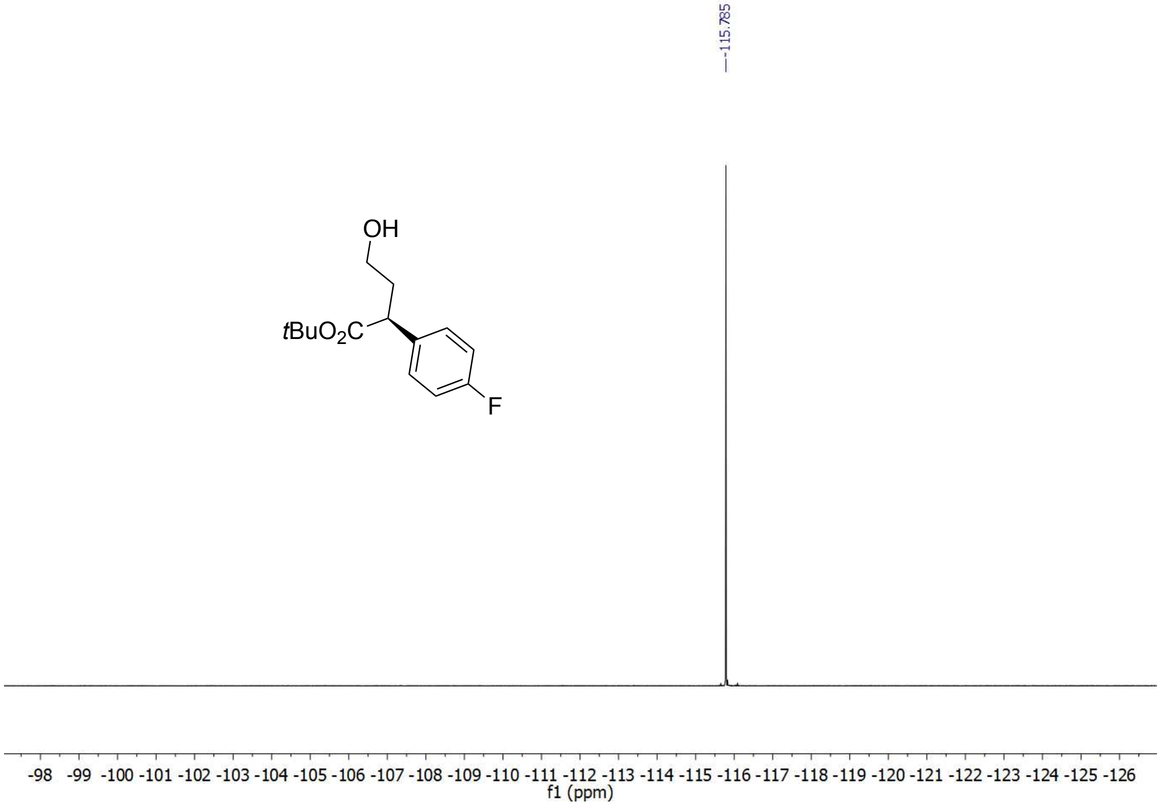

Compound 3k. Top: ^{1}H NMR ($CDCl_3$, 600 MHz). Bottom: ^{13}C NMR ($CDCl_3$, 150 MHz)

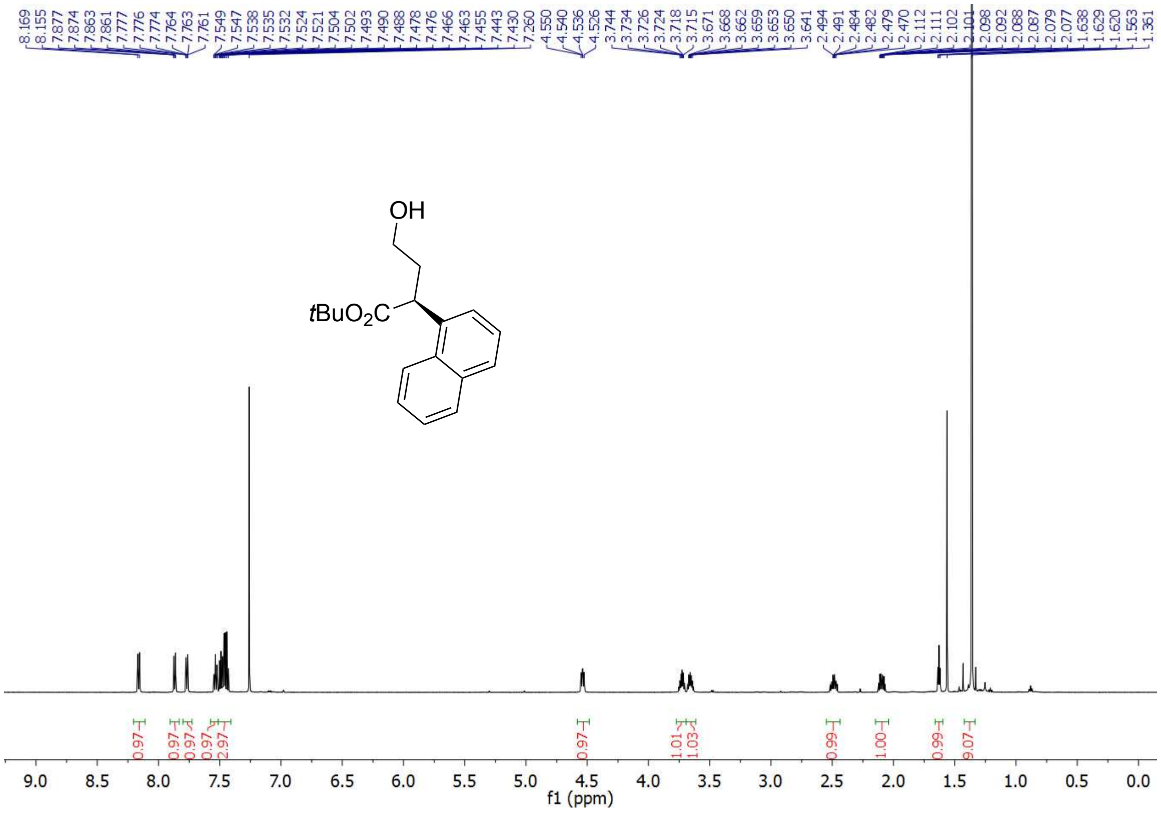

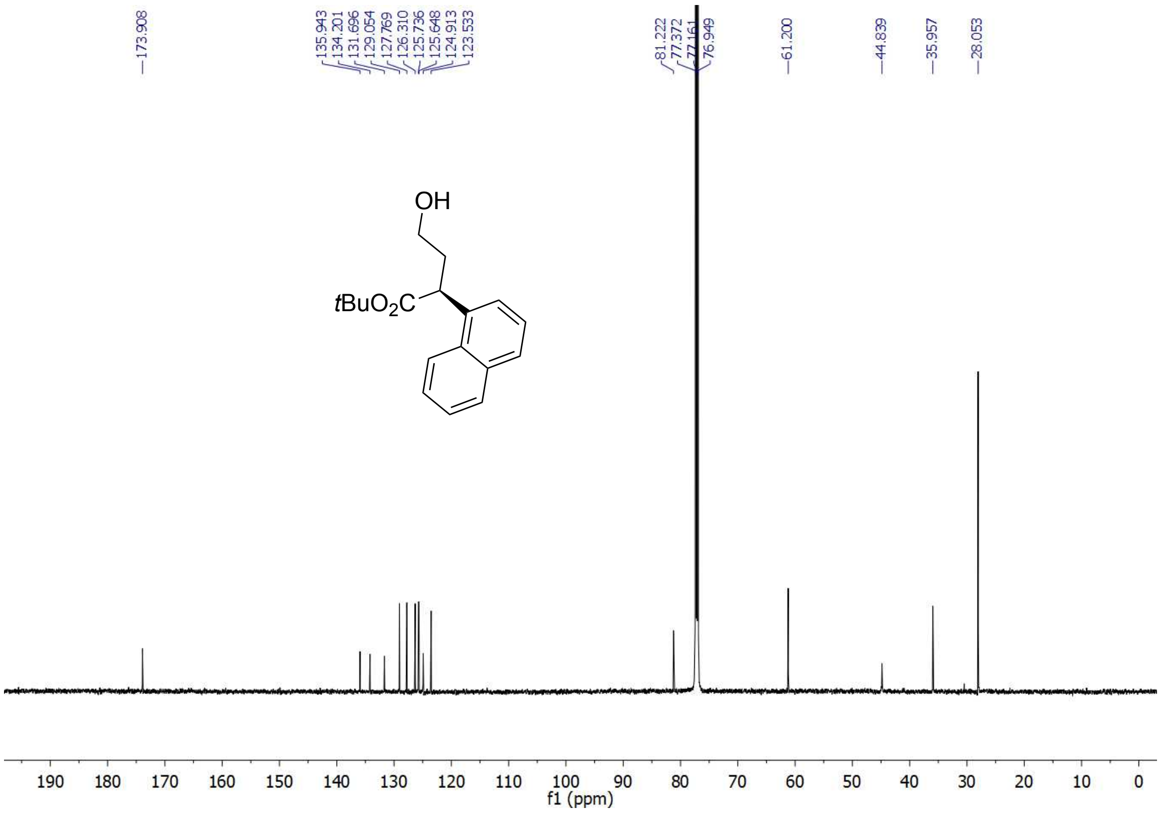

Compound 2l. Top: ^{1}H NMR ($CDCl_3$, 600 MHz). Bottom: ^{13}C NMR ($CDCl_3$, 150 MHz)

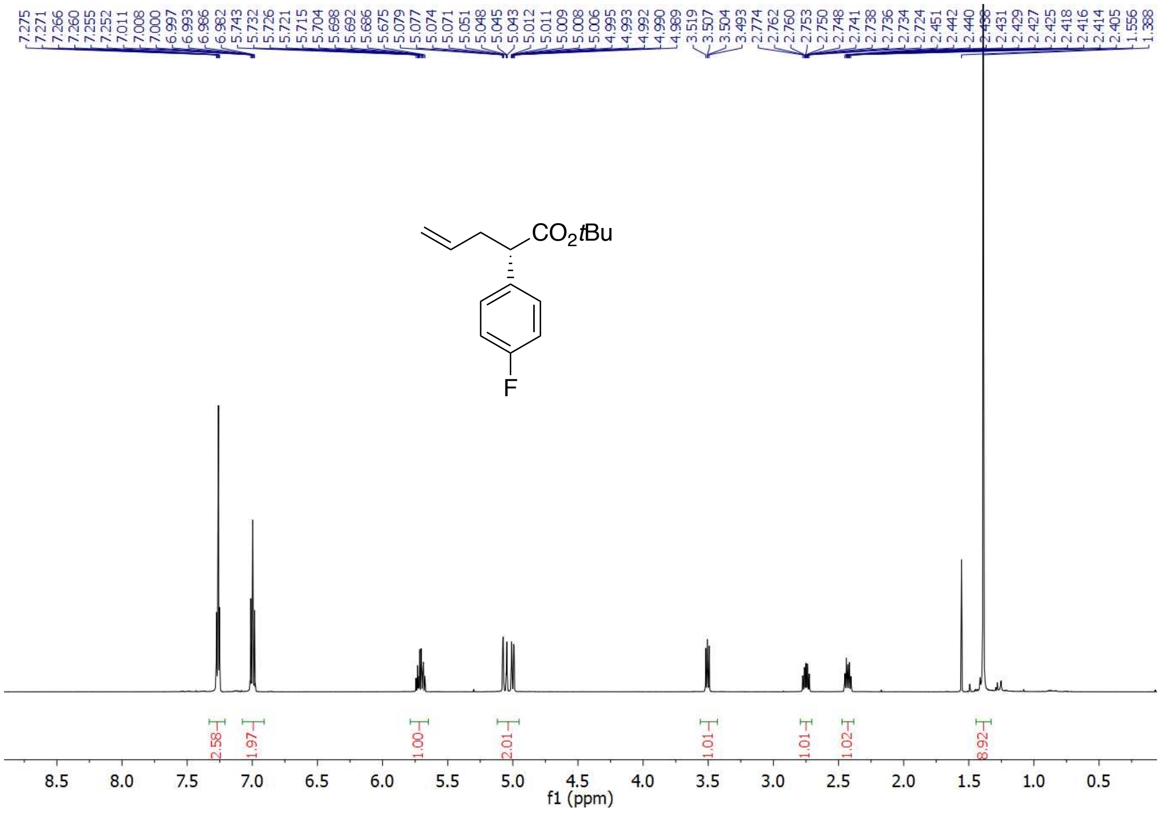

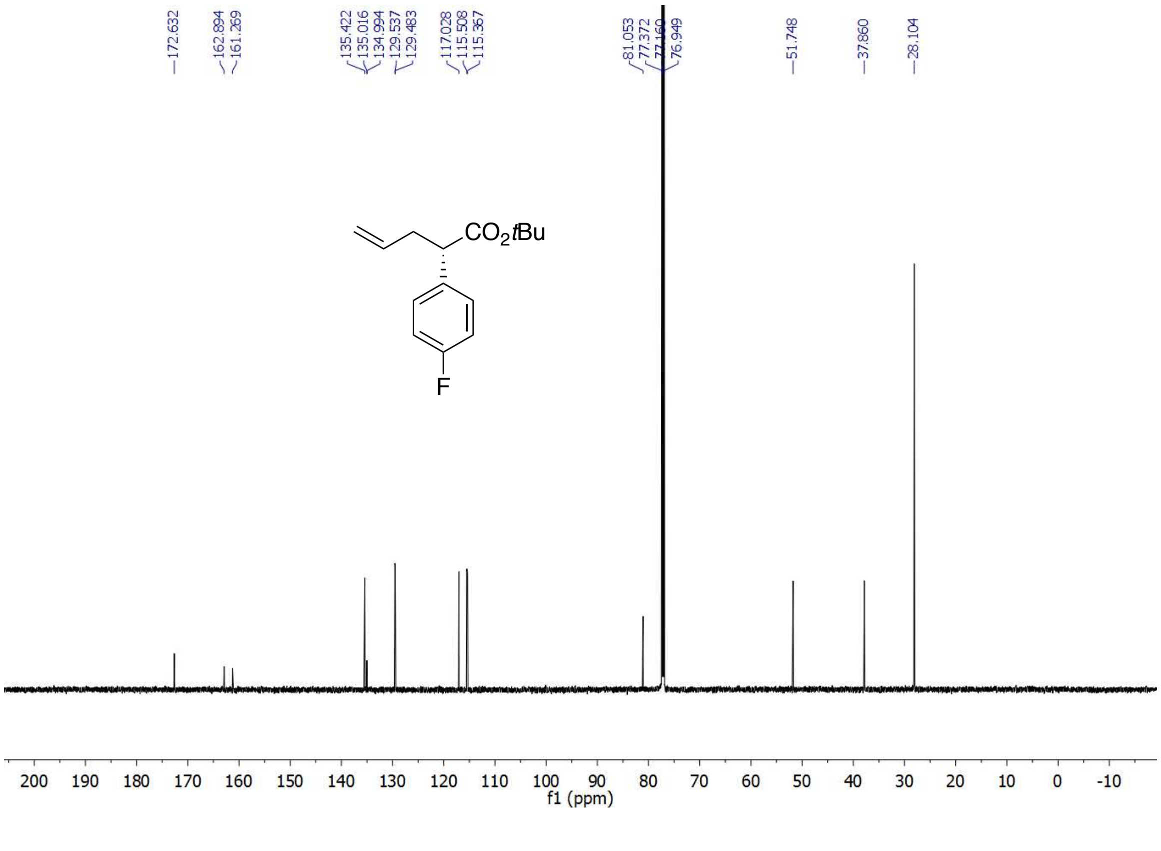

Compound 2l. ^{19}F NMR ($CDCl_3$, 565 MHz).

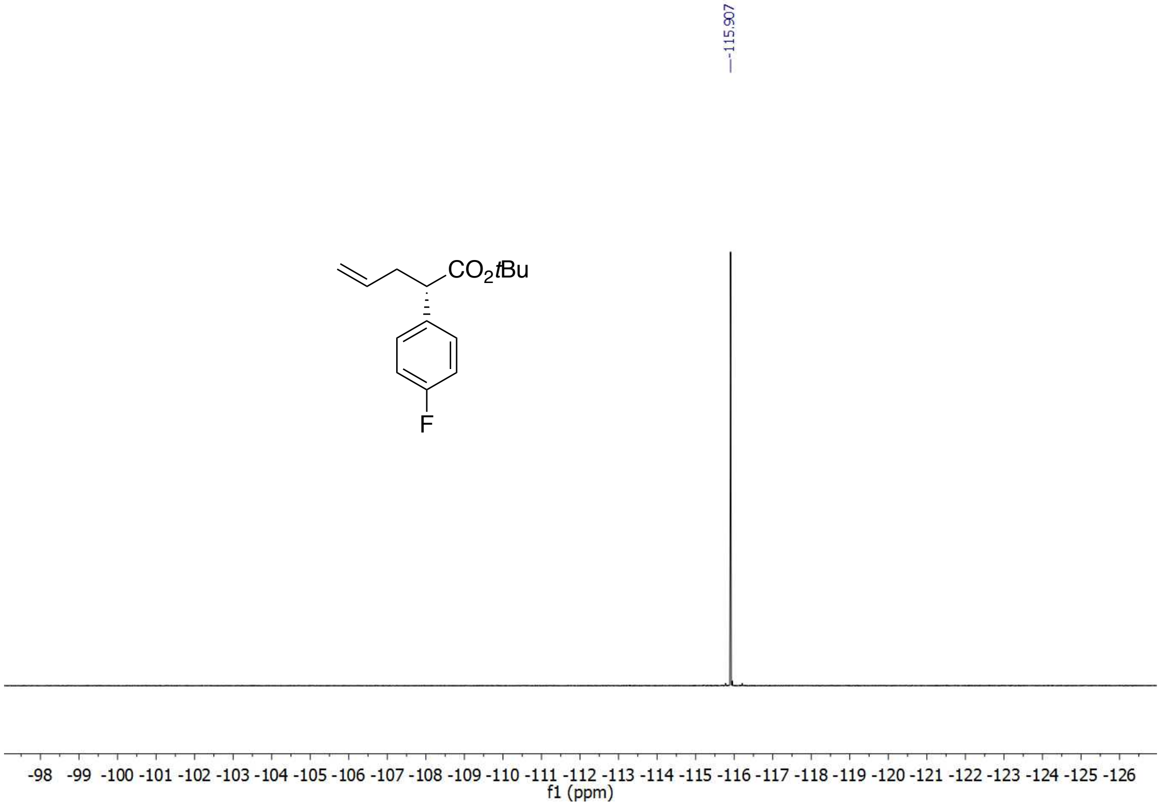

Compound 7a. Top: ^{1}H NMR ($CDCl_3$, 600 MHz). Bottom: ^{13}C NMR ($CDCl_3$, 150 MHz)

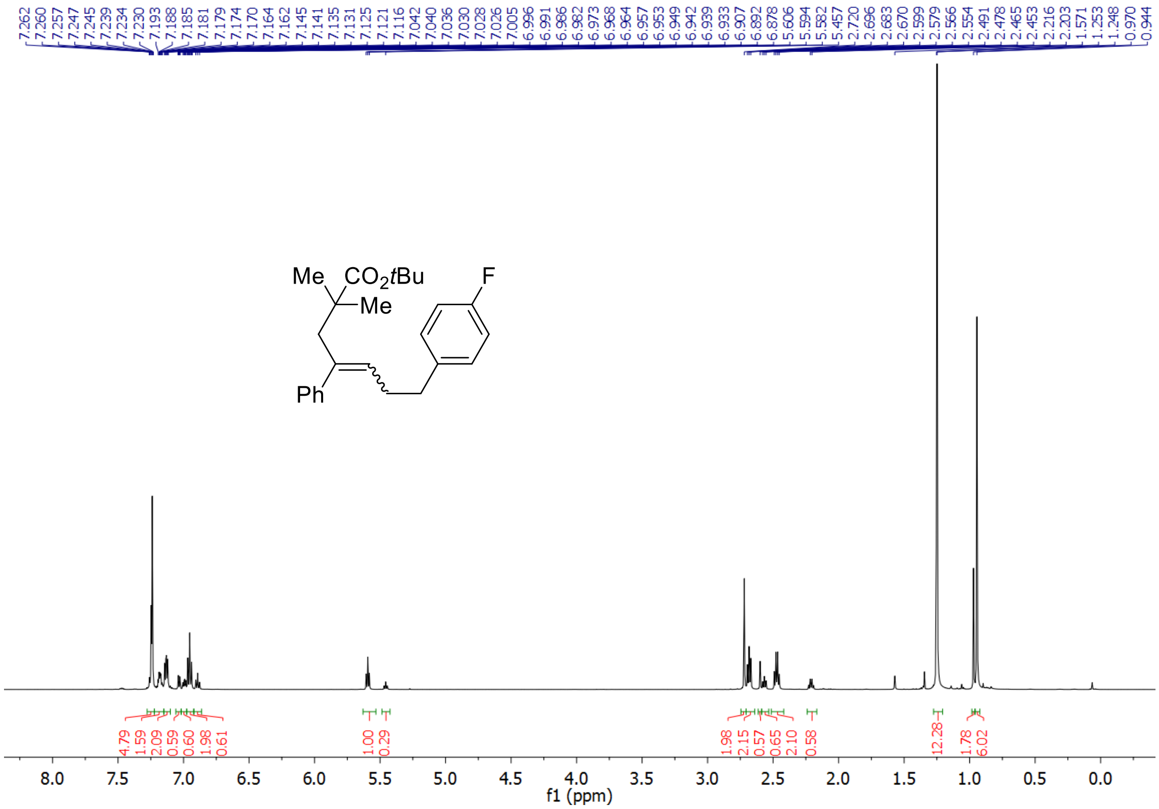

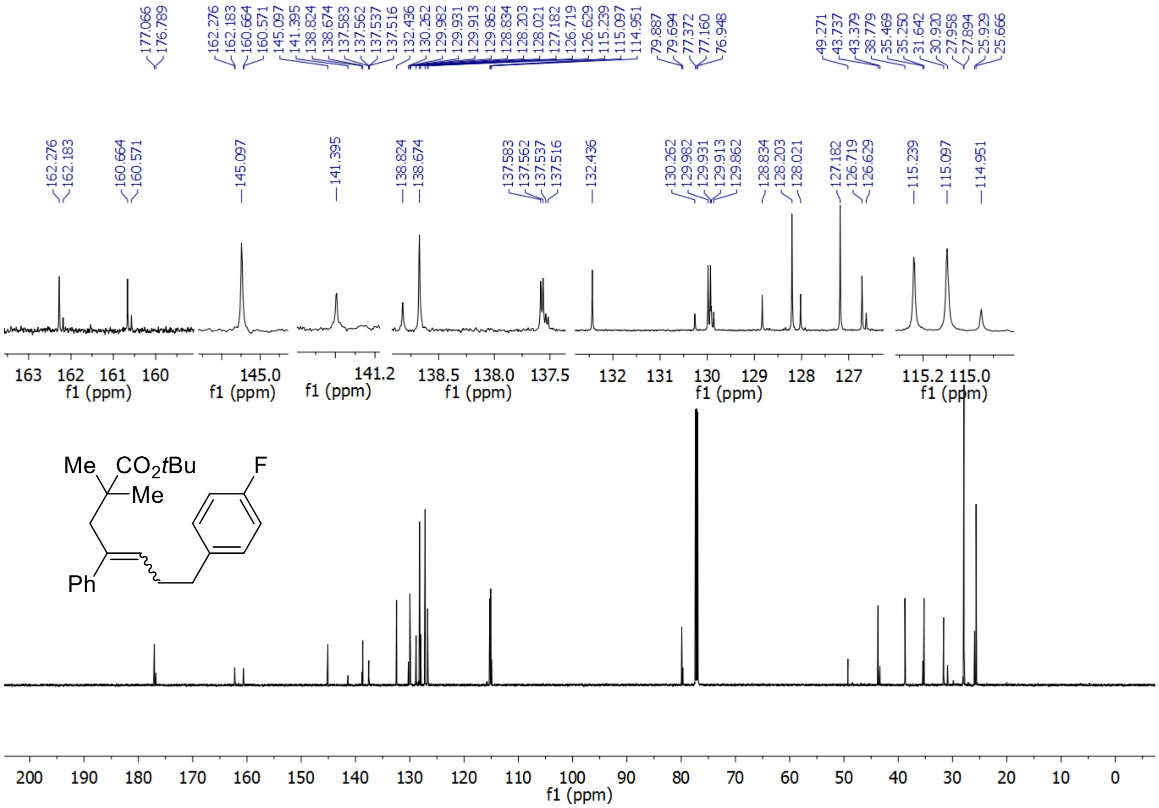

Compound 7a. ^{19}F NMR ($CDCl_3$, 565 MHz).

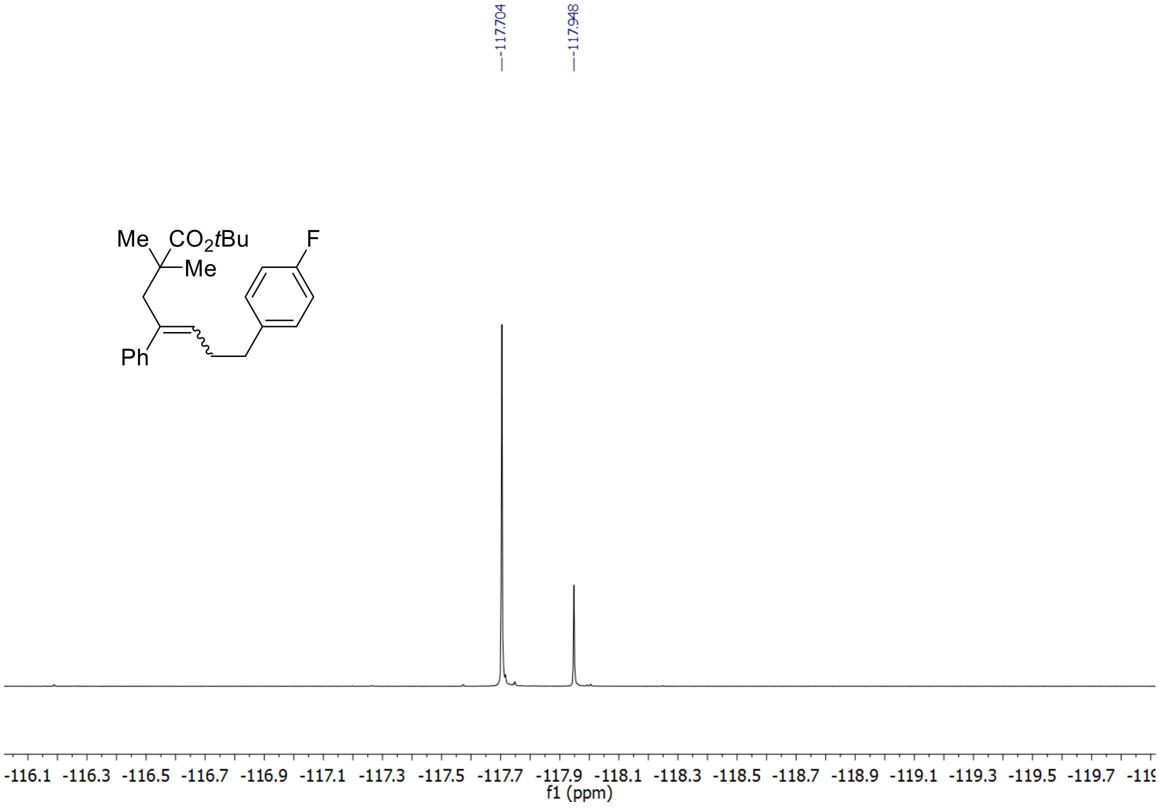

Compound 7b. Top: ^{1}H NMR ($CDCl_3$, 600 MHz). Bottom: ^{13}C NMR ($CDCl_3$, 150 MHz)

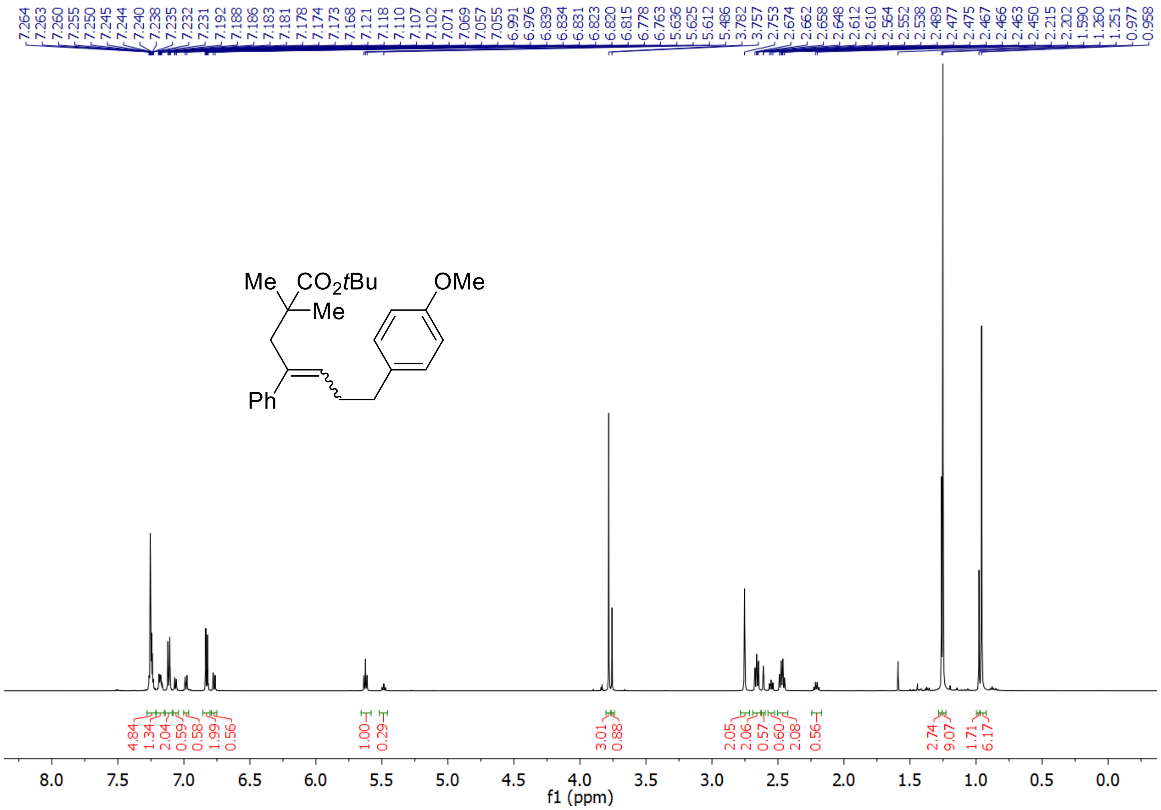

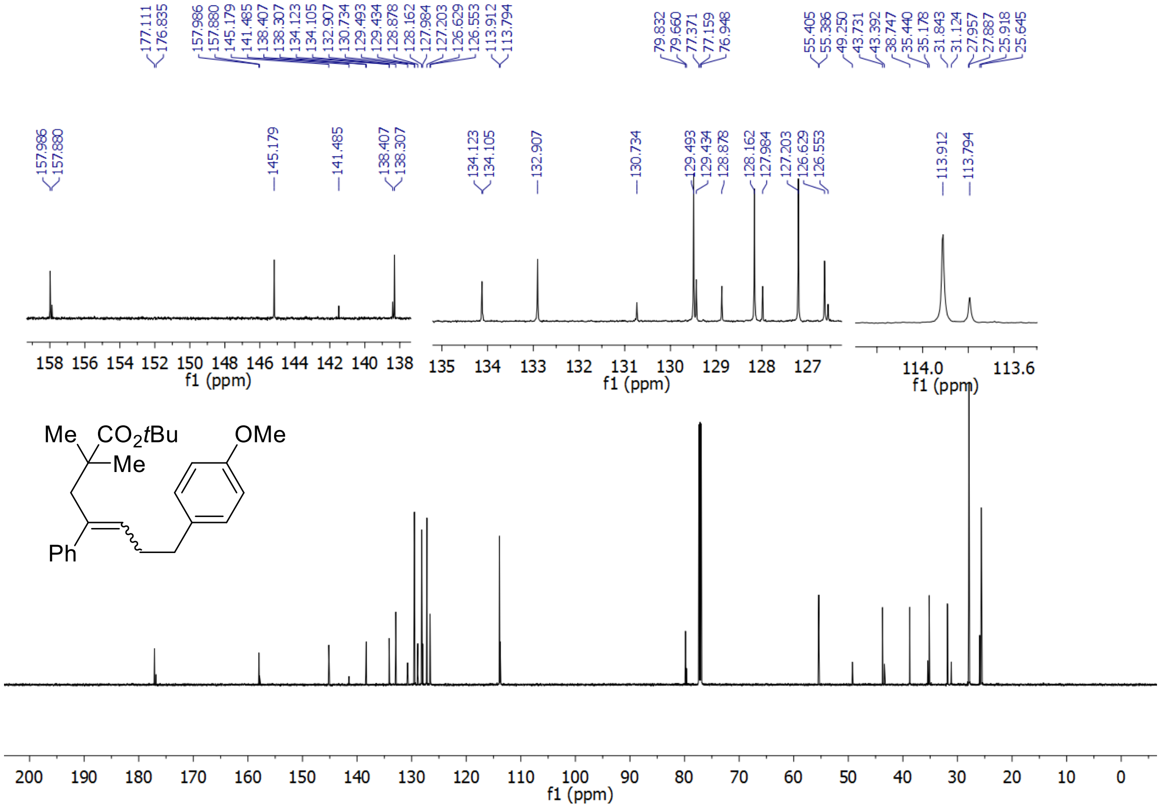

Compound 7c. Top: ^{1}H NMR ($CDCl_3$, 600 MHz). Bottom: ^{13}C NMR ($CDCl_3$, 150 MHz)

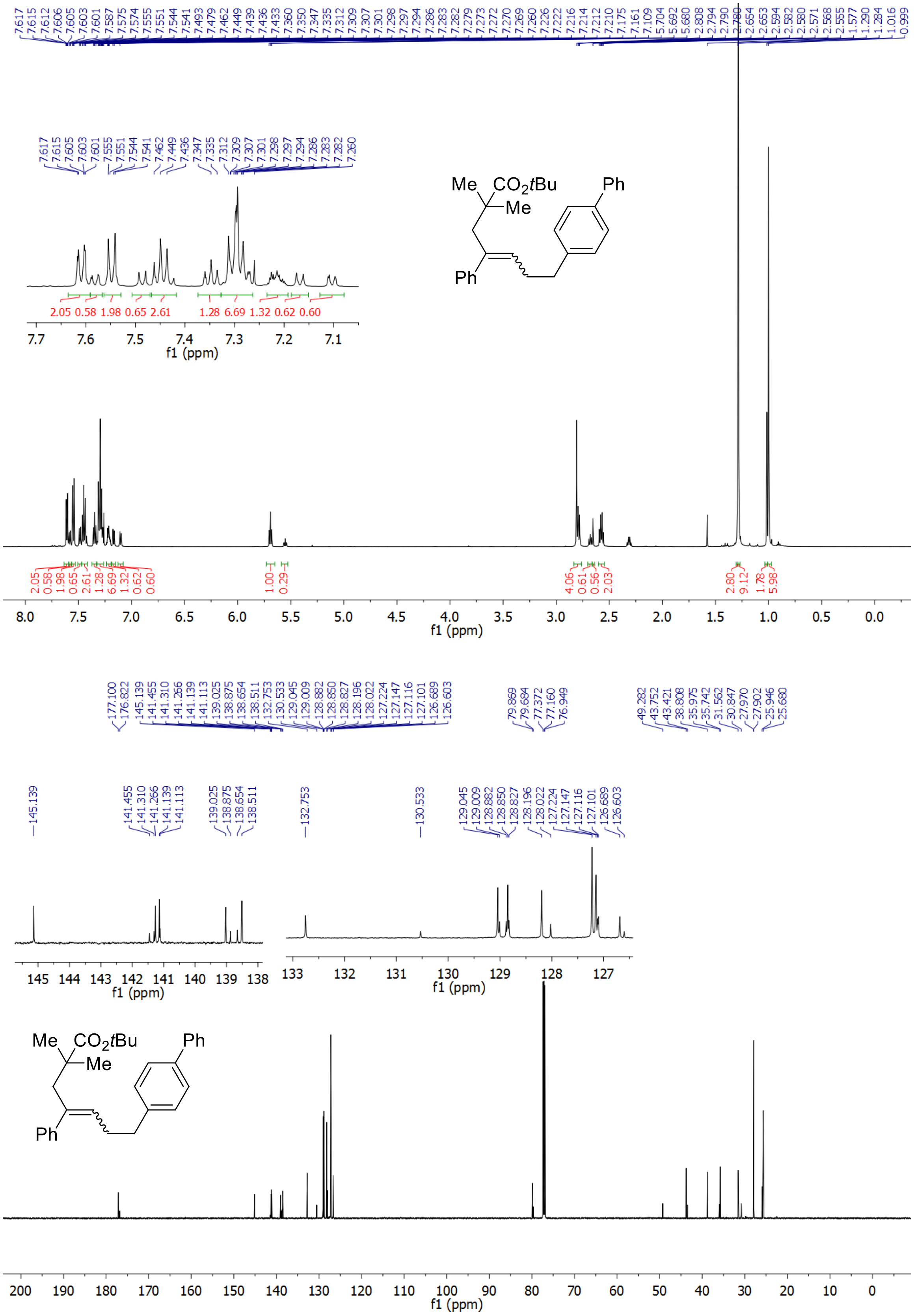

Compound 7d. Top: ^{1}H NMR ($CDCl_3$, 600 MHz). Bottom: ^{13}C NMR ($CDCl_3$, 150 MHz)

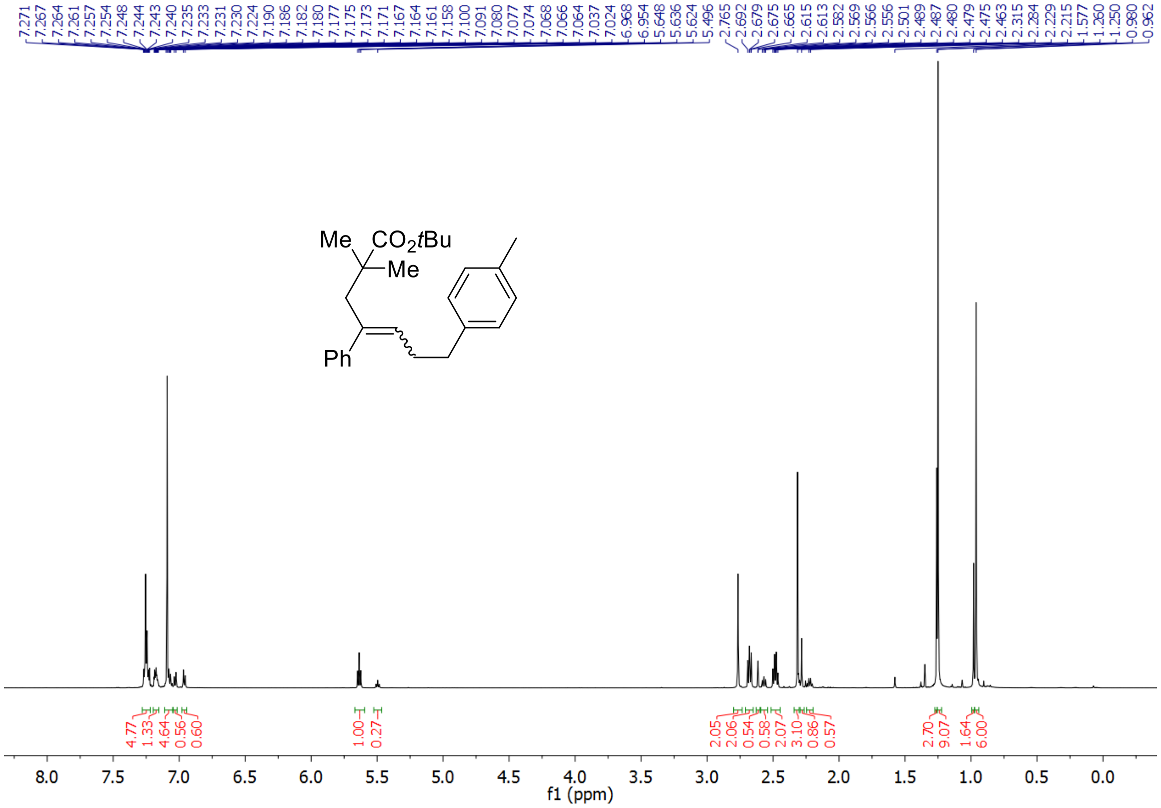

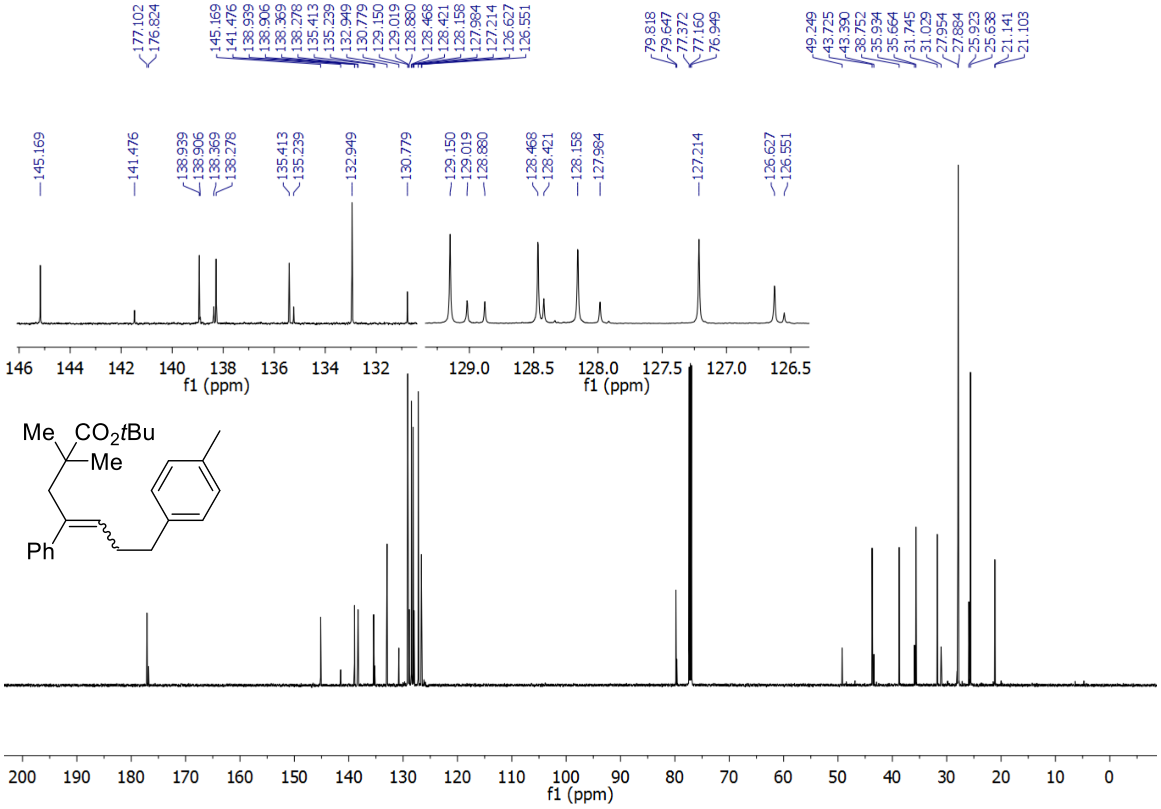

Compound 7e. Top: ^{1}H NMR ($CDCl_3$, 600 MHz). Bottom: ^{13}C NMR ($CDCl_3$, 150 MHz)

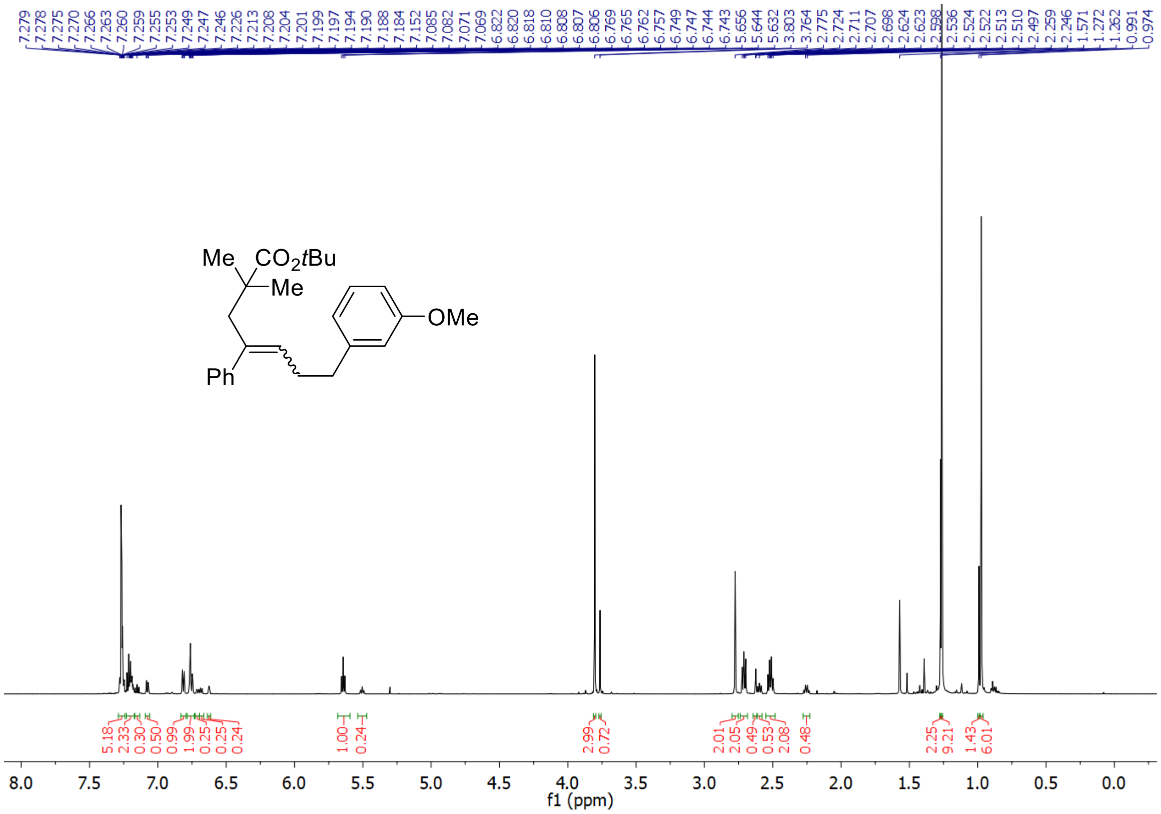

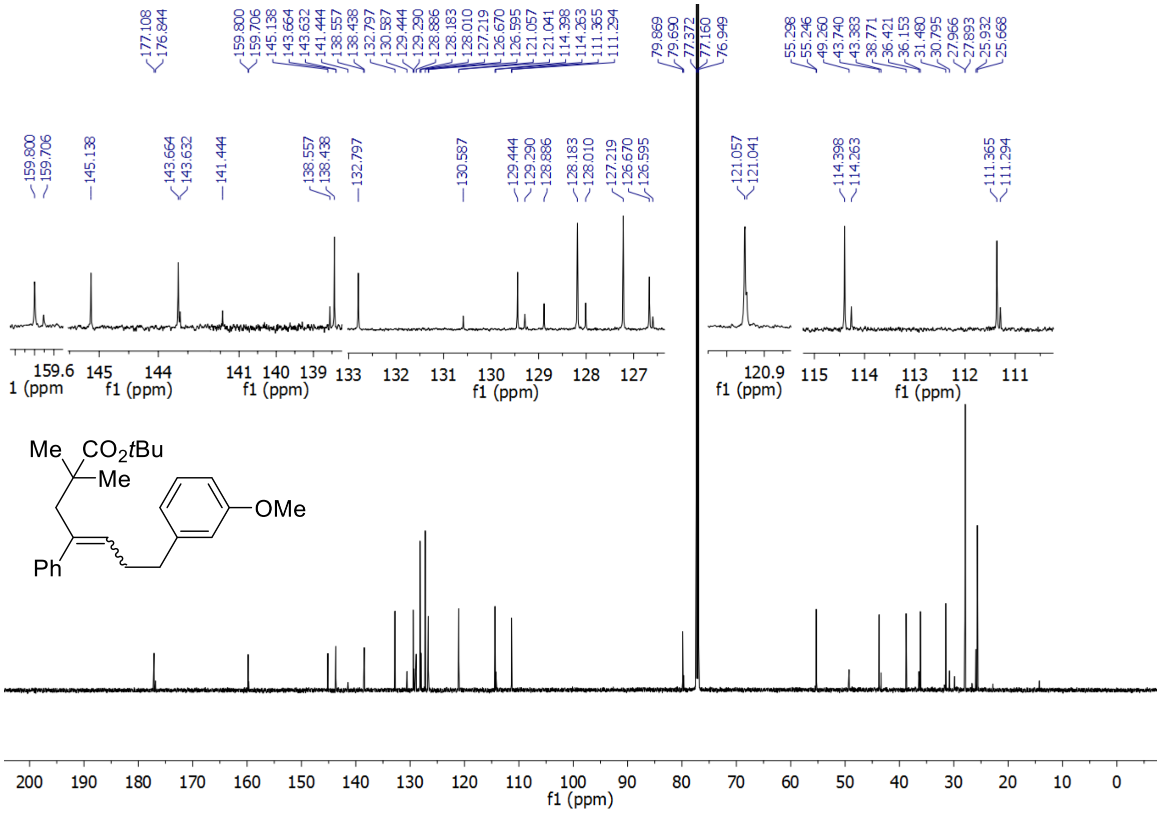

Compound 7f. Top: ^{1}H NMR ($CDCl_3$, 600 MHz). Bottom: ^{13}C NMR ($CDCl_3$, 150 MHz)

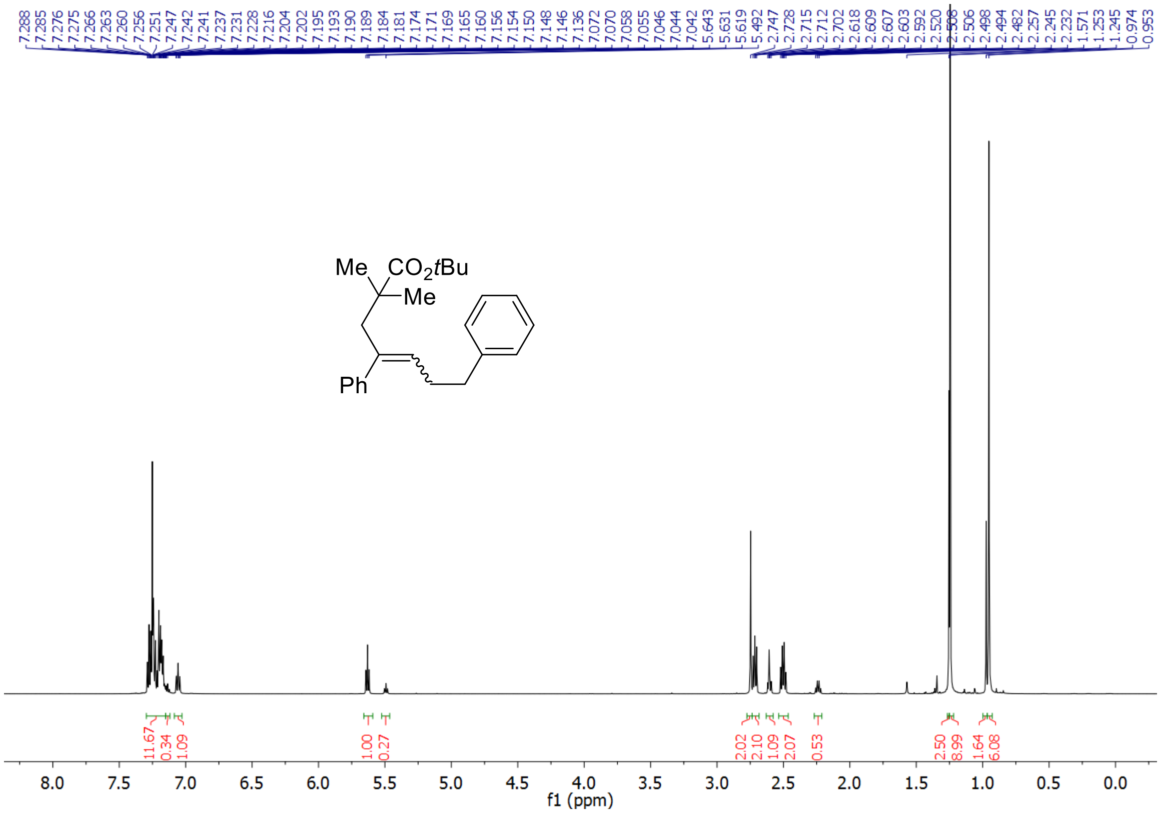

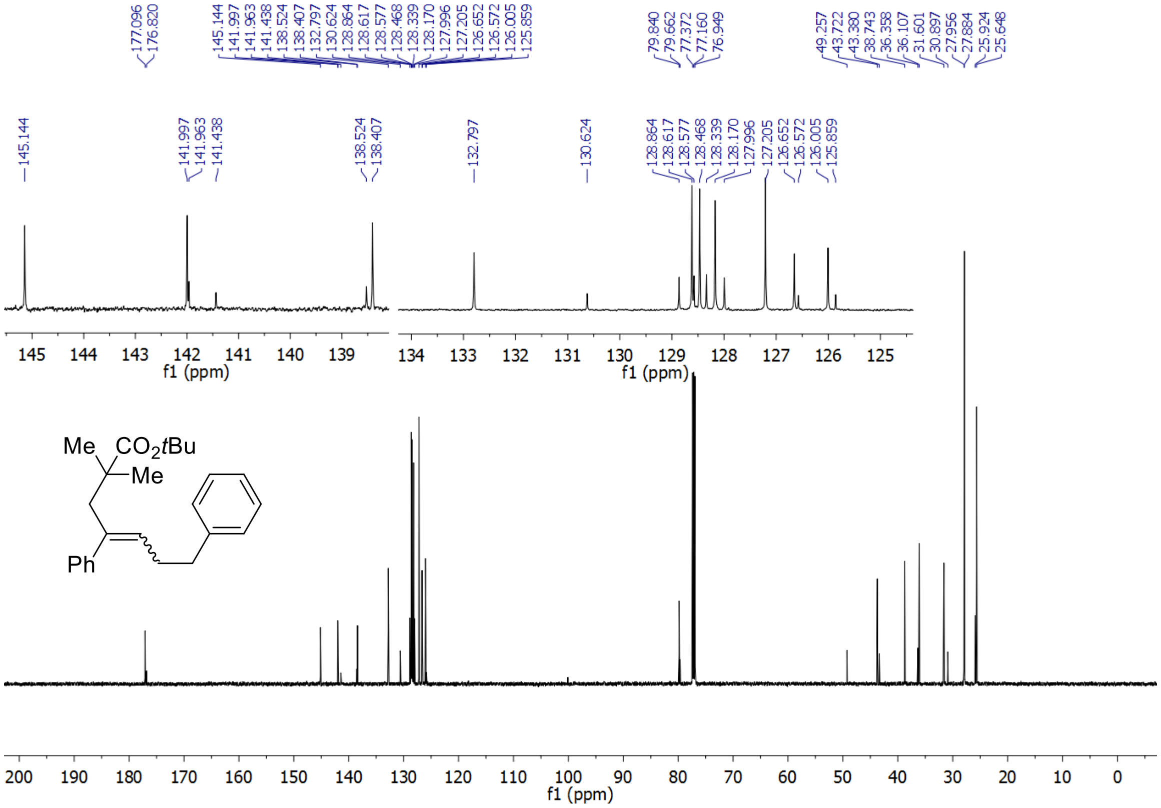

Compound 7g. Top: ^{1}H NMR ($CDCl_3$, 600 MHz). Bottom: ^{13}C NMR ($CDCl_3$, 150 MHz)

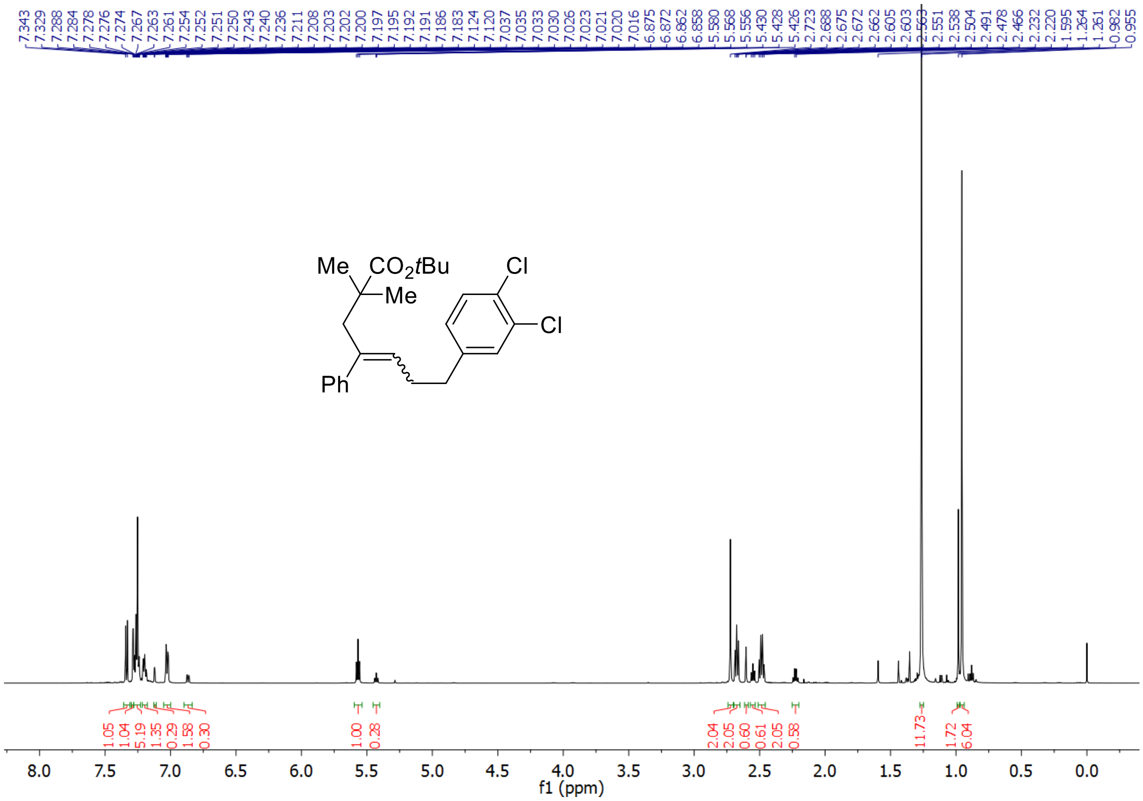

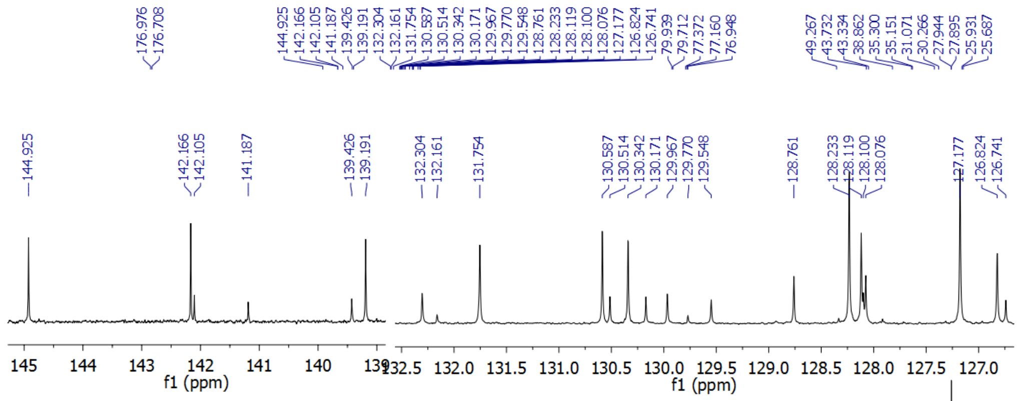

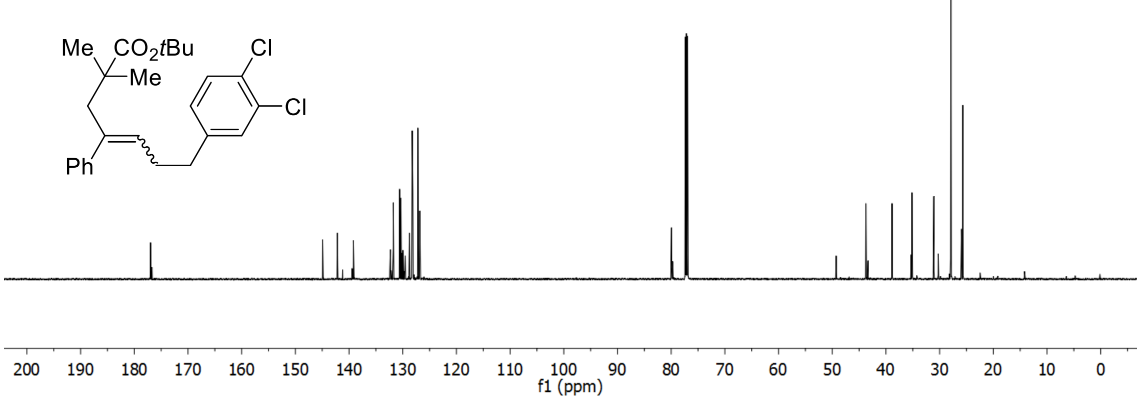

Compound 7h. Top: ^1H NMR ($CDCl_3$, 600 MHz). Bottom: ^{13}C NMR ($CDCl_3$, 150 MHz)

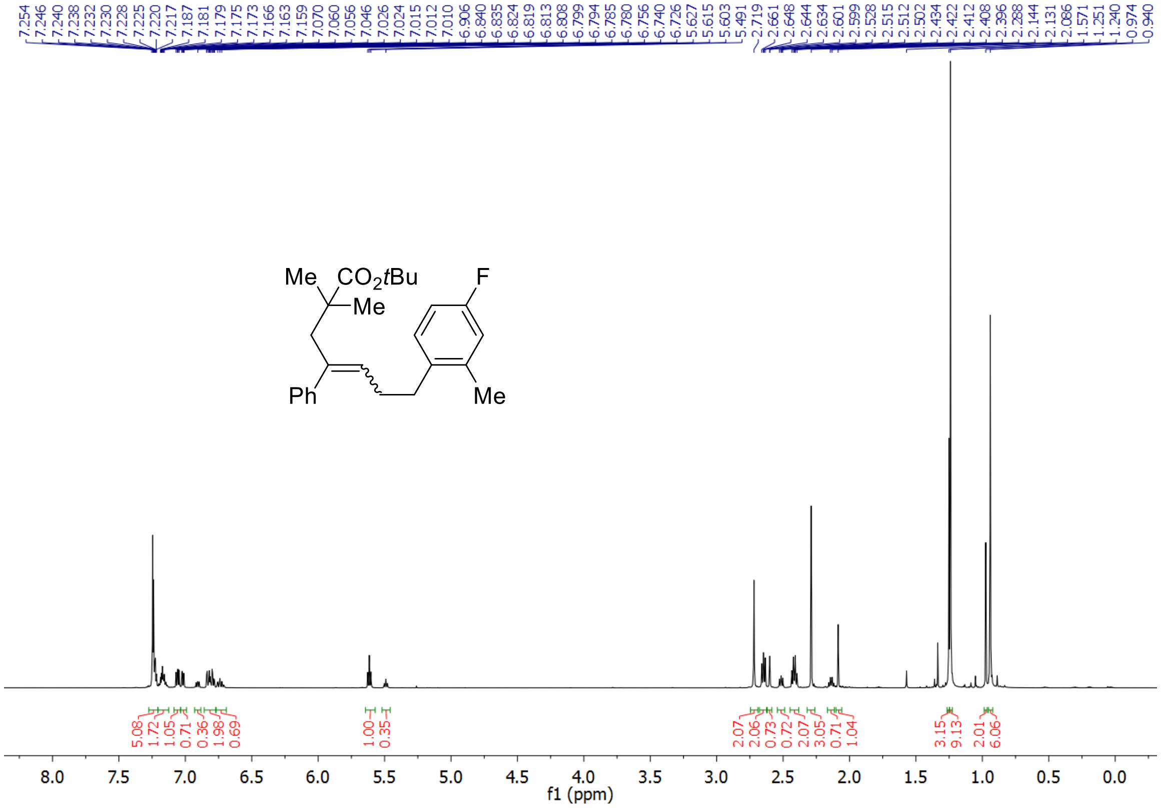

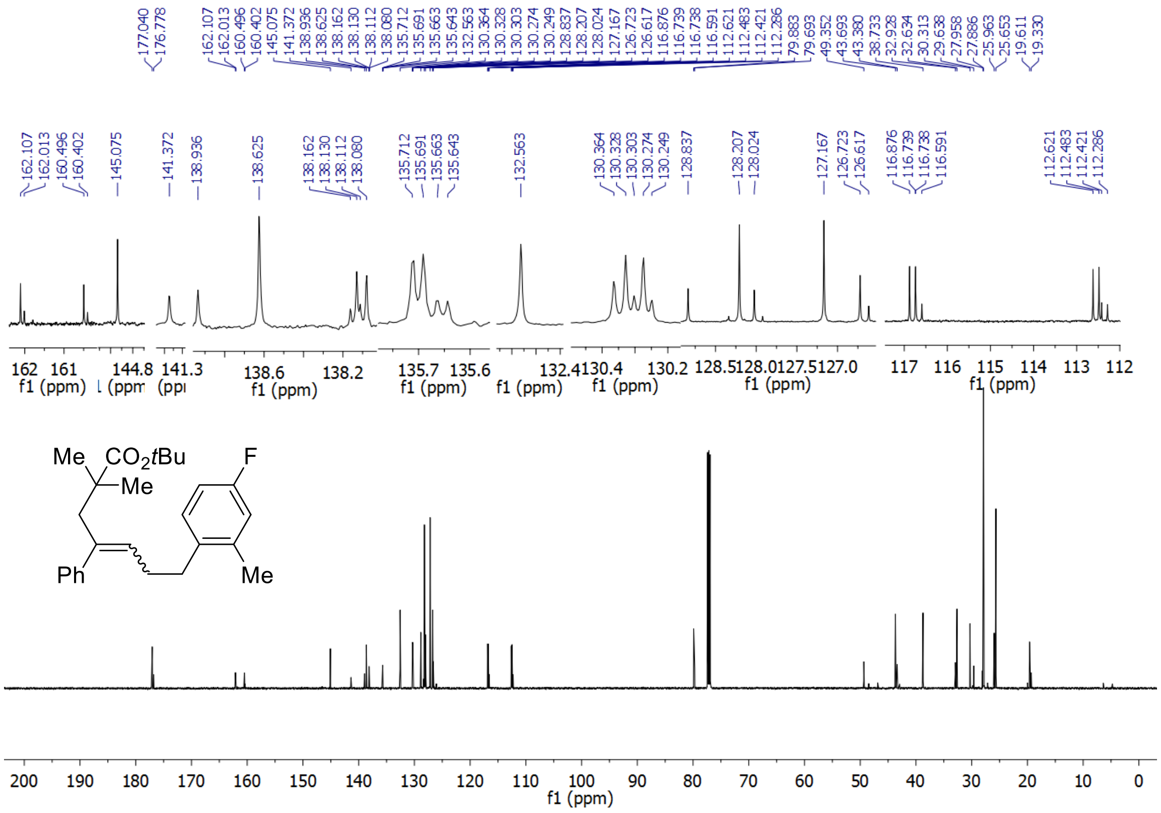

Compound 7h. ^{19}**F NMR (CDCl$_3$, 565 MHz).**

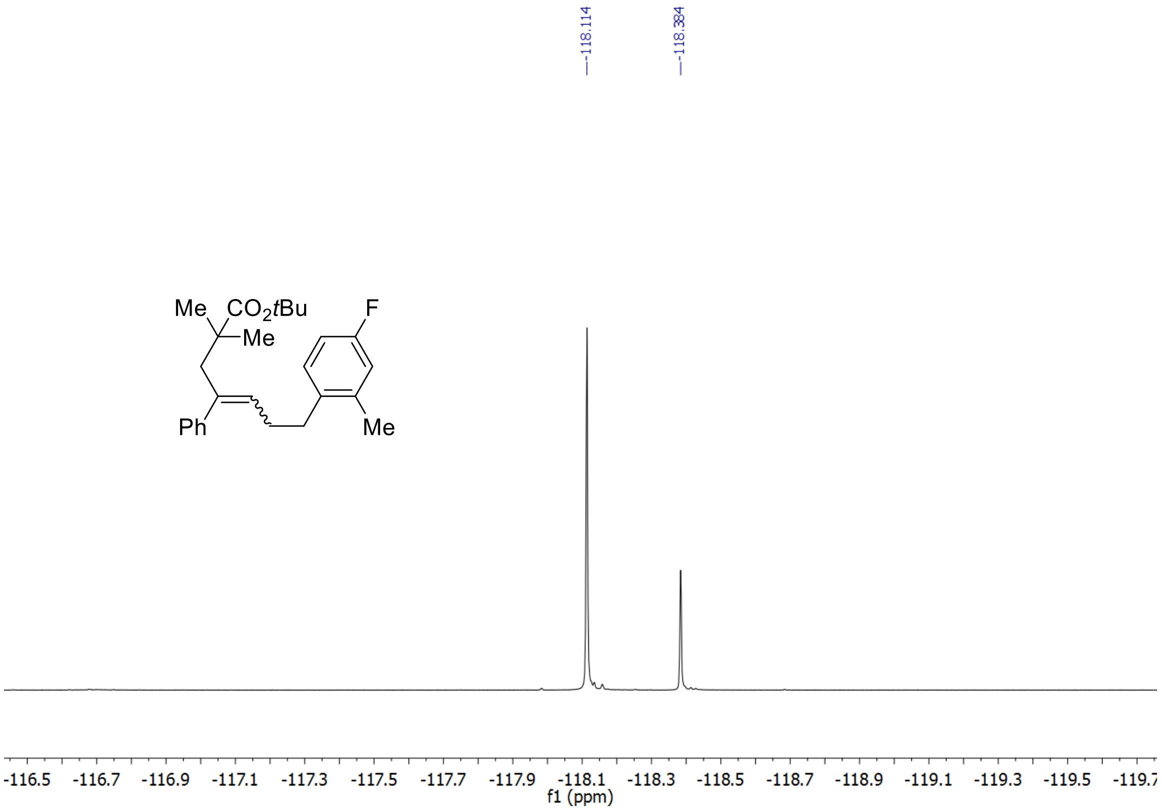

Compound 7i. Top: ^{1}H NMR ($CDCl_3$, 600 MHz). Bottom: ^{13}C NMR ($CDCl_3$, 150 MHz)

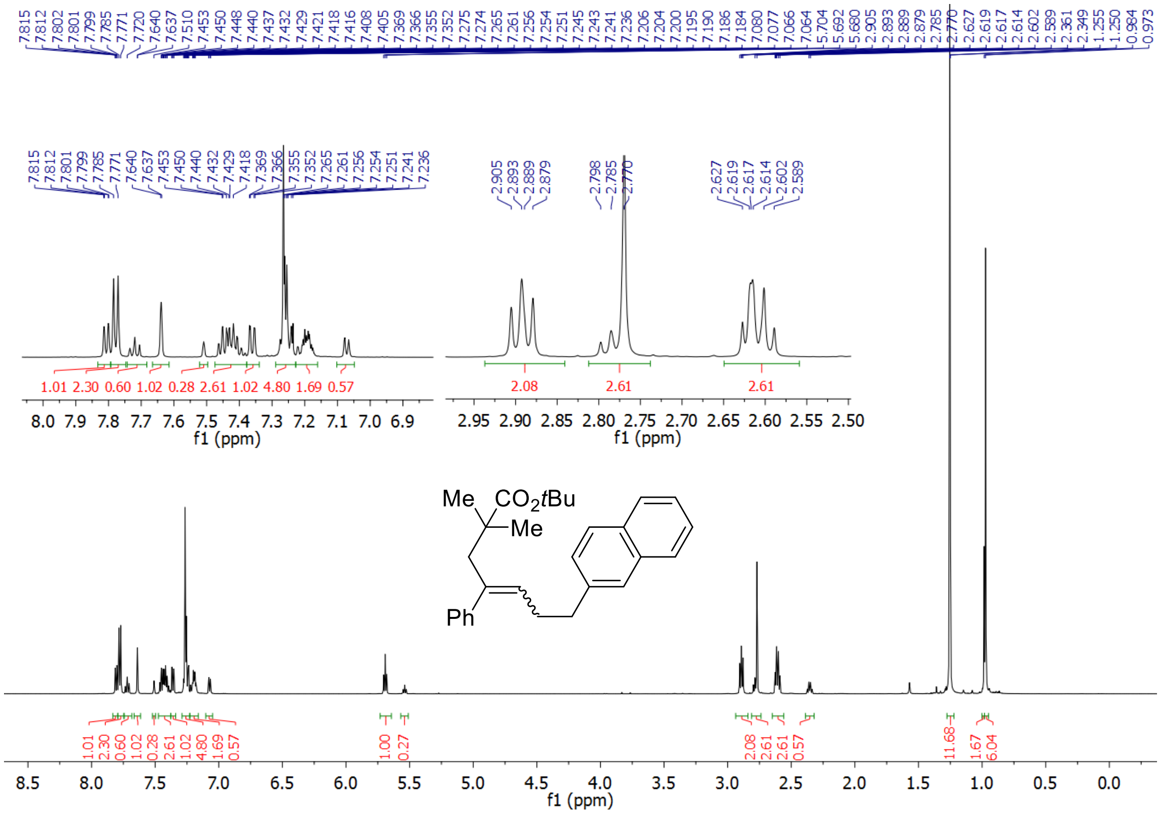

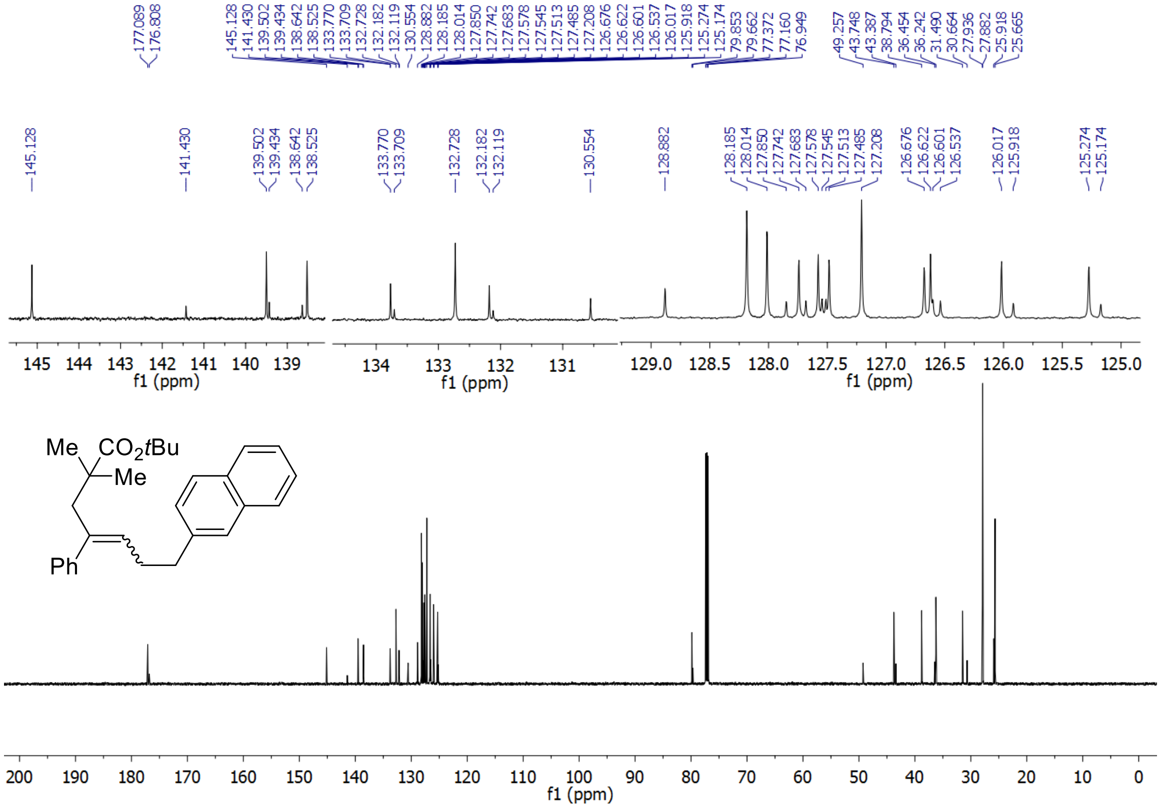

Compound 7j. Top: ^{1}H NMR ($CDCl_3$, 600 MHz). Bottom: ^{13}C NMR ($CDCl_3$, 150 MHz)

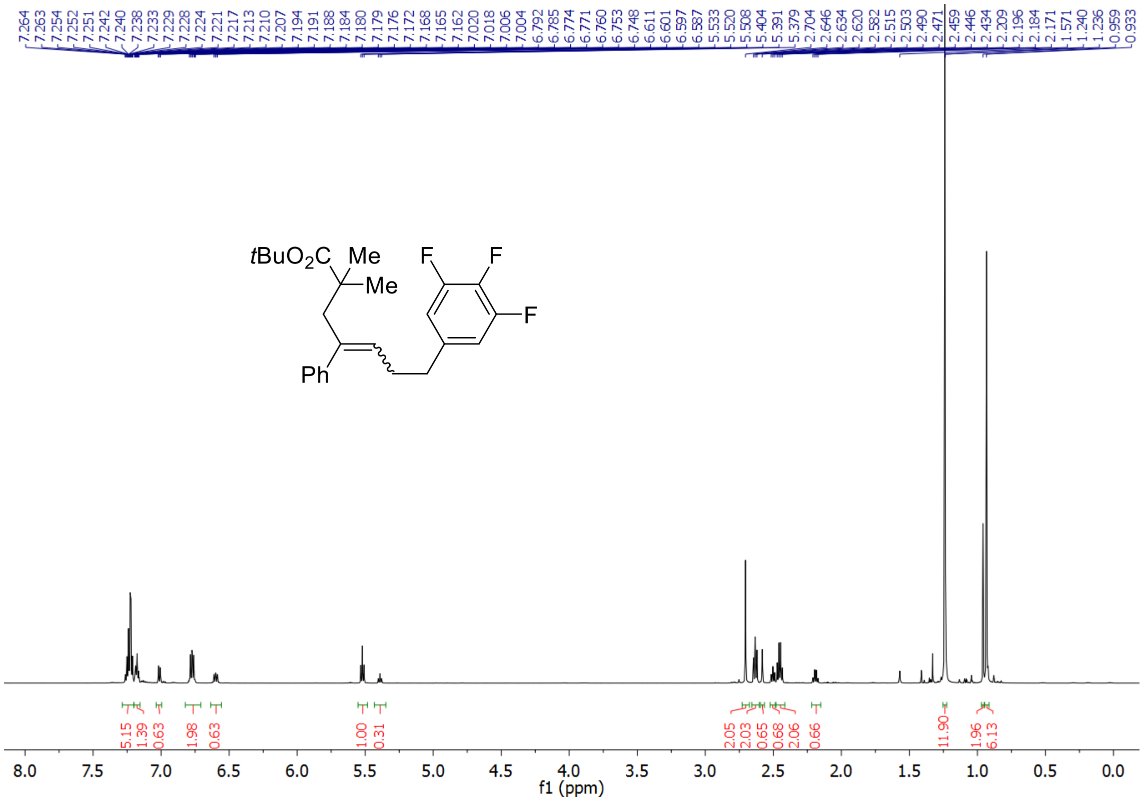

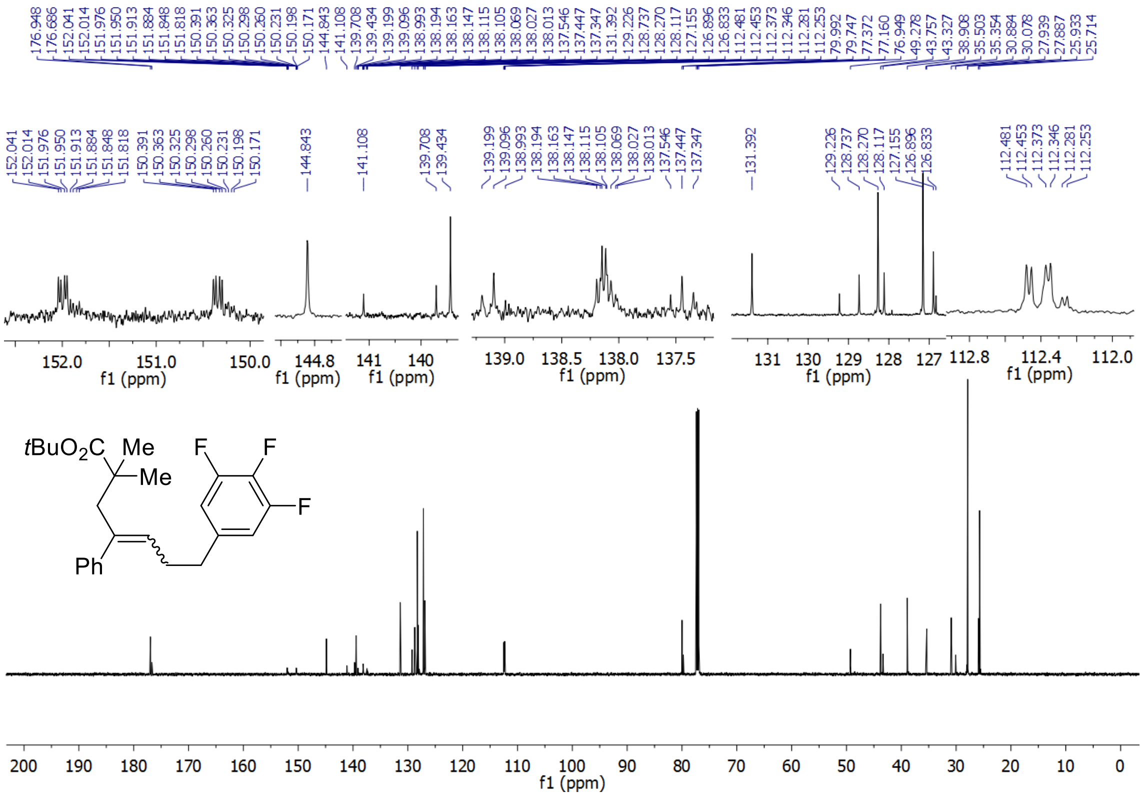

Compound 7j. [19]**F NMR ($CDCl_3$, 565 MHz).**

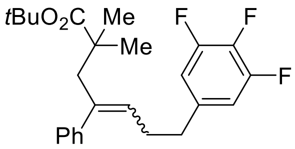

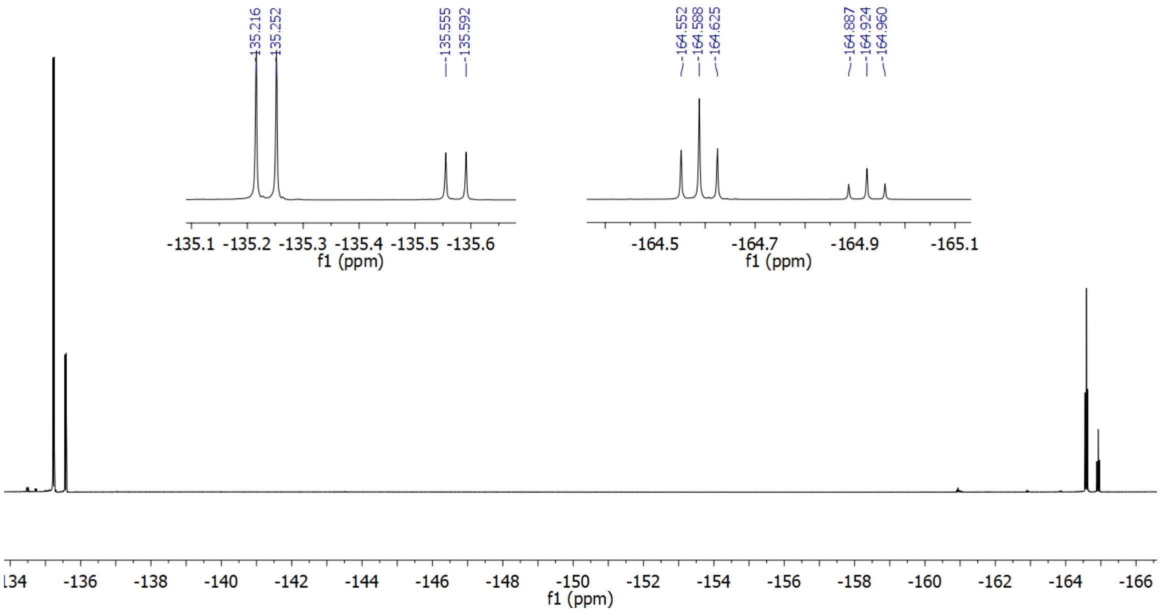

Compound 7k. Top: ^{1}H NMR ($CDCl_3$, 600 MHz). Bottom: ^{13}C NMR ($CDCl_3$, 150 MHz)

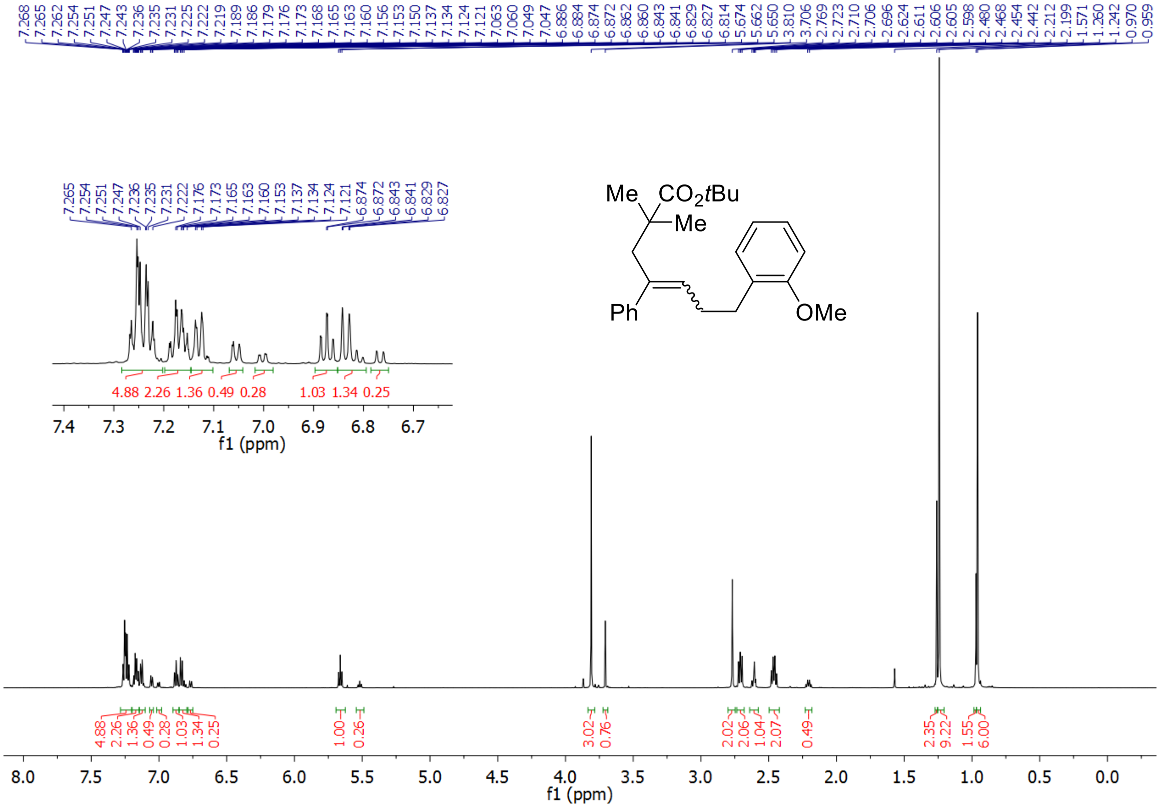

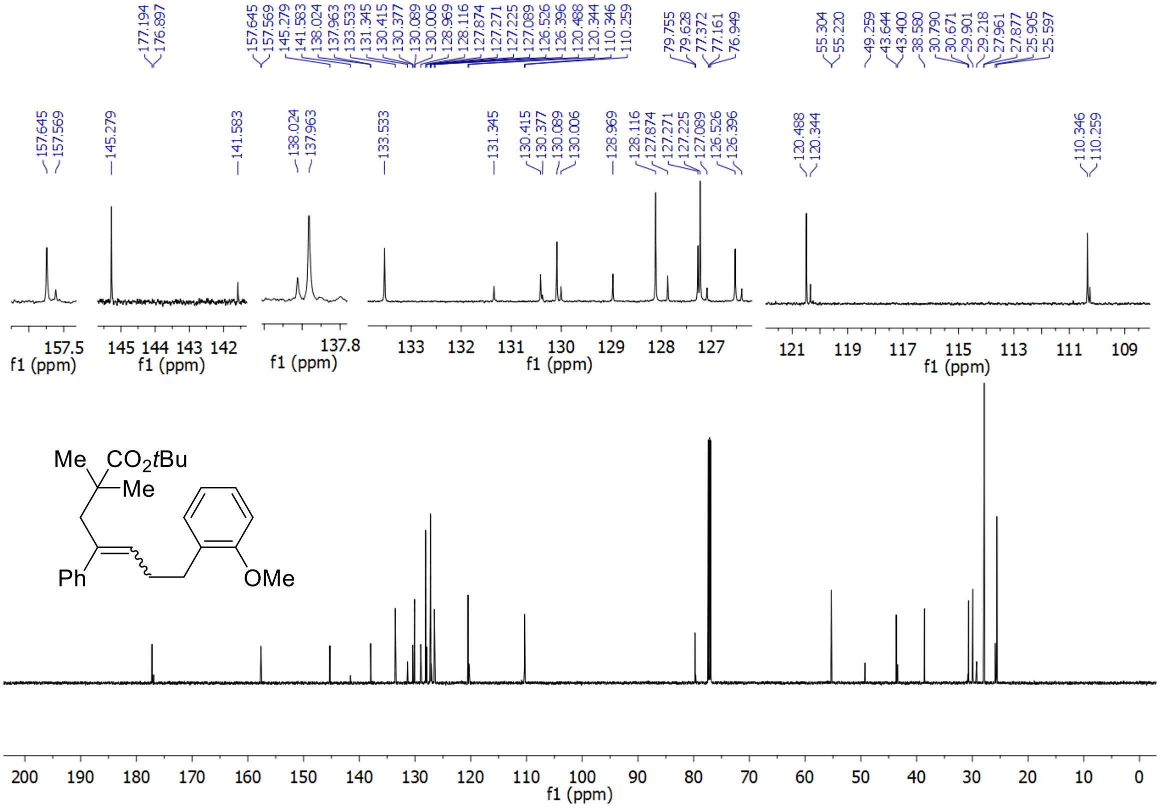

Compound 7l. Top: ^{1}H NMR ($CDCl_3$, 600 MHz). Bottom: ^{13}C NMR ($CDCl_3$, 150 MHz)

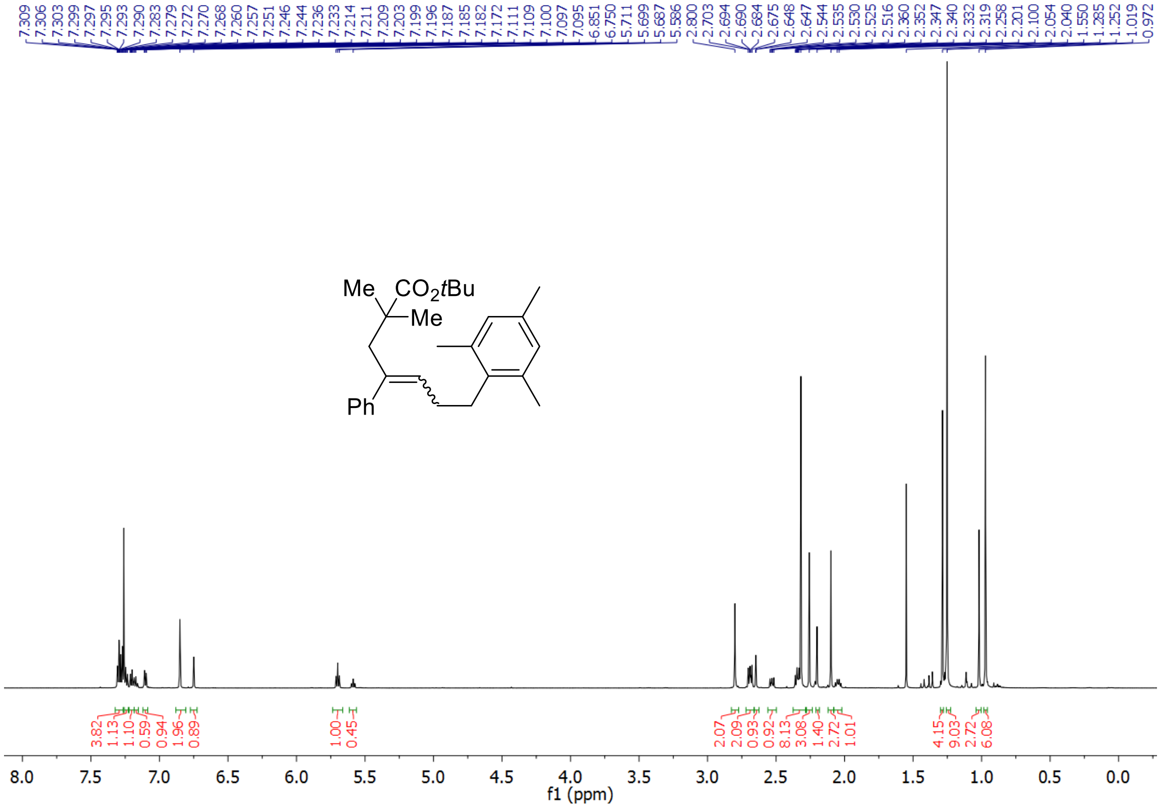

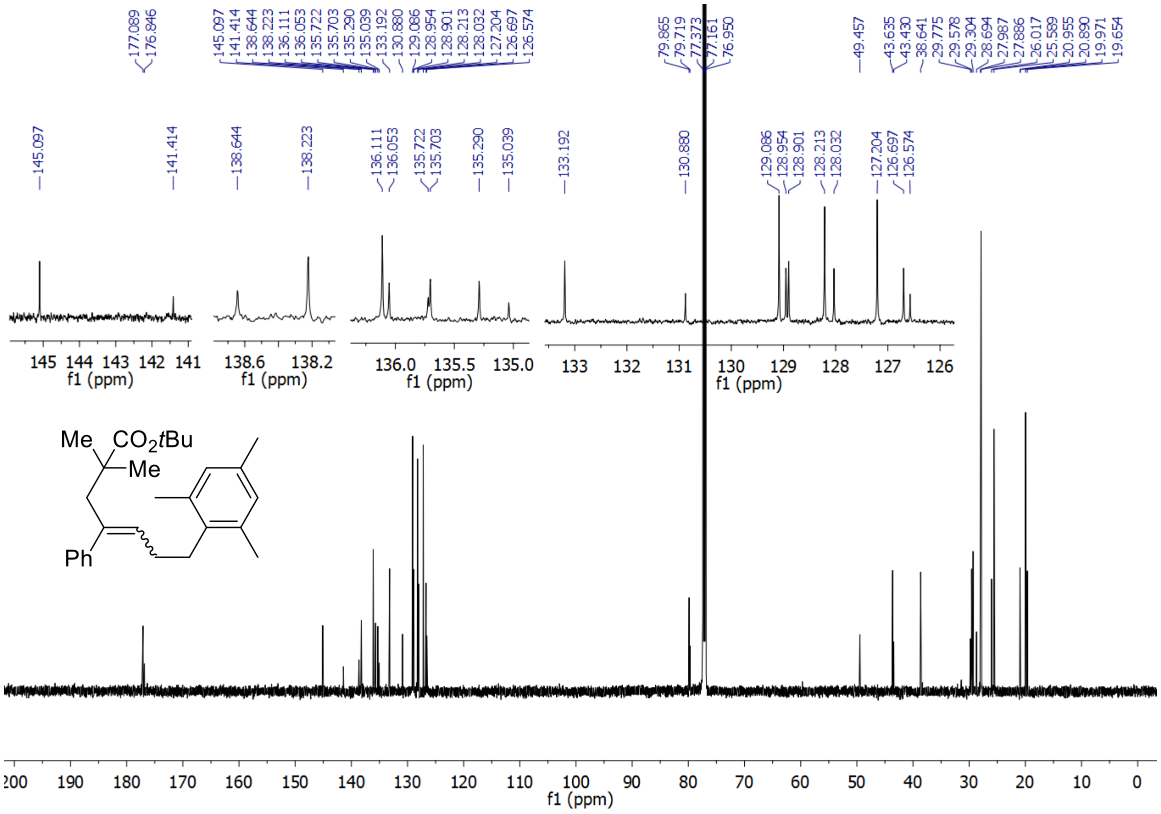

Compound 7m. Top: ^{1}H NMR ($CDCl_3$, 600 MHz). Bottom: ^{13}C NMR ($CDCl_3$, 150 MHz)

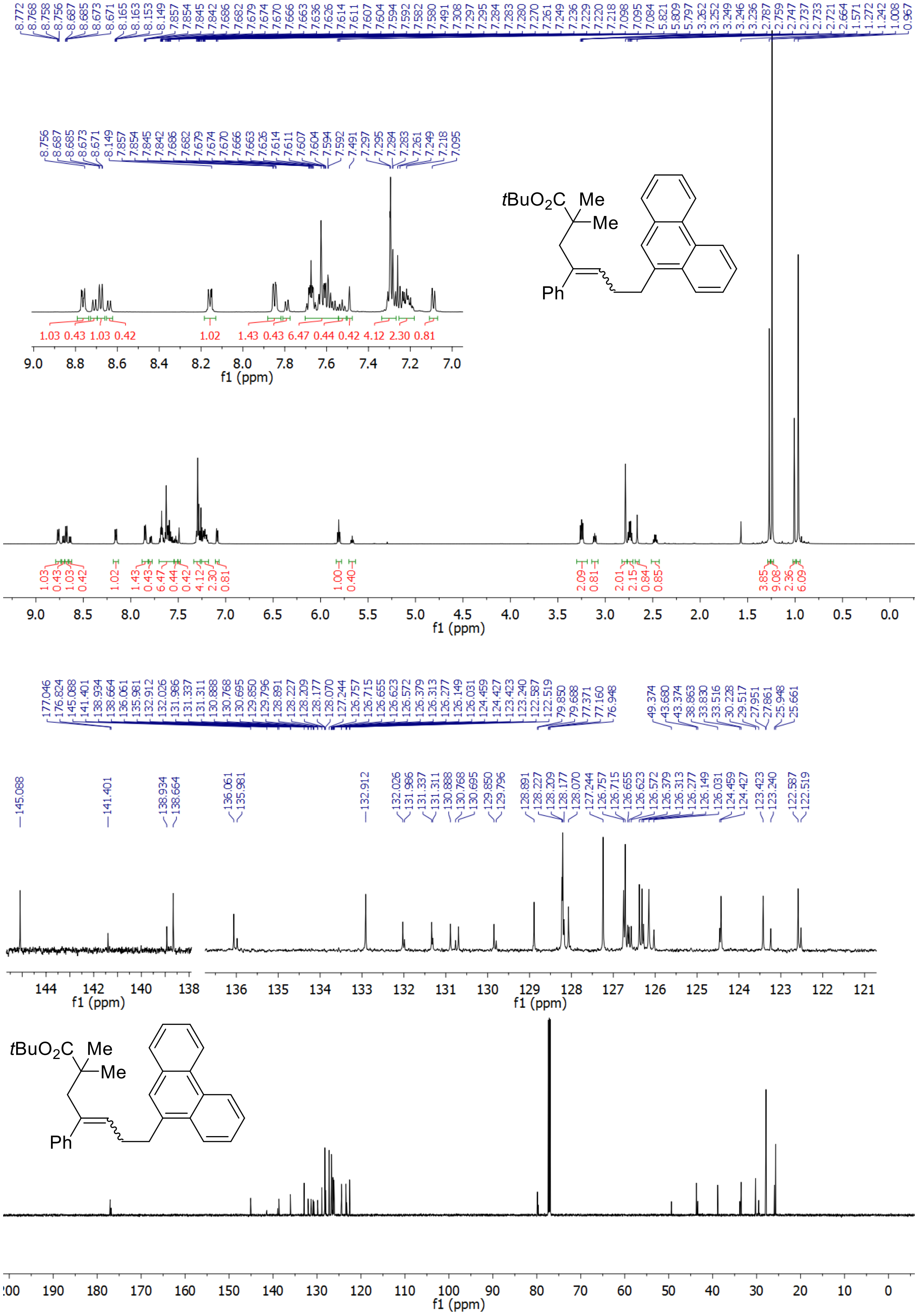

Compound 7n. Top: ^{1}H NMR ($CDCl_3$, 600 MHz). Bottom: ^{13}C NMR ($CDCl_3$, 150 MHz)

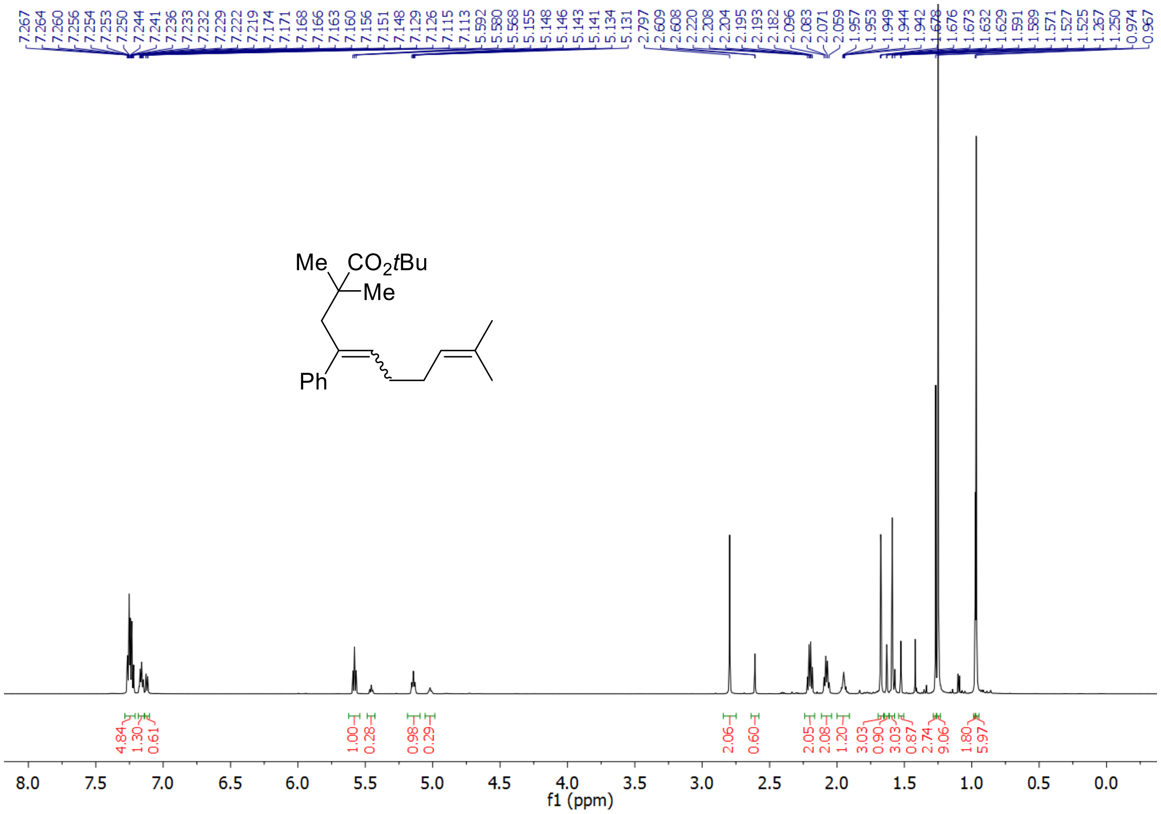

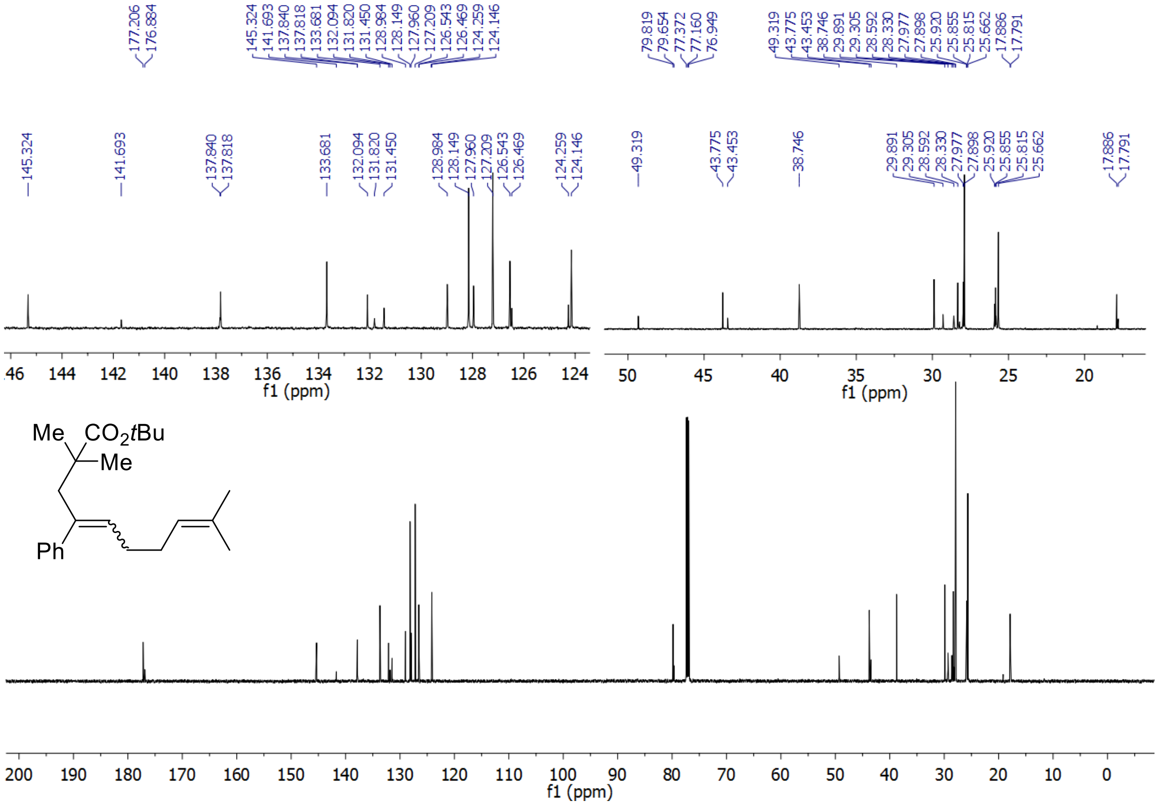

Compound 7o. Top: ^{1}H NMR ($CDCl_3$, 600 MHz). Bottom: ^{13}C NMR ($CDCl_3$, 150 MHz)

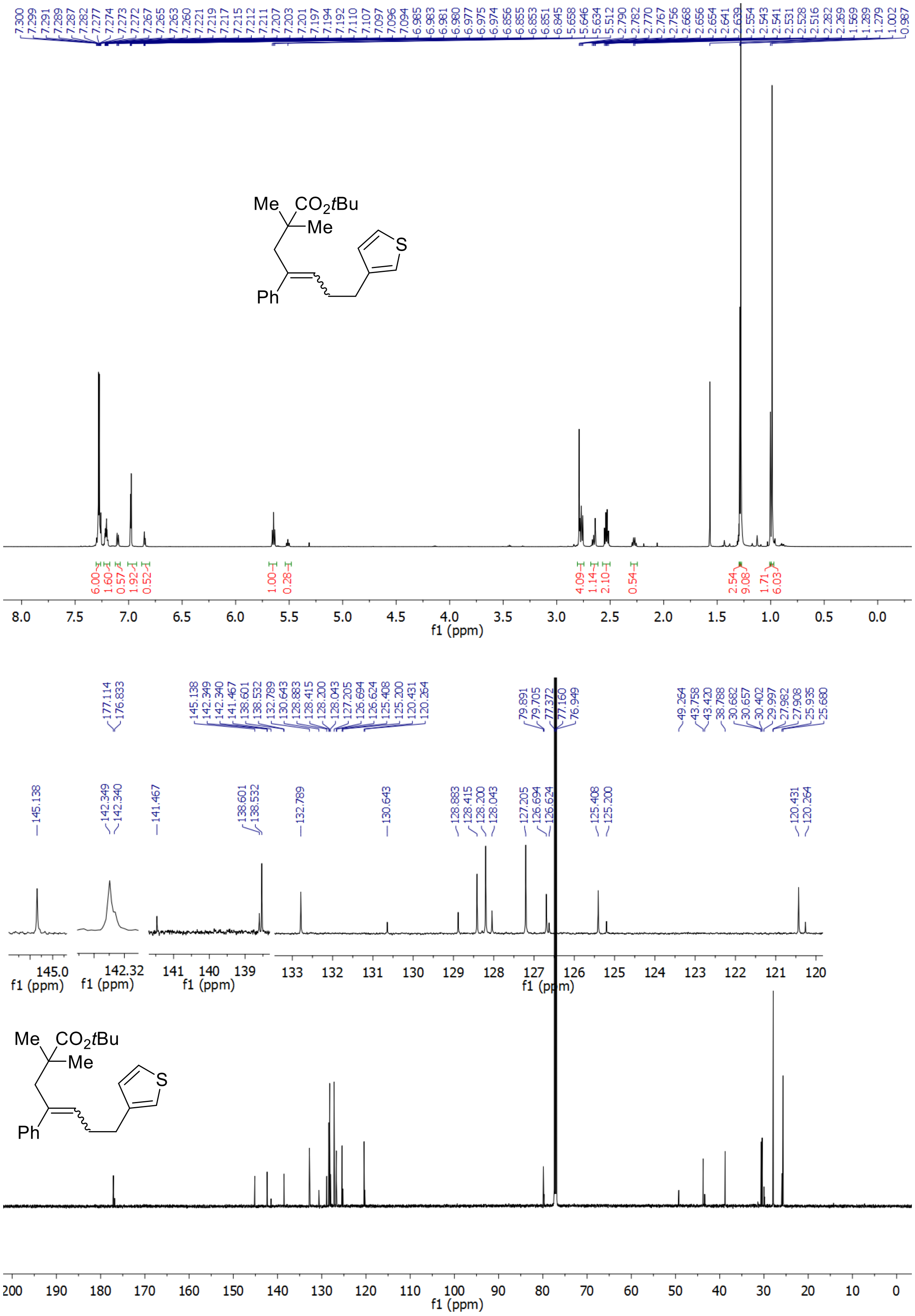

Compound 7p. Top: ^{1}H NMR ($CDCl_3$, 600 MHz). Bottom: ^{13}C NMR ($CDCl_3$, 150 MHz)

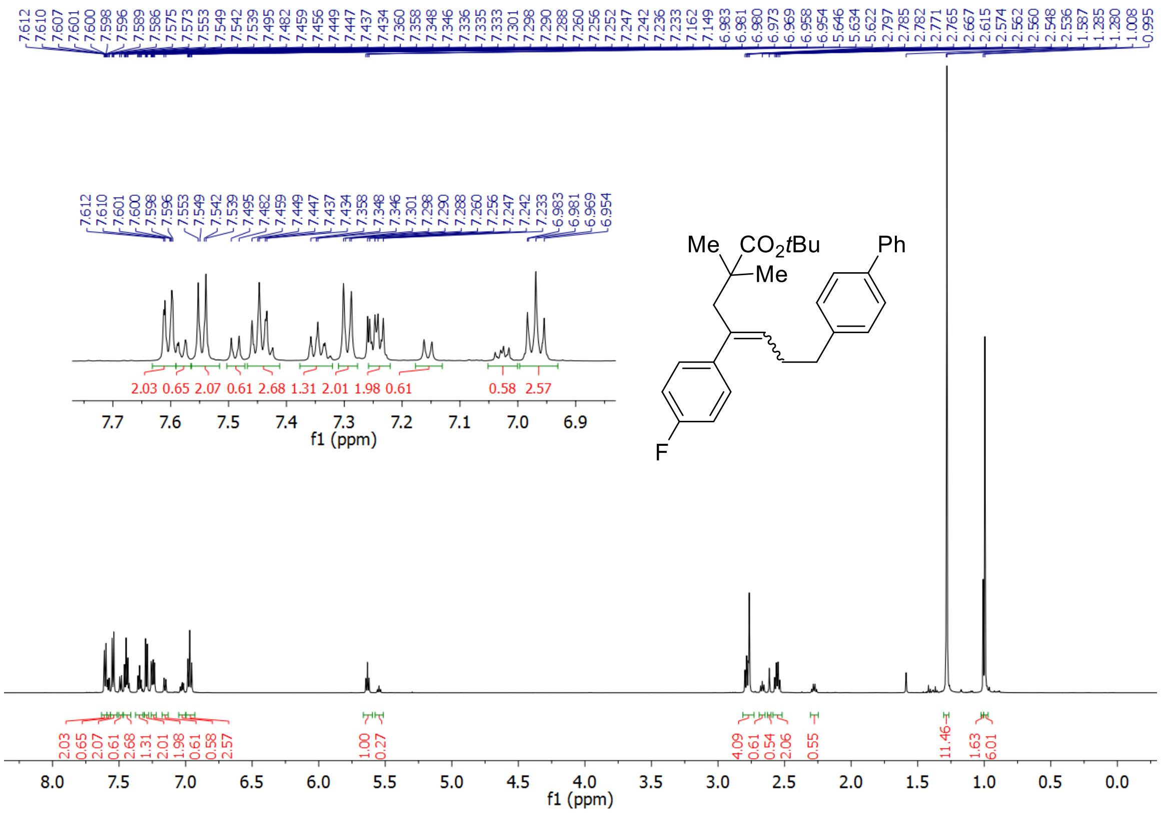

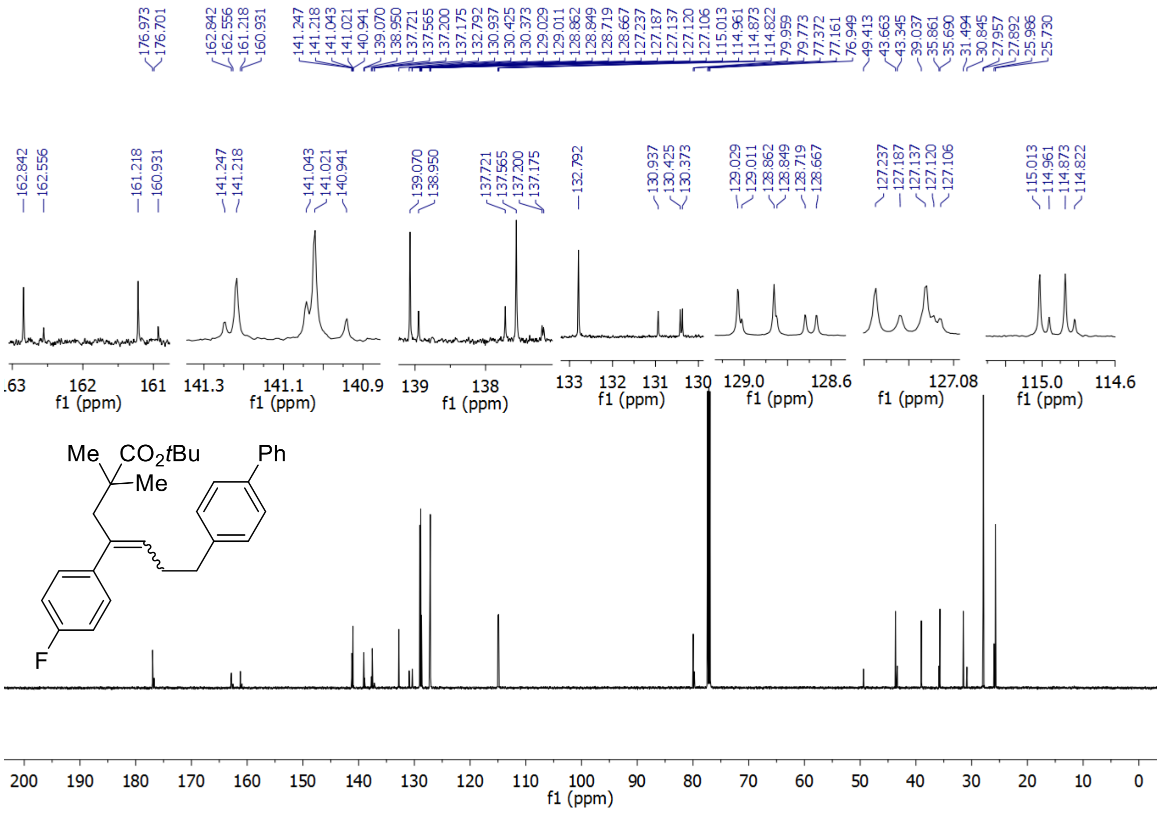

Compound 7p. ^{19}F NMR ($CDCl_3$, 565 MHz).

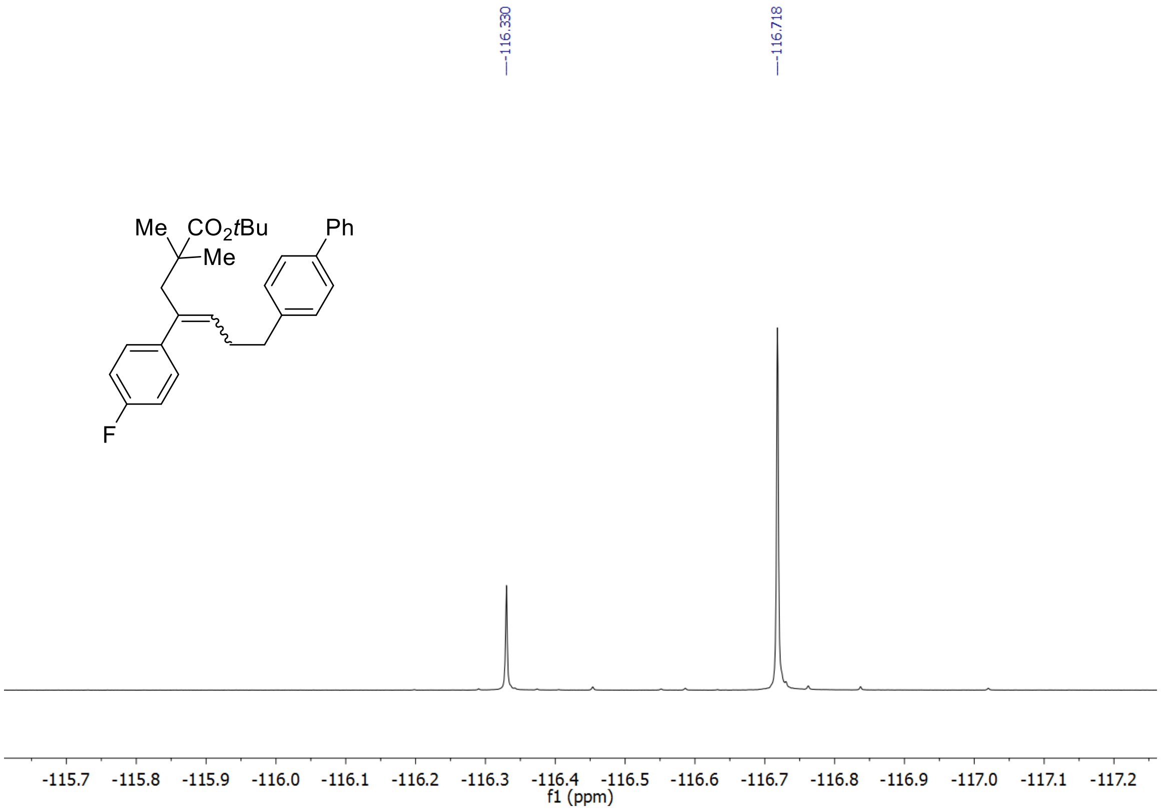

Compound 7q. Top: ^{1}H NMR ($CDCl_3$, 600 MHz). Bottom: ^{13}C NMR ($CDCl_3$, 150 MHz)

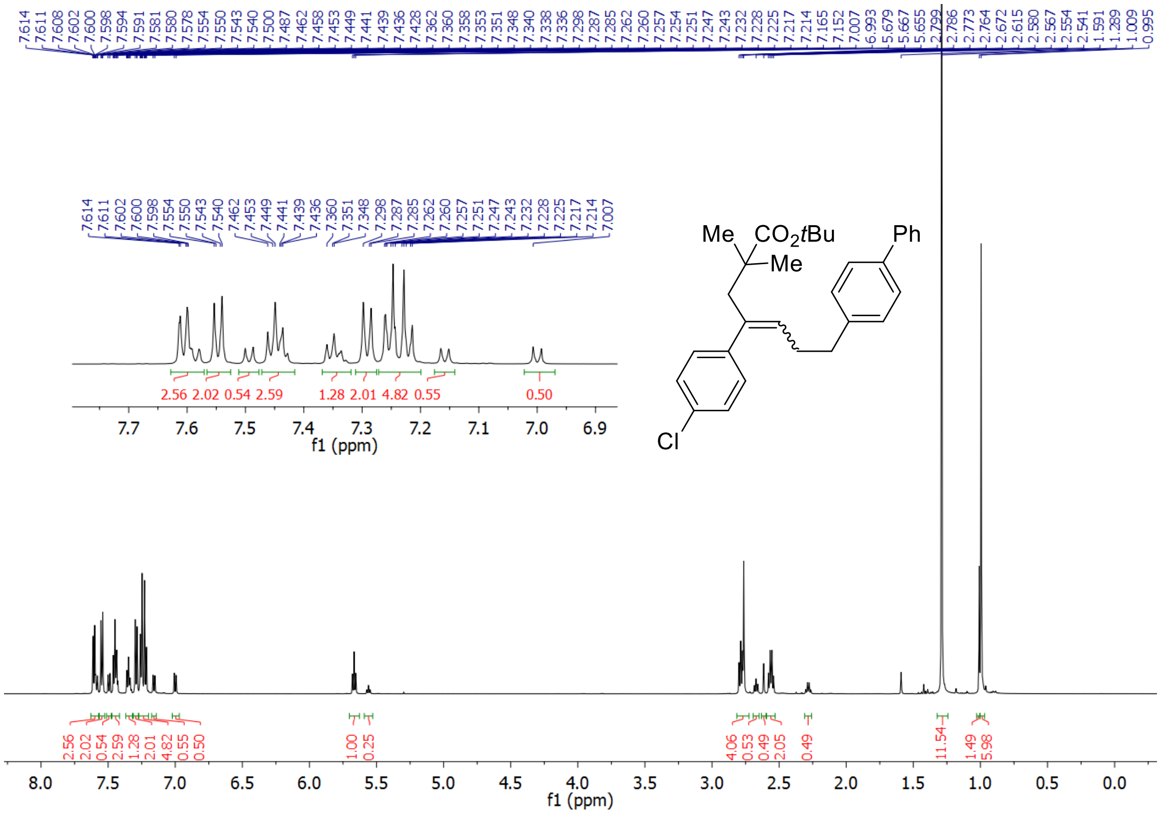

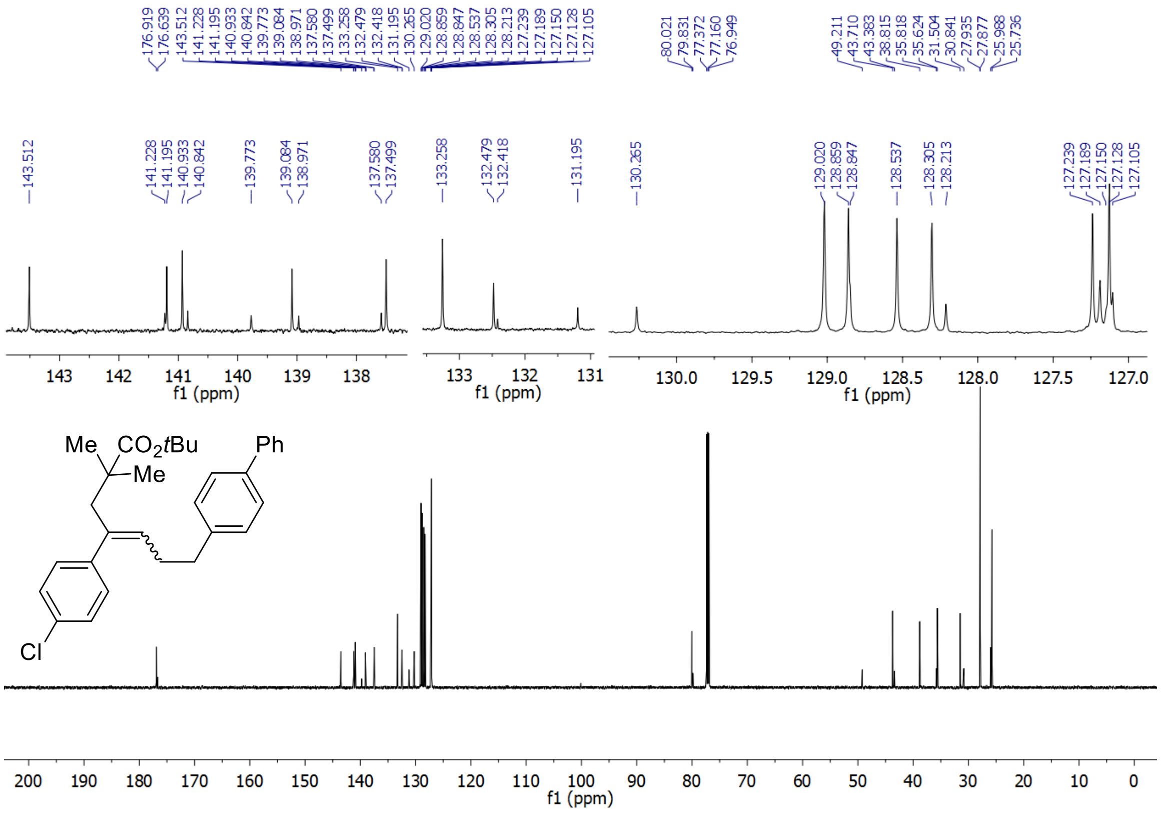

Compound 7r. Top: ^{1}H NMR ($CDCl_3$, 600 MHz). Bottom: ^{13}C NMR ($CDCl_3$, 150 MHz)

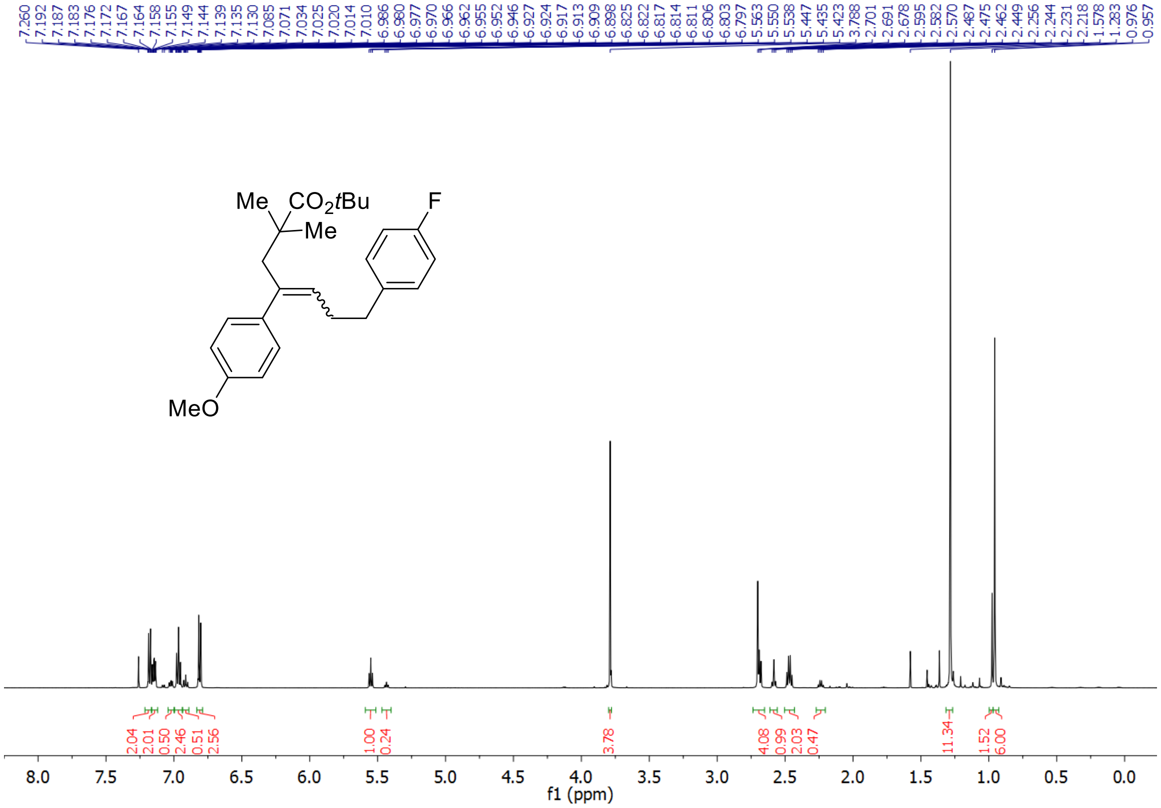

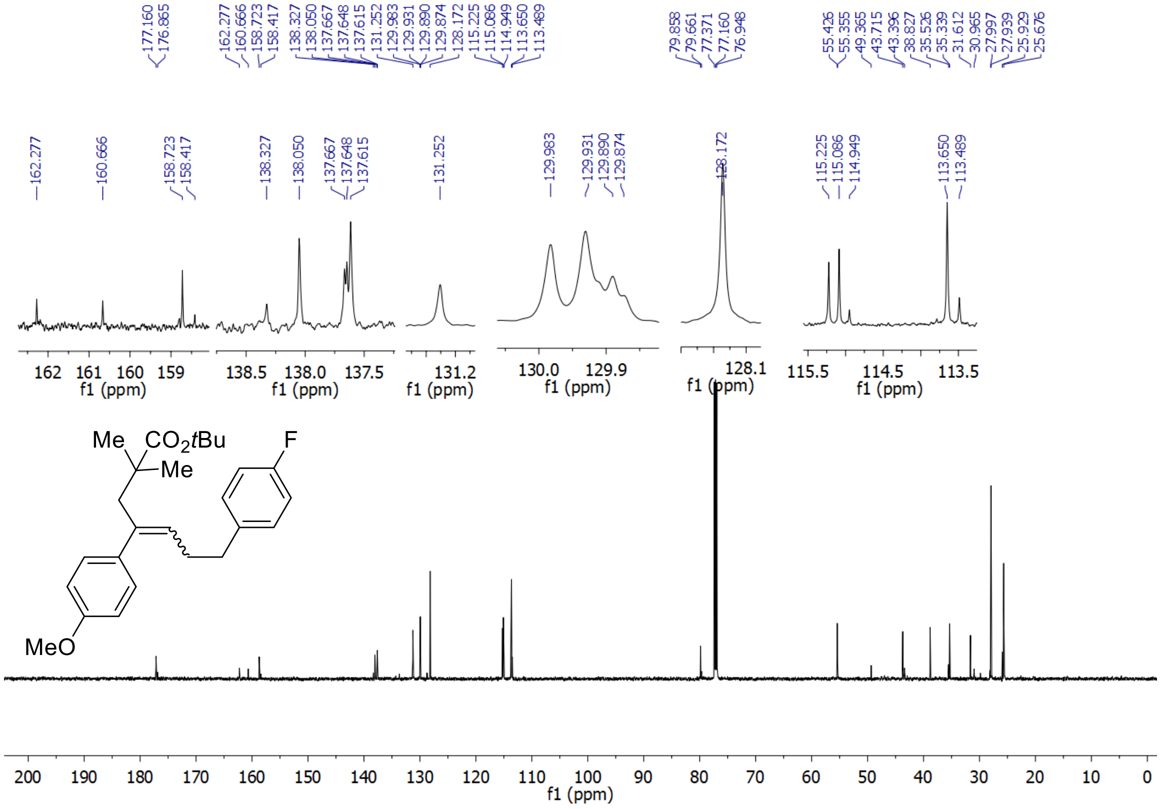

Compound 7r. ^{19}F NMR ($CDCl_3$, 565 MHz).

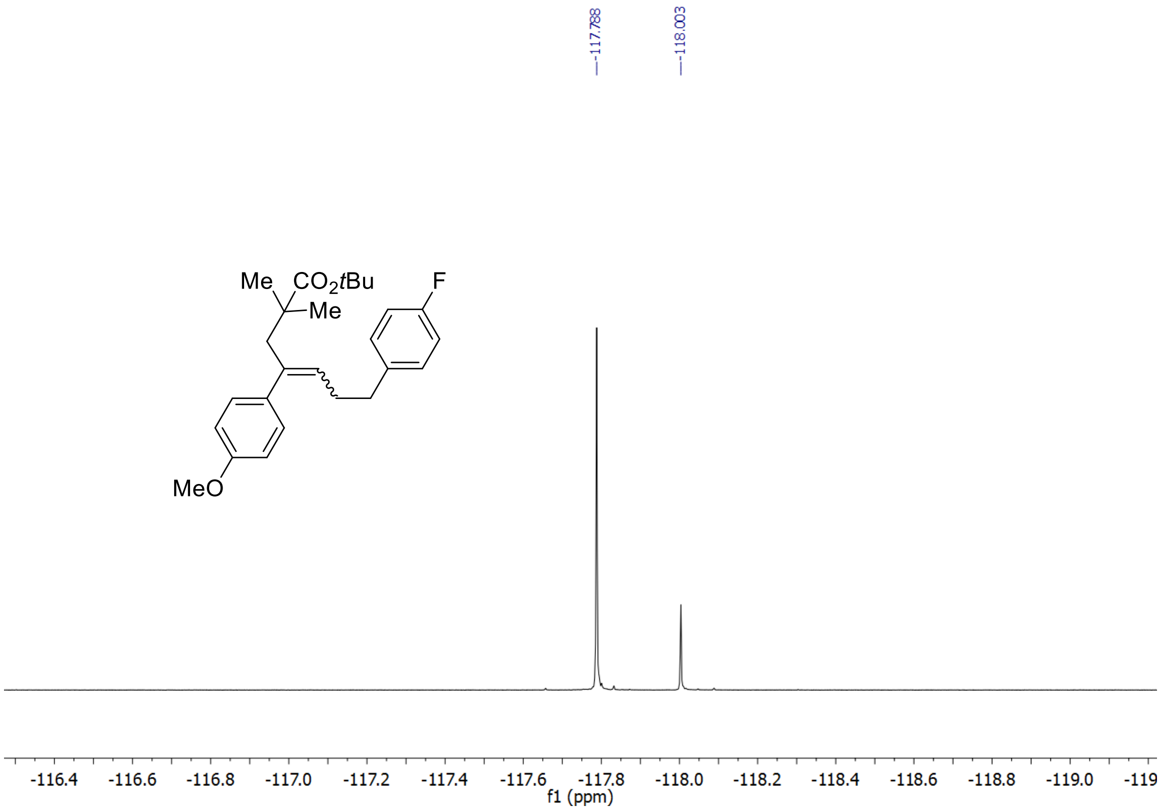

Compound (*E*)-7s. Top: ^{1}H NMR ($CDCl_3$, 600 MHz). Bottom: ^{13}C NMR ($CDCl_3$, 150 MHz)

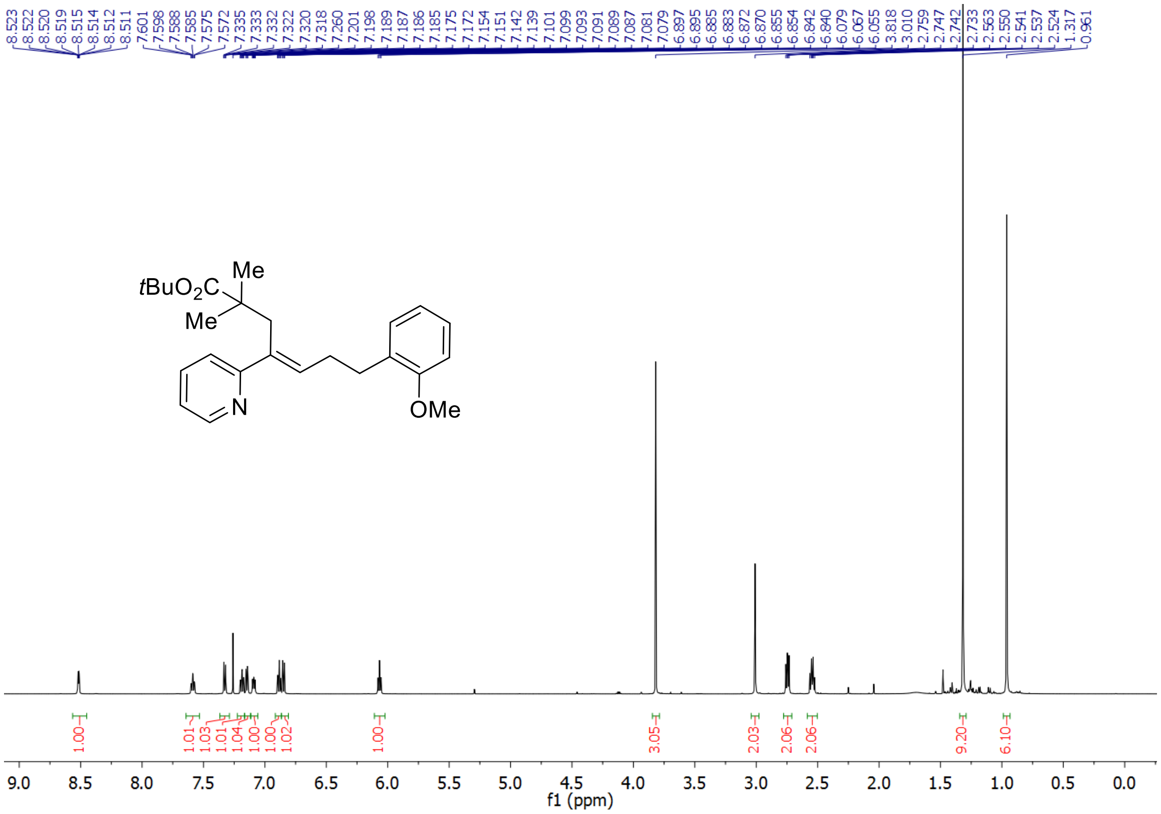

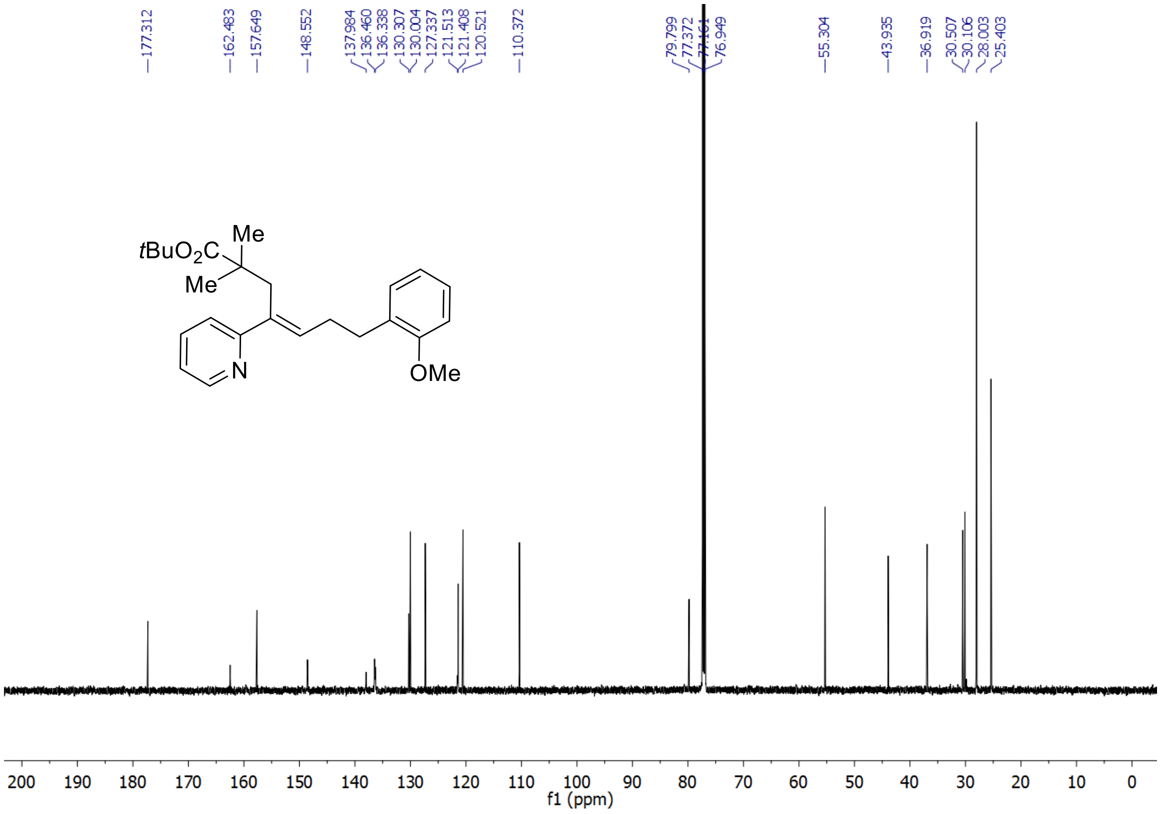

Compound 7t. Top: ^{1}H NMR ($CDCl_3$, 600 MHz). Bottom: ^{13}C NMR ($CDCl_3$, 150 MHz)

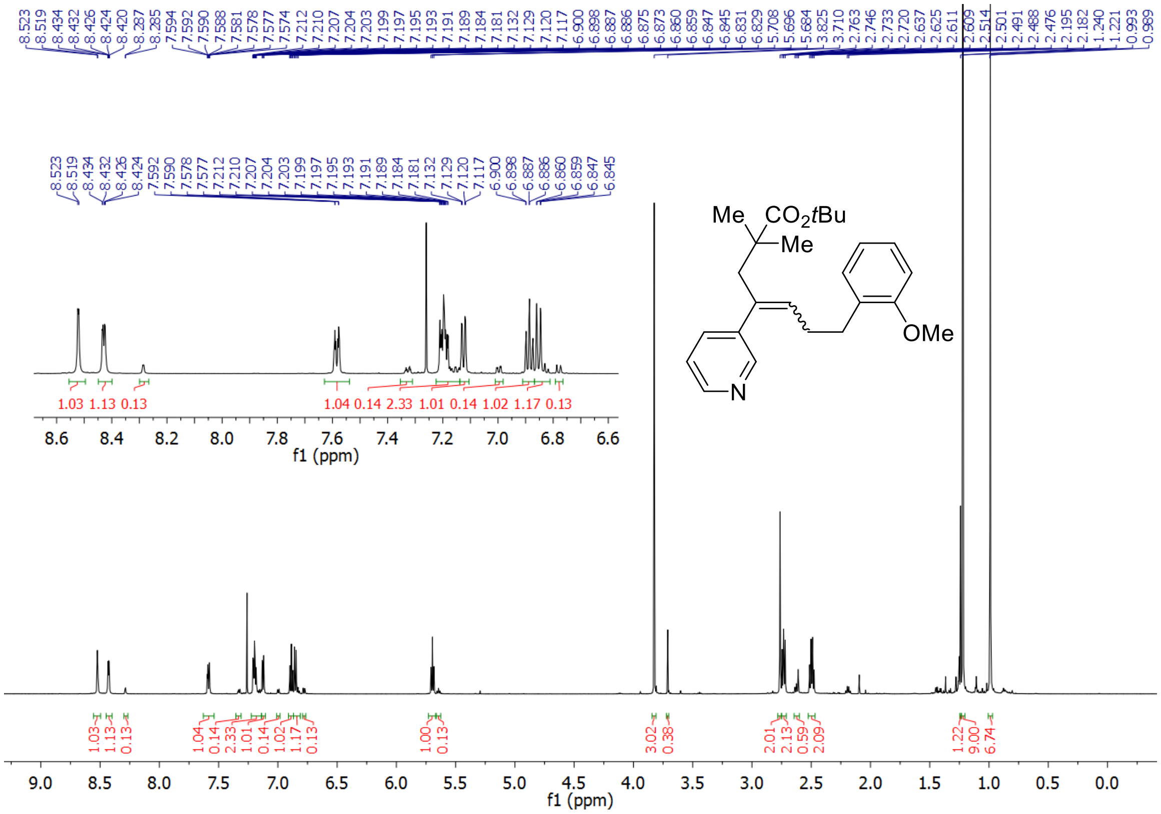

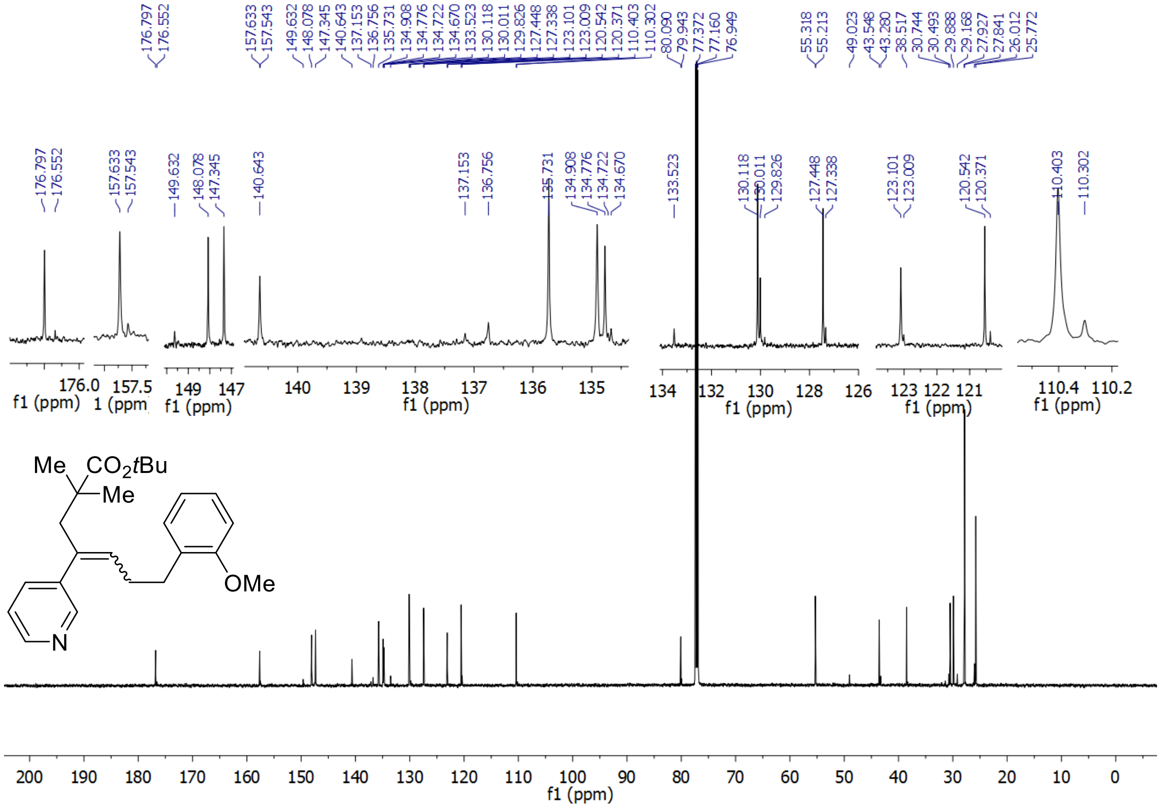

Compound 7u. Top: ^{1}H NMR ($CDCl_3$, 600 MHz). Bottom: ^{13}C NMR ($CDCl_3$, 150 MHz)

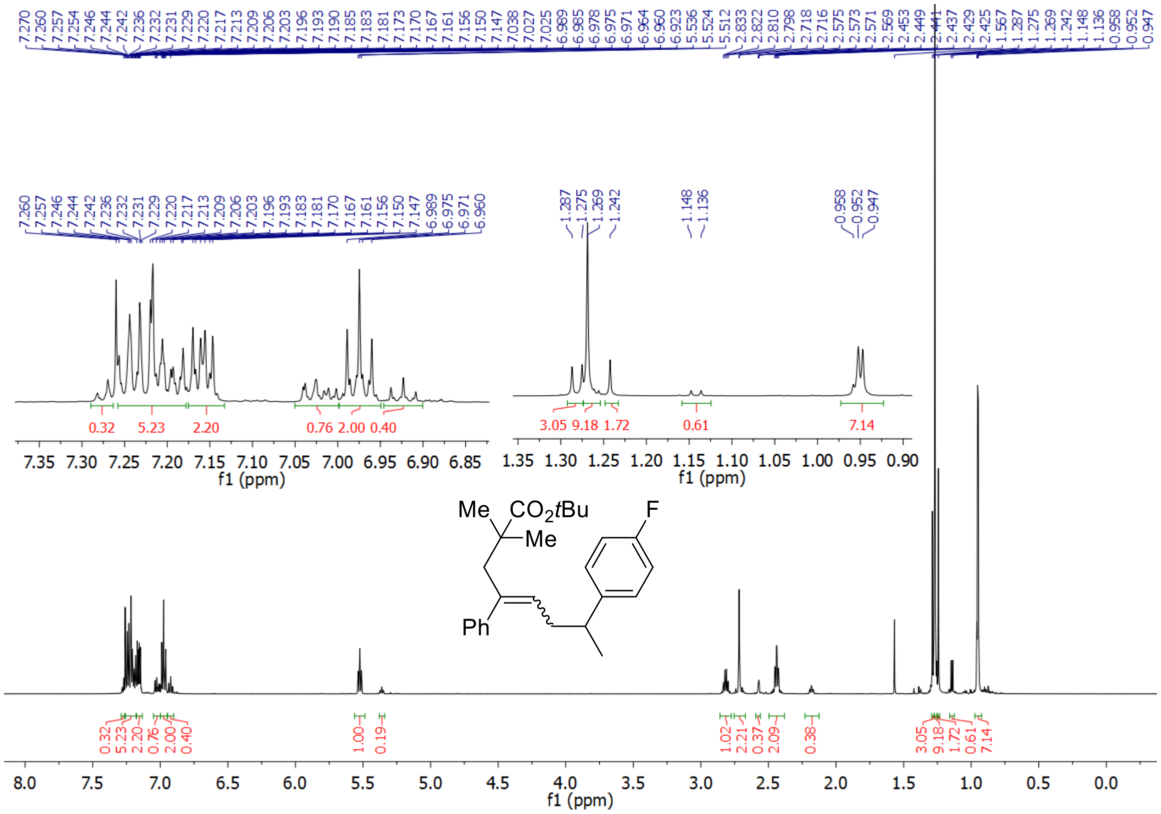

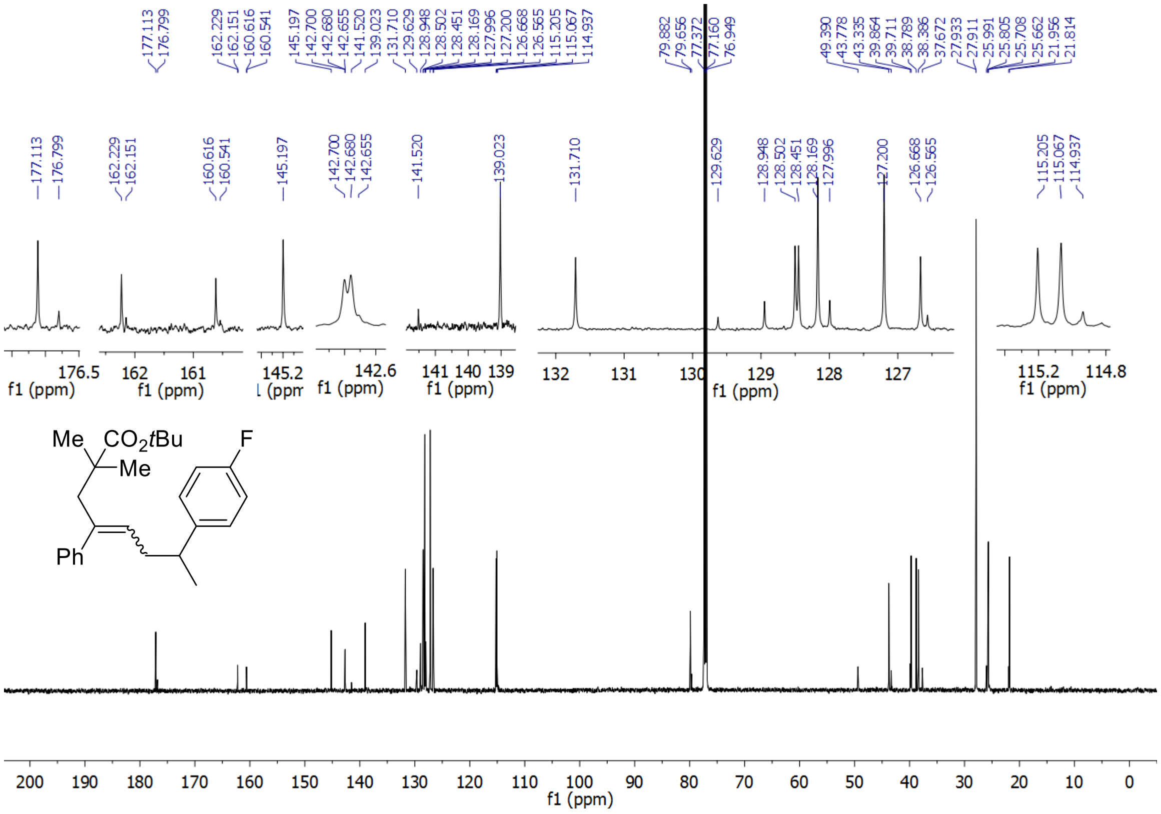

Compound 7u. ^{19}F NMR ($CDCl_3$, 565 MHz).

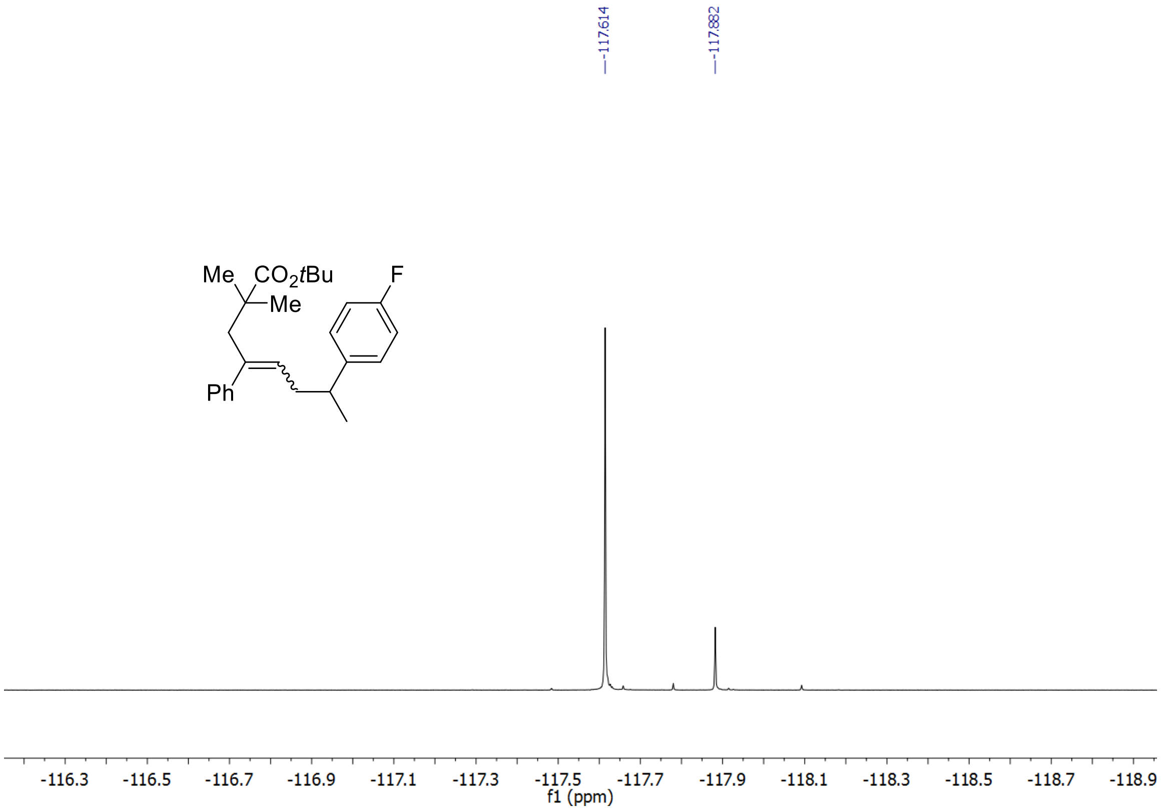

Compound 7v. Top: ^{1}H NMR ($CDCl_3$, 600 MHz). Bottom: ^{13}C NMR ($CDCl_3$, 150 MHz)

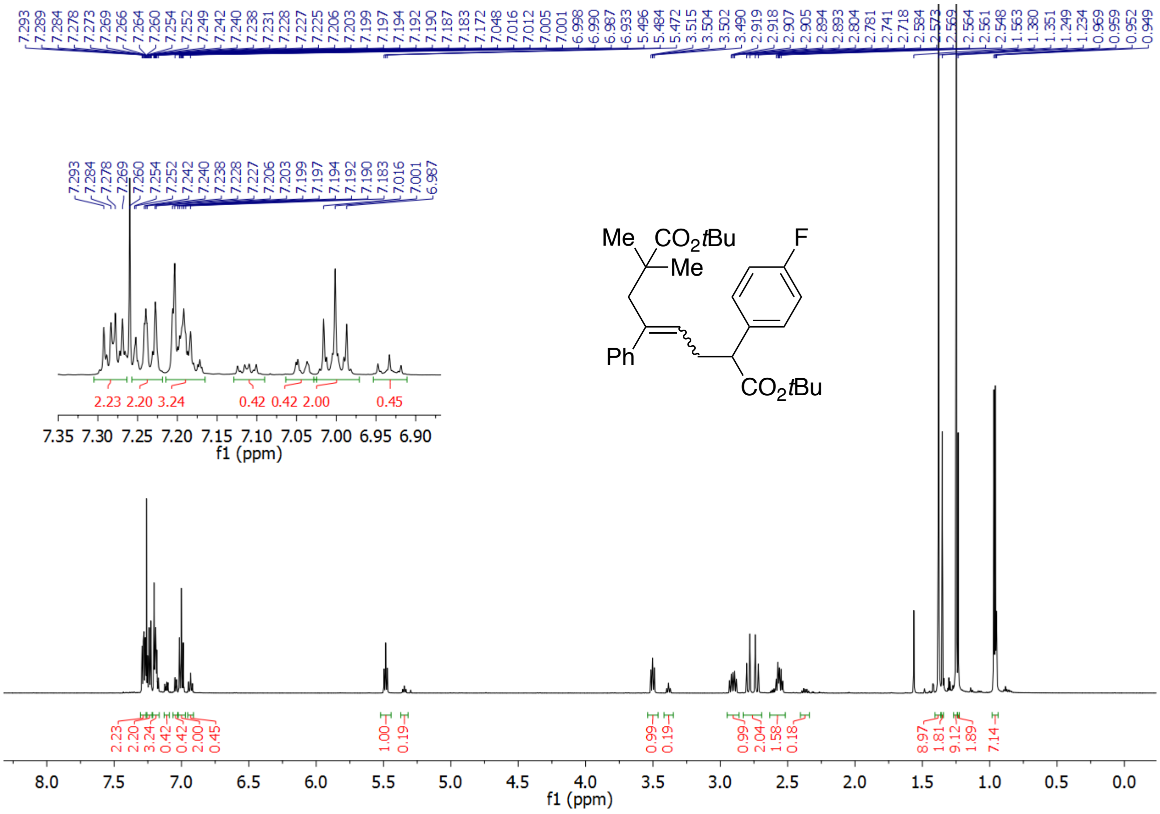

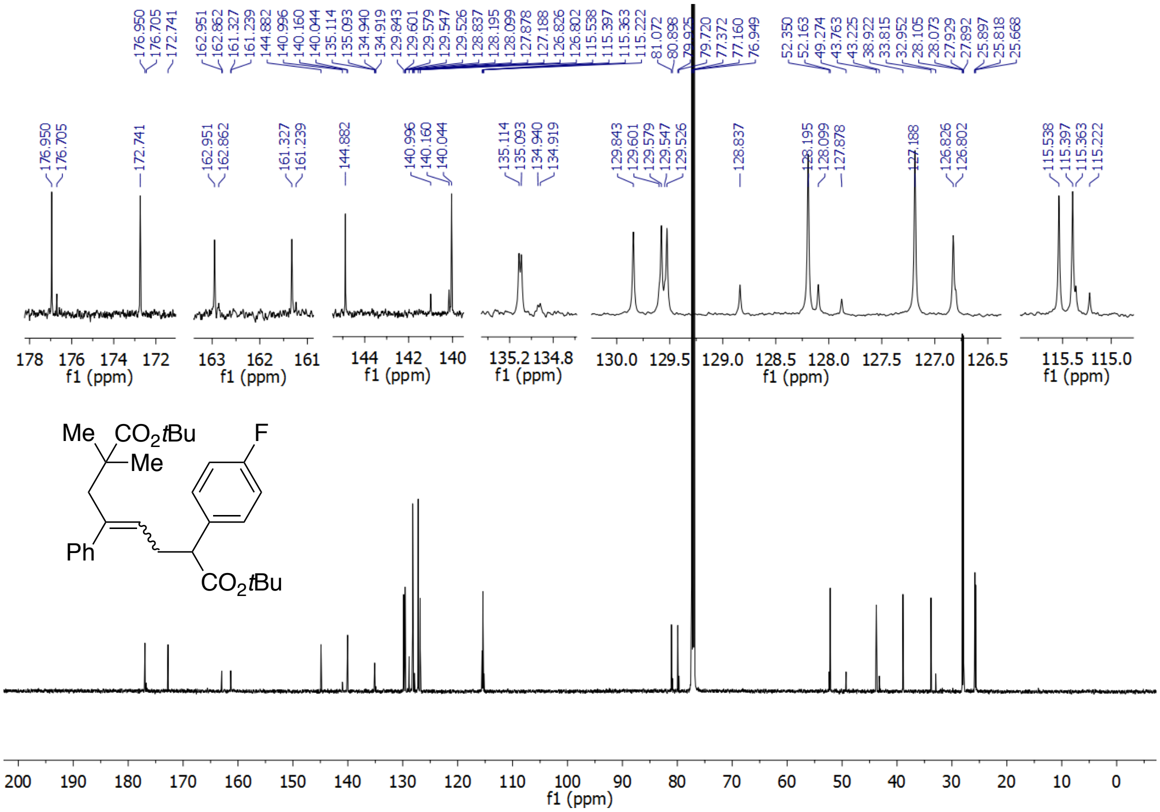

Compound 7v. ^{19}F NMR ($CDCl_3$, 565 MHz).

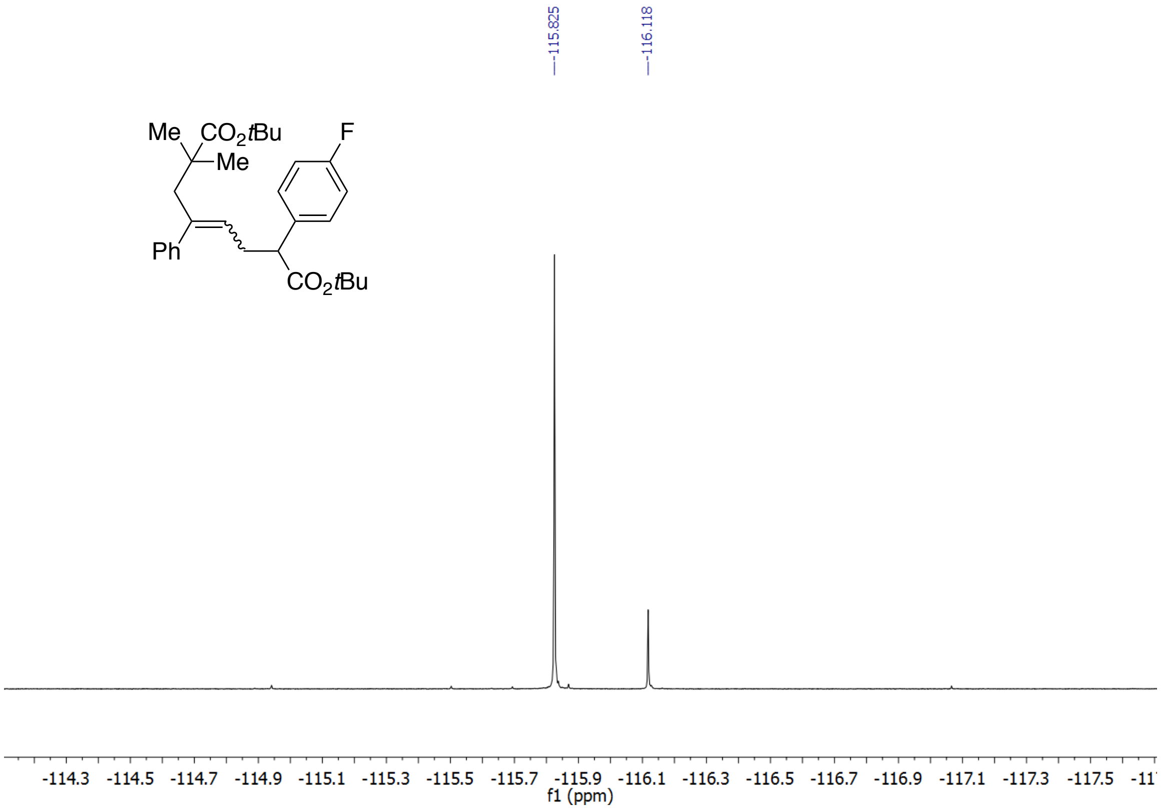

Compound 7x. Top: ^{1}H NMR ($CDCl_3$, 600 MHz). Bottom: ^{13}C NMR ($CDCl_3$, 150 MHz)

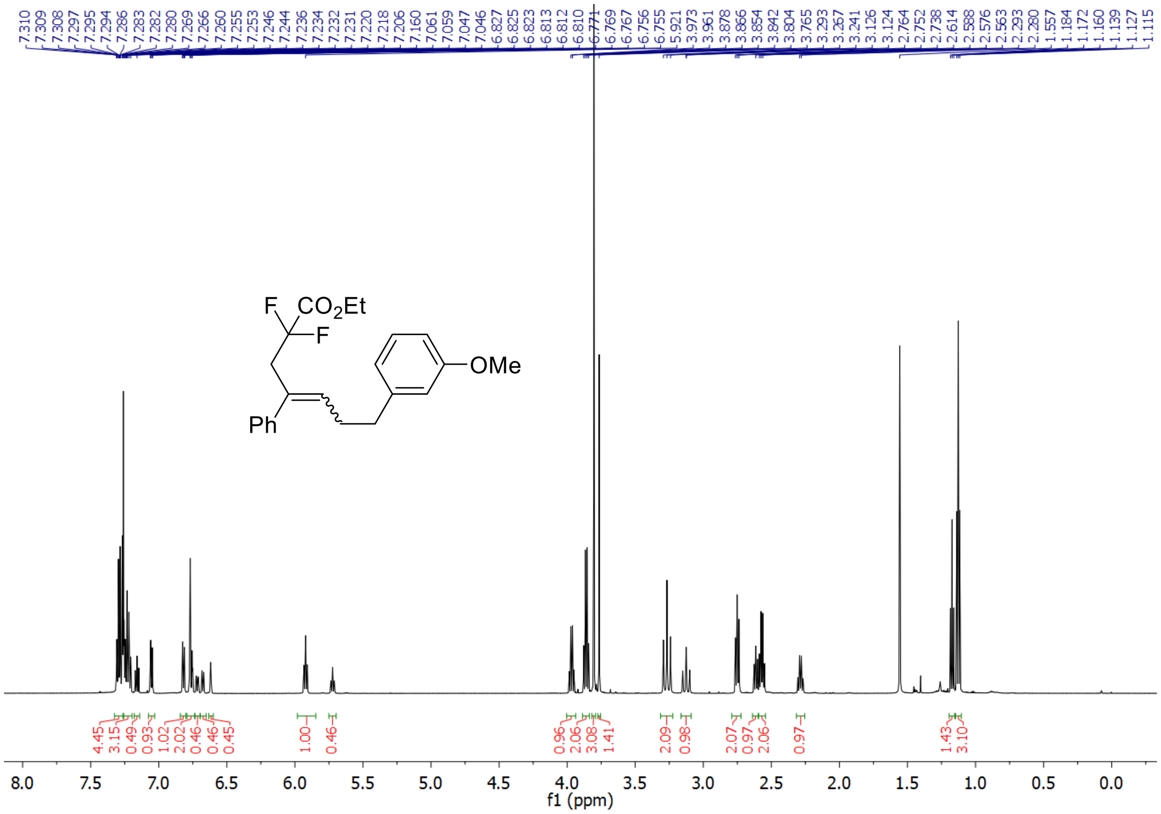

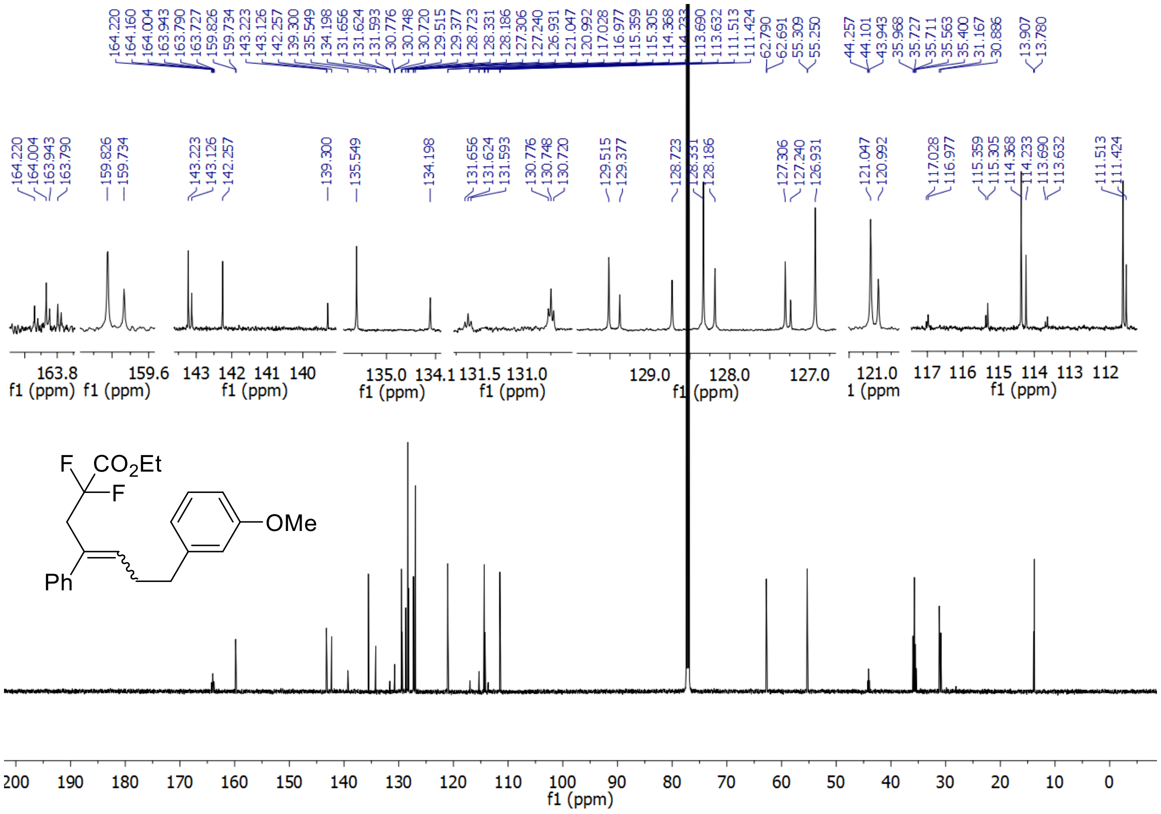

Compound 7x. ^{19}F NMR ($CDCl_3$, 565 MHz).

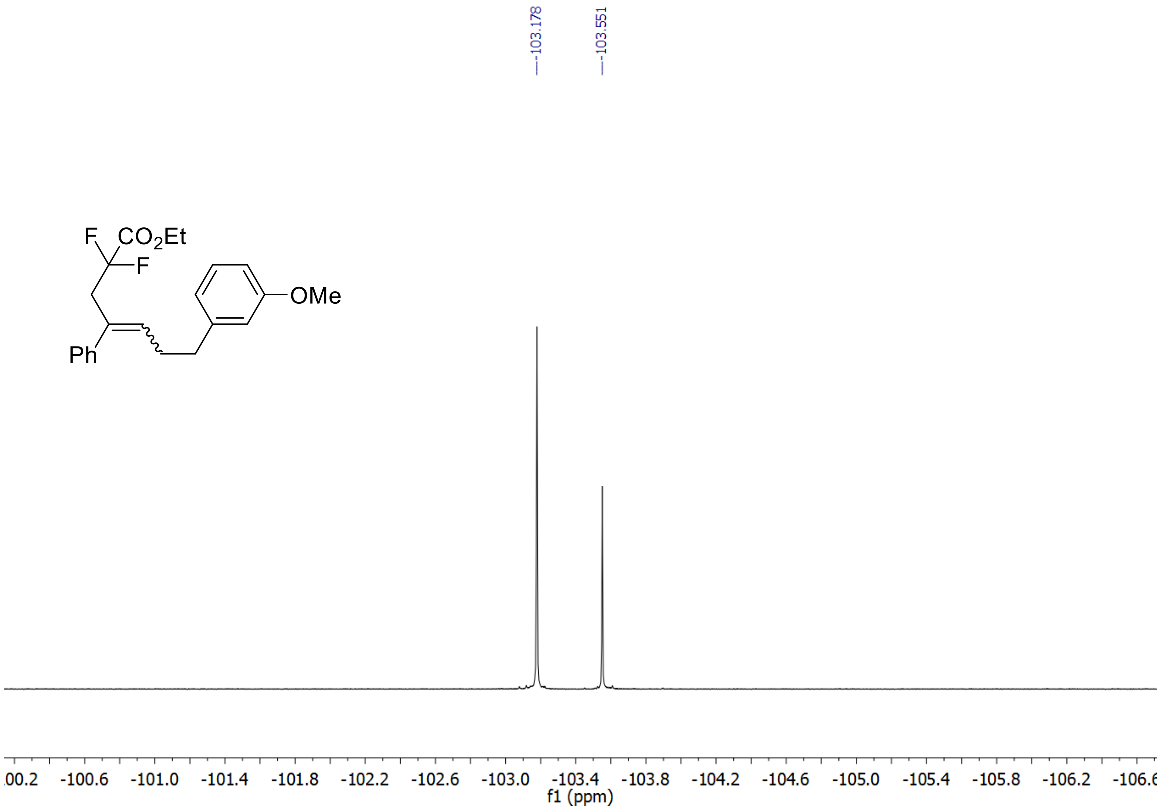

Compound (*E*)-7y. Top: ^{1}H NMR ($CDCl_3$, 600 MHz). Bottom: ^{13}C NMR ($CDCl_3$, 150 MHz)

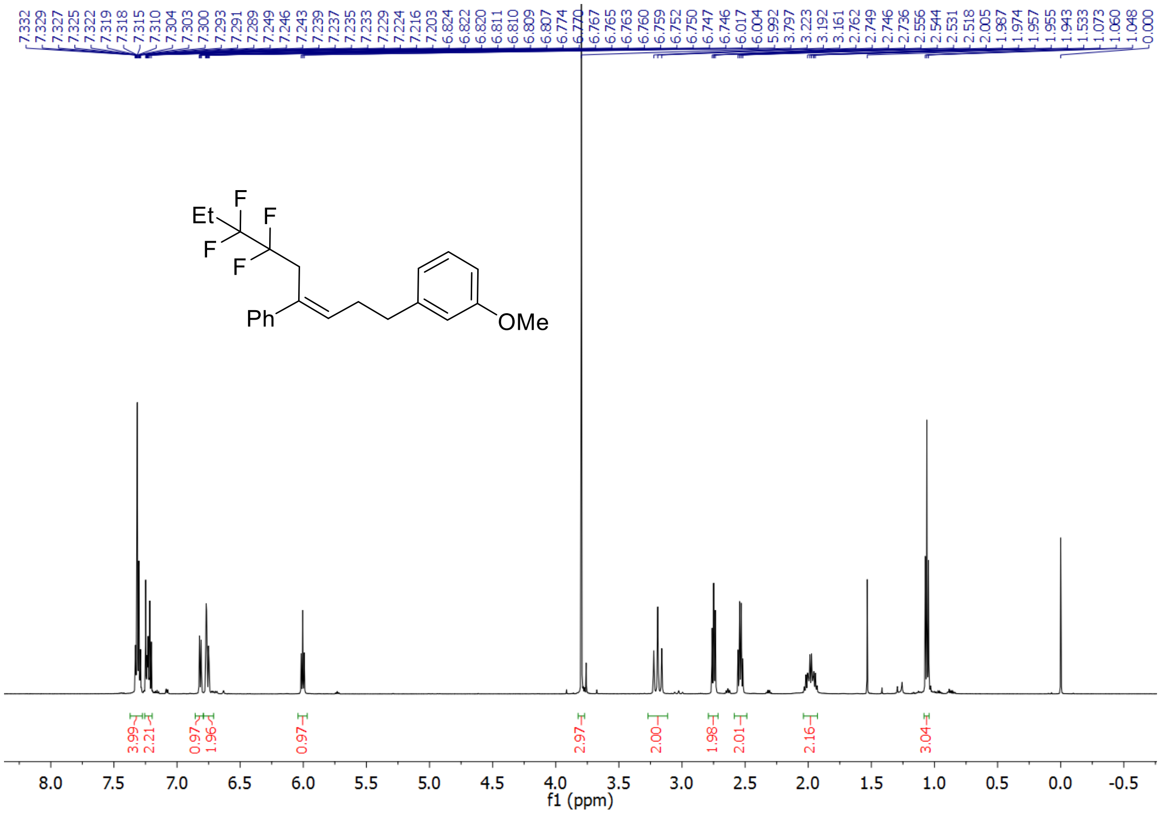

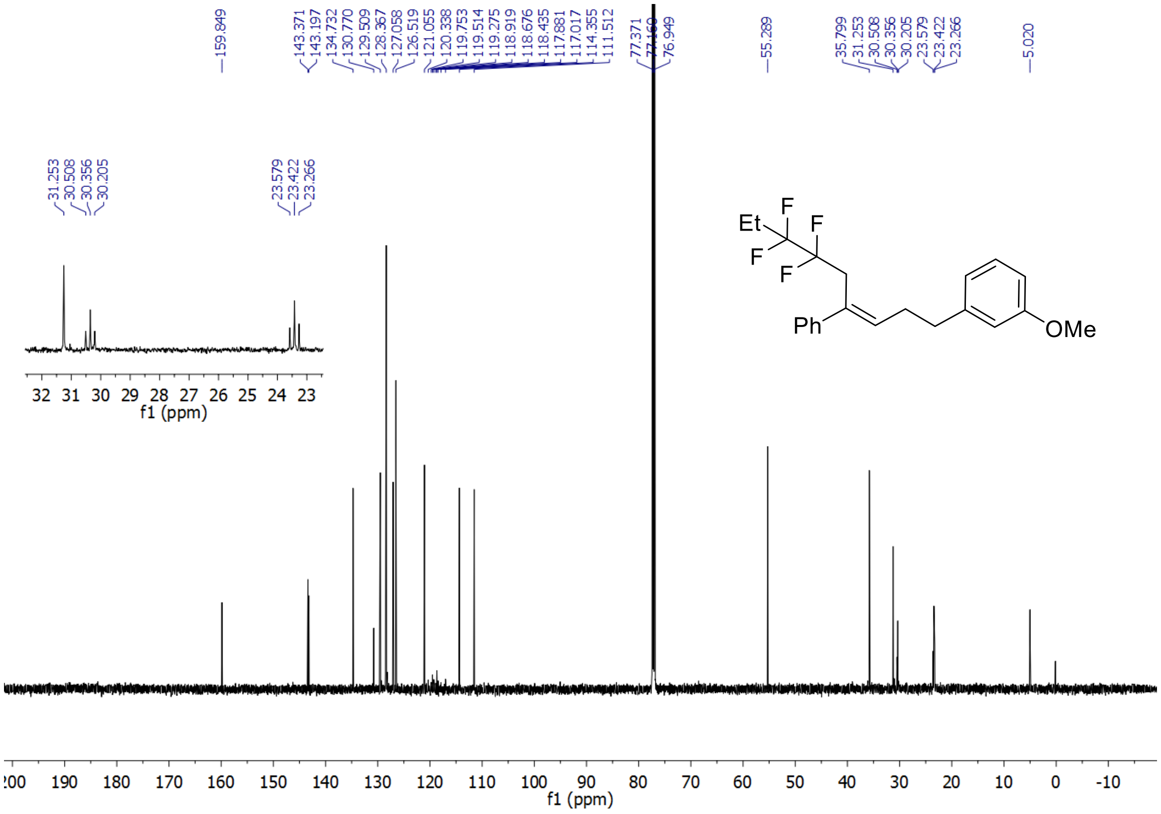

Compound (*E*)-7y. ^{19}F NMR ($CDCl_3$, 565 MHz).

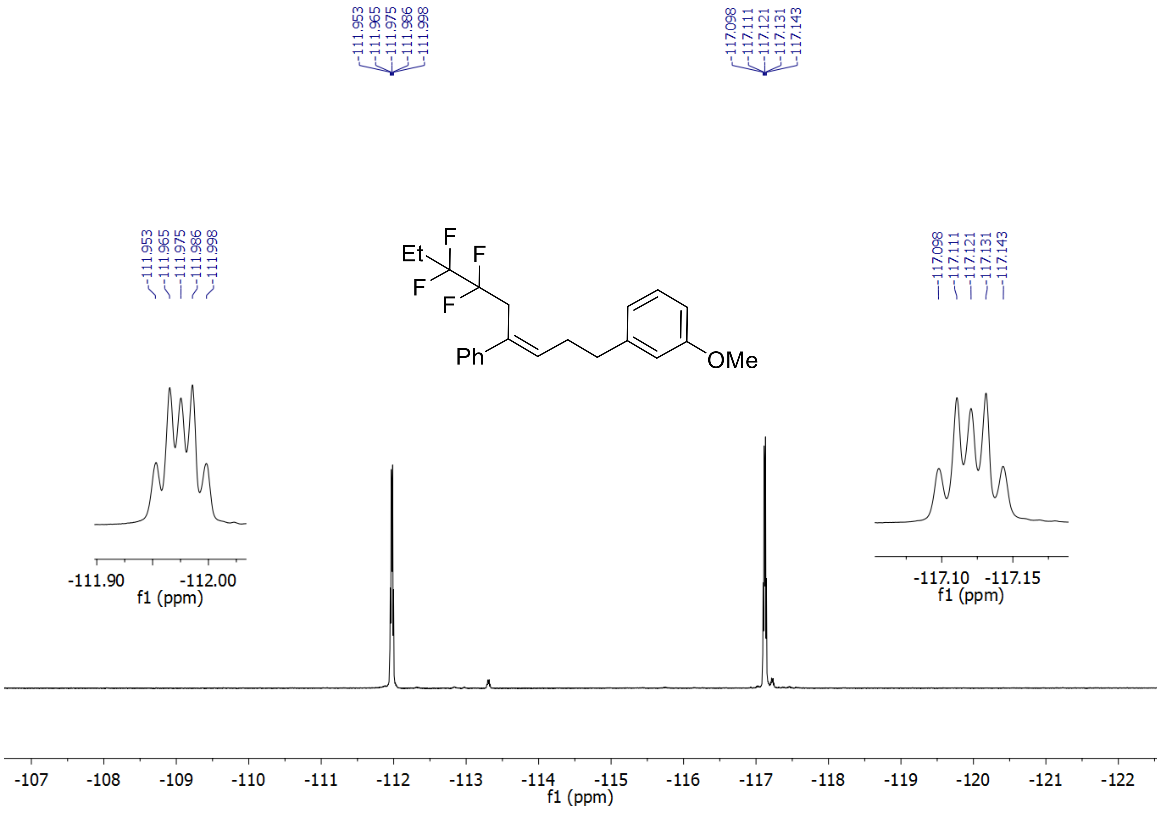

Compound (*E*)-7z. Top: ^{1}H NMR ($CDCl_3$, 600 MHz). Bottom: ^{13}C NMR ($CDCl_3$, 150 MHz)

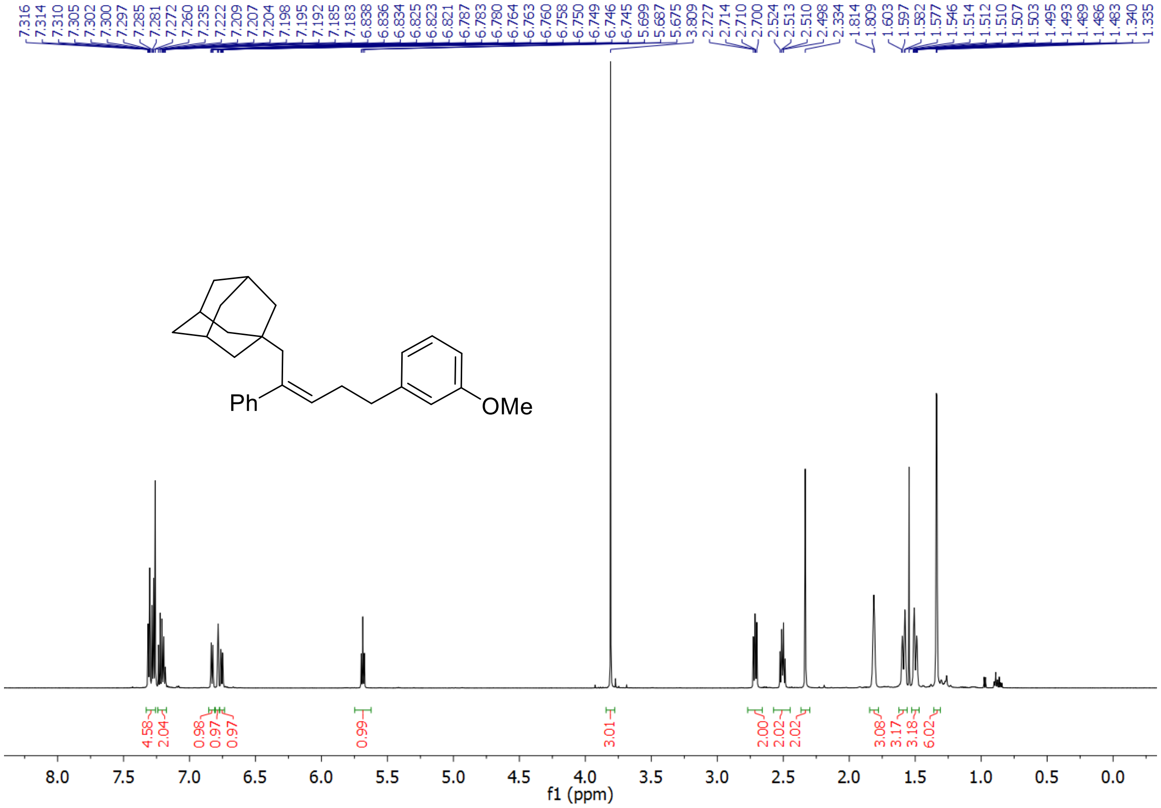

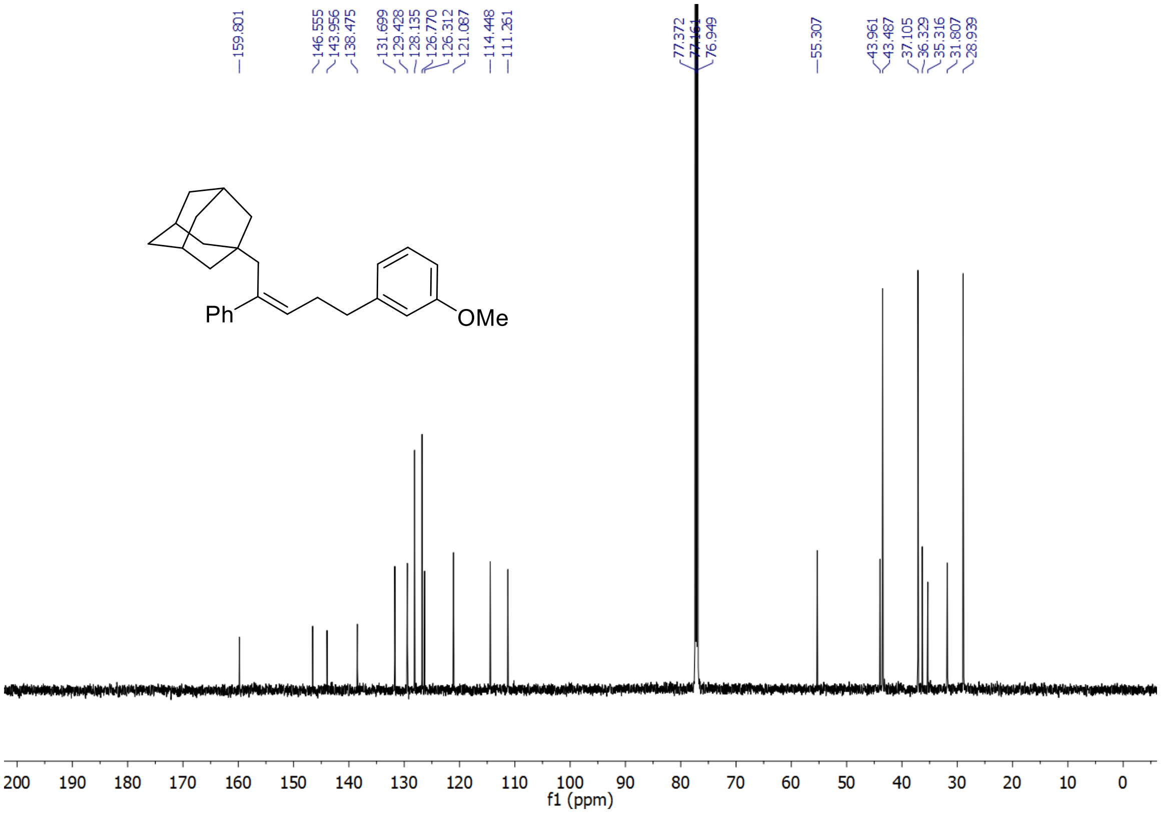

Compound (*E*)-7za. Top: ^{1}H NMR ($CDCl_3$, 600 MHz). Bottom: ^{13}C NMR ($CDCl_3$, 150 MHz)

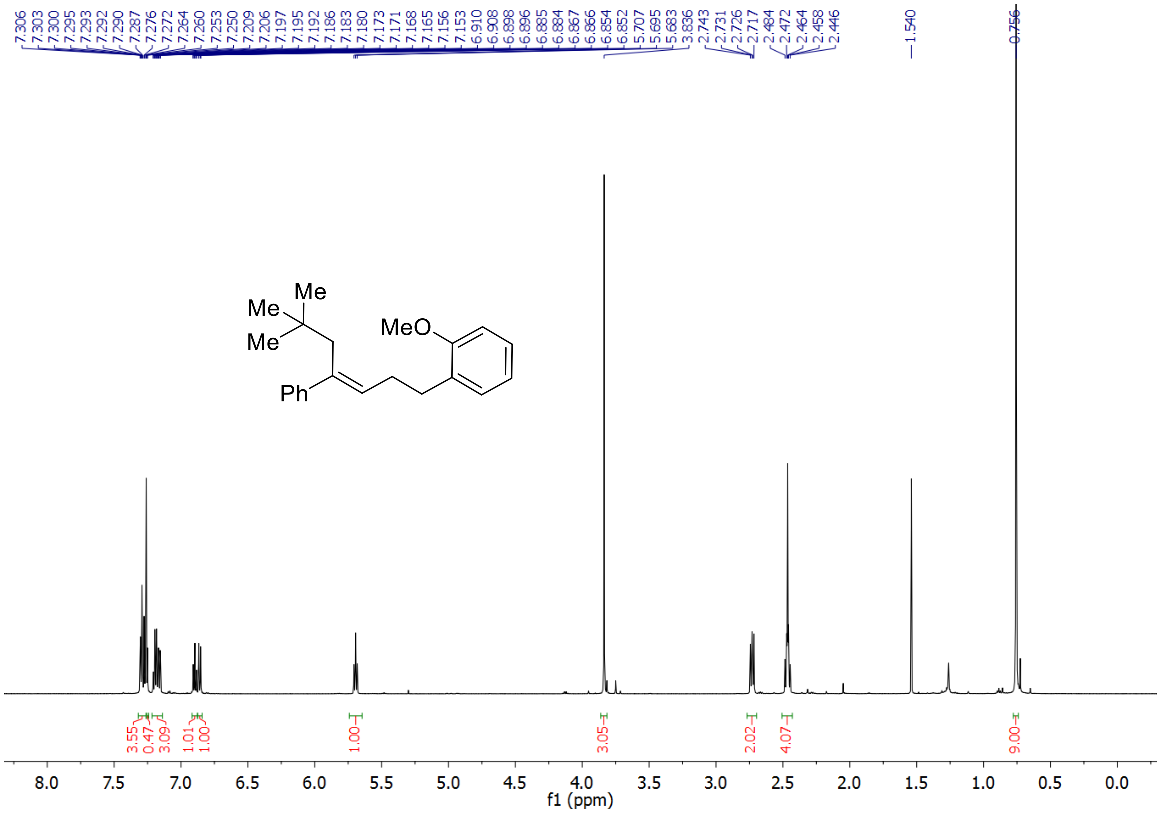

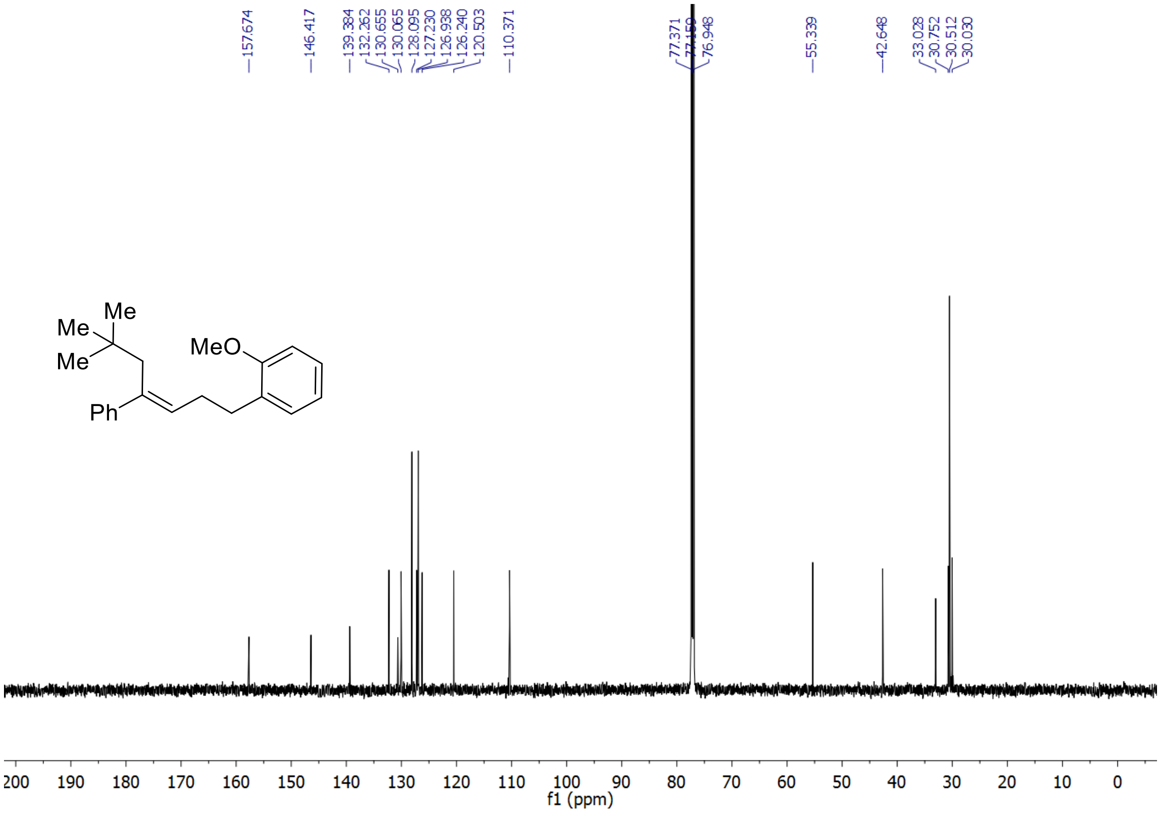

Compound 7zb. Top: ^{1}H NMR ($CDCl_3$, 600 MHz). Bottom: ^{13}C NMR ($CDCl_3$, 150 MHz)

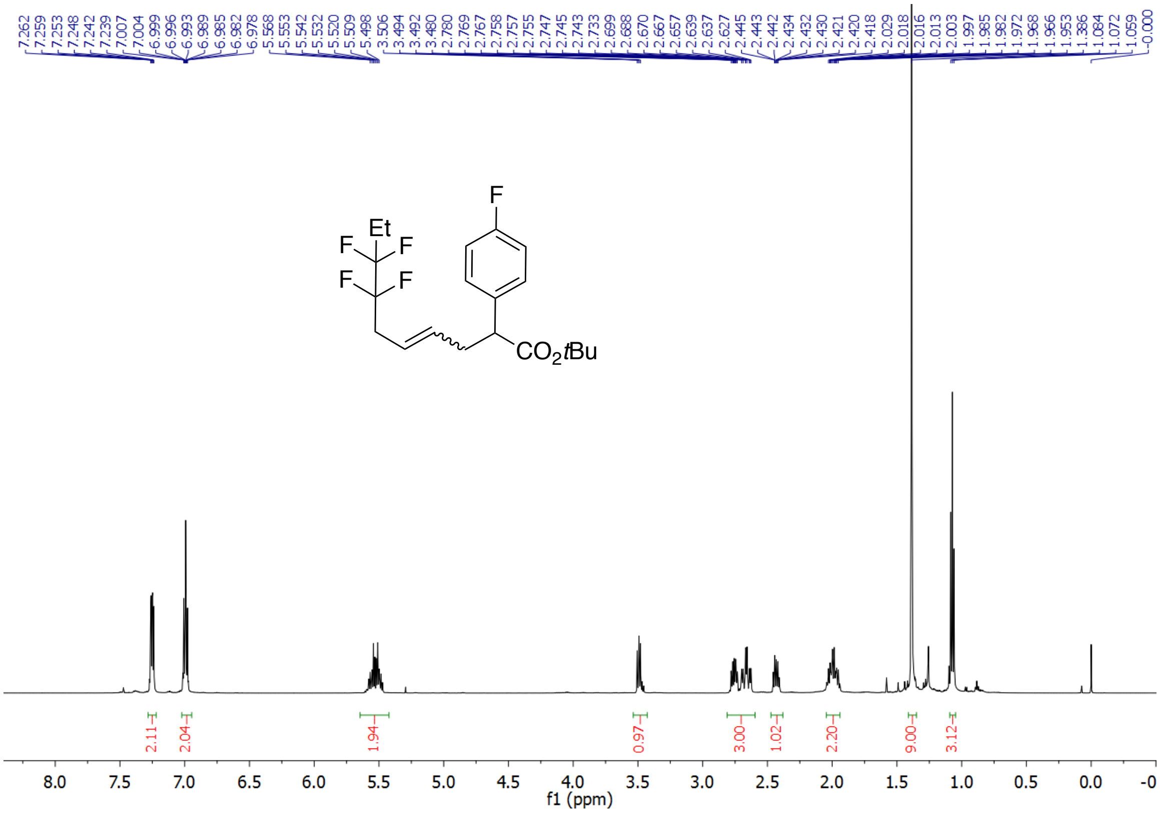

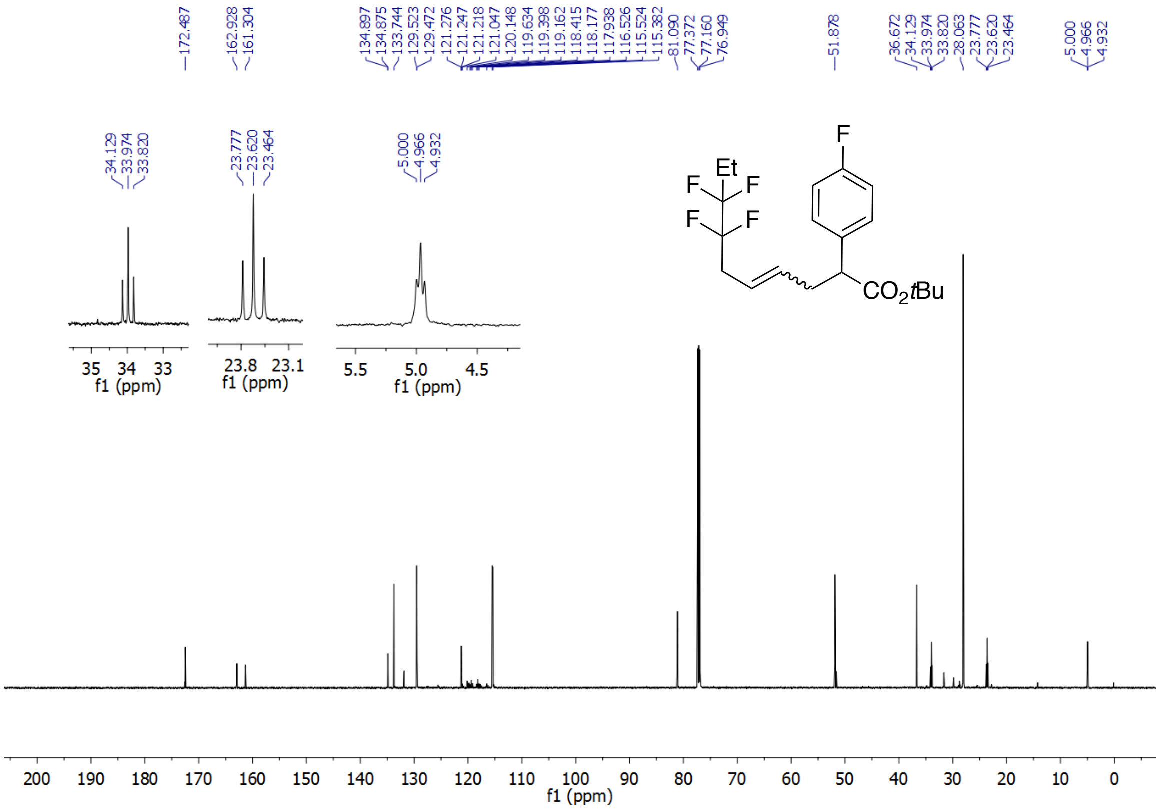

Compound 7zb. ^{19}F NMR ($CDCl_3$, 565 MHz).

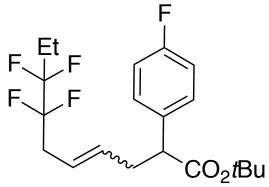

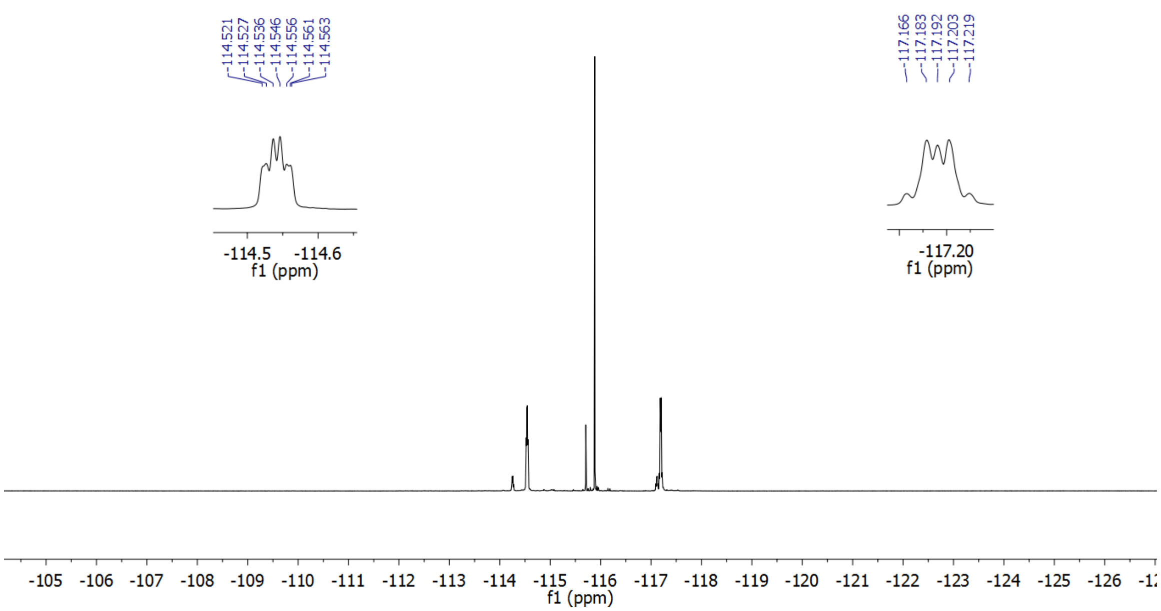

Compound 7zc. Top: ^{1}H NMR ($CDCl_3$, 600 MHz). Bottom: ^{13}C NMR ($CDCl_3$, 150 MHz)

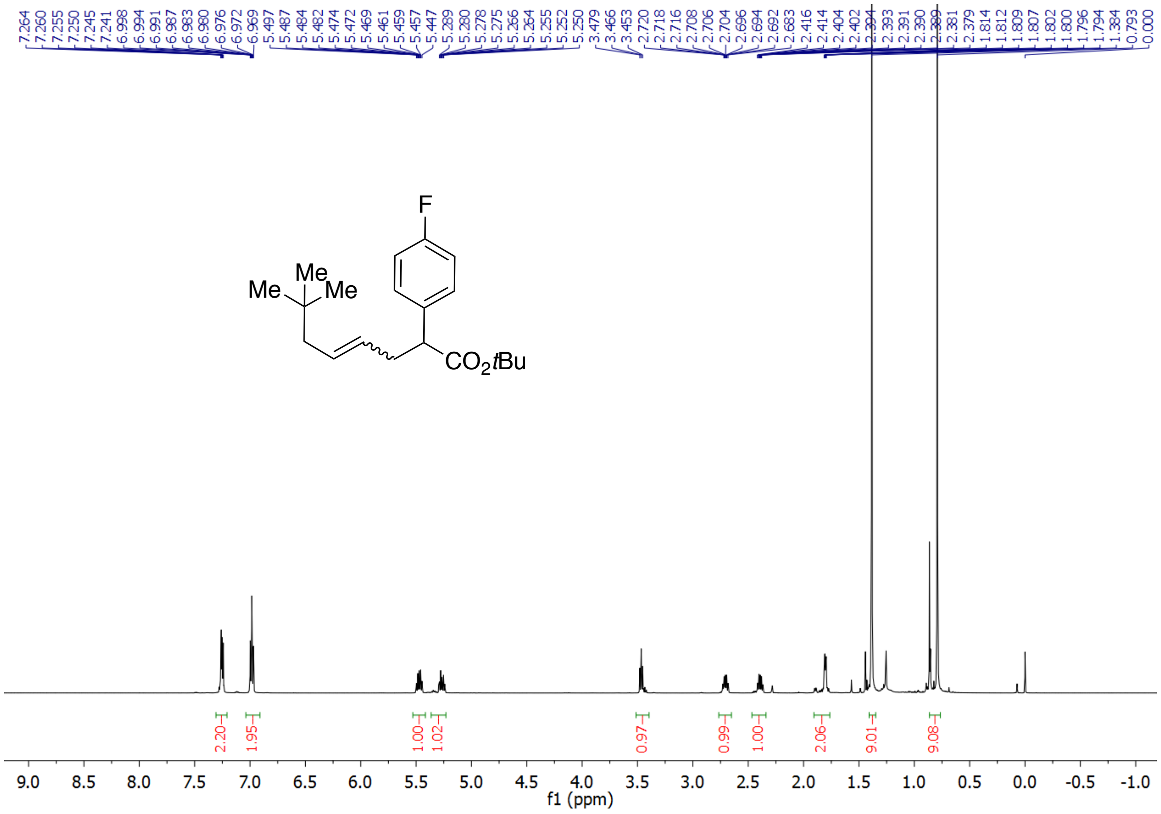

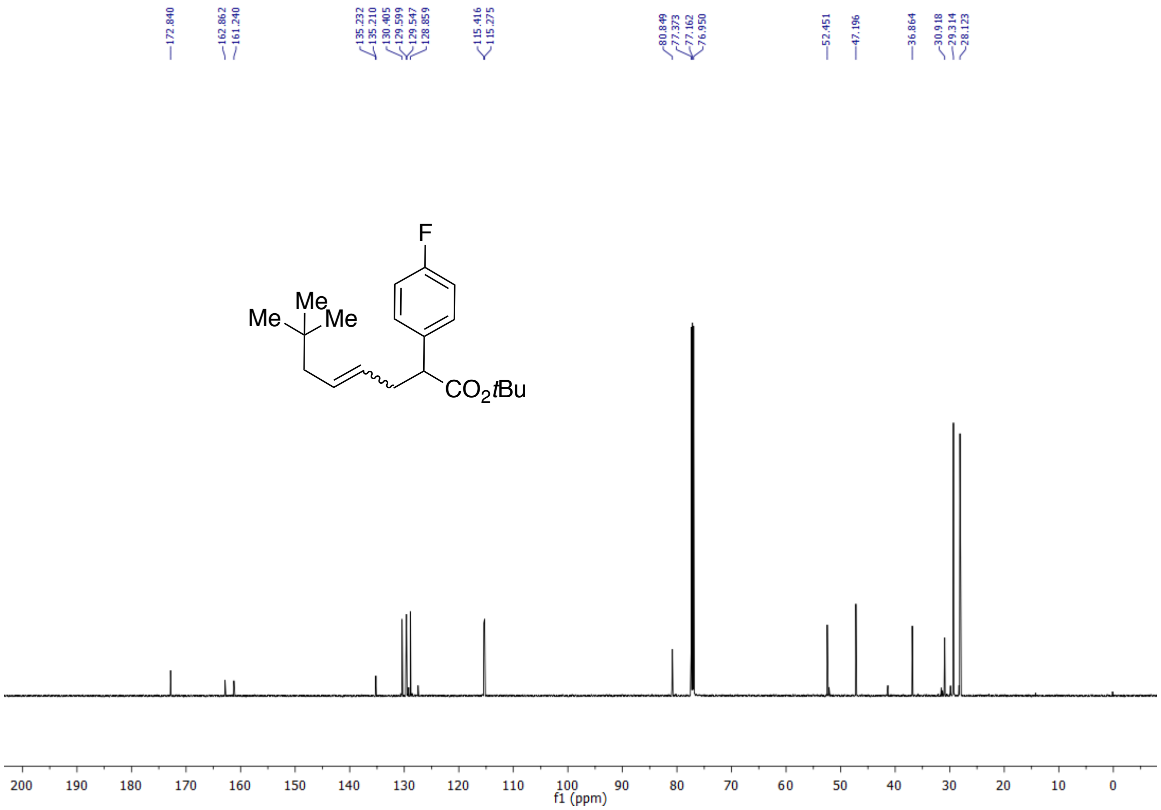

Compound 7zc. ^{19}F NMR ($CDCl_3$, 565 MHz).

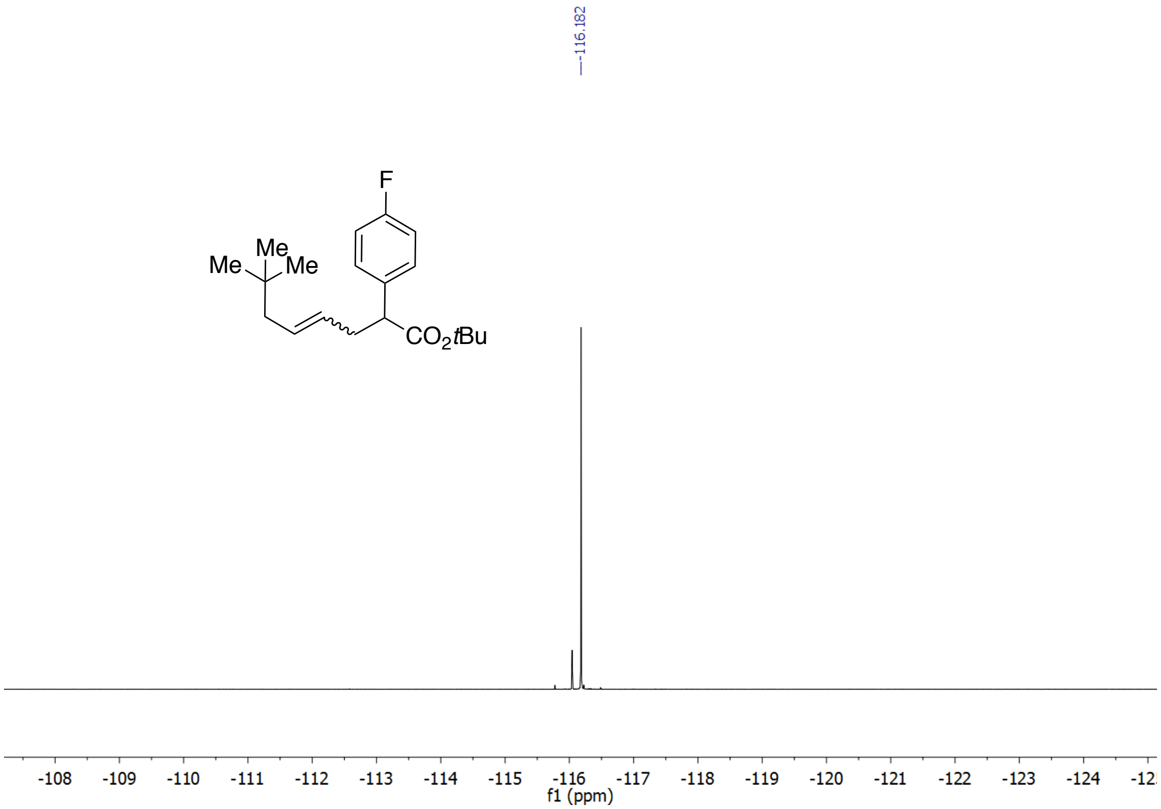

Compound 7zd. Top: ^{1}H NMR ($CDCl_3$, 600 MHz). Bottom: ^{13}C NMR ($CDCl_3$, 150 MHz)

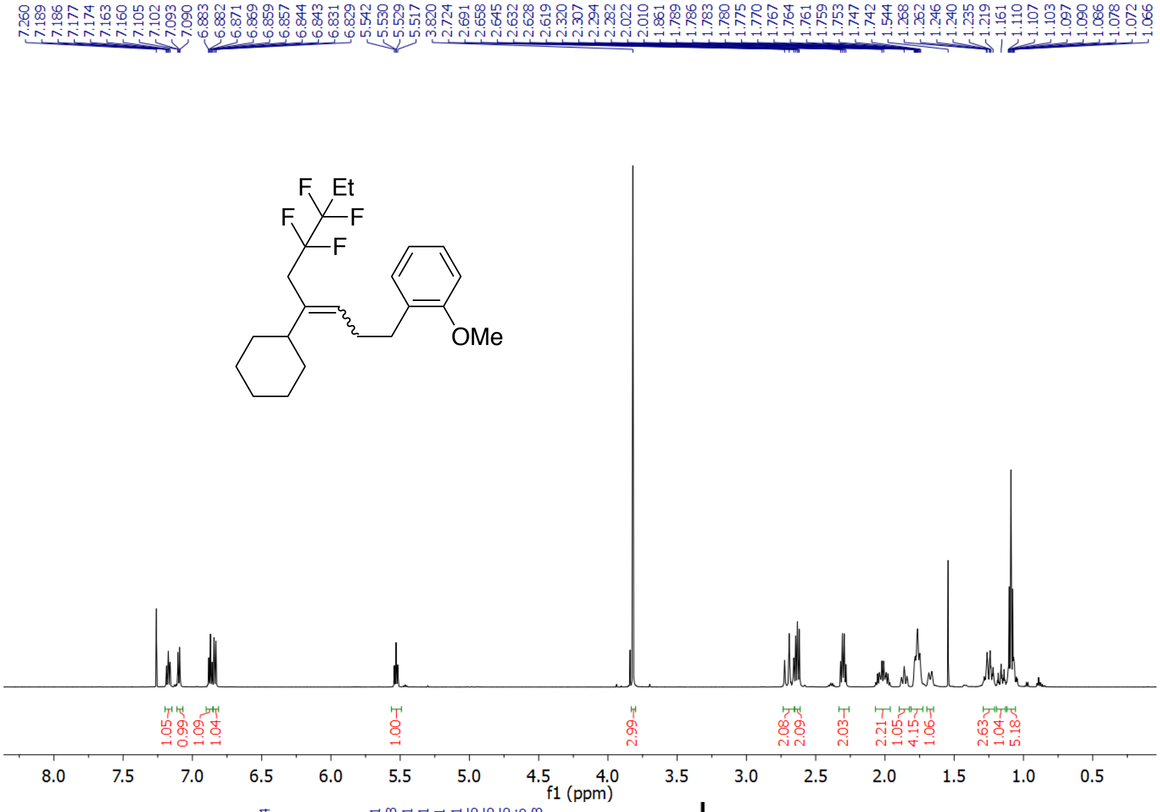

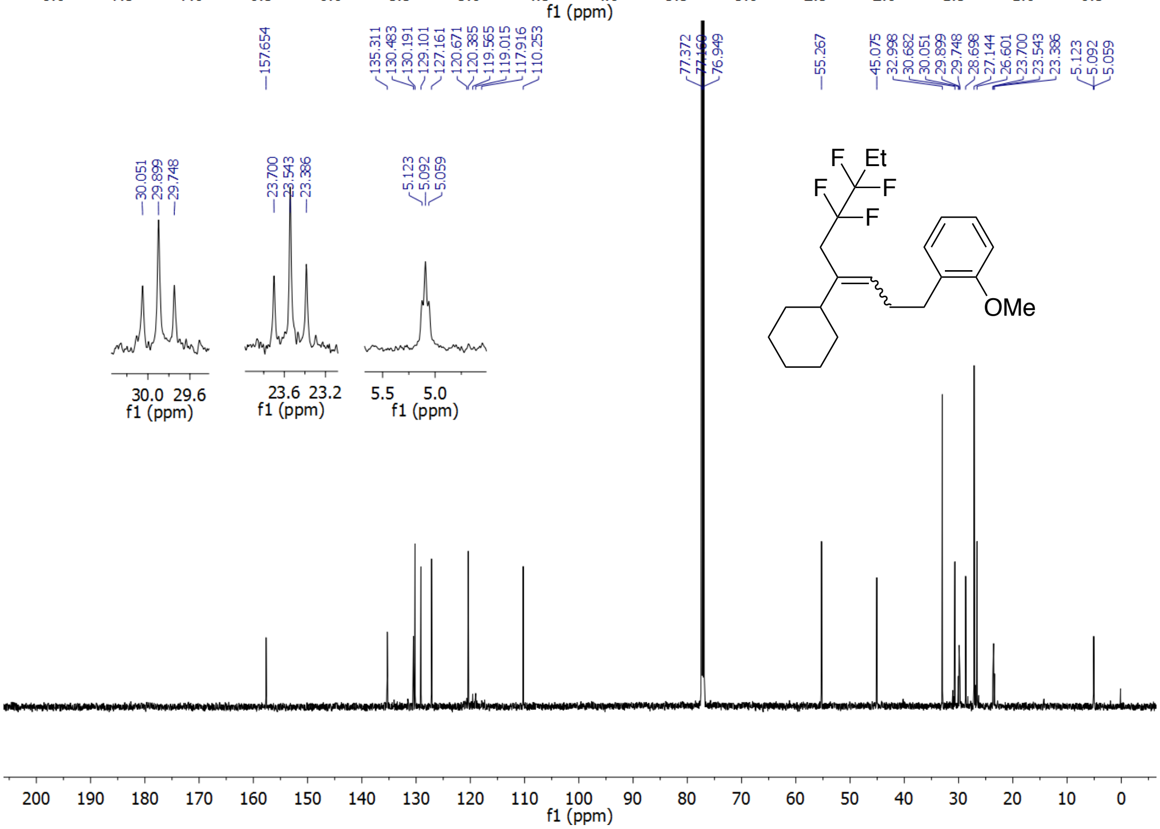

Compound 7zd. ^{19}F NMR ($CDCl_3$, 565 MHz).

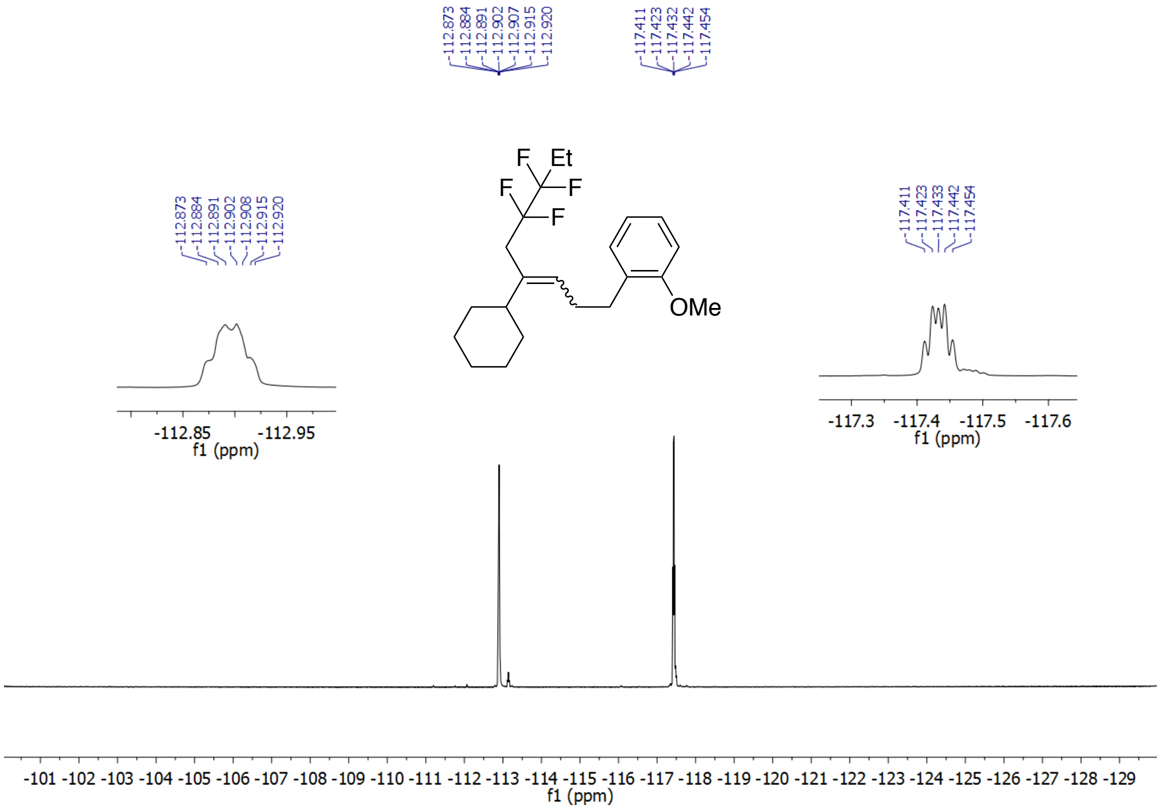

Compound (*E*)-7ze. Top: ^{1}H NMR ($CDCl_3$, 600 MHz). Bottom: ^{13}C NMR ($CDCl_3$, 150 MHz)

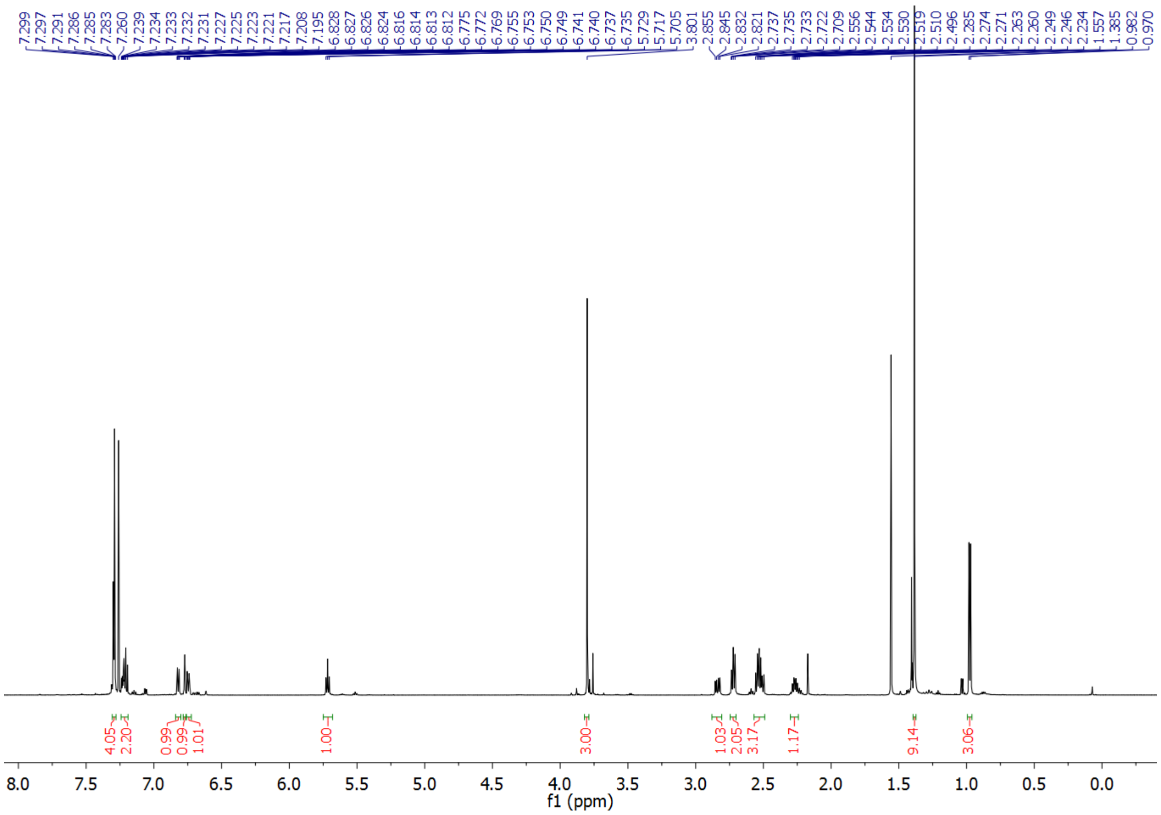

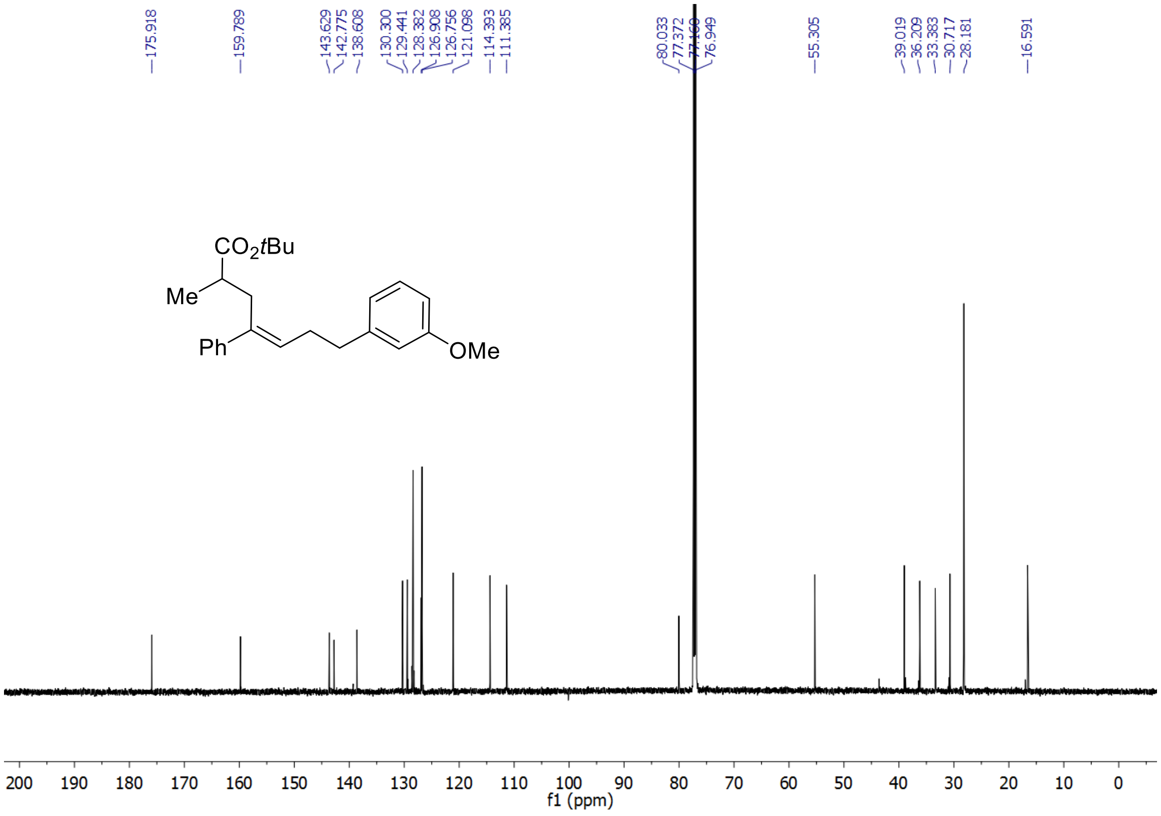

Compound (*E*)-8a. Top: ^{1}H NMR ($CDCl_3$, 600 MHz). Bottom: ^{13}C NMR ($CDCl_3$, 150 MHz)

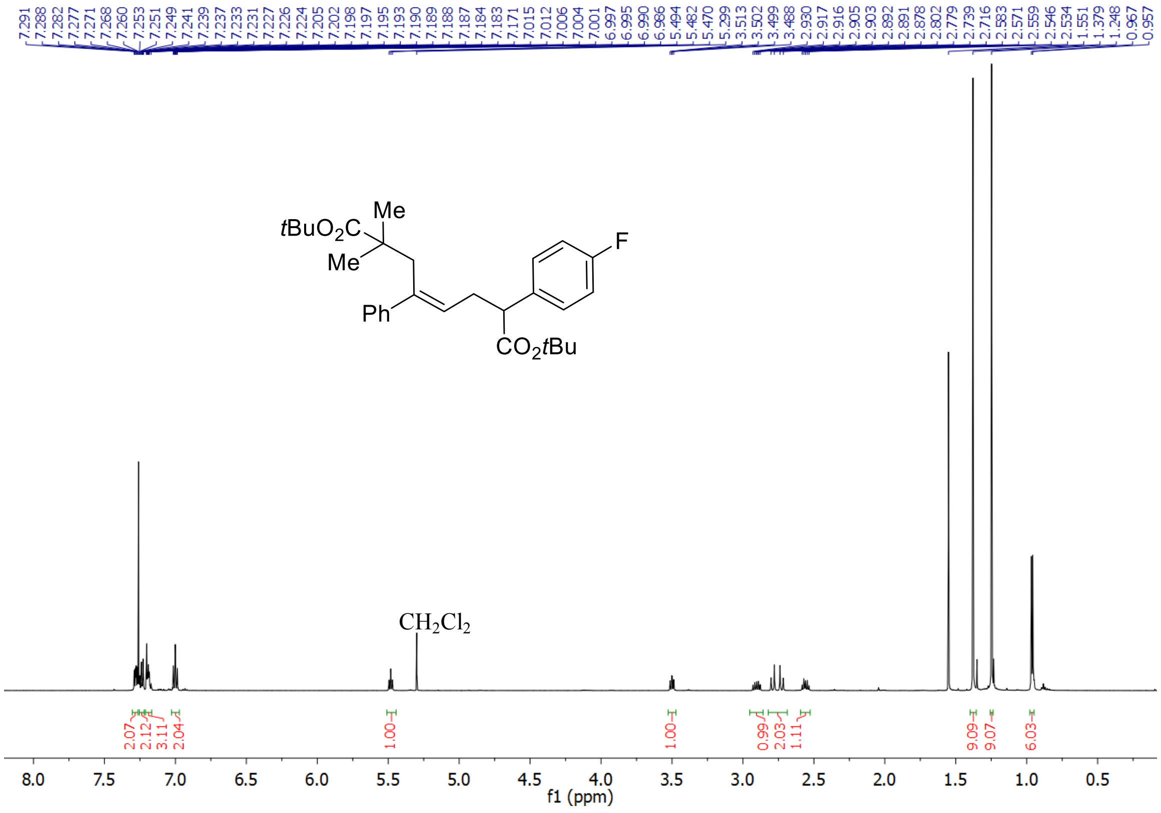

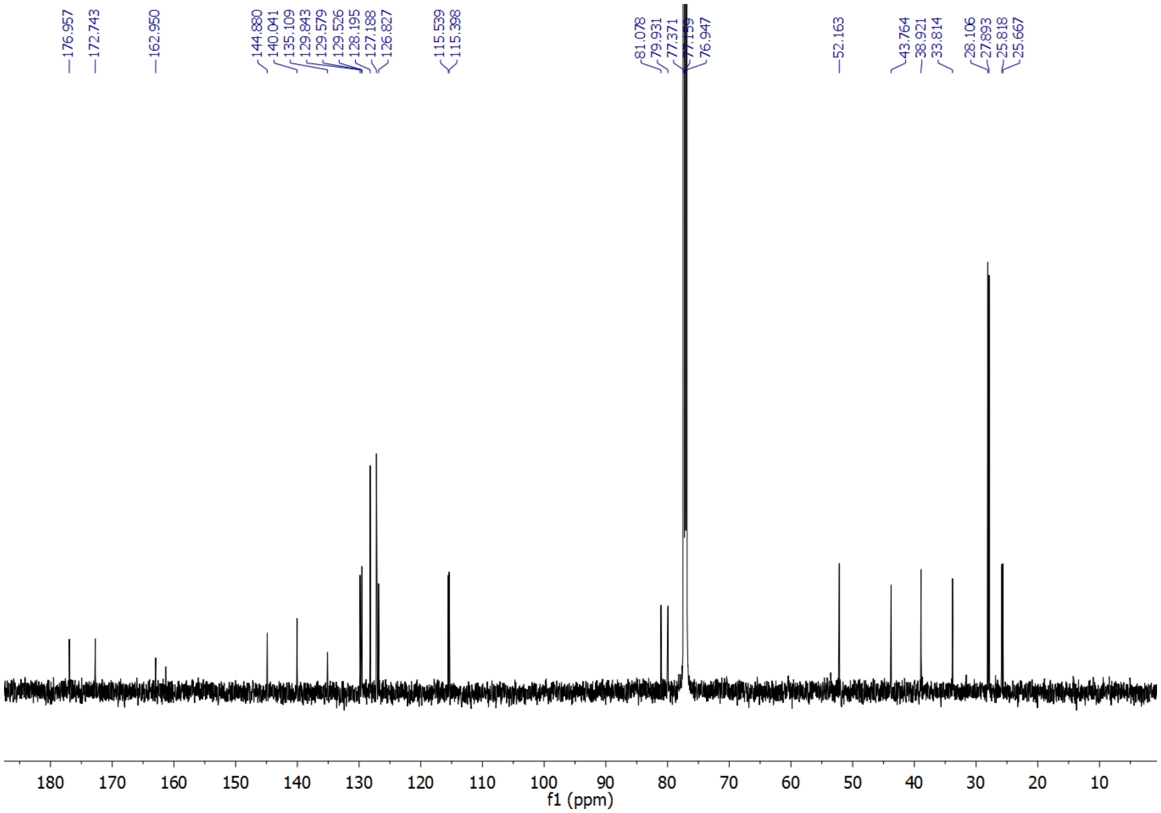

Compound (*E*)-8a. ^{19}F NMR ($CDCl_3$, 565 MHz).

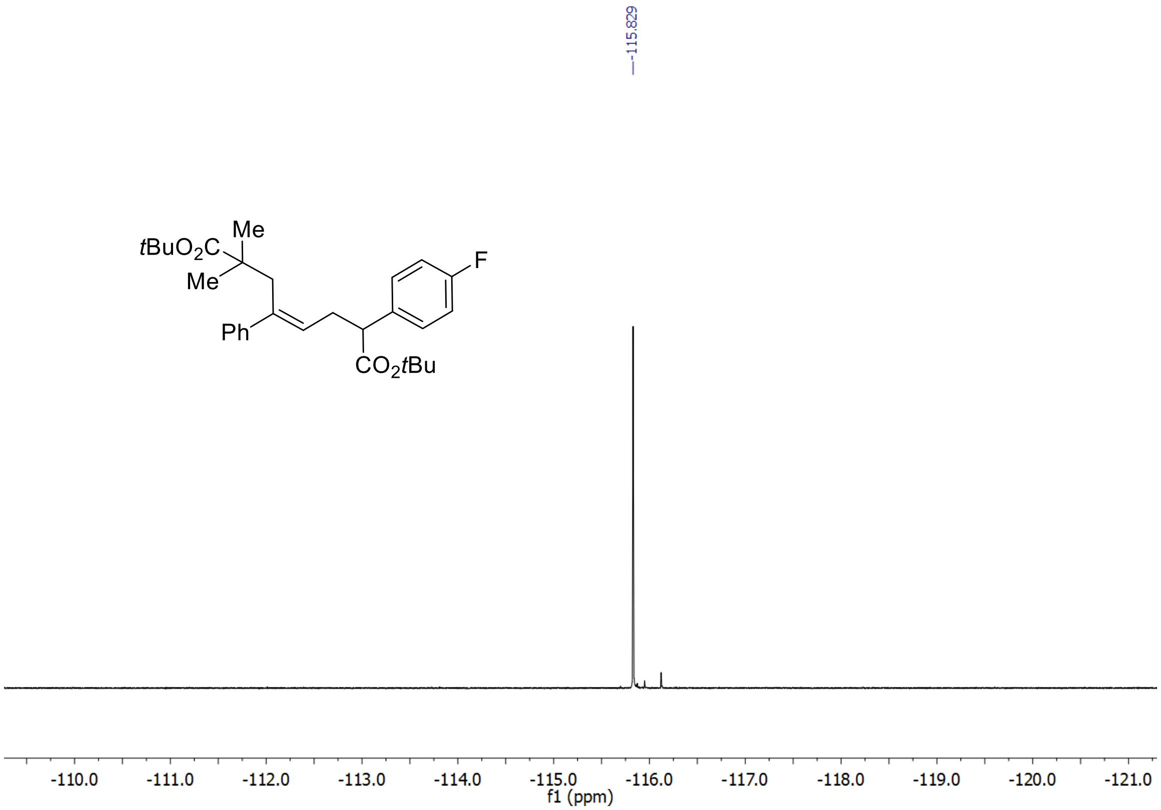

Compound 9. Top: ^1H NMR ($CDCl_3$, 600 MHz). Bottom: ^{13}C NMR ($CDCl_3$, 150 MHz)

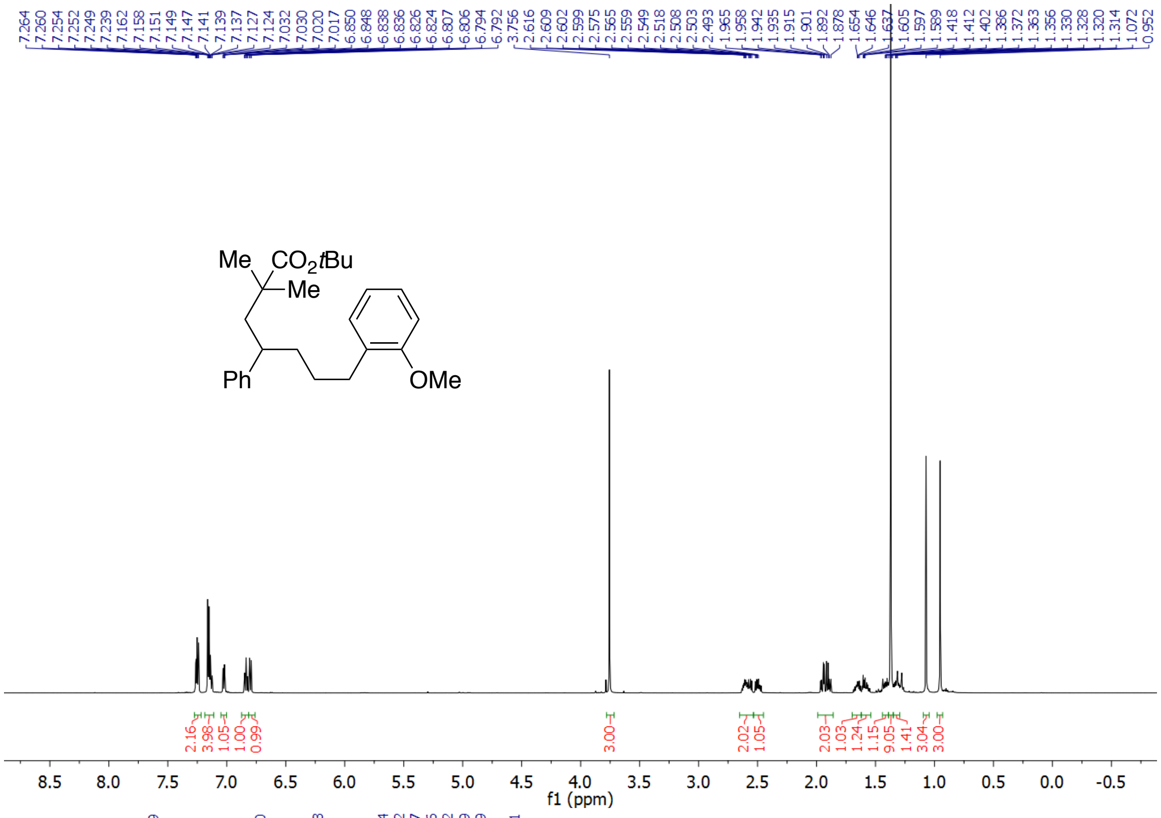

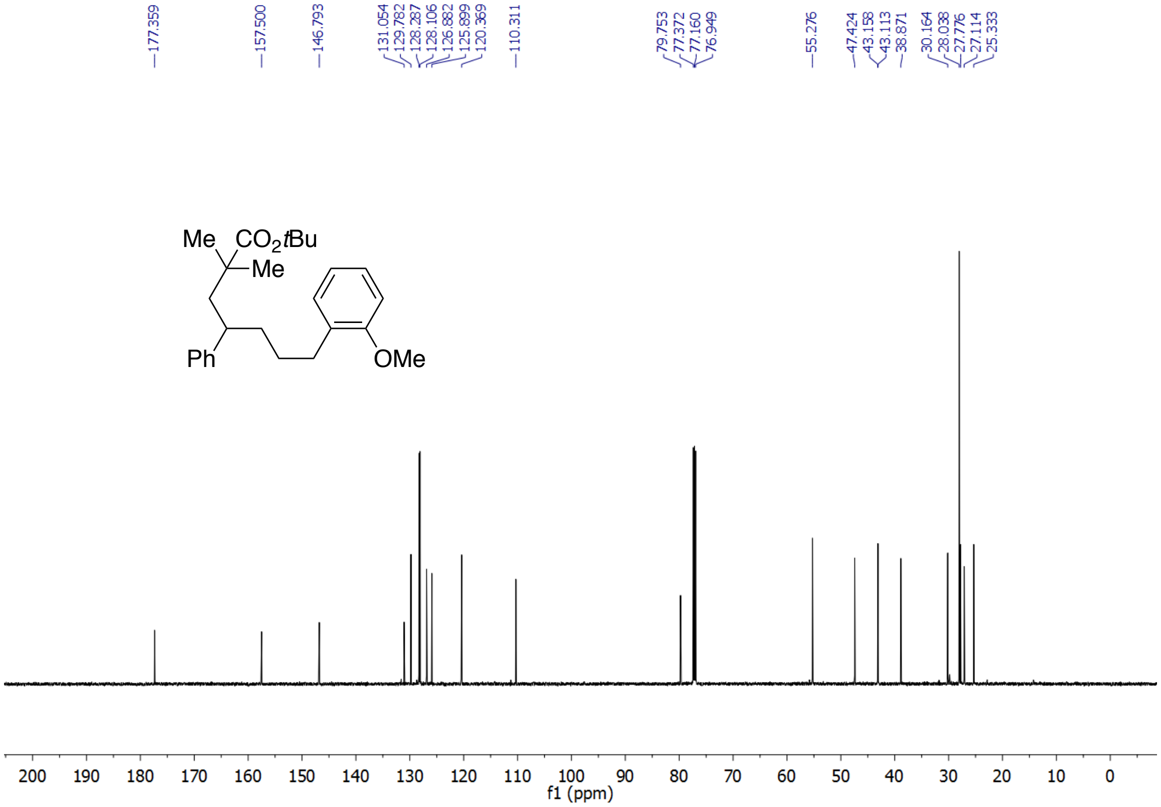

Compound (*E*)-10. Top: ^{1}H NMR ($CDCl_3$, 600 MHz). Bottom: ^{13}C NMR ($CDCl_3$, 150 MHz)

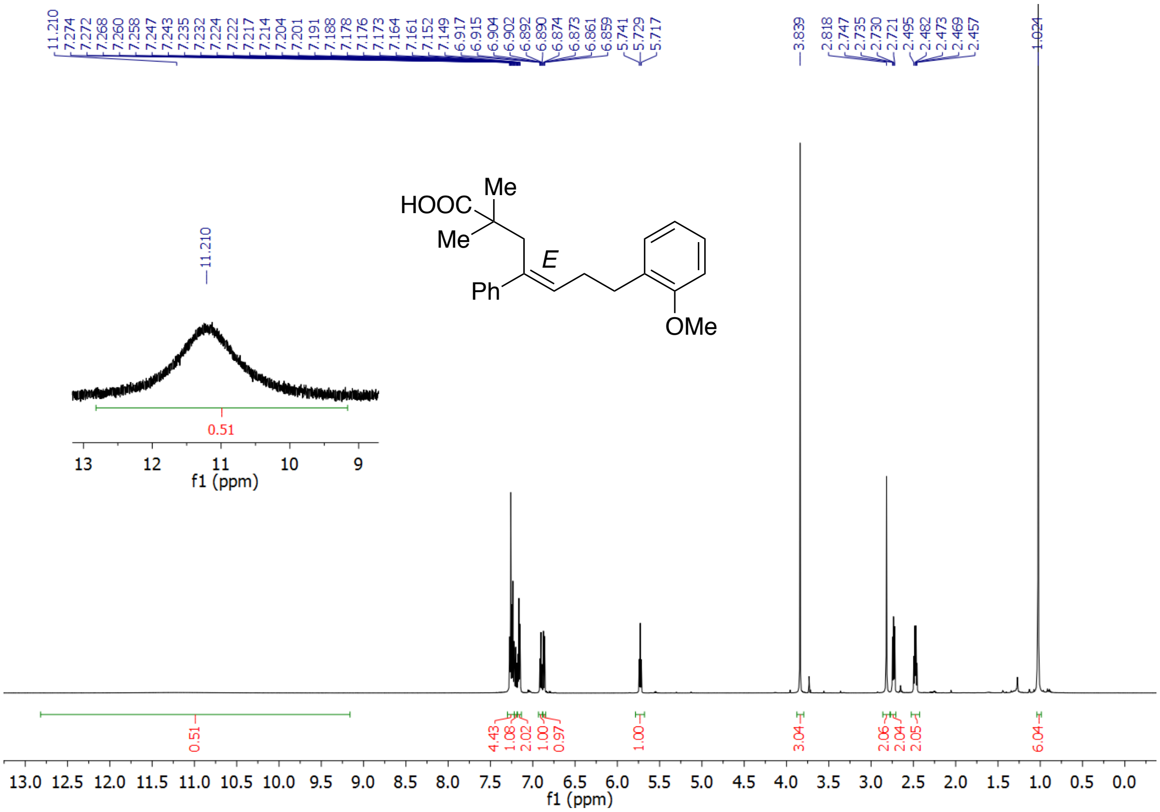

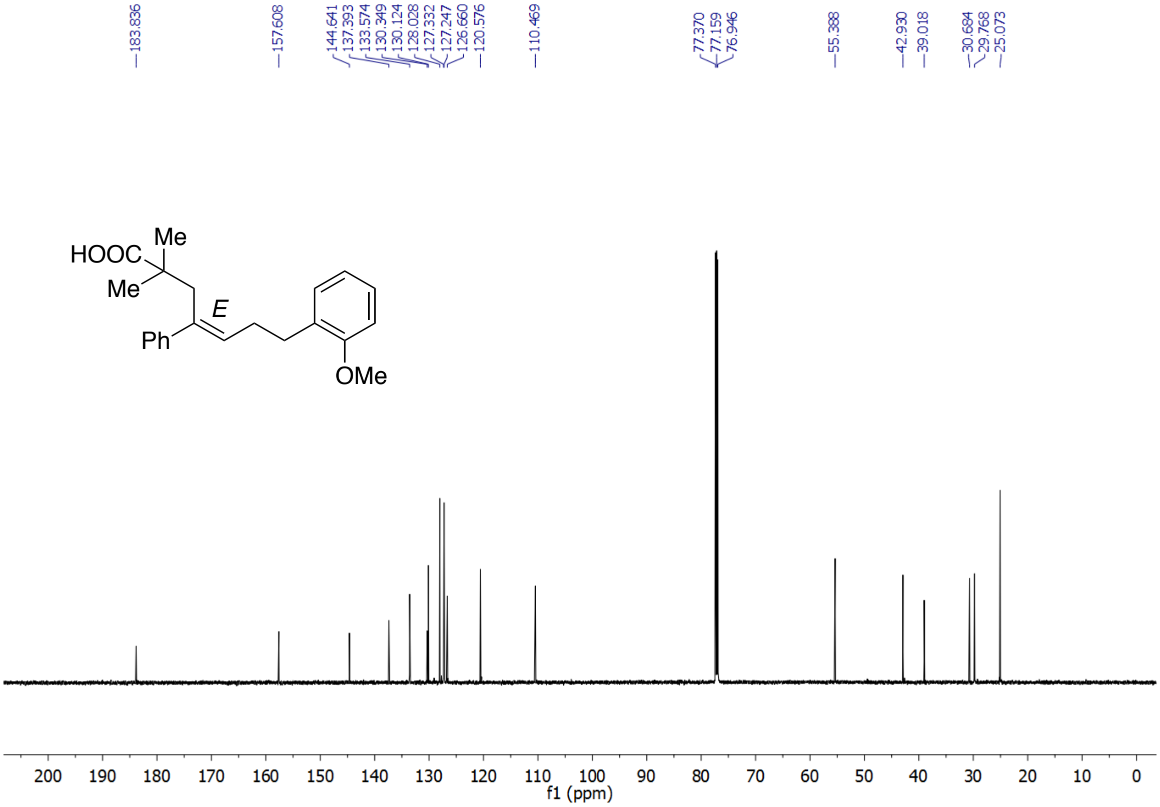

Compound 11. Top: ^{1}H NMR ($CDCl_3$, 600 MHz). Bottom: ^{13}C NMR ($CDCl_3$, 150 MHz)

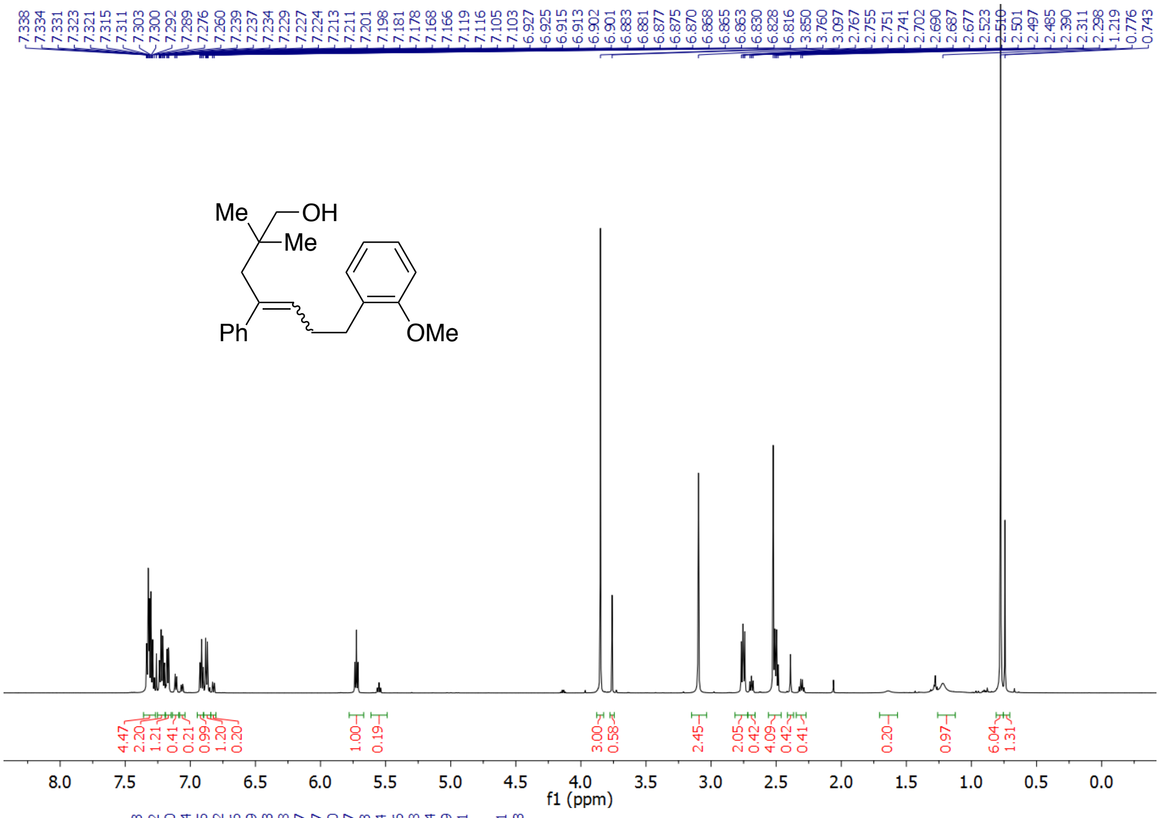

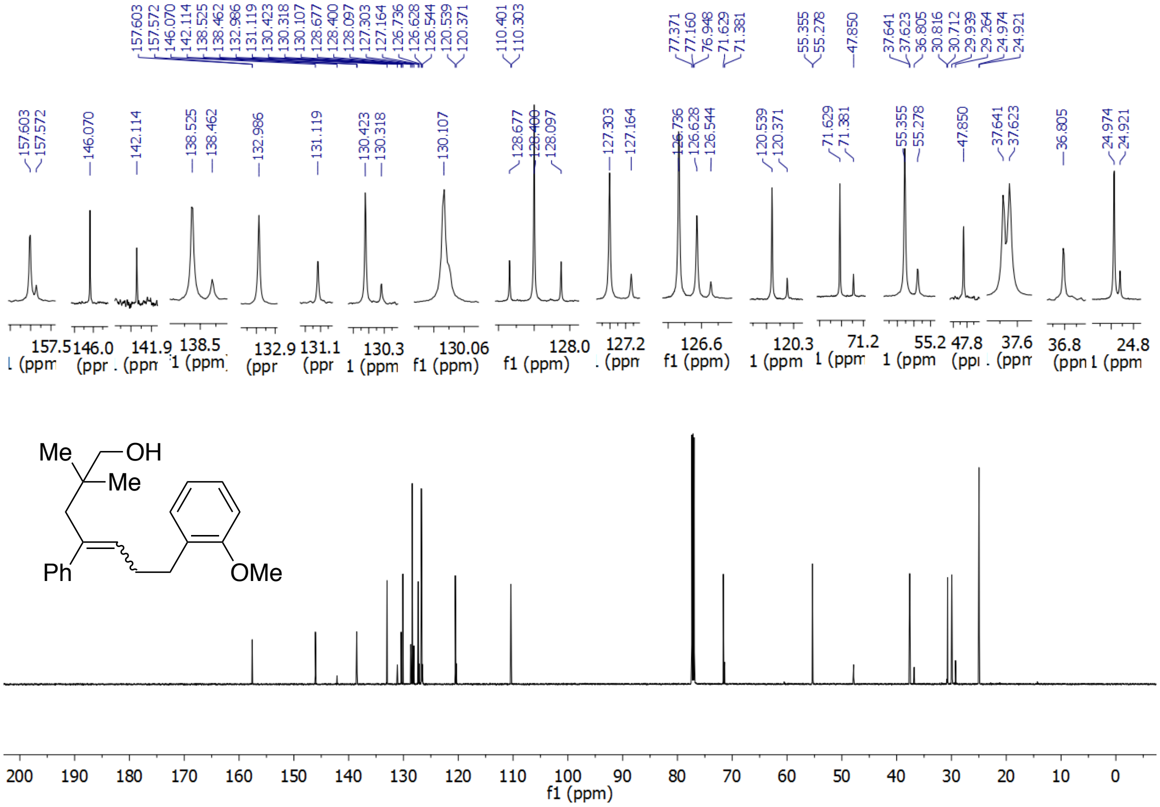

Compound 12 (major). Top: ^{1}H NMR ($CDCl_3$, 600 MHz). Bottom: ^{13}C NMR ($CDCl_3$, 150 MHz)

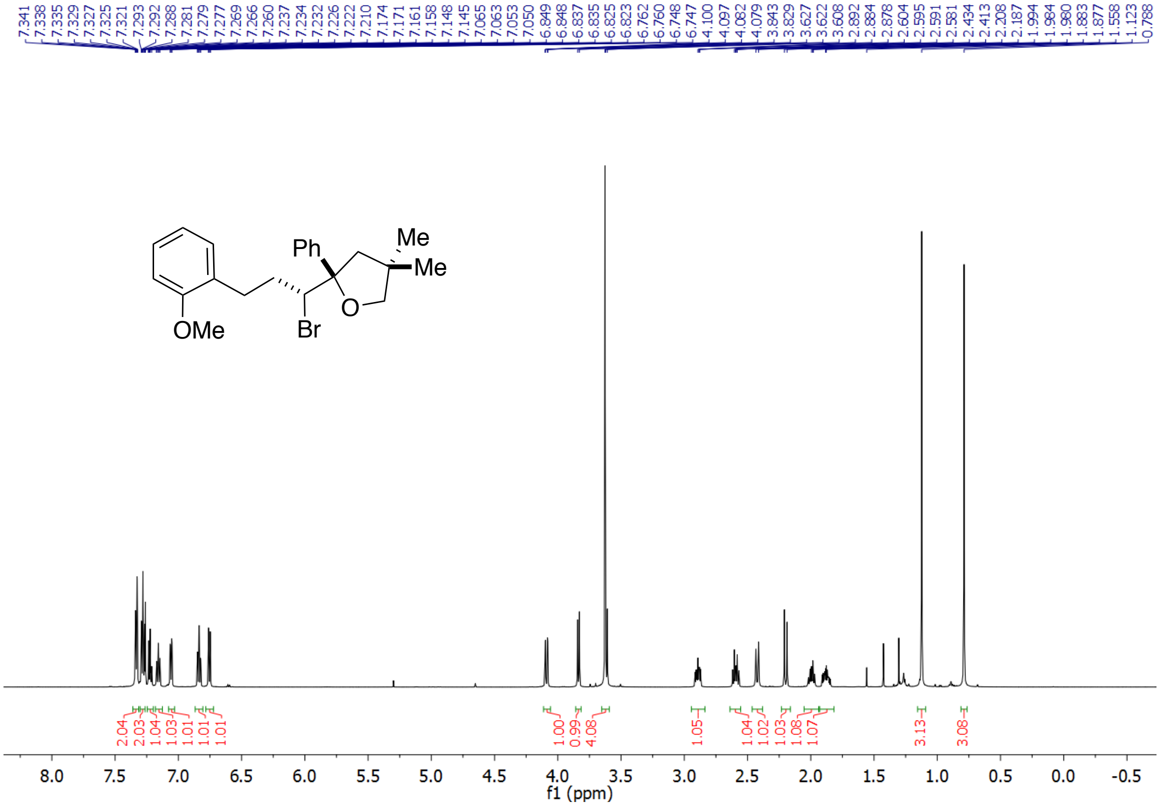

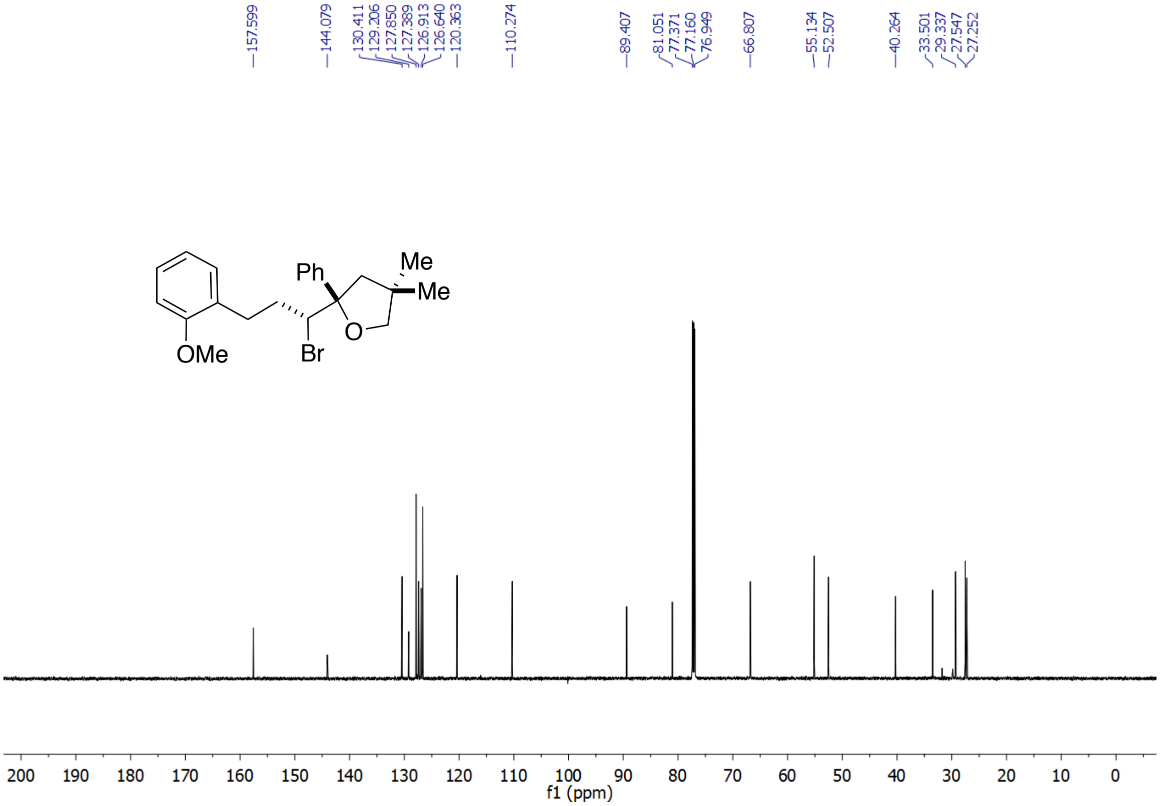

Compound 13. Top: ^{1}H NMR ($CDCl_3$, 600 MHz). Bottom: ^{13}C NMR ($CDCl_3$, 150 MHz)

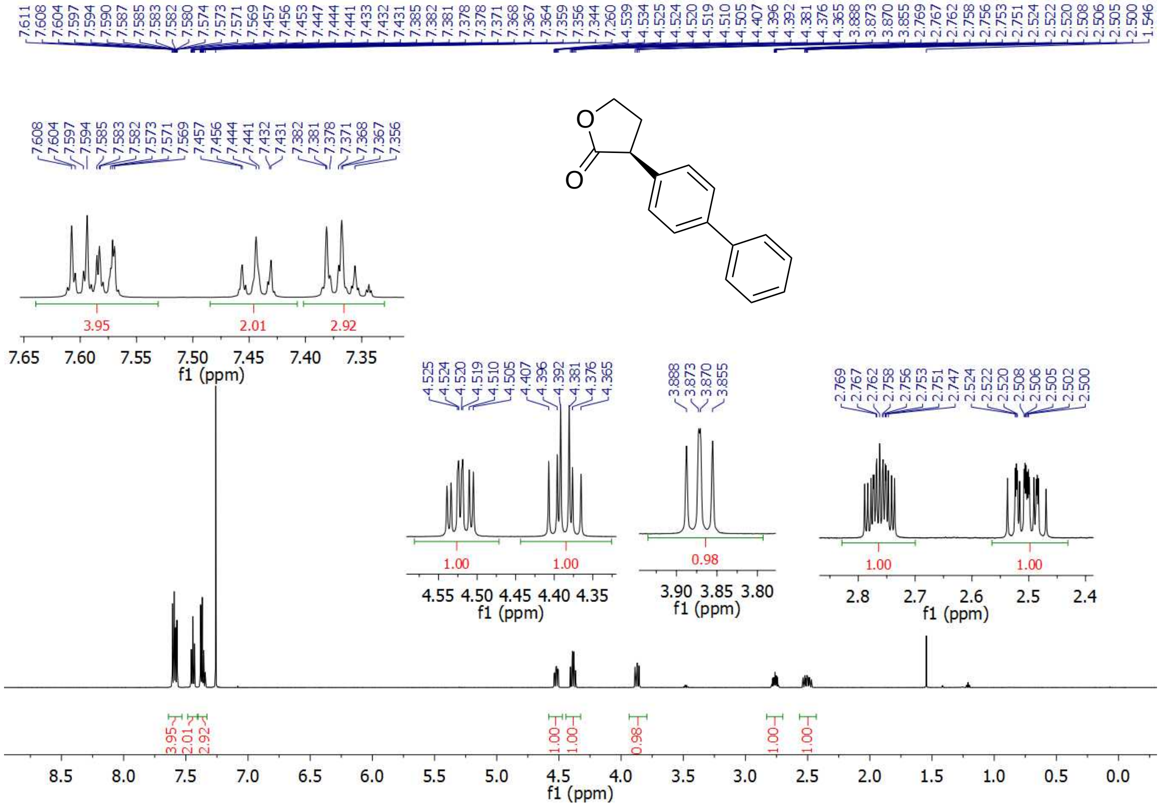

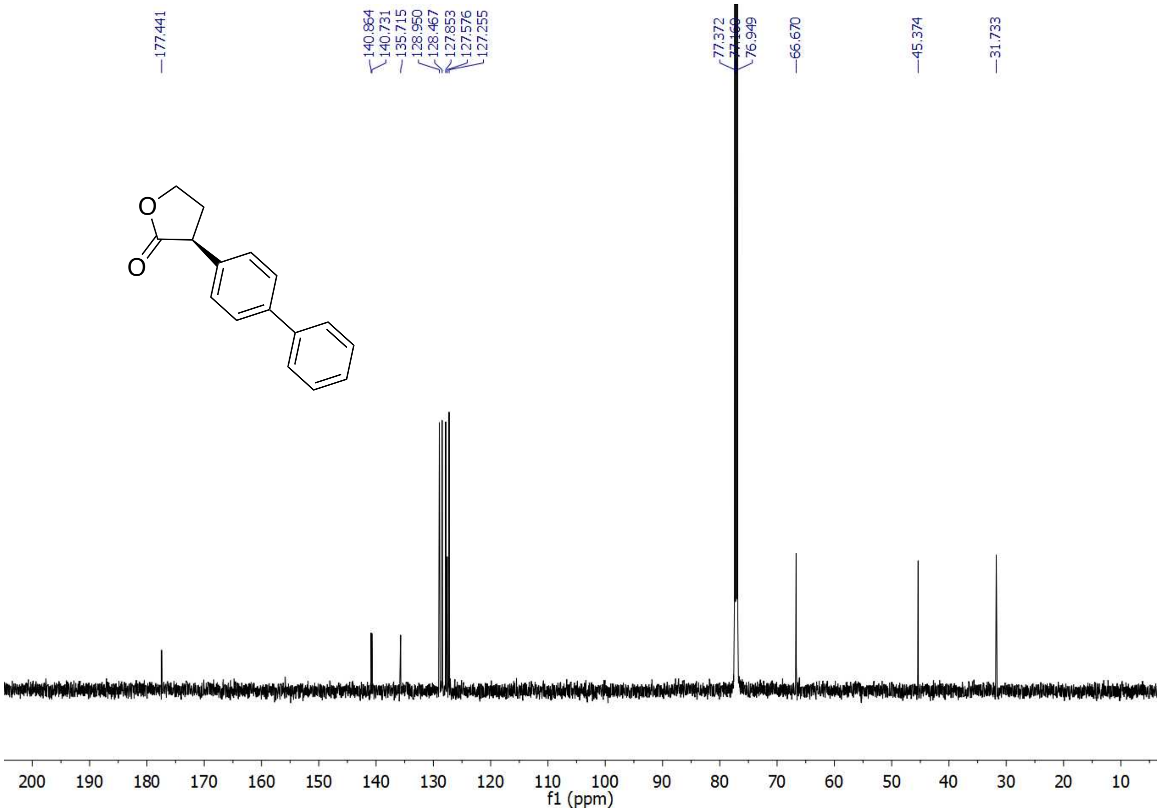

13. HPLC Chromatography of the Products

HPLC data for racemic (top) and optically enriched (bottom) 3a

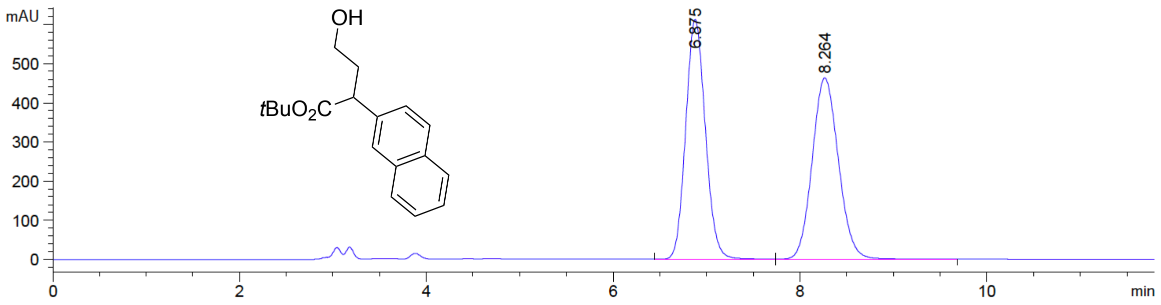

Signal 2: VWD1 B, Wavelength=214 nm

Peak #	RetTime [min]	Type	Width [min]	Area [mAU*s]	Height [mAU]	Area %
1	6.875	BB	0.2265	8944.05957	612.08398	49.8336
2	8.264	BB	0.3030	9003.78613	462.85126	50.1664

Totals : 1.79478e4 1074.93524

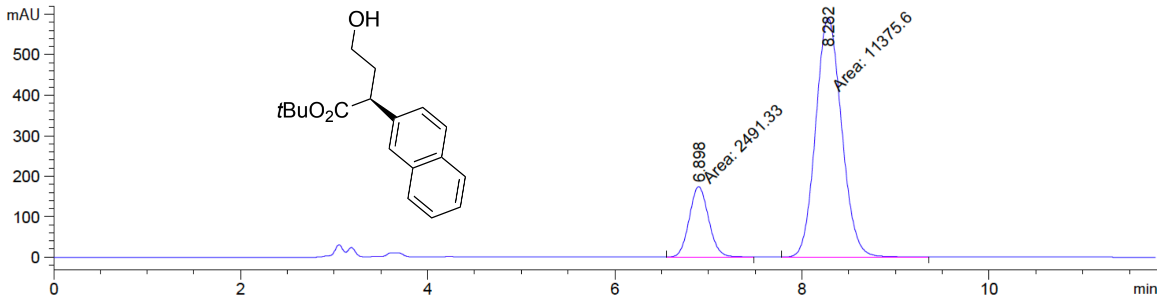

Signal 2: VWD1 B, Wavelength=214 nm

Peak #	RetTime [min]	Type	Width [min]	Area [mAU*s]	Height [mAU]	Area %
1	6.898	MM	0.2392	2491.32617	173.57509	17.9659
2	8.282	MM	0.3212	1.13756e4	590.32355	82.0341

Totals : 1.38669e4 763.89864

HPLC data for racemic (top) and optically enriched (bottom) 3b

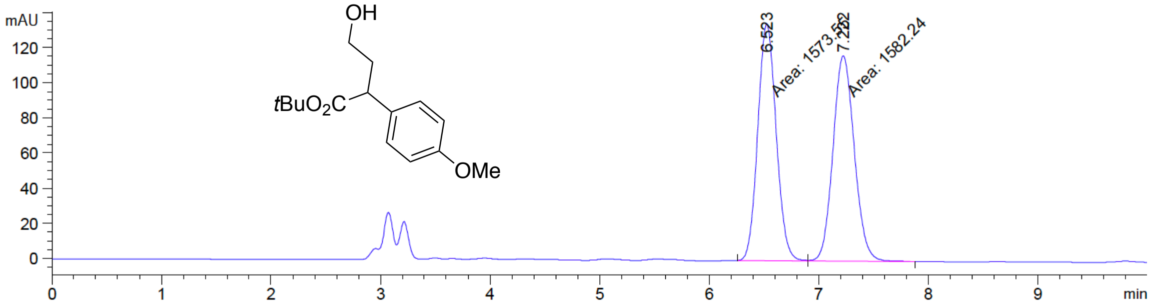

Signal 2: VWD1 B, Wavelength=214 nm

Peak #	RetTime [min]	Type	Width [min]	Area [mAU*s]	Height [mAU]	Area %
1	6.523	MV	0.1944	1573.54895	134.92484	49.8623
2	7.222	VM	0.2253	1582.24170	117.05945	50.1377
Totals :				3155.79065	251.98428	

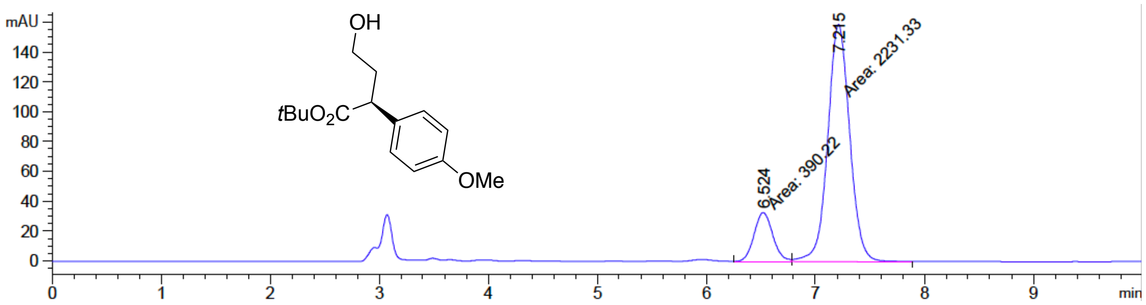

Signal 2: VWD1 B, Wavelength=214 nm

Peak #	RetTime [min]	Type	Width [min]	Area [mAU*s]	Height [mAU]	Area %
1	6.524	MV	0.1984	390.21957	32.78147	14.8851
2	7.215	VM	0.2339	2231.33350	159.02190	85.1149
Totals :				2621.55307	191.80337	

HPLC data for racemic (top) and optically enriched (bottom) 3c

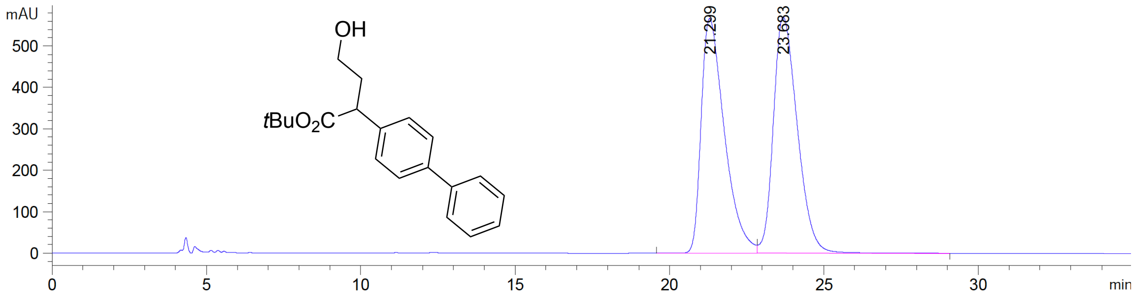

Signal 2: VWD1 B, Wavelength=214 nm

Peak #	RetTime [min]	Type	Width [min]	Area [mAU*s]	Height [mAU]	Area %
1	21.299	BV	0.7945	2.96731e4	567.06781	49.0353
2	23.683	VB	0.8365	3.08406e4	570.70038	50.9647

Totals : 6.05137e4 1137.76819

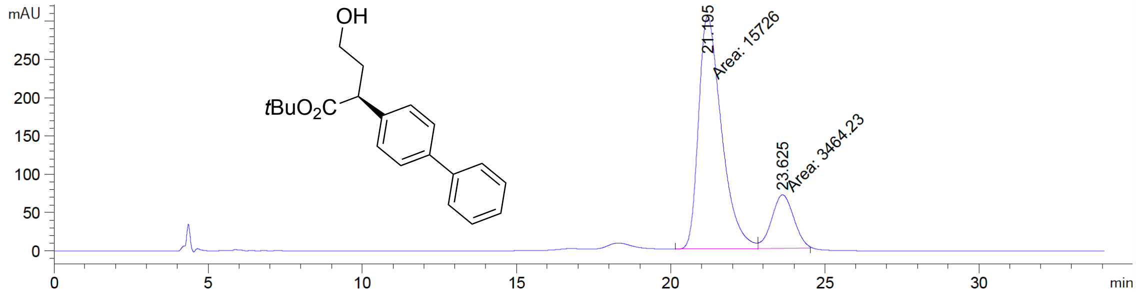

Signal 2: VWD1 B, Wavelength=214 nm

Peak #	RetTime [min]	Type	Width [min]	Area [mAU*s]	Height [mAU]	Area %
1	21.195	MF	0.8652	1.57260e4	302.94287	81.9479
2	23.625	FM	0.8288	3464.23486	69.66569	18.0521

Totals : 1.91902e4 372.60856

HPLC data for racemic (top) and optically enriched (bottom) 3d

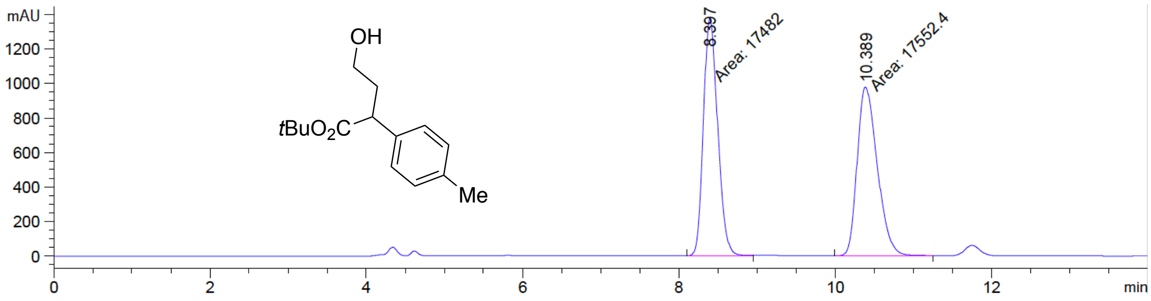

Signal 2: VWD1 B, Wavelength=214 nm

Peak #	RetTime [min]	Type	Width [min]	Area [mAU*s]	Height [mAU]	Area %
1	8.397	MM	0.2119	1.74820e4	1374.92139	49.8996
2	10.389	MM	0.2990	1.75524e4	978.42133	50.1004

Totals : 3.50345e4 2353.34271

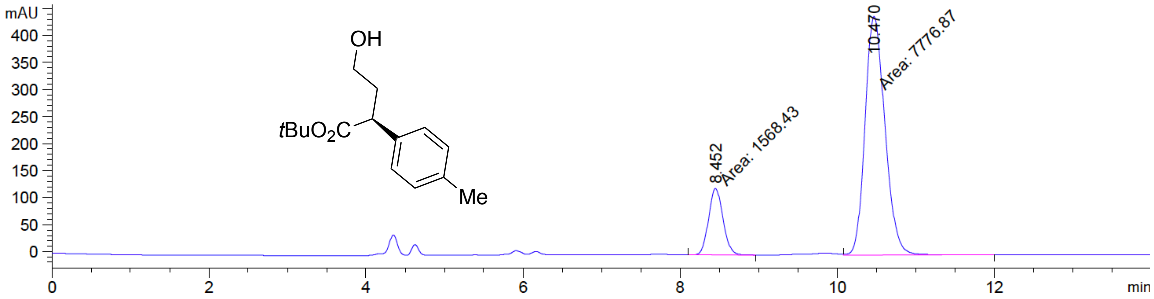

Signal 2: VWD1 B, Wavelength=214 nm

Peak #	RetTime [min]	Type	Width [min]	Area [mAU*s]	Height [mAU]	Area %
1	8.452	MM	0.2126	1568.42603	122.94728	16.7831
2	10.470	FM	0.2937	7776.86719	441.38831	83.2169

Totals : 9345.29321 564.33559

HPLC data for racemic (top) and optically enriched (bottom) 3e

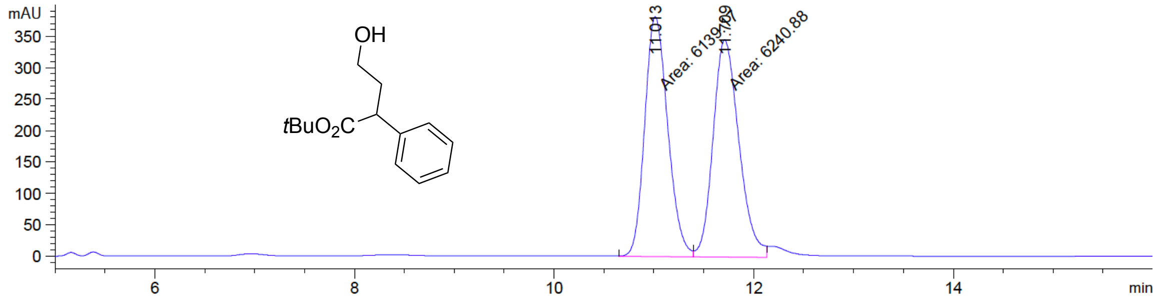

Signal 2: VWD1 B, Wavelength=214 nm

Peak #	RetTime [min]	Type	Width [min]	Area [mAU*s]	Height [mAU]	Area %
1	11.013	MF	0.2682	6139.16748	381.52042	49.5892
2	11.709	FM	0.3012	6240.88330	345.35803	50.4108

Totals : 1.23801e4 726.87845

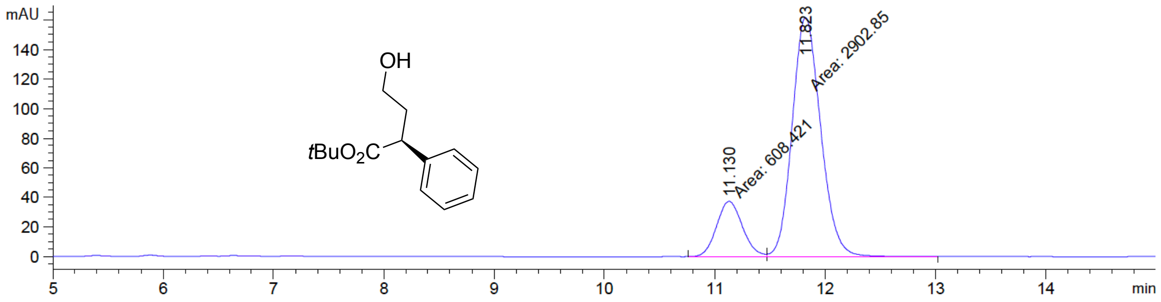

Signal 2: VWD1 B, Wavelength=214 nm

Peak #	RetTime [min]	Type	Width [min]	Area [mAU*s]	Height [mAU]	Area %
1	11.130	MF	0.2713	608.42053	37.37351	17.3276
2	11.823	FM	0.2991	2902.85132	161.75833	82.6724

Totals : 3511.27185 199.13184

HPLC data for racemic (top) and optically enriched (bottom) 3f

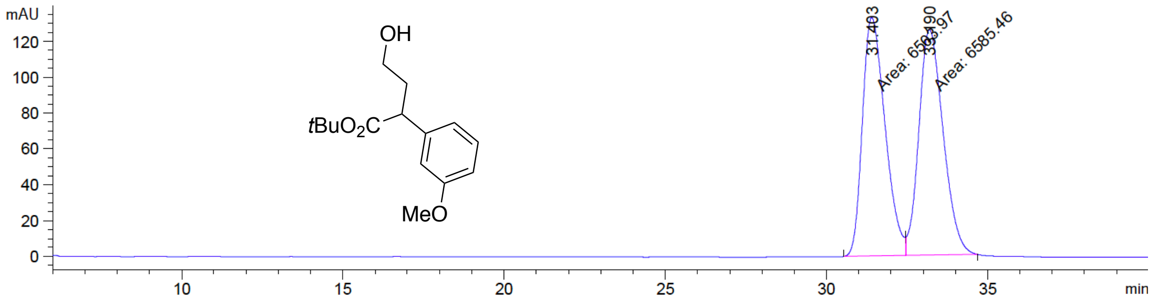

Signal 2: VWD1 B, Wavelength=214 nm

Peak #	RetTime [min]	Type	Width [min]	Area [mAU*s]	Height [mAU]	Area %
1	31.403	MF	0.8152	6503.97363	132.96921	49.6887
2	33.190	FM	0.8723	6585.46338	125.82796	50.3113

Totals : 1.30894e4 258.79717

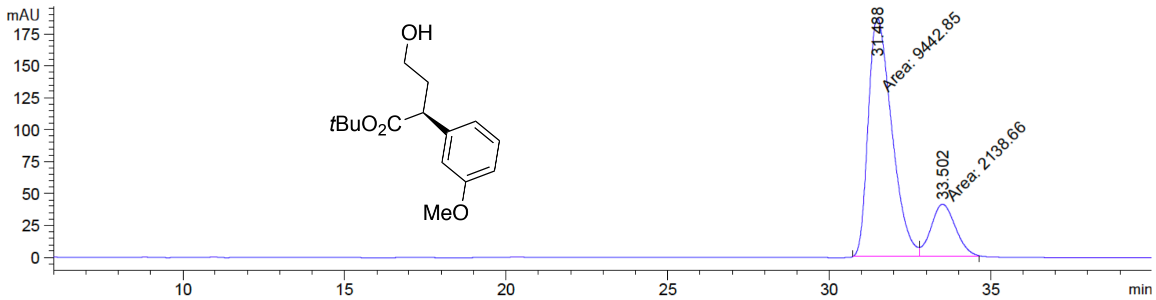

Signal 2: VWD1 B, Wavelength=214 nm

Peak #	RetTime [min]	Type	Width [min]	Area [mAU*s]	Height [mAU]	Area %
1	31.488	MF	0.8453	9442.85449	186.18658	81.5339
2	33.502	FM	0.8756	2138.65723	40.71030	18.4661

Totals : 1.15815e4 226.89688

HPLC data for racemic (top) and optically enriched (bottom) 3g

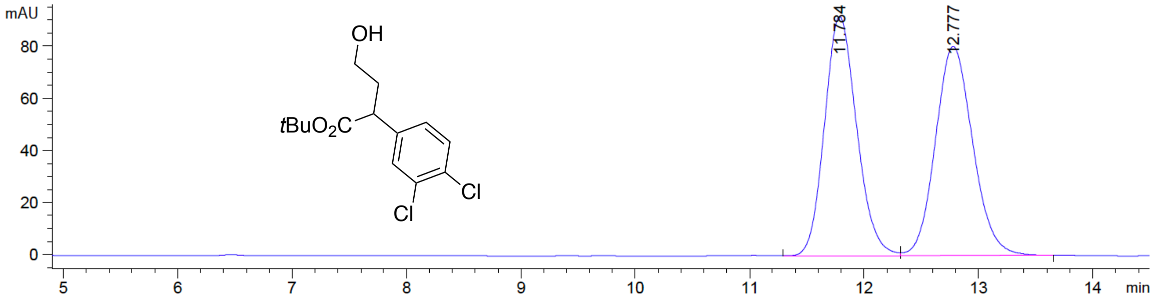

Signal 2: VWD1 B, Wavelength=214 nm

Peak #	RetTime [min]	Type	Width [min]	Area [mAU*s]	Height [mAU]	Area %
1	11.784	BV	0.3020	1792.55176	91.71566	49.6508
2	12.777	VB	0.3506	1817.76282	80.13982	50.3492

Totals : 3610.31458 171.85548

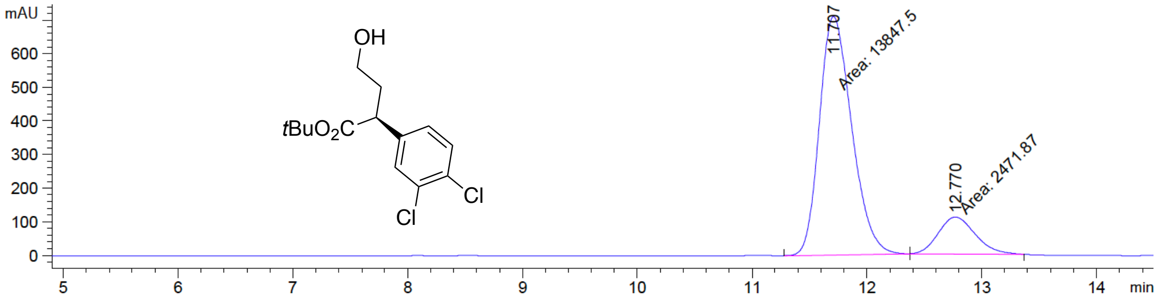

Signal 2: VWD1 B, Wavelength=214 nm

Peak #	RetTime [min]	Type	Width [min]	Area [mAU*s]	Height [mAU]	Area %
1	11.707	MM	0.3251	1.38475e4	709.83801	84.8531
2	12.770	MM	0.3745	2471.87354	110.01044	15.1469

Totals : 1.63194e4 819.84845

HPLC data for racemic (top) and optically enriched (bottom) 3h

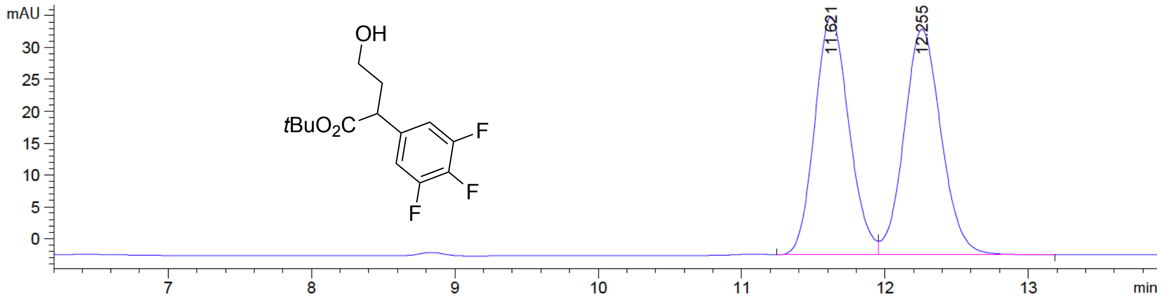

Signal 2: VWD1 B, Wavelength=214 nm

Peak #	RetTime [min]	Type	Width [min]	Area [mAU*s]	Height [mAU]	Area %
1	11.621	BV	0.2572	619.29919	37.39906	49.3568
2	12.255	VB	0.2773	635.43988	35.42442	50.6432

Totals : 1254.73907 72.82348

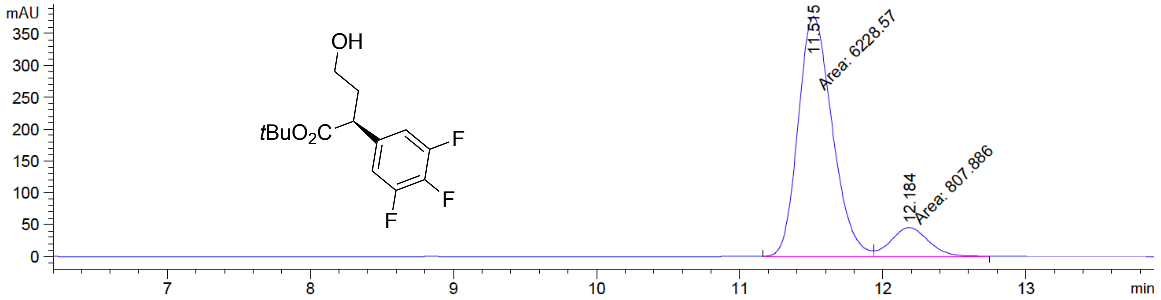

Signal 2: VWD1 B, Wavelength=214 nm

Peak #	RetTime [min]	Type	Width [min]	Area [mAU*s]	Height [mAU]	Area %
1	11.515	MF	0.2755	6228.57373	376.85663	88.5186
2	12.184	FM	0.2997	807.88647	44.92398	11.4814

Totals : 7036.46021 421.78061

HPLC data for racemic (top) and optically enriched (bottom) 3i

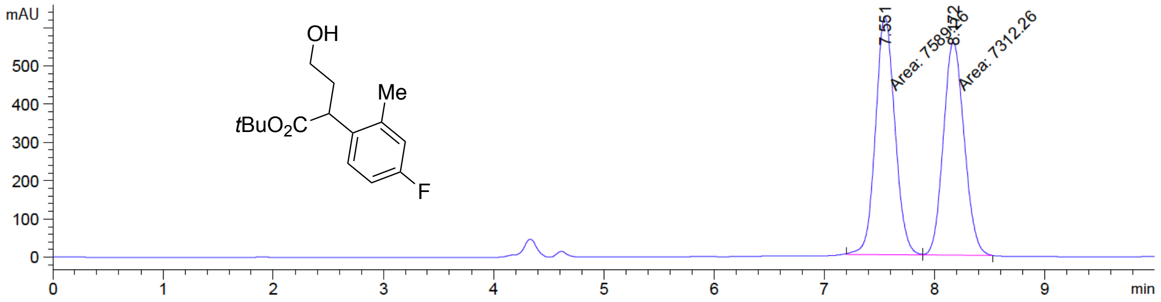

Signal 2: VWD1 B, Wavelength=214 nm

Peak #	RetTime [min]	Type	Width [min]	Area [mAU*s]	Height [mAU]	Area %
1	7.551	MF	0.2033	7589.26416	622.05206	50.9294
2	8.172	FM	0.2187	7312.26172	557.29456	49.0706
Totals :				1.49015e4	1179.34662	

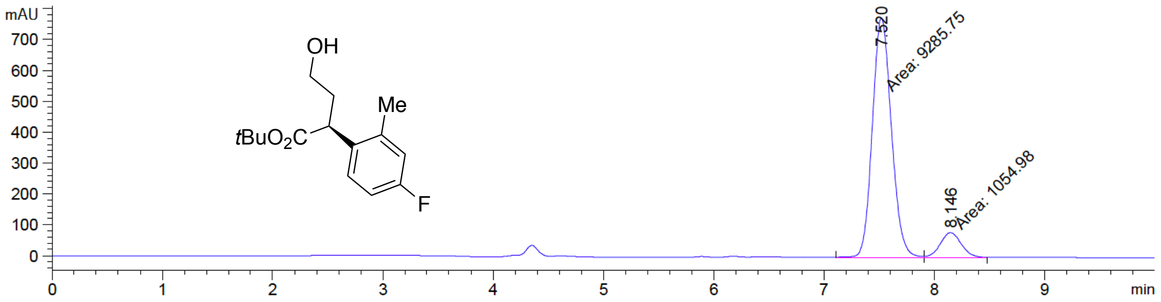

Signal 2: VWD1 B, Wavelength=214 nm

Peak #	RetTime [min]	Type	Width [min]	Area [mAU*s]	Height [mAU]	Area %
1	7.520	MF	0.1995	9285.75000	775.61798	89.7978
2	8.146	FM	0.2194	1054.97681	80.13116	10.2022
Totals :				1.03407e4	855.74915	

HPLC data for racemic (top) and optically enriched (bottom) 3j

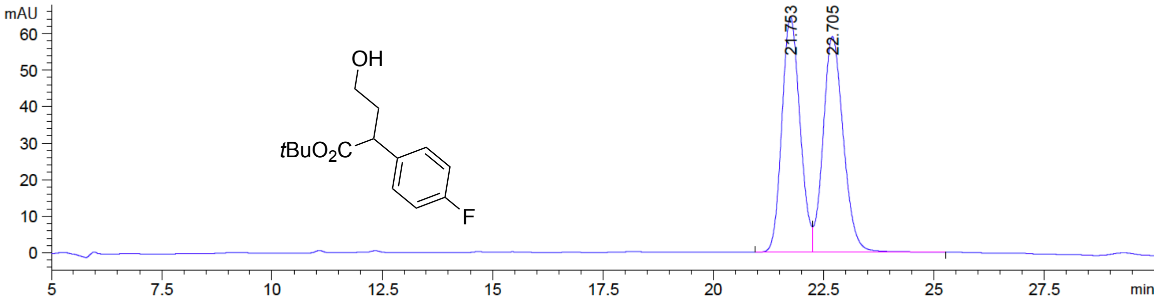

Signal 2: VWD1 B, Wavelength=214 nm

Peak #	RetTime [min]	Type	Width [min]	Area [mAU*s]	Height [mAU]	Area %
1	21.753	BV	0.4465	1837.04297	64.44881	49.3391
2	22.705	VB	0.4926	1886.25928	59.05387	50.6609

Totals : 3723.30225 123.50269

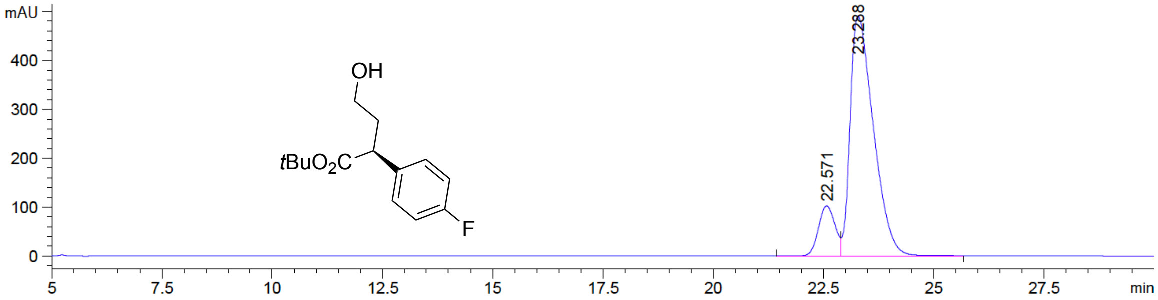

Signal 2: VWD1 B, Wavelength=214 nm

Peak #	RetTime [min]	Type	Width [min]	Area [mAU*s]	Height [mAU]	Area %
1	22.571	BV	0.4114	2663.27319	101.72823	12.9276
2	23.288	VB	0.5614	1.79382e4	489.60522	87.0724

Totals : 2.06014e4 591.33346

HPLC data for racemic (top) and optically enriched (bottom) 3k

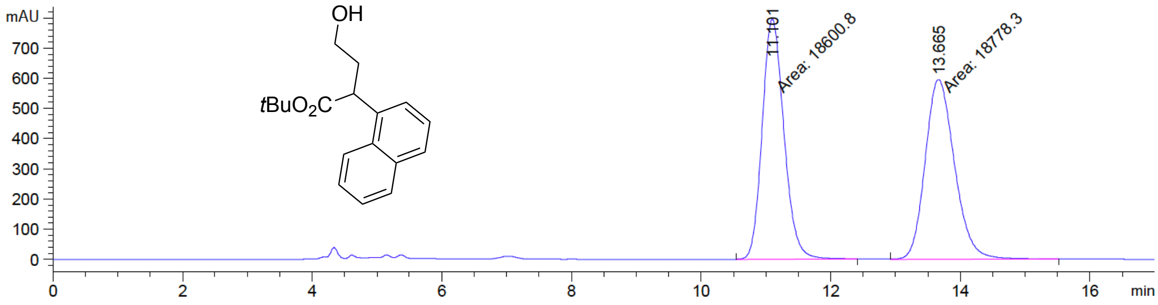

Signal 2: VWD1 B, Wavelength=214 nm

Peak #	RetTime [min]	Type	Width [min]	Area [mAU*s]	Height [mAU]	Area %
1	11.101	MM	0.3894	1.86008e4	796.15094	49.7626
2	13.665	MM	0.5245	1.87783e4	596.72876	50.2374

Totals : 3.73791e4 1392.87970

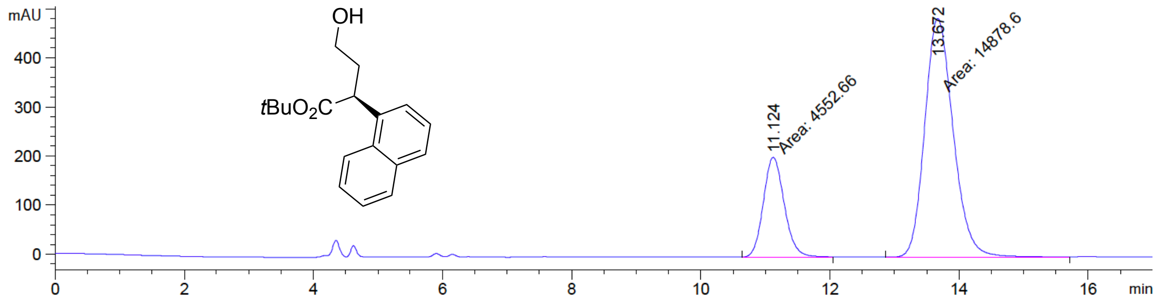

Signal 2: VWD1 B, Wavelength=214 nm

Peak #	RetTime [min]	Type	Width [min]	Area [mAU*s]	Height [mAU]	Area %
1	11.124	MM	0.3737	4552.66113	203.05753	23.4296
2	13.672	MM	0.5100	1.48786e4	486.23474	76.5704

Totals : 1.94312e4 689.29227

HPLC data for racemic (top) and optically enriched (bottom) 2l

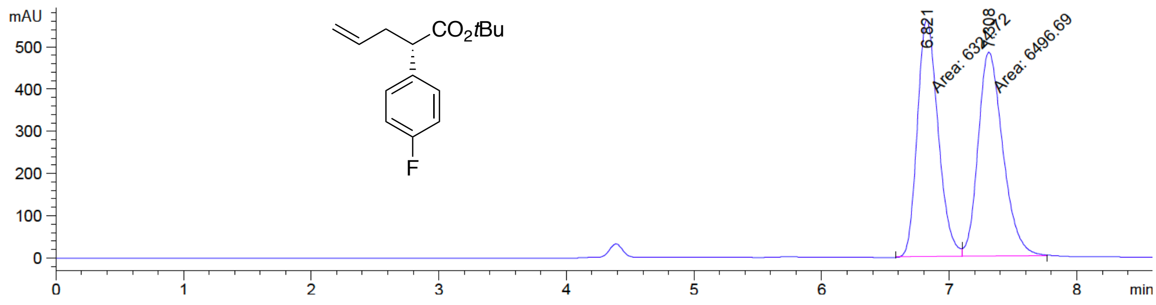

Signal 2: VWD1 B, Wavelength=214 nm

Peak #	RetTime [min]	Type	Width [min]	Area [mAU*s]	Height [mAU]	Area %
1	6.821	MF	0.1877	6324.71631	561.64685	49.3294
2	7.308	FM	0.2241	6496.68506	483.14310	50.6706

Totals : 1.28214e4 1044.78995

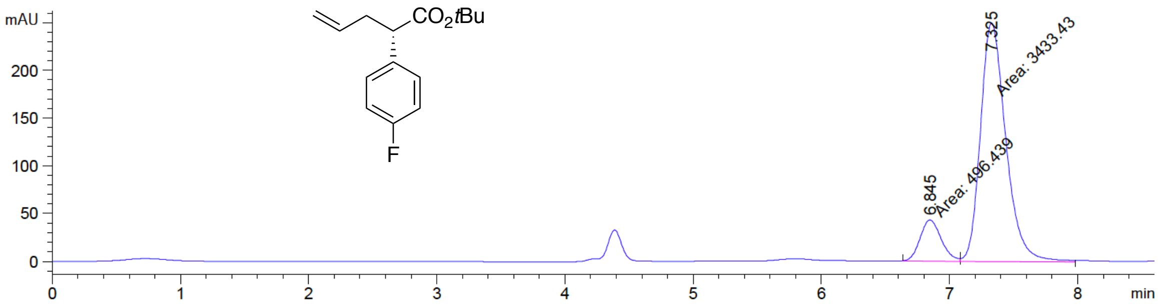

Signal 2: VWD1 B, Wavelength=214 nm

Peak #	RetTime [min]	Type	Width [min]	Area [mAU*s]	Height [mAU]	Area %
1	6.845	MF	0.1913	496.43896	43.25717	12.6325
2	7.325	FM	0.2290	3433.42822	249.85635	87.3675

Totals : 3929.86719 293.11353

HPLC data for racemic (top) and optically enriched (bottom) 9

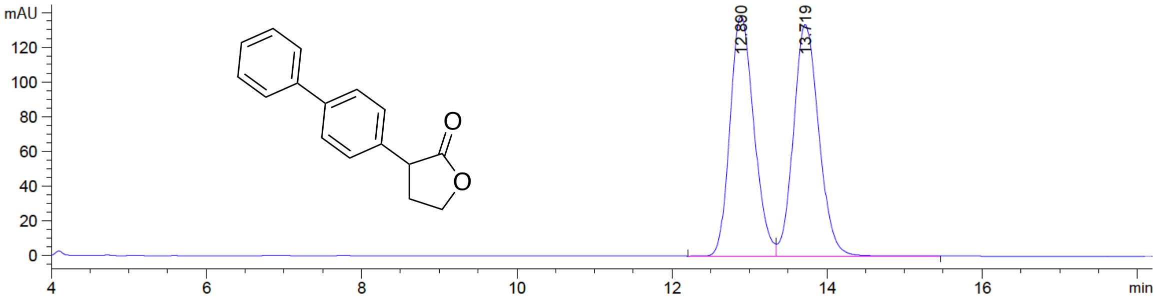

Signal 2: VWD1 B, Wavelength=214 nm

Peak #	RetTime [min]	Type	Width [min]	Area [mAU*s]	Height [mAU]	Area %
1	12.890	BV	0.3320	2956.27881	137.95514	49.4306
2	13.719	VB	0.3525	3024.38159	133.39445	50.5694

Totals : 5980.66040 271.34959

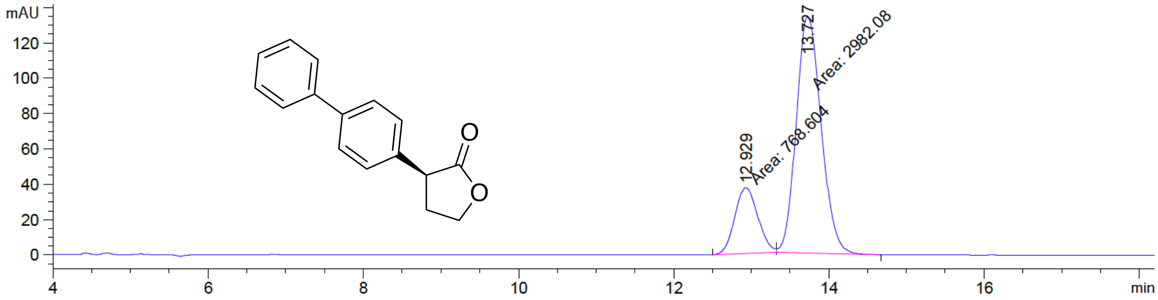

Signal 2: VWD1 B, Wavelength=214 nm

Peak #	RetTime [min]	Type	Width [min]	Area [mAU*s]	Height [mAU]	Area %
1	12.929	MM	0.3439	768.60382	37.24547	20.4924
2	13.727	MM	0.3711	2982.08228	133.91818	79.5076

Totals : 3750.68610 171.16365

14. Computational Methods, Energies, and Coordinates

All optimizations were carried out without constraints at the (U)B3LYP/6-31G(d)[16] level of theory with the "guess=mix" keyword as implemented in Gaussian16 (**Figure S8**)[17] and Gaussian 09 (**Figure S9** and **S10**)[18]. To refine energetics, we carried out single point energy calculations using (U)PBEPBE/6-311+G(d,p)-SDD(Fe)-THF(SMD) [19] in a polarizable continuum solvent (THF) with SMD as solvation model[20] to account for the condensed phase effects. All structural figures were generated using CYLview.[21] Vibrational frequencies were computed at the same level to obtain thermal corrections (at 298 K; enthalpic and free energy) and to characterize the stationary points as transition states (one and only one imaginary frequency) or minima (zero imaginary frequencies). Exhaustive conformational searches were performed for all intermediates to map out the lowest energy profile, and intrinsic reaction coordinate (IRCs) calculations were undertaken for selected transition state structures to ensure they connected the illustrated ground states.

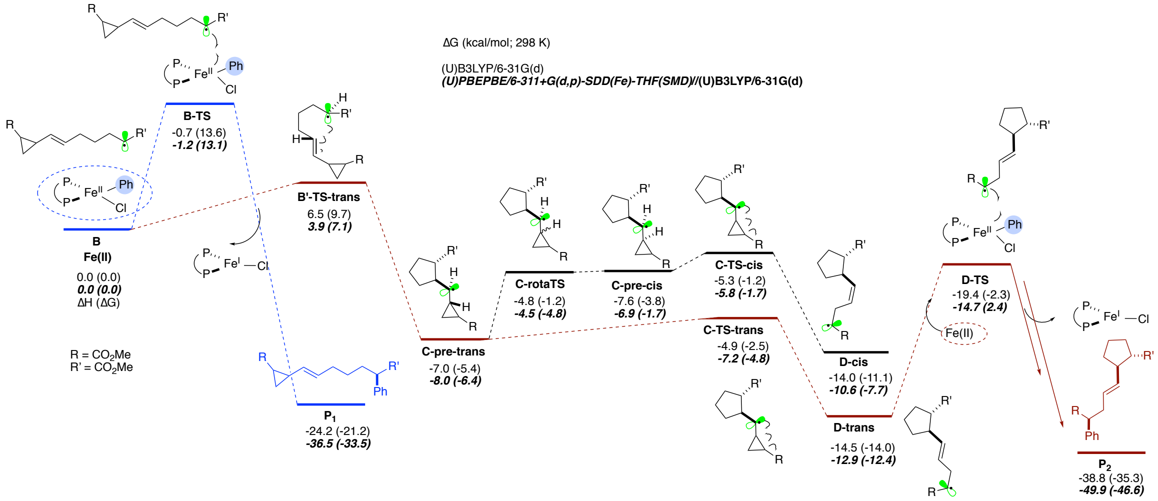

Figure S8. The energy coordinates of the two possible pathways. Blue: radical addition then reductive elimination (not shown) to form the cross-coupling product. Red: Cyclization forming the *trans*-five-member ring and ring opening of the cyclopropane to generate the distal radical for cross-coupling reaction. Black: possible diastereomers (*cis*) by single bond rotation that forms the *Z* alkene.

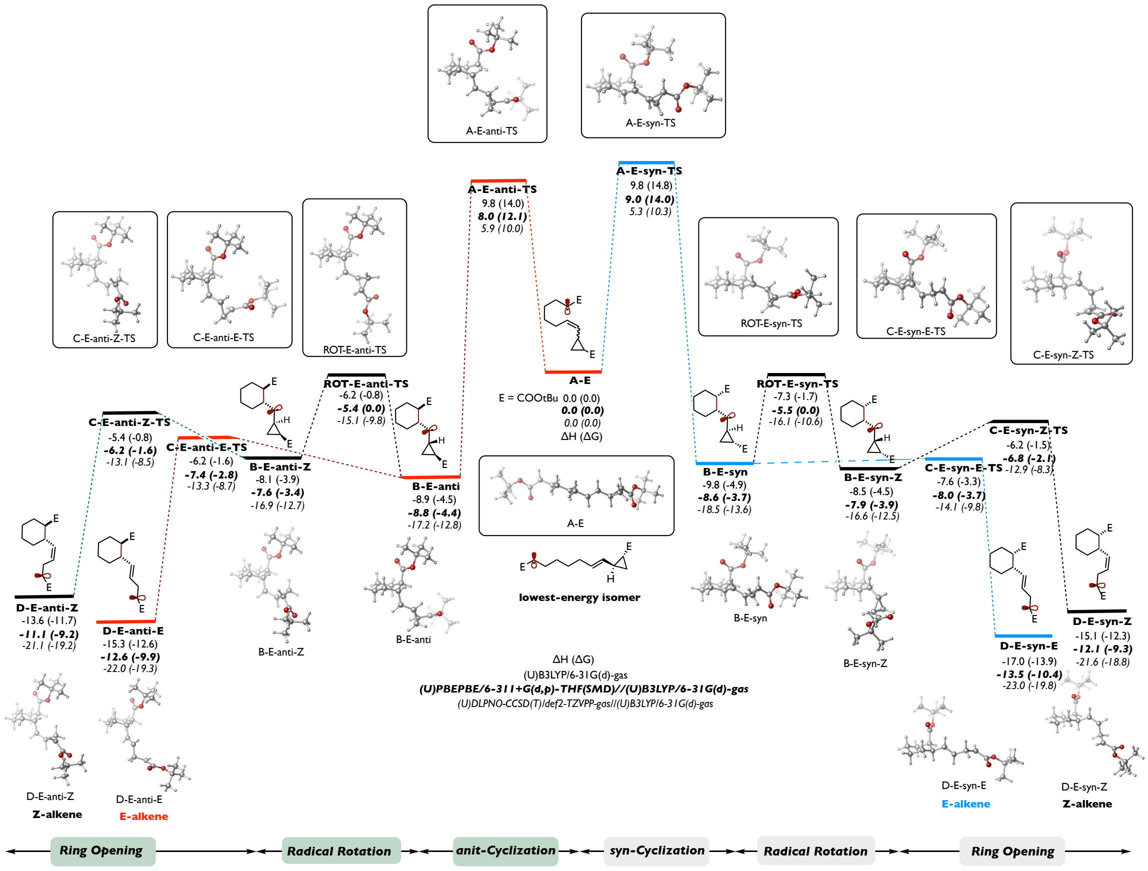

Figure S9. Energetics for the 6-exo-cyclization computed at the UPBEPBE/6-311+G(d,p)-THF(SMD)//UB3LYP/6-31G(d), UB3LYP/6-31G(d) and DLPNO-CCSD(T)/def2-TZVPP//UB3LYP/6-31G(d) levels of theory.

As shown in Figure S9, the barrier of 6-*exo*-cyclization via **A-E-anti-TS** was computed to be 12.1 kcal/mol, which is much higher than 5-*exo*-cyclization (only 7.1 kcal/mol, see **Scheme 2**). Thus, radical species is less favorable to undergo 6-*exo*-cyclization compared to the 5-*exo*-cyclization situation, which is consistent with the experimental result that no 6-*exo*-cyclization product was observed.

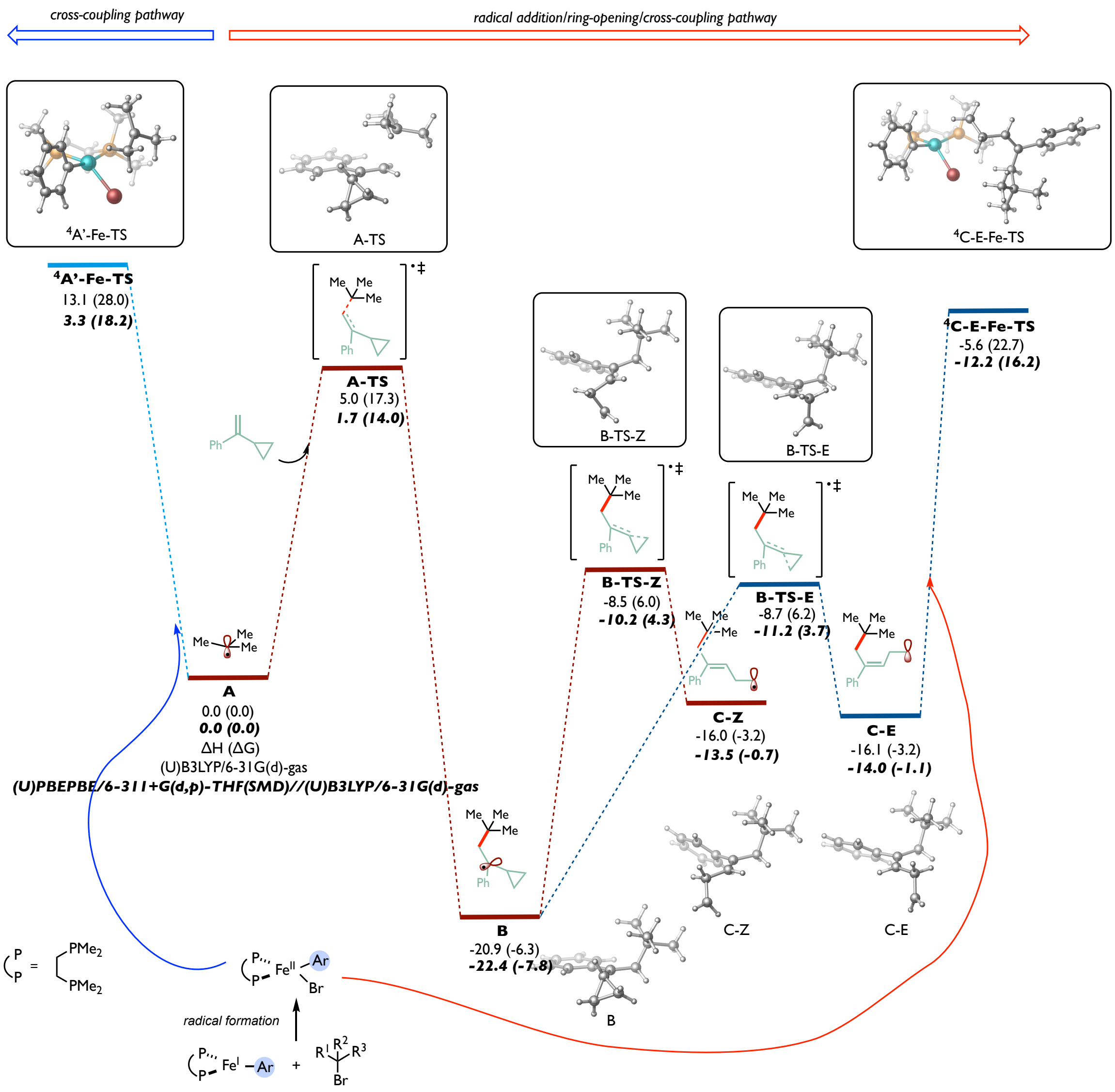

Figure S10. Energetics for the Fe-catalyzed intermolecular dicarbofunctionalization of vinyl cyclopropane computed at the UPBEPBE/6-311+G(d,p)-THF(SMD)//UB3LYP/6-31G(d), and UB3LYP/6-31G(d) levels of theory.

As shown in Figure S10, the barrier for radical addition of *tert*-butyl radical to olefin (via **A-TS**) is 4.2 kcal/mol *lower* in energy than the radical binding transition state to Fe center (**^{4}A'-Fe-TS**), which will lead to the generation of tertiary benzylic radical **B** and cross-coupling product *t*Bu-Ph, respectively. After radical species **B** is generated (kinetically favorable), it will undergo ring-opening to generate either *Z* alkene (via **B-TS-Z**) or *E* alkene (via **B-TS-E**), both of which are endergonic compared to radical

species **B**. Noticeably, the *E* alkene generated (-1.1 kcal/mol with respect to **A**) is in slightly lower energy level than the *Z* alkene (-0.7 kcal/mol with respect to **A**), which might explain the observed *E*/*Z* selectivity in experiment. After ring-opening, the primary radical **C-E** can undergo radical addition to Fe catalyst and generate the cross-coupled product eventually. Overall, these results show that similar to the Fe-catalyzed out-of-cage arylation as shown in Scheme 2, the radical addition/ring-opening/cross-coupling pathway (red) is also kinetically favored over the in-cage cross-coupling pathway (blue).

Figure S8.

B

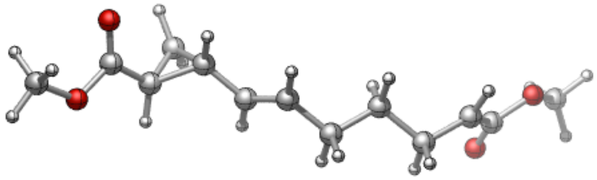

Zero-point correction= 0.302874 (Hartree/Particle)
Thermal correction to Energy= 0.322369
Thermal correction to Enthalpy= 0.323313
Thermal correction to Gibbs Free Energy= 0.249564
Sum of electronic and zero-point Energies= -807.347715
Sum of electronic and thermal Energies= -807.328220
Sum of electronic and thermal Enthalpies= -807.327276
Sum of electronic and thermal Free Energies= -807.401025
HF=-807.650589 (U)B3LYP/6-31G(d)
HF=-806.9126631 (U)PBEPBE/6-311+G(d,p)-THF(SMD)//(U)B3LYP/6-31G(d)

C	0.93600700	4.30182500	2.63355200
C	1.07825700	4.83662800	1.29371100
O	1.97112000	4.54567400	0.50709100
O	0.08573600	5.72202800	0.99662700
H	0.08372600	4.62764700	3.22346300
C	1.88820400	3.29854300	3.17758500
H	2.28303900	3.65383700	4.14291400
H	2.73464600	3.20156200	2.48983100
C	1.23680000	1.91240800	3.40643100
H	0.37117700	2.01869200	4.07474800
H	0.85130200	1.52967100	2.45384800
C	2.22816500	0.89438300	4.00326100
H	2.62948500	1.30774700	4.94182000
H	3.08263300	0.77531600	3.32420200
C	1.59729500	-0.44368800	4.27044400
H	0.78115500	-0.45399700	4.99585900
C	1.94740600	-1.59082200	3.67769100
H	2.75985500	-1.58653900	2.94787400

C	1.30632900	-2.89813000	3.93241600
C	1.21302200	-3.94167700	2.85683500
C	2.17944100	-4.16412700	3.99822500
H	0.48846500	-2.89454600	4.64896600
H	1.64186800	-3.71920300	1.88354200
H	0.32697900	-4.56883300	2.84511300
H	3.24113100	-4.04858000	3.80796500
C	1.81422800	-5.16992200	5.02724300
O	0.67848700	-5.45431400	5.35560700
O	2.91454300	-5.73721400	5.57304700
C	0.15892300	6.29619000	-0.31356100
H	-0.69329600	6.97317700	-0.38619500
H	1.09619700	6.84492400	-0.44540500
H	0.09743900	5.51972600	-1.08190300
C	2.65629700	-6.71864500	6.58961300
H	3.63680900	-7.07016200	6.91245300
H	2.11328000	-6.27192400	7.42689300
H	2.06529800	-7.54510700	6.18581500

Fe(II)-Ph-Cl

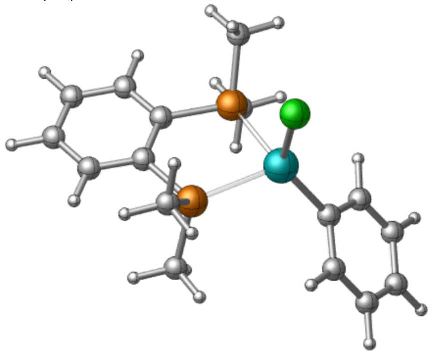

Zero-point correction= 0.326209 (Hartree/Particle)
Thermal correction to Energy= 0.350766
Thermal correction to Enthalpy= 0.351710
Thermal correction to Gibbs Free Energy= 0.267694
Sum of electronic and zero-point Energies= -3028.616000
Sum of electronic and thermal Energies= -3028.591443
Sum of electronic and thermal Enthalpies= -3028.590499
Sum of electronic and thermal Free Energies= -3028.674515
HF=-3028.942209 (U)B3LYP/6-31G(d)
HF=-1888.1441828 (U)PBEPBE/6-311+G(d,p)-SDD(Fe)-THF(SMD)//(U)B3LYP/6-31G(d)

C	-2.13430600	-0.05442200	0.70767000
C	-2.73754400	-0.62796500	1.83839000
C	-2.93339600	0.25586100	-0.41422700
C	-4.30990500	-0.01717400	-0.36932800
C	1.02444300	3.05538800	-1.59625300
C	0.65332100	3.98917700	-2.59003800
C	1.95875300	3.51841300	-0.64273900
C	1.16891200	5.28735400	-2.63342500
H	-0.05724100	3.69955900	-3.36560400
C	2.48443800	4.81305100	-0.67071500
H	2.29875700	2.85019000	0.14971500
C	2.08814700	5.70462700	-1.66917400
H	0.85747000	5.97301500	-3.41932800
H	3.20447500	5.12667200	0.08292900
H	2.49395300	6.71334400	-1.69736300
C	-4.10428500	-0.89290200	1.86897700
H	-4.55197700	-1.33789700	2.75342300
C	-4.89468700	-0.58596700	0.75919000
H	-5.96180300	-0.79028100	0.77389900
H	-2.13394300	-0.87360500	2.70860700
H	-4.93653900	0.21594100	-1.22652400
P	-0.31366000	0.30106400	0.66824600
P	-2.15585200	1.02108100	-1.91491800
Fe	0.28599800	1.16612000	-1.56864500

Cl	0.99970700	-0.73223600	-2.59087100
C	0.46453200	-1.29362900	1.18181000
H	1.53435000	-1.11780000	1.33429500
H	0.03363300	-1.70087200	2.10242100
H	0.35823800	-2.01887500	0.37077400
C	-0.06163800	1.37099700	2.15744600
H	-0.45714400	0.91641400	3.07200100
H	1.01169700	1.54523000	2.28362500
H	-0.54444300	2.34003000	2.00195600
C	-2.74634900	-0.02697900	-3.31630900
H	-3.83244600	-0.16657000	-3.31943000
H	-2.44606100	0.45631300	-4.25174400
H	-2.24855300	-0.99909100	-3.26901700
C	-3.12633300	2.58085900	-2.13835600
H	-2.81080200	3.05797800	-3.07170300
H	-4.20586200	2.40031500	-2.17769800
H	-2.90898700	3.27039600	-1.31762600

B-TS

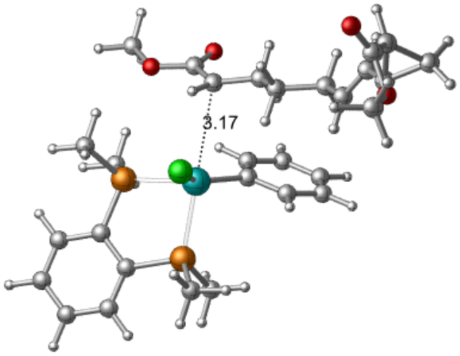

Imaginary frequency=-64.04
Zero-point correction= 0.629707 (Hartree/Particle)
Thermal correction to Energy= 0.674951
Thermal correction to Enthalpy= 0.675895
Thermal correction to Gibbs Free Energy= 0.541033
Sum of electronic and zero-point Energies= -3835.965145
Sum of electronic and thermal Energies= -3835.919901
Sum of electronic and thermal Enthalpies= -3835.918957
Sum of electronic and thermal Free Energies= -3836.053818
HF=-3836.5948517 (U)B3LYP/6-31G(d)
HF=-2695.059687 (U)PBEPBE/6-311+G(d,p)-SDD(Fe)-THF(SMD)//(U)B3LYP/6-31G(d)

C	-3.35374800	-0.19844100	-0.62622800
C	-4.55793300	-0.62584500	-1.20765300
C	-3.31872000	1.03986300	0.05169600
C	-4.49038300	1.80958300	0.12795600
C	-5.71271400	0.14788900	-1.12316500
H	-6.63555500	-0.20125700	-1.57834100
C	-5.67875800	1.37159300	-0.45115200
H	-6.57522800	1.98137600	-0.37873100
H	-4.59670600	-1.57588400	-1.73462300
H	-4.47876500	2.76444600	0.64743000
P	-1.81195200	-1.21963800	-0.73937200
P	-1.73604400	1.63792600	0.81290600
Fe	-0.05171200	-0.15391100	0.64174400
C	-2.35313300	-2.92928200	-0.30136900
C	-1.45988300	3.25424800	-0.04355600
C	2.18621400	1.14085300	2.47275100
Cl	-0.55265300	-1.28759200	2.57736000
C	1.50402000	-0.09352500	-0.65919200
C	2.12100100	-1.30194600	-1.05572300
C	1.99316100	1.07939800	-1.27160200
C	3.15607800	-1.34089200	-1.99467300
H	1.79432600	-2.24678300	-0.61843500
C	3.02446400	1.05564400	-2.21624000
H	1.57929400	2.04988500	-0.99829300

C	3.61043800	-0.15729700	-2.58099000
H	3.60791600	-2.29219500	-2.26954100
H	3.37734200	1.98482200	-2.65893800
H	4.41612400	-0.18027200	-3.31100100
C	-2.20357200	2.14151600	2.52371200
H	-1.31504300	2.58233800	2.98523800
H	-2.47582200	1.24845000	3.09258100
H	-3.02880300	2.86103000	2.54835700
C	-1.51683100	-1.33296200	-2.56140800
H	-0.63337300	-1.95568600	-2.73290900
H	-1.30234300	-0.33702800	-2.95927000
H	-2.37263800	-1.75651100	-3.09808300
C	2.20495000	2.55942300	2.17181700
O	3.11331300	3.15458700	1.61073500
O	1.08053400	3.21714100	2.62301500
C	1.14428700	4.64360200	2.50332200
H	1.26372500	4.94911600	1.45958000
H	0.20229700	5.01820900	2.90761200
H	1.98706700	5.04104700	3.07665600
H	1.38104600	0.75099200	3.08794100
C	3.41769200	0.33249200	2.22182900
H	4.28590300	0.89681100	2.59973800
H	3.58779200	0.26144600	1.13792900
C	3.38387200	-1.06849600	2.84411300
H	3.29362800	-0.98949200	3.93555100
H	2.48765300	-1.60121800	2.50249900
C	4.63578200	-1.89363200	2.49270000
H	5.53979300	-1.36002300	2.81717900
H	4.69656500	-1.97281300	1.39599500
C	4.61341000	-3.26926500	3.09860300
H	3.75611900	-3.89274600	2.83565500
C	5.53736400	-3.76299700	3.93013600
H	6.39629700	-3.14532800	4.20155200
C	5.49473200	-5.11690100	4.52523700
C	6.75551600	-5.88151100	4.81454000
C	5.97084400	-5.31077000	5.97674100
H	4.63295300	-5.72001300	4.25175400
H	7.70701400	-5.40728100	4.58851400
H	6.73652800	-6.95847400	4.68356100
H	6.37280500	-4.43634700	6.47764600
C	5.20880200	-6.18355600	6.90457500
O	5.01999000	-5.94169100	8.07897100
O	4.72985100	-7.29820000	6.29136700
C	3.96406100	-8.17393900	7.13099500
H	3.08413000	-7.65805800	7.52484000
H	3.66580600	-9.00628100	6.49224600

H	4.56841000	-8.53043500	7.96952600
H	-2.31943100	3.92662500	0.05019600
H	-0.58088000	3.73125600	0.39803900
H	-1.26119800	3.08046500	-1.10525700
H	-3.22273500	-3.26842900	-0.87396800
H	-1.51612300	-3.60835000	-0.49452800
H	-2.57586000	-2.96672500	0.76821200

P1

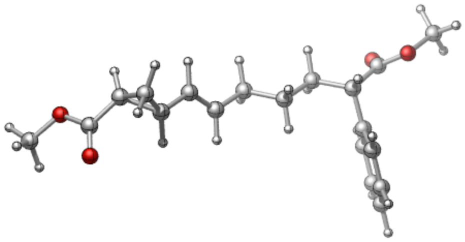

Zero-point correction= 0.397657 (Hartree/Particle)
Thermal correction to Energy= 0.421788
Thermal correction to Enthalpy= 0.422732
Thermal correction to Gibbs Free Energy= 0.338190
Sum of electronic and zero-point Energies= -1038.955938
Sum of electronic and thermal Energies= -1038.931807
Sum of electronic and thermal Enthalpies= -1038.930863
Sum of electronic and thermal Free Energies= -1039.015405
HF=-1039.3535957 (U)B3LYP/6-31G(d)
HF=-1038.3728126 (U)PBEPBE/6-311+G(d,p)-THF(SMD)//(U)B3LYP/6-31G(d)

C	0.21813600	3.86356300	1.87140000
C	0.85946100	5.06961500	1.19305200
O	1.92032200	5.56735300	1.50886800
O	0.08223800	5.54433200	0.19600200
C	1.30757900	2.83683400	2.25848900
H	1.98753500	3.29900700	2.98347500
H	1.91383100	2.62178500	1.36790200
C	0.74315700	1.52728700	2.81935300
H	0.13307600	1.73678500	3.70743600
H	0.06860400	1.06605500	2.08436700
C	1.84835500	0.51767500	3.18686000
H	2.52849300	0.99533800	3.90926100
H	2.45163800	0.28868900	2.29794600
C	1.30687500	-0.75572800	3.77530900
H	0.73074100	-0.65061900	4.69703300
C	1.46405200	-1.97561300	3.24956600
H	2.03602200	-2.08794200	2.32585300
C	0.91048500	-3.21849400	3.82849800
C	0.51414300	-4.37454700	2.95679600
C	1.75939000	-4.50266000	3.80536000
H	0.32519200	-3.09914500	4.73711600
H	0.65637600	-4.28369800	1.88331000
H	-0.34336700	-4.96389600	3.26653900
H	2.72681200	-4.45193400	3.31725100
C	1.68912000	-5.36777000	5.00928000
O	0.68711900	-5.57179200	5.66740400
O	2.89624200	-5.90612700	5.29967500
H	-0.46444600	3.40732600	1.14621500
C	2.92558300	-6.75055200	6.46108600

H	3.95630000	-7.09673700	6.54502300
H	2.63509000	-6.18881700	7.35303100
H	2.24439500	-7.59707700	6.33936800
C	0.57701400	6.72233300	-0.46312300
H	1.55087600	6.52677600	-0.91972900
H	-0.16378100	6.96314500	-1.22608600
H	0.67785400	7.54573400	0.24892100
C	-0.61149800	4.33939900	3.06351200
C	-1.96636400	4.00177100	3.16535300
C	-0.03118400	5.10992200	4.08191800
C	-2.72524200	4.41341400	4.26259100
H	-2.43080900	3.41031800	2.37974200
C	-0.78885600	5.52375700	5.17736800
H	1.01357800	5.39775000	4.00461500
C	-2.13802500	5.17550200	5.27298700
H	-3.77515800	4.13927600	4.32493700
H	-0.32441900	6.12257300	5.95658100
H	-2.72707700	5.49822800	6.12729900

B'-TS-trans

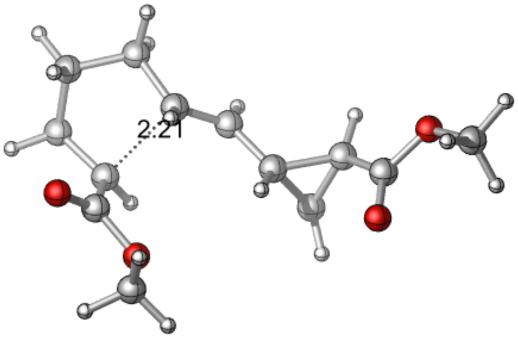

Imaginary frequency=-456.08
Zero-point correction= 0.303245 (Hartree/Particle)
Thermal correction to Energy= 0.321400
Thermal correction to Enthalpy= 0.322344
Thermal correction to Gibbs Free Energy= 0.253739
Sum of electronic and zero-point Energies= -807.335995
Sum of electronic and thermal Energies= -807.317840
Sum of electronic and thermal Enthalpies= -807.316896
Sum of electronic and thermal Free Energies= -807.385501
HF=-807.6392403 (U)B3LYP/6-31G(d)
HF=-806.9054483 (U)PBEPBE/6-311+G(d,p)-THF(SMD)//(U)B3LYP/6-31G(d)

C	-0.16102100	1.17027900	0.92547500
C	1.03607300	-0.31295000	-1.13207400
C	1.30596100	0.97324600	0.64354400
C	-0.47448800	-0.21512600	-1.10273400
C	-0.96686200	-0.00189800	0.33369400
H	-0.33991700	1.23884900	2.00651200
H	-0.49375200	2.11262800	0.47354900
H	-0.80040600	0.61660500	-1.74236000
H	-0.90935300	-1.13137200	-1.52621200
H	-2.04597700	0.19016800	0.36724000
H	-0.77108500	-0.89942900	0.93101400
C	2.06729500	0.07249400	1.50498500
C	1.79737000	0.22682200	-2.15289500
O	1.59049100	-0.72975200	2.29602400
O	3.41112200	0.18253100	1.28957400
H	1.45004500	-1.18069200	-0.61862600
H	1.34342600	0.96788500	-2.81268500
C	3.22757900	-0.04538300	-2.33893400
C	3.78199000	-0.23215300	-3.77398400
C	4.15068600	0.97438300	-2.94903800
H	3.68744700	-0.70256000	-1.60598300
H	3.08283200	-0.10856200	-4.59388800
H	5.16705300	1.00925600	-2.56920600
H	3.73094400	1.92905600	-3.25418100
C	4.80318700	-1.29546400	-3.94814500

O	5.68661300	-1.55327600	-3.15337700
O	4.62375900	-1.96256700	-5.11115600
C	4.22645100	-0.69966200	2.07097300
H	5.25418800	-0.49841200	1.76557100
H	4.09926600	-0.50125000	3.13930100
H	3.96802700	-1.74487000	1.87736200
C	5.56134500	-3.02253400	-5.35923100
H	6.58192300	-2.63235600	-5.39803000
H	5.50204800	-3.78033700	-4.57336900
H	5.27565300	-3.44584400	-6.32270100
H	1.87408300	1.79126500	0.21387700

C-pre-trans

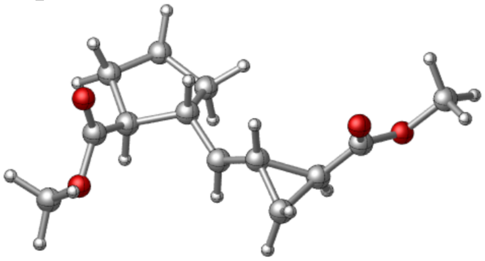

Zero-point correction= 0.305199 (Hartree/Particle)
Thermal correction to Energy= 0.323723
Thermal correction to Enthalpy= 0.324667
Thermal correction to Gibbs Free Energy= 0.253524
Sum of electronic and zero-point Energies= -807.357940
Sum of electronic and thermal Energies= -807.339417
Sum of electronic and thermal Enthalpies= -807.338472
Sum of electronic and thermal Free Energies= -807.409616
HF=-807.6631396 (U)B3LYP/6-31G(d)
HF= -806.926775 (U)PBEPBE/6-311+G(d,p)-THF(SMD)//(U)B3LYP/6-31G(d)

C	-2.13517500	4.66768800	-0.69778900
C	-2.89472400	5.78404800	0.08692600
C	-3.31599600	3.80917000	-1.22915700
H	-3.23519400	5.36813800	1.04164900
C	-1.12023100	3.92339700	0.10093000
H	-1.64523600	5.15106100	-1.55471100
H	-1.43942200	3.48711900	1.04774700
C	0.20793500	3.59457700	-0.39490500
C	1.29958000	3.08794300	0.50499400
C	0.67918900	2.09037700	-0.43377400
H	0.54964000	4.14887600	-1.26630000
H	1.07357500	2.97768300	1.56235300
H	-0.01467400	1.35808900	-0.03664300
C	-4.09425300	6.12610300	-0.82628300
C	-1.99903700	6.97205400	0.36510900
O	-1.58409700	7.74089000	-0.47767900
O	-1.68027800	7.06594800	1.67605600
C	-0.78923500	8.14145900	2.01484300
H	-0.63903600	8.06914500	3.09254400
H	0.16124600	8.03380400	1.48553000
H	-1.23300000	9.10548000	1.75203700
C	-4.40812400	4.82813400	-1.62604900
H	-5.40995500	4.44393800	-1.40796000
H	-4.37649900	5.02980100	-2.70218000
H	-4.95100600	6.48050400	-0.24395200
H	-3.80133500	6.93593200	-1.50114700
H	-3.67127800	3.15698900	-0.42052500
H	-3.00761000	3.15932500	-2.05462900

C	1.44364600	1.68452100	-1.63460500
O	2.30415000	2.35267800	-2.17682200
O	1.04888300	0.46801500	-2.08170400
C	1.72314300	0.00308100	-3.26112500
H	1.29269900	-0.97527700	-3.47784400
H	1.55725700	0.68923100	-4.09623900
H	2.79856200	-0.08162500	-3.08270200
H	2.31461100	3.39728900	0.27420900

C-TS-trans

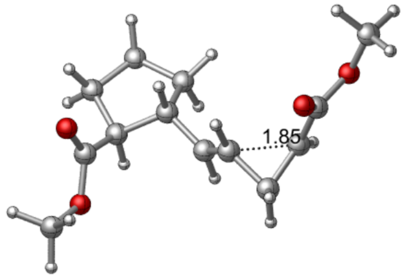

Imaginary frequency=-491.52
Zero-point correction= 0.304342 (Hartree/Particle)
Thermal correction to Energy= 0.322562
Thermal correction to Enthalpy= 0.323506
Thermal correction to Gibbs Free Energy= 0.253584
Sum of electronic and zero-point Energies= -807.354197
Sum of electronic and thermal Energies= -807.335977
Sum of electronic and thermal Enthalpies= -807.335033
Sum of electronic and thermal Free Energies= -807.404956
HF=-807.6585392 (U)B3LYP/6-31G(d)
HF= -806.9243578 (U)PBEPBE/6-311+G(d,p)-THF(SMD)//(U)B3LYP/6-31G(d)

C	2.16628600	0.03121500	-0.64233900
C	1.46033000	1.23413700	0.05918500
C	0.95314700	-0.81533200	-1.10338900
H	1.10621200	0.90942800	1.04372200
C	3.14910700	-0.69781100	0.21380000
H	2.67764100	0.43025700	-1.52876800
H	2.77246800	-1.12699300	1.14374800
C	4.48042300	-0.92581400	-0.14039200
C	5.46618200	-1.68001900	0.68671200
C	5.03148800	-2.68572200	-0.31759300
H	4.88716700	-0.42042700	-1.01209200
H	5.16890000	-1.87880300	1.71611800
H	4.23214900	-3.37730300	-0.08480500
C	0.27100600	1.55516900	-0.87674200
C	2.40499400	2.40303600	0.24152300
O	2.88274500	3.05435000	-0.66459800
O	2.68350900	2.62813600	1.54510500
C	3.60907300	3.69837600	1.79876400
H	3.71574200	3.74251100	2.88308900
H	4.57259800	3.49253200	1.32506800
H	3.21915300	4.64335800	1.41126500
C	-0.10067900	0.21369000	-1.57286100
H	-1.11528200	-0.11186400	-1.32128500
H	-0.06907900	0.33074400	-2.66136100
H	-0.56693200	1.98956300	-0.32198700
H	0.59430900	2.29691000	-1.61327300
H	0.57456800	-1.39049900	-0.24800200

H	1.23105000	-1.53676700	-1.87849600
C	5.80152200	-2.88373700	-1.53777000
O	6.73615700	-2.18489300	-1.90368900
O	5.34085600	-3.94762300	-2.25249200
C	6.04117500	-4.20221200	-3.47621500
H	5.56230700	-5.08052000	-3.91151700
H	5.96167200	-3.34770100	-4.15474100
H	7.09992000	-4.39943700	-3.28495700
H	6.50130300	-1.35719500	0.59126900

D-trans

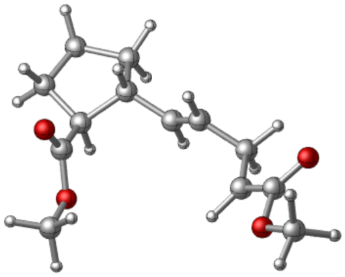

Zero-point correction= 0.304793 (Hartree/Particle)
Thermal correction to Energy= 0.323783
Thermal correction to Enthalpy= 0.324727
Thermal correction to Gibbs Free Energy= 0.251684
Sum of electronic and zero-point Energies= -807.370253
Sum of electronic and thermal Energies= -807.351263
Sum of electronic and thermal Enthalpies= -807.350319
Sum of electronic and thermal Free Energies= -807.423362
HF=-807.6750458 (U)B3LYP/6-31G(d)
HF= -806.9346044 (U)PBEPBE/6-311+G(d,p)-THF(SMD)//(U)B3LYP/6-31G(d)

C	1.71299400	-0.02831900	-0.66847000
C	1.71300600	1.25907600	0.20897300
C	0.20083500	-0.28608400	-0.85960900
H	1.45139000	0.98998000	1.23856900
C	2.46894400	-1.17998800	-0.07456100
H	2.15973500	0.24096700	-1.63470100
H	2.10731900	-1.53610800	0.89371500
C	3.51765500	-1.78661400	-0.63848100
C	4.27226900	-2.95112600	-0.03203300
C	5.68522700	-2.59046500	0.28939300
H	3.88749500	-1.43352800	-1.60159600
H	4.28125700	-3.80577800	-0.71870100
H	5.89357800	-1.77123600	0.97086100
C	0.60327700	2.12254600	-0.43275900
C	3.06712300	1.93550100	0.20844900
O	3.59160900	2.43384900	-0.76626500
O	3.64905400	1.90262600	1.42876200
C	4.95665600	2.49490100	1.50015700
H	4.91604300	3.55113700	1.22097400
H	5.27247700	2.38413000	2.53803800
H	5.64875100	1.97872200	0.82941400
C	-0.44707700	1.11613800	-0.98147100
H	-1.38648400	1.16537000	-0.42124700
H	-0.69118900	1.34937000	-2.02317300
H	0.17740200	2.83436300	0.28190700
H	1.04602700	2.70322400	-1.24811100
H	-0.18789000	-0.81131000	0.02293500
H	0.00522600	-0.92943200	-1.72358600

C	6.80442300	-3.28668100	-0.31455200
O	6.71307300	-4.21095100	-1.11303500
O	7.99768700	-2.79001900	0.11552000
C	9.15439600	-3.42985200	-0.43721300
H	10.01158700	-2.92323600	0.00813100
H	9.16606400	-4.49473200	-0.18674500
H	9.17195400	-3.32631700	-1.52620500
H	3.75550300	-3.26881900	0.88686700

D-TS

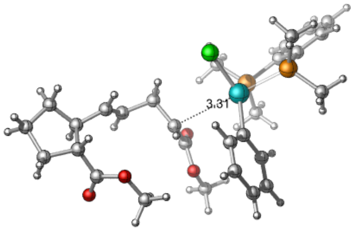

Imaginary frequency=-35.48
Zero-point correction= 0.632902 (Hartree/Particle)
Thermal correction to Energy= 0.677188
Thermal correction to Enthalpy= 0.678132
Thermal correction to Gibbs Free Energy= 0.547477
Sum of electronic and zero-point Energies= -3835.993848
Sum of electronic and thermal Energies= -3835.949562
Sum of electronic and thermal Enthalpies= -3835.948617
Sum of electronic and thermal Free Energies= -3836.079272
HF=-3836.6267496 (U)B3LYP/6-31G(d)
HF= -2695.0833054 (U)PBEPBE/6-311+G(d,p)-SDD(Fe)-THF(SMD)//(U)B3LYP/6-31G(d)

C	-2.23193600	-1.04579200	0.74373600
C	-2.79649200	-2.08136000	1.50530700
C	-3.05920500	0.00447200	0.28888500
C	-4.42601800	-0.01767300	0.60880000
C	-4.15414200	-2.08947700	1.81475600
H	-4.57273000	-2.90111700	2.40364700
C	-4.97329000	-1.05261500	1.36281000
H	-6.03437500	-1.05158000	1.59708100
H	-2.16973100	-2.89537800	1.86069700
H	-5.07484200	0.78353700	0.26462800
P	-0.42926600	-1.03210000	0.32045500
P	-2.33594700	1.40631400	-0.69148100
Fe	-0.00957800	0.79996400	-1.27328600
C	-0.07376300	-2.74975300	-0.25339100
H	1.00811800	-2.84135000	-0.39441500
H	-0.40727500	-3.51826900	0.45168100
H	-0.55246900	-2.90277900	-1.22402800
C	0.39984600	-0.97101100	1.97314600
H	0.06060100	-1.77115100	2.63968900
H	1.48050600	-1.06004300	1.82359100
H	0.20689000	-0.00503900	2.44828600
C	-3.49554000	1.65747500	-2.10196300
H	-4.54281000	1.71793200	-1.78840900
H	-3.20695300	2.60024400	-2.57691600
H	-3.37194900	0.84292400	-2.82071600
C	-2.69492600	2.87262600	0.37816500
H	-2.44021200	3.77060600	-0.19205500

H	-3.75055600	2.91602200	0.66752000
H	-2.08473500	2.83574500	1.28596300
C	0.23220600	3.37751400	-3.33074300
H	1.30030200	3.21167900	-3.24712900
Cl	-0.32420200	-0.41738200	-3.18765800
C	1.63025700	1.78505700	-0.60070100
C	2.87962900	1.66892800	-1.24805400
C	1.62066200	2.53812400	0.59176300
C	4.03983800	2.26473800	-0.74332600
H	2.95745500	1.09960000	-2.17467300
C	2.77089600	3.14288300	1.10844400
H	0.68713800	2.67044600	1.14311100
C	3.98805500	3.00732800	0.43891700
H	4.98573800	2.14523900	-1.26872500
H	2.71915500	3.71793300	2.03138300
H	4.88773400	3.47237400	0.83542500
C	-0.47012100	2.92657900	-4.57412800
H	-0.26511300	1.86774600	-4.76402700
H	-1.55159500	3.05658200	-4.46565100
C	0.06823200	3.78283200	-5.70508100
H	-0.27440700	4.81827800	-5.72231500
C	0.99559700	3.38384300	-6.58236800
H	1.35163400	2.35147200	-6.54728200
C	1.66239200	4.27811200	-7.58362200
C	3.17468900	4.51713500	-7.27290800
C	1.71944700	3.75421500	-9.03294000
H	1.17474400	5.26261900	-7.57385300
C	3.70392500	5.16828100	-8.56794300
H	3.65168200	3.54628500	-7.09787700
C	2.83050500	4.58732400	-9.71909800
H	0.75009500	3.83082700	-9.53668000
H	1.99058500	2.69006300	-9.01927500
H	3.57574800	6.25236800	-8.49034000
H	4.77235400	4.97524000	-8.70981200
H	2.39802200	5.39875700	-10.31433100
H	3.41934300	3.97325000	-10.40834500
C	3.34284300	5.37033800	-6.03267100
O	3.75148200	4.63536300	-4.97608100
O	3.09965700	6.55857600	-5.96960400
C	3.83431500	5.33680100	-3.72133000
H	4.51983300	6.18454400	-3.80145100
H	2.84719200	5.69543800	-3.41897000
H	4.20004800	4.60498100	-3.00060600
C	-0.36381900	4.34638500	-2.42980800
O	0.57544900	5.00853900	-1.70562200
O	-1.56922900	4.56949200	-2.31615500

C	0.08386300	5.97335100	-0.76981600
H	0.96912800	6.46519400	-0.36569100
H	-0.56948000	6.69804300	-1.26348700
H	-0.47089900	5.48360600	0.03635500

P2

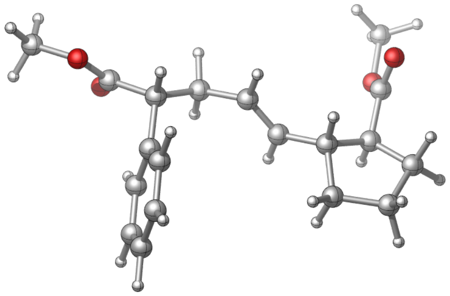

Zero-point correction= 0.399535 (Hartree/Particle)
Thermal correction to Energy= 0.423172
Thermal correction to Enthalpy= 0.424116
Thermal correction to Gibbs Free Energy= 0.340523
Sum of electronic and zero-point Energies= -1038.978760
Sum of electronic and thermal Energies= -1038.955124
Sum of electronic and thermal Enthalpies= -1038.954179
Sum of electronic and thermal Free Energies= -1039.037773
HF=-1039.3782953 (U)B3LYP/6-31G(d)
HF= -1038.3955761 (U)PBEPBE/6-311+G(d,p)-THF(SMD)//(U)B3LYP/6-31G(d)

C	-1.03412000	1.22556100	0.45498800
C	0.21768900	0.49551600	-0.08238400
C	1.03350600	1.66827300	-0.70139000
C	-0.04591600	2.51014500	-1.42070600
C	-1.34363900	2.33443800	-0.58153900
H	-1.86859100	0.53542100	0.61539500
H	-0.79803800	1.66940500	1.43114200
H	0.25657500	3.55756200	-1.52190800
H	-0.18146100	2.11007500	-2.43054300
H	-1.63619400	3.26658100	-0.08707100
H	-2.17963000	2.04779200	-1.22845200
C	2.11427700	1.16814300	-1.63552700
O	1.91136900	0.55767100	-2.66530800
O	3.35255900	1.46331000	-1.18010900
C	0.95756900	-0.30143800	0.95093600
H	1.30393300	0.25660500	1.82419700
C	1.20488400	-1.61247400	0.88076700
H	0.85258900	-2.16784700	0.00867700
C	1.93353500	-2.41145000	1.92454900
H	2.31850700	-1.75885700	2.71435800
H	2.80685300	-2.90332800	1.47250000
C	1.04669600	-3.51410300	2.56559300
H	-0.07479700	-0.16454700	-0.91005600
C	4.43586700	0.98702600	-1.99492100
H	5.34673100	1.30759000	-1.48821800
H	4.40426100	-0.10275800	-2.07632300
H	4.38130300	1.41820600	-2.99820900
C	1.89248100	-4.39561800	3.47717400

O	2.81105900	-4.00769800	4.16911000
O	1.47545500	-5.67922300	3.44396800
C	2.17299400	-6.57706100	4.32396200
H	2.06369900	-6.25646400	5.36338600
H	1.71024000	-7.55262500	4.17246600
H	3.23647100	-6.61189300	4.07306800
H	0.64414900	-4.15501300	1.77379100
C	-0.12908600	-2.94261500	3.35453700
C	-1.43781800	-3.08931200	2.88110600
C	0.08011200	-2.24245400	4.55118300
C	-2.51593800	-2.54385300	3.58022700
H	-1.61434600	-3.63311000	1.95611100
C	-0.99626700	-1.69832400	5.25190000
H	1.08942500	-2.13601700	4.93930100
C	-2.29804800	-1.84553800	4.76840100
H	-3.52533000	-2.66725800	3.19632400
H	-0.81725100	-1.16063200	6.17948400
H	-3.13594700	-1.42131000	5.31527100
H	1.50086800	2.24390600	0.10541600

Fe(I)-Cl

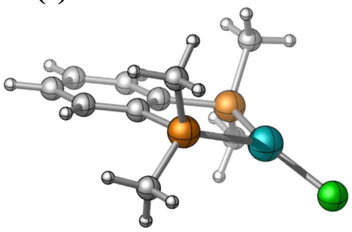

Zero-point correction= 0.236363 (Hartree/Particle)
Thermal correction to Energy= 0.254976
Thermal correction to Enthalpy= 0.255920
Thermal correction to Gibbs Free Energy= 0.187443
Sum of electronic and zero-point Energies= -2797.045046
Sum of electronic and thermal Energies= -2797.026433
Sum of electronic and thermal Enthalpies= -2797.025489
Sum of electronic and thermal Free Energies= -2797.093967
HF=-2797.2814095 (U)B3LYP/6-31G(d)
HF= -1656.7458714 (U)PBEPBE/6-311+G(d,p)-SDD(Fe)-THF(SMD)//(U)B3LYP/6-31G(d)

C	-2.06090600	-0.40717400	0.71970300
C	-2.76063700	-1.40942300	1.40683000
C	-2.76057500	0.44355600	-0.16143400
C	-4.14135800	0.26922900	-0.33277900
C	-4.13422800	-1.57099700	1.23091300
H	-4.66308900	-2.35186300	1.77100300
C	-4.82684400	-0.72908700	0.35801400
H	-5.89727800	-0.85162800	0.21556900
H	-2.23339700	-2.07435400	2.08674000
H	-4.69223200	0.91632300	-1.01106400
P	-0.23157900	-0.12886100	0.91858400
P	-1.78942100	1.76681900	-1.03810500
Fe	0.45699100	1.45343800	-0.61424200
Cl	2.31679500	1.83064000	-1.72264600
C	0.46819000	-1.84179200	0.87835200
H	1.54321600	-1.77548800	1.07348400
H	0.01318400	-2.51011500	1.61800000
H	0.33011600	-2.26753000	-0.11974800
C	-0.07439000	0.28522100	2.72111900
H	-0.56119100	-0.45882700	3.36122400
H	0.98802200	0.33464800	2.98101800
H	-0.52191900	1.26357800	2.92194400
C	-2.44120500	1.70188600	-2.76951300
H	-3.53018300	1.81057100	-2.82509300
H	-1.97393900	2.51106800	-3.33961500
H	-2.15356600	0.75243500	-3.23033000
C	-2.57421800	3.32793200	-0.41181000
H	-2.17170400	4.17711100	-0.97350700

H	-3.66422300	3.31580500	-0.52064900
H	-2.33198400	3.47092600	0.64586100

C-rotaTS

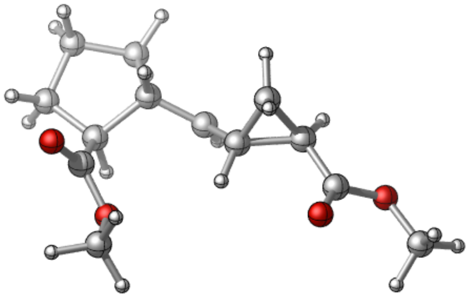

Imaginary frequency=-20.96
Zero-point correction= 0.304933 (Hartree/Particle)
Thermal correction to Energy= 0.322559
Thermal correction to Enthalpy= 0.323503
Thermal correction to Gibbs Free Energy= 0.255593
Sum of electronic and zero-point Energies= -807.353533
Sum of electronic and thermal Energies= -807.335907
Sum of electronic and thermal Enthalpies= -807.334963
Sum of electronic and thermal Free Energies= -807.402874
HF=-807.6584664 (U)B3LYP/6-31G(d)
HF= -806.9200327 (U)PBEPBE/6-311+G(d,p)-THF(SMD)//(U)B3LYP/6-31G(d)

C	-2.46587000	4.45941200	-0.90322300
C	-2.90505100	5.56929100	0.14093600
C	-3.82701500	3.89534700	-1.36399100
H	-2.97704400	5.09672300	1.12508000
C	-1.51533000	3.44263700	-0.36908000
H	-2.00360700	4.98370900	-1.75164400
H	-1.91077000	2.69423900	0.31823700
C	-0.04786700	3.57990200	-0.49635400
C	0.81856700	2.34596700	-0.72072500
C	0.65024800	3.39057900	-1.81350400
H	0.42608300	4.32219500	0.15097100
H	0.31358300	1.39425700	-0.84935400
H	0.05822900	3.09682300	-2.67558700
C	-4.29687800	6.06584800	-0.34362600
C	-1.87047500	6.67192400	0.21606300
O	-1.79971400	7.62015000	-0.53809100
O	-0.98268400	6.45419300	1.21585100
C	0.07303600	7.42526200	1.32399900
H	0.68324500	7.10075700	2.16719400
H	0.66783600	7.45050600	0.40729600
H	-0.34071500	8.42047900	1.50671000
C	-4.71294400	5.13901300	-1.51340100
H	-5.78241700	4.90367600	-1.50405100
H	-4.49894900	5.62772400	-2.47165000
H	-5.01509100	6.00754500	0.48111900
H	-4.24239400	7.11039000	-0.66003300

H	-4.22509900	3.22764800	-0.58657900
H	-3.73902000	3.30854100	-2.28501200
C	2.12610200	2.29534500	-0.01888500
O	2.83508300	3.25631800	0.21083600
O	2.44102200	1.03127100	0.34755900
C	3.69170900	0.88833200	1.03900400
H	3.68736800	1.46703900	1.96670300
H	3.78809600	-0.17679400	1.25229100
H	4.51964400	1.23136100	0.41268700
H	1.51483600	4.01221200	-2.02511900

C-pre-cis

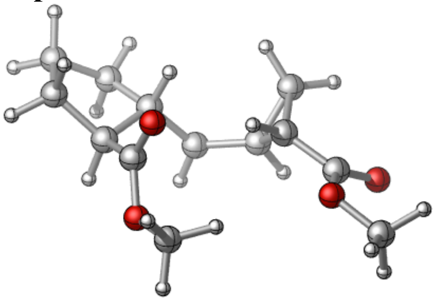

Zero-point correction= 0.305830 (Hartree/Particle)
Thermal correction to Energy= 0.324038
Thermal correction to Enthalpy= 0.324982
Thermal correction to Gibbs Free Energy= 0.257211
Sum of electronic and zero-point Energies= -807.358501
Sum of electronic and thermal Energies= -807.340293
Sum of electronic and thermal Enthalpies= -807.339349
Sum of electronic and thermal Free Energies= -807.407120
HF=-807.6643314 (U)B3LYP/6-31G(d)
HF= -806.9252484 (U)PBEPBE/6-311+G(d,p)-THF(SMD)//(U)B3LYP/6-31G(d)

C	-2.31608000	4.52670600	-0.80606500
C	-2.79467200	5.75872400	0.03702900
C	-3.64694900	3.75821600	-1.00688000
H	-2.97001500	5.43234900	1.06759100
C	-1.22496000	3.72592300	-0.18162200
H	-1.99381200	4.92502100	-1.77744200
H	-1.43584500	3.27868600	0.78823500
C	0.13132900	3.57138600	-0.69644500
C	0.51384200	3.65159600	-2.15217700
C	0.93665900	4.79537000	-1.27507300
H	0.77023500	2.91376900	-0.11290200
H	-0.26522400	3.85539400	-2.88114200
H	0.40336300	5.73513100	-1.35998100
C	-4.11766400	6.16572000	-0.64851600
C	-1.75578600	6.85712900	0.03787200
O	-1.39841300	7.46622200	-0.95288300
O	-1.22887400	7.05870800	1.26262700
C	-0.16165300	8.02391800	1.32665200
H	0.07428500	8.12494200	2.38655900
H	0.70829800	7.66129100	0.77218100
H	-0.48372200	8.98091100	0.90905400
C	-4.72953400	4.84728400	-1.20303900
H	-5.65645700	4.58033100	-0.68500600
H	-4.98445700	4.96192800	-2.26192800
H	-4.78803200	6.68556800	0.04370800
H	-3.88833300	6.85920100	-1.46371900
H	-3.85413500	3.16692800	-0.10546600

H	-3.58619600	3.05371500	-1.84278000
C	2.35213900	4.87883200	-0.85275600
O	3.13183900	3.94608200	-0.80740800
H	1.29901600	2.97940200	-2.48550400
O	2.68475600	6.14330500	-0.48415600
C	4.04090400	6.31172700	-0.04249300
H	4.14428100	7.37249100	0.18873900
H	4.23611700	5.70372000	0.84527800
H	4.74110900	6.02093700	-0.83001100

C-TS-cis

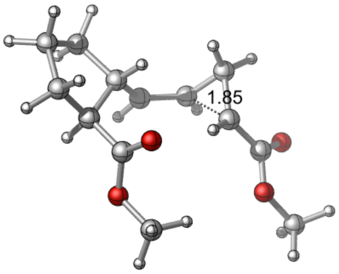

Imaginary frequency=-495.73
Zero-point correction= 0.304924 (Hartree/Particle)
Thermal correction to Energy= 0.322834
Thermal correction to Enthalpy= 0.323778
Thermal correction to Gibbs Free Energy= 0.256544
Sum of electronic and zero-point Energies= -807.354632
Sum of electronic and thermal Energies= -807.336722
Sum of electronic and thermal Enthalpies= -807.335778
Sum of electronic and thermal Free Energies= -807.403011
HF=-807.6595558 (U)B3LYP/6-31G(d)
HF= -806.9224317 (U)PBEPBE/6-311+G(d,p)-THF(SMD)//(U)B3LYP/6-31G(d)

C	2.13645500	0.01752100	-0.71047300
C	1.48379100	1.21102900	0.05796700
C	0.89077300	-0.81724400	-1.09924100
H	1.16323800	0.86345000	1.04623500
C	3.17011800	-0.72081100	0.07477900
H	2.57610200	0.43696600	-1.62419000
H	2.85581300	-1.09051000	1.05047600
C	4.51344100	-0.91146300	-0.26928500
C	5.19100000	-0.55682300	-1.55287200
C	5.55070900	0.57290900	-0.65848500
H	5.12181200	-1.50625600	0.40535600
H	4.54924100	-0.30409400	-2.39519400
H	4.96636500	1.48321700	-0.69006300
C	0.26423800	1.56725500	-0.81997000
C	2.46316900	2.35023300	0.23513500
O	2.97333700	2.96641300	-0.68161800
O	2.74540000	2.57743300	1.53308600
C	3.75034600	3.57807600	1.78894700
H	3.74083500	3.72819500	2.86903800
H	4.73000900	3.21795800	1.46225400
H	3.51004500	4.50658200	1.26606400
C	-0.20895300	0.22070100	-1.43907000
H	-1.17941700	-0.08975000	-1.03849200
H	-0.33473500	0.32149800	-2.52220000
H	-0.51979900	2.06751400	-0.24241600
H	0.58807500	2.26169100	-1.60190200
H	0.58967700	-1.43135400	-0.24088500

H	1.10584300	-1.50519700	-1.92321600
C	6.79331500	0.55482900	0.09732000
O	7.56932900	-0.38694500	0.17448400
O	6.99412700	1.73632500	0.75429900
C	8.19624400	1.79683100	1.53174100
H	8.21454300	2.79637200	1.96862600
H	8.18970600	1.03572500	2.31764400
H	9.07555500	1.64036200	0.90051900
H	6.01674700	-1.21151500	-1.82643100

D-cis

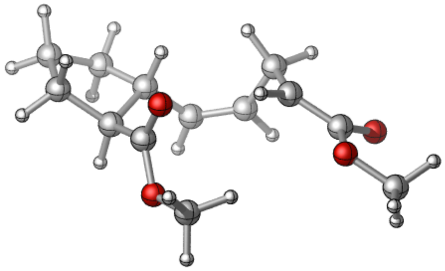

Zero-point correction= 0.305856 (Hartree/Particle)
Thermal correction to Energy= 0.324330
Thermal correction to Enthalpy= 0.325275
Thermal correction to Gibbs Free Energy= 0.256130
Sum of electronic and zero-point Energies= -807.369038
Sum of electronic and thermal Energies= -807.350563
Sum of electronic and thermal Enthalpies= -807.349619
Sum of electronic and thermal Free Energies= -807.418764
HF=-807.6748936 (U)B3LYP/6-31G(d)
HF= -806.9314457 (U)PBEPBE/6-311+G(d,p)-THF(SMD)//(U)B3LYP/6-31G(d)

C	1.76174800	2.39826700	-1.02526800
C	2.25906200	3.56649700	-0.11896800
C	0.32148300	2.18835000	-0.50970300
H	2.48173000	3.17475300	0.87964700
C	2.63585700	1.17974200	-0.96273500
H	1.72200100	2.79159500	-2.04800600
H	2.66384800	0.68926000	0.01260700
C	3.38752700	0.65455100	-1.93931300
C	3.53903900	1.16910600	-3.36473100
C	4.74131800	2.05614900	-3.43264600
H	4.01191900	-0.20655100	-1.70809200
H	2.64931400	1.72266600	-3.67974300
H	4.64151300	3.11778300	-3.23251000
C	1.04326500	4.52101900	-0.07525300
C	3.50831000	4.20938300	-0.68149200
O	3.56652400	4.76915100	-1.76040600
O	4.56774200	4.06575300	0.13876300
C	5.81394400	4.59856100	-0.34872900
H	6.51479600	4.50011300	0.48085100
H	6.16314400	4.02571800	-1.21238800
H	5.69731500	5.64658700	-0.63482200
C	-0.21122500	3.60760600	-0.19028900
H	-0.80100100	3.60970200	0.73209300
H	-0.87248200	3.96759000	-0.98551400
H	1.04142300	5.13252900	0.83278600
H	1.10435900	5.20346800	-0.92900900
H	0.35199200	1.57679400	0.40181400
H	-0.29707700	1.64874100	-1.23430800

C	6.06345800	1.49159500	-3.59484800
O	6.31013700	0.30690900	-3.78666600
O	7.03716900	2.44653900	-3.50425600
C	8.37510600	1.96282100	-3.67144300
H	9.01845700	2.83948400	-3.58429200
H	8.62261400	1.22760400	-2.89983000
H	8.49828300	1.49436900	-4.65231800
H	3.67286900	0.31339300	-4.03536300

OBn System

A•

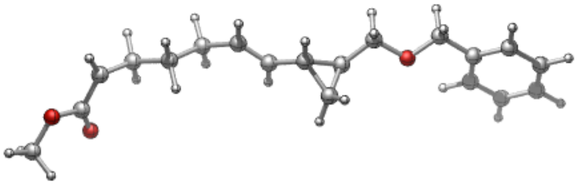

Zero-point correction= 0.402074 (Hartree/Particle)
Thermal correction to Energy= 0.425609
Thermal correction to Enthalpy= 0.426553
Thermal correction to Gibbs Free Energy= 0.340887
Sum of electronic and zero-point Energies= -964.250607
Sum of electronic and thermal Energies= -964.227073
Sum of electronic and thermal Enthalpies= -964.226128
Sum of electronic and thermal Free Energies= -964.311795
HF=-964.6526813 (U)B3LYP/6-31G(d)
HF= -963.7124893 (U)PBEPBE/6-311+G(d,p)-THF(SMD)//(U)B3LYP/6-31G(d)

C	0.95960600	4.22614900	2.58852400
C	1.28467800	4.74695800	1.27548000
O	2.28331600	4.45906600	0.62709300
O	0.33417200	5.61594400	0.82869000
H	0.02893400	4.54887900	3.04711800
C	1.83508600	3.24179600	3.27658100
H	2.09152100	3.61825300	4.27996600
H	2.76873600	3.14069600	2.71359800
C	1.16814100	1.85391700	3.44141000
H	0.21734200	1.96501700	3.98099400
H	0.92089400	1.44891700	2.45277800
C	2.07352700	0.85690900	4.19069200
H	2.33978700	1.29403800	5.16600100
H	3.01418200	0.73248700	3.63818700
C	1.42125800	-0.48240500	4.39547700
H	0.51315500	-0.48597700	5.00250100
C	1.85858700	-1.63734200	3.88074200
H	2.76402700	-1.63442300	3.26963400
C	1.21185100	-2.95374000	4.07003500
C	1.23110000	-3.99252500	2.96054200
C	2.05898800	-4.21335300	4.19279300
H	0.30904800	-2.93183700	4.67907300
H	1.74277200	-3.73780400	2.03628300
H	0.34220400	-4.60314500	2.82407700
H	3.13375000	-4.07680400	4.08702700
C	0.58639400	6.17556500	-0.46533700
H	-0.25435200	6.84058500	-0.66739600
H	1.52669900	6.73486700	-0.47031800

H	0.64345400	5.38949600	-1.22422000
C	1.67193800	-5.26845100	5.19432400
H	2.10037400	-5.03628700	6.18504500
H	0.57442000	-5.29582800	5.31381400
C	1.89164800	-7.57316100	5.66400800
H	2.31032900	-7.30929800	6.65316700
H	0.80539900	-7.70459200	5.81012400
O	2.14613000	-6.52829500	4.74502000
C	2.50702700	-8.86409300	5.17210400
C	1.97726100	-10.09178800	5.58573500
C	3.63129300	-8.85623100	4.33930600
C	2.56442800	-11.29193800	5.18393100
H	1.09634400	-10.11001800	6.22469400
C	4.21410400	-10.05629400	3.92964900
H	4.03314400	-7.90517600	4.00644000
C	3.68597600	-11.27740800	4.35265200
H	2.14034400	-12.23730000	5.51252600
H	5.08355800	-10.03667400	3.27736800
H	4.14142600	-12.21099500	4.03298100

TS-AB

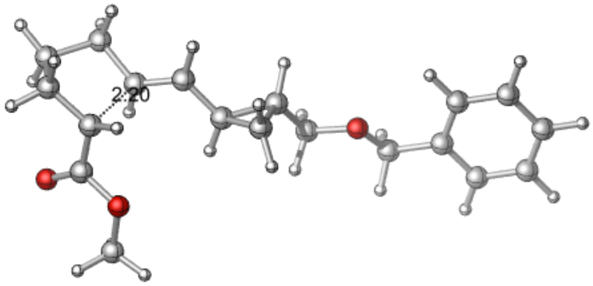

Imaginary frequency= -452.57
Zero-point correction= 0.402533 (Hartree/Particle)
Thermal correction to Energy= 0.424709
Thermal correction to Enthalpy= 0.425653
Thermal correction to Gibbs Free Energy= 0.345255
Sum of electronic and zero-point Energies= -964.239006
Sum of electronic and thermal Energies= -964.216830
Sum of electronic and thermal Enthalpies= -964.215886
Sum of electronic and thermal Free Energies= -964.296284
HF=-964.6415389 (U)B3LYP/6-31G(d)
HF= -963.705632 (U)PBEPBE/6-311+G(d,p)-THF(SMD)//(U)B3LYP/6-31G(d)

C	0.29069800	1.55046300	0.98993000
C	1.03374400	-0.17669100	-1.08457800
C	1.66443700	1.03394900	0.64594400
C	-0.42098500	0.23186700	-0.97993700
C	-0.77793800	0.56912600	0.47276400
H	0.18649100	1.67993700	2.07523400
H	0.14613000	2.53277100	0.52377100
H	-0.60312600	1.10208800	-1.62548000
H	-1.05987100	-0.58041500	-1.35413200
H	-1.78791100	0.98869300	0.55438400
H	-0.74590500	-0.33922500	1.08473400
C	2.25224000	0.00559600	1.49749800
C	1.82866500	0.18361300	-2.15850700
O	1.65246200	-0.66740700	2.32512000
O	3.58157000	-0.17698700	1.23141800
H	1.28569500	-1.10929100	-0.57941200
H	1.49650200	0.99818600	-2.80430600
C	3.16058700	-0.37216900	-2.42746700
C	3.64405500	-0.55581700	-3.86722700
C	4.26864500	0.50003900	-3.00993600
H	3.49041300	-1.12956400	-1.71906100
H	2.96160200	-0.21338300	-4.64281600
H	5.27774100	0.33453900	-2.64152300
H	4.03391100	1.53997600	-3.22009900
C	4.21984300	-1.19181800	2.01407900
H	5.25891100	-1.21159700	1.68109100
H	4.16701900	-0.95276900	3.08037800

H	3.74810700	-2.16613700	1.85381800
H	2.37522500	1.70361300	0.17391800
C	4.40801800	-1.80226100	-4.22799400
H	3.71271200	-2.62214500	-4.47920100
H	5.01210200	-2.14059500	-3.36784500
C	5.95586000	-2.65826400	-5.79398600
H	5.24349500	-3.47424400	-6.01718300
H	6.62913700	-3.03787300	-5.00614000
O	5.24790600	-1.52113200	-5.33631900
C	6.75123900	-2.31422200	-7.03307200
C	6.31865000	-1.31576500	-7.91285800
C	7.91537600	-3.02899900	-7.33710500
C	7.03875000	-1.03987200	-9.07582400
H	5.42492900	-0.75073800	-7.66993400
C	8.63124000	-2.76014000	-8.50423800
H	8.26722000	-3.80008300	-6.65437700
C	8.19451900	-1.76264200	-9.37774100
H	6.69575200	-0.25734300	-9.74796700
H	9.53430600	-3.32308100	-8.72567600
H	8.75384700	-1.54678300	-10.28423400

B•

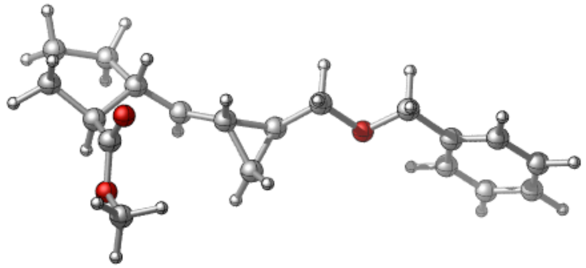

Zero-point correction= 0.404287 (Hartree/Particle)
Thermal correction to Energy= 0.426924
Thermal correction to Enthalpy= 0.427868
Thermal correction to Gibbs Free Energy= 0.344379
Sum of electronic and zero-point Energies= -964.260573
Sum of electronic and thermal Energies= -964.237936
Sum of electronic and thermal Enthalpies= -964.236992
Sum of electronic and thermal Free Energies= -964.320482
HF=-964.6648603 (U)B3LYP/6-31G(d)
HF= -963.7252391 (U)PBEPBE/6-311+G(d,p)-THF(SMD)//(U)B3LYP/6-31G(d)

C	-2.59877700	4.43834500	-1.00680200
C	-2.78666000	5.58385900	0.04804800
C	-4.06289900	4.07295200	-1.34343600
H	-3.09358800	5.14819400	1.00479200
C	-1.74960900	3.29477000	-0.56917700
H	-2.13710800	4.91881700	-1.88128800
H	-2.19064800	2.55215400	0.09607000
C	-0.31015300	3.22716600	-0.82067900
C	0.67668700	2.78776300	0.27044600
C	0.41677500	1.88134300	-0.89106100
H	0.07191900	3.99556400	-1.49130500
H	0.26319200	2.51832600	1.23824900
H	-0.20013600	1.00588200	-0.69656200
C	-3.93069900	6.42326800	-0.56305200
C	-1.51649300	6.37778500	0.25025000
O	-0.89034400	6.92023900	-0.63874900
O	-1.14063400	6.41370700	1.54861900
C	0.06931500	7.14016300	1.81785100
H	0.21667200	7.07487500	2.89638000
H	0.91248300	6.69029600	1.28678900
H	-0.02962400	8.18311700	1.50508300
C	-4.86790500	5.39629800	-1.25556700
H	-5.79180500	5.25362000	-0.68511900
H	-5.16494700	5.75378900	-2.24703800
H	-4.44508500	7.02998300	0.18958100
H	-3.49983600	7.11002900	-1.29960000
H	-4.43142800	3.35207000	-0.60205900
H	-4.14138800	3.58773200	-2.32179500

H	1.63393300	3.30185500	0.31661200
C	1.46929700	1.67108800	-1.94644500
H	1.00755400	1.33715700	-2.89193700
H	1.99164300	2.62063200	-2.15748100
C	3.38570000	0.38332100	-2.44807500
H	2.90382700	0.07686600	-3.39530800
H	3.99366600	1.27454900	-2.68159600
O	2.39431400	0.69583000	-1.48851100
C	4.27387300	-0.72972400	-1.93877100
C	5.58679900	-0.84651800	-2.40939700
C	3.79159400	-1.67910200	-1.03109100
C	6.40240000	-1.89801100	-1.99002500
H	5.97606200	-0.10746500	-3.10713600
C	4.60934300	-2.72628600	-0.60424200
H	2.77905000	-1.58016000	-0.65447200
C	5.91526200	-2.84199300	-1.08414500
H	7.42031900	-1.97412100	-2.36366100
H	4.22466200	-3.45385200	0.10606000
H	6.55061200	-3.65844400	-0.75099900

TS-BC

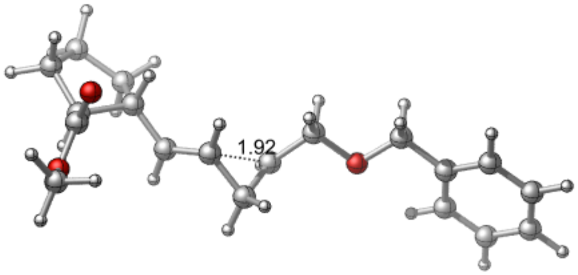

Imaginary frequency= -531.87
Zero-point correction= 0.402863 (Hartree/Particle)
Thermal correction to Energy= 0.425292
Thermal correction to Enthalpy= 0.426237
Thermal correction to Gibbs Free Energy= 0.343596
Sum of electronic and zero-point Energies= -964.249085
Sum of electronic and thermal Energies= -964.226655
Sum of electronic and thermal Enthalpies= -964.225711
Sum of electronic and thermal Free Energies= -964.308352
HF=-964.6519478 (U)B3LYP/6-31G(d)
HF= -963.7144329 (U)PBEPBE/6-311+G(d,p)-THF(SMD)//(U)B3LYP/6-31G(d)

C	2.04229900	0.00089200	-0.51317800
C	1.64706900	1.35142400	0.15995900
C	0.68047800	-0.73476500	-0.56326300
H	1.49740300	1.18641100	1.23264600
C	3.13977300	-0.74471400	0.17728000
H	2.35554800	0.24097300	-1.53839200
H	2.94306000	-1.07703100	1.19815100
C	4.34742800	-1.08136100	-0.41555900
C	5.43413900	-1.87829400	0.23584000
C	4.81153400	-2.93073200	-0.60657800
H	4.58009300	-0.65343200	-1.38825800
H	5.32744300	-2.00330500	1.31481800
H	4.04816800	-3.55647400	-0.15732200
C	0.31264000	1.71656600	-0.53030400
C	2.71775300	2.40324900	-0.02957200
O	3.09443200	2.82099400	-1.10593900
O	3.23022000	2.82389500	1.14886300
C	4.27518000	3.80479100	1.04834200
H	4.56653900	4.02910000	2.07500200
H	5.12363900	3.40532600	0.48634300
H	3.91109500	4.70513200	0.54607600
C	-0.37385400	0.36014100	-0.86057200
H	-1.27950500	0.20889500	-0.26391700
H	-0.68323200	0.33557500	-1.91086100
H	-0.30706500	2.36419300	0.09848800
H	0.53623400	2.26930000	-1.44844300
H	0.48845700	-1.19569100	0.41463400

H	0.68371500	-1.54364900	-1.30127500
H	6.45466300	-1.59821900	-0.03061300
C	5.39672300	-3.33935800	-1.91613800
H	4.64204600	-3.82918200	-2.55301600
H	5.77168000	-2.46089400	-2.47021600
C	7.07506000	-4.70554200	-2.88335400
H	6.29950100	-5.15870200	-3.52892800
H	7.50480100	-3.86500700	-3.45576000
O	6.48495200	-4.24174600	-1.68256300
C	8.14850800	-5.72480500	-2.57518800
C	8.05624700	-6.54692000	-1.44628500
C	9.23183700	-5.88770600	-3.44605800
C	9.03003000	-7.51442700	-1.19630500
H	7.22526600	-6.40954800	-0.76235800
C	10.20170600	-6.86088900	-3.20184700
H	9.31985800	-5.24632200	-4.32089200
C	10.10359400	-7.67770000	-2.07413500
H	8.95006800	-8.14251500	-0.31256800
H	11.03784100	-6.97404600	-3.88715600
H	10.86101900	-8.43213200	-1.87814700

C•

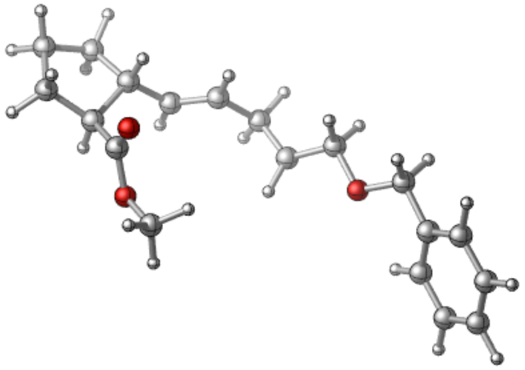

Zero-point correction= 0.403037 (Hartree/Particle)
Thermal correction to Energy= 0.426289
Thermal correction to Enthalpy= 0.427233
Thermal correction to Gibbs Free Energy= 0.341611
Sum of electronic and zero-point Energies= -964.266397
Sum of electronic and thermal Energies= -964.243145
Sum of electronic and thermal Enthalpies= -964.242201
Sum of electronic and thermal Free Energies= -964.327823
HF=-964.6694337 (U)B3LYP/6-31G(d)
HF= -963.7260033 (U)PBEPBE/6-311+G(d,p)-THF(SMD)//(U)B3LYP/6-31G(d)

C	1.75682600	-0.18012100	-0.57190000
C	1.85373500	1.17608600	0.18993700
C	0.22850000	-0.37395600	-0.68368500
H	1.62436500	1.00762100	1.24809800
C	2.48936500	-1.30976900	0.09036900
H	2.17425400	-0.01411400	-1.57415500
H	2.13603500	-1.58632200	1.08730400
C	3.52230800	-1.97312500	-0.43815700
C	4.28866500	-3.08160900	0.25628500
C	5.70744800	-2.68704000	0.54538700
H	3.88374800	-1.69145200	-1.42846400
H	4.27574300	-3.98724800	-0.36894700
H	5.89580700	-1.88793700	1.25764900
C	0.76013200	2.03897200	-0.47961200
C	3.23999000	1.77372500	0.07889500
O	3.74387600	2.16509900	-0.95418500
O	3.87634700	1.79736200	1.27100300
C	5.22247400	2.30044600	1.23904000
H	5.24091100	3.32683500	0.86312200
H	5.57408300	2.26237200	2.27053300
H	5.84906100	1.67606000	0.59660700
C	-0.34939800	1.04288300	-0.92417300
H	-1.27806200	1.19176500	-0.36356700
H	-0.59439600	1.19287700	-1.98101400
H	0.38694600	2.81575800	0.19573500

H	1.19811900	2.54290500	-1.34688000
H	-0.15052500	-0.78636100	0.26099600
H	-0.03587300	-1.08752200	-1.47106900
H	3.77034800	-3.33651200	1.19197200
C	6.83119600	-3.08414700	-0.34525900
H	6.76493200	-4.16085000	-0.59877100
H	6.78617900	-2.54653000	-1.31870100
C	9.18632000	-3.09662500	-0.51107400
H	9.13914900	-4.15269900	-0.83574000
H	9.17846000	-2.48562000	-1.43009900
O	8.06225800	-2.79642000	0.29434400
C	10.46086900	-2.85270900	0.26465400
C	10.49883400	-3.01345300	1.65440400
C	11.63583600	-2.50390700	-0.41067800
C	11.69224100	-2.82890200	2.35344600
H	9.58487100	-3.26935200	2.18014800
C	12.83187700	-2.32802700	0.28633800
H	11.61525000	-2.36513200	-1.48989400
C	12.86305600	-2.48947600	1.67252100
H	11.70736700	-2.95078800	3.43359800
H	13.73585100	-2.05574800	-0.25234700
H	13.79189600	-2.34680700	2.21853400

Figure S9
A-E

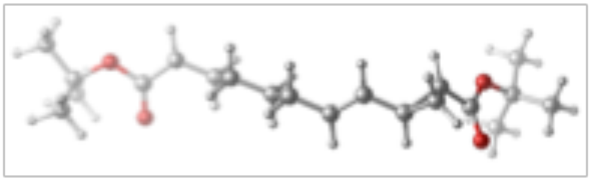

UB3LYP/6-31G(d)
Zero-point correction= 0.500116 (Hartree/Particle)
Thermal correction to Energy= 0.528750
Thermal correction to Enthalpy= 0.529694
Thermal correction to Gibbs Free Energy= 0.435634
Sum of electronic and zero-point Energies= -1082.365108
Sum of electronic and thermal Energies= -1082.336474
Sum of electronic and thermal Enthalpies= -1082.335530
Sum of electronic and thermal Free Energies= -1082.429589

C	-1.86889300	3.62484900	-1.10348200
C	-5.38470000	3.77917700	0.55414600
C	-4.14114400	4.26118300	-0.10319100
C	-3.16650200	3.11319500	-0.46614600
H	-1.36465600	4.31619500	-0.41431600
H	-2.11107800	4.20969600	-2.00226500
H	-5.34226800	3.31912500	1.53757400
H	-4.40883300	4.81719000	-1.00753100
H	-3.61609600	4.95498800	0.57221700
H	-2.93110000	2.53364100	0.43771000
H	-3.67097800	2.42335200	-1.15549800
C	-6.67648700	3.85209900	-0.10806800
O	-7.65335800	3.33157700	0.67968300
O	-6.84666500	4.32328800	-1.22823800
C	-9.05573500	3.28861500	0.24129400
C	-9.58202100	4.71131400	0.01671600
H	-9.07626300	5.19173900	-0.82224500
H	-10.65706100	4.67591100	-0.19366100
H	-9.43185700	5.31819000	0.91643100
C	-9.75906300	2.63408500	1.43363700
H	-9.62565500	3.23606300	2.33840600
H	-10.83182600	2.53956900	1.23453100
H	-9.35153700	1.63541800	1.62180800
C	-9.18972100	2.41653100	-1.01276800
H	-8.76267200	1.42375700	-0.83262700
H	-10.24972600	2.29018600	-1.26087100
H	-8.68173100	2.87066500	-1.86487300
C	-0.89443700	2.49202700	-1.48112200
H	-0.62996100	1.91846000	-0.58239500

H	-1.41565900	1.79377000	-2.15431700
C	0.35510000	2.99636400	-2.14902600
H	0.21208900	3.53174900	-3.09000200
C	1.59685700	2.85144300	-1.67314600
H	1.74764400	2.31771000	-0.73211700
C	2.81603900	3.35931600	-2.33749900
C	3.94132300	3.96803600	-1.48198700
C	4.13289000	2.65132000	-2.19970700
H	2.66205400	3.87240100	-3.28377000
H	3.80953800	3.95542300	-0.40538900
H	4.79731700	2.66186300	-3.05822000
H	4.17564700	1.75546400	-1.58600700
C	4.62452200	5.16847500	-2.03733300
O	4.87384900	5.31982800	-3.22019300
O	4.90888600	6.04650800	-1.05436000
C	5.57027400	7.33566400	-1.33257800
C	6.96320800	7.09410000	-1.92408600
H	6.89915300	6.63547400	-2.91195000
H	7.49376900	8.04895600	-2.01303400
H	7.54798400	6.44128000	-1.26665500
C	5.67318200	7.96316800	0.05982600
H	6.15519500	8.94454200	-0.00371500
H	4.67919000	8.09255600	0.50011700
H	6.26536600	7.32836200	0.72697200
C	4.68774000	8.18849700	-2.25026900
H	3.68659000	8.29991200	-1.81945700
H	5.12564200	9.18773900	-2.35426100
H	4.59852200	7.73974800	-3.24076800

UPBEPBE/6-311+G(d,p)-THF(SMD)//UB3LYP/6-31G(d)
HF= -1081.7995031
DLPNO-CCSD(T)/def2-TZVPP-gas//UB3LYP/6-31G(d)
HF= -1081.028528411918

A-E-anti-TS

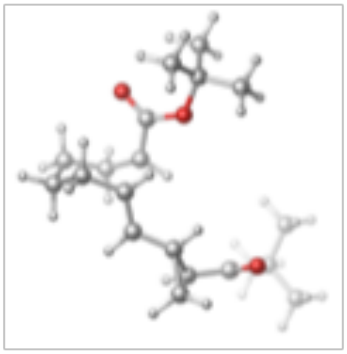

Imaginary frequency= -418.70
UB3LYP/6-31G(d)
Zero-point correction= 0.501209 (Hartree/Particle)
Thermal correction to Energy= 0.528175
Thermal correction to Enthalpy= 0.529119
Thermal correction to Gibbs Free Energy= 0.441673
Sum of electronic and zero-point Energies= -1082.347820
Sum of electronic and thermal Energies= -1082.320854
Sum of electronic and thermal Enthalpies= -1082.319910
Sum of electronic and thermal Free Energies= -1082.407356

C	-2.45644100	2.09884200	-0.47973900
C	-0.43577100	4.51881900	-0.60738100
C	-1.93033400	4.61317000	-0.77603400
C	-2.76597200	3.53943900	-0.05023500
H	-2.72267200	1.95633200	-1.53704400
H	-3.10392900	1.41683000	0.08644600
H	0.17565500	4.92658200	-1.40573900
H	-2.26495900	5.60270500	-0.42449000
H	-2.15194900	4.58065300	-1.85099200
H	-2.60584300	3.63956800	1.02738300
H	-3.82710500	3.74827000	-0.23985500
C	0.17101600	4.60314900	0.72026900
O	1.52153800	4.76525800	0.61774600
O	-0.43445600	4.50757400	1.78328300
C	2.38083800	4.86772000	1.80464200
C	1.99579800	6.10151700	2.63006200
H	0.99615400	5.99481900	3.05378600
H	2.71357900	6.23600100	3.44741400
H	2.02195300	7.00032000	2.00399000
C	3.77549400	5.03996800	1.19565400
H	3.81683500	5.93968200	0.57321500
H	4.52602500	5.13056900	1.98820700
H	4.03372900	4.17876100	0.57063800
C	2.30824600	3.57655400	2.62908300
H	2.55050800	2.71055100	2.00248600

H	3.04067200	3.61848800	3.44345400
H	1.31431400	3.43762300	3.05673600
C	-0.99294100	1.68570800	-0.24413800
H	-0.73388900	1.89208600	0.80063800
H	-0.89582800	0.59804700	-0.37510700
C	0.02141700	2.36954200	-1.14686200
H	1.01598700	2.50976500	-0.72675400
C	-0.06405600	2.34441800	-2.51973000
H	-0.99874400	2.05124800	-2.99792500
C	1.01922100	2.78219400	-3.40980400
C	1.12105000	2.28282800	-4.82505100
C	0.69305900	3.71090800	-4.60915900
H	1.96679900	3.01753800	-2.93099800
H	0.36550800	1.58583200	-5.17739600
H	2.11906900	2.13458100	-5.22563000
H	-0.34888700	3.97069100	-4.76111300
C	1.68230600	4.78649300	-4.89455900
O	2.88868000	4.63020200	-4.82856100
O	1.05116600	5.93369600	-5.21377900
C	1.79682800	7.16885300	-5.52713800
C	2.67127300	6.95221300	-6.76677100
H	3.12272600	7.90465600	-7.06692200
H	3.46825600	6.23395300	-6.56834600
H	2.06417100	6.58693800	-7.60243400
C	2.61368600	7.60923100	-4.30784200
H	3.40658900	6.89376200	-4.08483200
H	3.06679700	8.58737200	-4.50514100
H	1.96571500	7.70701300	-3.42983900
C	0.67534900	8.16834500	-5.82091700
H	0.06461800	7.82890300	-6.66383200
H	0.02388400	8.28593100	-4.94891700
H	1.09945700	9.14640000	-6.07160400

UPBEPBE/6-311+G(d,p)-THF(SMD)//UB3LYP/6-31G(d)
HF= -1081.7862163
DLPNO-CCSD(T)/def2-TZVPP-gas//UB3LYP/6-31G(d)
HF= -1081.018617171460

B-E-anti

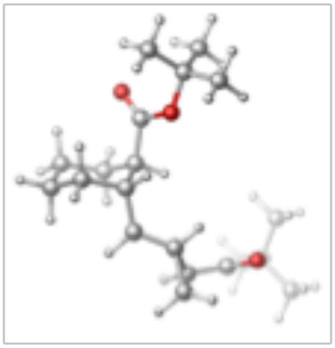

UB3LYP/6-31G(d)
Zero-point correction= 0.503410 (Hartree/Particle)
Thermal correction to Energy= 0.530298
Thermal correction to Enthalpy= 0.531242
Thermal correction to Gibbs Free Energy= 0.444162
Sum of electronic and zero-point Energies= -1082.377500
Sum of electronic and thermal Energies= -1082.350612
Sum of electronic and thermal Enthalpies= -1082.349668
Sum of electronic and thermal Free Energies= -1082.436748

C	-2.34155400	2.10760600	-0.47670900
C	-0.20844000	4.19808000	-0.69981000
C	-1.69898300	4.53714300	-0.88117300
C	-2.63330500	3.57475500	-0.12918400
H	-2.61909100	1.91524200	-1.52309800
H	-2.96787300	1.44253800	0.13150800
H	0.38943400	4.80907300	-1.38908500
H	-1.88101600	5.56601500	-0.55293900
H	-1.92231300	4.49803100	-1.95596200
H	-2.50698800	3.72987600	0.94812600
H	-3.67716400	3.81829400	-0.36525900
C	0.29394400	4.56304500	0.69805800
O	1.62263000	4.34040200	0.78843900
O	-0.39668900	5.01065500	1.59298700
C	2.38373100	4.61405100	2.02283700
C	2.32598800	6.10992000	2.35090600
H	1.31327600	6.41772500	2.61565400
H	2.99125600	6.32528700	3.19475000
H	2.66493300	6.70115600	1.49310300
C	3.80510100	4.19717000	1.63683600
H	4.16116600	4.78236300	0.78290500
H	4.48708800	4.36028300	2.47808100
H	3.83745600	3.13706700	1.36487100
C	1.85492800	3.74625800	3.16975700
H	1.85967400	2.68934500	2.88071500
H	2.50483400	3.86040000	4.04477000

H	0.83966000	4.03293900	3.44768800
C	-0.85971700	1.76395300	-0.25863100
H	-0.63013100	1.84983300	0.81297700
H	-0.66222300	0.72113300	-0.53720900
C	0.09300600	2.68977600	-1.04641300
H	1.12145800	2.50181900	-0.70995400
C	0.04420500	2.43665200	-2.52387300
H	-0.83251300	1.96577800	-2.96330100
C	1.09065200	2.91198200	-3.42037900
C	1.25242100	2.38193600	-4.81844200
C	0.72031700	3.78146600	-4.67610900
H	2.01231600	3.24958500	-2.95013300
H	0.55535400	1.61891500	-5.15475700
H	2.26533900	2.29025600	-5.19880300
H	-0.33557400	3.95815300	-4.84699300
C	1.63395200	4.91363500	-4.97499300
O	2.84793800	4.84841600	-4.88186900
O	0.92960900	6.00639900	-5.33687400
C	1.59338300	7.28074600	-5.67070600
C	2.50478600	7.09409000	-6.88864700
H	2.89816500	8.06709300	-7.20434200
H	3.34318400	6.43490200	-6.65853600
H	1.93897700	6.66971400	-7.72540200
C	2.35359400	7.81073900	-4.45012400
H	3.18703300	7.15670100	-4.18933400
H	2.74548200	8.81071700	-4.66833800
H	1.68156000	7.89143000	-3.58848200
C	0.41171800	8.19191700	-6.01205300
H	-0.15671400	7.78739700	-6.85572200
H	-0.26329400	8.28669800	-5.15535200
H	0.77182200	9.19016300	-6.28265700

UPBEPBE/6-311+G(d,p)-THF(SMD)//UB3LYP/6-31G(d)
HF= -1081.8150171
DLPNO-CCSD(T)/def2-TZVPP-gas//UB3LYP/6-31G(d)
HF= -1081.057484486941

ROT-E-anti-TS

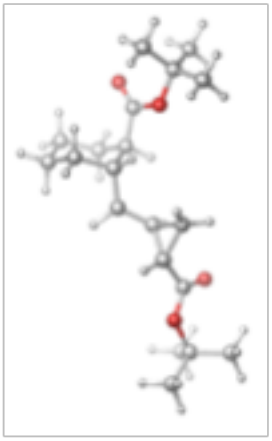

Imaginary frequency= -21.34
UB3LYP/6-31G(d)
Zero-point correction= 0.502845 (Hartree/Particle)
Thermal correction to Energy= 0.529108
Thermal correction to Enthalpy= 0.530052
Thermal correction to Gibbs Free Energy= 0.444557
Sum of electronic and zero-point Energies= -1082.372624
Sum of electronic and thermal Energies= -1082.346361
Sum of electronic and thermal Enthalpies= -1082.345416
Sum of electronic and thermal Free Energies= -1082.430912

C	0.22861800	0.78213900	-0.83033700
C	-0.61416600	3.61995500	-0.38048300
C	-1.58369800	2.56521000	-0.94409200
C	-1.23248500	1.13129900	-0.51316700
H	0.36600400	0.73271600	-1.92001300
H	0.47186700	-0.21630100	-0.44506300
H	-0.81458600	4.59051500	-0.85344000
H	-2.60651700	2.80736400	-0.63646900
H	-1.55214800	2.63420000	-2.03999900
H	-1.40931800	1.03232200	0.56364500
H	-1.90631700	0.42353300	-1.01276300
C	-0.83441300	3.85606700	1.11430300
O	-0.04799200	4.86447400	1.54860700
O	-1.62172800	3.24314900	1.80885200
C	-0.07075300	5.32923000	2.94909100
C	-1.46047400	5.87411000	3.29607200
H	-2.20855400	5.07999400	3.29417600
H	-1.43607200	6.33195800	4.29147700
H	-1.75787100	6.64424700	2.57586300
C	0.96660800	6.45494200	2.94328300
H	0.68123600	7.23934300	2.23491600
H	1.04563700	6.90018600	3.94077100
H	1.95160400	6.07283000	2.65576000

C	0.36080700	4.19601200	3.88574400
H	1.33099000	3.79328500	3.57388400
H	0.46700600	4.58364200	4.90530800
H	-0.37221100	3.38804500	3.89222800
C	1.19498500	1.81768500	-0.23299200
H	1.12976600	1.77479900	0.86343300
H	2.23215300	1.56942000	-0.49294900
C	0.88494500	3.25778000	-0.69586700
H	1.49266300	3.95792800	-0.10486900
C	1.21831700	3.47516100	-2.14336800
H	1.41531700	2.61782400	-2.78360100
C	1.16310900	4.81733700	-2.76669200
C	2.07368100	5.92816100	-2.32611400
C	2.29414400	5.27738600	-3.68227800
H	0.18442700	5.16691200	-3.10835500
H	2.81468900	5.71641700	-1.56068000
H	1.68168000	6.94057400	-2.33383500
H	3.14832900	4.61766400	-3.79245000
C	1.92056500	6.06480600	-4.88987500
O	0.98583700	6.84543100	-4.92714900
O	2.75331900	5.78553900	-5.91286800
C	2.60561600	6.42893900	-7.23244000
C	2.79257100	7.94403000	-7.09976600
H	2.79887700	8.40115300	-8.09583600
H	1.98812900	8.39092900	-6.51372000
H	3.75031800	8.16877600	-6.61750200
C	1.25035500	6.06036400	-7.84526600
H	0.42764200	6.49042300	-7.27210400
H	1.19851400	6.43692400	-8.87321300
H	1.13044300	4.97177600	-7.87648000
C	3.75166500	5.80788200	-8.03507000
H	4.71620000	6.03007000	-7.56713100
H	3.63903800	4.72033700	-8.09199000
H	3.75942900	6.20981900	-9.05380700

UPBEPBE/6-311+G(d,p)-THF(SMD)//UB3LYP/6-31G(d)
HF= -1081.8084662
DLPNO-CCSD(T)/def2-TZVPP-gas//UB3LYP/6-31G(d)
HF= -1081.052994389596

B-E-anti-Z

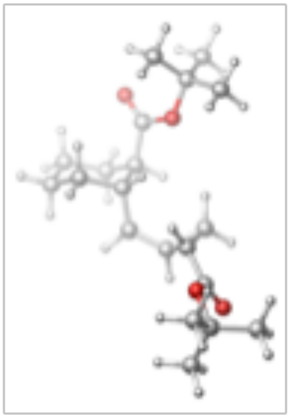

UB3LYP/6-31G(d)
Zero-point correction= 0.503445 (Hartree/Particle)
Thermal correction to Energy= 0.530417
Thermal correction to Enthalpy= 0.531361
Thermal correction to Gibbs Free Energy= 0.444035
Sum of electronic and zero-point Energies= -1082.376369
Sum of electronic and thermal Energies= -1082.349397
Sum of electronic and thermal Enthalpies= -1082.348453
Sum of electronic and thermal Free Energies= -1082.435779

C	-0.17804700	0.62844600	-0.94558600
C	-1.06000500	3.41535200	-0.27329400
C	-2.03893800	2.22702600	-0.28348500
C	-1.36346200	0.87565800	-0.00216700
H	-0.54550100	0.49312800	-1.97283700
H	0.33166000	-0.30615400	-0.67890800
H	-1.57461600	4.30482300	-0.65713200
H	-2.83609800	2.40577300	0.44631200
H	-2.51221600	2.19753500	-1.27414800
H	-1.01712100	0.86102100	1.03729700
H	-2.10178500	0.06991100	-0.10355500
C	-0.61387000	3.78482100	1.14036000
O	0.09523600	4.93723800	1.11574400
O	-0.86896400	3.15146900	2.14550700
C	0.65198100	5.54241700	2.34245300
C	-0.48579500	5.93754900	3.28985300
H	-1.01307400	5.05861100	3.66377100
H	-0.07646000	6.49069800	4.14275400
H	-1.20106300	6.58882800	2.77559200
C	1.36905000	6.78425000	1.80722400
H	0.66488100	7.44764400	1.29458100
H	1.82849500	7.33882800	2.63225200
H	2.15565300	6.50283500	1.09937800
C	1.64870600	4.58078600	2.99835000

H	2.41524000	4.27593300	2.27719300
H	2.15064900	5.08630400	3.83114900
H	1.14709800	3.69042300	3.37984900
C	0.82371700	1.79238300	-0.89786600
H	1.26328400	1.83686700	0.10889800
H	1.65322900	1.61467600	-1.59367700
C	0.17955500	3.16219500	-1.21706100
H	0.91301500	3.94272300	-0.97675800
C	-0.20403100	3.30005100	-2.66026000
H	-0.47676300	2.41196300	-3.22332800
C	-0.23044900	4.57096400	-3.37895300
C	-0.13869100	5.91940600	-2.71771500
C	1.06766300	5.45671200	-3.48778400
H	-0.77853600	4.56913400	-4.31810000
H	-0.04317300	5.96476400	-1.63678600
H	-0.71170200	6.72326900	-3.17058200
H	1.93508200	5.11033200	-2.93728900
C	1.33047500	6.05918300	-4.81825400
O	0.46499300	6.54749900	-5.52506400
O	2.64153100	5.96962200	-5.12781800
C	3.17171400	6.47413000	-6.40777200
C	2.94817100	7.98710100	-6.50782600
H	3.45604500	8.37453400	-7.39836500
H	1.88580100	8.22542500	-6.57773600
H	3.36824400	8.49226500	-5.63104100
C	2.53487300	5.71394300	-7.57619300
H	1.46824100	5.93026600	-7.65273500
H	3.02210500	6.00718700	-8.51308200
H	2.67015300	4.63442900	-7.44695300
C	4.66453500	6.15232500	-6.29967800
H	5.10840200	6.66127900	-5.43789300
H	4.82027300	5.07513100	-6.18104600
H	5.18720400	6.48140300	-7.20406900

UPBEPBE/6-311+G(d,p)-THF(SMD)//UB3LYP/6-31G(d)
HF= -1081.8132932
DLPNO-CCSD(T)/def2-TZVPP-gas//UB3LYP/6-31G(d)
HF= -1081.057123843382

C-E-anti-E-TS

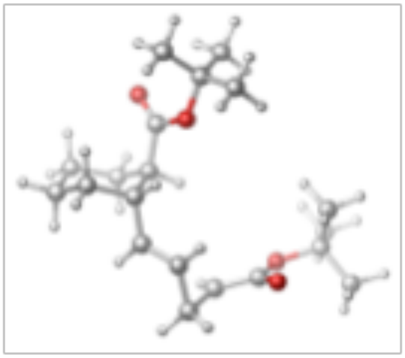

Imaginary frequency= -491.13
UB3LYP/6-31G(d)
Zero-point correction= 0.502582 (Hartree/Particle)
Thermal correction to Energy= 0.529195
Thermal correction to Enthalpy= 0.530139
Thermal correction to Gibbs Free Energy= 0.443422
Sum of electronic and zero-point Energies= -1082.372958
Sum of electronic and thermal Energies= -1082.346345
Sum of electronic and thermal Enthalpies= -1082.345401
Sum of electronic and thermal Free Energies= -1082.432118

C	-2.19998900	1.67479200	-0.30825400
C	-0.60101000	4.08650900	-1.08132900
C	-2.14034700	4.11026600	-1.04855100
C	-2.73952400	3.09173600	-0.06459200
H	-2.57440800	1.29543700	-1.26997200
H	-2.58183200	0.98796400	0.45774500
H	-0.24401400	4.70302900	-1.91687000
H	-2.48255100	5.11909800	-0.79501800
H	-2.50400800	3.89645900	-2.06288300
H	-2.49927600	3.40551600	0.95745500
H	-3.83365300	3.09956900	-0.15012200
C	0.00209300	4.72301700	0.17397300
O	1.34771200	4.76113800	0.07646500
O	-0.63882200	5.15601000	1.11163400
C	2.19860300	5.33208300	1.13934100
C	1.88508200	6.82144000	1.31590000
H	0.87726300	6.97041400	1.70623300
H	2.60259300	7.26583000	2.01495900
H	1.97613800	7.34556800	0.35809700
C	3.60852200	5.13649600	0.57622500
H	3.71921800	5.65969800	-0.37898000
H	4.35251700	5.53074800	1.27646400
H	3.81616000	4.07408000	0.41223800
C	2.01631100	4.54065600	2.43870500
H	2.19783000	3.47422600	2.26425200
H	2.74031000	4.88905500	3.18398700

H	1.01048400	4.66743500	2.84216400
C	-0.66349100	1.65076100	-0.30106200
H	-0.30879100	1.91556300	0.70549600
H	-0.29431700	0.63819900	-0.50567400
C	-0.03760900	2.63649800	-1.31708300
H	1.04144700	2.68616400	-1.13549900
C	-0.23649300	2.20067400	-2.74227500
H	-1.22489500	1.86533000	-3.05285200
C	0.75952800	2.31676700	-3.71271700
C	0.59893200	1.98313200	-5.15790900
C	0.44048400	3.46069900	-5.14617900
H	1.77248800	2.55707200	-3.40015800
H	-0.29427100	1.41491800	-5.41727000
H	1.49719300	1.62369500	-5.65679100
H	-0.54752900	3.89903600	-5.08976300
C	1.58750600	4.33009600	-5.38730600
O	2.74147100	3.92967500	-5.48751400
O	1.19657400	5.62843300	-5.46049600
C	2.16571100	6.71180700	-5.67627600
C	2.86005800	6.53701400	-7.03209500
H	3.49034500	7.40972400	-7.23821100
H	3.48388100	5.64195900	-7.04175400
H	2.11572000	6.45907200	-7.83229800
C	3.16677600	6.76242400	-4.51558900
H	3.79750900	5.87237000	-4.50190400
H	3.80565300	7.64715000	-4.61835200
H	2.63592900	6.83621000	-3.55966600
C	1.27875200	7.96001100	-5.67962900
H	0.53437400	7.90267100	-6.48048800
H	0.75040400	8.06141600	-4.72595100
H	1.88857900	8.85628600	-5.83579100

UPBEPBE/6-311+G(d,p)-THF(SMD)//UB3LYP/6-31G(d)
HF= -1081.8117299
DLPNO-CCSD(T)/def2-TZVPP-gas//UB3LYP/6-31G(d)
HF= -1081.050146193693

C-E-anti-Z-TS

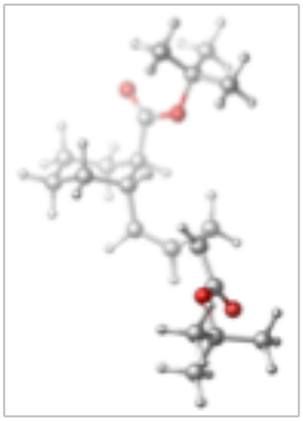

Imaginary frequency= -487.75
UB3LYP/6-31G(d)
Zero-point correction= 0.502622 (Hartree/Particle)
Thermal correction to Energy= 0.529238
Thermal correction to Enthalpy= 0.530182
Thermal correction to Gibbs Free Energy= 0.443370
Sum of electronic and zero-point Energies= -1082.371671
Sum of electronic and thermal Energies= -1082.345055
Sum of electronic and thermal Enthalpies= -1082.344111
Sum of electronic and thermal Free Energies= -1082.430923

C	-1.83023800	1.53109700	-0.48993200
C	-0.32373600	4.11729700	-0.65810900
C	-1.84701100	4.04227900	-0.87196300
C	-2.50899300	2.86291200	-0.14195400
H	-2.01788900	1.28640700	-1.54536300
H	-2.27227500	0.71451300	0.09485000
H	0.09658900	4.85989500	-1.34762300
H	-2.30547300	4.98478200	-0.55369000
H	-2.02525100	3.94812500	-1.95173800
H	-2.45160900	3.03564300	0.93848100
H	-3.57435400	2.82336600	-0.40239500
C	0.03997600	4.61662700	0.74032400
O	1.37102100	4.84477900	0.81687700
O	-0.74796600	4.80288500	1.64610300
C	2.01138100	5.35028100	2.04863800
C	1.46059400	6.73933000	2.38830800
H	0.40476700	6.69104900	2.65883200
H	2.02085300	7.15857600	3.23165000
H	1.57834300	7.41549700	1.53436400
C	3.48676200	5.43431700	1.64996400
H	3.62063200	6.11144900	0.80005600
H	4.08177200	5.81072800	2.48875200
H	3.87053400	4.44778700	1.36992600
C	1.81446200	4.34924300	3.19176800

H	2.17228200	3.35692800	2.89515400
H	2.39561300	4.67119800	4.06324100
H	0.76453200	4.27652500	3.47881300
C	-0.31741800	1.59088200	-0.22678100
H	-0.15244100	1.72056500	0.85214800
H	0.15879300	0.64156900	-0.50288100
C	0.38402500	2.74749600	-0.97554300
H	1.40603900	2.82936800	-0.58648200
C	0.46218900	2.51009700	-2.46053000
H	-0.28502900	1.86666900	-2.91864000
C	1.44562600	3.03990400	-3.29820400
C	2.57033600	3.93694500	-2.88998000
C	3.23277600	2.60849300	-2.95154000
H	1.34755400	2.88993000	-4.36902000
H	2.50075300	4.38731400	-1.90110400
H	2.89663300	4.63681900	-3.65748100
H	3.31894800	2.00007100	-2.06029500
C	3.92386100	2.18640800	-4.16510200
O	3.89925800	2.81107100	-5.21955200
O	4.57590400	1.01287800	-3.96235300
C	5.35241300	0.37087900	-5.03262700
C	6.51009500	1.28024300	-5.46141200
H	7.14930900	0.74984200	-6.17648700
H	6.13965000	2.19289800	-5.93073000
H	7.12247000	1.55074000	-4.59398600
C	4.43558700	0.00841300	-6.20694100
H	4.05007800	0.90467800	-6.69498700
H	4.99497800	-0.58208100	-6.94166000
H	3.59177200	-0.59630000	-5.85664900
C	5.88185200	-0.89348600	-4.34997500
H	6.49641100	-0.63498200	-3.48146400
H	5.05411700	-1.52443200	-4.00976100
H	6.49490100	-1.47224100	-5.04912100

UPBEPBE/6-311+G(d,p)-THF(SMD)//UB3LYP/6-31G(d)
HF= -1081.8098557
DLPNO-CCSD(T)/def2-TZVPP-gas//UB3LYP/6-31G(d)
HF= -1081.049813671432

D-E-anti-E

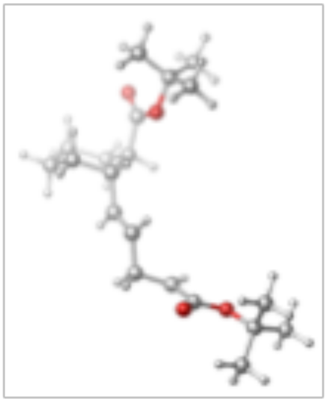

UB3LYP/6-31G(d)

Zero-point correction= 0.503213 (Hartree/Particle)
Thermal correction to Energy= 0.530544
Thermal correction to Enthalpy= 0.531488
Thermal correction to Gibbs Free Energy= 0.441752
Sum of electronic and zero-point Energies= -1082.388249
Sum of electronic and thermal Energies= -1082.360918
Sum of electronic and thermal Enthalpies= -1082.359974
Sum of electronic and thermal Free Energies= -1082.449710

C	-1.80953300	1.16780600	-0.22870100
C	-0.92863100	4.02626200	-0.17306000
C	-2.41709600	3.63671300	-0.08629300
C	-2.63490800	2.23452100	0.50611200
H	-2.19428100	1.03916700	-1.25037000
H	-1.92494800	0.19450700	0.26493800
H	-0.83120900	4.95710500	-0.74926300
H	-2.95157000	4.38161700	0.51182900
H	-2.84233200	3.67658200	-1.09805600
H	-2.35306500	2.25108300	1.56537500
H	-3.70210900	1.98147100	0.46717100
C	-0.36263700	4.35951300	1.21240400
O	0.94435300	4.68273100	1.12500800
O	-1.01119600	4.36618000	2.24025600
C	1.73800200	5.07363200	2.30723100
C	1.16678200	6.35746000	2.91836300
H	0.17232300	6.18864900	3.33403100
H	1.82835100	6.70733200	3.71894000
H	1.10612200	7.14608000	2.16027300
C	3.12380700	5.32645400	1.70840300
H	3.07978500	6.11623100	0.95151100
H	3.82266900	5.63615900	2.49262500
H	3.51342700	4.41857500	1.23639800
C	1.78274700	3.91896200	3.31343000

H	2.14606900	3.00574700	2.82892400
H	2.47421100	4.16782600	4.12645700
H	0.79684000	3.72629700	3.73891800
C	-0.32160700	1.54889200	-0.27741900
H	0.07717100	1.55500800	0.74769600
H	0.25133900	0.79563700	-0.83178500
C	-0.07293200	2.94208800	-0.90794400
H	0.98017700	3.20239700	-0.76273600
C	-0.33677800	2.95001300	-2.39492600
H	-1.34536200	2.70009700	-2.72711900
C	0.58834200	3.22708300	-3.31887200
C	0.35038700	3.23178600	-4.81678200
C	0.51994600	4.60154800	-5.38813400
H	1.60163200	3.48324200	-3.00690100
H	-0.66676500	2.86737300	-5.01930600
H	1.05981700	2.55837600	-5.31150600
H	-0.19443800	5.38269200	-5.14653900
C	1.67499000	4.94662900	-6.20028000
O	2.57133800	4.15675800	-6.48099500
O	1.61990200	6.24404600	-6.59591400
C	2.67674800	6.84265500	-7.42526600
C	2.76693600	6.11674000	-8.77264600
H	3.46597100	6.64750400	-9.42905900
H	3.11485100	5.09052600	-8.64583300
H	1.78680600	6.10140600	-9.26190800
C	4.00930900	6.82684400	-6.66702900
H	4.36339600	5.80639600	-6.51269600
H	4.76423300	7.37839400	-7.23887400
H	3.89761000	7.31522000	-5.69260700
C	2.17899900	8.27799400	-7.61623000
H	1.20663100	8.28559600	-8.11949600
H	2.07020800	8.78101600	-6.64990000
H	2.88948900	8.84647600	-8.22563700

UPBEPBE/6-311+G(d,p)-THF(SMD)//UB3LYP/6-31G(d)
HF= -1081.8214519
DLPNO-CCSD(T)/def2-TZVPP-gas//UB3LYP/6-31G(d)
HF= -1081.065328112647

D-E-anti-Z

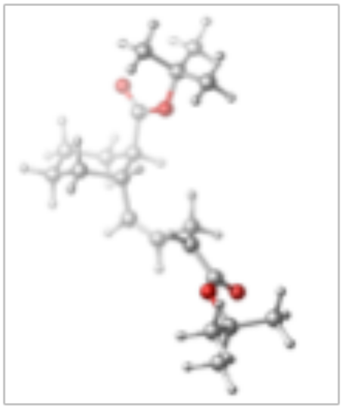

UB3LYP/6-31G(d)
Zero-point correction= 0.503469 (Hartree/Particle)
Thermal correction to Energy= 0.530766
Thermal correction to Enthalpy= 0.531710
Thermal correction to Gibbs Free Energy= 0.440658
Sum of electronic and zero-point Energies= -1082.385399
Sum of electronic and thermal Energies= -1082.358102
Sum of electronic and thermal Enthalpies= -1082.357157
Sum of electronic and thermal Free Energies= -1082.448209

C	-2.18861500	1.77260700	-0.15725400
C	-0.56909700	4.26393400	-0.53085500
C	-2.10421800	4.26618200	-0.65628400
C	-2.77809700	3.15363600	0.16346600
H	-2.46033300	1.48365800	-1.18238700
H	-2.62889700	1.01117600	0.49901900
H	-0.14682400	4.96383800	-1.26395000
H	-2.49220200	5.24311700	-0.34919600
H	-2.35665800	4.14314700	-1.71806800
H	-2.64756700	3.37159800	1.22939500
H	-3.85808100	3.15780400	-0.03155900
C	-0.11236500	4.79042000	0.83255300
O	1.23592600	4.86946100	0.87510800
O	-0.85413200	5.11789100	1.73753100
C	1.95467800	5.37228600	2.06323800
C	1.56969400	6.83189500	2.32637800
H	0.52366200	6.91739100	2.62414000
H	2.19794600	7.23736500	3.12757700
H	1.73435200	7.43673700	1.42787800
C	3.42080400	5.26637000	1.63582900
H	3.60996900	5.87608500	0.74640600
H	4.07415400	5.61707700	2.44169100
H	3.68341600	4.22851000	1.40564400
C	1.67391500	4.46911200	3.26873300

H	1.90912900	3.42653800	3.02704500
H	2.30872500	4.77287100	4.10877200
H	0.62963300	4.53419300	3.57777500
C	-0.65969500	1.76383500	0.00092200
H	-0.41236500	1.92371200	1.06032800
H	-0.25246000	0.78117600	-0.26862400
C	0.04912600	2.85667900	-0.83705100
H	1.09561200	2.89254600	-0.52384700
C	0.00315600	2.54938300	-2.31656600
H	-0.95298000	2.20114400	-2.70606400
C	1.00481500	2.67136900	-3.19745000
C	2.42298800	3.14770300	-2.92936200
C	3.38567000	2.01460100	-3.07495100
H	0.81981500	2.39198500	-4.23405700
H	2.50059500	3.58370200	-1.92662600
H	2.68301000	3.92279900	-3.65909900
H	3.44052200	1.24479500	-2.31135700
C	4.17789400	1.84981800	-4.28195400
O	4.14180500	2.62686900	-5.23138100
O	4.94848300	0.73437900	-4.20930300
C	5.83534100	0.33681000	-5.31267600
C	6.89987100	1.41457000	-5.54927800
H	7.62789400	1.05546600	-6.28564900
H	6.45146900	2.33744400	-5.92014500
H	7.43604100	1.62956200	-4.61835600
C	5.00996700	0.05045700	-6.57281300
H	4.54136700	0.95973900	-6.95233900
H	5.66058800	-0.36249200	-7.35214900
H	4.22949800	-0.68726900	-6.35597900
C	6.47506000	-0.94872500	-4.78120100
H	7.02879200	-0.75173500	-3.85739000
H	5.70946100	-1.70213600	-4.56891900
H	7.17000200	-1.35892100	-5.52159400

UPBEPBE/6-311+G(d,p)-THF(SMD)//UB3LYP/6-31G(d)
HF= -1081.8191917
DLPNO-CCSD(T)/def2-TZVPP-gas//UB3LYP/6-31G(d)
HF= -1081.064106342332

A-E-syn-TS

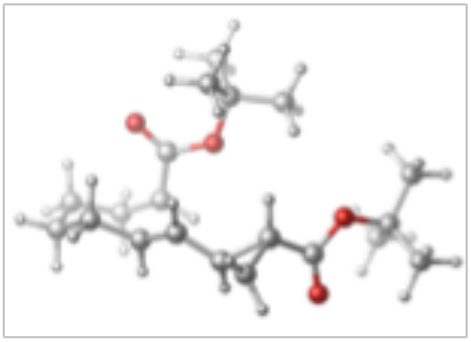

Imaginary frequency= -417.77
UB3LYP/6-31G(d)
Zero-point correction= 0.500930 (Hartree/Particle)
Thermal correction to Energy= 0.527870
Thermal correction to Enthalpy= 0.528814
Thermal correction to Gibbs Free Energy= 0.442610
Sum of electronic and zero-point Energies= -1082.347722
Sum of electronic and thermal Energies= -1082.320783
Sum of electronic and thermal Enthalpies= -1082.319838
Sum of electronic and thermal Free Energies= -1082.406042

C	-1.92163300	2.30611500	-0.93664500
C	-0.13412100	4.81288100	-0.20040000
C	-1.60509900	4.82878100	-0.54162300
C	-2.40380200	3.55902600	-0.19663800
H	-2.07896300	2.43129800	-2.01923300
H	-2.54634300	1.45526100	-0.63563500
H	0.49879200	5.49253400	-0.76319000
H	-2.07333800	5.68403700	-0.02560800
H	-1.70500000	5.04066400	-1.61534200
H	-2.35225500	3.38982400	0.88225400
H	-3.45730200	3.73911400	-0.44872200
C	0.30731600	4.58370400	1.17572800
O	1.52612900	5.15121900	1.39118700
O	-0.31345700	3.94823600	2.02257400
C	2.18798400	5.07848300	2.70086800
C	1.30834100	5.70896500	3.78847100
H	0.41035100	5.11725300	3.96810400
H	1.87899300	5.78181800	4.72155300
H	1.01149600	6.72179400	3.49395200
C	3.44937200	5.92083500	2.48919500
H	3.18111400	6.95867400	2.26343900
H	4.06525600	5.91256800	3.39523800
H	4.04584600	5.53720600	1.65629900
C	2.54935400	3.62436900	3.02525600
H	3.16591100	3.19737400	2.22610600
H	3.12628000	3.58331900	3.95637600

H	1.65067300	3.01649000	3.14294500
C	-0.44862400	1.94452600	-0.68854700
H	-0.26579500	1.83969900	0.38824900
H	-0.25425100	0.95867800	-1.13601600
C	0.53793700	2.93236700	-1.28598200
H	0.21148500	3.42294200	-2.20447900
C	1.89600800	2.81532700	-1.10443200
H	2.27063400	2.19021900	-0.29284000
C	2.89631300	3.50691900	-1.92920500
C	4.21670500	3.99653100	-1.30430500
C	4.20520400	2.84211300	-2.27617500
H	2.50685600	4.17125000	-2.69705200
H	4.37844600	3.78145000	-0.25401100
H	4.60967700	3.03500200	-3.26504800
H	4.39978100	1.85179100	-1.87389800
C	4.74456500	5.30004600	-1.79466800
O	4.61731500	5.69126700	-2.94120100
O	5.37452600	5.96158600	-0.80302300
C	5.99491500	7.28421500	-1.02628100
C	7.10275000	7.17254300	-2.07919000
H	6.69350200	6.93291400	-3.06152300
H	7.64117200	8.12485500	-2.14426400
H	7.82140700	6.39612000	-1.79450700
C	6.58607400	7.61329000	0.34668100
H	7.08821700	8.58600900	0.31515700
H	5.80115100	7.65292100	1.10851200
H	7.31785200	6.85523800	0.64432800
C	4.91912200	8.30332800	-1.41554800
H	4.11880700	8.31587200	-0.66771300
H	5.36168600	9.30510000	-1.45703000
H	4.48854100	8.07012700	-2.39060300

UPBEPBE/6-311+G(d,p)-THF(SMD)//UB3LYP/6-31G(d)
HF= -1081.7842317
DLPNO-CCSD(T)/def2-TZVPP-gas//UB3LYP/6-31G(d)
HF= -1081.019169648369

B-E-syn

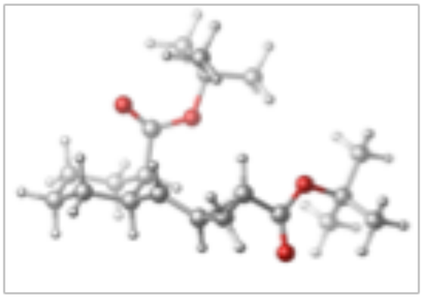

UB3LYP/6-31G(d)
Zero-point correction= 0.503580 (Hartree/Particle)
Thermal correction to Energy= 0.530424
Thermal correction to Enthalpy= 0.531368
Thermal correction to Gibbs Free Energy= 0.445156
Sum of electronic and zero-point Energies= -1082.379006
Sum of electronic and thermal Energies= -1082.352162
Sum of electronic and thermal Enthalpies= -1082.351217
Sum of electronic and thermal Free Energies= -1082.437429

C	-2.03221500	2.65079500	-1.06917500
C	0.20354900	4.50346400	-0.29989300
C	-1.27487700	4.96281500	-0.36653900
C	-2.28204700	3.83072700	-0.11966100
H	-2.23769200	2.96509200	-2.10410200
H	-2.73086000	1.83362200	-0.84920700
H	0.83813500	5.31661800	-0.66395700
H	-1.43777400	5.78698300	0.34019900
H	-1.43917900	5.37949500	-1.37070400
H	-2.19807000	3.48787900	0.91742000
H	-3.30122100	4.21627200	-0.25228400
C	0.63211200	4.22262100	1.13546500
O	1.62517500	5.05902000	1.50233400
O	0.14289300	3.36983000	1.85448200
C	2.21791100	5.03053600	2.85393600
C	1.15086400	5.37477200	3.89840200
H	0.38718400	4.59748600	3.95495300
H	1.62149900	5.47816200	4.88273300
H	0.67035800	6.32779200	3.65108000
C	3.27709000	6.13278800	2.77030400
H	2.80816100	7.10246300	2.57205200
H	3.82731300	6.20091800	3.71491400
H	3.98699300	5.92419600	1.96361200
C	2.87005400	3.66933300	3.11903800
H	3.59498500	3.43334700	2.33196200
H	3.40794900	3.70203700	4.07330800
H	2.12424000	2.87451100	3.16354400

C	-0.58703900	2.14082500	-0.97119200
H	-0.41360500	1.71991200	0.02600600
H	-0.41996100	1.33411500	-1.69609000
C	0.44285500	3.26046900	-1.23240200
H	0.26590700	3.64633800	-2.24973400
C	1.85297800	2.75780300	-1.16451300
H	2.10484400	2.03527200	-0.38901400
C	2.93436700	3.32826300	-1.95706300
C	4.21841400	3.89606200	-1.25023000
C	4.26910000	2.65130100	-2.09543800
H	2.63968300	3.93794500	-2.80897500
H	4.25939500	3.79568300	-0.17197200
H	4.77339000	2.73974900	-3.05306200
H	4.41352400	1.70738000	-1.57645100
C	4.77610400	5.14854000	-1.81918200
O	4.80219400	5.39688100	-3.01264800
O	5.23193700	5.95948200	-0.83898000
C	5.83305200	7.27298700	-1.14510300
C	7.08363300	7.08885000	-2.01184300
H	6.82647200	6.70278100	-2.99902500
H	7.59127200	8.05291100	-2.13053600
H	7.78169700	6.39538300	-1.52987800
C	6.21069000	7.80292600	0.24025300
H	6.68707000	8.78487200	0.14948500
H	5.32291100	7.90657000	0.87229400
H	6.91090200	7.12300200	0.73649700
C	4.79124200	8.17999100	-1.80855600
H	3.89210700	8.24349700	-1.18581900
H	5.20089200	9.19065400	-1.91794000
H	4.51312000	7.80546900	-2.79481000

UPBEPBE/6-311+G(d,p)-THF(SMD)//UB3LYP/6-31G(d)
HF= -1081.8148778
DLPNO-CCSD(T)/def2-TZVPP-gas//UB3LYP/6-31G(d)
HF= -1081.059707156171

ROT-E-syn-TS

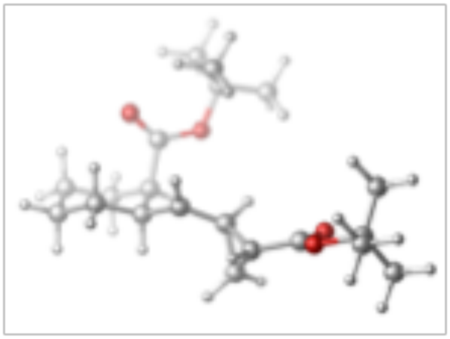

Imaginary frequency= -12.37
UB3LYP/6-31G(d)
Zero-point correction= 0.502965 (Hartree/Particle)
Thermal correction to Energy= 0.529217
Thermal correction to Enthalpy= 0.530161
Thermal correction to Gibbs Free Energy= 0.444902
Sum of electronic and zero-point Energies= -1082.374309
Sum of electronic and thermal Energies= -1082.348057
Sum of electronic and thermal Enthalpies= -1082.347113
Sum of electronic and thermal Free Energies= -1082.432373

C	-2.06215100	2.42475000	-1.00123200
C	0.11118100	4.41371000	-0.39961200
C	-1.37287500	4.82384100	-0.55577200
C	-2.36908200	3.70587700	-0.21361500
H	-2.23662900	2.60209400	-2.07373000
H	-2.75024000	1.62358200	-0.70313400
H	0.73953700	5.18958300	-0.84691000
H	-1.57505500	5.71564500	0.05125100
H	-1.51496300	5.12404900	-1.60397700
H	-2.32061800	3.49442200	0.85948000
H	-3.38830000	4.05011200	-0.43218200
C	0.52594600	4.30221100	1.06281400
O	1.66383800	4.99532300	1.28234200
O	-0.07681100	3.67161500	1.91222200
C	2.30395400	5.05425000	2.61234300
C	1.35884700	5.72540300	3.61483500
H	0.47382000	5.11353200	3.79556200
H	1.88244400	5.87700300	4.56554500
H	1.04299600	6.70610800	3.24226300
C	3.53113000	5.93147700	2.35167400
H	3.22997100	6.91917000	1.98800200
H	4.10353100	6.06207400	3.27629500
H	4.18439000	5.47383900	1.60164600
C	2.72374500	3.64961300	3.05823700
H	3.35558800	3.18084600	2.29588200
H	3.30612400	3.71998600	3.98392700

H	1.85485200	3.01478600	3.23786100
C	-0.61009600	1.97101500	-0.79398600
H	-0.45949000	1.69304400	0.25662000
H	-0.40117400	1.07689100	-1.39550400
C	0.39850700	3.07212000	-1.17443100
H	0.22972000	3.33595400	-2.23212900
C	1.82003700	2.61994700	-1.02447900
H	2.04000700	1.85861600	-0.27631500
C	2.95720500	3.37114000	-1.60175700
C	4.01590800	2.64042700	-2.43046400
C	3.06880900	3.64311800	-3.07102100
H	3.38922600	4.15332200	-0.97180500
H	3.85130300	1.58840400	-2.63693600
H	3.50524200	4.58740500	-3.38172500
H	2.31285200	3.23406400	-3.73577800
C	5.43291600	3.05480000	-2.24178500
O	5.77895600	4.19921500	-2.00545200
O	6.25708100	1.99390600	-2.36186000
C	7.71902100	2.12579200	-2.21529800
C	8.27199400	3.05605700	-3.30031000
H	7.92261500	4.07977800	-3.15803400
H	9.36738800	3.04914400	-3.26582700
H	7.96155700	2.70979200	-4.29226800
C	8.20497500	0.69023800	-2.43079600
H	9.29603900	0.64451300	-2.34744900
H	7.77135500	0.01882200	-1.68257200
H	7.91762000	0.32984900	-3.42389100
C	8.06207900	2.61059600	-0.80266800
H	7.61017700	1.95078700	-0.05378000
H	9.14874400	2.58963200	-0.66179900
H	7.70608800	3.62873200	-0.63832000

UPBEPBE/6-311+G(d,p)-THF(SMD)//UB3LYP/6-31G(d)
HF= -1081.808808
DLPNO-CCSD(T)/def2-TZVPP-gas//UB3LYP/6-31G(d)
HF= -1081.054621545049

B-E-syn-Z

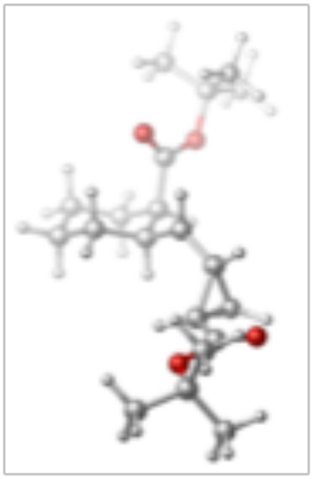

UB3LYP/6-31G(d)
Zero-point correction= 0.503602 (Hartree/Particle)
Thermal correction to Energy= 0.530499
Thermal correction to Enthalpy= 0.531443
Thermal correction to Gibbs Free Energy= 0.443854
Sum of electronic and zero-point Energies= -1082.376986
Sum of electronic and thermal Energies= -1082.350089
Sum of electronic and thermal Enthalpies= -1082.349145
Sum of electronic and thermal Free Energies= -1082.436734

C	-0.26555500	2.07556400	-1.19744900
C	0.88098800	4.58875900	-0.02880900
C	-0.55818800	4.51719100	-0.60665400
C	-1.16570800	3.11140900	-0.50955200
H	-0.22871400	2.29085500	-2.27644100
H	-0.69623600	1.07120800	-1.09617500
H	1.29336600	5.57391700	-0.27094000
H	-1.19138200	5.25659200	-0.10140900
H	-0.50773800	4.81649200	-1.66316800
H	-1.29783200	2.83651100	0.54508500
H	-2.16454500	3.11147600	-0.96425900
C	0.82380800	4.51118000	1.49428200
O	0.63848500	5.74544800	2.00641000
O	0.89323000	3.48932900	2.15189700
C	0.47465800	5.97841300	3.45540300
C	-0.77553300	5.25401200	3.96532400
H	-0.65667400	4.17085300	3.90893400
H	-0.96003100	5.53302800	5.00897000
H	-1.65238800	5.54679100	3.37709100
C	0.29143900	7.49646300	3.52858100
H	-0.58899200	7.80845200	2.95737200
H	0.15868400	7.81001600	4.56941900

H	1.16693900	8.01136600	3.12004600
C	1.74263700	5.54788200	4.20033000
H	2.62166500	6.04020800	3.77019600
H	1.66759800	5.84573900	5.25240200
H	1.88302500	4.46716100	4.15074400
C	1.15875100	2.09121500	-0.62150500
H	1.14128300	1.75040900	0.41848600
H	1.79813800	1.39501700	-1.17910900
C	1.79934400	3.50266600	-0.67335000
H	1.85773600	3.78785000	-1.73516600
C	3.18107300	3.50818300	-0.08909100
H	3.29007700	3.13962100	0.92694900
C	4.40091100	3.86547900	-0.80515800
C	4.68622900	3.31121200	-2.24940200
C	4.45027800	4.77535000	-2.00497400
H	5.30446800	3.88388000	-0.20127600
H	3.89609000	2.72443400	-2.70416400
H	5.31907700	5.42127800	-2.09102200
H	3.51984200	5.19627300	-2.37570300
C	6.07388100	2.85080300	-2.50559700
O	7.06834200	3.44481700	-2.12455700
O	6.06626400	1.68805600	-3.19184300
C	7.31514500	0.99178600	-3.55147900
C	8.15941700	1.87364400	-4.47761600
H	8.51113500	2.76603600	-3.95793500
H	9.02732000	1.30648200	-4.83286600
H	7.57284900	2.17941900	-5.35102600
C	6.80410700	-0.24497700	-4.29500800
H	7.64683900	-0.86304400	-4.62267600
H	6.16295400	-0.84929300	-3.64506500
H	6.22376900	0.04608600	-5.17659400
C	8.07356700	0.58783400	-2.28232900
H	7.42392700	0.00456200	-1.62045700
H	8.93281400	-0.03710300	-2.55129500
H	8.43319800	1.46473700	-1.74180100

UPBEPBE/6-311+G(d,p)-THF(SMD)//UB3LYP/6-31G(d)
HF= -1081.813887
DLPNO-CCSD(T)/def2-TZVPP-gas//UB3LYP/6-31G(d)
HF= -1081.056746864673

C-E-syn-E-TS

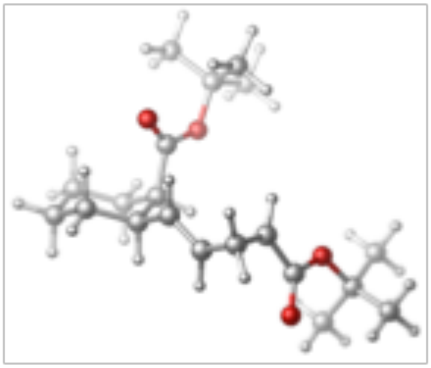

Imaginary frequency= -486.51
UB3LYP/6-31G(d)
Zero-point correction= 0.502544 (Hartree/Particle)
Thermal correction to Energy= 0.529221
Thermal correction to Enthalpy= 0.530165
Thermal correction to Gibbs Free Energy= 0.442990
Sum of electronic and zero-point Energies= -1082.375313
Sum of electronic and thermal Energies= -1082.348636
Sum of electronic and thermal Enthalpies= -1082.347692
Sum of electronic and thermal Free Energies= -1082.434867

C	-1.67019300	3.55800100	-1.18970800
C	0.90504200	4.63556800	-0.09838400
C	-0.40412700	5.46821500	-0.11853600
C	-1.65991900	4.58889100	-0.05169500
H	-1.74982200	4.08645500	-2.15191500
H	-2.55696900	2.91613500	-1.11347100
H	1.74082200	5.31670900	-0.29324200
H	-0.38596300	6.19431900	0.70240300
H	-0.41751200	6.04689300	-1.05267000
H	-1.69589300	4.06640100	0.91406300
H	-2.55514000	5.22170200	-0.10069800
C	1.13708600	4.06873600	1.30059800
O	1.64250200	5.03047800	2.10017800
O	0.88312300	2.93302600	1.65714900
C	1.92963200	4.79481300	3.52944700
C	0.64086300	4.42984900	4.27352300
H	0.25338700	3.46418000	3.94549500
H	0.84215600	4.38081900	5.34963800
H	-0.12523100	5.19543000	4.10772100
C	2.45669300	6.15582500	3.99067400
H	1.69963700	6.93319700	3.84473900
H	2.71536200	6.11899400	5.05422400
H	3.35132900	6.43556700	3.42516100
C	3.00965400	3.71697500	3.67170000
H	3.89684600	3.98869800	3.08905400

H	3.30748800	3.63335500	4.72302900
H	2.64595000	2.74583800	3.33265100
C	-0.40027700	2.69392300	-1.18289300
H	-0.38409600	2.06774700	-0.28488100
H	-0.39940100	2.01927900	-2.04814300
C	0.88936100	3.54913200	-1.22017100
H	0.87839000	4.12078500	-2.16128900
C	2.12803300	2.69992700	-1.21143100
H	2.21977500	1.96710500	-0.41188100
C	3.18819400	2.87156600	-2.10408000
C	4.83879000	3.33039400	-1.40105700
C	4.45018200	2.07613300	-2.09758900
H	3.06057600	3.53484100	-2.95582300
H	4.80855900	3.38082400	-0.32035000
H	4.91645400	1.92969500	-3.07020500
H	4.44061200	1.17645900	-1.48282700
C	5.44975400	4.42762100	-2.14334400
O	5.54838500	4.45860800	-3.36477900
O	5.86777500	5.40516300	-1.29707300
C	6.50842700	6.62969900	-1.79523600
C	7.81518700	6.28577600	-2.51999400
H	7.62137500	5.71609700	-3.43007000
H	8.34268100	7.20892700	-2.78628100
H	8.46861200	5.69757900	-1.86609300
C	6.79360700	7.40639500	-0.50651500
H	7.28303300	8.35833700	-0.73875000
H	5.86339600	7.61632300	0.03164100
H	7.45041900	6.83036800	0.15351100
C	5.53698900	7.40709300	-2.69146000
H	4.59684300	7.59268700	-2.15994800
H	5.97465100	8.37672900	-2.95550600
H	5.32213100	6.85621400	-3.60826300

UPBEPBE/6-311+G(d,p)-THF(SMD)//UB3LYP/6-31G(d)
HF= -1081.8127959
DLPNO-CCSD(T)/def2-TZVPP-gas//UB3LYP/6-31G(d)
HF= -1081.051510885410

C-E-syn-Z-TS

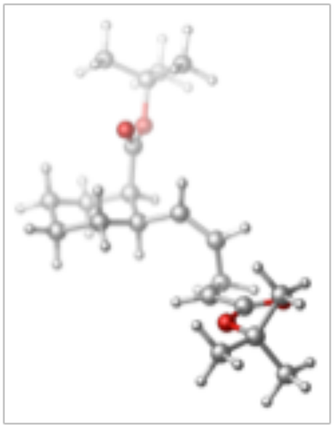

Imaginary frequency= -491.47
UB3LYP/6-31G(d)
Zero-point correction= 0.502733 (Hartree/Particle)
Thermal correction to Energy= 0.529351
Thermal correction to Enthalpy= 0.530295
Thermal correction to Gibbs Free Energy= 0.443676
Sum of electronic and zero-point Energies= -1082.372940
Sum of electronic and thermal Energies= -1082.346322
Sum of electronic and thermal Enthalpies= -1082.345378
Sum of electronic and thermal Free Energies= -1082.431997

C	-0.35681600	2.41281200	-1.48302900
C	0.82108800	4.80183300	-0.10587300
C	-0.67631000	4.72526900	-0.50766300
C	-1.20330900	3.28424200	-0.54443600
H	-0.47462700	2.77717800	-2.51508400
H	-0.72351800	1.37859100	-1.47524500
H	1.15298300	5.83685600	-0.24265600
H	-1.26872700	5.34520600	0.17529400
H	-0.77692700	5.17238600	-1.50672600
H	-1.18398100	2.85586800	0.46670700
H	-2.25234100	3.28493600	-0.86645500
C	0.96690800	4.50184800	1.38440100
O	0.68137700	5.60997500	2.09793800
O	1.25912800	3.42238800	1.86434100
C	0.67050200	5.61423700	3.57498700
C	-0.38723800	4.63570500	4.09582600
H	-0.11791200	3.60288300	3.86988300
H	-0.48188300	4.74413500	5.18219300
H	-1.36286300	4.85519700	3.64802100
C	0.28074600	7.05869600	3.89853500
H	-0.70130000	7.29761400	3.47759300
H	0.23803200	7.20349900	4.98321700
H	1.01303000	7.75876900	3.48365300
C	2.07159200	5.29636500	4.10779900

H	2.80855200	5.97982900	3.67204400
H	2.08787900	5.42870900	5.19556900
H	2.36060000	4.27009400	3.87611700
C	1.12947700	2.44008200	-1.09522600
H	1.26788300	1.95745400	-0.12252500
H	1.71913400	1.87027900	-1.82456500
C	1.68727900	3.88484900	-1.02308400
H	1.57971200	4.31993700	-2.02740500
C	3.13865200	3.90345700	-0.63555100
H	3.38909300	3.41719200	0.30397000
C	4.19640900	4.41832900	-1.39240800
C	4.47354700	3.75085000	-3.10086100
C	4.11581700	5.13608600	-2.69989800
H	5.18485200	4.41801700	-0.94280100
H	3.69842400	3.04779200	-3.37750900
H	4.90164800	5.87104300	-2.86503300
H	3.13341500	5.48616100	-3.01279100
C	5.86902800	3.38421300	-3.31859900
O	6.81835400	4.11511200	-3.05926700
O	5.96064700	2.12850100	-3.82722900
C	7.26136500	1.51268300	-4.12402300
C	7.99346500	2.32052900	-5.20203200
H	8.26482600	3.31070400	-4.83306400
H	8.90560600	1.79221000	-5.50251900
H	7.35878100	2.43366200	-6.08797900
C	6.86460600	0.13314700	-4.65769400
H	7.75879900	-0.44237700	-4.91979000
H	6.30121400	-0.42502300	-3.90279200
H	6.23944400	0.23001400	-5.55136800
C	8.08796400	1.37816100	-2.83959500
H	7.51607800	0.84073800	-2.07497100
H	8.99953400	0.80588000	-3.04688200
H	8.36823400	2.35714300	-2.44810700

UPBEPBE/6-311+G(d,p)-THF(SMD)//UB3LYP/6-31G(d)
HF= -1081.8108939
DLPNO-CCSD(T)/def2-TZVPP-gas//UB3LYP/6-31G(d)
HF= -1081.049757600660

D-E-syn-E

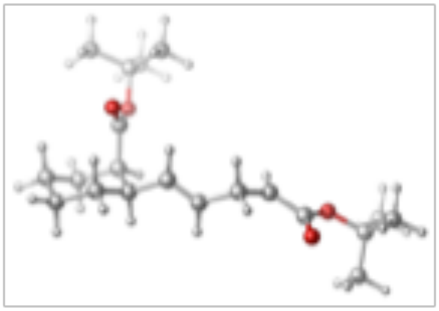

UB3LYP/6-31G(d)
Zero-point correction= 0.503178 (Hartree/Particle)
Thermal correction to Energy= 0.530519
Thermal correction to Enthalpy= 0.531463
Thermal correction to Gibbs Free Energy= 0.442387
Sum of electronic and zero-point Energies= -1082.390879
Sum of electronic and thermal Energies= -1082.363539
Sum of electronic and thermal Enthalpies= -1082.362595
Sum of electronic and thermal Free Energies= -1082.451670

C	-1.98004700	3.11709800	-1.02654400
C	0.07241700	4.67281500	0.51129400
C	-1.44106000	5.01697600	0.55440800
C	-2.33175000	3.79170600	0.30682000
H	-2.21547700	3.80675500	-1.85154100
H	-2.60358600	2.22695900	-1.17797400
H	0.63293900	5.61329500	0.54527700
H	-1.68038900	5.49234000	1.51308300
H	-1.63326700	5.76664900	-0.22592900
H	-2.20582700	3.07028800	1.12516900
H	-3.38577300	4.09727000	0.31443300
C	0.45402500	3.90354800	1.77337400
O	0.70738200	4.77199300	2.77449600
O	0.49287300	2.69152700	1.87959200
C	1.04195000	4.32236200	4.14048100
C	-0.12236400	3.51990100	4.73048900
H	-0.26733500	2.57987300	4.19599200
H	0.08422800	3.29649600	5.78330800
H	-1.04921200	4.10245200	4.68443600
C	1.22459700	5.64469200	4.88985900
H	0.30330500	6.23558900	4.86272300
H	1.48228900	5.45167300	5.93658700
H	2.02684100	6.23733900	4.43849100
C	2.34950800	3.52344400	4.12160300
H	3.14400400	4.10562900	3.64200000
H	2.66107300	3.30698600	5.14973000
H	2.22931200	2.58067400	3.58595100

C	-0.49484600	2.73065300	-1.08955700
H	-0.28697900	1.94453300	-0.35623500
H	-0.25180100	2.32371100	-2.07899100
C	0.43327800	3.93804400	-0.81231400
H	0.25442800	4.67817400	-1.60726100
C	1.88698300	3.54225200	-0.87081800
H	2.20442500	2.79341300	-0.14425200
C	2.77347600	4.01795700	-1.75006100
C	5.15792500	4.73945600	-1.57499300
C	4.22676900	3.59966500	-1.82593200
H	2.45973500	4.76443700	-2.48162300
H	5.07688000	5.31337300	-0.65674400
H	4.45854700	3.17746900	-2.81115500
H	4.40498000	2.80796300	-1.08085400
C	6.17347900	5.12204100	-2.54215300
O	6.34314100	4.55454600	-3.61678200
O	6.89761500	6.17899500	-2.09116600
C	7.99627800	6.75321400	-2.88146500
C	9.09695200	5.70637500	-3.09028200
H	8.74431300	4.88337400	-3.71367900
H	9.96041900	6.17303000	-3.57785300
H	9.42705300	5.30496100	-2.12578700
C	8.49406600	7.89073800	-1.98537200
H	9.33219300	8.40903700	-2.46325300
H	7.69534900	8.61716000	-1.80295300
H	8.83197000	7.50149600	-1.01940200
C	7.46092100	7.30389700	-4.20858300
H	6.64221800	8.00850400	-4.02514300
H	8.26026200	7.84068000	-4.73217900
H	7.09813300	6.50061800	-4.85161400

UPBEPBE/6-311+G(d,p)-THF(SMD)//UB3LYP/6-31G(d)
HF= -1081.8228257
DLPNO-CCSD(T)/def2-TZVPP-gas//UB3LYP/6-31G(d)
HF= -1081.066899666632

D-E-syn-Z

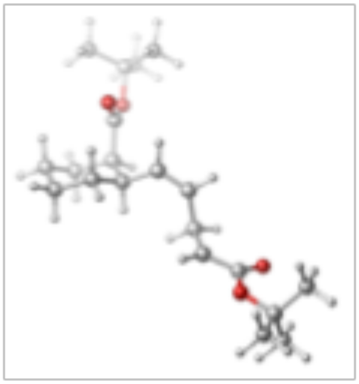

UB3LYP/6-31G(d)
Zero-point correction= 0.503546 (Hartree/Particle)
Thermal correction to Energy= 0.530792
Thermal correction to Enthalpy= 0.531736
Thermal correction to Gibbs Free Energy= 0.442184
Sum of electronic and zero-point Energies= -1082.387779
Sum of electronic and thermal Energies= -1082.360534
Sum of electronic and thermal Enthalpies= -1082.359590
Sum of electronic and thermal Free Energies= -1082.449142

C	-0.89889900	2.50722000	-1.20085300
C	0.69034200	4.70253800	0.08507700
C	-0.74422100	4.92324600	-0.46669800
C	-1.59101600	3.64359500	-0.43481700
H	-0.83889400	2.77656100	-2.26654400
H	-1.49954200	1.59057800	-1.14538500
H	1.26951900	5.61209100	-0.10683000
H	-1.22959000	5.73204600	0.09242400
H	-0.65187100	5.27014600	-1.50557700
H	-1.75557100	3.33470000	0.60590700
H	-2.57991700	3.84728300	-0.86479800
C	0.62880800	4.54758600	1.60277500
O	0.63548500	5.76471600	2.18347000
O	0.54569100	3.49171800	2.20300300
C	0.51361500	5.93977900	3.64509500
C	-0.83023800	5.38580100	4.12971100
H	-0.87721400	4.30200100	4.01308200
H	-0.96577700	5.63092300	5.18917600
H	-1.65487100	5.84054600	3.56955200
C	0.56234400	7.46169700	3.80106500
H	-0.26182700	7.93305100	3.25582400
H	0.48024900	7.73514300	4.85840600
H	1.50483800	7.86021000	3.41193100
C	1.70351100	5.28209500	4.35189500
H	2.64636400	5.66042500	3.94216200

H	1.67493300	5.52691800	5.41973200
H	1.67951800	4.19710700	4.24060200
C	0.51335500	2.23840400	-0.65937700
H	0.44877800	1.84168200	0.35889100
H	1.01423200	1.47793200	-1.27179000
C	1.38557700	3.51735300	-0.64759900
H	1.48154900	3.85021600	-1.69046400
C	2.75780100	3.23439000	-0.08861700
H	2.75668200	2.84785800	0.92999100
C	3.94625800	3.39826200	-0.68402600
C	4.83286000	2.83862300	-2.93317000
C	4.23073000	3.90952000	-2.08400400
H	4.83944400	3.12301200	-0.12354700
H	4.26050100	1.94594800	-3.16643600
H	4.93324000	4.74958600	-2.03783300
H	3.30637600	4.27366300	-2.55211300
C	6.20341800	2.92494100	-3.40905000
O	6.95029200	3.87016700	-3.17472300
O	6.53183100	1.82815900	-4.13916500
C	7.86792100	1.66795500	-4.73128400
C	8.13483900	2.78912600	-5.74262600
H	8.20404200	3.75894200	-5.24759500
H	9.07673100	2.59315900	-6.26769200
H	7.33231900	2.82712700	-6.48750400
C	7.75988000	0.31480500	-5.43973600
H	8.70740400	0.06854100	-5.93060400
H	7.52352100	-0.47851900	-4.72314800
H	6.97143800	0.33842400	-6.19906800
C	8.93005200	1.61782900	-3.62653500
H	8.68362000	0.83713500	-2.89842800
H	9.90523000	1.37759600	-4.06535300
H	9.00546400	2.57459300	-3.10742800

UPBEPBE/6-311+G(d,p)-THF(SMD)//UB3LYP/6-31G(d)
HF= -1081.8208444
DLPNO-CCSD(T)/def2-TZVPP-gas//UB3LYP/6-31G(d)
HF= -1081.065031165502

Figure S10
A (*tert*-butyl radical)

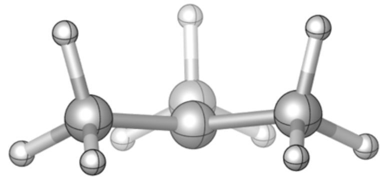

UB3LYP/6-31G(d)
Zero-point correction= 0.117263 (Hartree/Particle)
Thermal correction to Energy= 0.123654
Thermal correction to Enthalpy= 0.124599
Thermal correction to Gibbs Free Energy= 0.087720
Sum of electronic and zero-point Energies= -157.681059
Sum of electronic and thermal Energies= -157.674668
Sum of electronic and thermal Enthalpies= -157.673724
Sum of electronic and thermal Free Energies= -157.710603

C	-1.03902100	2.59763800	-1.57900000
C	-1.88899000	2.14116500	-2.72450300
H	-2.95294200	2.11501600	-2.45623700
H	-1.80507200	2.81223200	-3.60082600
H	-1.59990100	1.14110900	-3.07232100
C	-1.53543100	3.71023700	-0.70784600
H	-2.61346600	3.62866100	-0.51858000
H	-1.01950700	3.72981800	0.26069900
H	-1.37386700	4.70448100	-1.16688400
C	0.42725600	2.29349200	-1.59954600
H	0.62839400	1.28673700	-1.98773200
H	0.99051600	2.99453100	-2.24498600
H	0.87270900	2.36855300	-0.59925900

UPBEPBE/6-311+G(d,p)-THF(SMD)//UB3LYP/6-31G(d)
HF= -157.607061

A (1-phenyl-1-cyclopropylethene)

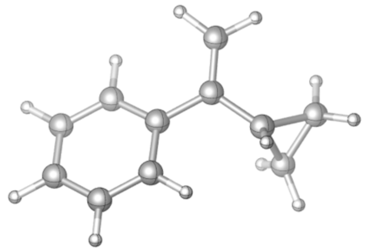

UB3LYP/6-31G(d)
Zero-point correction= 0.196882 (Hartree/Particle)
Thermal correction to Energy= 0.206461
Thermal correction to Enthalpy= 0.207405
Thermal correction to Gibbs Free Energy= 0.161118
Sum of electronic and zero-point Energies= -426.148247
Sum of electronic and thermal Energies= -426.138668
Sum of electronic and thermal Enthalpies= -426.137724
Sum of electronic and thermal Free Energies= -426.184011

C	-4.72782200	1.94309100	-1.38219700
H	-5.24652100	1.95422900	-0.42853600
H	-5.34283400	2.06908300	-2.26755400
C	-3.39570600	1.80876500	-1.47894400
C	-2.72101600	1.86981700	-2.82146900
C	-2.13461200	0.62636000	-3.45479400
C	-3.35209000	1.28397000	-4.05896600
H	-2.14544800	2.78105400	-2.98683500
H	-2.28066300	-0.31575800	-2.93328300
H	-1.18303400	0.70721200	-3.97350800
H	-4.30297600	0.76943500	-3.95456000
C	-2.52412800	1.63507500	-0.28245100
C	-3.02898300	1.12391900	0.92774700
C	-1.16000500	1.97175400	-0.32794200
C	-2.21452000	0.98240800	2.04810000
H	-4.06686500	0.80992700	0.98477200
C	-0.34306700	1.83401600	0.79430000
H	-0.73041200	2.35079800	-1.24933700
C	-0.86578100	1.34152800	1.98974300
H	-2.63191900	0.57941400	2.96738200
H	0.70623300	2.11073900	0.73069600
H	-0.22908900	1.22828200	2.86306500
H	-3.23924800	1.82346600	-4.99548200

UPBEPBE/6-311+G(d,p)-THF(SMD)//UB3LYP/6-31G(d)
HF= -425.8912302

A-TS

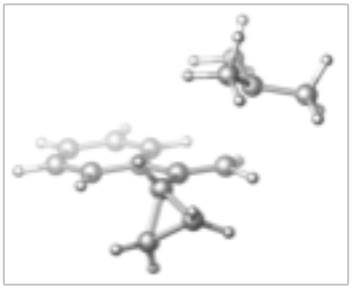

Imaginary frequency= -336.84
UB3LYP/6-31G(d)
Zero-point correction= 0.316089 (Hartree/Particle)
Thermal correction to Energy= 0.332357
Thermal correction to Enthalpy= 0.333301
Thermal correction to Gibbs Free Energy= 0.269790
Sum of electronic and zero-point Energies= -583.820667
Sum of electronic and thermal Energies= -583.804399
Sum of electronic and thermal Enthalpies= -583.803455
Sum of electronic and thermal Free Energies= -583.866966

C	-4.71749600	4.72767100	-2.30889400
H	-3.63395300	4.67879400	-2.15331500
H	-4.94830400	4.24321300	-3.26464400
H	-4.98117000	5.79553400	-2.41477200
C	-6.93913400	3.87323400	-1.37270400
H	-7.15320500	3.43325400	-2.35410000
H	-7.36430400	3.21745300	-0.60255100
H	-7.49715400	4.82547400	-1.32145500
C	-5.46581600	4.10005800	-1.16614900
C	-4.71517400	1.83815900	-1.24950700
H	-5.14928600	1.69164400	-0.26673300
H	-5.40052000	1.71405800	-2.07916800
C	-3.36519400	1.72384200	-1.46617100
C	-2.83389100	1.69433800	-2.87049900
C	-2.41545100	0.38752200	-3.52485100
C	-3.62011000	1.13799200	-4.03153000
H	-2.19030600	2.53849000	-3.12295700
H	-2.59342300	-0.52724900	-2.96612000
H	-1.51150800	0.37562000	-4.12887600
H	-4.59675400	0.70287800	-3.84049600
C	-2.37454700	1.68937200	-0.37029000
C	-2.74652600	1.76978300	0.99107700
C	-0.99568200	1.56472300	-0.64599500
C	-1.79991100	1.72819800	2.00894900
H	-3.79242700	1.86989200	1.26038800
C	-0.04693700	1.52649100	0.37441400
H	-0.66270600	1.48610600	-1.67455400
C	-0.43948000	1.60802500	1.71025400

H	-2.12614900	1.79198600	3.04417800
H	1.00589800	1.42958400	0.12074400
H	0.29920400	1.57758200	2.50656700
H	-3.54557800	1.65099400	-4.98669700
C	-5.04996400	4.53790300	0.21151000
H	-3.96659100	4.45941100	0.35369400
H	-5.32399500	5.59420000	0.38738800
H	-5.54345100	3.95009100	0.99526300

UPBEPBE/6-311+G(d,p)-THF(SMD)//UB3LYP/6-31G(d)

HF= -583.4968655

B

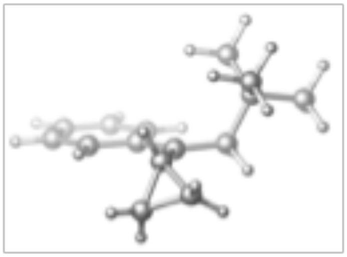

UB3LYP/6-31G(d)
Zero-point correction= 0.320427 (Hartree/Particle)
Thermal correction to Energy= 0.335826
Thermal correction to Enthalpy= 0.336770
Thermal correction to Gibbs Free Energy= 0.276914
Sum of electronic and zero-point Energies= -583.861152
Sum of electronic and thermal Energies= -583.845754
Sum of electronic and thermal Enthalpies= -583.844809
Sum of electronic and thermal Free Energies= -583.904666

C	-4.98184700	4.41306300	-2.59292000
H	-3.90180300	4.49780200	-2.75085800
H	-5.40281700	3.85787500	-3.43950900
H	-5.40063300	5.42690700	-2.61625000
C	-6.83909500	3.71060000	-1.06342500
H	-7.33346700	3.16034900	-1.87378500
H	-7.11778300	3.23314400	-0.11575600
H	-7.24557900	4.72933200	-1.05501300
C	-5.30936900	3.73118700	-1.25179800
C	-4.81813500	2.23783500	-1.23946000
H	-5.16470800	1.78929600	-0.30124700
H	-5.37383600	1.72423100	-2.03469700
C	-3.34536800	1.97607600	-1.43391900
C	-2.79333500	1.94380200	-2.82657200
C	-2.34883400	0.64128800	-3.48010600
C	-3.55095600	1.37986500	-4.00463700
H	-2.14860300	2.79037300	-3.07590200
H	-2.52252400	-0.27746800	-2.92677900
H	-1.43337100	0.64395900	-4.06672900
H	-4.52651700	0.93384300	-3.83211700
C	-2.44144800	1.78768700	-0.33225700
C	-2.86800200	1.77886500	1.02838800
C	-1.04516800	1.59076600	-0.55236800
C	-1.97407200	1.58956700	2.07317000
H	-3.91585400	1.92213500	1.26438500
C	-0.15789500	1.40768800	0.49875900
H	-0.66750300	1.58101600	-1.56807400
C	-0.60960000	1.40381700	1.82343600

H	-2.34339000	1.58726700	3.09584100
H	0.89865500	1.26373400	0.28571500
H	0.08682600	1.25758400	2.64431000
H	-3.46829400	1.89701300	-4.95695500
C	-4.67185200	4.53695500	-0.10614800
H	-3.57969200	4.55309800	-0.18306300
H	-5.02665000	5.57504500	-0.12577000
H	-4.93167600	4.11852600	0.87351000

UPBEPBE/6-311+G(d,p)-THF(SMD)//UB3LYP/6-31G(d)
HF= -583.5387225

B-TS-Z

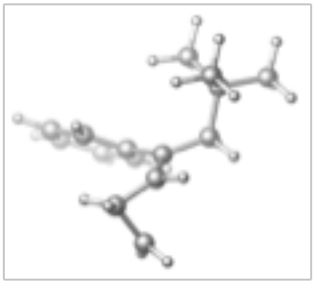

Imaginary frequency= -549.05
UB3LYP/6-31G(d)
Zero-point correction= 0.317692 (Hartree/Particle)
Thermal correction to Energy= 0.333263
Thermal correction to Enthalpy= 0.334207
Thermal correction to Gibbs Free Energy= 0.274184
Sum of electronic and zero-point Energies= -583.841533
Sum of electronic and thermal Energies= -583.825961
Sum of electronic and thermal Enthalpies= -583.825017
Sum of electronic and thermal Free Energies= -583.885040

C	-4.33486900	5.90033300	-2.13264100
H	-3.25741500	5.73035200	-2.23325800
H	-4.79390100	5.74305300	-3.11734300
H	-4.48639200	6.95216700	-1.86042700
C	-6.46722600	5.23749700	-0.98674900
H	-6.96220500	5.05186200	-1.94832500
H	-6.94265500	4.59379500	-0.23595800
H	-6.66486500	6.27910600	-0.70507300
C	-4.95169400	4.96963100	-1.07330600
C	-4.76180200	3.47178900	-1.48402400
H	-5.27663700	2.86319400	-0.72769800
H	-5.31168200	3.31616600	-2.42170300
C	-3.34089000	2.97023900	-1.66712000
C	-2.82979900	2.88847700	-2.96274600
C	-1.57379800	2.22279000	-3.43903300
C	-2.51134500	1.11912900	-3.75532500
H	-3.41244400	3.36030000	-3.74988500
H	-0.84019500	1.99214700	-2.66858000
H	-1.09923500	2.70570100	-4.29616600
H	-2.99789000	1.04864800	-4.72087800
C	-2.57510400	2.50638500	-0.48859600
C	-3.15692900	1.66563500	0.48217000
C	-1.23398500	2.89605900	-0.28540400
C	-2.43247900	1.22521900	1.58841200
H	-4.18394400	1.33427300	0.35662600
C	-0.51044300	2.45925700	0.82254900
H	-0.77131100	3.57893100	-0.99229900
C	-1.10420100	1.61771700	1.76577000

H	-2.90721900	0.56883100	2.31356200
H	0.51769000	2.78694800	0.95527900
H	-0.54121100	1.27749200	2.63064900
C	-4.31454900	5.25936200	0.29805800
H	-4.72113400	4.59942500	1.07403600
H	-3.22948900	5.11872700	0.27970500
H	-4.51457300	6.29484000	0.60131500
H	-2.70652700	0.34771100	-3.02116000

UPBEPBE/6-311+G(d,p)-THF(SMD)//UB3LYP/6-31G(d)
HF= -583.5168145

C-Z

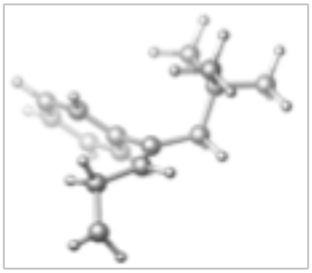

UB3LYP/6-31G(d)
Zero-point correction= 0.317233 (Hartree/Particle)
Thermal correction to Energy= 0.333842
Thermal correction to Enthalpy= 0.334787
Thermal correction to Gibbs Free Energy= 0.272107
Sum of electronic and zero-point Energies= -583.854576
Sum of electronic and thermal Energies= -583.837966
Sum of electronic and thermal Enthalpies= -583.837022
Sum of electronic and thermal Free Energies= -583.899701

C	-4.36187500	5.85400500	-2.14114100
H	-3.30358500	5.62147500	-2.29985800
H	-4.88827500	5.67371500	-3.08715600
H	-4.44279500	6.92414600	-1.91427800
C	-6.45073900	5.37072600	-0.83840600
H	-7.01281800	5.16746100	-1.75846000
H	-6.91340700	4.79337500	-0.02814000
H	-6.57275700	6.43466000	-0.60119000
C	-4.95973900	5.01147600	-1.00015100
C	-4.88643200	3.49003600	-1.34839400
H	-5.37226900	2.94146400	-0.52976800
H	-5.50929600	3.32808600	-2.23728200
C	-3.51446000	2.88343300	-1.61258200
C	-3.11084100	2.68435800	-2.88261900
C	-1.85053300	2.01821100	-3.38170600
C	-2.12810200	0.96634800	-4.41006000
H	-3.79129600	2.99693500	-3.67570100
H	-1.29290000	1.58788700	-2.53342000
H	-1.17521100	2.77310900	-3.81627800
H	-1.37986300	0.70039100	-5.15020200
C	-2.68108900	2.49234800	-0.43552800
C	-3.20156800	1.65838000	0.56923200
C	-1.35715800	2.94301500	-0.29385800
C	-2.42626600	1.28048100	1.66487600
H	-4.21837300	1.28475500	0.48224000
C	-0.58128300	2.57138000	0.80496300
H	-0.94250900	3.60864900	-1.04591000
C	-1.11249900	1.73689200	1.78905500

H	-2.84930000	0.62625800	2.42298500
H	0.43777100	2.93935100	0.89305200
H	-0.51019100	1.44666000	2.64574200
C	-4.22842400	5.33343300	0.31654800
H	-4.61411800	4.72832000	1.14601100
H	-3.15286800	5.14712100	0.24398700
H	-4.36798900	6.38929700	0.58040200
H	-3.00998800	0.33814900	-4.33037100

UPBEPBE/6-311+G(d,p)-THF(SMD)//UB3LYP/6-31G(d)
HF= -583.5226674

B-TS-E

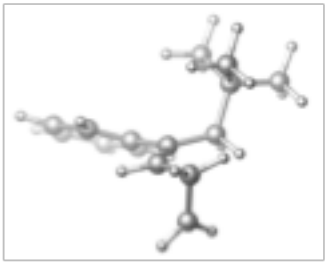

Imaginary frequency= -554.83
UB3LYP/6-31G(d)
Zero-point correction= 0.318020 (Hartree/Particle)
Thermal correction to Energy= 0.333448
Thermal correction to Enthalpy= 0.334392
Thermal correction to Gibbs Free Energy= 0.274984
Sum of electronic and zero-point Energies= -583.841682
Sum of electronic and thermal Energies= -583.826253
Sum of electronic and thermal Enthalpies= -583.825309
Sum of electronic and thermal Free Energies= -583.884717

C	-4.45252800	6.04190800	-1.95650900
H	-3.38590800	5.85594000	-2.12214800
H	-4.96416800	5.96879200	-2.92424800
H	-4.56388700	7.07447100	-1.60339400
C	-6.55128100	5.32304400	-0.78348500
H	-7.07936600	5.17989200	-1.73469000
H	-7.00433700	4.65041900	-0.04434100
H	-6.73474900	6.35264800	-0.45268100
C	-5.04072700	5.05456500	-0.93254200
C	-4.87385500	3.57555700	-1.42526400
H	-5.38521400	2.93570400	-0.69483700
H	-5.45460600	3.47504400	-2.35071800
C	-3.46415500	3.06917800	-1.65382000
C	-2.91677600	2.99793800	-2.93618500
C	-3.54639000	3.37870200	-4.23881000
C	-3.85152700	1.92885500	-4.30737100
H	-1.90098400	2.62528900	-3.02516500
H	-2.84240000	3.74905500	-4.98743400
H	-4.41174900	4.03754700	-4.17535600
H	-4.77920800	1.54470800	-3.90226300
C	-2.66802900	2.56304500	-0.51610500
C	-3.26450600	1.91211300	0.58573400
C	-1.26118500	2.69872500	-0.48997700
C	-2.50064100	1.41116300	1.63755600
H	-4.34017400	1.76914200	0.61082600
C	-0.49815400	2.19980700	0.56202000
H	-0.76422900	3.23502000	-1.29326100
C	-1.11142400	1.54915000	1.63506100

H	-2.99559600	0.90424400	2.46234400
H	0.58094100	2.33241400	0.54991800
H	-0.51719900	1.16291900	2.45863700
C	-4.35802300	5.27543500	0.42981900
H	-4.74049700	4.58234500	1.18839800
H	-3.27468300	5.13431100	0.36844000
H	-4.54487000	6.29648000	0.78551800
H	-3.15876400	1.22816400	-4.75710000

UPBEPBE/6-311+G(d,p)-THF(SMD)//UB3LYP/6-31G(d)
HF= -583.5185922

C-E

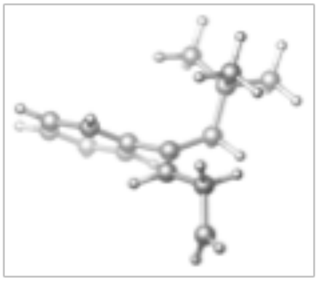

UB3LYP/6-31G(d)
Zero-point correction= 0.317366 (Hartree/Particle)
Thermal correction to Energy= 0.333918
Thermal correction to Enthalpy= 0.334862
Thermal correction to Gibbs Free Energy= 0.272268
Sum of electronic and zero-point Energies= -583.854632
Sum of electronic and thermal Energies= -583.838081
Sum of electronic and thermal Enthalpies= -583.837137
Sum of electronic and thermal Free Energies= -583.899731

C	-4.39163900	5.93974900	-2.02903300
H	-3.34166300	5.69118200	-2.21759500
H	-4.94400300	5.80644900	-2.96746700
H	-4.44384200	7.00258600	-1.76288800
C	-6.46659500	5.43325000	-0.71172600
H	-7.04318400	5.25080100	-1.62719800
H	-6.91999800	4.84199700	0.09367500
H	-6.58044500	6.49272300	-0.45179000
C	-4.98019700	5.07099600	-0.90270500
C	-4.91611300	3.55465900	-1.28162300
H	-5.39654800	2.99719200	-0.46736500
H	-5.55581700	3.40643600	-2.15895300
C	-3.54525100	2.95545300	-1.54951400
C	-3.04334300	2.76287600	-2.78682400
C	-3.66388300	3.09214200	-4.12252200
C	-3.75008800	1.90426800	-5.03002200
H	-2.07264600	2.27352000	-2.86433400
H	-3.06567900	3.87637100	-4.61422800
H	-4.66001000	3.54458800	-3.98836100
H	-3.95393700	0.91685600	-4.62792600
C	-2.72193100	2.52091500	-0.37861500
C	-3.28173000	1.79406700	0.68674300
C	-1.34469700	2.80155300	-0.32487200
C	-2.49609500	1.35448200	1.75179000
H	-4.33892300	1.54455300	0.67409800
C	-0.55786000	2.36595600	0.74076300
H	-0.89519500	3.38634000	-1.12253100
C	-1.12984000	1.63855600	1.78555500

H	-2.95369700	0.78406000	2.55618100
H	0.50266200	2.60426600	0.75827300
H	-0.51948000	1.30114300	2.61892400
C	-4.22661300	5.35938200	0.40940600
H	-4.60411200	4.74067000	1.23232000
H	-3.15387800	5.16569300	0.31554100
H	-4.35361800	6.41081700	0.69607800
H	-3.75336100	2.02638700	-6.10868600

UPBEPBE/6-311+G(d,p)-THF(SMD)//UB3LYP/6-31G(d)
HF= -583.5234239

^{5}Fe-Ar-Br

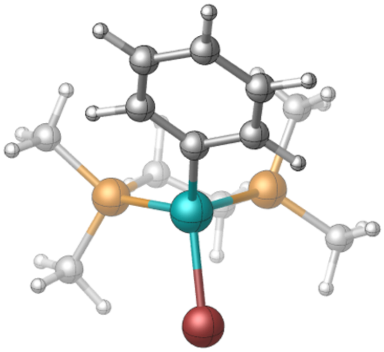

UB3LYP/6-31G(d)
Zero-point correction= 0.301739 (Hartree/Particle)
Thermal correction to Energy= 0.324250
Thermal correction to Enthalpy= 0.325194
Thermal correction to Gibbs Free Energy= 0.245459
Sum of electronic and zero-point Energies= -4987.743558
Sum of electronic and thermal Energies= -4987.721047
Sum of electronic and thermal Enthalpies= -4987.720102
Sum of electronic and thermal Free Energies= -4987.799837

P	-2.00088200	-1.36743800	-0.70579700
P	-1.65457400	1.63780300	0.59884700
Fe	-0.08476300	-0.28989300	0.43636900
C	-2.47328100	-3.10122700	-0.29356700
C	-1.28860100	3.31331300	-0.09136600
C	1.56369700	-0.09865700	-0.72284200
C	2.15905000	-1.24863000	-1.28964600
C	2.19131200	1.12971600	-1.02418600
C	3.29556400	-1.18341900	-2.09959200
H	1.73223000	-2.23171000	-1.08461600
C	3.32832300	1.21272500	-1.83327400
H	1.78995000	2.05756600	-0.61375400
C	3.88384200	0.05260300	-2.37578000
H	3.72533300	-2.09399700	-2.51299600
H	3.78348300	2.17998100	-2.03871700
H	4.76920200	0.11066900	-3.00492900
C	-2.25272100	2.02273100	2.30282500
H	-1.40773900	2.38463300	2.89668600
H	-2.61256600	1.10690900	2.77946700
H	-3.04734100	2.77760100	2.30030700
C	-2.08317900	-1.33502900	-2.55311100
H	-1.27251700	-1.94941800	-2.95697000
H	-1.93201900	-0.31433400	-2.91786500
H	-3.04008600	-1.71214900	-2.93195500
H	-2.15976900	3.97688300	-0.04901800
H	-0.47386900	3.76535700	0.48319000

H	-0.95761500	3.22254400	-1.13049500
H	-3.42681400	-3.39814100	-0.74448100
H	-1.68416800	-3.77348300	-0.64531900
H	-2.53121900	-3.20025600	0.79414400
C	-3.21189700	1.14487700	-0.31794500
H	-4.07074500	1.73114000	0.03234600
H	-3.05242700	1.41021000	-1.37095400
C	-3.49321000	-0.36600700	-0.19378100
H	-4.37671500	-0.64504400	-0.78180500
H	-3.70056100	-0.62861900	0.85126400
Br	-0.37627600	-1.31803200	2.56757100

UPBEPBE/6-311+G(d,p)-SDD(Fe)-THF(SMD)//UB3LYP/6-31G(d)

HF= -3849.5802828

^{4}A'-Fe-TS

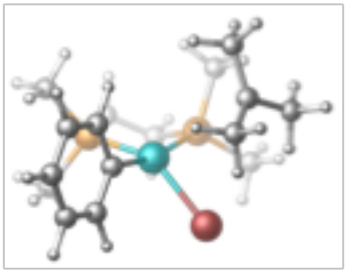

Imaginary frequency= -22.60
UB3LYP/6-31G(d)
Zero-point correction= 0.421435 (Hartree/Particle)
Thermal correction to Energy= 0.450441
Thermal correction to Enthalpy= 0.451385
Thermal correction to Gibbs Free Energy= 0.358509
Sum of electronic and zero-point Energies= -5145.402887
Sum of electronic and thermal Energies= -5145.373881
Sum of electronic and thermal Enthalpies= -5145.372937
Sum of electronic and thermal Free Energies= -5145.465813

P	-2.26402700	-1.40255000	-0.36835400
P	-1.69681100	1.62377900	-0.01863600
Fe	-0.40140200	-0.15682800	0.33930300
C	-2.72738500	-3.00942500	0.39724100
C	-1.27552800	2.93069800	-1.25057000
C	1.17691100	1.00765300	0.54626600
C	1.75302800	1.35140600	1.78572200
C	1.80950400	1.53298000	-0.60157200
C	2.88545800	2.16608600	1.87466900
H	1.32588700	0.94995700	2.70180500
C	2.95280200	2.33611300	-0.52481200
H	1.41132700	1.31229100	-1.59445500
C	3.49434300	2.66139700	0.71968400
H	3.30246500	2.40395100	2.85159400
H	3.41759800	2.70824200	-1.43623700
H	4.37997300	3.28865100	0.78811300
C	-1.93290500	2.59359800	1.53525900
H	-0.95998100	2.97691300	1.85691100
H	-2.31647400	1.94110100	2.32557200
H	-2.62543100	3.42977900	1.38677800
C	-2.42813900	-1.74296800	-2.18225800
H	-1.65767000	-2.45662700	-2.48803300
H	-2.27816500	-0.82310700	-2.75648700
H	-3.41147100	-2.15877300	-2.43001500
H	-2.03696700	3.71799900	-1.27479900
H	-0.30762900	3.36563900	-0.98852200
H	-1.18868900	2.48658300	-2.24733600
H	-3.69059900	-3.37632900	0.02537100

H	-1.94700900	-3.74415200	0.18129300
H	-2.76674400	-2.88601500	1.48216300
C	-3.43600400	1.12081100	-0.51123900
H	-4.17597500	1.83761900	-0.13561900
H	-3.48220200	1.15745600	-1.60687700
C	-3.73348100	-0.30605200	-0.02027800
H	-4.65152000	-0.70255800	-0.47161700
H	-3.87145600	-0.31528900	1.06823200
Br	0.14643100	-1.75014900	2.02488200
C	1.82123200	-2.42449100	-1.66307400
C	2.96788600	-2.08532200	-0.75926500
H	2.72265400	-2.28799500	0.28849300
H	3.86302600	-2.68669100	-1.01050000
H	3.25381100	-1.03106200	-0.83908300
C	1.07791400	-3.69788700	-1.38825800
H	0.78870700	-3.76342700	-0.33188800
H	0.17656400	-3.79538100	-2.00782000
H	1.70057400	-4.58732100	-1.60360700
C	1.82853400	-1.87516100	-3.05853300
H	0.84676200	-1.96018300	-3.54329100
H	2.13244000	-0.82107700	-3.07557600
H	2.54362400	-2.41540400	-3.70906200

UPBEPBE/6-311+G(d,p)-SDD(Fe)-THF(SMD)//UB3LYP/6-31G(d)
HF= -4007.1836398

^{4}C-E-Fe-TS

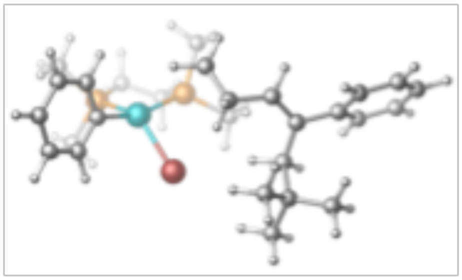

Imaginary frequency= -16.41
UB3LYP/6-31G(d)
Zero-point correction= 0.621398 (Hartree/Particle)
Thermal correction to Energy= 0.660767
Thermal correction to Enthalpy= 0.661711
Thermal correction to Gibbs Free Energy= 0.543968
Sum of electronic and zero-point Energies= -5571.580835
Sum of electronic and thermal Energies= -5571.541465
Sum of electronic and thermal Enthalpies= -5571.540521
Sum of electronic and thermal Free Energies= -5571.658265

P	-1.17610400	-0.44198300	1.31468700
P	-2.02970900	0.85000000	-1.36285000
Fe	-0.13790500	0.98800800	-0.22137800
C	-0.96037500	-0.25793100	3.13121000
C	-2.11086100	0.22193000	-3.09451400
C	0.82363500	1.69037100	-1.78369100
C	1.06496800	3.05174900	-2.05519600
C	1.38093900	0.76165900	-2.68894500
C	1.81951200	3.46102200	-3.15762400
H	0.67959900	3.80877600	-1.37529500
C	2.14766300	1.16094900	-3.78920500
H	1.21884100	-0.30977100	-2.54636100
C	2.36729400	2.51747800	-4.03012700
H	1.98990700	4.52200100	-3.33062200
H	2.57016700	0.41260700	-4.45721000
H	2.95907900	2.83580300	-4.88496400
C	-2.81110600	2.51870800	-1.49812000
H	-2.15071200	3.16759900	-2.08122200
H	-2.92118100	2.95831500	-0.50220400
H	-3.79139500	2.46489100	-1.98487100
C	-0.92594900	-2.25769500	1.05603400
H	0.11685600	-2.50320500	1.27813300
H	-1.12094500	-2.52683800	0.01298900
H	-1.57855100	-2.85272600	1.70475700
H	-3.12867300	0.27605300	-3.49634300
H	-1.43910500	0.81896900	-3.71686000
H	-1.76763600	-0.81683000	-3.12905900
H	-1.62664900	-0.92160600	3.69361000

H	0.07914900	-0.48895300	3.38154400
H	-1.14541000	0.78245600	3.40904700
C	-3.30918000	-0.18653300	-0.46466200
H	-4.32486700	0.17531000	-0.66483200
H	-3.24353800	-1.20081800	-0.87805300
C	-3.00825200	-0.21113700	1.04449000
H	-3.59529300	-0.98273800	1.55786000
H	-3.26373500	0.75391100	1.50002400
Br	0.71219400	2.46285400	1.44955300
C	4.63723900	2.36472000	3.73054800
H	5.27293900	1.48689800	3.56922700
H	4.13093200	2.59926000	2.78745900
H	5.29119900	3.21053700	3.97686100
C	2.72921100	3.37229400	5.00558900
H	2.15686300	3.54973100	4.08756500
H	2.01537500	3.25770500	5.83174900
H	3.33381500	4.26480200	5.20977300
C	3.62378700	2.12364400	4.86384100
C	2.67690200	0.92640000	4.52049300
H	1.95317600	0.84956200	5.34271000
H	2.09734700	1.21413100	3.63796500
C	3.31215300	-0.43705800	4.29626100
C	3.60961700	-0.92839200	3.07485300
C	3.46319100	-0.25920900	1.73280700
C	2.83186900	-1.12045800	0.68614500
H	4.01969700	-1.93876300	3.02761600
H	4.47310400	0.03240400	1.37787500
H	2.91876200	0.69003300	1.80171200
H	2.75389100	-2.19694100	0.81769000
C	3.59549200	-1.29415600	5.48896300
C	2.66159600	-1.44535900	6.52937900
C	4.80694100	-2.00163500	5.59466300
C	2.92011400	-2.27844700	7.61742400
H	1.71055000	-0.92253000	6.48012700
C	5.06942800	-2.83277900	6.68314000
H	5.55856800	-1.87523300	4.82051200
C	4.12585600	-2.97688000	7.70137200
H	2.17467700	-2.38320800	8.40199000
H	6.01877900	-3.35955100	6.74032700
H	4.33008400	-3.61974400	8.55349000
C	4.37987600	1.88808000	6.18434200
H	3.68802800	1.69205400	7.01274900
H	5.06464100	1.03624400	6.11700400
H	4.97279200	2.77351700	6.44680400
H	2.69286700	-0.72317100	-0.31566300

UPBEPBE/6-311+G(d,p)-SDD(Fe)-THF(SMD)//UB3LYP/6-31G(d)

HF= -4433.1024959

15. Crystallographic Data

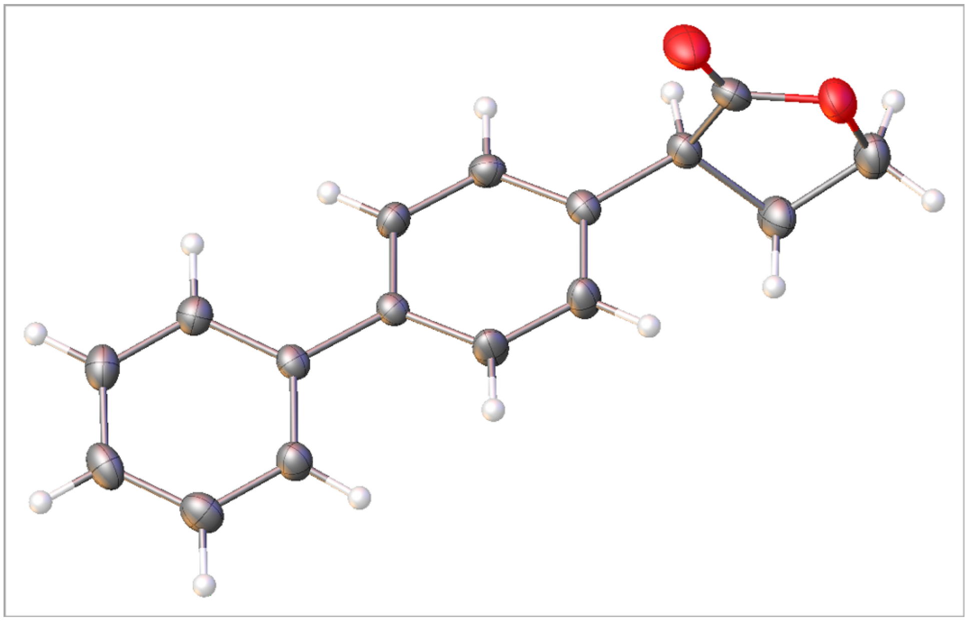

Table S5. Crystal data and structure refinement for UM3320.

Identification code	UM3320
Empirical formula	C16H14O2
Formula weight	238.27
Temperature/K	150(2)
Crystal system	monoclinic
Space group	$P2_1$
a/Å	6.2309(7)
b/Å	7.4169(8)
c/Å	13.0054(15)
α/°	90
β/°	90.1686(18)
γ/°	90
Volume/Å3	601.03(12)
Z	2
ρcalcg/cm^3	1.317
μ/mm^{-1}	0.086

F(000)	252.0
Crystal size/mm^3	0.44 × 0.20 × 0.025
Radiation	MoKα (λ = 0.71073)
2Θ range for data collection/°	3.132 to 60
Index ranges	-8 ≤ h ≤ 8, -10 ≤ k ≤ 10, -17 ≤ l ≤ 18
Reflections collected	8759
Independent reflections	3504 [R_{int} = 0.0171, R_{sigma} = 0.0233]
Data/restraints/parameters	3504/1/219
Goodness-of-fit on F^2	1.000
Final R indexes [I>=2σ (I)]	R_1 = 0.0364, wR_2 = 0.0709
Final R indexes [all data]	R_1 = 0.0424, wR_2 = 0.0725
Largest diff. peak/hole / e Å^{-3}	0.27/-0.16
Flack parameter	0.1(5)

Table S6. Fractional Atomic Coordinates and Equivalent Isotropic Displacement Parameters ($Å_2$) for UM3320. U_{eq} is defined as 1/3 of of the trace of the orthogonalised U_{IJ} tensor.

Atom	x	y	z	U(eq)
O1	0.4025(3)	0.7044(3)	0.09860(12)	0.0419(4)
C2	0.5568(3)	0.6086(3)	0.09369(14)	0.0284(4)
O3	0.7320(2)	0.6562(2)	0.03879(11)	0.0342(4)
C4	0.8910(4)	0.5119(3)	0.04036(18)	0.0334(5)
C5	0.8327(3)	0.3963(3)	0.13235(16)	0.0294(4)
C6	0.5895(3)	0.4225(3)	0.14136(14)	0.0239(4)
C7	0.4942(3)	0.4068(2)	0.24762(13)	0.0208(4)
C8	0.3109(3)	0.3045(3)	0.26506(14)	0.0231(4)
C9	0.2160(3)	0.2968(3)	0.36181(15)	0.0232(4)
C10	0.3023(3)	0.3915(2)	0.44541(13)	0.0189(3)
C11	0.4914(3)	0.4883(3)	0.42787(15)	0.0253(4)
C12	0.5854(3)	0.4961(3)	0.33130(16)	0.0260(4)
C13	0.2008(3)	0.3882(2)	0.54925(14)	0.0198(3)

C14	-0.0044(3)	0.3150(3)	0.56447(16)	0.0252(4)
C15	-0.0935(3)	0.3052(3)	0.66208(16)	0.0291(4)
C16	0.0181(4)	0.3690(3)	0.74686(16)	0.0302(4)
C17	0.2198(4)	0.4441(3)	0.73316(16)	0.0303(4)
C18	0.3098(3)	0.4536(3)	0.63577(15)	0.0261(4)

Table S7. Anisotropic Displacement Parameters (Å^2) for UM3320. The Anisotropic displacement factor exponent takes the form: $-2\pi^2[h^2a^{*2}U_{11}+2hka^*b^*U_{12}+\ldots]$.

Atom	U11	U22	U33	U23	U13	U12
O1	0.0372(9)	0.0491(10)	0.0392(9)	0.0143(8)	0.0007(7)	0.0132(8)
C2	0.0299(9)	0.0372(11)	0.0181(8)	0.0030(8)	-0.0026(7)	-0.0003(8)
O3	0.0351(8)	0.0370(9)	0.0305(7)	0.0094(7)	0.0048(6)	-0.0012(7)
C4	0.0307(11)	0.0406(13)	0.0290(11)	-0.0021(10)	0.0078(8)	-0.0032(10)
C5	0.0272(9)	0.0336(11)	0.0273(10)	-0.0006(9)	0.0051(7)	0.0055(8)
C6	0.0257(9)	0.0276(10)	0.0183(8)	-0.0022(8)	0.0017(7)	-0.0013(8)
C7	0.0235(8)	0.0193(9)	0.0195(8)	0.0005(7)	0.0015(6)	0.0016(7)
C8	0.0228(8)	0.0257(9)	0.0210(9)	-0.0027(8)	-0.0026(7)	-0.0012(8)
C9	0.0201(8)	0.0248(9)	0.0246(9)	-0.0007(8)	0.0002(7)	-0.0045(7)
C10	0.0211(8)	0.0172(8)	0.0185(8)	0.0011(7)	0.0004(6)	0.0008(7)
C11	0.0284(10)	0.0264(10)	0.0212(9)	-0.0041(8)	0.0001(7)	-0.0095(8)
C12	0.0282(10)	0.0261(10)	0.0239(10)	-0.0032(8)	0.0037(8)	-0.0084(8)
C13	0.0233(8)	0.0164(8)	0.0198(8)	0.0020(7)	0.0015(6)	0.0011(7)
C14	0.0240(9)	0.0246(10)	0.0268(9)	-0.0003(8)	0.0017(7)	-0.0005(8)
C15	0.0266(10)	0.0276(10)	0.0331(11)	0.0027(9)	0.0079(8)	-0.0002(9)
C16	0.0381(11)	0.0281(11)	0.0243(10)	0.0011(8)	0.0098(8)	0.0039(9)
C17	0.0374(11)	0.0306(11)	0.0230(10)	-0.0035(8)	0.0004(8)	0.0003(9)
C18	0.0278(10)	0.0272(10)	0.0232(10)	-0.0018(8)	0.0018(7)	-0.0035(8)

Table S8. Bond Lengths for UM3320.

Atom	Atom	Length/Å	Atom	Atom	Length/Å

O1	C2	1.197(3)	C9	C10	1.400(3)
C2	O3	1.353(2)	C10	C11	1.399(3)
C2	C6	1.527(3)	C10	C13	1.493(2)
O3	C4	1.459(3)	C11	C12	1.388(3)
C4	C5	1.517(3)	C13	C18	1.400(3)
C5	C6	1.533(3)	C13	C14	1.403(3)
C6	C7	1.510(2)	C14	C15	1.389(3)
C7	C8	1.391(3)	C15	C16	1.385(3)
C7	C12	1.394(3)	C16	C17	1.387(3)
C8	C9	1.393(3)	C17	C18	1.388(3)

Table S9. Bond Angles for UM3320.

Atom	Atom	Atom	Angle/˚	Atom	Atom	Atom	Angle/˚
O1	C2	O3	121.5(2)	C11	C10	C9	116.89(16)
O1	C2	C6	128.4(2)	C11	C10	C13	121.01(16)
O3	C2	C6	110.06(17)	C9	C10	C13	122.09(15)
C2	O3	C4	110.51(16)	C12	C11	C10	121.77(18)
O3	C4	C5	105.17(16)	C11	C12	C7	120.97(18)
C4	C5	C6	103.16(17)	C18	C13	C14	117.45(18)
C7	C6	C2	112.88(16)	C18	C13	C10	121.03(16)
C7	C6	C5	116.85(16)	C14	C13	C10	121.49(16)
C2	C6	C5	102.38(17)	C15	C14	C13	121.05(19)
C8	C7	C12	117.72(17)	C16	C15	C14	120.56(19)
C8	C7	C6	121.11(16)	C15	C16	C17	119.22(19)
C12	C7	C6	121.17(16)	C16	C17	C18	120.4(2)
C7	C8	C9	121.37(18)	C17	C18	C13	121.29(19)
C8	C9	C10	121.19(18)				

Table S10. Torsion Angles for UM3320.

A	B	C	D	Angle/˚	A	B	C	D	Angle/˚
O1	C2	O3	C4	-176.6(2)	C8	C9	C10	C13	178.75(18)

C6	C2	O3	C4	1.9(2)	C9	C10	C11	C12	2.4(3)
C2	O3	C4	C5	-19.8(2)	C13	C10	C11	C12	-178.57(19)
O3	C4	C5	C6	28.8(2)	C10	C11	C12	C7	-0.1(3)
O1	C2	C6	C7	-38.8(3)	C8	C7	C12	C11	-2.3(3)
O3	C2	C6	C7	142.84(16)	C6	C7	C12	C11	176.9(2)
O1	C2	C6	C5	-165.3(2)	C11	C10	C13	C18	-11.6(3)
O3	C2	C6	C5	16.4(2)	C9	C10	C13	C18	167.4(2)
C4	C5	C6	C7	-150.78(18)	C11	C10	C13	C14	170.3(2)
C4	C5	C6	C2	-26.9(2)	C9	C10	C13	C14	-10.7(3)
C2	C6	C7	C8	108.5(2)	C18	C13	C14	C15	-1.1(3)
C5	C6	C7	C8	-133.2(2)	C10	C13	C14	C15	177.10(18)
C2	C6	C7	C12	-70.7(2)	C13	C14	C15	C16	0.5(3)
C5	C6	C7	C12	47.6(3)	C14	C15	C16	C17	0.4(3)
C12	C7	C8	C9	2.5(3)	C15	C16	C17	C18	-0.7(3)
C6	C7	C8	C9	-176.72(18)	C16	C17	C18	C13	0.0(3)
C7	C8	C9	C10	-0.2(3)	C14	C13	C18	C17	0.9(3)
C8	C9	C10	C11	-2.2(3)	C10	C13	C18	C17	-177.32(18)

Table S11. Hydrogen Atom Coordinates and Isotropic Displacement Parameters (Å2) for UM3320.

Atom	x	y	z	U(eq)
H4A	0.874(4)	0.449(4)	-0.025(2)	0.044(7)
H4B	1.036(4)	0.564(4)	0.0444(18)	0.035(7)
H5A	0.904(3)	0.437(3)	0.1944(18)	0.029(6)
H5B	0.875(4)	0.260(4)	0.1230(18)	0.038(7)
H6	0.516(3)	0.345(3)	0.0931(17)	0.027(6)
H8	0.247(4)	0.234(3)	0.2067(17)	0.027(6)
H9	0.089(4)	0.231(3)	0.3716(17)	0.032(6)
H11	0.558(4)	0.551(4)	0.4839(19)	0.035(7)
H12	0.713(4)	0.565(3)	0.3220(18)	0.035(7)
H14	-0.086(3)	0.272(3)	0.5038(17)	0.026(6)

H15	-0.236(4)	0.258(4)	0.6708(18)	0.039(7)
H16	-0.044(3)	0.358(3)	0.8143(17)	0.025(6)
H17	0.305(4)	0.478(3)	0.7936(19)	0.033(6)
H18	0.455(4)	0.492(4)	0.6291(19)	0.035(7)

Experimental:

A suitable single crystals of $C_{16}H_{14}O_2$ (UM3320) was selected and measured on a Bruker Smart Apex II CCD diffractometer.[22] The crystal was kept at 150(2) K during data collection. The integral intensity were correct for absorption using SADABS software[23] using multi-scan method. Resulting minimum and maximum transmission are 0.844 and 0.998 respectively. The structure was solved with the ShelXT-2014 (Sheldrick, 2015a)[24] program and refined with the ShelXL-2015 (Sheldrick, 2015c)[25] program and least-square minimisation using ShelX software package.[24] Number of restraints used = 1.

Crystal structure determination:

Crystal Data for $C_{16}H_{14}O_2$ (M =238.27 g/mol): monoclinic, space group $P2_1$ (no. 4), a = 6.2309(7) Å, b = 7.4169(8) Å, c = 13.0054(15) Å, β = 90.1686(18)°, V = 601.03(12) Å3, Z = 2, T = 150(2) K, μ(MoKα) = 0.086 mm^{-1}, *Dcalc* = 1.317 g/cm^3, 8759 reflections measured (3.132° ≤ 2Θ ≤ 60°), 3504 unique (R_{int} = 0.0171, R_{sig} = 0.0233) which were used in all calculations. The final R_1 was 0.0364 (I > 2σ(I)) and wR_2 was 0.0725 (all data).

Refinement details:

?

This report has been created with Olex2 [26], compiled on 2018.05.29 svn.r3508 for OlexSys.

16. References

1 Charette, A. B.; Molinaro, C.; Brochu, C. Stability, Reactivity, Solution, and Solid-State Structure of Halomethylzinc Alkoxides. *J. Am. Chem. Soc.* **2001**, *123*, 12160–12167.

2 Kazuta, Y.; Matsuda, A.; Shuto, S. Development of Versatile cis- and trans-Dicarbon-Substituted Chiral Cyclopropane Units: Synthesis of (1*S*,2*R*)- and (1*R*,2*R*)-2-Aminomethyl-1-(1H-imidazol-4-yl)cyclopropanes and Their Enantiomers as Conformationally Restricted Analogues of Histamine. *J. Org. Chem.* **2002**, *67*, 1669–1677.

3 Tan, C. K.; Zhou, L.; Yeung, Y-Y. Amino-thiocarbamate Catalyzed Asymmetric Bromolactonization of 1,2-Disubstituted Olefinic Acids. *Org. Lett.*, **2011**, *13*, 2738–2741.

4 (a) Curley, R. W.; DeLuca, H. F. Ethyl 13,14-Dihydro-13,14-methyleneretinoates: Analogues of all-*trans*- and 13-*cis*-Retinoic Acid. *J. Org. Chem.* **1984**, *49*, 1941–1944; (b) Ieki, R.; Kani, Y.; Tsunoi, S.; Shibata, I. Transition-Metal-Free Coupling Reaction of Vinylcyclopropanes with Aldehydes Catalyzed by Tin Hydride. *Chem. Eur. J.* **2015**, *21*, 6295–6300.

5 Chatterjee, A. K.; Choi, T-L.; Sanders, D. P.; Grubbs, R. H. A General Model for Selectivity in Olefin Cross Metathesis. *J. Am. Chem. Soc.* **2003**, *125*, 11360–11370.

6 Lundin, P. M.; Fu, G. C. Asymmetric Suzuki Cross-Couplings of Activated Secondary Alkyl Electrophiles: Arylations of Racemic α-Chloroamides. *J. Am. Chem. Soc.* **2010**, *132*, 11027–11029.

7 (a) Li, J.; Chen, J.; Jiao, W.; Wang, G.; Li, Y.; Cheng, X.; Li, G. Difluoroalkylation/C–H Annulation Cascade Reaction Induced by Visible-Light Photoredox Catalysis. *J. Org. Chem.* **2016**, *81*, 9992–10001. (b) Chen, C.; Shen, X.; Chen, J.; Hong, X.; Lu, Z. Iron-Catalyzed Hydroboration of Vinylcyclopropanes. *Org. Lett.* **2017**, *19*, 5422–5425. (c) Han, P.; Wang, R.; Wang, D. Z. Electronic polarizability-based stereochemical model for Sharpless AD reactions. *Terahedron* **2011**, *67*, 8873–8878.

8 (a) Jin, M.; Adak, L.; Nakamura, M. Iron-Catalyzed Enantioselective Cross-Coupling Reactions of α-Chloroesters with Aryl Grignard Reagents. *J. Am. Chem. Soc.*, **2015**, *137*, 7128–7134; (b) Liu, L.; Lee, W.; Zhou, J.; Bandyopadhyay, S.; Gutierrez, O. Radical-clock α-halo-esters as Mechanistic Probes for Bisphosphine Iron-catalyzed Cross-coupling Reactions. *Tetrahedron* **2019**, *75*, 129–136; (c) Mo, X.; Hall, D. G. Dual Catalysis Using Boronic Acid and Chiral Amine: Acyclic Quaternary Carbons via Enantioselective Alkylation of Branched Aldehydes with Allylic Alcohols. *J. Am. Chem. Soc.* **2016**, *138*, 10762−10765. (d) Mao, J.; Liu, F.; Wang, M.; Wu, L.; Zheng, B.; Liu, S.; Zhong, J.; Bian, Q.; Walsh, P. J. Cobalt−Bisoxazoline-Catalyzed Asymmetric Kumada Cross-Coupling of Racemic α-Bromo Esters with Aryl Grignard Reagents. *J. Am. Chem. Soc.* **2014**, *136*, 17662−17668.

9 Mandal, P. K.; McMurray, J. S. Pd−C-Induced Catalytic Transfer Hydrogenation with Triethylsilane. *J. Org. Chem*. **2007**, *72*, 6599–6601.

10 Shultz, L. H.; Brookhart, M. Measurement of the Barrier to β-Hydride Elimination in a β-Agostic Palladium−Ethyl Complex: A Model for the Energetics of Chain-Walking in (α-Diimine)PdR+Olefin Polymerization Catalysts**.** *Organometallics*, **2001**, *20*, 3975–3982.

11 Holland, P. L. Distinctive Reaction Pathways at Base Metals in High-Spin Organometallic Catalysts. *Acc. Chem. Res.* **2015**, *48*, 1696–1702.

12 (a) E. Pretsch, T. Clerc, J. Seibl, W. Simon, *Tables of Spectral Data for Structure Determination of Organic Compounds,* 2nd Ed. Spring-Verlag: Berlin **1989**; (b) Wu, Z.; Sun, X.; Potter, K.; Cao, Y.; Zakharov, L. N.; Blakemore, P. R. Stereospecific Synthesis of Alkenes by Eliminative Cross-Coupling of Enantioenriched sp^3-Hybridized Carbenoids. *Angew. Chem. Int. Ed.* **2016**, *55*, 12285–12289.

13 Qabaja, G.; Benavides, A. R.; Liu, S.; Petersen, K. S. Asymmetric Synthesis of Hydroxy Esters with Multiple Stereocenters via a Chiral Phosphoric Acid Catalyzed Kinetic Resolution. *J. Org. Chem.* **2015**, *80*, 133−140.

14 Paras, N. A.; MacMillan, D. W. C. New Strategies in Organic Catalysis: The First Enantioselective Organocatalytic Friedel-Crafts Alkylation. *J. Am. Chem. Soc.* **2001**, *123*, 4370-4371.

15 Qabaja, G.; Wilent, J. E.; Benavides, A. R.; Bullard, G. E.; Petersen, K. S. Facile Synthesis of Versatile Enantioenriched α-Substituted Hydroxy Esters through a Brønsted Acid Catalyzed Kinetic Resolution. *Org. Lett.*, **2013**, *15*, 1266–1269.

16 (a) A. D. Becke. Density□functional Thermochemistry. I. The Effect of the Exchange-only Gradient Correction. *J. Chem. Phys*. **1992**, *96*, 2155–2160. (b) A. D. Becke. Density□functional Thermochemistry. II. The Effect of the Perdew-Wang Generalized-gradient Correlation Correction. *J. Chem. Phys*. **1992**, *97*, 9173–1977. (c) A. D. Becke. Density-functional Thermochemistry. III. The Role of Exact Exchange. *J. Chem. Phys*. **1993**, *98*, 5648–5652.

17 Gaussian 16, Revision B.01, M. J. Frisch, G. W. Trucks, H. B. Schlegel, G. E. Scuseria, M. A. Robb, J. R. Cheeseman, G. Scalmani, V. Barone, G. A. Petersson, H. Nakatsuji, X. Li, M. Caricato, A. V. Marenich, J. Bloino, B. G. Janesko, R. Gomperts, B. Mennucci, H. P. Hratchian, J. V. Ortiz, A. F. Izmaylov, J. L. Sonnenberg, D. Williams-Young, F. Ding, F. Lipparini, F. Egidi, J. Goings, B. Peng, A. Petrone, T. Henderson, D. Ranasinghe, V. G. Zakrzewski, J. Gao, N. Rega, G. Zheng, W. Liang, M. Hada, M. Ehara, K. Toyota, R. Fukuda, J. Hasegawa, M. Ishida, T. Nakajima, Y. Honda, O. Kitao, H. Nakai, T. Vreven, K. Throssell, J. A. Montgomery, Jr., J. E. Peralta, F. Ogliaro, M. J. Bearpark, J. J. Heyd, E. N. Brothers, K. N. Kudin, V. N. Staroverov, T. A. Keith, R. Kobayashi, J. Normand, K. Raghavachari, A. P. Rendell, J. C. Burant, S. S. Iyengar, J. Tomasi, M. Cossi, J. M. Millam, M. Klene, C. Adamo, R. Cammi, J. W. Ochterski, R. L. Martin, K. Morokuma, O. Farkas, J. B. Foresman, and D. J. Fox, Gaussian, Inc., Wallingford CT, 2016.

18 Frisch, M. J. T., G. W.; Schlegel, H. B.; Scuseria, G. E.; Robb, M. A.; Cheeseman, J. R.; Scalmani, G.; Barone, V.; Petersson, G. A.; Nakatsuji, H.; Li, X.; Caricato, M.; Marenich, A.; Bloino, J.; Janesko, B. G.; Gomperts, R.; Mennucci, B.; Hratchian, H. P.; Ortiz, J. V.; Izmaylov, A. F.; Sonnenberg, J. L.; Williams-Young, D.; Ding, F.; Lipparini, F.; Egidi, F.; Goings, J.; Peng, B.; Petrone, A.; Henderson, T.; Ranasinghe, D.; Zakrzewski, V. G.; Gao, J.; Rega, N.; Zheng, G.; Liang, W.; Hada, M.; Ehara, M.; Toyota, K.; Fukuda, R.; Hasegawa, J.; Ishida, M.; Nakajima, T.; Honda, Y.; Kitao, O.; Nakai, H.; Vreven, T.; Throssell, K.; Montgomery, J. A.; Peralta, Jr., J. E.; Ogliaro, F.; Bearpark, M.; Heyd, J. J.; Brothers, E.; Kudin, K. N.; Staroverov, V. N.; Keith, T.; Kobayashi, R.; Normand, J.; Raghavachari, K.; Rendell, A.; Burant, J. C.; Iyengar, S. S.; Tomasi, J.; Cossi, M.; Millam, J. M.; Klene, M.; Adamo, C.; Cammi, R.; Ochterski, J. W.; Martin, R. L.; Morokuma, K.; Farkas, O.; Foresman, J. B.; Fox,D. J. *Gaussian 09, Revision D.01*, Gaussian, Inc., Wallingford CT, 2009.

19 Perdew, J. P.; Burke, K.; Ernzerhof, M. Generalized Gradient Approximation Made Simple. *Phys. Rev. Lett*. **1996**, *77*, 3865–3868.

20 Marenich, A.V.; Cramer, C. J.; Truhlar, D. G. Universal Solvation Model Based on Solute Electron Density and on a Continuum Model of the Solvent Defined by the Bulk Dielectric Constant and Atomic Surface Tensions. *J. Phys. Chem. B* **2009**, *113*, 6378–6396.

21 CYLview, 1.0b; Legault, C. Y., Université de Sherbrooke, 2009 (http://www.cylview.org).

22 Bruker, 2010. Apex2. Bruker AXS Inc., Madison, Wisconsin, USA.

23 Krause, L.; Herbst-Irmer, R.; Sheldrick, G. M.; Stalke, D. *J. Appl. Cryst.* **2015**, *48*, 3–10.

24 Sheldrick, G. M. *Acta Cryst*. **2015**, *A17*, 3–8.

25 Sheldrick, G. M. *Acta Cryst*. **2015**, *C17*, 3–8.

26 Dolomanov, O. V.; Bourhis, L. J.; Gildea, R. J.; Howard, J. A. K.; Puschmann, H. *J. Appl. Cryst*. **2009**, *42*, 339–341.
